# Simvastatin inhibits PD-L1 via ILF3 to induce ferroptosis in gastric cancer cells

**DOI:** 10.1038/s41419-025-07562-8

**Published:** 2025-03-26

**Authors:** Danping Sun, Xiaohan Cui, Wenshuo Yang, Meng Wei, Zhibo Yan, Mingxiang Zhang, Wenbin Yu

**Affiliations:** 1 Department of Gastrointestinal Surgery, General Surgery, Qilu Hospital of Shandong University, 107 West Wen Hua Road, Jinan, 250012 China; 2 The Key Laboratory of Cardiovascular Remodeling and Function Research, Chinese Ministry of Education, Chinese Ministry of Health and Chinese Academy of Medical Sciences, Department of Cardiology, Qilu Hospital of Shandong University, 107 West Wen Hua Road, Jinan, 250012 China

**Keywords:** Cancer therapy, Gastrointestinal diseases

## Abstract

The treatment of gastric cancer remains challenging, with immunotherapy serving as a critical component of the holistic approach to its treatment. The results of this study indicated that statins could decrease the serum levels of interleukin-enhancing binding factor 3 (ILF3) and programmed cell death ligand 1(PD-L1) in GC patients and improve their prognosis. Functional experiments demonstrated that simvastatin induced ferroptosis by inhibiting ILF3 in GC cells and enhanced the killing effect of activated CD8^+^ T cells on GC cells. The CUT&Tag assay revealed that, mechanistically, simvastatin inhibited ILF3 expression by reducing the acetylation level at residue site H3K14 in ILF3. Next-generation sequencing and Kyoto Encyclopedia of Genes and Genomes analysis revealed that ILF3 regulated PD-L1 expression through the DEPTOR/mTOR signaling pathway. Overall, simvastatin induced ferroptosis in GC cells by inhibiting ILF3 expression while promoting the activation of CD8^+^ T cells to augment antitumor immune responses, thereby facilitating synergistic immunotherapy.

## Introduction

Gastric cancer (GC) is the fifth most prevalent malignancy worldwide and the fourth leading cause of cancer-related mortality, with an annual global incidence surpassing one million newly diagnosed cases [1]. Despite the implementation of a comprehensive treatment strategy primarily centered on surgical intervention supplemented by chemotherapy and immunotherapy, the five-year survival rate for patients with advanced GC remains below 30% [2, 3]. Importantly, chemotherapeutic resistance, recurrence, and distant metastases constitute the principal factors contributing to the failure of GC treatment [4, 5]. Therefore, there is an urgent need to identify novel mechanisms and therapeutic targets for diagnosing and treating GC to improve therapeutic outcomes.

As inhibitors of 3-hydroxy-3-methylglutaryl coenzyme A (HMGCR), statins are extensively utilized in clinical practice as lipid-lowering agents and inducers of ferroptosis [6, 7]. Statins may exert anticancer effects through multiple mechanisms, including inhibiting tumor proliferation by suppressing cholesterol synthesis, which alters cellular susceptibility to ferroptosis [8, 9]. In breast cancer, simvastatin inhibits HMGCR expression and suppresses the mevalonate (MVA) pathway and glutathione peroxidase 4 (GPX4) production, which induces cancer cell ferroptosis [10]. Rosuvastatin (RSV) encapsulated in silk fibroin nanoparticles, known as Cu-SF(RSV)NPs, increases breast cancer cell sensitivity to ferroptosis by inhibiting the CoQ/FSP1 axis [11]. Furthermore, statins are crucial for improving the effectiveness of immunotherapy for treating malignant tumors. Statins may synergize with programmed cell death protein-1 (PD-1) inhibitors to improve the prognosis of patients with malignant pleural mesothelioma and non-small cell lung cancer [12]. Moreover, RSV effectively hinders MVA-induced PD-L1 expression in various tumor cells, including colorectal cancer and melanoma cells [13]. However, research is needed to determine the specific molecular mechanisms of statins to potentially increase therapeutic efficacy in patients with GC.

The interleukin-enhancing binding factor 3 (ILF3) gene is located on chromosome 19 and encodes a double-stranded RNA-binding protein that regulates gene expression and stabilizes mRNA levels [14, 15]. Recently, increasing research on ILF3 has elucidated its role in fostering the progression of malignant tumors via the regulation of nutrient metabolism. For example, ILF3 promotes colorectal cancer (CRC) by stabilizing mRNA levels in the serine–glycine–one-carbon metabolic pathway [16]. Several pivotal metabolites within the glycolysis pathway are markedly diminished in esophageal cancer tissues with high ILF3 expression, leading to metabolic reprogramming and promoting esophageal cancer progression [17]. Moreover, ILF3 impedes the maturation of dendritic cells and restricts innate immune responses by regulating lipid metabolism [18]. Recent studies have demonstrated a significant association between ILF3 expression and immune cell infiltration. For example, ILF3 is positively correlated with PD-L1 expression in hepatocellular carcinoma, and the suppression of ILF3 reduces PD-L1 expression, consequently increasing the vulnerability of hepatocellular carcinoma to T-cell cytotoxicity [19]. A previous study revealed that statins inhibited ILF3 expression in GC cells [20]. Therefore, this study further investigated whether statins can suppress the expression of PD-L1 in GC cells through ILF3 to achieve therapeutic effects on GC.

The programmed cell death ligand 1(PD-L1) gene, also known as CD274, is a type I transmembrane immunoglobulin and is the primary ligand of PD-1 [21, 22]. PD-L1 is expressed in T cells, B cells, macrophages, mast cells, vascular endothelial cells, pancreatic islet cells, tumor cells, and astrocytes [23]. The immunosuppressive receptor PD-1 is expressed predominantly in activated T cells [24]. The immunosuppressive effects are exerted by PD-L1, which is expressed on the surface of cancer cells, through its binding to the PD-1 receptor on activated T cells [25, 26]. The PD-1/PD-L1 axis assumes a crucial function in facilitating the evasion of immune surveillance by malignant tumors and promoting their progression [23, 27]. Previous research has demonstrated that TRIM28 facilitates GC deterioration by suppressing PD-L1 ubiquitination and activating the TBKI-IRF1 and TBK1-mTOR signaling pathways, thereby increasing the stability and expression of PD-L1 [28]. Elevated PD-L1 expression is associated with resistance to radiotherapy and chemotherapy in lung cancer patients [29]. The knockdown of HnRNPL has been shown to initiate ferroptosis by activating Jurkat T cells via the suppression of PD-L1, increasing the recruitment of CD8^+^ T cells, and increasing the efficacy of anti-PD-L1 therapy [30]. Therefore, identifying prospective targets that regulate PD-L1 and impede its expression is important for enhancing immunotherapy efficacy.

This study demonstrated that statins effectively reduced the serum levels of PD-L1 and ILF3, thereby improving the prognosis for patients with GC. Specifically, simvastatin induced the overexpression of HDAC6 and reduced the acetylation level at the H3K14 residue in ILF3, leading to decreased ILF3 expression. The downregulation of ILF3 induced ferroptosis in GC cells by regulating SLC7A11/GPX4 through the DEPTOR/mTOR signaling pathway. Additionally, ILF3 facilitated the recruitment of activated CD8^+^ T cells by inhibiting PD-L1 expression, thus enhancing the cytotoxic efficacy of these cells against GC cells. These findings elucidate the substantial role of simvastatin in enhancing gastric cancer immunotherapy through ILF3, thereby providing a theoretical basis for the synergistic use of statins in immunotherapeutic applications.

## Materials and methods

### Patients and tissue specimens

Between January 2017 and December 2019, fundamental clinicopathological data, along with serum, gastric cancer tissue, and adjacent noncancerous tissues, were systematically collected from patients undergoing radical cancer surgery for gastric cancer at the Department of Gastrointestinal Surgery, General Surgery, Qilu Hospital of Shandong University, Jinan, China. GC patients who received statin therapy underwent treatment for more than six months. Furthermore, all participants enrolled in the study provided informed consent.

### Cell lines and cell culture

The GC cell lines (Ncl-N87 and HGC-27), in conjunction with mouse forestomach carcinoma (MFC) cells, were maintained in culture medium comprised of RPMI 1640 supplemented with 10% fetal bovine serum and 1% streptomycin-penicillin. The HEK293T cells were maintained in culture medium comprised of DMEM supplemented with 10% fetal bovine serum and 1% streptomycin-penicillin. The cells were incubated under controlled environmental conditions of 5% CO_2_ at 37 °C. All the cell lines were identified by short tandem repeat (STR) analysis and confirmed to be free of mycoplasma contamination.

### Transfection and sequences of small interfering RNAs (siRNAs)

Before transfection, GC cells were inoculated in a six-well plate and allowed to reach a confluence of 70%. The cells were subsequently transfected with Lipofectamine RNAiMax (13778150, Invitrogen, USA) to introduce siRNAs targeting various genes (Table S1). RNA extraction was performed 24 h post-transfection, while protein extraction was carried out 48 h post-transfection for subsequent experiments.

### Construction and transfection of overexpression plasmids

To investigate the overexpression of ILF3 and PD-L1 in GC cells, the genes HOMO-ILF3-C-3xFLAG and HOMO-PD-L1-C-3xFLAG were cloned and inserted into the pcDNA3.1 vector to produce the pcDNA3.1-ILF3 and pcDNA3.1-PD-L1 plasmids. GC cells were cultured in six-well plates for 24 h before transfection. Transfection was carried out for 24 h with Lipofectamine 3000 (L3000075, Invitrogen, USA) and P3000 (L3000075, Invitrogen, USA) transfection reagents mixed with OPTI-MEM (Gibco, Shanghai, China).

### Western blotting (WB)

Whole-cell proteins were extracted from GC tissues and cells with RIPA lysis buffer (P0013B, Beyotime, China) supplemented with PMSF (P0100, Solarbio, China) and a protein phosphatase inhibitor (P1260, Solarbio, China). Protein samples were separated by 10% SDS PAGE and transferred to PVDF membranes (200 mA for 90 min). The proteins on the PVDF membrane were blocked with 5% skim milk (1 h at room temperature), followed by an overnight incubation (4 °C) with the primary antibody. The proteins on the PVDF membrane were subsequently incubated with either a secondary rabbit antibody (ZB-2301, ZSGB-BIO, China) or a mouse antibody (ZB-2305, ZSGB-BIO, China) (1 h at room temperature). The primary antibodies used were against ILF3 (ab92355, Abcam, UK), SLC7A11 (ab307601, Abcam, UK), GPX4 (ab252833, Abcam, UK), GAPDH (ABclonal, A19056), PCNA (ab92552, Abcam, UK), HDAC1 (A19571, ABclonal, China), HDAC2 (A19626, ABclonal, China), HDAC6 (A3572, ABclonal, China), DEPTOR (A9447, ABclonal, China), mTOR (2972S, Cell Signaling Technology, USA), p-mTOR (5536S, Cell Signaling Technology, USA), H3K14ac (A7254, ABclonal, China), PD-L1 (66248-1-Ig, Proteintech, China), CD8 (66868-1-Ig, Proteintech, China), pan acetyl lysine (PTM-105, PTM Bio, China), and 4-hydroxynonenal (4-HNE) (bs-6313R, Bioss, China). The relative expression of proteins was determined with an Amersham Imager 680 (Marlborough, USA) and a Tanon 4800 (Shanghai, China).

### RNA isolation and quantitative real-time polymerase chain reaction (qRT‒PCR)

Total RNA from pretreated GC cells was extracted with an RNAfast200 kit (220010, Fastagen, China). The extracted RNA was reverse transcribed into cDNA with HiScript III RT SuperMix for qPCR (R323-01, Vazyme, China), and cDNA was used to perform qRT‒PCR with the ChamQ Universal SYBR qPCR Master Mix (Q711-02, Vazyme, China). The designed primer sequences are shown in Table S2.

### Immunohistochemistry (IHC) staining

IHC staining was performed as previously described [20]. IHC staining was performed with primary antibodies against ILF3, DEPTOR, 4NHE, PD-L1, and CD8. Analysis was performed with ImageJ software, which integrated the mean gray value (representing the staining intensity) and the proportion of positively stained cells (defining the degree of staining coverage) as critical metrics for IHC staining measurements. The scoring system for the IHC staining results was structured into four distinct categories: a score of 1 denoted a negative result, 2 indicated a low level of positivity, 3 was assigned for a moderate positivity, and 4 signified a high level of positivity. The corresponding H-scores were plotted on histograms with statistics.

### Isolation and culture of human CD8^+^ T cells

Venous blood was collected, and peripheral blood mononuclear cells (PBMCs) were isolated by Ficoll density gradient centrifugation. CD8^+^ T cells were isolated and purified with the Dynabeads^®^ Untouched™ Human CD8^+^ T Cell Kit (11348D, Thermo Fisher, USA). The anti-CD3 antibody was diluted with phosphate-buffered saline (PBS) to a concentration of 5 μg/ml. A volume (500 μl) of the diluted antibody was subsequently added to each well of a 6-well plate and incubated overnight at 4 °C. Simultaneously, CD8^+^ T cells were activated and cultured in complete RPMI 1640 medium supplemented with anti-CD28 antibody (5 μg/ml) and IL-2 (10 ng/ml) in each well. The medium was changed every 48 h, and the cell condition and proliferation were observed. GC cells were cocultivated with activated CD8^+^ T cells at a 1:6 ratio in 6-well plates (48 h).

### Enzyme-linked immunosorbent assay (ELISA)

Following the knockdown and overexpression of ILF3 in GC cells, the levels of PD-L1 secreted into the culture medium were quantified with an ELISA kit (Elabscience, China). The experimental procedure followed the manufacturer’s instructions, and the absorbance at 450 nm was measured with an enzyme labeler.

### Methyl thiazolyl tetrazolium (MTT) cytotoxicity determination

A 5 × 10^4^ cell/ml suspension of GC cell in 100 μl was added to each well of a 96-well plate. After 24 h of stimulation with different concentrations of drugs, 10 μl of MTT solution (C0009S, Beyotime, China) was added to each well, and incubation was continued for 4 h. To each well was added 100 μl of formazan solution, followed by mixing well and incubation for 3 h. At the end of the incubation, the absorbance value of each well was measured at 570 nm with an enzyme labeler.

### Calcein AM/PI cell viability assay

After GC cells were inoculated into 12-well cell plates, various experimental treatments were applied for 24 h. Subsequently, the cells were stained with calcein AM and propidium iodide (PI) (C2015M, Beyotime, China) (30 min at 37 °C in the dark). Finally, a fluorescence microscope was used to observe the green fluorescence of live cells and the red fluorescence of dead cells.

### Transmission electron microscopy (TEM)

The GC cells in the pretreated 6-well plate were incubated with electron microscope fixative, fixed at room temperature, and protected from light for 5 min. Then, the cells were gently scraped off in one direction with a cell scraper and transferred to a centrifuge tube. The GC cell precipitate was collected after centrifugation (1000 rpm/min for 5 min). After the fixative was discarded and the cells were resuspended in a new electron microscope fixative, the cells were fixed (room temperature in dark conditions for 30 min). Servicebio (Wuhan, China) conducted the TEM imaging.

### Intracellular reactive oxygen species (ROS) determination

After different pretreatments, a ROS assay kit (S0033S, Beyotime, China) was used to determine the ROS levels in the GC cells. The DCFH-DA stock solution was diluted at a ratio of 1:1000, added to a 12-well plate, and incubated (37 °C for 30 min in the dark). At the end of incubation, the ROS level was determined by washing with HBSS 3 times, after which the samples were observed and photographed under a fluorescence microscope.

### Intracellular Fe^2+^ determination

After different pretreatments, a FerroOrange assay kit (F374, Dojindo, China) was used to determine the Fe^2+^ levels in GC cells. FerroOrange solution was added to a twelve-well plate at a 1 mmol/L concentration. The intracellular Fe^2+^ level was subsequently determined by observation and imaging under a fluorescence microscope after incubation (in the dark for 30 min).

### Malondialdehyde (MDA) determination

A lipid peroxidation MDA assay kit (S0131M, Beyotime, China) was used to determine the content of MDA in GC cells subjected to different pretreatments. The MDA content reflects the level of lipid oxidation. The samples to be tested and the MDA working solution were mixed and heated (100 °C for 15 min), allowed to cool to room temperature, and then centrifuged (1000 × g for 10 min). A volume (200 μl) of the supernatant was added to a 96-well plate, and the absorbance value at 532 nm was measured. The amount of MDA per unit weight of protein was calculated.

### Glutathione (GSH)/oxidized glutathione (GSSG) determination

After different pretreatments, a GSH and GSSG assay kit (S0053, Beyotime, China) was used to determine the GSH/GSSG levels in GC cells. The experimental procedure followed the manufacturer’s instructions, and the absorbance at 412 nm was measured with an enzyme labeler.

### Lipid peroxide (LPO) determination

After different pretreatments, an LPO assay kit (L248, Dojindo, China) was used to measure LPO levels in GC cells. After the pretreated GC cells were washed once with HBSS, an appropriate 10 µmol/L Liperfluo working solution was added, and the mixture was incubated (30 min for 37 °C). The cells were washed twice more at the end of the incubation period. Trypsin-digested cells were collected by centrifugation and resuspended, and the intracellular LPO levels were determined by flow cytometry at 488 nm.

### Analysis of cell death

GC cells were cocultured with activated CD8^+^ T cells, followed by digestion and resuspension in 1× binding buffer to achieve a cell concentration of 1 × 10^6^ cells/ml. An Annexin V-PI staining kit (556547a, BD Pharmingen™, USA) was used to assess apoptosis in the GC cells, which were incubated with the kit reagents at room temperature in the dark for 15 min. Cell death was subsequently assessed by flow cytometry with a BD FACSCalibur system.

### Crystal violet staining assay

Various groups of GC cells were cocultivated alongside CD8^+^ T cells for 48 h. The supernatant was removed, and the GC cells were immobilized by the addition of 4% paraformaldehyde for 15 min. After the supernatant was removed, the GC cells were stained with 0.1% crystal violet solution, after which images were captured.

### Cut&Tag experiment

The Hyperactive Universal Cut and Tag Assay Kit for Illumina Pro (TD904, Vazyme, China) was used for subsequent experiments. Pretreated GC cells were collected, ensuring 100,000 cells per sample. The collected cells were incubated with activated ConA Beads Pro for 10 min at room temperature. At the end of the incubation, the supernatant was discarded. After the cells were resuspended again, they were incubated with a primary antibody against H3K14ac at 4 °C overnight. The secondary antibody diluted at a 1:100 ratio with Dig-wash Buffer was added and incubated for 60 min at room temperature with rotation. The samples were washed 3 times at the end of the incubation. Then, the samples were washed 3 times after rotary incubation with pA/G-Tnp Pro at room temperature for 60 min. Diluted TTBL was then added, and the mixture was incubated (37 °C for 60 min). SDS (10%) and the appropriate amount of DNA were added, and the mixture was mixed well and incubated at 55 °C for 10 min before the supernatant was collected. Activated DNA Extra Beads Pro were added to the collected supernatant, which was subsequently incubated for 20 min at room temperature. The supernatant was discarded after incubation with 1× B&W Buffer for 30 s at room temperature, and this process was repeated once. The DNA Extra Beads Pro were resuspended by adding another 15 μl of ddH_2_O, and the samples were subjected to PCR amplification. The PCR products were purified and sequenced on the Illumina platform.

### Luciferase activity assay

To investigate the binding site of ILF3 on the DEPTOR promoter, a series of pGL3-DEPTOR-Luc reporter plasmids featuring progressively truncated 5′-flanking regions spanning from the −1300 to +300 bp region were constructed. Promoter regions of varying lengths of the DEPTOR sequence were constructed, specifically encompassing the following ranges: −1300 to −1100, −1100 to −900, −900 to −700, −700 to −500, −500 to −300, −300 to −100, −100 to +100, and +100 to +300 bp. HEK293T cells were cultured in 24-well plates and cotransfected with luciferase reporter plasmids and a Renilla reporter plasmid. In addition, HEK293T cells cultured in 24-well plates were transfected with either the wild-type or mutant DEPTOR promoter region in conjunction with an ILF3 overexpression vector. After 48 h, the firefly and Renilla luciferase signals were quantified with the Dual Luciferase Reporter Gene Assay Kit (11402ES60, Yeasen, China).

### Lentivirus production and construction of stable GC and MFC cell lines

The lentiviral vector was successfully constructed at Nanjing Corues Biotechnology. The virus was obtained from the transfected HEK293T cells, after which the GC and MFC cells were exposed to the viral supernatants for 48 h. The GC and MFC cells that underwent transfection were subjected to puromycin screening at a concentration of 2 µg/ml for 24 h to establish stable GC and MFC cell lines capable of expressing ILF3.

### Animal experiments

All the mice were housed in a standardized pathogen-free (SPF) animal facility and provided adequate water and food. All the mice were euthanized by CO_2_ asphyxiation at the end of the experiment. Stable ILF3 overexpression and silencing (con-ILF3, oe-ILF3, nc-ILF3, and sh-ILF3) were established in GC cells by lentivirus-mediated techniques, followed by in vivo experiments to study the effect of ILF3 expression on tumor growth. Moreover, stable ILF3 overexpression (con-ILF3, oe-ILF3) was established in MFC cells by lentivirus-mediated techniques. A subcutaneous tumor model was constructed with 4-week-old nude mice and 615 mice. Twenty-four nude mice were randomly divided into 6 groups, and each group was injected with 1 × 10^7^GC cells subcutaneously in the left axilla. Sixteen 615 mice were randomly divided into 4 groups, and each group was injected with 1 × 10^7^MFC cells subcutaneously in the left axilla. Tumor size was measured, and tumor volume was calculated every 4 days. After 3 weeks, the tumors were removed to calculate the final volume and weight, and growth curves and weight histograms were generated.

### Quantification and statistical analysis

SPSS and GraphPad Prism 8.0 were used for statistical data analysis. The experimental data are presented as the means ± SDs of three independent replicates. Unpaired two-tailed Student’s t tests were used to analyze differences between two groups. Survival curves were constructed with the Kaplan‒Meier method, and the log-rank test was used to assess the statistical significance of differences between survival curves. Count data were analyzed by Pearson’s chi-square test. A *P*-value < 0.05 was considered statistically significant.

## Results

### Statins targeted ILF3 to increase the chemosensitivity of GC patients

**Fig. 1:**
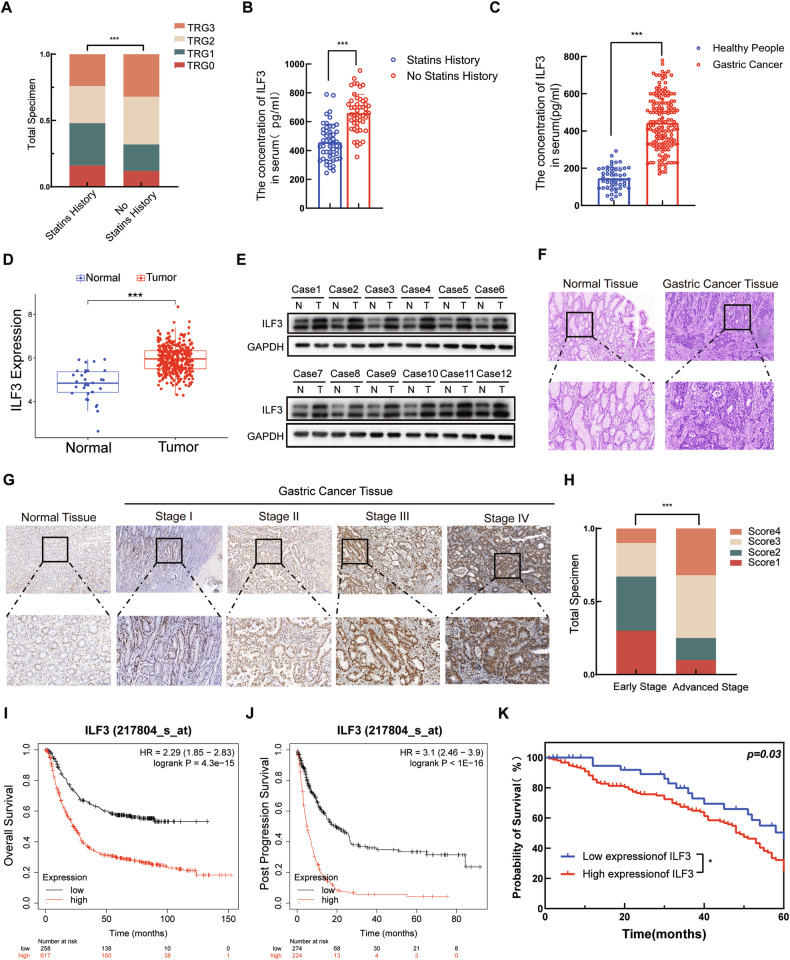
Statins targeted ILF3 to increase the chemosensitivity of GC patients. **A** TRG-graded histogram of preoperative patients with neoadjuvant GC with or without statin treatment. **B** Serum levels of ILF3 in patients with GC treated with or without statins. **C** Serum levels of ILF3 in healthy individuals and GC patients. **D** Differences in ILF3 transcription levels between GC and normal gastric tissues in the TCGA database. **E** WB analysis of the expression of ILF3 in GC and normal gastric tissues. **F** HE staining of GC and normal gastric tissues. **G** ILF3 expression levels in normal gastric tissues and GC tissues from patients with different stages of GC were determined by IHC staining. **H** Relationship between clinical stage and ILF3 expression in GC patients. **I**, **J** Relationships between ILF3 expression and the OS and PPS of GC patients were assessed via K‒M analysis. **K** Probability of survival of GC patients in cohort 1 and cohort 2 with low and high ILF3 expression levels.

GC patients who underwent preoperative neoadjuvant chemotherapy were divided into two groups: those with and without statins. The Tumor Regression Grading (TRG) system (Table S3) is commonly used to evaluate the treatment response of GC patients subjected to neoadjuvant therapies, typically chemotherapy or radiotherapy [31]. TRG grading of the postoperative pathology revealed that the statin-treated group exhibited more prominent tumor regression and was more sensitive to chemotherapy (Fig. 1A). The serum ILF3 level was significantly lower in the statin-treated group (Fig. 1B), and the corresponding clinical information is provided in Table S4. Compared with that in healthy individuals, the serum ILF3 level was considerably elevated in GC patients (Fig. 1C), and the corresponding clinical data are detailed in Table S5. Analysis of data from The Cancer Genome Atlas (TCGA) revealed that the mRNA level of ILF3 was greater in GC tissues than in normal tissues (Fig. 1D). The results from clinical samples from Qilu Hospital revealed that ILF3 expression was elevated in GC tissues compared with that in normal gastric tissues, which aligns with TCGA outcomes (Fig. 1E). ILF3 expression was compared between different GC stages and gastric tissues by hematoxylin‒eosin (HE) staining and IHC staining (Fig. 1F, G). These results revealed that ILF3 expression in advanced-stage GC patients surpassed that in early-stage GC patients (Fig. 1H). The Kaplan‒Meier (K‒M) plot revealed that elevated ILF3 expression was associated with poorer overall survival (OS) and postprogression survival (PPS) (Fig. 1I, J). Analysis of the clinical samples revealed that high ILF3 expression in GC patients was associated with a significantly poorer OS (Fig. 1K).

### ILF3 inhibited the expression of PD-L1 and enhanced the killing effect of activated CD8^+^ T cells on GC cells

**Fig. 2:**
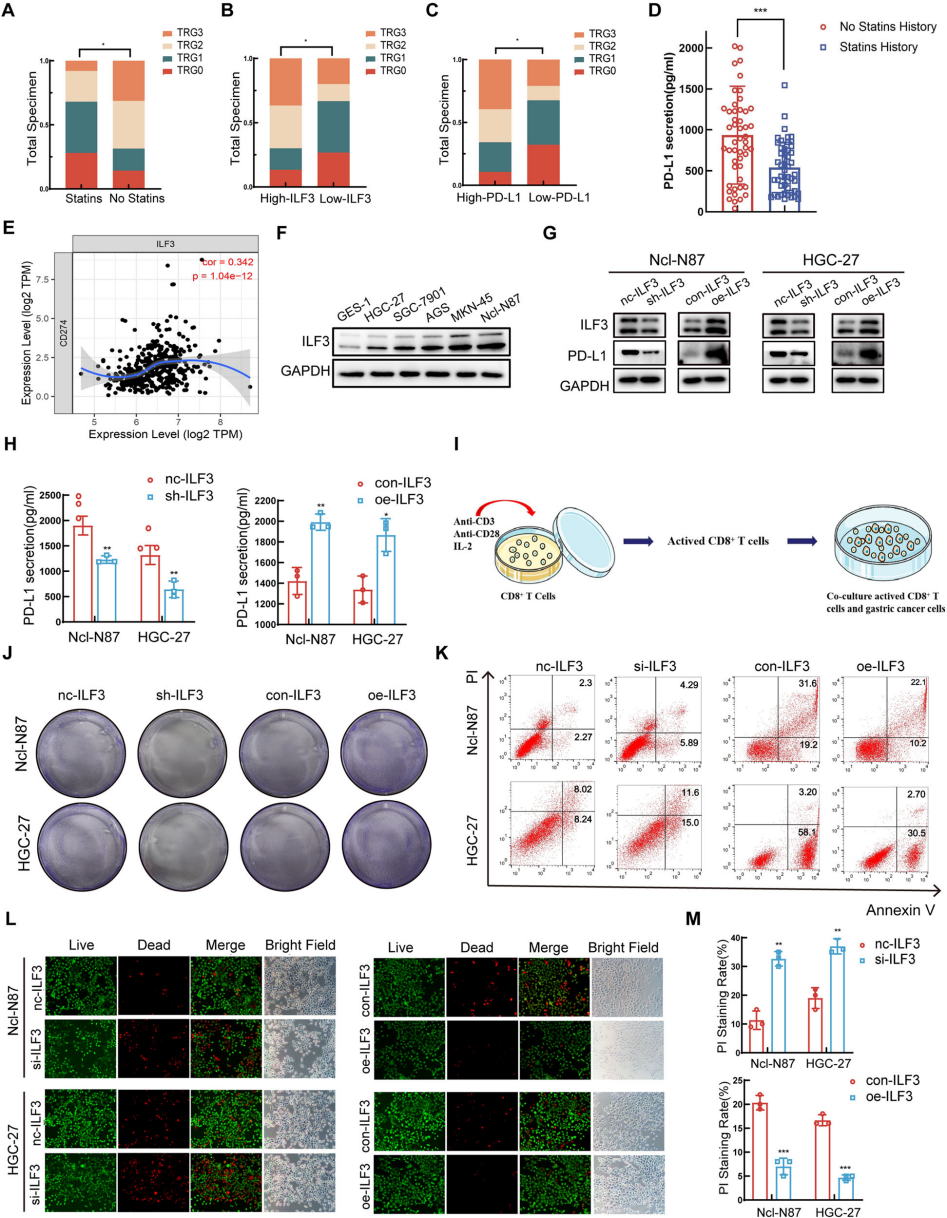
ILF3 inhibited the expression of PD-L1 and enhanced the killing effect of activated CD8^+^ T cells on GC cells. TRG-graded histogram of preoperative neoadjuvant and immunotherapy-treated GC patients according to (**A**) treatment with or without statins, **(B**) high or low expression of ILF3, and **(C**) high or low expression of PD-L1. **D** Serum levels of PD-L1 in patients with GC treated with preoperative neoadjuvant chemotherapy and immunotherapy with or without statin treatment. **E** Correlation between ILF3 and PD-L1 expression in GC tissues from TIMER. **F** WB analysis of ILF3 expression in GES-1 and GC cell lines. **G** The protein levels of PD-L1 following the knockdown or overexpression of ILF3 were measured via WB. **H** The level of PD-L1 secreted from GC cells following the knockdown or overexpression of ILF3 was determined via ELISA. **I** Schematic diagram of activated CD8^+^ T cells cocultured with GC cells. **J** GC cells with ILF3 knockdown or overexpression were cocultured with activated CD8^+^ T cells, and the surviving GC cells were analyzed by crystal violet staining. **K** GC cells with ILF3 knockdown or overexpression were cocultured with activated CD8^+^ T cells, and the number of dead GC cells was analyzed by flow cytometry. **L**, **M** The killing effect of activated CD8^+^ T cells on GC cells following the knockdown or overexpression of ILF3 was analyzed by calcein/PI staining.

GC patients who underwent preoperative neoadjuvant chemotherapy and immunotherapy were divided into two groups on the basis of their treatment with statins and the expression levels of ILF3 and PD-L1 (Tables S6–8). The results revealed that the statin-treated group (Fig. 2A) and the low-ILF3 (Fig. 2B) and low-PD-L1 (Fig. 2C) expression groups exhibited more prominent tumor regression and were more sensitive to neoadjuvant chemotherapy on the basis of TRG grading. The serum PD-L1 level was significantly reduced in the statin-treated group (Fig. 2D). Tumor Immune Estimation Resource (TIMER) analysis revealed that ILF3 expression was positively correlated with PD-L1 expression in GC tissues (Fig. 2E). The expression of ILF3 was greater in various GC cell lines than in gastric epithelium cells (GES-1) (Fig. 2F). WB revealed that PD-L1 expression levels were decreased following ILF3 knockdown (sh-ILF3) and increased following ILF3 overexpression (oe-ILF3) in GC cells (Fig. 2G). Previous studies have shown that tumor cells release PD-L1-positive extracellular vesicles to evade the immune system [27]. The ELISA results demonstrated that the knockdown of ILF3 reduced the secretion of PD-L1 and that the overexpression of ILF3 increased the secretion of PD-L1 (Fig. 2H). CD8^+^ T cells were activated by costimulation with anti-CD3 and anti-CD28 antibodies and with IL-2 (Fig. 2I). Activated CD8^+^ T cells were cocultured with GC cells for 48 h, and crystal violet staining revealed that the number of viable GC cells decreased in the sh-ILF3 group and that the number of viable GC cells increased in the oe-ILF3 group (Fig. 2J, Figure S1A). Flow cytometry analysis revealed that si-ILF3 increased the percentage of dead GC cells, whereas oe-ILF3 decreased the percentage of dead GC cells (Fig. 2K, Figure S1B). Calcein/PI staining revealed the killing effect of activated CD8^+^ T cells on GC cells with a fluorescence microscopy, which revealed that si-ILF3 enhanced the killing effect of activated CD8^+^ T cells on GC cells, whereas oe-ILF3 had the opposite effect (Fig. 2L, M). TIMER analysis revealed elevated PD-L1 expression in multiple malignancies. Among these malignancies, the expression of PD-L1 was upregulated in GC tissues (Figure S1C). Further investigation revealed that elevated PD-L1 was associated with a poorer OS among cancer patients (Figure S1D). TCGA analysis revealed that the mRNA level of PD-L1 was greater in GC tissues than in normal gastric tissues (Figure S1E). Compared with that in normal gastric tissues, PD-L1 expression in GC tissues was elevated at both the mRNA and protein levels in samples donated from Qilu Hospital (Figure S1F, G). IHC staining revealed that PD-L1 expression was elevated in GC tissues compared with paracancerous tissues and that a high expression of PD-L1 was associated with a low expression of CD8, which inhibited the infiltration of CD8^+^ T cells in GC tissues (Figure S1H).

### Simvastatin suppressed ILF3 expression and induced ferroptosis in GC cells

**Fig. 3:**
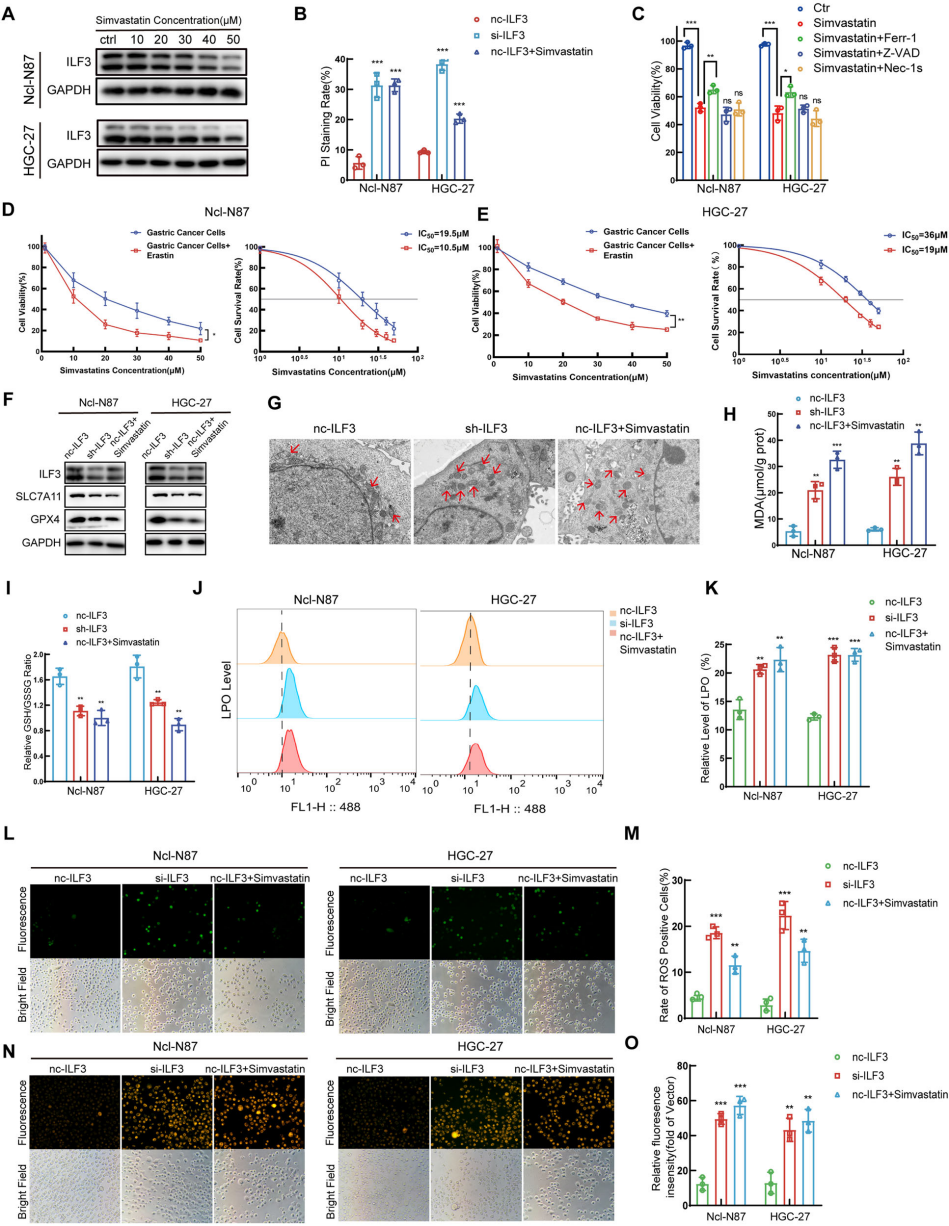
Simvastatin suppressed ILF3 expression and induced ferroptosis in GC cells. **A** The protein level of ILF3 in GC cells treated with simvastatin was analyzed by WB. **B** The percentage of dead GC cells in the ILF3-knockdown and simvastatin stimulation groups was analyzed by calcein/PI staining. **C** MTT assay analysis of the viability of GC cells stimulated with simvastatin and treated with Ferr-1, Z-VAD, or Nec-1s. **D**, **E** Cell activity and IC_50_ values of GC cells treated with simvastatin and cocultured with erastin were determined by MTT assays. **F** The protein levels of ILF3, SLC7A11, and GPX4 in GC cells in the ILF3 knockdown and simvastatin stimulation groups were analyzed by WB. **G** Morphology of mitochondria observed by electron microscopy. The MDA (**H**) and relative GSH/GSSG (**I**) levels were determined in the ILF3 knockdown and simvastatin stimulation groups. **J**, **K** LPO levels were determined in the ILF3 knockdown and simvastatin stimulation groups. The ROS (**L**, **M**) and Fe^2+^ (**N**, **O**) levels in the ILF3 knockdown and simvastatin stimulation groups were determined by fluorescence microscopy.

Simvastatin, a lipophilic statin with enhanced lipid solubility, facilitates its permeation across cell membranes, increasing the likelihood of exerting anticancer effects [32]. WB analyses revealed a concentration-dependent suppression of ILF3 expression at the protein level by simvastatin (Fig. 3A). Calcein/PI staining revealed a greater percentage of dead GC cells in the si-ILF3 and simvastatin-stimulated groups than in the nc-ILF3 group (Fig. 3B, Figure S2A, B). Next, simvastatin stimulation or knockdown of ILF3 in GC cells was paired with various inhibitors, including ferrostatin-1 (Ferr-1; a ferroptosis inhibitor), Z-VAD-FMK (Z-VAD; an apoptosis inhibitor), and necrostatin-1s (Nec-1s; a necrosis inhibitor). MTT assays revealed a significant increase in cell activity only when the GC cells were costimulated with the ferroptosis inhibitor Ferr-1 (Fig. 3C, Figure S2C). The GC cells were segregated into two groups: one group was exposed to varying concentrations of simvastatin, and the other was exposed to costimulation with simvastatin and erastin. The results of the MTT assay indicated that the IC_50_ values for simvastatin and the costimulation of Ncl-N87 cells with simvastatin and erastin were 19.5 μM and 10.5 μM, respectively. The IC_50_ values in HGC-27 cells were 36 μM and 19 μM, respectively (Fig. 3D, E). These results suggested that erastin may act synergistically with simvastatin to inhibit the activity of GC cells and increase their sensitivity to simvastatin. SLC7A11 and GPX4 were identified as potential targets of ILF3 on the basis of data from the hTFtarget database (Table S9). WB revealed that the protein expression levels of SLC7A11 and GPX4 were decreased after sh-ILF3 and simvastatin stimulation in GC cells (Fig. 3F). A crucial characteristic of ferroptosis involves the diminishment or elimination of mitochondrial cristae, rupture of the outer mitochondrial membrane, and its subsequent crumpling [33]. The diminishment of mitochondrial cristae and rupture of the outer mitochondrial membrane were observed by TEM in GC cells of the sh-ILF3 and simvastatin stimulation group (Fig. 3G, Figure S2D). The levels of MDA were elevated, and the GSH/GSSG levels were decreased in the sh-ILF3 and simvastatin stimulation group (Fig. 3H, I). Flow cytometry analysis revealed an increased level of LPO (Fig. 3J, K), and fluorescence microscopy revealed increased levels of ROS (Fig. 3L, M) and Fe^2+^ (Fig. 3N, O) in the si-ILF3 and simvastatin stimulation group.

A rescue experiment was conducted to verify the induction of ferroptosis in GC cells by simvastatin through the inhibition of ILF3. WB revealed that the protein levels of ILF3, SLC7A11, and GPX4 were lower in the oe-ILF3+simvastatin group than in the oe-ILF3 group (Figure S2E). Calcein/PI staining revealed that, compared with oe-ILF3 alone, oe-ILF3+simvastatin enhanced the cytotoxicity of simvastatin in GC cells (Figure S2F, G). The levels of MDA were elevated, and the GSH/GSSG levels were decreased in the oe-ILF3+simvastatin group compared with those in the oe-ILF3 group (Figure S2H, I). Fluorescence microscopy revealed increased levels of ROS (Figure S2J, K) and Fe^2+^ (Figure S2L, M), and flow cytometry analysis revealed an increased level of LPO (Figure S2N, O) in the oe-ILF3+simvastatin group compared with that in the oe-ILF3 group.

### Simvastatin affected activated CD8^+^ T-cell-mediated killing of GC cells by inhibiting PD-L1 expression

**Fig. 4:**
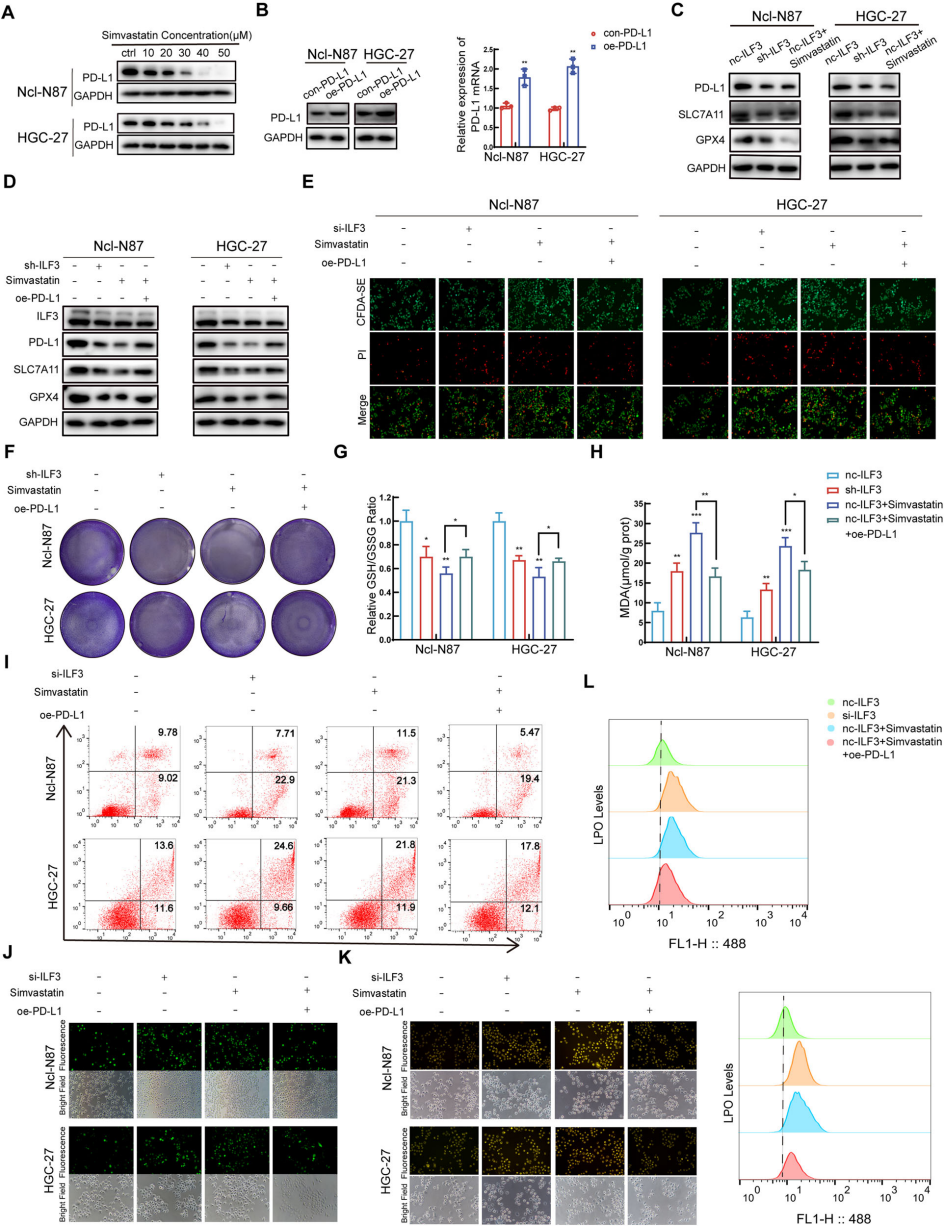
Simvastatin affected activated CD8^+^ T-cell-mediated killing of GC cells by inhibiting PD-L1 expression. **A** The protein level of PD-L1 in GC cells treated with simvastatin was analyzed by WB. **B** The protein and mRNA levels of PD-L1 in GC cells after transfection with overexpression plasmids. **C** The protein levels of PD-L1, SLC7A11, and GPX4 in GC cells following ILF3 knockdown or simvastatin stimulation. **D** The protein levels of ILF3, PD-L1, SLC7A11, and GPX4 in GC cells in the nc-ILF3, sh-ILF3, nc-ILF3+simvastatin, and nc-ILF3+simvastatin+oe-PD-L1 groups were analyzed by WB. **E** The killing effect of activated CD8^+^ T cells on GC cells in the nc-ILF3, si-ILF3, and nc-ILF3+simvastatin+oe-PD-L1 groups was analyzed by CFDA-SE/PI staining. **F** Surviving GC cells cocultured with activated CD8^+^ T cells in the nc-ILF3, sh-ILF3, and nc-ILF3+simvastatin+oe-PD-L1 groups were visualized by crystal violet staining. The relative GSH/GSSG (**G**) and MDA (**H**) levels were determined in the nc-ILF3, sh-ILF3, and nc-ILF3+simvastatin+oe-PD-L1 groups cocultured with activated CD8^+^ T cells. **I** The percentages of dead GC cells cocultured with activated CD8^+^ T cells in the nc-ILF3, si-ILF3, and nc-ILF3+simvastatin+oe-PD-L1 groups were analyzed by flow cytometry. The ROS (**J**) and Fe^2+^ (**K**) levels in the nc-ILF3, si-ILF3, and nc-ILF3+simvastatin+oe-PD-L1 groups cocultured with activated CD8^+^ T cells were determined by fluorescence microscopy. LPO (**L**) levels were determined in the nc-ILF3, si-ILF3, and nc-ILF3+simvastatin+oe-PD-L1 groups cocultured with activated CD8^+^ T cells.

The WB results revealed that simvastatin inhibited the protein expression of PD-L1 in GC cells in a concentration-dependent manner (Fig. 4A). GC cells were transfected with plasmids to overexpress PD-L1 at the protein and mRNA levels (Fig. 4B). WB revealed that PD-L1, SLC7A11, and GPX4 expression was reduced in the sh-ILF3- and simvastatin-stimulated groups of GC cells cocultured with activated CD8^+^ T cells (Fig. 4C). WB revealed that the protein levels of ILF3, PD-L1, SLC7A11, and GPX4 were greater in the simvastatin+oe-PD-L1 group than in the simvastatin stimulation groups of GC cells when cocultured with activated CD8^+^ T cells (Fig. 4D). CFDA-SE/PI staining revealed that oe-PD-L1 reduced simvastatin-induced activated CD8^+^ T-cell killing of GC cells to some extent (Fig. 4E, Figure S3A). Crystal violet staining revealed that the number of viable GC cells was greater in the simvastatin+oe-PD-L1 group than in the simvastatin stimulation groups of GC cells when cocultured with activated CD8^+^ T cells (Fig. 4F, Figure S3B). Previous studies demonstrated that the inhibition of PD-L1 promoted CD8^+^ T cells to induce ferroptosis in tumor cells, providing a new direction for the immunotherapy of malignant tumors [34, 35]. Moreover, activated CD8^+^ T cells secreted interferon γ (IFN-γ), which inhibits GPX4 and other GSH-dependent enzymes, leading to ferroptosis in cancer cells [36]. Increased GSH/GSSG (Fig. 4G) and decreased MDA (Fig. 4H) levels were detected in GC cells in the simvastatin+oe-PD-L1 group compared with those in the stimulation groups of GC cells when cocultured with activated CD8^+^ T cells. Flow cytometry analysis revealed that when cocultured with activated CD8^+^ T cells, the simvastatin+oe-PD-L1 group had a decreased percentage of dead GC cells compared with the simvastatin stimulation groups of GC cells (Fig. 4I, Figure S3C). The levels of ROS (Fig. 4J, Figure S3D) and Fe^2+^ (Fig. 4K, Figure S3E) were lower in the simvastatin+oe-PD-L1 group than in the simvastatin stimulation groups of GC cells when cocultured with activated CD8^+^ T cells. Flow cytometry analysis revealed a decreased level of LPO in the simvastatin+oe-PD-L1 group compared with the simvastatin stimulation groups of GC cells when cocultured with activated CD8^+^ T cells (Fig. 4L, Figure S3F).

### Simvastatin suppressed ILF3 expression by decreasing H3K14ac levels

**Fig. 5:**
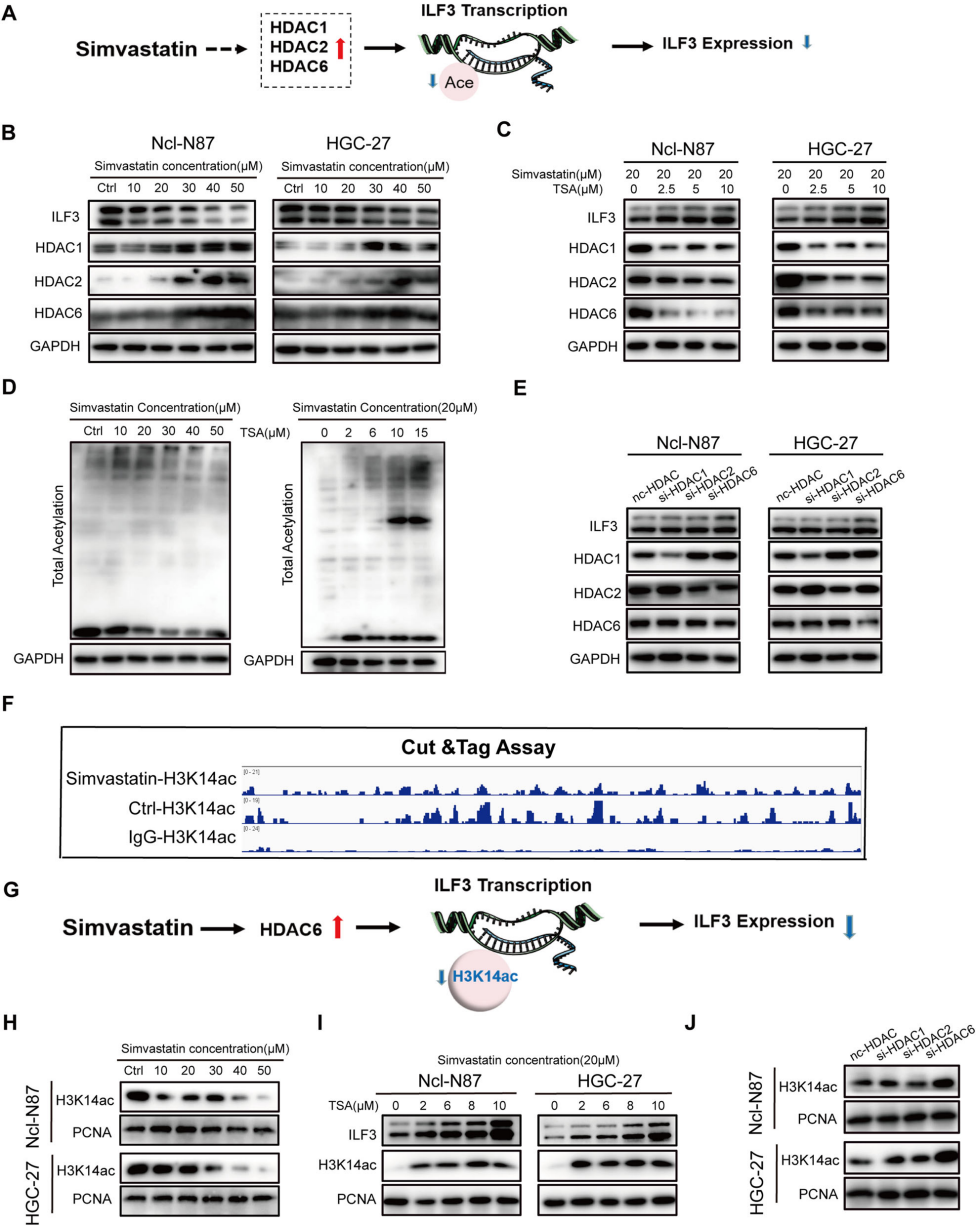
Simvastatin suppressed ILF3 expression by decreasing H3K14ac levels. **A** Schematic diagram of the potential mechanism of by which simvastatin regulates ILF3 expression. **B** WB analysis of ILF3, HDAC1, HDAC2, and HDAC6 protein levels in GC cells treated with simvastatin. **C** The protein levels of ILF3, HDAC1, HDAC2, and HDAC6 in GC cells treated with simvastatin and TSA were analyzed by WB. **D** Levels of total acetylation in GC cells treated with simvastatin only and costimulated with simvastatin and TSA, as determined by WB. **E** WB determination of the protein levels of ILF3, HDAC1, HDAC2, and HDAC6 after the knockdown of HDAC1, HDAC2, and HDAC6 with small interfering RNAs. **F** Results of the Cut&Tag assay. **G** Schematic diagram of the mechanism by which simvastatin inhibits ILF3 expression. **H** WB analysis of the acetylation level of the H3K14 site in GC cells treated with simvastatin. **I** WB analysis of the acetylation level of the H3K14 site in GC cells treated with simvastatin and TSA. **J** WB determination of the acetylation levels of the H3K14 site after knockdown of HDAC1, HDAC2, and HDAC6 with small interfering RNAs.

Swiss Target Prediction was used to predict potential targets of simvastatin by analysis of the drug structure, which revealed that HDAC1, HDAC2, and HDAC6 could be targeted by simvastatin (Table S10). These findings indicated that simvastatin might inhibit ILF3 expression by inducing the overexpression of the HDAC family and subsequently reducing ILF3 histone acetylation (Fig. 5A). WB revealed that the protein levels of HDAC1, HDAC2, and HDAC6 increased and that the protein level of ILF3 decreased after stimulation with different concentrations of simvastatin (Fig. 5B). Trichostatin (TSA) is a repressor of the HDAC family that induces increased histone acetylation and promotes gene transcription [37]. WB revealed that the protein levels of HDAC1, HDAC2, and HDAC6 decreased and that the protein level of ILF3 increased after costimulation with different concentrations of simvastatin and TSA (Fig. 5C). Moreover, the total acetylation level in GC cells decreased under simvastatin stimulation, and TSA increased the total acetylation level (Fig. 5D). WB revealed that only the knockdown of HDAC6 (si-HDAC6) increased the protein level of ILF3 (Fig. 5E). The Cut&Tag assay revealed reduced acetylation at the H3K14 site in GC cells stimulated with simvastatin (Fig. 5F). Simvastatin might inhibit ILF3 expression by inducing HDAC6 overexpression and reducing the acetylation level at the H3K14 residue in ILF3 (Fig. 5G). WB revealed a decrease in acetylation at the H3K14 site in GC cells treated with varying concentrations of simvastatin (Fig. 5H). Additionally, stimulation with simvastatin (20 µM) in combination with stimulation with different concentrations of TSA resulted in a progressive increase in acetylation at the H3K14 site (Fig. 5I). WB revealed that acetylation of the H3K14 site of ILF3 in the si-HDAC6 group was increased (Fig. 5J).

### ILF3 regulated PD-L1 expression and ferroptosis through the DEPTOR/mTOR signaling pathway

**Fig. 6:**
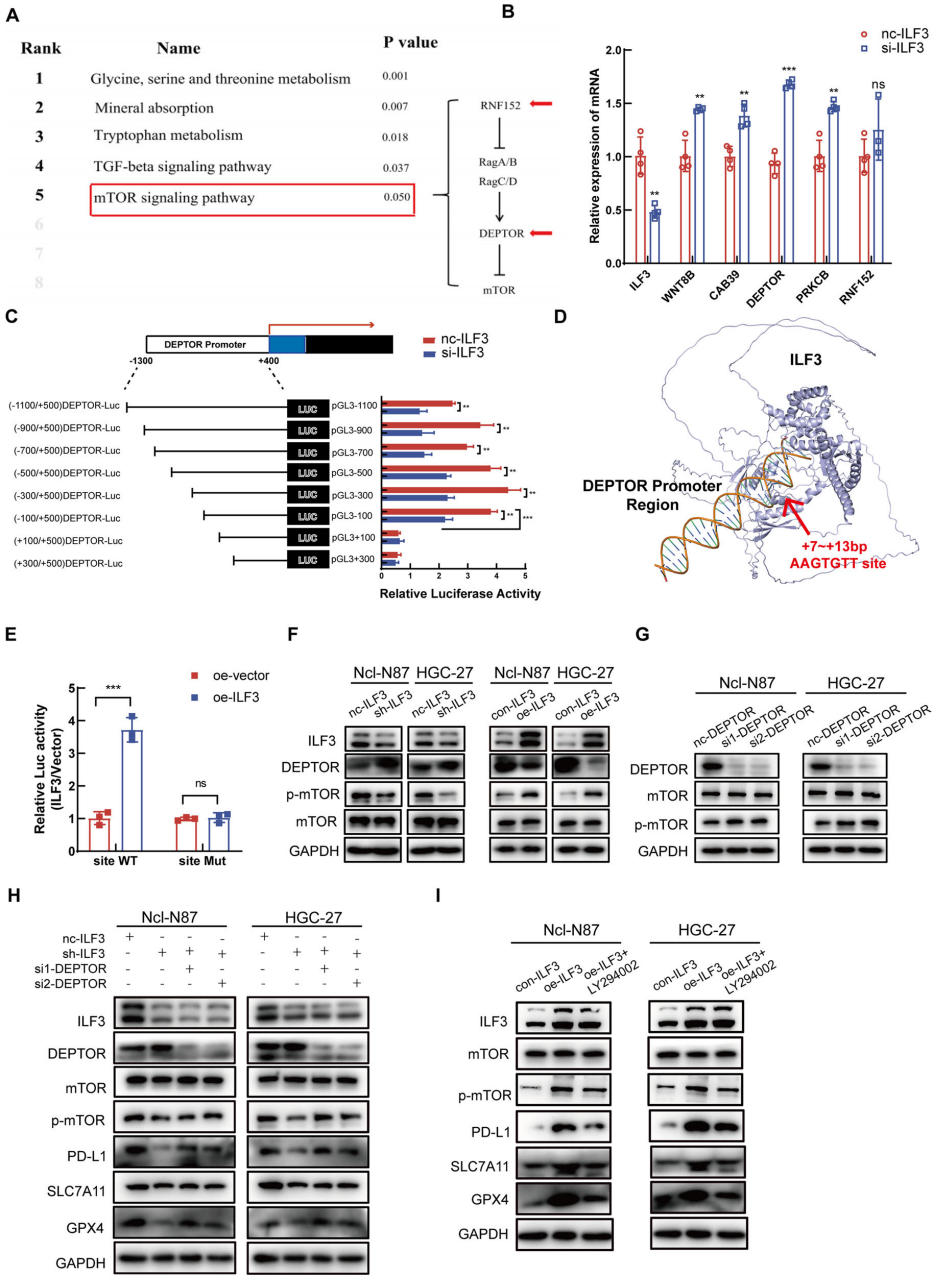
ILF3 regulated PD-L1 expression and ferroptosis through the DEPTOR/mTOR signaling pathway. **A** KEGG enrichment analysis after NGS. **B** mRNA levels of WNT8B, CAB39, DEPTOR, PRKCB, and RNF152 in the nc-ILF3 and si-ILF3 groups of Ncl-N87 cells. **C** A relative luciferase activity assay was conducted on HEK293T cells following transfection with pGL3 promoter constructs that contained DNA fragments serially deleted from the −1300 to +500 bp region of the DEPTOR promoter. **D** AlphaFold3 software was used to analyze the spatial structure of the binding domains between the DEPTOR promoter and ILF3. **E** Assessment of the effect of ILF3 on the transcription level of DEPTOR by a dual-luciferase reporter assay. **F** Protein expression levels of ILF3, DEPTOR, mTOR, and p-mTOR in nc-ILF3, sh-ILF3, con-ILF3, and oe-ILF3 cells. **G** Protein expression levels of DEPTOR, mTOR, and p-mTOR in the nc-DEPTOR, si-DEPTOR1, and si-DEPTOR2 groups. **H** The protein expression levels of ILF3, DEPTOR, mTOR, p-mTOR, PD-L1, SLC7A11, and GPX4 were determined by WB in the nc-ILF3, sh-ILF3, sh-ILF3+si1-DEPTOR, and sh-ILF3+si2-DEPTOR groups. **I** ILF3, mTOR, p-mTOR, PD-L1, SLC7A11, and GPX4 expression levels in the con-ILF3, oe-ILF3 and oe-ILF3 + LY294002 groups were determined by WB.

Kyoto Encyclopedia of Genes (KEGG) enrichment analysis was performed on the next-generation sequencing (NGS) results of nc-ILF3 and si-ILF3, which revealed that ILF3 regulated the mTOR signaling pathway (Fig. 6A). The differential genes enriched in the mTOR signaling pathway were WNT8B, CAB39, DEPTOR, PRKCB, and RNF152. The qRT‒PCR results revealed that DEPTOR expression was the most significantly different between the nc-ILF3 and si-ILF3 groups (Fig. 6B). Previous research has demonstrated that ILF3 functions as a crucial transcription factor in regulating the transcription and expression of downstream molecules [38, 39]. Consequently, we hypothesized that ILF3 may modulate the expression of DEPTOR by regulating its transcription. To test this hypothesis, we engineered pGL3-DEPTOR-Luc reporter plasmids featuring sequential deletions of the 5′-flanking regions, spanning from the −1300 bp region to the +500 bp region. The results from the dual luciferase assay indicated a reduction in luciferase activity in the si-ILF3 group relative to the nc-ILF3 group, implying that ILF3 interacts with the DEPTOR promoter region. Furthermore, luciferase activity was significantly reduced within the −100 to +100 bp region upstream of the DEPTOR promoter, indicating the presence of an ILF3 binding site in this region (Fig. 6C). To elucidate the binding sequences of the DEPTOR promoters with ILF3, AlphaFold3 software was used to analyze the spatial structure of the binding domains between the DEPTOR promoter and ILF3. These findings suggested that ILF3 may interact with the AAGTGTT site located within the +7 to +13 bp fragment of the DEPTOR promoter (Fig. 6D). Dual-luciferase reporter assays demonstrated that the overexpression of ILF3 significantly enhanced DEPTOR transcriptional activity. Conversely, the overexpression of ILF3 with a mutated binding site did not alter the transcriptional activity of DEPTOR (Fig. 6E). When ILF3 was knocked down, the protein level of DEPTOR increased, and the protein level of p-mTOR decreased; these effects were reversed after the overexpression of ILF3 (Fig. 6F). Previous studies demonstrated that DEPTOR is a suppressor of mTOR, which was further validated in GC cells [40]. WB revealed an increase in p-mTOR expression when DEPTOR was knocked down (Fig. 6G). When DEPTOR was rescued in sh-ILF3 cells, the WB results revealed that the protein levels of p-mTOR, PD-L1, SLC7A11, and GPX4 were also increased compared to those in the sh-ILF3 group (Fig. 6H). WB revealed that the protein expression levels of ILF3, p-mTOR, PD-L1, SLC7A11, and GPX4 were lower in the oe-ILF3 group treated with LY294002, which is an inhibitor of the mTOR signaling pathway, than in the oe-ILF3 group (Fig. 6I).

### Simvastatin induced ferroptosis in GC cells in nude mice by inhibiting ILF3 expression

**Fig. 7:**
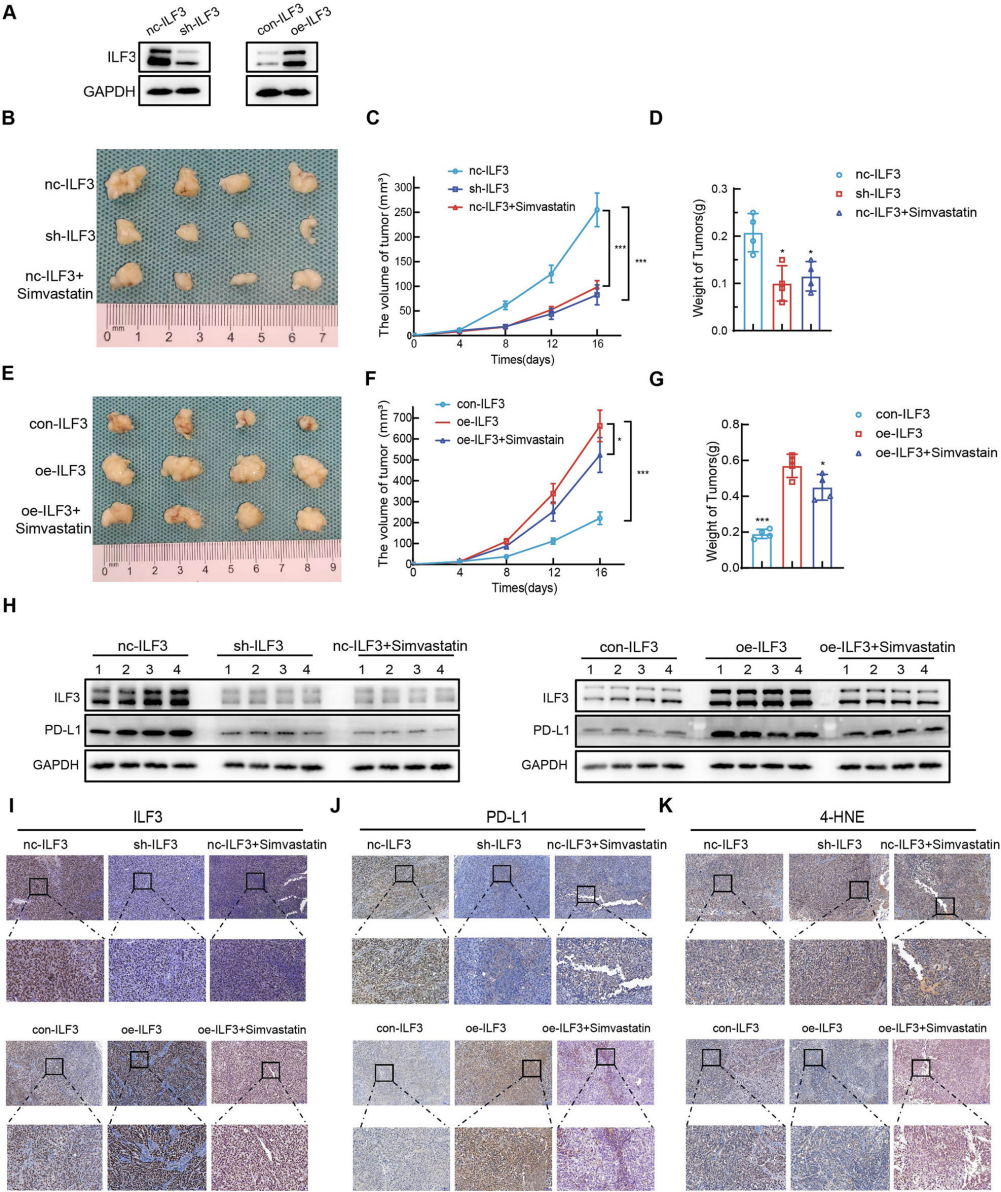
Simvastatin induced ferroptosis in GC cells in nude mice by inhibiting ILF3 expression. **A** Lentiviruses were used to transfect HGC-27 cells to stably express ILF3. Photographs of subcutaneous tumors (**B**), growth curves (**C**), and histograms of tumor weight (**D**) in the nc-ILF3, sh-ILF3, and nc-ILF3+simvastatin groups. Photographs of subcutaneous tumors (**E**), growth curves (**F**), and histograms of tumor weight (**G**) in the con-ILF3, oe-ILF3, and oe-ILF3+simvastatin groups. **H** WB was used to determine the protein levels of ILF3 and PD-L1 in subcutaneous tumors from the nc-ILF3, sh-ILF3, nc-ILF3+simvastatin, con-ILF3, oe-ILF3 and oe-ILF3+simvastatin groups. **I–K** IHC staining was used to determine the expression levels of ILF3, PD-L1, and 4-HNE in subcutaneous tumors from the nc-ILF3, sh-ILF3, nc-ILF3+simvastatin, con-ILF3, oe-ILF3 and oe-ILF3+simvastatin groups.

GC cells stably expressing ILF3 were generated by lentiviral vector transfection (Fig. 7A). Nude mice were randomly assigned to 6 distinct groups: the nc-ILF3, sh-ILF3, nc-ILF3+simvastatin, con-ILF3, oe-ILF3, and oe-ILF3+simvastatin groups. The results revealed that the suppression of ILF3 and the administration of simvastatin effectively impeded the proliferation of subcutaneous tumors and the weight of the subcutaneous tumors (Fig. 7B–D). Moreover, overexpression of ILF3 increased the proliferation and weight of subcutaneous tumors, and simvastatin inhibited the proliferation of subcutaneous tumors (Fig. 7E–G). ILF3 knockdown and simvastatin stimulation decreased the protein levels of both ILF3 and PD-L1. The overexpression of ILF3 with concomitant simvastatin stimulation reduced the protein expression levels of ILF3 and PD-L1 to some extent (Fig. 7H). IHC staining analysis revealed that sh-ILF3 and simvastatin treatment were accompanied by decreased ILF3 and PD-L1 expression and elevated 4-HNE expression levels. Simvastatin cotreatment with oe-ILF3 resulted in a reduction in ILF3 and PD-L1 expression to a certain degree, whereas 4-HNE expression was increased in comparison to the oe-ILF3 group (Fig. 7I–K).

### Simvastatin synergized with anti-PD-L1 therapy to achieve therapeutic effects in 615 mice

**Fig. 8:**
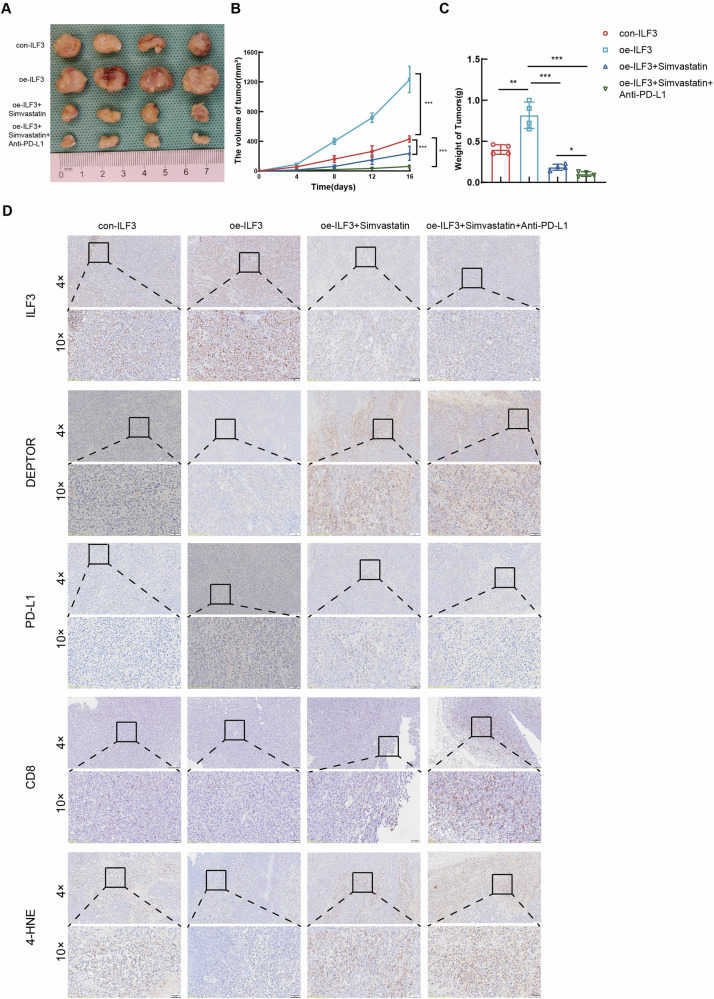
Simvastatin synergized with anti-PD-L1 therapy to achieve therapeutic effects in 615 mice. **A** Photographs of the subcutaneous tumors in each group. Growth curves (**B**) and histogram of subcutaneous tumor weight (**C**) in each group. **D** ILF3, DEPTOR, PD-L1, CD8, and 4-HNE expression in subcutaneous tumors was determined in each group by IHC staining.

MFC cells stably expressing ILF3 were generated by lentiviral vector transfection. The 615 mice were randomly assigned to 4 distinct groups: con-ILF3, oe-ILF3, oe-ILF3+simvastatin, and oe-ILF3+simvastatin+anti-PD-L1. The results revealed that high expression of ILF3 promoted the growth of tumors. Compared with treatment with simvastatin alone, coadministration of simvastatin and anti-PD-L1 resulted in a notable decrease in tumor burden (Fig. 8A–C). IHC staining revealed that simvastatin had a synergistic effect with anti-PD-L1 therapy and that ILF3 suppressed PD-L1 via the DEPTOR/mTOR signaling pathway, facilitated CD8^+^ T-cell infiltration, upregulated 4-HNE expression, and triggered ferroptosis (Fig. 8D).

## Discussion

Despite the development of a comprehensive treatment model incorporating surgery, chemotherapy, and immunotherapy, the prognosis of GC patients remains unfavorable [41]. Immunotherapy has emerged as a promising therapeutic approach for GC treatment, as it effectively augments the immune response against tumors [42]. Immune checkpoint inhibitors, especially PD-L1/PD-1 inhibitors, are pivotal in immunotherapy [43]. Hence, exploring novel mechanisms regulating PD-L1 expression and identifying pharmaceutical agents that modulate PD-L1 expression holds immense importance for the clinical management of GC and enhances prognostic outcomes among GC patients. This study revealed that simvastatin enhanced the sensitivity of GC patients to immunotherapy, which led to further investigation of the role of simvastatin in promoting cytotoxic T-cell-mediated killing through ILF3-regulated PD-L1.

Various factors, both in vivo and in vitro, influence the efficacy of immunotherapy in treating tumors. The immune cells and metabolic status of the tumor microenvironment significantly impact tumor immunogenicity [44]. Metabolic reprogramming is one of the characteristics of cancer cells [45, 46]. The rapid proliferation of tumor cells requires substantial quantities of lipids to synthesize cell membranes [47, 48]. Thus, the inhibition of lipid metabolism inhibits tumor cell growth and enhances sensitivity to immunotherapy [49]. It has been reported that aberrant expression of the lipid metabolism differential gene CYP19A1 in CRC is not only a prognostic risk factor for CRC patients but also enhances the ability of activated CD8^+^ T cells to kill CRC cells by suppressing the expression of PD-L1 in CRC cells [50]. Research has demonstrated that CD8^+^ tumor-infiltrating lymphocytes internalize ox-LDL through the CD36 receptor, triggering lipid peroxidation and subsequently impairing the functionality of CD8^+^ T cells [51]. The impact of lipid metabolism on PD-L1 expression and CD8^+^ T-cell function may have significant implications for the effectiveness of immunotherapy, thereby opening novel avenues for further exploration in the field.

Statins are inhibitors of the MVA pathway, inhibit cholesterol synthesis [52], and are the most common lipid-lowering drugs used in clinical practice [53]. Previous research has shown that statins can exert their antitumor effects through two different mechanisms: metabolism-dependent and noncholesterol-dependent [54]. Cholesterol is essential for maintaining intracellular homeostasis [55], and elevated cholesterol levels within tumor cells play a crucial role in enabling evasion of immune surveillance and fostering the upregulation of inhibitory immune checkpoint genes, consequently impeding the efficacy of antitumor responses [56]. The intermediate metabolites of the MVA pathway, specifically isopentenyl pyrophosphate (IPP), farnesyl pyrophosphate (FPP), and geranylgeranyl pyrophosphate (GGPP), can prenylate GTPases, thereby facilitating tumor progression [57]. Several studies have elucidated the cancer-inhibitory effects of statins through metabolism-dependent pathways. For example, statins suppress the Hedgehog signaling pathway by inhibiting cholesterol synthesis in combination with vismodegib to treat medulloblastoma [58]. Statins decrease the levels of the MVA pathway intermediates IPP and FPP, leading to the inhibition of Ras and Rho family GTPases and ultimately contributing to their anticancer properties [59]. Consequently, the present study aimed to investigate the mechanism of action of simvastatin in treating gastric cancer, with a specific focus on the nonmetabolism-dependent pathway. For example, lovastatin enhances sensitivity to cisplatin by increasing autophagy in tumor cells [60]. Simvastatin induces apoptosis in tumor cells by upregulating Bax and downregulating Bcl-2 expression [61]. In recent years, a growing body of research has investigated the potential role of statins in immunotherapy for malignant tumors. Simvastatin inhibits the activation of YAP by the lncRNA SNHG29 and enhances the antitumor immune response by suppressing PD-L1 expression in CRC. In addition, statins improve breast cancer treatment by blocking PD-1/PD-L1 [62]. Therefore, it is plausible that statins could enhance the therapeutic outcomes of immunotherapy and improve the prognosis of patients with tumors.

ILF3 is a double-stranded RNA-binding protein that plays a role in the modulation of gene expression and maintains mRNA stability [63]. Furthermore, ILF3 plays a crucial role in the pathogenesis and progression of diverse malignant neoplasms. Recent research revealed a positive correlation between the expression levels of ILF3 and tumor mutational burden and microsatellite instability in diverse malignant tumors [19]. Nevertheless, the relationship between ILF3 expression levels in tumors and the infiltration of immune cells remains inadequately explored. Elevated ILF3 expression and increased immune cell infiltration in the tumor area have been reported in HCC studies [19]. Notably, the suppression of ILF3 diminishes the expression of PD-L1 and augments the susceptibility of HCC cells to T-cell cytotoxicity [64]. However, few studies on the correlation between ILF3 and PD-L1 in GC exist. This study revealed a positive correlation between the expression levels of ILF3 and PD-L1 in GC cells and tissues. Following coculture with activated CD8^+^ T cells, the mortality rate was elevated in si-ILF3 GC cells and reduced in oe-ILF3 GC cells. Consequently, the suppression of ILF3 resulted in decreased PD-L1, enhancing the cytotoxicity of activated CD8^+^ T cells toward GC cells, which triggered an immune response and played a therapeutic role in GC. Further exploration revealed that simvastatin effectively suppressed the expression of PD-L1 through ILF3, consequently augmenting the cytotoxicity of CD8^+^ T cells to GC cells. The aforementioned empirical findings elucidated a novel mechanism underlying the therapeutic efficacy of simvastatin in GC and established a theoretical basis for the combined utilization of statins in GC immunotherapy.

Previous studies have shown that MIR155HG binding to ILF3 promotes PD-L1 expression after enhancing the stability of HIF-1α, which leads to the immune escape of HCC cells [65]. In head and neck squamous cell carcinoma, PD-L1 binds to ILF3 and IL2 to activate STAT3 for PD-L1 signaling to promote tumor progression [66]. Thus, there may be multiple mechanisms by which ILF3 regulates PD-L1 expression. The KEGG analysis results in this study suggested that ILF3 regulated the TGF-β and mTOR pathways. Recent studies utilizing simultaneous KEGG and gene set enrichment analyses have demonstrated that ILF3 plays a crucial role in positively regulating the mTOR pathway [20]. The results in this study revealed that ILF3 was a key transcription factor that regulated DEPTOR, an important repressor in the mTOR pathway, highlighting the importance of the role of ILF3 in regulating mTOR signaling. The findings of the present study confirmed the results of previous studies. The mTOR pathway plays a vital role in normal physiological processes but is also dysregulated in various malignant tumors and promotes tumor progression through various mechanisms [67, 68]. On the one hand, there is a close relationship between the mTOR pathway and ferroptosis. The mTOR pathway was found to inhibit ferroptosis in tumor cells through the activation of SREBP1/SCD1 [69]. In rheumatoid arthritis, the mTOR pathway inhibits synovial fibroblast ferroptosis by increasing GPX4 expression [70]. On the other hand, the mTOR pathway is crucial for enabling tumor cells to evade the immune system. In GC cells, TRIM28 significantly increases PD-L1 expression by activating the TBK1-mTOR pathway, suppressing T-cell activation, and promoting tumor progression [28]. In this study, these results revealed that ILF3 increased the expression of SLC7A11/GPX4 through the mTOR pathway and inhibited ferroptosis in GC cells. Moreover, PD-L1 promoted the expression of PD-L1 and inhibited the killing effect of activated CD8^+^ T cells on GC cells, which was consistent with the current findings on mTOR. However, whether ILF3 regulates both ferroptosis and the expression of PD-L1 in GC cells through the TGF-β pathway still needs to be investigated in further experimental studies.

Ferroptosis is a newly discovered form of regulated cell death (RCD) characterized by iron-dependent excess LPO and ROS production [71, 72]. Despite the significant contribution of immunotherapy in the management of malignant tumors, a considerable proportion of patients continue to experience unsatisfactory treatment outcomes. Recent investigations have increasingly highlighted the pivotal involvement of ferroptosis in the modulation of immunotherapy [73]. Inducing the onset of ferroptosis in cancer cells may improve the efficacy of immunotherapy. CD8^+^ T cells are significant contributors to ferroptosis induction in tumor cells. Previous studies have suggested that the clearance of tumor cells is accomplished by activated CD8^+^ T cells through the utilization of the perforin-granzyme- and Fas-L/Fas ligand pathways [74]. In addition, IFN-γ is a key effector molecule released by activated CD8^+^ T cells that functions by binding to IFN-γ receptor receptors on cancer cells [75]. Arachidonic acid and IFN-γ facilitate the upregulation of ACSL4 expression by activating the STAT1 and IRF1 signaling pathways, ultimately leading to ferroptosis induction in tumor cells [76]. In addition, IFN-γ was found to trigger ferroptosis in hepatocellular carcinoma by activating the STAT1/IRF1/ACSL4 axis [36]. Moreover, the SLC7A11/GPX4 pathway plays a crucial role in mitigating the accumulation of lipid peroxidation products and inhibiting the process of ferroptosis [77, 78]. For example, IFN-γ upregulated STAT3 phosphorylation and suppressed SLC7A11 transcription, leading to decreased cystine uptake and the initiation of ferroptosis [36]. This finding implies the significant involvement of IFN-γ in the induction of ferroptosis in tumor cells by activated CD8^+^ T cells. Research has demonstrated that increased expression of PD-L1 in tumor cells leads to immunosuppression within the tumor microenvironment, serving as a critical mechanism for tumor evasion by the immune system [79]. Therefore, immune checkpoint blockade is a critical method for enhancing the efficacy of immunotherapy. This study demonstrated that simvastatin inhibited ILF3 expression, which subsequently suppressed SLC7A11/GPX4 expression via the DEPTOR/mTOR signaling pathway, leading to ferroptosis in GC cells. Additionally, simvastatin downregulated PD-L1 expression in GC cells, enhancing the cytotoxicity of activated CD8^+^ T cells against GC cells. These findings elucidate a novel mechanism through which simvastatin induces ferroptosis in GC cells and improves the effectiveness of immunotherapy.

## Conclusion

In summary, simvastatin reduced the acetylation of ILF3 at the H3K14 site by increasing HDAC6 expression, which subsequently led to a decrease in ILF3 expression. The downregulation of ILF3 resulted in decreased expression of SLC7A11/GPX4 via the DEPTOR/mTOR signaling pathway, which induced ferroptosis. Moreover, ILF3-mediated inhibition of PD-L1 expression facilitated the recruitment of CD8^+^ T cells and augmented their cytotoxicity against gastric cancer cells.

## Supplementary information

Supplemental Materials

Original western blot

## Data Availability

The datasets used and analyzed in this study are available from the corresponding author.
