# Supplementary material for: Simvastatin inhibits PD-L1 via ILF3 to induce ferroptosis in gastric cancer cells
Source: Cell Death Dis. 2025 Mar 26;16(1):208. doi: 10.1038/s41419-025-07562-8 (PMC11947124; doi:10.1038/s41419-025-07562-8)
Supplement: Supplementary file 1 — Supplemental Materials [file 41419_2025_7562_MOESM1_ESM.docx]

**Supplemental Figure 1**


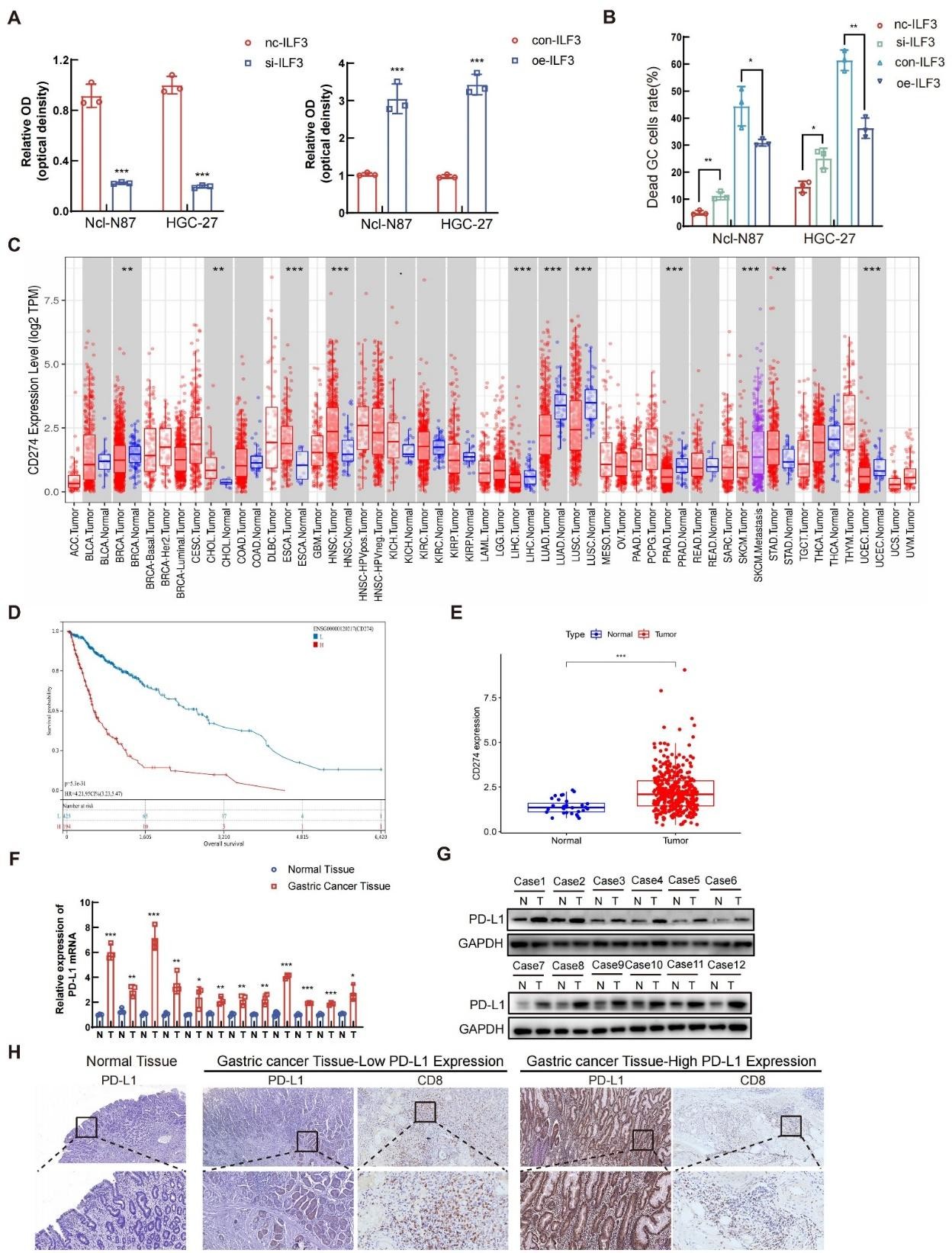


**Figure S1 PD-L1 expression levels correlate with the prognosis of GC patients.**

**(A)** Relative absorbance values at OD590 after crystal violet staining following knockdown and overexpression of ILF3. **(B)** Flow cytometry analysis of the rate of dead GC cells co-cultured with activated CD8^+^ T cells following knockdown and overexpression of ILF3. **(C)** Pan-cancer

analysis of PD-L1 expression levels in multiple normal and tumor groups.

**(D)** Relationship between PD-L1 expression levels and OS in patients with malignant tumors. **(E)** PD-L1 mRNA levels between GC and normal gastric tissues from TCGA database. **(F)** qRT-PCR analysis of PD-L1 expression in GC and normal gastric tissues. **(G)** WB analysis of PD-L1 expression in GC and normal gastric tissues. **(H)** IHC staining analysis PD-L1 and CD8 expression levels in GC and normal tissues.

**Supplemental Figure 2**

**
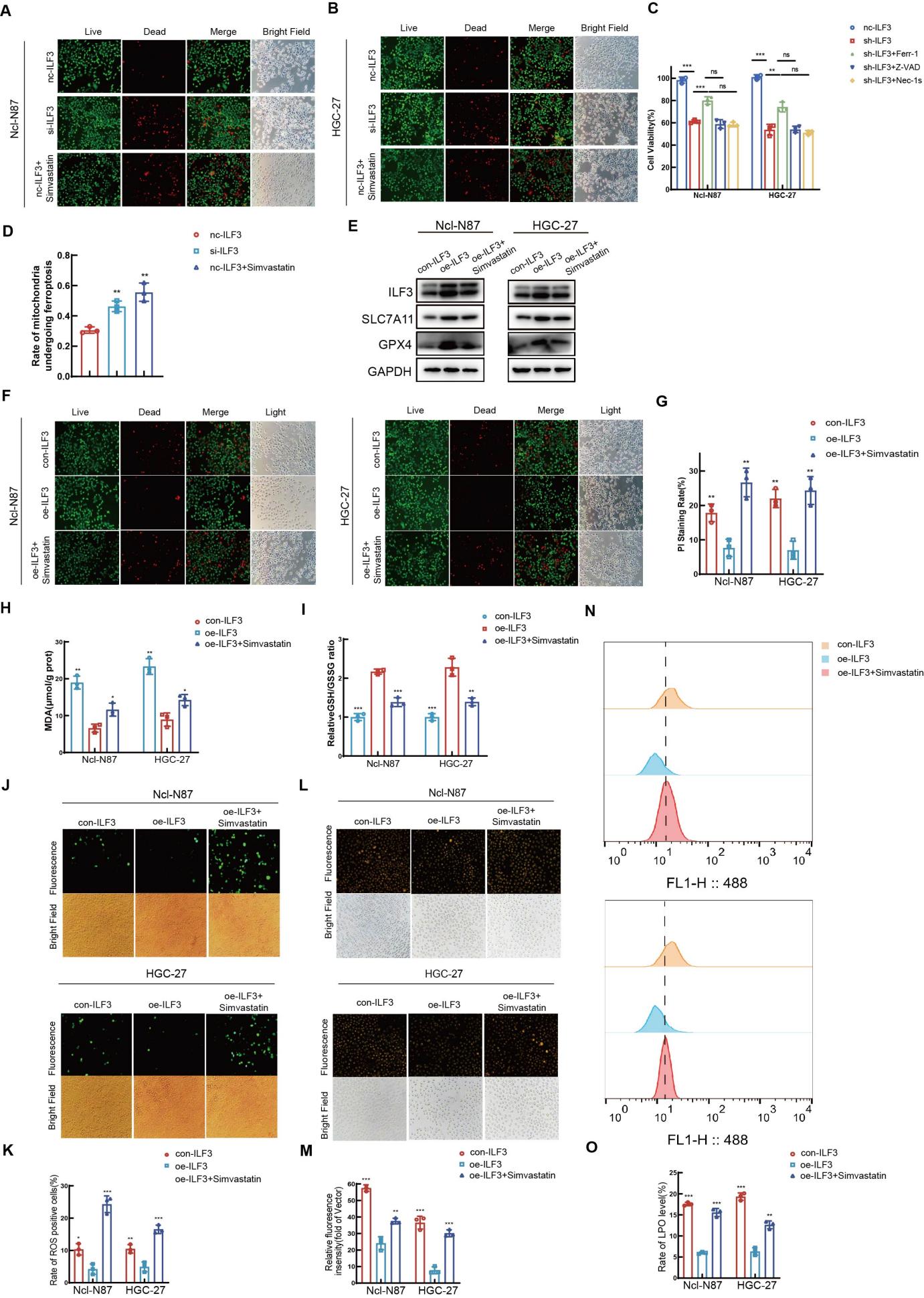
**

**Figure S2 Simvastatin partially counteracted the suppression of ferroptosis induced by the overexpression of ILF3.**

**(A, B)** Calcein/PI staining to detect the activity of GC cells following knockdown ILF3 and simvastatin stimulation by fluorescence microscopy.

**(C)** MTT assay analysis cell viability of GC cells knocked down ILF3 treated with Ferr-1, Z-VAD, and Nec-1s. **(D)** Histogram of the rate of mitochondria undergoing ferroptosis following knockdown ILF3 and simvastatin stimulation. **(E)** WB analysis of ILF3, SLC7A11, and GPX4 following overexpression of ILF3 and simvastatin stimulation. **(F, G)** Calcein/PI staining to detect the activity of GC cells following overexpression of ILF3 and simvastatin stimulation by fluorescence microscopy. Relative ferroptosis levels determined by MDA **(H)**, GSH **(I),** ROS **(J, K)**, Fe2+ **(L, M)**, and LPO **(N, O)** in GC cells following overexpression of ILF3 and simvastatin stimulation.

**Supplemental Figure 3**

**
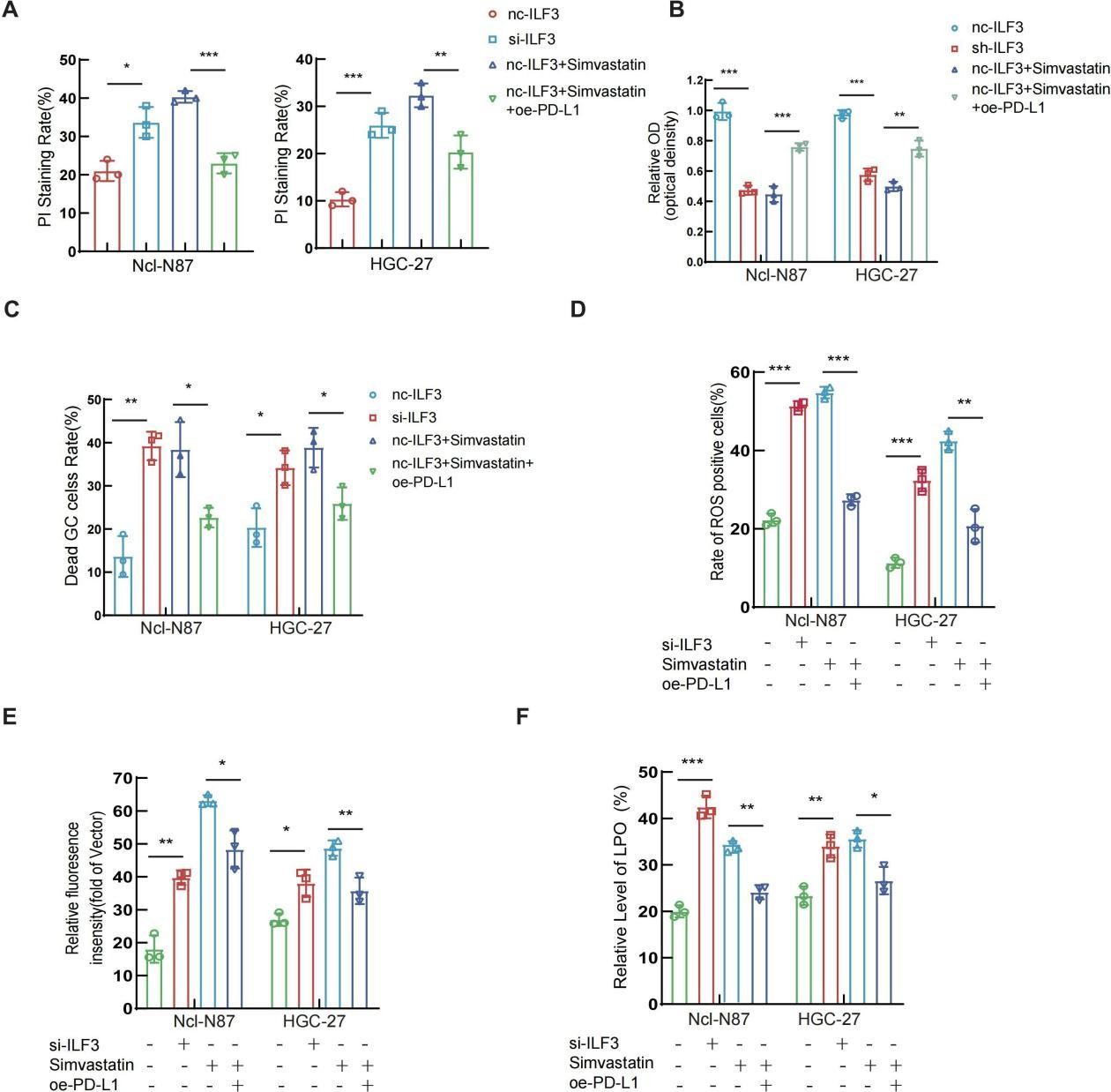
**

**Figure S3 Overexpression of PD-L1 can partially rescue the effect of simvastatin-inducing ferroptosis in GC cells.**

**(A)** Histogram of the activity of GC cells in Calcein/PI staining assay. **(B)** Histogram of the relative absorbance values at OD590 after crystal violet staining. **(C)** Histogram of the rate of dead GC cells in flow cytometry assay. Histogram of relative ferroptosis levels determined by ROS **(D)**, Fe2+ **(E)**, and LPO **(F)** in GC cells.

**Table S1 The sequence of siRNA Name Sequences**

si-nc 5’-UUC UCC GAA CGU GUC ACG UTT-3.' si-ILF3 5’-ACG UGA CAC GUU CGG AGA ATT-3' si-DEPTOR-1 5'-GCC ATG ACA ATC GGA AAT CTA-3.' si-DEPTOR-2 5'- CCT ACA TGA TAG AAC TGC CTT-3 si-HDAC1 5'-CAACUAUGGUCUCUACCGA-3'

si-HDAC2 5’-AAGCATCAGGATTCTGTTA-3ʹ

si-HDAC6 3'-GGACAACATGGAGAGGACAATGTA-5'

**Table S2 The primer sequences used for qRT-PCR experiments Name Sequences**

Homo-ILF3-F Homo-ILF3-R Homo-GAPDH-F Homo-GAPDH-R Homo-WNT8B-F Homo-WNT8B-R Homo-CAB39-F Homo-CAB39-R Homo-DEPTOR-F Homo-DEPTOR-R Homo-PRKCB -F Homo-PRKCB-R Homo-PD-L1-F Homo-PD-L1-R

CATTACGCCCATGAAACGCC TAAAGATGGGGGCATGGACG GCACCGTCAAGGCTGAGAAC TGGTGAAGACGCCAGTGGA AAGTACCACGCAGCACTCAA GTTCTCCAGGCAGTAGTCCG CTGCTGCGAGACAAAAGTCG GGGCTGCGTCTTGTTAGGAT GGTGCGAGGAAGTAAGCCAT CCCGTTGACAGAGACGACAA GTCAAGAACCACAAATTCACCGC ATCGGAGGCTGGACCCTTG GGTAAGACCACCACCACC TGATTCTCAGTGTGCTGGT

**Table S3 Tumor Regression Grade (TRG) Grading Criteria** Tumor Regression Grade (TRG) Microscopic View TRG0 No residual tumor cells

TRG1 A single or small group of cells

TRG2 Cancer with fibrotic response

TRG3 Minimal tumor response to treatment

**Table S4 Clinical data of gastric cancer patients treated with preoperative neoadjuvant chemotherapy, grouped by the treatment with and without statins.**

| Index | Statins History | No Statins History | P value |
| --- | --- | --- | --- |
|  | (n=50) | (n=50) |  |
| Age(years) | 59.32±10.26 | 61.06±7.71 | 0.34 |
| Sex |  |  |  |
| Male | 13 | 18 | 0.28 |
| Female | 37 | 32 |  |
| Chemotherapy |  |  |  |
| SOX | 24 | 22 |  |
| DOS | 7 | 8 | 0.816 |
| FLOT | 8 | 6 |  |
| XELOX | 6 | 11 |  |
| FOLFOX | 5 | 3 |  |
| Chemotherapy Cycles | | | |
| 2 | 22 | 13 |  |
| 3 | 12 | 12 | 0.297 |
| 4 | 8 | 15 |  |
| 5 | 4 | 4 |  |
| 6 | 4 | 6 |  |
| TRG score |  |  |  |
| 0 | 8 | 6 |  |
| 1 | 16 | 10 | 0.000 |
| 2 | 14 | 18 |  |
| 3 | 12 | 16 |  |

| **Table S5 Clinical data o** | **f gastric cancer p** | **atients and normal peop** | **le.** |
| --- | --- | --- | --- |
| Index | Normal People  (n=50) | Gastric cancer patients  (n=150) | P value |
| Age(years)  Sex | 62.6±7.20 | 62.9±9.42 | 0.165 |
| Male | 26 | 81 | 0.806 |
| Female  Comorbidity | 24 | 69 |  |
| Coronary heart disease | 5 | 4 | 0.868 |
| Hypertension | 8 | 9 |  |
| Diabetes mellitus | 3 | 4 |  |

**Table S6 Clinical data of gastric cancer patients treated with preoperative neoadjuvant chemotherapy combined with immunotherapy, grouped by the treatment with and without statins.**

| Index | Statins History | No Statins History | P value |
| --- | --- | --- | --- |
|  | (n=30) | (n=30) |  |
| Age(years) | 60.60±8.39 | 61.13±11.58 | 0.839 |
| Sex |  |  |  |
| Male | 12 | 14 | 0.602 |
| Female | 18 | 16 |  |
| Chemotherapy |  |  |  |
| Cycles | 12 | 5 |  |
| 2 | 3 | 8 | 0.199 |
| 3 | 3 | 5 |  |
| 4 | 6 | 5 |  |
| 5 | 6 | 7 |  |
| 6 |  |  |  |
| TRG score |  |  |  |
| 0 | 8 | 4 |  |
| 1 | 12 | 5 | 0.031 |
| 2 | 7 | 11 |  |
| 3 | 3 | 10 |  |

**Table S7 Clinical data of gastric cancer patients treated with preoperative neoadjuvant chemotherapy combined with immunotherapy, grouped by the high and low expression of ILF3.**

| Index | High ILF3 Expression | Low ILF3 Expression | P value |
| --- | --- | --- | --- |
|  | (n=30) | (n=30) |  |
| Age(years) | 60.60±8.39 | 58.57±13.15 | 0.478 |
| Sex |  |  |  |
| Male | 12 | 11 | 0.791 |
| Female | 18 | 19 |  |
| pTNM Stage |  |  |  |
| I | 11 | 10 | 0.70 |
| II | 7 | 5 |  |
| III | 12 | 15 |  |

TRG score

| 0 | 11 | 6 |  |
| --- | --- | --- | --- |
| 1 | 10 | 4 | 0.037 |
| 2 | 5 | 12 |  |
| 3 | 4 | 8 |  |

**Table S8 Clinical data of gastric cancer patients treated with preoperative neoadjuvant chemotherapy combined with immunotherapy, grouped by the PD- L1 expression level.**

| Index | High PD-L1 Expression | Low PD-L1 Expression | P value |
| --- | --- | --- | --- |
|  | (n=38) | (n=31) |  |
| Age(years) | 62.00±9.00 | 61.79±10.90 | 0.498 |
| Sex |  |  |  |
| Male | 18 | 12 | 0.47 |
| Female | 20 | 19 |  |
| pTNM Stage |  |  |  |
| I | 14 | 12 | 0.98 |
| II | 8 | 6 |  |
| III | 16 | 13 |  |

TRG score

| 0 | 15 | 7 |  |
| --- | --- | --- | --- |
| 1 | 10 | 4 | 0.047 |
| 2 | 9 | 11 |  |
| 3 | 4 | 10 |  |

**Table S9 The potential targets of ILF3 from the hTFtarget database.**

| TF_name | target_id | target_name |
| --- | --- | --- |
| ILF3 | ENSG0000027064 | TAF15 |
| ILF3 | 7  ENSG0000022461 | RP11-153F1.1 |
| ILF3 | 3  ENSG0000021903 | AC005102.1 |
| ILF3 | 9  ENSG0000027440 | RP11-294N21.3 |
| ILF3 | 0  ENSG0000004834 | CC2D2A |
|  | 2 |  |
| ILF3 | ENSG0000020438 | HSPA1B |
| ILF3 | 8  ENSG0000020407 | SYS1 |
| ILF3 | 0  ENSG0000011173 | C2CD5 |
| ILF3 | 1  ENSG0000011733 | CD46 |
| ILF3 | 5  ENSG0000022098 | SNORD88C |
| ILF3 | 8  ENSG0000018458 | TMEM173 |
| ILF3 | 4  ENSG0000019664 | RABL6 |
| ILF3 | 2  ENSG0000023158 | AC034228.2 |
| ILF3 | 5  ENSG0000016528 | PIGO |
| ILF3 | 2  ENSG0000021070 | RNU6ATAC3P |
|  | 9 |  |
| ILF3 | ENSG0000024931 | CTC-493P15.2 |
| ILF3 | 6  ENSG0000024927 | HNRNPA1P44 |
| ILF3 | 1  ENSG0000016678 | C16orf45 |
| ILF3 | 0  ENSG0000010525 | SHD |
| ILF3 | 1  ENSG0000008902 | MAPKAPK5 |
| ILF3 | 2  ENSG0000020247 | RNA5SP248 |
| ILF3 | 2  ENSG0000016904 | HNRNPH1 |
| ILF3 | 5  ENSG0000015653 | PHF6 |
| ILF3 | 1  ENSG0000012383 | PFKFB2 |
| ILF3 | 6  ENSG0000016781 | PRDX2 |
|  | 5 |  |
| ILF3 | ENSG0000008411 | SSH1 |
| ILF3 | 2  ENSG0000023417 | AC016683.5 |
| ILF3 | 4  ENSG0000012053 | ENY2 |

|  | 3 |  |
| --- | --- | --- |
| ILF3 | ENSG0000013258 | FLOT2 |
| ILF3 | 9  ENSG0000026039 | CTD-2649C14.1 |
| ILF3 | 5  ENSG0000018279 | CCDC87 |
| ILF3 | 1  ENSG0000020073 | RNU6-194P |
| ILF3 | 2  ENSG0000012587 | TBC1D20 |
| ILF3 | 5  ENSG0000011101 | CYP27B1 |
| ILF3 | 2  ENSG0000016300 | CFAP36 |
|  | 1 |  |
| ILF3 | ENSG0000024178 | RN7SL390P |
| ILF3 | 5  ENSG0000021417 | AMZ2P1 |
| ILF3 | 4  ENSG0000026375 | XXbac- |
| ILF3 | 6  ENSG0000017073 | BPG181M17.6 POLH |
| ILF3 | 4  ENSG0000016767 | UBXN6 |
| ILF3 | 1  ENSG0000014578 | FEM1C |
| ILF3 | 0  ENSG0000022487 | AC083949.1 |
| ILF3 | 5  ENSG0000022631 | RP3-323P24.3 |
| ILF3 | 0  ENSG0000012859 | DNAJB9 |
| ILF3 | 0  ENSG0000024965 | RP11-310P5.1 |
|  | 0 |  |
| ILF3 | ENSG0000011560 | IL18R1 |
| ILF3 | 4  ENSG0000027898 | LA16c-431H6.7 |
| ILF3 | 7  ENSG0000010433 | IMPAD1 |
| ILF3 | 1  ENSG0000016973 | RFNG |
| ILF3 | 3  ENSG0000020667 | RNU6-945P |
|  | 4 |  |

| ILF3 | ENSG00000273299 | CTB-13L3.1 |
| --- | --- | --- |
| ILF3 | ENSG00000185813 | PCYT2 |
| ILF3 | ENSG00000104901 | DKKL1 |
| ILF3 | ENSG00000105048 | TNNT1 |
| ILF3 | ENSG00000228801 | RP11-110G21.1 |
| ILF3 | ENSG00000212335 | Y_RNA |
| ILF3 | ENSG00000148019 | CEP78 |
| ILF3 | ENSG00000272034 | SNORD14A |
| ILF3 | ENSG00000269404 | SPIB |
| ILF3 | ENSG00000157349 | DDX19B |
| ILF3 | ENSG00000115966 | ATF2 |
| ILF3 | ENSG00000175766 | EIF4E1B |
| ILF3 | ENSG00000101966 | XIAP |
| ILF3 | ENSG00000198324 | FAM109A |
| ILF3 | ENSG00000174483 | BBS1 |
| ILF3 | ENSG00000198429 | ZNF69 |
| ILF3 | ENSG00000122566 | HNRNPA2B1 |
| ILF3 | ENSG00000234371 | RPSAP31 |
| ILF3 | ENSG00000204856 | FAM216A |
| ILF3 | ENSG00000077348 | EXOSC5 |
| ILF3 | ENSG00000206597 | SNORA57 |
| ILF3 | ENSG00000225422 | RBMS1P1 |
| ILF3 | ENSG00000252545 | RNU6-362P |
| ILF3 | ENSG00000005981 | ASB4 |
| ILF3 | ENSG00000109736 | MFSD10 |
| ILF3 | ENSG00000268975 | MIA-RAB4B |
| ILF3 | ENSG00000213035 | RPL23AP80 |
| ILF3 | ENSG00000197256 | KANK2 |
| ILF3 | ENSG00000160349 | LCN1 |
| ILF3 | ENSG00000171858 | RPS21 |
| ILF3 | ENSG00000115694 | STK25 |
| ILF3 | ENSG00000277599 | NEAT1_3 |
| ILF3 | ENSG00000120948 | TARDBP |
| ILF3 | ENSG00000268742 | CTD-2396E7.7 |
| ILF3 | ENSG00000270806 | C17orf50 |
| ILF3 | ENSG00000249249 | AC010226.4 |
| ILF3 | ENSG00000156642 | NPTN |
| ILF3 | ENSG00000232956 | SNHG15 |
| ILF3 | ENSG00000135930 | EIF4E2 |
| ILF3 | ENSG00000232600 | AC084125.4 |
| ILF3 | ENSG00000261512 | RP11-46D6.1 |
| ILF3 | ENSG00000253355 | KB-1460A1.2 |
| ILF3 | ENSG00000100796 | SMEK1 |
| ILF3 | ENSG00000231530 | RP11-187A9.3 |
| ILF3 | ENSG00000222043 | AC079305.10 |
| ILF3 | ENSG00000243364 | EFNA4 |
| ILF3 | ENSG00000196547 | MAN2A2 |
| ILF3 | ENSG00000255621 | RP11-377D9.3 |
| ILF3 | ENSG00000186591 | UBE2H |
| ILF3 | ENSG00000198900 | TOP1 |
| ILF3 | ENSG00000182749 | PAQR7 |

| ILF3 | ENSG00000189114 | BLOC1S3 |
| --- | --- | --- |
| ILF3 | ENSG00000169607 | CKAP2L |
| ILF3 | ENSG00000130881 | LRP3 |
| ILF3 | ENSG00000196792 | STRN3 |
| ILF3 | ENSG00000275437 | RP5-908M14.10 |
| ILF3 | ENSG00000225077 | LINC00337 |
| ILF3 | ENSG00000226209 | RP11-24J20.5 |
| ILF3 | ENSG00000137714 | FDX1 |
| ILF3 | ENSG00000067182 | TNFRSF1A |
| ILF3 | ENSG00000162616 | DNAJB4 |
| ILF3 | ENSG00000128039 | SRD5A3 |
| ILF3 | ENSG00000268471 | MIR4453 |
| ILF3 | ENSG00000101624 | CEP76 |
| ILF3 | ENSG00000265753 | RN7SL444P |
| ILF3 | ENSG00000277264 | MIR6833 |
| ILF3 | ENSG00000253251 | CTC-534A2.2 |
| ILF3 | ENSG00000274390 | MIR6885 |
| ILF3 | ENSG00000237934 | RP11-467D18.2 |
| ILF3 | ENSG00000114125 | RNF7 |
| ILF3 | ENSG00000273885 | snoU2-30 |
| ILF3 | ENSG00000227492 | RP11-316M21.6 |
| ILF3 | ENSG00000232860 | SMG7-AS1 |
| ILF3 | ENSG00000260874 | RP11-715J22.4 |
| ILF3 | ENSG00000246877 | DNM1P35 |
| ILF3 | ENSG00000135547 | HEY2 |
| ILF3 | ENSG00000240889 | NDUFB2-AS1 |
| ILF3 | ENSG00000267986 | AC130469.2 |
| ILF3 | ENSG00000199075 | MIR26A1 |
| ILF3 | ENSG00000200087 | SNORA73B |
| ILF3 | ENSG00000279949 | RP11-10A14.9 |
| ILF3 | ENSG00000234882 | EIF3EP1 |
| ILF3 | ENSG00000106991 | ENG |
| ILF3 | ENSG00000261071 | RP1-223E5.4 |
| ILF3 | ENSG00000116254 | CHD5 |
| ILF3 | ENSG00000268129 | RP11-91G21.1 |
| ILF3 | ENSG00000205111 | CDKL4 |
| ILF3 | ENSG00000126653 | NSRP1 |
| ILF3 | ENSG00000269807 | AC007292.4 |
| ILF3 | ENSG00000236882 | LINC01554 |
| ILF3 | ENSG00000145949 | MYLK4 |
| ILF3 | ENSG00000122912 | SLC25A16 |
| ILF3 | ENSG00000254806 | SYS1-DBNDD2 |
| ILF3 | ENSG00000105369 | CD79A |
| ILF3 | ENSG00000267750 | RUNDC3A- |
| ILF3 | ENSG00000265943 | AS1  RP11-739L10.1 |
| ILF3 | ENSG00000049283 | EPN3 |
| ILF3 | ENSG00000275560 | RP11-180M15.7 |
| ILF3 | ENSG00000230749 | MEIS1-AS2 |
| ILF3 | ENSG00000236519 | LINC01424 |
| ILF3 | ENSG00000090539 | CHRD |
| ILF3 | ENSG00000166233 | ARIH1 |

| ILF3 | ENSG00000262660 | SLC25A10 |
| --- | --- | --- |
| ILF3 | ENSG00000084764 | MAPRE3 |
| ILF3 | ENSG00000143756 | FBXO28 |
| ILF3 | ENSG00000178772 | CPN2 |
| ILF3 | ENSG00000122642 | FKBP9 |
| ILF3 | ENSG00000238609 | RNU7-94P |
| ILF3 | ENSG00000236794 | BCRP8 |
| ILF3 | ENSG00000227432 | AC053503.11 |
| ILF3 | ENSG00000123395 | ATG101 |
| ILF3 | ENSG00000266714 | MYO15B |
| ILF3 | ENSG00000267776 | AC006116.24 |
| ILF3 | ENSG00000188321 | ZNF559 |
| ILF3 | ENSG00000266919 | MIR3184 |
| ILF3 | ENSG00000135535 | CD164 |
| ILF3 | ENSG00000142230 | SAE1 |
| ILF3 | ENSG00000232611 | RP11-1114A5.4 |
| ILF3 | ENSG00000158555 | GDPD5 |
| ILF3 | ENSG00000165046 | LETM2 |
| ILF3 | ENSG00000276259 | RP11-481J2.4 |
| ILF3 | ENSG00000047230 | CTPS2 |
| ILF3 | ENSG00000169299 | PGM2 |
| ILF3 | ENSG00000261505 | LA16c-358B7.3 |
| ILF3 | ENSG00000104848 | KCNA7 |
| ILF3 | ENSG00000155465 | SLC7A7 |
| ILF3 | ENSG00000113643 | RARS |
| ILF3 | ENSG00000182903 | ZNF721 |
| ILF3 | ENSG00000116032 | GRIN3B |
| ILF3 | ENSG00000115525 | ST3GAL5 |
| ILF3 | ENSG00000267361 | LOC100421166 |
| ILF3 | ENSG00000090097 | PCBP4 |
| ILF3 | ENSG00000117472 | TSPAN1 |
| ILF3 | ENSG00000137513 | NARS2 |
| ILF3 | ENSG00000172469 | MANEA |
| ILF3 | ENSG00000077312 | SNRPA |
| ILF3 | ENSG00000266124 | MIR5587 |
| ILF3 | ENSG00000270209 | RP4-592A1.4 |
| ILF3 | ENSG00000115257 | PCSK4 |
| ILF3 | ENSG00000154723 | ATP5J |
| ILF3 | ENSG00000188895 | MSL1 |
| ILF3 | ENSG00000104381 | GDAP1 |
| ILF3 | ENSG00000274904 | CTD- |
| ILF3 | ENSG00000166181 | 2515O10.5  API5 |
| ILF3 | ENSG00000162734 | PEA15 |
| ILF3 | ENSG00000213741 | RPS29 |
| ILF3 | ENSG00000130038 | CRACR2A |
| ILF3 | ENSG00000226944 | RP1-120G22.11 |
| ILF3 | ENSG00000140365 | COMMD4 |
| ILF3 | ENSG00000173011 | TADA2B |
| ILF3 | ENSG00000156831 | NSMCE2 |
| ILF3 | ENSG00000259431 | THTPA |

| ILF3 | ENSG00000106948 | AKNA |
| --- | --- | --- |
| ILF3 | ENSG00000167716 | WDR81 |
| ILF3 | ENSG00000255284 | AP006621.5 |
| ILF3 | ENSG00000179454 | KLHL28 |
| ILF3 | ENSG00000047249 | ATP6V1H |
| ILF3 | ENSG00000267592 | CTC-507E2.2 |
| ILF3 | ENSG00000258542 | AC068831.11 |
| ILF3 | ENSG00000170619 | COMMD5 |
| ILF3 | ENSG00000122644 | ARL4A |
| ILF3 | ENSG00000197937 | ZNF347 |
| ILF3 | ENSG00000230641 | USP12-AS2 |
| ILF3 | ENSG00000130812 | ANGPTL6 |
| ILF3 | ENSG00000240687 | RP11-521D12.1 |
| ILF3 | ENSG00000213849 | AC104306.1 |
| ILF3 | ENSG00000243978 | RGAG1 |
| ILF3 | ENSG00000237505 | PKN2-AS1 |
| ILF3 | ENSG00000262959 | LA16c-321D4.1 |
| ILF3 | ENSG00000278189 | RNA5-8S5 |
| ILF3 | ENSG00000187778 | MCRS1 |
| ILF3 | ENSG00000011332 | DPF1 |
| ILF3 | ENSG00000099949 | LZTR1 |
| ILF3 | ENSG00000170684 | ZNF296 |
| ILF3 | ENSG00000100577 | GSTZ1 |
| ILF3 | ENSG00000162144 | CYB561A3 |
| ILF3 | ENSG00000099290 | FAM21A |
| ILF3 | ENSG00000254441 | RP11-718B12.5 |
| ILF3 | ENSG00000258459 | CTD- |
| ILF3 | ENSG00000130811 | 2292M16.7 EIF3G |
| ILF3 | ENSG00000164032 | H2AFZ |
| ILF3 | ENSG00000180917 | CMTR2 |
| ILF3 | ENSG00000158163 | DZIP1L |
| ILF3 | ENSG00000155660 | PDIA4 |
| ILF3 | ENSG00000278619 | MRM1 |
| ILF3 | ENSG00000130312 | MRPL34 |
| ILF3 | ENSG00000197291 | RAMP2-AS1 |
| ILF3 | ENSG00000071243 | ING3 |
| ILF3 | ENSG00000004779 | NDUFAB1 |
| ILF3 | ENSG00000270876 | ZNF30-AS1 |
| ILF3 | ENSG00000177640 | CASC2 |
| ILF3 | ENSG00000159921 | GNE |
| ILF3 | ENSG00000272892 | RP11-57G10.8 |
| ILF3 | ENSG00000013288 | MAN2B2 |
| ILF3 | ENSG00000236425 | AC090018.1 |
| ILF3 | ENSG00000176619 | LMNB2 |
| ILF3 | ENSG00000141349 | G6PC3 |
| ILF3 | ENSG00000166747 | AP1G1 |
| ILF3 | ENSG00000119535 | CSF3R |
| ILF3 | ENSG00000137274 | BPHL |
| ILF3 | ENSG00000181201 | HIST3H2BA |
| ILF3 | ENSG00000201524 | RNU6-450P |
| ILF3 | ENSG00000245281 | CTD-2547L16.1 |

| ILF3 | ENSG00000070010 | UFD1L |
| --- | --- | --- |
| ILF3 | ENSG00000125743 | SNRPD2 |
| ILF3 | ENSG00000226137 | BAIAP2-AS1 |
| ILF3 | ENSG00000105136 | ZNF419 |
| ILF3 | ENSG00000207756 | MIR580 |
| ILF3 | ENSG00000114853 | ZBTB47 |
| ILF3 | ENSG00000273703 | HIST1H2BM |
| ILF3 | ENSG00000167380 | ZNF226 |
| ILF3 | ENSG00000134686 | PHC2 |
| ILF3 | ENSG00000145088 | EAF2 |
| ILF3 | ENSG00000237803 | LINC00211 |
| ILF3 | ENSG00000251660 | AC007036.5 |
| ILF3 | ENSG00000267322 | SNHG22 |
| ILF3 | ENSG00000211456 | SACM1L |
| ILF3 | ENSG00000198258 | UBL5 |
| ILF3 | ENSG00000099822 | HCN2 |
| ILF3 | ENSG00000137825 | ITPKA |
| ILF3 | ENSG00000102316 | MAGED2 |
| ILF3 | ENSG00000206698 | RNU1-73P |
| ILF3 | ENSG00000250379 | RP11-23P13.4 |
| ILF3 | ENSG00000145912 | NHP2 |
| ILF3 | ENSG00000237883 | DGUOK-AS1 |
| ILF3 | ENSG00000108578 | BLMH |
| ILF3 | ENSG00000166526 | ZNF3 |
| ILF3 | ENSG00000197780 | TAF13 |
| ILF3 | ENSG00000034971 | MYOC |
| ILF3 | ENSG00000070495 | JMJD6 |
| ILF3 | ENSG00000254021 | RP11-778D12.2 |
| ILF3 | ENSG00000261243 | RP11-517C16.4 |
| ILF3 | ENSG00000166797 | FAM96A |
| ILF3 | ENSG00000267565 | CTC-559E9.8 |
| ILF3 | ENSG00000186862 | PDZD7 |
| ILF3 | ENSG00000138594 | TMOD3 |
| ILF3 | ENSG00000222636 | RN7SKP54 |
| ILF3 | ENSG00000168062 | BATF2 |
| ILF3 | ENSG00000268836 | LA16c-OS12.2 |
| ILF3 | ENSG00000251537 | RP11-385D13.1 |
| ILF3 | ENSG00000106344 | RBM28 |
| ILF3 | ENSG00000129925 | TMEM8A |
| ILF3 | ENSG00000144182 | LIPT1 |
| ILF3 | ENSG00000099917 | MED15 |
| ILF3 | ENSG00000182473 | EXOC7 |
| ILF3 | ENSG00000132591 | ERAL1 |
| ILF3 | ENSG00000224333 | GAPDHP20 |
| ILF3 | ENSG00000134070 | IRAK2 |
| ILF3 | ENSG00000111785 | RIC8B |
| ILF3 | ENSG00000117899 | MESDC2 |
| ILF3 | ENSG00000116095 | PLEKHA3 |
| ILF3 | ENSG00000138760 | SCARB2 |
| ILF3 | ENSG00000070214 | SLC44A1 |
| ILF3 | ENSG00000156381 | ANKRD9 |

| ILF3 | ENSG00000253043 | RNU7-181P |
| --- | --- | --- |
| ILF3 | ENSG00000127528 | KLF2 |
| ILF3 | ENSG00000143294 | PRCC |
| ILF3 | ENSG00000224800 | RP11-235D19.2 |
| ILF3 | ENSG00000277916 | uc_338 |
| ILF3 | ENSG00000206417 | H1FX-AS1 |
| ILF3 | ENSG00000119686 | FLVCR2 |
| ILF3 | ENSG00000226659 | RP11-137H2.4 |
| ILF3 | ENSG00000128512 | DOCK4 |
| ILF3 | ENSG00000137074 | APTX |
| ILF3 | ENSG00000249700 | SRD5A3-AS1 |
| ILF3 | ENSG00000197245 | FAM110D |
| ILF3 | ENSG00000135999 | EPC2 |
| ILF3 | ENSG00000213471 | TTLL13 |
| ILF3 | ENSG00000110218 | PANX1 |
| ILF3 | ENSG00000108671 | PSMD11 |
| ILF3 | ENSG00000255072 | PIGY |
| ILF3 | ENSG00000272791 | RP11-464F9.22 |
| ILF3 | ENSG00000170385 | SLC30A1 |
| ILF3 | ENSG00000279599 | AC005514.2 |
| ILF3 | ENSG00000275043 | SNORD25 |
| ILF3 | ENSG00000272221 | XXbac- |
| ILF3 | ENSG00000241709 | BPG181B23.7  RN7SL265P |
| ILF3 | ENSG00000214402 | LCNL1 |
| ILF3 | ENSG00000104881 | PPP1R13L |
| ILF3 | ENSG00000204130 | RUFY2 |
| ILF3 | ENSG00000110328 | GALNT18 |
| ILF3 | ENSG00000127184 | COX7C |
| ILF3 | ENSG00000226797 | AC015923.1 |
| ILF3 | ENSG00000140264 | SERF2 |
| ILF3 | ENSG00000167785 | ZNF558 |
| ILF3 | ENSG00000219529 | AP000580.1 |
| ILF3 | ENSG00000144935 | TRPC1 |
| ILF3 | ENSG00000274135 | Metazoa_SRP |
| ILF3 | ENSG00000175854 | SWI5 |
| ILF3 | ENSG00000161544 | CYGB |
| ILF3 | ENSG00000240747 | KRBOX1 |
| ILF3 | ENSG00000176840 | MIR7-3HG |
| ILF3 | ENSG00000274979 | RP11-1143G9.5 |
| ILF3 | ENSG00000267561 | RP5-1052I5.2 |
| ILF3 | ENSG00000200877 | RNU6-560P |
| ILF3 | ENSG00000236320 | SLFN14 |
| ILF3 | ENSG00000071537 | SEL1L |
| ILF3 | ENSG00000127507 | EMR2 |
| ILF3 | ENSG00000279700 | RP13-554M15.2 |
| ILF3 | ENSG00000105793 | GTPBP10 |
| ILF3 | ENSG00000085377 | PREP |
| ILF3 | ENSG00000183091 | NEB |
| ILF3 | ENSG00000221102 | SNORA11B |
| ILF3 | ENSG00000137309 | HMGA1 |
| ILF3 | ENSG00000272688 | RP13-270P17.3 |

| ILF3 | ENSG00000265100 | RP11-147L13.2 |
| --- | --- | --- |
| ILF3 | ENSG00000131148 | EMC8 |
| ILF3 | ENSG00000272021 | AC008592.8 |
| ILF3 | ENSG00000268001 | CTC-241F20.3 |
| ILF3 | ENSG00000225142 | RP11-490K7.1 |
| ILF3 | ENSG00000105171 | POP4 |
| ILF3 | ENSG00000227698 | AP001619.2 |
| ILF3 | ENSG00000270060 | RP11-390K5.6 |
| ILF3 | ENSG00000185619 | PCGF3 |
| ILF3 | ENSG00000085511 | MAP3K4 |
| ILF3 | ENSG00000126746 | ZNF384 |
| ILF3 | ENSG00000146857 | STRA8 |
| ILF3 | ENSG00000227269 | RP11-96L7.2 |
| ILF3 | ENSG00000173275 | ZNF449 |
| ILF3 | ENSG00000115468 | EFHD1 |
| ILF3 | ENSG00000251580 | RP11-539L10.3 |
| ILF3 | ENSG00000143569 | UBAP2L |
| ILF3 | ENSG00000141552 | ANAPC11 |
| ILF3 | ENSG00000244115 | DNAJC25- |
| ILF3 | ENSG00000238622 | GNG10  SNORD97 |
| ILF3 | ENSG00000077063 | CTTNBP2 |
| ILF3 | ENSG00000117640 | MTFR1L |
| ILF3 | ENSG00000115464 | USP34 |
| ILF3 | ENSG00000121892 | PDS5A |
| ILF3 | ENSG00000113569 | NUP155 |
| ILF3 | ENSG00000226040 | AC005740.3 |
| ILF3 | ENSG00000166986 | MARS |
| ILF3 | ENSG00000221394 | MIR1229 |
| ILF3 | ENSG00000174748 | RPL15 |
| ILF3 | ENSG00000169599 | NFU1 |
| ILF3 | ENSG00000153201 | RANBP2 |
| ILF3 | ENSG00000259967 | RP11-2E17.2 |
| ILF3 | ENSG00000234390 | USP27X-AS1 |
| ILF3 | ENSG00000163900 | TMEM41A |
| ILF3 | ENSG00000171574 | ZNF584 |
| ILF3 | ENSG00000221990 | C5orf55 |
| ILF3 | ENSG00000224745 | RP11-380G5.2 |
| ILF3 | ENSG00000278264 | MIR6803 |
| ILF3 | ENSG00000166166 | TRMT61A |
| ILF3 | ENSG00000136997 | MYC |
| ILF3 | ENSG00000204682 | CASC10 |
| ILF3 | ENSG00000267040 | RP11-35G9.3 |
| ILF3 | ENSG00000261832 | CLN3 |
| ILF3 | ENSG00000177239 | MAN1B1 |
| ILF3 | ENSG00000232132 | NDFIP2-AS1 |
| ILF3 | ENSG00000109775 | UFSP2 |
| ILF3 | ENSG00000230216 | HSPB1P2 |
| ILF3 | ENSG00000156983 | BRPF1 |
| ILF3 | ENSG00000229666 | MAST4-AS1 |
| ILF3 | ENSG00000084070 | SMAP2 |
| ILF3 | ENSG00000102710 | SUPT20H |

| ILF3 | ENSG00000254779 | EGLN1P1 |
| --- | --- | --- |
| ILF3 | ENSG00000225265 | TAF1A-AS1 |
| ILF3 | ENSG00000270061 | RP11-214K3.19 |
| ILF3 | ENSG00000021574 | SPAST |
| ILF3 | ENSG00000145592 | RPL37 |
| ILF3 | ENSG00000122678 | POLM |
| ILF3 | ENSG00000224645 | RP11-126K1.8 |
| ILF3 | ENSG00000105997 | HOXA3 |
| ILF3 | ENSG00000277112 | RP11-755J8.1 |
| ILF3 | ENSG00000258343 | RP11-536G4.2 |
| ILF3 | ENSG00000127564 | PKMYT1 |
| ILF3 | ENSG00000211696 | TRGV8 |
| ILF3 | ENSG00000033100 | CHPF2 |
| ILF3 | ENSG00000235763 | SNRPGP5 |
| ILF3 | ENSG00000227191 | TRGC2 |
| ILF3 | ENSG00000131591 | C1orf159 |
| ILF3 | ENSG00000238423 | SNORD42B |
| ILF3 | ENSG00000153310 | FAM49B |
| ILF3 | ENSG00000105887 | MTPN |
| ILF3 | ENSG00000127554 | GFER |
| ILF3 | ENSG00000169062 | UPF3A |
| ILF3 | ENSG00000160888 | IER2 |
| ILF3 | ENSG00000175773 | RP11-121M22.1 |
| ILF3 | ENSG00000103381 | CPPED1 |
| ILF3 | ENSG00000167664 | TMIGD2 |
| ILF3 | ENSG00000266594 | MIR4766 |
| ILF3 | ENSG00000135862 | LAMC1 |
| ILF3 | ENSG00000222222 | RNU2-17P |
| ILF3 | ENSG00000059588 | TARBP1 |
| ILF3 | ENSG00000180822 | PSMG4 |
| ILF3 | ENSG00000160953 | MUM1 |
| ILF3 | ENSG00000133195 | SLC39A11 |
| ILF3 | ENSG00000271052 | RP11-479I16.2 |
| ILF3 | ENSG00000163507 | KIAA1524 |
| ILF3 | ENSG00000066583 | ISOC1 |
| ILF3 | ENSG00000224532 | RP3-470L22.1 |
| ILF3 | ENSG00000252764 | RNU6-1092P |
| ILF3 | ENSG00000159314 | ARHGAP27 |
| ILF3 | ENSG00000165169 | DYNLT3 |
| ILF3 | ENSG00000205485 | AC004980.7 |
| ILF3 | ENSG00000274211 | SOCS7 |
| ILF3 | ENSG00000206888 | RNU6-48P |
| ILF3 | ENSG00000258317 | RP11-603J24.5 |
| ILF3 | ENSG00000257052 | RP11-881M11.2 |
| ILF3 | ENSG00000156162 | DPY19L4 |
| ILF3 | ENSG00000199565 | Y_RNA |
| ILF3 | ENSG00000183530 | PRR14L |
| ILF3 | ENSG00000257702 | LBX2-AS1 |
| ILF3 | ENSG00000140259 | MFAP1 |
| ILF3 | ENSG00000075399 | VPS9D1 |
| ILF3 | ENSG00000267758 | RP11-358B23.5 |

| ILF3 | ENSG00000217130 | RP3-375P9.2 |
| --- | --- | --- |
| ILF3 | ENSG00000025434 | NR1H3 |
| ILF3 | ENSG00000099617 | EFNA2 |
| ILF3 | ENSG00000241217 | RN7SL809P |
| ILF3 | ENSG00000175130 | MARCKSL1 |
| ILF3 | ENSG00000041357 | PSMA4 |
| ILF3 | ENSG00000063854 | HAGH |
| ILF3 | ENSG00000275741 | RP11-40E6.1 |
| ILF3 | ENSG00000266036 | MIR3615 |
| ILF3 | ENSG00000236866 | AL157902.3 |
| ILF3 | ENSG00000215277 | RNF212B |
| ILF3 | ENSG00000100354 | TNRC6B |
| ILF3 | ENSG00000166275 | C10orf32 |
| ILF3 | ENSG00000130590 | SAMD10 |
| ILF3 | ENSG00000143303 | RRNAD1 |
| ILF3 | ENSG00000230107 | CTA-126B4.7 |
| ILF3 | ENSG00000233231 | HNRNPA1P49 |
| ILF3 | ENSG00000198954 | KIAA1279 |
| ILF3 | ENSG00000274678 | RP11-2C24.7 |
| ILF3 | ENSG00000182165 | TP53TG1 |
| ILF3 | ENSG00000087884 | AAMDC |
| ILF3 | ENSG00000214029 | ZNF891 |
| ILF3 | ENSG00000132581 | SDF2 |
| ILF3 | ENSG00000225761 | RP11-417O11.5 |
| ILF3 | ENSG00000201451 | Y_RNA |
| ILF3 | ENSG00000234261 | RP11-146I2.1 |
| ILF3 | ENSG00000148297 | MED22 |
| ILF3 | ENSG00000111875 | ASF1A |
| ILF3 | ENSG00000178694 | NSUN3 |
| ILF3 | ENSG00000158859 | ADAMTS4 |
| ILF3 | ENSG00000231471 | HMGN2P34 |
| ILF3 | ENSG00000149577 | SIDT2 |
| ILF3 | ENSG00000177553 | RP11-56N19.5 |
| ILF3 | ENSG00000257345 | RP11-511B23.1 |
| ILF3 | ENSG00000116221 | MRPL37 |
| ILF3 | ENSG00000267801 | RP11-552F3.9 |
| ILF3 | ENSG00000238061 | RP4-635A23.4 |
| ILF3 | ENSG00000196787 | HIST1H2AG |
| ILF3 | ENSG00000089558 | KCNH4 |
| ILF3 | ENSG00000214331 | RP11-252A24.2 |
| ILF3 | ENSG00000130479 | MAP1S |
| ILF3 | ENSG00000162642 | C1orf52 |
| ILF3 | ENSG00000145020 | AMT |
| ILF3 | ENSG00000172900 | FLJ42102 |
| ILF3 | ENSG00000278916 | CEP83-AS1 |
| ILF3 | ENSG00000269350 | CTD-2278I10.1 |
| ILF3 | ENSG00000117155 | SSX2IP |
| ILF3 | ENSG00000265590 | AP000275.65 |
| ILF3 | ENSG00000111653 | ING4 |
| ILF3 | ENSG00000182712 | CMC4 |
| ILF3 | ENSG00000080031 | PTPRH |

| ILF3 | ENSG00000108256 | NUFIP2 |
| --- | --- | --- |
| ILF3 | ENSG00000164675 | IQUB |
| ILF3 | ENSG00000248932 | RP11-319G6.1 |
| ILF3 | ENSG00000065615 | CYB5R4 |
| ILF3 | ENSG00000146670 | CDCA5 |
| ILF3 | ENSG00000257763 | OR5BK1P |
| ILF3 | ENSG00000233483 | CTD- |
| ILF3 | ENSG00000213014 | 2020K17.4 VN2R17P |
| ILF3 | ENSG00000246250 | RP11-613D13.5 |
| ILF3 | ENSG00000164506 | STXBP5 |
| ILF3 | ENSG00000143434 | SEMA6C |
| ILF3 | ENSG00000221874 | ZNF321P |
| ILF3 | ENSG00000103642 | LACTB |
| ILF3 | ENSG00000170919 | TPT1-AS1 |
| ILF3 | ENSG00000235257 | ITGA9-AS1 |
| ILF3 | ENSG00000221771 | MIR1205 |
| ILF3 | ENSG00000143398 | PIP5K1A |
| ILF3 | ENSG00000259178 | CTD-2184D3.3 |
| ILF3 | ENSG00000075275 | CELSR1 |
| ILF3 | ENSG00000217862 | HIST1H4PS1 |
| ILF3 | ENSG00000187642 | PERM1 |
| ILF3 | ENSG00000116133 | DHCR24 |
| ILF3 | ENSG00000155090 | KLF10 |
| ILF3 | ENSG00000255717 | SNHG1 |
| ILF3 | ENSG00000229327 | RP11-135A24.2 |
| ILF3 | ENSG00000117222 | RBBP5 |
| ILF3 | ENSG00000108506 | INTS2 |
| ILF3 | ENSG00000111358 | GTF2H3 |
| ILF3 | ENSG00000143127 | ITGA10 |
| ILF3 | ENSG00000163159 | VPS72 |
| ILF3 | ENSG00000278099 | U1 |
| ILF3 | ENSG00000258344 | RP11-968A15.8 |
| ILF3 | ENSG00000091136 | LAMB1 |
| ILF3 | ENSG00000271931 | RP11-63L7.5 |
| ILF3 | ENSG00000272894 | RP5-1159O4.1 |
| ILF3 | ENSG00000201162 | RNU6-454P |
| ILF3 | ENSG00000131941 | RHPN2 |
| ILF3 | ENSG00000127561 | SYNGR3 |
| ILF3 | ENSG00000263369 | RP11-640N20.9 |
| ILF3 | ENSG00000224970 | RP11-114L10.2 |
| ILF3 | ENSG00000269400 | CTD-2529P6.4 |
| ILF3 | ENSG00000004700 | RECQL |
| ILF3 | ENSG00000177889 | UBE2N |
| ILF3 | ENSG00000125520 | SLC2A4RG |
| ILF3 | ENSG00000163481 | RNF25 |
| ILF3 | ENSG00000160446 | ZDHHC12 |
| ILF3 | ENSG00000183137 | CEP57L1 |
| ILF3 | ENSG00000279838 | RP4-635A23.6 |
| ILF3 | ENSG00000177614 | PGBD5 |
| ILF3 | ENSG00000137166 | FOXP4 |
| ILF3 | ENSG00000133302 | ANKRD32 |

| ILF3 | ENSG00000163682 | RPL9 |
| --- | --- | --- |
| ILF3 | ENSG00000247735 | CTD-2574D22.2 |
| ILF3 | ENSG00000112759 | SLC29A1 |
| ILF3 | ENSG00000196262 | PPIA |
| ILF3 | ENSG00000226989 | AL049758.2 |
| ILF3 | ENSG00000278852 | NPPA-AS1_2 |
| ILF3 | ENSG00000272824 | RP6-74O6.6 |
| ILF3 | ENSG00000268189 | AC005785.2 |
| ILF3 | ENSG00000239521 | GATS |
| ILF3 | ENSG00000105700 | KXD1 |
| ILF3 | ENSG00000111554 | MDM1 |
| ILF3 | ENSG00000089685 | BIRC5 |
| ILF3 | ENSG00000264187 | RP11-45M22.4 |
| ILF3 | ENSG00000113712 | CSNK1A1 |
| ILF3 | ENSG00000178381 | ZFAND2A |
| ILF3 | ENSG00000173976 | RAX2 |
| ILF3 | ENSG00000116198 | CEP104 |
| ILF3 | ENSG00000129084 | PSMA1 |
| ILF3 | ENSG00000258777 | HIF1A-AS1 |
| ILF3 | ENSG00000270850 | RP11-395N6.4 |
| ILF3 | ENSG00000130244 | FAM98C |
| ILF3 | ENSG00000119401 | TRIM32 |
| ILF3 | ENSG00000270733 | RP11-231P20.5 |
| ILF3 | ENSG00000140995 | DEF8 |
| ILF3 | ENSG00000258101 | RP11-977B10.2 |
| ILF3 | ENSG00000175826 | CTDNEP1 |
| ILF3 | ENSG00000061656 | SPAG4 |
| ILF3 | ENSG00000260352 | CTD-2288F12.1 |
| ILF3 | ENSG00000166825 | ANPEP |
| ILF3 | ENSG00000201965 | Y_RNA |
| ILF3 | ENSG00000255726 | XXbac- |
| ILF3 | ENSG00000278637 | BPG299F13.15  HIST1H4A |
| ILF3 | ENSG00000186918 | ZNF395 |
| ILF3 | ENSG00000204661 | C5orf60 |
| ILF3 | ENSG00000162604 | TM2D1 |
| ILF3 | ENSG00000197324 | LRP10 |
| ILF3 | ENSG00000233230 | AC079807.2 |
| ILF3 | ENSG00000269392 | CTB-191K22.6 |
| ILF3 | ENSG00000179335 | CLK3 |
| ILF3 | ENSG00000074696 | PTPLAD1 |
| ILF3 | ENSG00000171806 | METTL18 |
| ILF3 | ENSG00000199357 | Y_RNA |
| ILF3 | ENSG00000104976 | SNAPC2 |
| ILF3 | ENSG00000267120 | AD000671.6 |
| ILF3 | ENSG00000252797 | RN7SKP272 |
| ILF3 | ENSG00000163798 | SLC4A1AP |
| ILF3 | ENSG00000128923 | FAM63B |
| ILF3 | ENSG00000223886 | RP11-251G23.2 |
| ILF3 | ENSG00000050405 | LIMA1 |
| ILF3 | ENSG00000177971 | IMP3 |
| ILF3 | ENSG00000269228 | CTD-3214H19.12 |

| ILF3 | ENSG00000006634 | DBF4 |
| --- | --- | --- |
| ILF3 | ENSG00000204396 | VWA7 |
| ILF3 | ENSG00000148400 | NOTCH1 |
| ILF3 | ENSG00000279267 | RP11-395L14.18 |
| ILF3 | ENSG00000224977 | RP11-160H22.3 |
| ILF3 | ENSG00000167580 | AQP2 |
| ILF3 | ENSG00000085382 | HACE1 |
| ILF3 | ENSG00000229352 | AC007563.3 |
| ILF3 | ENSG00000266373 | RP11-710M11.1 |
| ILF3 | ENSG00000153406 | NMRAL1 |
| ILF3 | ENSG00000260193 | RP11-83N9.5 |
| ILF3 | ENSG00000001460 | STPG1 |
| ILF3 | ENSG00000221025 | MIR1250 |
| ILF3 | ENSG00000164329 | PAPD4 |
| ILF3 | ENSG00000235836 | AC124944.4 |
| ILF3 | ENSG00000102763 | VWA8 |
| ILF3 | ENSG00000228380 | AC016697.2 |
| ILF3 | ENSG00000100784 | RPS6KA5 |
| ILF3 | ENSG00000129911 | KLF16 |
| ILF3 | ENSG00000273724 | RP11-347C12.12 |
| ILF3 | ENSG00000255553 | RP11-810P12.5 |
| ILF3 | ENSG00000128596 | CCDC136 |
| ILF3 | ENSG00000207630 | MIR7-3 |
| ILF3 | ENSG00000215088 | RPS5P3 |
| ILF3 | ENSG00000177455 | CD19 |
| ILF3 | ENSG00000108439 | PNPO |
| ILF3 | ENSG00000176473 | WDR25 |
| ILF3 | ENSG00000111652 | COPS7A |
| ILF3 | ENSG00000107175 | CREB3 |
| ILF3 | ENSG00000216937 | CCDC7 |
| ILF3 | ENSG00000243704 | RN7SL105P |
| ILF3 | ENSG00000198042 | MAK16 |
| ILF3 | ENSG00000265453 | RP11-173M1.3 |
| ILF3 | ENSG00000130724 | CHMP2A |
| ILF3 | ENSG00000043143 | JADE2 |
| ILF3 | ENSG00000121897 | LIAS |
| ILF3 | ENSG00000277947 | SNORD3D |
| ILF3 | ENSG00000069424 | KCNAB2 |
| ILF3 | ENSG00000266313 | RP11-173M1.4 |
| ILF3 | ENSG00000080298 | RFX3 |
| ILF3 | ENSG00000215148 | PRSS41 |
| ILF3 | ENSG00000117751 | PPP1R8 |
| ILF3 | ENSG00000160194 | NDUFV3 |
| ILF3 | ENSG00000105479 | CCDC114 |
| ILF3 | ENSG00000122882 | ECD |
| ILF3 | ENSG00000105202 | FBL |
| ILF3 | ENSG00000122335 | SERAC1 |
| ILF3 | ENSG00000169230 | PRELID1 |
| ILF3 | ENSG00000120333 | MRPS14 |
| ILF3 | ENSG00000129968 | ABHD17A |
| ILF3 | ENSG00000273424 | CTA-223H9.9 |

| ILF3 | ENSG00000083123 | BCKDHB |
| --- | --- | --- |
| ILF3 | ENSG00000085433 | WDR47 |
| ILF3 | ENSG00000013503 | POLR3B |
| ILF3 | ENSG00000198960 | ARMCX6 |
| ILF3 | ENSG00000206967 | Y_RNA |
| ILF3 | ENSG00000213453 | FTH1P3 |
| ILF3 | ENSG00000261055 | RP11-195M16.3 |
| ILF3 | ENSG00000254319 | RP11-134O21.1 |
| ILF3 | ENSG00000275745 | Y_RNA |
| ILF3 | ENSG00000255292 | SDHD |
| ILF3 | ENSG00000182866 | LCK |
| ILF3 | ENSG00000201785 | SNORD117 |
| ILF3 | ENSG00000229016 | RP11-224O19.4 |
| ILF3 | ENSG00000126970 | ZC4H2 |
| ILF3 | ENSG00000186577 | C6orf1 |
| ILF3 | ENSG00000151353 | TMEM18 |
| ILF3 | ENSG00000069275 | NUCKS1 |
| ILF3 | ENSG00000123219 | CENPK |
| ILF3 | ENSG00000264113 | RN7SL784P |
| ILF3 | ENSG00000264210 | MIR4716 |
| ILF3 | ENSG00000254413 | CHKB-CPT1B |
| ILF3 | ENSG00000223653 | RP11-131L23.1 |
| ILF3 | ENSG00000117408 | IPO13 |
| ILF3 | ENSG00000101417 | PXMP4 |
| ILF3 | ENSG00000139921 | TMX1 |
| ILF3 | ENSG00000143442 | POGZ |
| ILF3 | ENSG00000260035 | CTD- |
| ILF3 | ENSG00000186594 | 2651B20.6 MIR22HG |
| ILF3 | ENSG00000231789 | PIK3CD-AS2 |
| ILF3 | ENSG00000163517 | HDAC11 |
| ILF3 | ENSG00000099817 | POLR2E |
| ILF3 | ENSG00000277524 | SMAD5-AS1_3 |
| ILF3 | ENSG00000196531 | NACA |
| ILF3 | ENSG00000258704 | SRP54-AS1 |
| ILF3 | ENSG00000207265 | Y_RNA |
| ILF3 | ENSG00000212221 | RNU6-220P |
| ILF3 | ENSG00000181045 | SLC26A11 |
| ILF3 | ENSG00000229431 | RP1-92O14.6 |
| ILF3 | ENSG00000162601 | MYSM1 |
| ILF3 | ENSG00000228474 | OST4 |
| ILF3 | ENSG00000124067 | SLC12A4 |
| ILF3 | ENSG00000108064 | TFAM |
| ILF3 | ENSG00000278527 | SNORD22 |
| ILF3 | ENSG00000105726 | ATP13A1 |
| ILF3 | ENSG00000106245 | BUD31 |
| ILF3 | ENSG00000139725 | RHOF |
| ILF3 | ENSG00000008710 | PKD1 |
| ILF3 | ENSG00000148225 | WDR31 |
| ILF3 | ENSG00000125534 | PPDPF |
| ILF3 | ENSG00000279187 | RP11-455O6.5 |
| ILF3 | ENSG00000198492 | YTHDF2 |

| ILF3 | ENSG00000170542 | SERPINB9 |
| --- | --- | --- |
| ILF3 | ENSG00000099910 | KLHL22 |
| ILF3 | ENSG00000231725 | VN1R110P |
| ILF3 | ENSG00000223254 | Y_RNA |
| ILF3 | ENSG00000169223 | LMAN2 |
| ILF3 | ENSG00000078403 | MLLT10 |
| ILF3 | ENSG00000122218 | COPA |
| ILF3 | ENSG00000149930 | TAOK2 |
| ILF3 | ENSG00000164050 | PLXNB1 |
| ILF3 | ENSG00000179085 | DPM3 |
| ILF3 | ENSG00000135316 | SYNCRIP |
| ILF3 | ENSG00000132692 | BCAN |
| ILF3 | ENSG00000208018 | MIR645 |
| ILF3 | ENSG00000138663 | COPS4 |
| ILF3 | ENSG00000106609 | TMEM248 |
| ILF3 | ENSG00000112031 | MTRF1L |
| ILF3 | ENSG00000076513 | ANKRD13A |
| ILF3 | ENSG00000233184 | RP11-421L21.3 |
| ILF3 | ENSG00000250222 | CTC-338M12.5 |
| ILF3 | ENSG00000155850 | SLC26A2 |
| ILF3 | ENSG00000261305 | RP4-584D14.7 |
| ILF3 | ENSG00000214293 | APTR |
| ILF3 | ENSG00000230951 | GPS2P2 |
| ILF3 | ENSG00000269980 | RP11-282O18.7 |
| ILF3 | ENSG00000164291 | ARSK |
| ILF3 | ENSG00000139350 | NEDD1 |
| ILF3 | ENSG00000115415 | STAT1 |
| ILF3 | ENSG00000184916 | JAG2 |
| ILF3 | ENSG00000155229 | MMS19 |
| ILF3 | ENSG00000146373 | RNF217 |
| ILF3 | ENSG00000260077 | RP11-254F7.2 |
| ILF3 | ENSG00000135776 | ABCB10 |
| ILF3 | ENSG00000174720 | LARP7 |
| ILF3 | ENSG00000131018 | SYNE1 |
| ILF3 | ENSG00000091039 | OSBPL8 |
| ILF3 | ENSG00000261471 | RP11-61F12.1 |
| ILF3 | ENSG00000092445 | TYRO3 |
| ILF3 | ENSG00000156508 | EEF1A1 |
| ILF3 | ENSG00000235627 | SNRPFP4 |
| ILF3 | ENSG00000112282 | MED23 |
| ILF3 | ENSG00000179632 | MAF1 |
| ILF3 | ENSG00000228606 | RP11-574F21.2 |
| ILF3 | ENSG00000228589 | SPCS2P4 |
| ILF3 | ENSG00000087206 | UIMC1 |
| ILF3 | ENSG00000132341 | RAN |
| ILF3 | ENSG00000277887 | SNORA76C |
| ILF3 | ENSG00000174738 | NR1D2 |
| ILF3 | ENSG00000181408 | UTS2R |
| ILF3 | ENSG00000036530 | CYP46A1 |
| ILF3 | ENSG00000239557 | RP11-168J18.6 |
| ILF3 | ENSG00000164068 | RNF123 |

| ILF3 | ENSG00000102189 | EEA1 |
| --- | --- | --- |
| ILF3 | ENSG00000197261 | C6orf141 |
| ILF3 | ENSG00000244515 | KRT18P34 |
| ILF3 | ENSG00000095209 | TMEM38B |
| ILF3 | ENSG00000201868 | Y_RNA |
| ILF3 | ENSG00000239143 | Y_RNA |
| ILF3 | ENSG00000018699 | TTC27 |
| ILF3 | ENSG00000224015 | AC063976.3 |
| ILF3 | ENSG00000229388 | RP11-442N24 B.1 |
| ILF3 | ENSG00000164466 | SFXN1 |
| ILF3 | ENSG00000279692 | RP11-1055B8.1 |
| ILF3 | ENSG00000205937 | RNPS1 |
| ILF3 | ENSG00000111667 | USP5 |
| ILF3 | ENSG00000207357 | RNU6-2 |
| ILF3 | ENSG00000065717 | TLE2 |
| ILF3 | ENSG00000217030 | RP11-428J1.2 |
| ILF3 | ENSG00000165775 | FUNDC2 |
| ILF3 | ENSG00000260669 | AL136419.6 |
| ILF3 | ENSG00000223538 | RP11-216P16.6 |
| ILF3 | ENSG00000153291 | SLC25A27 |
| ILF3 | ENSG00000261373 | VPS9D1-AS1 |
| ILF3 | ENSG00000038219 | BOD1L1 |
| ILF3 | ENSG00000232907 | DLGAP4-AS1 |
| ILF3 | ENSG00000255529 | POLR2M |
| ILF3 | ENSG00000262962 | KARSP3 |
| ILF3 | ENSG00000168904 | LRRC28 |
| ILF3 | ENSG00000109606 | DHX15 |
| ILF3 | ENSG00000267041 | ZNF850 |
| ILF3 | ENSG00000279579 | bP-2189O9.3 |
| ILF3 | ENSG00000214870 | AC004540.5 |
| ILF3 | ENSG00000234444 | ZNF736 |
| ILF3 | ENSG00000249715 | FER1L5 |
| ILF3 | ENSG00000255201 | RP11-350N15.4 |
| ILF3 | ENSG00000242798 | RP11-506M12.1 |
| ILF3 | ENSG00000246394 | RP11-386I8.6 |
| ILF3 | ENSG00000155085 | AK9 |
| ILF3 | ENSG00000266210 | RN7SL371P |
| ILF3 | ENSG00000276524 | RP11-323F24.3 |
| ILF3 | ENSG00000177736 | RP11-474P12.3 |
| ILF3 | ENSG00000184047 | DIABLO |
| ILF3 | ENSG00000241135 | LINC00881 |
| ILF3 | ENSG00000111832 | RWDD1 |
| ILF3 | ENSG00000126107 | HECTD3 |
| ILF3 | ENSG00000118160 | SLC8A2 |
| ILF3 | ENSG00000115221 | ITGB6 |
| ILF3 | ENSG00000151468 | CCDC3 |
| ILF3 | ENSG00000207392 | SNORA20 |
| ILF3 | ENSG00000260247 | SUB1P4 |
| ILF3 | ENSG00000158571 | PFKFB1 |
| ILF3 | ENSG00000075218 | GTSE1 |
| ILF3 | ENSG00000273080 | RP11-301O19.1 |

| ILF3 | ENSG00000252671 | Y_RNA |
| --- | --- | --- |
| ILF3 | ENSG00000062194 | GPBP1 |
| ILF3 | ENSG00000238160 | AC116366.5 |
| ILF3 | ENSG00000081721 | DUSP12 |
| ILF3 | ENSG00000140961 | OSGIN1 |
| ILF3 | ENSG00000231702 | RP11-54O7.10 |
| ILF3 | ENSG00000140798 | ABCC12 |
| ILF3 | ENSG00000265386 | RN7SL219P |
| ILF3 | ENSG00000109220 | CHIC2 |
| ILF3 | ENSG00000010803 | SCMH1 |
| ILF3 | ENSG00000273149 | RP11-290D2.6 |
| ILF3 | ENSG00000163584 | RPL22L1 |
| ILF3 | ENSG00000121716 | PILRB |
| ILF3 | ENSG00000088298 | EDEM2 |
| ILF3 | ENSG00000108883 | EFTUD2 |
| ILF3 | ENSG00000172171 | TEFM |
| ILF3 | ENSG00000250106 | ANKRD33B- |
| ILF3 | ENSG00000141642 | AS1  ELAC1 |
| ILF3 | ENSG00000196502 | SULT1A1 |
| ILF3 | ENSG00000142409 | ZNF787 |
| ILF3 | ENSG00000273403 | RP11-329B9.3 |
| ILF3 | ENSG00000105939 | ZC3HAV1 |
| ILF3 | ENSG00000252113 | RNU6-523P |
| ILF3 | ENSG00000112365 | ZBTB24 |
| ILF3 | ENSG00000163995 | ABLIM2 |
| ILF3 | ENSG00000223782 | RPS21P8 |
| ILF3 | ENSG00000164258 | NDUFS4 |
| ILF3 | ENSG00000018189 | RUFY3 |
| ILF3 | ENSG00000198356 | ASNA1 |
| ILF3 | ENSG00000222685 | RN7SKP119 |
| ILF3 | ENSG00000278705 | HIST1H4B |
| ILF3 | ENSG00000213865 | C8orf44 |
| ILF3 | ENSG00000173898 | SPTBN2 |
| ILF3 | ENSG00000185753 | CXorf38 |
| ILF3 | ENSG00000188938 | FAM120AOS |
| ILF3 | ENSG00000109919 | MTCH2 |
| ILF3 | ENSG00000254505 | CHMP4A |
| ILF3 | ENSG00000231024 | AC092431.3 |
| ILF3 | ENSG00000138823 | MTTP |
| ILF3 | ENSG00000260260 | SNHG19 |
| ILF3 | ENSG00000267419 | CTC-559E9.6 |
| ILF3 | ENSG00000260932 | RP11-483P21.6 |
| ILF3 | ENSG00000152700 | SAR1B |
| ILF3 | ENSG00000185955 | C7orf61 |
| ILF3 | ENSG00000275635 | U7 |
| ILF3 | ENSG00000176919 | C8G |
| ILF3 | ENSG00000223375 | RP11-224O19.5 |
| ILF3 | ENSG00000155097 | ATP6V1C1 |
| ILF3 | ENSG00000155099 | TMEM55A |
| ILF3 | ENSG00000112139 | MDGA1 |
| ILF3 | ENSG00000269792 | CTB-60E11.9 |

| ILF3 | ENSG00000125484 | GTF3C4 |
| --- | --- | --- |
| ILF3 | ENSG00000270103 | RNU11 |
| ILF3 | ENSG00000159199 | ATP5G1 |
| ILF3 | ENSG00000239106 | Y_RNA |
| ILF3 | ENSG00000279069 | RP11-159D12.6 |
| ILF3 | ENSG00000172845 | SP3 |
| ILF3 | ENSG00000280287 | RP13-554M15.7 |
| ILF3 | ENSG00000264583 | MIR4487 |
| ILF3 | ENSG00000201733 | SNORA43 |
| ILF3 | ENSG00000226933 | NRBF2P2 |
| ILF3 | ENSG00000264999 | MIR5089 |
| ILF3 | ENSG00000126524 | SBDS |
| ILF3 | ENSG00000206897 | SNORA9 |
| ILF3 | ENSG00000263361 | MIR378H |
| ILF3 | ENSG00000104936 | DMPK |
| ILF3 | ENSG00000239215 | RPL27P12 |
| ILF3 | ENSG00000087266 | SH3BP2 |
| ILF3 | ENSG00000258591 | RP11-545M17.3 |
| ILF3 | ENSG00000165156 | ZHX1 |
| ILF3 | ENSG00000264862 | RN7SL45P |
| ILF3 | ENSG00000189419 | SPATA41 |
| ILF3 | ENSG00000025770 | NCAPH2 |
| ILF3 | ENSG00000231852 | CYP21A2 |
| ILF3 | ENSG00000257576 | RP11-153M3.1 |
| ILF3 | ENSG00000099957 | P2RX6 |
| ILF3 | ENSG00000055483 | USP36 |
| ILF3 | ENSG00000263312 | RP11-459C13.1 |
| ILF3 | ENSG00000263201 | CTC-479C5.6 |
| ILF3 | ENSG00000269054 | CTD-2619J13.3 |
| ILF3 | ENSG00000066135 | KDM4A |
| ILF3 | ENSG00000135090 | TAOK3 |
| ILF3 | ENSG00000131480 | AOC2 |
| ILF3 | ENSG00000170892 | TSEN34 |
| ILF3 | ENSG00000142444 | C19orf52 |
| ILF3 | ENSG00000182196 | ARL6IP4 |
| ILF3 | ENSG00000275223 | RP1-63M2.7 |
| ILF3 | ENSG00000278399 | RP11-74M13.5 |
| ILF3 | ENSG00000131979 | GCH1 |
| ILF3 | ENSG00000103043 | VAC14 |
| ILF3 | ENSG00000199325 | RNU4-39P |
| ILF3 | ENSG00000100918 | REC8 |
| ILF3 | ENSG00000196338 | NLGN3 |
| ILF3 | ENSG00000256364 | RP11-173P15.3 |
| ILF3 | ENSG00000153487 | ING1 |
| ILF3 | ENSG00000123552 | USP45 |
| ILF3 | ENSG00000252334 | RNU6-1337P |
| ILF3 | ENSG00000203778 | FAM229B |
| ILF3 | ENSG00000188596 | CFAP54 |
| ILF3 | ENSG00000180694 | TMEM64 |
| ILF3 | ENSG00000160271 | RALGDS |
| ILF3 | ENSG00000165097 | KDM1B |

| ILF3 | ENSG00000130307 | USHBP1 |
| --- | --- | --- |
| ILF3 | ENSG00000154582 | TCEB1 |
| ILF3 | ENSG00000165630 | PRPF18 |
| ILF3 | ENSG00000165672 | PRDX3 |
| ILF3 | ENSG00000227834 | AP003385.2 |
| ILF3 | ENSG00000264961 | MIR4730 |
| ILF3 | ENSG00000163577 | EIF5A2 |
| ILF3 | ENSG00000225075 | RP11-426L16.3 |
| ILF3 | ENSG00000152133 | GPATCH11 |
| ILF3 | ENSG00000067704 | IARS2 |
| ILF3 | ENSG00000273257 | RP11-177J6.1 |
| ILF3 | ENSG00000099203 | TMED1 |
| ILF3 | ENSG00000273003 | ARL2-SNX15 |
| ILF3 | ENSG00000243979 | RP11-347C18.1 |
| ILF3 | ENSG00000252660 | Y_RNA |
| ILF3 | ENSG00000102145 | GATA1 |
| ILF3 | ENSG00000084636 | COL16A1 |
| ILF3 | ENSG00000022277 | RTFDC1 |
| ILF3 | ENSG00000163565 | IFI16 |
| ILF3 | ENSG00000260063 | RP5-968P14.2 |
| ILF3 | ENSG00000280247 | AC005578.3 |
| ILF3 | ENSG00000258976 | CTD-2207P18.2 |
| ILF3 | ENSG00000134758 | RNF138 |
| ILF3 | ENSG00000160049 | DFFA |
| ILF3 | ENSG00000135108 | FBXO21 |
| ILF3 | ENSG00000261916 | RP11-235E17.4 |
| ILF3 | ENSG00000256525 | POLG2 |
| ILF3 | ENSG00000139719 | VPS33A |
| ILF3 | ENSG00000105613 | MAST1 |
| ILF3 | ENSG00000272854 | RP4-593H12.1 |
| ILF3 | ENSG00000248993 | XXbac- |
| ILF3 | ENSG00000089693 | BPG181M17.5  MLF2 |
| ILF3 | ENSG00000100949 | RABGGTA |
| ILF3 | ENSG00000226645 | AP006216.10 |
| ILF3 | ENSG00000267011 | CTB-50L17.16 |
| ILF3 | ENSG00000230530 | LIMD1-AS1 |
| ILF3 | ENSG00000243710 | CFAP57 |
| ILF3 | ENSG00000108423 | TUBD1 |
| ILF3 | ENSG00000161958 | FGF11 |
| ILF3 | ENSG00000199906 | RNU5B-2P |
| ILF3 | ENSG00000096088 | PGC |
| ILF3 | ENSG00000233954 | UQCRHL |
| ILF3 | ENSG00000213892 | CEACAM16 |
| ILF3 | ENSG00000109670 | FBXW7 |
| ILF3 | ENSG00000163319 | MRPS18C |
| ILF3 | ENSG00000068028 | RASSF1 |
| ILF3 | ENSG00000149600 | COMMD7 |
| ILF3 | ENSG00000101193 | GID8 |
| ILF3 | ENSG00000198730 | CTR9 |
| ILF3 | ENSG00000224110 | MTRF1LP2 |
| ILF3 | ENSG00000262333 | HNRNPA1P16 |

| ILF3 | ENSG00000274428 | U1 |
| --- | --- | --- |
| ILF3 | ENSG00000234311 | RP11-432J24.3 |
| ILF3 | ENSG00000198182 | ZNF607 |
| ILF3 | ENSG00000179958 | DCTPP1 |
| ILF3 | ENSG00000260448 | LCMT1-AS1 |
| ILF3 | ENSG00000148180 | GSN |
| ILF3 | ENSG00000167333 | TRIM68 |
| ILF3 | ENSG00000264399 | MIR4736 |
| ILF3 | ENSG00000169499 | PLEKHA2 |
| ILF3 | ENSG00000259001 | RPPH1 |
| ILF3 | ENSG00000141570 | CBX8 |
| ILF3 | ENSG00000273361 | RP11-378A13.2 |
| ILF3 | ENSG00000266208 | CTD- |
| ILF3 | ENSG00000131871 | 2267D19.3 VIMP |
| ILF3 | ENSG00000164346 | NSA2 |
| ILF3 | ENSG00000252690 | SCARNA15 |
| ILF3 | ENSG00000162889 | MAPKAPK2 |
| ILF3 | ENSG00000160208 | RRP1B |
| ILF3 | ENSG00000234961 | RP11-124N14.3 |
| ILF3 | ENSG00000161267 | BDH1 |
| ILF3 | ENSG00000095574 | IKZF5 |
| ILF3 | ENSG00000105325 | FZR1 |
| ILF3 | ENSG00000110066 | SUV420H1 |
| ILF3 | ENSG00000189180 | ZNF33A |
| ILF3 | ENSG00000143013 | LMO4 |
| ILF3 | ENSG00000174628 | IQCK |
| ILF3 | ENSG00000257449 | RP11-603J24.4 |
| ILF3 | ENSG00000162576 | MXRA8 |
| ILF3 | ENSG00000221296 | MIR548T |
| ILF3 | ENSG00000137198 | GMPR |
| ILF3 | ENSG00000171469 | ZNF561 |
| ILF3 | ENSG00000141971 | MVB12A |
| ILF3 | ENSG00000198326 | TMEM239 |
| ILF3 | ENSG00000130706 | ADRM1 |
| ILF3 | ENSG00000161654 | LSM12 |
| ILF3 | ENSG00000157992 | KRTCAP3 |
| ILF3 | ENSG00000137040 | RANBP6 |
| ILF3 | ENSG00000260030 | RP11-686F15.2 |
| ILF3 | ENSG00000268931 | RP11-886P16.6 |
| ILF3 | ENSG00000174456 | C12orf76 |
| ILF3 | ENSG00000145982 | FARS2 |
| ILF3 | ENSG00000272030 | RP1-178F15.4 |
| ILF3 | ENSG00000146067 | FAM193B |
| ILF3 | ENSG00000173991 | TCAP |
| ILF3 | ENSG00000233058 | LINC00884 |
| ILF3 | ENSG00000113296 | THBS4 |
| ILF3 | ENSG00000261971 | RP11-473M20.7 |
| ILF3 | ENSG00000205794 | RP11-333E13.4 |
| ILF3 | ENSG00000138614 | VWA9 |
| ILF3 | ENSG00000247746 | USP51 |
| ILF3 | ENSG00000023228 | NDUFS1 |

| ILF3 | ENSG00000277234 | U1 |
| --- | --- | --- |
| ILF3 | ENSG00000259642 | ST20-AS1 |
| ILF3 | ENSG00000171522 | PTGER4 |
| ILF3 | ENSG00000145604 | SKP2 |
| ILF3 | ENSG00000261183 | RP11-532F12.5 |
| ILF3 | ENSG00000183048 | SLC25A10 |
| ILF3 | ENSG00000206337 | HCP5 |
| ILF3 | ENSG00000157911 | PEX10 |
| ILF3 | ENSG00000230454 | U73166.2 |
| ILF3 | ENSG00000102225 | CDK16 |
| ILF3 | ENSG00000225005 | RP11-483G21.3 |
| ILF3 | ENSG00000261595 | RP11-426L16.9 |
| ILF3 | ENSG00000207187 | SNORA64 |
| ILF3 | ENSG00000237980 | RP11-338C15.3 |
| ILF3 | ENSG00000231584 | FAHD2CP |
| ILF3 | ENSG00000250195 | RP11-371F15.3 |
| ILF3 | ENSG00000252074 | RNU6-416P |
| ILF3 | ENSG00000250833 | DNM1P17 |
| ILF3 | ENSG00000187922 | LCN10 |
| ILF3 | ENSG00000226872 | AC002472.11 |
| ILF3 | ENSG00000076706 | MCAM |
| ILF3 | ENSG00000090905 | TNRC6A |
| ILF3 | ENSG00000277283 | RP1-267D11.6 |
| ILF3 | ENSG00000165832 | TRUB1 |
| ILF3 | ENSG00000132128 | LRRC41 |
| ILF3 | ENSG00000180999 | C1orf105 |
| ILF3 | ENSG00000183431 | SF3A3 |
| ILF3 | ENSG00000237371 | RP13-152O15.5 |
| ILF3 | ENSG00000117862 | TXNDC12 |
| ILF3 | ENSG00000276584 | MIR6737 |
| ILF3 | ENSG00000258996 | CTD- |
| ILF3 | ENSG00000132024 | 2315A10.1  CC2D1A |
| ILF3 | ENSG00000250882 | CTC-459M5.1 |
| ILF3 | ENSG00000184497 | TMEM255B |
| ILF3 | ENSG00000068697 | LAPTM4A |
| ILF3 | ENSG00000172748 | ZNF596 |
| ILF3 | ENSG00000163884 | KLF15 |
| ILF3 | ENSG00000277566 | RP11-545P7.9 |
| ILF3 | ENSG00000198919 | DZIP3 |
| ILF3 | ENSG00000184949 | FAM227A |
| ILF3 | ENSG00000273828 | RP11-394O2.3 |
| ILF3 | ENSG00000176349 | AC110781.3 |
| ILF3 | ENSG00000063438 | AHRR |
| ILF3 | ENSG00000146054 | TRIM7 |
| ILF3 | ENSG00000137776 | SLTM |
| ILF3 | ENSG00000086232 | EIF2AK1 |
| ILF3 | ENSG00000224509 | AC010884.1 |
| ILF3 | ENSG00000254245 | PCDHGA3 |
| ILF3 | ENSG00000262223 | RP11-1055B8.3 |
| ILF3 | ENSG00000251778 | SNORA3 |
| ILF3 | ENSG00000204971 | RP11-807H22.7 |

| ILF3 | ENSG00000196172 | ZNF681 |
| --- | --- | --- |
| ILF3 | ENSG00000136950 | ARPC5L |
| ILF3 | ENSG00000187775 | DNAH17 |
| ILF3 | ENSG00000267363 | CTD-3162L10.4 |
| ILF3 | ENSG00000167840 | ZNF232 |
| ILF3 | ENSG00000213901 | SLC23A3 |
| ILF3 | ENSG00000125089 | SH3TC1 |
| ILF3 | ENSG00000204435 | CSNK2B |
| ILF3 | ENSG00000108106 | UBE2S |
| ILF3 | ENSG00000258260 | RP11-977G19.14 |
| ILF3 | ENSG00000168005 | C11orf84 |
| ILF3 | ENSG00000261737 | RP4-612B15.3 |
| ILF3 | ENSG00000221496 | U3 |
| ILF3 | ENSG00000251920 | RNA5SP216 |
| ILF3 | ENSG00000170890 | PLA2G1B |
| ILF3 | ENSG00000122692 | SMU1 |
| ILF3 | ENSG00000095752 | IL11 |
| ILF3 | ENSG00000269584 | TDGF1P7 |
| ILF3 | ENSG00000253948 | RP11-410L14.2 |
| ILF3 | ENSG00000241720 | RP4-735C1.4 |
| ILF3 | ENSG00000240449 | RP4-584D14.5 |
| ILF3 | ENSG00000160299 | PCNT |
| ILF3 | ENSG00000277072 | STAG3L2 |
| ILF3 | ENSG00000272114 | RP1-261G23.7 |
| ILF3 | ENSG00000231359 | AC072052.7 |
| ILF3 | ENSG00000207507 | RNU6-9 |
| ILF3 | ENSG00000176485 | PLA2G16 |
| ILF3 | ENSG00000138381 | ASNSD1 |
| ILF3 | ENSG00000169567 | HINT1 |
| ILF3 | ENSG00000261349 | RP3-465N24.5 |
| ILF3 | ENSG00000198105 | ZNF248 |
| ILF3 | ENSG00000184111 | RP11-366I13.3 |
| ILF3 | ENSG00000156976 | EIF4A2 |
| ILF3 | ENSG00000178202 | KDELC2 |
| ILF3 | ENSG00000238012 | AC114752.1 |
| ILF3 | ENSG00000143320 | CRABP2 |
| ILF3 | ENSG00000222629 | RNU2-42P |
| ILF3 | ENSG00000259138 | RP11-950C14.7 |
| ILF3 | ENSG00000237232 | ZNF295-AS1 |
| ILF3 | ENSG00000152556 | PFKM |
| ILF3 | ENSG00000234450 | AC005534.6 |
| ILF3 | ENSG00000254854 | CTD-2523D13.2 |
| ILF3 | ENSG00000206783 | RNU6-292P |
| ILF3 | ENSG00000259547 | CYCSP2 |
| ILF3 | ENSG00000162441 | LZIC |
| ILF3 | ENSG00000269743 | SLC25A53 |
| ILF3 | ENSG00000183773 | AIFM3 |
| ILF3 | ENSG00000137818 | RPLP1 |
| ILF3 | ENSG00000120049 | KCNIP2 |
| ILF3 | ENSG00000259728 | LINC00933 |
| ILF3 | ENSG00000249148 | AC006445.7 |

| ILF3 | ENSG00000088543 | C3orf18 |
| --- | --- | --- |
| ILF3 | ENSG00000152443 | ZNF776 |
| ILF3 | ENSG00000270117 | AP000769.7 |
| ILF3 | ENSG00000134265 | NAPG |
| ILF3 | ENSG00000131725 | WDR44 |
| ILF3 | ENSG00000147316 | MCPH1 |
| ILF3 | ENSG00000262691 | CTC-277H1.7 |
| ILF3 | ENSG00000163029 | SMC6 |
| ILF3 | ENSG00000249241 | AC195454.1 |
| ILF3 | ENSG00000259736 | RP11-387D10.2 |
| ILF3 | ENSG00000100401 | RANGAP1 |
| ILF3 | ENSG00000113845 | TIMMDC1 |
| ILF3 | ENSG00000145113 | MUC4 |
| ILF3 | ENSG00000235066 | AC009303.1 |
| ILF3 | ENSG00000201273 | RNU6-776P |
| ILF3 | ENSG00000275515 | RP11-295D4.7 |
| ILF3 | ENSG00000127989 | MTERF1 |
| ILF3 | ENSG00000197487 | GALP |
| ILF3 | ENSG00000119227 | PIGZ |
| ILF3 | ENSG00000107937 | GTPBP4 |
| ILF3 | ENSG00000112640 | PPP2R5D |
| ILF3 | ENSG00000253404 | AC034243.1 |
| ILF3 | ENSG00000235374 | SSR4P1 |
| ILF3 | ENSG00000163322 | FAM175A |
| ILF3 | ENSG00000224543 | SNRPGP15 |
| ILF3 | ENSG00000267394 | CTB-175E5.7 |
| ILF3 | ENSG00000108785 | HSD17B1P1 |
| ILF3 | ENSG00000175294 | CATSPER1 |
| ILF3 | ENSG00000092841 | MYL6 |
| ILF3 | ENSG00000198342 | ZNF442 |
| ILF3 | ENSG00000204792 | LINC01291 |
| ILF3 | ENSG00000101052 | IFT52 |
| ILF3 | ENSG00000138621 | PPCDC |
| ILF3 | ENSG00000272323 | CTD- |
| ILF3 | ENSG00000109103 | 2517O10.6 UNC119 |
| ILF3 | ENSG00000065600 | TMEM206 |
| ILF3 | ENSG00000207395 | Y_RNA |
| ILF3 | ENSG00000266329 | MIR4281 |
| ILF3 | ENSG00000274507 | RP5-965G21.5 |
| ILF3 | ENSG00000166348 | USP54 |
| ILF3 | ENSG00000152926 | ZNF117 |
| ILF3 | ENSG00000260360 | RP11-533E19.5 |
| ILF3 | ENSG00000159445 | THEM4 |
| ILF3 | ENSG00000263370 | RP11-68I3.5 |
| ILF3 | ENSG00000093000 | NUP50 |
| ILF3 | ENSG00000262155 | RP11-266L9.5 |
| ILF3 | ENSG00000265390 | MIR4999 |
| ILF3 | ENSG00000119899 | SLC17A5 |
| ILF3 | ENSG00000117360 | PRPF3 |
| ILF3 | ENSG00000233436 | BTBD18 |
| ILF3 | ENSG00000196535 | MYO18A |

| ILF3 | ENSG00000162302 | RPS6KA4 |
| --- | --- | --- |
| ILF3 | ENSG00000102931 | ARL2BP |
| ILF3 | ENSG00000153208 | MERTK |
| ILF3 | ENSG00000231911 | TPRKBP1 |
| ILF3 | ENSG00000138036 | DYNC2LI1 |
| ILF3 | ENSG00000151422 | FER |
| ILF3 | ENSG00000156170 | NDUFAF6 |
| ILF3 | ENSG00000268650 | AC068499.10 |
| ILF3 | ENSG00000270149 | RP11-544M22.13 |
| ILF3 | ENSG00000205085 | FAM71F2 |
| ILF3 | ENSG00000054523 | KIF1B |
| ILF3 | ENSG00000269038 | AP001462.6 |
| ILF3 | ENSG00000279821 | RP11-1334A24.5 |
| ILF3 | ENSG00000219700 | PTCHD3P3 |
| ILF3 | ENSG00000204344 | STK19 |
| ILF3 | ENSG00000110876 | SELPLG |
| ILF3 | ENSG00000168071 | CCDC88B |
| ILF3 | ENSG00000272691 | RP11-290M5.4 |
| ILF3 | ENSG00000114982 | KANSL3 |
| ILF3 | ENSG00000275915 | NPPA-AS1_3 |
| ILF3 | ENSG00000164815 | ORC5 |
| ILF3 | ENSG00000144554 | FANCD2 |
| ILF3 | ENSG00000237749 | RP3-423B22.5 |
| ILF3 | ENSG00000130477 | UNC13A |
| ILF3 | ENSG00000162419 | GMEB1 |
| ILF3 | ENSG00000124370 | MCEE |
| ILF3 | ENSG00000103187 | COTL1 |
| ILF3 | ENSG00000185024 | BRF1 |
| ILF3 | ENSG00000259230 | CTD-2555C10.3 |
| ILF3 | ENSG00000229758 | DYNLT3P2 |
| ILF3 | ENSG00000253513 | RP11-6D1.3 |
| ILF3 | ENSG00000089472 | HEPH |
| ILF3 | ENSG00000254531 | FLJ20021 |
| ILF3 | ENSG00000272988 | RP11-150D20.5 |
| ILF3 | ENSG00000090013 | BLVRB |
| ILF3 | ENSG00000127334 | DYRK2 |
| ILF3 | ENSG00000258451 | RP11-903H12.3 |
| ILF3 | ENSG00000240877 | RN7SL521P |
| ILF3 | ENSG00000213592 | AP000662.9 |
| ILF3 | ENSG00000277521 | MIR8078 |
| ILF3 | ENSG00000266578 | RP11-838N2.5 |
| ILF3 | ENSG00000185122 | HSF1 |
| ILF3 | ENSG00000188211 | NCR3LG1 |
| ILF3 | ENSG00000196151 | WDSUB1 |
| ILF3 | ENSG00000223745 | RP4-717I23.3 |
| ILF3 | ENSG00000143933 | CALM2 |
| ILF3 | ENSG00000255384 | RP11-770J1.4 |
| ILF3 | ENSG00000228791 | THRB-AS1 |
| ILF3 | ENSG00000241549 | GUSBP2 |
| ILF3 | ENSG00000116017 | ARID3A |
| ILF3 | ENSG00000271029 | RP11-849F2.10 |

| ILF3 | ENSG00000105370 | LIM2 |
| --- | --- | --- |
| ILF3 | ENSG00000110871 | COQ5 |
| ILF3 | ENSG00000142511 | GPR32 |
| ILF3 | ENSG00000066084 | DIP2B |
| ILF3 | ENSG00000112514 | CUTA |
| ILF3 | ENSG00000189227 | C15orf61 |
| ILF3 | ENSG00000002834 | LASP1 |
| ILF3 | ENSG00000154945 | ANKRD40 |
| ILF3 | ENSG00000260999 | RP11-521L9.1 |
| ILF3 | ENSG00000197769 | MAP1LC3C |
| ILF3 | ENSG00000151458 | ANKRD50 |
| ILF3 | ENSG00000227057 | WDR46 |
| ILF3 | ENSG00000067191 | CACNB1 |
| ILF3 | ENSG00000262668 | AJ003147.9 |
| ILF3 | ENSG00000267385 | CTB-50L17.14 |
| ILF3 | ENSG00000235185 | RP5-1056L3.1 |
| ILF3 | ENSG00000164530 | PI16 |
| ILF3 | ENSG00000260101 | RP11-568N6.1 |
| ILF3 | ENSG00000261725 | RP11-480G7.3 |
| ILF3 | ENSG00000261430 | LA16c-390E6.3 |
| ILF3 | ENSG00000143952 | VPS54 |
| ILF3 | ENSG00000278719 | MCM8-AS1 |
| ILF3 | ENSG00000127328 | RAB3IP |
| ILF3 | ENSG00000243147 | MRPL33 |
| ILF3 | ENSG00000132912 | DCTN4 |
| ILF3 | ENSG00000259682 | RP11-702L15.4 |
| ILF3 | ENSG00000164070 | HSPA4L |
| ILF3 | ENSG00000251692 | PTX4 |
| ILF3 | ENSG00000113522 | RAD50 |
| ILF3 | ENSG00000164442 | CITED2 |
| ILF3 | ENSG00000244005 | NFS1 |
| ILF3 | ENSG00000011304 | PTBP1 |
| ILF3 | ENSG00000126001 | CEP250 |
| ILF3 | ENSG00000272277 | RP1-40E16.12 |
| ILF3 | ENSG00000115594 | IL1R1 |
| ILF3 | ENSG00000257167 | TMPO-AS1 |
| ILF3 | ENSG00000173757 | STAT5B |
| ILF3 | ENSG00000274823 | RP11-348M3.6 |
| ILF3 | ENSG00000063177 | RPL18 |
| ILF3 | ENSG00000066027 | PPP2R5A |
| ILF3 | ENSG00000258740 | RP11-293M10.1 |
| ILF3 | ENSG00000187699 | C2orf88 |
| ILF3 | ENSG00000137502 | RAB30 |
| ILF3 | ENSG00000232879 | RP11-544M22.3 |
| ILF3 | ENSG00000150873 | C2orf50 |
| ILF3 | ENSG00000166801 | FAM111A |
| ILF3 | ENSG00000254873 | RP11-770J1.5 |
| ILF3 | ENSG00000054983 | GALC |
| ILF3 | ENSG00000265134 | MIR3190 |
| ILF3 | ENSG00000115155 | OTOF |
| ILF3 | ENSG00000170260 | ZNF212 |

| ILF3 | ENSG00000280046 | RP11-1099M24.6 |
| --- | --- | --- |
| ILF3 | ENSG00000151806 | GUF1 |
| ILF3 | ENSG00000229122 | AGBL5-IT1 |
| ILF3 | ENSG00000102081 | FMR1 |
| ILF3 | ENSG00000167394 | ZNF668 |
| ILF3 | ENSG00000129646 | QRICH2 |
| ILF3 | ENSG00000182534 | MXRA7 |
| ILF3 | ENSG00000125508 | SRMS |
| ILF3 | ENSG00000145246 | ATP10D |
| ILF3 | ENSG00000124783 | SSR1 |
| ILF3 | ENSG00000180549 | FUT7 |
| ILF3 | ENSG00000163528 | CHCHD4 |
| ILF3 | ENSG00000279204 | RP11-175K6.2 |
| ILF3 | ENSG00000102796 | DHRS12 |
| ILF3 | ENSG00000241673 | RPS27P12 |
| ILF3 | ENSG00000113790 | EHHADH |
| ILF3 | ENSG00000062524 | LTK |
| ILF3 | ENSG00000101546 | RBFA |
| ILF3 | ENSG00000057468 | MSH4 |
| ILF3 | ENSG00000243414 | TICAM2 |
| ILF3 | ENSG00000236137 | RP11-27K13.3 |
| ILF3 | ENSG00000143799 | PARP1 |
| ILF3 | ENSG00000185591 | SP1 |
| ILF3 | ENSG00000116120 | FARSB |
| ILF3 | ENSG00000183011 | NAA38 |
| ILF3 | ENSG00000118307 | CASC1 |
| ILF3 | ENSG00000168404 | MLKL |
| ILF3 | ENSG00000212525 | RNA5SP212 |
| ILF3 | ENSG00000260944 | FOXC2-AS1 |
| ILF3 | ENSG00000113407 | TARS |
| ILF3 | ENSG00000139549 | DHH |
| ILF3 | ENSG00000103326 | CAPN15 |
| ILF3 | ENSG00000160951 | PTGER1 |
| ILF3 | ENSG00000276302 | RP5-874C20.8 |
| ILF3 | ENSG00000235015 | GEMIN2P1 |
| ILF3 | ENSG00000225676 | RP3-438O4.4 |
| ILF3 | ENSG00000162722 | TRIM58 |
| ILF3 | ENSG00000103549 | RNF40 |
| ILF3 | ENSG00000136197 | C7orf25 |
| ILF3 | ENSG00000162227 | TAF6L |
| ILF3 | ENSG00000151093 | OXSM |
| ILF3 | ENSG00000267132 | HMGB3P27 |
| ILF3 | ENSG00000239944 | TRBV8-2 |
| ILF3 | ENSG00000280057 | RP1-168L15.6 |
| ILF3 | ENSG00000160097 | FNDC5 |
| ILF3 | ENSG00000100811 | YY1 |
| ILF3 | ENSG00000185896 | LAMP1 |
| ILF3 | ENSG00000267484 | CTC-518P12.6 |
| ILF3 | ENSG00000228829 | AC005077.5 |
| ILF3 | ENSG00000267852 | CTB-133G6.2 |
| ILF3 | ENSG00000119703 | ZC2HC1C |

| ILF3 | ENSG00000273712 | RP5-874C20.7 |
| --- | --- | --- |
| ILF3 | ENSG00000089351 | GRAMD1A |
| ILF3 | ENSG00000166913 | YWHAB |
| ILF3 | ENSG00000146072 | TNFRSF21 |
| ILF3 | ENSG00000124731 | TREM1 |
| ILF3 | ENSG00000020129 | NCDN |
| ILF3 | ENSG00000108474 | PIGL |
| ILF3 | ENSG00000126246 | IGFLR1 |
| ILF3 | ENSG00000163734 | CXCL3 |
| ILF3 | ENSG00000267938 | EIF1P6 |
| ILF3 | ENSG00000111880 | RNGTT |
| ILF3 | ENSG00000185115 | NDNL2 |
| ILF3 | ENSG00000184863 | RBM33 |
| ILF3 | ENSG00000050426 | LETMD1 |
| ILF3 | ENSG00000228413 | AC024937.2 |
| ILF3 | ENSG00000260107 | AC005606.15 |
| ILF3 | ENSG00000141665 | FBXO15 |
| ILF3 | ENSG00000158104 | HPD |
| ILF3 | ENSG00000266969 | RP11-773H22.4 |
| ILF3 | ENSG00000169641 | LUZP1 |
| ILF3 | ENSG00000162592 | CCDC27 |
| ILF3 | ENSG00000120885 | CLU |
| ILF3 | ENSG00000245534 | RP11-219B17.1 |
| ILF3 | ENSG00000264578 | RP11-360L9.8 |
| ILF3 | ENSG00000102908 | NFAT5 |
| ILF3 | ENSG00000227154 | MKRNP2 |
| ILF3 | ENSG00000124614 | RPS10 |
| ILF3 | ENSG00000188451 | SRP72P2 |
| ILF3 | ENSG00000171236 | LRG1 |
| ILF3 | ENSG00000231185 | AC005592.2 |
| ILF3 | ENSG00000083812 | ZNF324 |
| ILF3 | ENSG00000247240 | UBL7-AS1 |
| ILF3 | ENSG00000237540 | RP11-730A19.7 |
| ILF3 | ENSG00000134852 | CLOCK |
| ILF3 | ENSG00000157227 | MMP14 |
| ILF3 | ENSG00000006125 | AP2B1 |
| ILF3 | ENSG00000227634 | RP11-431K24.3 |
| ILF3 | ENSG00000251246 | RP11-540D14.8 |
| ILF3 | ENSG00000086848 | ALG9 |
| ILF3 | ENSG00000258568 | RHOQP1 |
| ILF3 | ENSG00000261553 | RP11-29G8.3 |
| ILF3 | ENSG00000166848 | TERF2IP |
| ILF3 | ENSG00000168769 | TET2 |
| ILF3 | ENSG00000265564 | PIGPP4 |
| ILF3 | ENSG00000226543 | MYL6P1 |
| ILF3 | ENSG00000278239 | AC020956.3 |
| ILF3 | ENSG00000224916 | APOC4-APOC2 |
| ILF3 | ENSG00000072401 | UBE2D1 |
| ILF3 | ENSG00000123191 | ATP7B |
| ILF3 | ENSG00000279807 | CTC-510F12.3 |
| ILF3 | ENSG00000112742 | TTK |

| ILF3 | ENSG00000201749 | Y_RNA |
| --- | --- | --- |
| ILF3 | ENSG00000274685 | RP11-122K13.15 |
| ILF3 | ENSG00000163738 | MTHFD2L |
| ILF3 | ENSG00000206824 | Y_RNA |
| ILF3 | ENSG00000158246 | FAM46B |
| ILF3 | ENSG00000119335 | SET |
| ILF3 | ENSG00000183250 | LINC01547 |
| ILF3 | ENSG00000261838 | RP11-303E16.6 |
| ILF3 | ENSG00000173267 | SNCG |
| ILF3 | ENSG00000214514 | KRT42P |
| ILF3 | ENSG00000125835 | SNRPB |
| ILF3 | ENSG00000100726 | TELO2 |
| ILF3 | ENSG00000151690 | MFSD6 |
| ILF3 | ENSG00000221604 | MIR1293 |
| ILF3 | ENSG00000227256 | MIS18A-AS1 |
| ILF3 | ENSG00000173406 | DAB1 |
| ILF3 | ENSG00000173456 | RNF26 |
| ILF3 | ENSG00000120137 | PANK3 |
| ILF3 | ENSG00000279259 | RP11-334C17.3 |
| ILF3 | ENSG00000263343 | RP11-388M20.7 |
| ILF3 | ENSG00000115541 | HSPE1 |
| ILF3 | ENSG00000272393 | U6 |
| ILF3 | ENSG00000091129 | NRCAM |
| ILF3 | ENSG00000156026 | MCU |
| ILF3 | ENSG00000143333 | RGS16 |
| ILF3 | ENSG00000173852 | DPY19L1 |
| ILF3 | ENSG00000196453 | ZNF777 |
| ILF3 | ENSG00000123106 | CCDC91 |
| ILF3 | ENSG00000198837 | DENND4B |
| ILF3 | ENSG00000247982 | LINC00926 |
| ILF3 | ENSG00000081320 | STK17B |
| ILF3 | ENSG00000125148 | MT2A |
| ILF3 | ENSG00000252787 | SNORD19 |
| ILF3 | ENSG00000254267 | KB-1458E12.1 |
| ILF3 | ENSG00000179271 | GADD45GIP1 |
| ILF3 | ENSG00000248848 | PPBPP2 |
| ILF3 | ENSG00000074935 | TUBE1 |
| ILF3 | ENSG00000213033 | AURKAPS1 |
| ILF3 | ENSG00000125977 | EIF2S2 |
| ILF3 | ENSG00000161270 | NPHS1 |
| ILF3 | ENSG00000261043 | MIR4313 |
| ILF3 | ENSG00000197921 | HES5 |
| ILF3 | ENSG00000262814 | MRPL12 |
| ILF3 | ENSG00000273549 | Metazoa_SRP |
| ILF3 | ENSG00000238419 | RNU7-186P |
| ILF3 | ENSG00000176222 | ZNF404 |
| ILF3 | ENSG00000173120 | KDM2A |
| ILF3 | ENSG00000207605 | MIR191 |
| ILF3 | ENSG00000259354 | RP11-519G16.3 |
| ILF3 | ENSG00000198331 | HYLS1 |
| ILF3 | ENSG00000123349 | PFDN5 |

| ILF3 | ENSG00000212388 | RNU6-796P |
| --- | --- | --- |
| ILF3 | ENSG00000067167 | TRAM1 |
| ILF3 | ENSG00000271933 | RP11-338I21.1 |
| ILF3 | ENSG00000155846 | PPARGC1B |
| ILF3 | ENSG00000186166 | CCDC84 |
| ILF3 | ENSG00000119402 | FBXW2 |
| ILF3 | ENSG00000207973 | MIR589 |
| ILF3 | ENSG00000007392 | LUC7L |
| ILF3 | ENSG00000169189 | NSMCE1 |
| ILF3 | ENSG00000199347 | RNU5E-1 |
| ILF3 | ENSG00000198003 | CCDC151 |
| ILF3 | ENSG00000074755 | ZZEF1 |
| ILF3 | ENSG00000080986 | NDC80 |
| ILF3 | ENSG00000225138 | CTD-2228K2.7 |
| ILF3 | ENSG00000260488 | RP11-166B2.7 |
| ILF3 | ENSG00000264078 | RP11-73M7.9 |
| ILF3 | ENSG00000090372 | STRN4 |
| ILF3 | ENSG00000140988 | RPS2 |
| ILF3 | ENSG00000200495 | RNU6-1205P |
| ILF3 | ENSG00000101577 | LPIN2 |
| ILF3 | ENSG00000167741 | GGT6 |
| ILF3 | ENSG00000127603 | MACF1 |
| ILF3 | ENSG00000213047 | DENND1B |
| ILF3 | ENSG00000197557 | TTC30A |
| ILF3 | ENSG00000267360 | CTC-454I21.3 |
| ILF3 | ENSG00000130287 | NCAN |
| ILF3 | ENSG00000108387 | SEPT4 |
| ILF3 | ENSG00000199313 | RNU4-82P |
| ILF3 | ENSG00000231999 | FLJ27354 |
| ILF3 | ENSG00000117305 | HMGCL |
| ILF3 | ENSG00000014919 | COX15 |
| ILF3 | ENSG00000278851 | MIR6879 |
| ILF3 | ENSG00000211626 | IGKV6D-41 |
| ILF3 | ENSG00000120899 | PTK2B |
| ILF3 | ENSG00000173960 | UBXN2A |
| ILF3 | ENSG00000267141 | CTB-31O20.8 |
| ILF3 | ENSG00000085733 | CTTN |
| ILF3 | ENSG00000126603 | GLIS2 |
| ILF3 | ENSG00000237714 | P4HA2-AS1 |
| ILF3 | ENSG00000104852 | SNRNP70 |
| ILF3 | ENSG00000169689 | STRA13 |
| ILF3 | ENSG00000106404 | CLDN15 |
| ILF3 | ENSG00000269425 | AC007292.7 |
| ILF3 | ENSG00000276408 | RP11-490B18.5 |
| ILF3 | ENSG00000166261 | ZNF202 |
| ILF3 | ENSG00000136240 | KDELR2 |
| ILF3 | ENSG00000204524 | ZNF805 |
| ILF3 | ENSG00000183569 | SERHL2 |
| ILF3 | ENSG00000271538 | RP11-326I11.4 |
| ILF3 | ENSG00000135424 | ITGA7 |
| ILF3 | ENSG00000156471 | PTDSS1 |

| ILF3 | ENSG00000267001 | AC006538.4 |
| --- | --- | --- |
| ILF3 | ENSG00000256020 | RP5-1154L15.2 |
| ILF3 | ENSG00000146918 | NCAPG2 |
| ILF3 | ENSG00000099337 | KCNK6 |
| ILF3 | ENSG00000259605 | AC074212.5 |
| ILF3 | ENSG00000163689 | C3orf67 |
| ILF3 | ENSG00000152348 | ATG10 |
| ILF3 | ENSG00000271982 | SNORD58B |
| ILF3 | ENSG00000175221 | MED16 |
| ILF3 | ENSG00000107371 | EXOSC3 |
| ILF3 | ENSG00000270022 | RNU12 |
| ILF3 | ENSG00000273391 | RP11-634H22.1 |
| ILF3 | ENSG00000226314 | ZNF192P1 |
| ILF3 | ENSG00000059915 | PSD |
| ILF3 | ENSG00000167977 | KCTD5 |
| ILF3 | ENSG00000238215 | RP11-167P22.5 |
| ILF3 | ENSG00000248774 | RP11-798M19.3 |
| ILF3 | ENSG00000246548 | RP11-7F17.5 |
| ILF3 | ENSG00000275807 | RP11-1348G14.8 |
| ILF3 | ENSG00000264796 | MIR5009 |
| ILF3 | ENSG00000280420 | AC005355.3 |
| ILF3 | ENSG00000262001 | DLGAP1-AS2 |
| ILF3 | ENSG00000097033 | SH3GLB1 |
| ILF3 | ENSG00000165416 | SUGT1 |
| ILF3 | ENSG00000225822 | UBXN7-AS1 |
| ILF3 | ENSG00000122741 | DCAF10 |
| ILF3 | ENSG00000218739 | CEBPZOS |
| ILF3 | ENSG00000228995 | MTND1P9 |
| ILF3 | ENSG00000260100 | RP11-220I1.5 |
| ILF3 | ENSG00000141756 | FKBP10 |
| ILF3 | ENSG00000181649 | PHLDA2 |
| ILF3 | ENSG00000133328 | HRASLS2 |
| ILF3 | ENSG00000187066 | TMEM262 |
| ILF3 | ENSG00000261211 | RP1-80N2.3 |
| ILF3 | ENSG00000216754 | RP1-190J20.2 |
| ILF3 | ENSG00000270542 | RP11-311H10.7 |
| ILF3 | ENSG00000247095 | MIR210HG |
| ILF3 | ENSG00000176472 | ZNF575 |
| ILF3 | ENSG00000167371 | PRRT2 |
| ILF3 | ENSG00000189099 | PRSS48 |
| ILF3 | ENSG00000146192 | FGD2 |
| ILF3 | ENSG00000163040 | CCDC74A |
| ILF3 | ENSG00000198315 | ZKSCAN8 |
| ILF3 | ENSG00000223946 | RP11-533O20.2 |
| ILF3 | ENSG00000105088 | OLFM2 |
| ILF3 | ENSG00000259467 | NDUFAF4P1 |
| ILF3 | ENSG00000262899 | LA16c-360H6.3 |
| ILF3 | ENSG00000235316 | DUSP8P5 |
| ILF3 | ENSG00000250659 | RP11-864I4.3 |
| ILF3 | ENSG00000259315 | RP11-752G15.9 |
| ILF3 | ENSG00000075043 | KCNQ2 |

| ILF3 | ENSG00000205639 | MFSD2B |
| --- | --- | --- |
| ILF3 | ENSG00000131095 | GFAP |
| ILF3 | ENSG00000239544 | RP11-109N23.1 |
| ILF3 | ENSG00000168672 | FAM84B |
| ILF3 | ENSG00000113073 | SLC4A9 |
| ILF3 | ENSG00000223092 | RNA5SP115 |
| ILF3 | ENSG00000115520 | COQ10B |
| ILF3 | ENSG00000266970 | RP11-806H10.4 |
| ILF3 | ENSG00000197536 | C5orf56 |
| ILF3 | ENSG00000201114 | Y_RNA |
| ILF3 | ENSG00000261202 | RP3-496C20.1 |
| ILF3 | ENSG00000270765 | GAS2L2 |
| ILF3 | ENSG00000265236 | SNORD84 |
| ILF3 | ENSG00000140612 | SEC11A |
| ILF3 | ENSG00000234892 | Z82214.2 |
| ILF3 | ENSG00000271046 | RP11-138I18.2 |
| ILF3 | ENSG00000164008 | C1orf50 |
| ILF3 | ENSG00000111412 | C12orf49 |
| ILF3 | ENSG00000166037 | CEP57 |
| ILF3 | ENSG00000104888 | SLC17A7 |
| ILF3 | ENSG00000160401 | C9orf117 |
| ILF3 | ENSG00000112981 | NME5 |
| ILF3 | ENSG00000048405 | ZNF800 |
| ILF3 | ENSG00000100603 | SNW1 |
| ILF3 | ENSG00000144535 | DIS3L2 |
| ILF3 | ENSG00000259802 | CTD-2256P15.2 |
| ILF3 | ENSG00000267457 | RP5-837J1.4 |
| ILF3 | ENSG00000223313 | RNU6-516P |
| ILF3 | ENSG00000215114 | UBXN2B |
| ILF3 | ENSG00000181773 | GPR3 |
| ILF3 | ENSG00000182180 | MRPS16 |
| ILF3 | ENSG00000084207 | GSTP1 |
| ILF3 | ENSG00000137841 | PLCB2 |
| ILF3 | ENSG00000275785 | RP11-111E14.2 |
| ILF3 | ENSG00000126461 | SCAF1 |
| ILF3 | ENSG00000153827 | TRIP12 |
| ILF3 | ENSG00000250234 | CTD-2024P10.1 |
| ILF3 | ENSG00000200344 | Y_RNA |
| ILF3 | ENSG00000061936 | SFSWAP |
| ILF3 | ENSG00000242198 | CTD- |
| ILF3 | ENSG00000162396 | 2235C13.1  PARS2 |
| ILF3 | ENSG00000104522 | TSTA3 |
| ILF3 | ENSG00000243679 | RP11-274B21.3 |
| ILF3 | ENSG00000213963 | AC074286.1 |
| ILF3 | ENSG00000269713 | NBPF9 |
| ILF3 | ENSG00000003393 | ALS2 |
| ILF3 | ENSG00000167553 | TUBA1C |
| ILF3 | ENSG00000006016 | CRLF1 |
| ILF3 | ENSG00000144560 | VGLL4 |
| ILF3 | ENSG00000106538 | RARRES2 |
| ILF3 | ENSG00000266458 | hsa-mir-4259 |

| ILF3 | ENSG00000142534 | RPS11 |
| --- | --- | --- |
| ILF3 | ENSG00000171608 | PIK3CD |
| ILF3 | ENSG00000184678 | HIST2H2BE |
| ILF3 | ENSG00000197959 | DNM3 |
| ILF3 | ENSG00000199516 | Y_RNA |
| ILF3 | ENSG00000257084 | U47924.27 |
| ILF3 | ENSG00000279381 | bP-2171C21.5 |
| ILF3 | ENSG00000229686 | SNORD56 |
| ILF3 | ENSG00000196843 | ARID5A |
| ILF3 | ENSG00000178691 | SUZ12 |
| ILF3 | ENSG00000185267 | CDNF |
| ILF3 | ENSG00000170145 | SIK2 |
| ILF3 | ENSG00000254686 | RP1-276E15.1 |
| ILF3 | ENSG00000235214 | FAM83C-AS1 |
| ILF3 | ENSG00000196436 | NPIPB15 |
| ILF3 | ENSG00000144455 | SUMF1 |
| ILF3 | ENSG00000275901 | Metazoa_SRP |
| ILF3 | ENSG00000108370 | RGS9 |
| ILF3 | ENSG00000137338 | PGBD1 |
| ILF3 | ENSG00000257809 | RP11-603J24.14 |
| ILF3 | ENSG00000169679 | BUB1 |
| ILF3 | ENSG00000255224 | CTD-3065J16.9 |
| ILF3 | ENSG00000214313 | AZGP1P1 |
| ILF3 | ENSG00000111737 | RAB35 |
| ILF3 | ENSG00000143228 | NUF2 |
| ILF3 | ENSG00000118965 | WDR35 |
| ILF3 | ENSG00000186628 | FSD2 |
| ILF3 | ENSG00000252258 | SNORA70 |
| ILF3 | ENSG00000215910 | C1orf167 |
| ILF3 | ENSG00000151532 | VTI1A |
| ILF3 | ENSG00000159753 | RLTPR |
| ILF3 | ENSG00000273523 | RP11-248G5.9 |
| ILF3 | ENSG00000273010 | RP11-96K19.5 |
| ILF3 | ENSG00000267649 | CTD- |
| ILF3 | ENSG00000129691 | 2587H24.10 ASH2L |
| ILF3 | ENSG00000004864 | SLC25A13 |
| ILF3 | ENSG00000079459 | FDFT1 |
| ILF3 | ENSG00000177738 | CTD-2201E18.3 |
| ILF3 | ENSG00000266513 | RP11-874J12.3 |
| ILF3 | ENSG00000270512 | RP5-1136G2.1 |
| ILF3 | ENSG00000270553 | RP11-15E18.5 |
| ILF3 | ENSG00000152749 | GPR180 |
| ILF3 | ENSG00000202408 | RNU1-122P |
| ILF3 | ENSG00000100228 | RAB36 |
| ILF3 | ENSG00000070366 | SMG6 |
| ILF3 | ENSG00000248530 | BCL2L12P1 |
| ILF3 | ENSG00000207459 | RNU6-1311P |
| ILF3 | ENSG00000253390 | CTC-756D1.2 |
| ILF3 | ENSG00000138814 | PPP3CA |
| ILF3 | ENSG00000253716 | RP13-582O9.5 |
| ILF3 | ENSG00000267148 | AC005777.3 |

| ILF3 | ENSG00000128000 | ZNF780B |
| --- | --- | --- |
| ILF3 | ENSG00000187325 | TAF9B |
| ILF3 | ENSG00000184708 | EIF4ENIF1 |
| ILF3 | ENSG00000211451 | GNRHR2 |
| ILF3 | ENSG00000206625 | RNU6-1 |
| ILF3 | ENSG00000128283 | CDC42EP1 |
| ILF3 | ENSG00000114331 | ACAP2 |
| ILF3 | ENSG00000186496 | ZNF396 |
| ILF3 | ENSG00000110911 | SLC11A2 |
| ILF3 | ENSG00000275091 | CTB-55O6.13 |
| ILF3 | ENSG00000107779 | BMPR1A |
| ILF3 | ENSG00000163820 | FYCO1 |
| ILF3 | ENSG00000262074 | SNORD3B-2 |
| ILF3 | ENSG00000118292 | C1orf54 |
| ILF3 | ENSG00000139496 | NUPL1 |
| ILF3 | ENSG00000257355 | CTD- 2006C1.10 |
| ILF3 | ENSG00000138246 | DNAJC13 |
| ILF3 | ENSG00000230457 | PA2G4P4 |
| ILF3 | ENSG00000135486 | HNRNPA1 |
| ILF3 | ENSG00000159592 | GPBP1L1 |
| ILF3 | ENSG00000176225 | RTTN |
| ILF3 | ENSG00000261253 | AC137932.6 |
| ILF3 | ENSG00000233108 | AC006042.7 |
| ILF3 | ENSG00000177383 | MAGEF1 |
| ILF3 | ENSG00000228687 | RP3-419C19.3 |
| ILF3 | ENSG00000076554 | TPD52 |
| ILF3 | ENSG00000272462 | U91328.19 |
| ILF3 | ENSG00000223482 | NUTM2A-AS1 |
| ILF3 | ENSG00000162384 | C1orf123 |
| ILF3 | ENSG00000264553 | MIR4257 |
| ILF3 | ENSG00000104081 | BMF |
| ILF3 | ENSG00000159792 | PSKH1 |
| ILF3 | ENSG00000259350 | RP11-63A23.2 |
| ILF3 | ENSG00000010810 | FYN |
| ILF3 | ENSG00000116584 | ARHGEF2 |
| ILF3 | ENSG00000108651 | UTP6 |
| ILF3 | ENSG00000260997 | RP4-647J21.1 |
| ILF3 | ENSG00000165525 | NEMF |
| ILF3 | ENSG00000189221 | MAOA |
| ILF3 | ENSG00000090263 | MRPS33 |
| ILF3 | ENSG00000238279 | BX470102.3 |
| ILF3 | ENSG00000236546 | RP1-118J21.5 |
| ILF3 | ENSG00000277888 | MIR6846 |
| ILF3 | ENSG00000224988 | RP11-409K20.7 |
| ILF3 | ENSG00000225235 | DDX26B-AS1 |
| ILF3 | ENSG00000249456 | RP11-298J20.4 |
| ILF3 | ENSG00000136098 | NEK3 |
| ILF3 | ENSG00000227627 | RP1-101K10.6 |
| ILF3 | ENSG00000117151 | CTBS |
| ILF3 | ENSG00000271105 | SCML2P2 |
| ILF3 | ENSG00000110660 | SLC35F2 |

| ILF3 | ENSG00000268186 | CTC-241F20.4 |
| --- | --- | --- |
| ILF3 | ENSG00000117614 | SYF2 |
| ILF3 | ENSG00000263316 | RP11-530N7.3 |
| ILF3 | ENSG00000237429 | RP1-159A19.4 |
| ILF3 | ENSG00000280331 | RP11-545D22.1 |
| ILF3 | ENSG00000236527 | ARF4P2 |
| ILF3 | ENSG00000014914 | MTMR11 |
| ILF3 | ENSG00000229862 | RP11-505P4.7 |
| ILF3 | ENSG00000224812 | TMEM72-AS1 |
| ILF3 | ENSG00000261693 | RP13-467H17.1 |
| ILF3 | ENSG00000113742 | CPEB4 |
| ILF3 | ENSG00000105364 | MRPL4 |
| ILF3 | ENSG00000198093 | ZNF649 |
| ILF3 | ENSG00000267507 | CTD-2587H24.1 |
| ILF3 | ENSG00000171488 | LRRC8C |
| ILF3 | ENSG00000091181 | IL5RA |
| ILF3 | ENSG00000162104 | ADCY9 |
| ILF3 | ENSG00000134440 | NARS |
| ILF3 | ENSG00000236144 | TMEM147-AS1 |
| ILF3 | ENSG00000122952 | ZWINT |
| ILF3 | ENSG00000258527 | ASB9P1 |
| ILF3 | ENSG00000187024 | PTRH1 |
| ILF3 | ENSG00000260394 | LA16c-313D11.9 |
| ILF3 | ENSG00000153187 | HNRNPU |
| ILF3 | ENSG00000181638 | ZFP41 |
| ILF3 | ENSG00000218672 | AC008060.7 |
| ILF3 | ENSG00000280239 | CTB-50L17.8 |
| ILF3 | ENSG00000267180 | AC007136.1 |
| ILF3 | ENSG00000160957 | RECQL4 |
| ILF3 | ENSG00000235069 | RP4-785J1.1 |
| ILF3 | ENSG00000102554 | KLF5 |
| ILF3 | ENSG00000199934 | SNORD81 |
| ILF3 | ENSG00000035115 | SH3YL1 |
| ILF3 | ENSG00000106603 | COA1 |
| ILF3 | ENSG00000117724 | CENPF |
| ILF3 | ENSG00000251661 | RP11-326C3.11 |
| ILF3 | ENSG00000256690 | RP11-727F15.9 |
| ILF3 | ENSG00000125630 | POLR1B |
| ILF3 | ENSG00000171227 | TMEM37 |
| ILF3 | ENSG00000132670 | PTPRA |
| ILF3 | ENSG00000178295 | GEN1 |
| ILF3 | ENSG00000162614 | NEXN |
| ILF3 | ENSG00000204538 | PSORS1C2 |
| ILF3 | ENSG00000279613 | RP13-638C3.5 |
| ILF3 | ENSG00000205861 | C1QTNF9B-AS1 |
| ILF3 | ENSG00000201223 | RNU6-1305P |
| ILF3 | ENSG00000163214 | DHX57 |
| ILF3 | ENSG00000236159 | RP3-343K2.4 |
| ILF3 | ENSG00000201255 | RNU6-990P |
| ILF3 | ENSG00000237193 | RP11-275O4.4 |
| ILF3 | ENSG00000132356 | PRKAA1 |

| ILF3 | ENSG00000197568 | HHLA3 |
| --- | --- | --- |
| ILF3 | ENSG00000198363 | ASPH |
| ILF3 | ENSG00000153485 | TMEM251 |
| ILF3 | ENSG00000221740 | SNORD93 |
| ILF3 | ENSG00000234271 | RP1-138B7.4 |
| ILF3 | ENSG00000123080 | CDKN2C |
| ILF3 | ENSG00000075651 | PLD1 |
| ILF3 | ENSG00000231468 | PRDX3P2 |
| ILF3 | ENSG00000185245 | GP1BA |
| ILF3 | ENSG00000223908 | AC068657.2 |
| ILF3 | ENSG00000223271 | Y_RNA |
| ILF3 | ENSG00000179387 | ELMOD2 |
| ILF3 | ENSG00000199293 | SNORA21 |
| ILF3 | ENSG00000101079 | NDRG3 |
| ILF3 | ENSG00000233155 | HMGA1P8 |
| ILF3 | ENSG00000186141 | POLR3C |
| ILF3 | ENSG00000169213 | RAB3B |
| ILF3 | ENSG00000163950 | SLBP |
| ILF3 | ENSG00000130707 | ASS1 |
| ILF3 | ENSG00000136521 | NDUFB5 |
| ILF3 | ENSG00000164403 | SHROOM1 |
| ILF3 | ENSG00000171155 | C1GALT1C1 |
| ILF3 | ENSG00000159335 | PTMS |
| ILF3 | ENSG00000088727 | KIF9 |
| ILF3 | ENSG00000267100 | ILF3-AS1 |
| ILF3 | ENSG00000116698 | SMG7 |
| ILF3 | ENSG00000140598 | EFTUD1 |
| ILF3 | ENSG00000184220 | CMSS1 |
| ILF3 | ENSG00000162517 | PEF1 |
| ILF3 | ENSG00000271253 | CTD-2320J21.1 |
| ILF3 | ENSG00000204516 | MICB |
| ILF3 | ENSG00000267454 | ZNF582-AS1 |
| ILF3 | ENSG00000012822 | CALCOCO1 |
| ILF3 | ENSG00000142945 | KIF2C |
| ILF3 | ENSG00000106605 | BLVRA |
| ILF3 | ENSG00000196415 | PRTN3 |
| ILF3 | ENSG00000158417 | EIF5B |
| ILF3 | ENSG00000164733 | CTSB |
| ILF3 | ENSG00000224936 | SUCLA2P1 |
| ILF3 | ENSG00000153339 | TRAPPC8 |
| ILF3 | ENSG00000138796 | HADH |
| ILF3 | ENSG00000199933 | RNY1P16 |
| ILF3 | ENSG00000181315 | ZNF322 |
| ILF3 | ENSG00000122515 | ZMIZ2 |
| ILF3 | ENSG00000114023 | FAM162A |
| ILF3 | ENSG00000263490 | RN7SL155P |
| ILF3 | ENSG00000164404 | GDF9 |
| ILF3 | ENSG00000066336 | SPI1 |
| ILF3 | ENSG00000125144 | MT1G |
| ILF3 | ENSG00000201811 | SNORA71 |
| ILF3 | ENSG00000275067 | MIR6825 |

| ILF3 | ENSG00000176714 | CCDC121 |
| --- | --- | --- |
| ILF3 | ENSG00000106608 | URGCP |
| ILF3 | ENSG00000115942 | ORC2 |
| ILF3 | ENSG00000253336 | RP11-468O2.1 |
| ILF3 | ENSG00000212994 | RPS26P6 |
| ILF3 | ENSG00000111665 | CDCA3 |
| ILF3 | ENSG00000130005 | GAMT |
| ILF3 | ENSG00000135722 | FBXL8 |
| ILF3 | ENSG00000259224 | SLC35G6 |
| ILF3 | ENSG00000199266 | SNORA60 |
| ILF3 | ENSG00000077782 | FGFR1 |
| ILF3 | ENSG00000140368 | PSTPIP1 |
| ILF3 | ENSG00000274367 | LA16c-380H5.6 |
| ILF3 | ENSG00000219608 | HIGD1AP16 |
| ILF3 | ENSG00000278986 | RP11-723J4.3 |
| ILF3 | ENSG00000207782 | MIR150 |
| ILF3 | ENSG00000060642 | PIGV |
| ILF3 | ENSG00000235062 | BCRP5 |
| ILF3 | ENSG00000156110 | ADK |
| ILF3 | ENSG00000215533 | LINC00189 |
| ILF3 | ENSG00000228126 | FALEC |
| ILF3 | ENSG00000132716 | DCAF8 |
| ILF3 | ENSG00000266750 | MIR4645 |
| ILF3 | ENSG00000145779 | TNFAIP8 |
| ILF3 | ENSG00000232179 | RP11-100G15.2 |
| ILF3 | ENSG00000196943 | NOP9 |
| ILF3 | ENSG00000095380 | NANS |
| ILF3 | ENSG00000223452 | HMGN2P18 |
| ILF3 | ENSG00000185065 | AC000068.5 |
| ILF3 | ENSG00000270682 | KB-1907C4.2 |
| ILF3 | ENSG00000178947 | LINC00086 |
| ILF3 | ENSG00000182973 | CNOT10 |
| ILF3 | ENSG00000212565 | SNORA68 |
| ILF3 | ENSG00000100764 | PSMC1 |
| ILF3 | ENSG00000230658 | KLHL7-AS1 |
| ILF3 | ENSG00000224966 | TBC1D3P6 |
| ILF3 | ENSG00000136286 | MYO1G |
| ILF3 | ENSG00000254480 | RP11-23F23.2 |
| ILF3 | ENSG00000212747 | FAM127C |
| ILF3 | ENSG00000170234 | PWWP2A |
| ILF3 | ENSG00000163154 | TNFAIP8L2 |
| ILF3 | ENSG00000274996 | CTB-58E17.2 |
| ILF3 | ENSG00000148090 | AUH |
| ILF3 | ENSG00000164776 | PHKG1 |
| ILF3 | ENSG00000100410 | PHF5A |
| ILF3 | ENSG00000114942 | EEF1B2 |
| ILF3 | ENSG00000101391 | CDK5RAP1 |
| ILF3 | ENSG00000116497 | S100PBP |
| ILF3 | ENSG00000153094 | BCL2L11 |
| ILF3 | ENSG00000142961 | MOB3C |
| ILF3 | ENSG00000001630 | CYP51A1 |

| ILF3 | ENSG00000067842 | ATP2B3 |
| --- | --- | --- |
| ILF3 | ENSG00000235314 | LINC00957 |
| ILF3 | ENSG00000231636 | AGBL5-AS1 |
| ILF3 | ENSG00000259706 | HSP90B2P |
| ILF3 | ENSG00000253384 | CTD-2547L16.3 |
| ILF3 | ENSG00000189266 | PNRC2 |
| ILF3 | ENSG00000100242 | SUN2 |
| ILF3 | ENSG00000228113 | AC003991.3 |
| ILF3 | ENSG00000267632 | RP11-400F19.18 |
| ILF3 | ENSG00000137509 | PRCP |
| ILF3 | ENSG00000103274 | NUBP1 |
| ILF3 | ENSG00000254690 | GS1-393G12.12 |
| ILF3 | ENSG00000272288 | RP11-140K17.3 |
| ILF3 | ENSG00000275857 | AC009133.21 |
| ILF3 | ENSG00000177096 | FAM109B |
| ILF3 | ENSG00000162723 | SLAMF9 |
| ILF3 | ENSG00000188785 | ZNF548 |
| ILF3 | ENSG00000103202 | NME4 |
| ILF3 | ENSG00000244052 | RPL5P24 |
| ILF3 | ENSG00000206760 | SNORA6 |
| ILF3 | ENSG00000238142 | RP11-108M9.4 |
| ILF3 | ENSG00000148158 | SNX30 |
| ILF3 | ENSG00000114353 | GNAI2 |
| ILF3 | ENSG00000103254 | FAM173A |
| ILF3 | ENSG00000145916 | RMND5B |
| ILF3 | ENSG00000106636 | YKT6 |
| ILF3 | ENSG00000164144 | ARFIP1 |
| ILF3 | ENSG00000162896 | PIGR |
| ILF3 | ENSG00000089818 | NECAP1 |
| ILF3 | ENSG00000133639 | BTG1 |
| ILF3 | ENSG00000246982 | RP1-179N16.6 |
| ILF3 | ENSG00000215005 | HSPD1P7 |
| ILF3 | ENSG00000170264 | FAM161A |
| ILF3 | ENSG00000131475 | VPS25 |
| ILF3 | ENSG00000132481 | TRIM47 |
| ILF3 | ENSG00000212296 | SNORD72 |
| ILF3 | ENSG00000255272 | RP11-646J21.6 |
| ILF3 | ENSG00000173511 | VEGFB |
| ILF3 | ENSG00000072134 | EPN2 |
| ILF3 | ENSG00000259755 | RP11-505E24.2 |
| ILF3 | ENSG00000212628 | RNA5SP241 |
| ILF3 | ENSG00000217555 | CKLF |
| ILF3 | ENSG00000116863 | ADPRHL2 |
| ILF3 | ENSG00000155980 | KIF5A |
| ILF3 | ENSG00000206931 | RNU6-1042P |
| ILF3 | ENSG00000151292 | CSNK1G3 |
| ILF3 | ENSG00000103044 | HAS3 |
| ILF3 | ENSG00000203795 | FAM24A |
| ILF3 | ENSG00000244510 | GS1-124K5.7 |
| ILF3 | ENSG00000245768 | RP11-410D17.2 |
| ILF3 | ENSG00000137364 | TPMT |

| ILF3 | ENSG00000268324 | LRRC2-AS1 |
| --- | --- | --- |
| ILF3 | ENSG00000112167 | SAYSD1 |
| ILF3 | ENSG00000181418 | DDN |
| ILF3 | ENSG00000236035 | RP11-90O23.1 |
| ILF3 | ENSG00000233862 | AC016907.3 |
| ILF3 | ENSG00000170515 | PA2G4 |
| ILF3 | ENSG00000205930 | C21orf62-AS1 |
| ILF3 | ENSG00000143751 | SDE2 |
| ILF3 | ENSG00000163116 | STPG2 |
| ILF3 | ENSG00000213753 | CENPBD1P1 |
| ILF3 | ENSG00000234807 | LINC01135 |
| ILF3 | ENSG00000117394 | SLC2A1 |
| ILF3 | ENSG00000201264 | SNORD73 |
| ILF3 | ENSG00000253133 | RP11-360L9.4 |
| ILF3 | ENSG00000176092 | AIM1L |
| ILF3 | ENSG00000232028 | AC007391.2 |
| ILF3 | ENSG00000261898 | RP11-314A20.5 |
| ILF3 | ENSG00000109689 | STIM2 |
| ILF3 | ENSG00000135976 | ANKRD36 |
| ILF3 | ENSG00000059691 | GATB |
| ILF3 | ENSG00000126878 | AIF1L |
| ILF3 | ENSG00000230565 | ZNF32-AS2 |
| ILF3 | ENSG00000267505 | CTC-296K1.3 |
| ILF3 | ENSG00000214842 | RAD51AP2 |
| ILF3 | ENSG00000204599 | TRIM39 |
| ILF3 | ENSG00000196653 | ZNF502 |
| ILF3 | ENSG00000130751 | NPAS1 |
| ILF3 | ENSG00000166596 | CFAP52 |
| ILF3 | ENSG00000258651 | RP11-545M17.1 |
| ILF3 | ENSG00000117385 | LEPRE1 |
| ILF3 | ENSG00000204237 | OXLD1 |
| ILF3 | ENSG00000076864 | RAP1GAP |
| ILF3 | ENSG00000103121 | CMC2 |
| ILF3 | ENSG00000085224 | ATRX |
| ILF3 | ENSG00000222808 | RNU4-47P |
| ILF3 | ENSG00000215883 | CYB5RL |
| ILF3 | ENSG00000253015 | RN7SKP64 |
| ILF3 | ENSG00000125505 | MBOAT7 |
| ILF3 | ENSG00000122966 | CIT |
| ILF3 | ENSG00000253291 | TRBV7-7 |
| ILF3 | ENSG00000251131 | CTD-2035E11.3 |
| ILF3 | ENSG00000236829 | Z97634.3 |
| ILF3 | ENSG00000225294 | OSTCP2 |
| ILF3 | ENSG00000116731 | PRDM2 |
| ILF3 | ENSG00000167011 | NAT16 |
| ILF3 | ENSG00000273214 | RP5-1039K5.18 |
| ILF3 | ENSG00000174326 | SLC16A11 |
| ILF3 | ENSG00000119943 | PYROXD2 |
| ILF3 | ENSG00000101452 | DHX35 |
| ILF3 | ENSG00000146955 | RAB19 |
| ILF3 | ENSG00000137947 | GTF2B |

| ILF3 ILF3 ILF3 | ENSG00000271855 ENSG00000111642 ENSG00000245112 | RP11-214N9.1 CHD4  SMARCA5- AS1 |
| --- | --- | --- |
| ILF3 | ENSG00000181036 | FCRL6 |
| ILF3 | ENSG00000224662 | ATP6V1G1P3 |
| ILF3 | ENSG00000133030 | MPRIP |
| ILF3 | ENSG00000224906 | RP1-102E24.9 |
| ILF3 | ENSG00000011258 | MBTD1 |
| ILF3 | ENSG00000261744 | RP11-21B21.4 |
| ILF3 | ENSG00000169902 | TPST1 |
| ILF3 | ENSG00000249215 | AC138517.4 |
| ILF3 | ENSG00000111790 | FGFR1OP2 |
| ILF3 | ENSG00000116489 | CAPZA1 |
| ILF3 | ENSG00000135185 | TMEM243 |
| ILF3 | ENSG00000242288 | RP11-464F9.1 |
| ILF3 | ENSG00000146007 | ZMAT2 |
| ILF3 | ENSG00000090565 | RAB11FIP3 |
| ILF3 | ENSG00000206560 | ANKRD28 |
| ILF3 | ENSG00000170100 | ZNF778 |
| ILF3 | ENSG00000115649 | CNPPD1 |
| ILF3 | ENSG00000102178 | UBL4A |
| ILF3 | ENSG00000169314 | C22orf15 |
| ILF3 | ENSG00000243777 | RP11-864G5.1 |
| ILF3 | ENSG00000088356 | PDRG1 |
| ILF3 | ENSG00000267348 | CTB-179K24.3 |
| ILF3 | ENSG00000233873 | RPL7P44 |
| ILF3 | ENSG00000175305 | CCNE2 |
| ILF3 | ENSG00000262165 | RP11-81A22.5 |
| ILF3 | ENSG00000176533 | GNG7 |
| ILF3 | ENSG00000258633 | RP11-857B24.1 |
| ILF3 | ENSG00000232768 | RP11-201O14.2 |
| ILF3 | ENSG00000230408 | AC007163.6 |
| ILF3 | ENSG00000275022 | MIR6753 |
| ILF3 | ENSG00000109016 | DHRS7B |
| ILF3 | ENSG00000165983 | PTER |
| ILF3 | ENSG00000196366 | C9orf163 |
| ILF3 | ENSG00000070371 | CLTCL1 |
| ILF3 | ENSG00000237781 | RP11-54A4.2 |
| ILF3 | ENSG00000116922 | C1orf109 |
| ILF3 | ENSG00000263072 | ZNF213-AS1 |
| ILF3 | ENSG00000271803 | RP1-63M2.5 |
| ILF3 | ENSG00000254844 | RP11-728F11.3 |
| ILF3 | ENSG00000240344 | PPIL3 |
| ILF3 | ENSG00000237360 | CHCHD4P2 |
| ILF3 | ENSG00000155666 | KDM8 |
| ILF3 | ENSG00000258289 | CHURC1 |
| ILF3 | ENSG00000232969 | AP001062.9 |
| ILF3 | ENSG00000168264 | IRF2BP2 |
| ILF3 | ENSG00000214593 | RP11-65N13.6 |
| ILF3 | ENSG00000270104 | RP11-245P10.6 |
| ILF3 | ENSG00000155189 | AGPAT5 |

| ILF3 | ENSG00000137288 | UQCC2 |
| --- | --- | --- |
| ILF3 | ENSG00000184900 | SUMO3 |
| ILF3 | ENSG00000236204 | LINC01376 |
| ILF3 | ENSG00000186301 | MST1P2 |
| ILF3 | ENSG00000242251 | RN7SL20P |
| ILF3 | ENSG00000230747 | AC021188.4 |
| ILF3 | ENSG00000211591 | MIR762 |
| ILF3 | ENSG00000221949 | LINC01465 |
| ILF3 | ENSG00000263826 | RP11-573D15.9 |
| ILF3 | ENSG00000265392 | MIR4252 |
| ILF3 | ENSG00000152422 | XRCC4 |
| ILF3 | ENSG00000198909 | MAP3K3 |
| ILF3 | ENSG00000164109 | MAD2L1 |
| ILF3 | ENSG00000111801 | BTN3A3 |
| ILF3 | ENSG00000083817 | ZNF416 |
| ILF3 | ENSG00000090316 | MAEA |
| ILF3 | ENSG00000238198 | RP11-31F15.2 |
| ILF3 | ENSG00000185522 | LMNTD2 |
| ILF3 | ENSG00000167992 | VWCE |
| ILF3 | ENSG00000138674 | SEC31A |
| ILF3 | ENSG00000110092 | CCND1 |
| ILF3 | ENSG00000276784 | JPX_2 |
| ILF3 | ENSG00000203791 | METTL10 |
| ILF3 | ENSG00000066651 | TRMT11 |
| ILF3 | ENSG00000198715 | C1orf85 |
| ILF3 | ENSG00000180035 | ZNF48 |
| ILF3 | ENSG00000241878 | PISD |
| ILF3 | ENSG00000069702 | TGFBR3 |
| ILF3 | ENSG00000203780 | FANK1 |
| ILF3 | ENSG00000133315 | MACROD1 |
| ILF3 | ENSG00000175764 | TTLL11 |
| ILF3 | ENSG00000052723 | SIKE1 |
| ILF3 | ENSG00000264004 | MIR4717 |
| ILF3 | ENSG00000205544 | TMEM256 |
| ILF3 | ENSG00000267582 | CTD-3252C9.2 |
| ILF3 | ENSG00000106346 | USP42 |
| ILF3 | ENSG00000200303 | RNU6-940P |
| ILF3 | ENSG00000114026 | OGG1 |
| ILF3 | ENSG00000223551 | TMSB4XP4 |
| ILF3 | ENSG00000163126 | ANKRD23 |
| ILF3 | ENSG00000275406 | RP11-125A15.3 |
| ILF3 | ENSG00000145293 | ENOPH1 |
| ILF3 | ENSG00000033627 | ATP6V0A1 |
| ILF3 | ENSG00000161533 | ACOX1 |
| ILF3 | ENSG00000101146 | RAE1 |
| ILF3 | ENSG00000119041 | GTF3C3 |
| ILF3 | ENSG00000115310 | RTN4 |
| ILF3 | ENSG00000232515 | LL22NC03- |
| ILF3 | ENSG00000168453 | 75A1.9  HR |
| ILF3 | ENSG00000168209 | DDIT4 |
| ILF3 | ENSG00000273015 | LINC00938 |

| ILF3 | ENSG00000199866 | Y_RNA |
| --- | --- | --- |
| ILF3 | ENSG00000106355 | LSM5 |
| ILF3 | ENSG00000186625 | KATNA1 |
| ILF3 | ENSG00000188313 | PLSCR1 |
| ILF3 | ENSG00000125449 | ARMC7 |
| ILF3 | ENSG00000207554 | MIR647 |
| ILF3 | ENSG00000227811 | FAM212B-AS1 |
| ILF3 | ENSG00000142669 | SH3BGRL3 |
| ILF3 | ENSG00000182575 | NXPH3 |
| ILF3 | ENSG00000256030 | CBX3P4 |
| ILF3 | ENSG00000166228 | PCBD1 |
| ILF3 | ENSG00000140682 | TGFB1I1 |
| ILF3 | ENSG00000275665 | CTB-58E17.9 |
| ILF3 | ENSG00000132507 | EIF5A |
| ILF3 | ENSG00000106086 | PLEKHA8 |
| ILF3 | ENSG00000236156 | CHCHD4P3 |
| ILF3 | ENSG00000260823 | RP11-249C24.10 |
| ILF3 | ENSG00000123405 | NFE2 |
| ILF3 | ENSG00000251017 | RP11-629B11.4 |
| ILF3 | ENSG00000276216 | RNVU1-14 |
| ILF3 | ENSG00000168872 | DDX19A |
| ILF3 | ENSG00000235445 | XXbac- |
| ILF3 | ENSG00000234694 | B476C20.14  RP1-92O14.3 |
| ILF3 | ENSG00000225903 | RP1-144F13.3 |
| ILF3 | ENSG00000162888 | C1orf147 |
| ILF3 | ENSG00000162769 | FLVCR1 |
| ILF3 | ENSG00000071242 | RPS6KA2 |
| ILF3 | ENSG00000173575 | CHD2 |
| ILF3 | ENSG00000242893 | RN7SL413P |
| ILF3 | ENSG00000234028 | AC062029.1 |
| ILF3 | ENSG00000185974 | GRK1 |
| ILF3 | ENSG00000200237 | SNORA70 |
| ILF3 | ENSG00000151623 | NR3C2 |
| ILF3 | ENSG00000272831 | RP11-792A8.4 |
| ILF3 | ENSG00000189316 | RP11-797H7.5 |
| ILF3 | ENSG00000185973 | TMLHE |
| ILF3 | ENSG00000189152 | GRAPL |
| ILF3 | ENSG00000237840 | FAM21FP |
| ILF3 | ENSG00000166965 | RCCD1 |
| ILF3 | ENSG00000133460 | SLC2A11 |
| ILF3 | ENSG00000280033 | CTD-2095E4.3 |
| ILF3 | ENSG00000252645 | RNU7-111P |
| ILF3 | ENSG00000095951 | HIVEP1 |
| ILF3 | ENSG00000206957 | Y_RNA |
| ILF3 | ENSG00000224579 | AC012314.19 |
| ILF3 | ENSG00000198821 | CD247 |
| ILF3 | ENSG00000167881 | SRP68 |
| ILF3 | ENSG00000242509 | RN7SL156P |
| ILF3 | ENSG00000185272 | RBM11 |
| ILF3 | ENSG00000104728 | ARHGEF10 |
| ILF3 | ENSG00000173559 | NABP1 |

| ILF3 | ENSG00000261697 | RP11-178L8.5 |
| --- | --- | --- |
| ILF3 | ENSG00000267598 | CTC-250I14.6 |
| ILF3 | ENSG00000229282 | RP1-40E16.2 |
| ILF3 | ENSG00000231128 | RP5-1073O3.2 |
| ILF3 | ENSG00000058673 | ZC3H11A |
| ILF3 | ENSG00000231464 | AC024937.4 |
| ILF3 | ENSG00000151364 | KCTD14 |
| ILF3 | ENSG00000075290 | WNT8B |
| ILF3 | ENSG00000101384 | JAG1 |
| ILF3 | ENSG00000252469 | RNU7-160P |
| ILF3 | ENSG00000255741 | RP11-757G1.5 |
| ILF3 | ENSG00000259797 | RP11-96D1.3 |
| ILF3 | ENSG00000136159 | NUDT15 |
| ILF3 | ENSG00000135870 | RC3H1 |
| ILF3 | ENSG00000182087 | TMEM259 |
| ILF3 | ENSG00000146776 | ATXN7L1 |
| ILF3 | ENSG00000167414 | GNG8 |
| ILF3 | ENSG00000248049 | UBA6-AS1 |
| ILF3 | ENSG00000260528 | FAM157C |
| ILF3 | ENSG00000066427 | ATXN3 |
| ILF3 | ENSG00000135390 | ATP5G2 |
| ILF3 | ENSG00000251562 | MALAT1 |
| ILF3 | ENSG00000080802 | CNOT4 |
| ILF3 | ENSG00000266994 | RP11-127I20.7 |
| ILF3 | ENSG00000224536 | RP11-134G8.7 |
| ILF3 | ENSG00000133858 | ZFC3H1 |
| ILF3 | ENSG00000130560 | UBAC1 |
| ILF3 | ENSG00000145685 | LHFPL2 |
| ILF3 | ENSG00000205138 | SDHAF1 |
| ILF3 | ENSG00000233538 | AC017104.2 |
| ILF3 | ENSG00000125249 | RAP2A |
| ILF3 | ENSG00000110693 | SOX6 |
| ILF3 | ENSG00000143107 | FNDC7 |
| ILF3 | ENSG00000269058 | CALR3 |
| ILF3 | ENSG00000264486 | CTD-2008P7.3 |
| ILF3 | ENSG00000175866 | BAIAP2 |
| ILF3 | ENSG00000107104 | KANK1 |
| ILF3 | ENSG00000143811 | PYCR2 |
| ILF3 | ENSG00000187837 | HIST1H1C |
| ILF3 | ENSG00000265451 | RP11-204L24.2 |
| ILF3 | ENSG00000267959 | MIR3188 |
| ILF3 | ENSG00000113387 | SUB1 |
| ILF3 | ENSG00000221926 | TRIM16 |
| ILF3 | ENSG00000113648 | H2AFY |
| ILF3 | ENSG00000221261 | MIR1208 |
| ILF3 | ENSG00000272703 | RP11-78A19.4 |
| ILF3 | ENSG00000092969 | TGFB2 |
| ILF3 | ENSG00000145777 | TSLP |
| ILF3 | ENSG00000260086 | RP11-42I10.1 |
| ILF3 | ENSG00000127922 | SHFM1 |
| ILF3 | ENSG00000156017 | C9orf41 |

| ILF3 | ENSG00000188021 | UBQLN2 |
| --- | --- | --- |
| ILF3 | ENSG00000144848 | ATG3 |
| ILF3 | ENSG00000186260 | MKL2 |
| ILF3 | ENSG00000177200 | CHD9 |
| ILF3 | ENSG00000179091 | CYC1 |
| ILF3 | ENSG00000163961 | RNF168 |
| ILF3 | ENSG00000189337 | KAZN |
| ILF3 | ENSG00000006062 | MAP3K14 |
| ILF3 | ENSG00000100441 | KHNYN |
| ILF3 | ENSG00000118900 | UBN1 |
| ILF3 | ENSG00000107290 | SETX |
| ILF3 | ENSG00000258472 | RP11-192H23.4 |
| ILF3 | ENSG00000064419 | TNPO3 |
| ILF3 | ENSG00000257529 | RPL36A- |
| ILF3 | ENSG00000119414 | HNRNPH2  PPP6C |
| ILF3 | ENSG00000184508 | HDDC3 |
| ILF3 | ENSG00000238003 | RP11-465N4.2 |
| ILF3 | ENSG00000160219 | GAB3 |
| ILF3 | ENSG00000261759 | RP11-626G11.3 |
| ILF3 | ENSG00000259182 | RP11-424I19.2 |
| ILF3 | ENSG00000239821 | RN7SL513P |
| ILF3 | ENSG00000150773 | PIH1D2 |
| ILF3 | ENSG00000064687 | ABCA7 |
| ILF3 | ENSG00000272456 | CTD-2342N23.3 |
| ILF3 | ENSG00000198496 | NBR2 |
| ILF3 | ENSG00000179057 | IGSF22 |
| ILF3 | ENSG00000167775 | CD320 |
| ILF3 | ENSG00000239382 | ALKBH6 |
| ILF3 | ENSG00000166900 | STX3 |
| ILF3 | ENSG00000111445 | RFC5 |
| ILF3 | ENSG00000264808 | MIR4523 |
| ILF3 | ENSG00000196576 | PLXNB2 |
| ILF3 | ENSG00000269846 | RP4-621N11.2 |
| ILF3 | ENSG00000133612 | AGAP3 |
| ILF3 | ENSG00000100109 | TFIP11 |
| ILF3 | ENSG00000147140 | NONO |
| ILF3 | ENSG00000204348 | DXO |
| ILF3 | ENSG00000185684 | EP400NL |
| ILF3 | ENSG00000185305 | ARL15 |
| ILF3 | ENSG00000267939 | CTD-2325M2.1 |
| ILF3 | ENSG00000267121 | CTD-2020K17.1 |
| ILF3 | ENSG00000249709 | ZNF564 |
| ILF3 | ENSG00000278756 | MIR6722 |
| ILF3 | ENSG00000256349 | CTD-3074O7.11 |
| ILF3 | ENSG00000267248 | CTD-2319I12.4 |
| ILF3 | ENSG00000074201 | CLNS1A |
| ILF3 | ENSG00000215915 | ATAD3C |
| ILF3 | ENSG00000129680 | MAP7D3 |
| ILF3 | ENSG00000146066 | HIGD2A |
| ILF3 | ENSG00000277720 | RP1-34B20.19 |
| ILF3 | ENSG00000090674 | MCOLN1 |

| ILF3 | ENSG00000124257 | NEURL2 |
| --- | --- | --- |
| ILF3 | ENSG00000069020 | MAST4 |
| ILF3 | ENSG00000134294 | SLC38A2 |
| ILF3 | ENSG00000227751 | RP1-20B21.4 |
| ILF3 | ENSG00000127720 | METTL25 |
| ILF3 | ENSG00000219027 | RPS3AP2 |
| ILF3 | ENSG00000144589 | STK11IP |
| ILF3 | ENSG00000149541 | B3GAT3 |
| ILF3 | ENSG00000205981 | DNAJC19 |
| ILF3 | ENSG00000166796 | LDHC |
| ILF3 | ENSG00000228949 | UGT1A12P |
| ILF3 | ENSG00000101558 | VAPA |
| ILF3 | ENSG00000197818 | SLC9A8 |
| ILF3 | ENSG00000071859 | FAM50A |
| ILF3 | ENSG00000260509 | RP11-271M24.2 |
| ILF3 | ENSG00000235194 | PPP1R3E |
| ILF3 | ENSG00000164022 | AIMP1 |
| ILF3 | ENSG00000138032 | PPM1B |
| ILF3 | ENSG00000261592 | RP11-178L8.3 |
| ILF3 | ENSG00000230989 | HSBP1 |
| ILF3 | ENSG00000125827 | TMX4 |
| ILF3 | ENSG00000165195 | PIGA |
| ILF3 | ENSG00000126259 | KIRREL2 |
| ILF3 | ENSG00000235021 | RP11-439E19.7 |
| ILF3 | ENSG00000232613 | AC007386.4 |
| ILF3 | ENSG00000213465 | ARL2 |
| ILF3 | ENSG00000133401 | PDZD2 |
| ILF3 | ENSG00000167705 | RILP |
| ILF3 | ENSG00000102595 | UGGT2 |
| ILF3 | ENSG00000137842 | TMEM62 |
| ILF3 | ENSG00000084093 | REST |
| ILF3 | ENSG00000227729 | RD3L |
| ILF3 | ENSG00000092847 | AGO1 |
| ILF3 | ENSG00000167220 | HDHD2 |
| ILF3 | ENSG00000213760 | ATP6V1G2 |
| ILF3 | ENSG00000265902 | MIR4696 |
| ILF3 | ENSG00000182670 | TTC3 |
| ILF3 | ENSG00000207083 | RNU6-22P |
| ILF3 | ENSG00000176974 | SHMT1 |
| ILF3 | ENSG00000185090 | MANEAL |
| ILF3 | ENSG00000170242 | USP47 |
| ILF3 | ENSG00000273734 | LLfos-48D6.2 |
| ILF3 | ENSG00000275620 | FLJ16779 |
| ILF3 | ENSG00000261079 | RP11-252A24.3 |
| ILF3 | ENSG00000267855 | NDUFA7 |
| ILF3 | ENSG00000165629 | ATP5C1 |
| ILF3 | ENSG00000082515 | MRPL22 |
| ILF3 | ENSG00000235499 | AC073046.25 |
| ILF3 | ENSG00000112773 | FAM46A |
| ILF3 | ENSG00000203667 | COX20 |
| ILF3 | ENSG00000106105 | GARS |

| ILF3 | ENSG00000009954 | BAZ1B |
| --- | --- | --- |
| ILF3 | ENSG00000185651 | UBE2L3 |
| ILF3 | ENSG00000114999 | TTL |
| ILF3 | ENSG00000261218 | RP11-960L18.1 |
| ILF3 | ENSG00000257286 | RP11-545P7.4 |
| ILF3 | ENSG00000247151 | CSTF3-AS1 |
| ILF3 | ENSG00000149527 | PLCH2 |
| ILF3 | ENSG00000124120 | TTPAL |
| ILF3 | ENSG00000274344 | ST7-AS2_1 |
| ILF3 | ENSG00000262728 | RP11-1000B6.8 |
| ILF3 | ENSG00000189325 | C6orf222 |
| ILF3 | ENSG00000147224 | PRPS1 |
| ILF3 | ENSG00000272145 | NFYC-AS1 |
| ILF3 | ENSG00000145725 | PPIP5K2 |
| ILF3 | ENSG00000272296 | SNORD96A |
| ILF3 | ENSG00000178741 | COX5A |
| ILF3 | ENSG00000162623 | TYW3 |
| ILF3 | ENSG00000168481 | LGI3 |
| ILF3 | ENSG00000105376 | ICAM5 |
| ILF3 | ENSG00000269859 | CTD-2537I9.16 |
| ILF3 | ENSG00000280426 | RP11-837J7.3 |
| ILF3 | ENSG00000230160 | AC004854.5 |
| ILF3 | ENSG00000261559 | FSCN1P1 |
| ILF3 | ENSG00000101911 | PRPS2 |
| ILF3 | ENSG00000249417 | RP11-438D8.2 |
| ILF3 | ENSG00000272663 | RP11-191L17.1 |
| ILF3 | ENSG00000255920 | CCND2-AS2 |
| ILF3 | ENSG00000143621 | ILF2 |
| ILF3 | ENSG00000206979 | SNORD61 |
| ILF3 | ENSG00000213965 | NUDT19 |
| ILF3 | ENSG00000103184 | SEC14L5 |
| ILF3 | ENSG00000105321 | CCDC9 |
| ILF3 | ENSG00000162600 | OMA1 |
| ILF3 | ENSG00000237719 | RP1-179N16.3 |
| ILF3 | ENSG00000116161 | CACYBP |
| ILF3 | ENSG00000227507 | LTB |
| ILF3 | ENSG00000158715 | SLC45A3 |
| ILF3 | ENSG00000280126 | AC069542.1 |
| ILF3 | ENSG00000228312 | GAPDHP45 |
| ILF3 | ENSG00000132680 | KIAA0907 |
| ILF3 | ENSG00000226239 | RP1-310O13.7 |
| ILF3 | ENSG00000276598 | MIR6893 |
| ILF3 | ENSG00000259466 | NPM1P47 |
| ILF3 | ENSG00000229124 | VIM-AS1 |
| ILF3 | ENSG00000167604 | NFKBID |
| ILF3 | ENSG00000105576 | TNPO2 |
| ILF3 | ENSG00000211563 | MIR3065 |
| ILF3 | ENSG00000145908 | ZNF300 |
| ILF3 | ENSG00000198482 | ZNF808 |
| ILF3 | ENSG00000100564 | PIGH |
| ILF3 | ENSG00000055211 | GINM1 |

| ILF3 | ENSG00000071082 | RPL31 |
| --- | --- | --- |
| ILF3 | ENSG00000131899 | LLGL1 |
| ILF3 | ENSG00000256751 | PLBD1-AS1 |
| ILF3 | ENSG00000177666 | PNPLA2 |
| ILF3 | ENSG00000099968 | BCL2L13 |
| ILF3 | ENSG00000116809 | ZBTB17 |
| ILF3 | ENSG00000171735 | CAMTA1 |
| ILF3 | ENSG00000248916 | NUP210P3 |
| ILF3 | ENSG00000197826 | C4orf22 |
| ILF3 | ENSG00000129474 | AJUBA |
| ILF3 | ENSG00000101363 | MANBAL |
| ILF3 | ENSG00000087299 | L2HGDH |
| ILF3 | ENSG00000237436 | RP11-312B8.1 |
| ILF3 | ENSG00000117528 | ABCD3 |
| ILF3 | ENSG00000234707 | RP11-745C15.2 |
| ILF3 | ENSG00000124635 | HIST1H2BJ |
| ILF3 | ENSG00000167005 | NUDT21 |
| ILF3 | ENSG00000270362 | HMGN3-AS1 |
| ILF3 | ENSG00000169738 | DCXR |
| ILF3 | ENSG00000267512 | CTC-250I14.3 |
| ILF3 | ENSG00000161010 | C5orf45 |
| ILF3 | ENSG00000167702 | KIFC2 |
| ILF3 | ENSG00000164621 | SMAD5-AS1 |
| ILF3 | ENSG00000170855 | TRIAP1 |
| ILF3 | ENSG00000237493 | RP11-603J24.7 |
| ILF3 | ENSG00000110200 | ANAPC15 |
| ILF3 | ENSG00000236806 | RPL7AP15 |
| ILF3 | ENSG00000176244 | ACBD7 |
| ILF3 | ENSG00000123983 | ACSL3 |
| ILF3 | ENSG00000151348 | EXT2 |
| ILF3 | ENSG00000262179 | RP1-302G2.5 |
| ILF3 | ENSG00000123374 | CDK2 |
| ILF3 | ENSG00000275740 | RP11-449H3.3 |
| ILF3 | ENSG00000271040 | RP5-933K21.3 |
| ILF3 | ENSG00000225357 | RPF2P1 |
| ILF3 | ENSG00000257075 | RPEP6 |
| ILF3 | ENSG00000278233 | RNA5-8S5 |
| ILF3 | ENSG00000105699 | LSR |
| ILF3 | ENSG00000275457 | RP1-198K11.5 |
| ILF3 | ENSG00000100852 | ARHGAP5 |
| ILF3 | ENSG00000213664 | RPS16P8 |
| ILF3 | ENSG00000120647 | CCDC77 |
| ILF3 | ENSG00000151657 | KIN |
| ILF3 | ENSG00000196998 | WDR45 |
| ILF3 | ENSG00000141854 | MIR1199 |
| ILF3 | ENSG00000031691 | CENPQ |
| ILF3 | ENSG00000198301 | SDAD1 |
| ILF3 | ENSG00000149054 | ZNF215 |
| ILF3 | ENSG00000204590 | GNL1 |
| ILF3 | ENSG00000231907 | GAPDHP37 |
| ILF3 | ENSG00000140577 | CRTC3 |

| ILF3 | ENSG00000162542 | TMCO4 |
| --- | --- | --- |
| ILF3 | ENSG00000200831 | SNORD36B |
| ILF3 | ENSG00000130643 | CALY |
| ILF3 | ENSG00000253540 | FAM86HP |
| ILF3 | ENSG00000110844 | PRPF40B |
| ILF3 | ENSG00000196361 | ELAVL3 |
| ILF3 | ENSG00000136783 | NIPSNAP3A |
| ILF3 | ENSG00000070081 | NUCB2 |
| ILF3 | ENSG00000267589 | CTC-232P5.4 |
| ILF3 | ENSG00000106261 | ZKSCAN1 |
| ILF3 | ENSG00000163428 | LRRC58 |
| ILF3 | ENSG00000164897 | TMUB1 |
| ILF3 | ENSG00000139684 | ESD |
| ILF3 | ENSG00000267549 | AC006116.17 |
| ILF3 | ENSG00000007968 | E2F2 |
| ILF3 | ENSG00000168795 | ZBTB5 |
| ILF3 | ENSG00000137876 | RSL24D1 |
| ILF3 | ENSG00000254452 | RP11-867G23.4 |
| ILF3 | ENSG00000250299 | MRPS31P4 |
| ILF3 | ENSG00000115902 | SLC1A4 |
| ILF3 | ENSG00000165409 | TSHR |
| ILF3 | ENSG00000108219 | TSPAN14 |
| ILF3 | ENSG00000172828 | CES3 |
| ILF3 | ENSG00000061273 | HDAC7 |
| ILF3 | ENSG00000273901 | CTD-2619J13.27 |
| ILF3 | ENSG00000260612 | RP11-432I5.4 |
| ILF3 | ENSG00000254855 | RP11-867G23.1 |
| ILF3 | ENSG00000221039 | MIR1286 |
| ILF3 | ENSG00000118096 | IFT46 |
| ILF3 | ENSG00000100429 | HDAC10 |
| ILF3 | ENSG00000212330 | RNU6-244P |
| ILF3 | ENSG00000222800 | RNU2-62P |
| ILF3 | ENSG00000173627 | APOBEC4 |
| ILF3 | ENSG00000127445 | PIN1 |
| ILF3 | ENSG00000103260 | METRN |
| ILF3 | ENSG00000143393 | PI4KB |
| ILF3 | ENSG00000277918 | U1 |
| ILF3 | ENSG00000234183 | AC004854.4 |
| ILF3 | ENSG00000136463 | TACO1 |
| ILF3 | ENSG00000120733 | KDM3B |
| ILF3 | ENSG00000173436 | MINOS1 |
| ILF3 | ENSG00000178252 | WDR6 |
| ILF3 | ENSG00000120802 | TMPO |
| ILF3 | ENSG00000176692 | FOXC2 |
| ILF3 | ENSG00000105669 | COPE |
| ILF3 | ENSG00000169242 | EFNA1 |
| ILF3 | ENSG00000214783 | POLR2J4 |
| ILF3 | ENSG00000185101 | ANO9 |
| ILF3 | ENSG00000252540 | RNU6-919P |
| ILF3 | ENSG00000162976 | PQLC3 |
| ILF3 | ENSG00000142694 | EVA1B |

| ILF3 | ENSG00000197409 | HIST1H3D |
| --- | --- | --- |
| ILF3 | ENSG00000267033 | CTD-2562J15.4 |
| ILF3 | ENSG00000147687 | TATDN1 |
| ILF3 | ENSG00000259215 | RP11-253M7.4 |
| ILF3 | ENSG00000228109 | MFI2-AS1 |
| ILF3 | ENSG00000135164 | DMTF1 |
| ILF3 | ENSG00000257384 | RP11-644F5.12 |
| ILF3 | ENSG00000267475 | CTD-2538C1.2 |
| ILF3 | ENSG00000178078 | STAP2 |
| ILF3 | ENSG00000073670 | ADAM11 |
| ILF3 | ENSG00000260492 | CISTR |
| ILF3 | ENSG00000110717 | NDUFS8 |
| ILF3 | ENSG00000162928 | PEX13 |
| ILF3 | ENSG00000224164 | RP3-369A17.4 |
| ILF3 | ENSG00000269890 | RP5-1139B12.2 |
| ILF3 | ENSG00000228700 | RP11-155G14.1 |
| ILF3 | ENSG00000167861 | HID1 |
| ILF3 | ENSG00000183891 | TTC32 |
| ILF3 | ENSG00000133641 | C12orf29 |
| ILF3 | ENSG00000077152 | UBE2T |
| ILF3 | ENSG00000182327 | GLTPD2 |
| ILF3 | ENSG00000136874 | STX17 |
| ILF3 | ENSG00000100412 | ACO2 |
| ILF3 | ENSG00000267221 | CTD- |
| ILF3 | ENSG00000230148 | 2132N18.2 HOXB-AS1 |
| ILF3 | ENSG00000134107 | BHLHE40 |
| ILF3 | ENSG00000197070 | ARRDC1 |
| ILF3 | ENSG00000234869 | RP3-439F8.1 |
| ILF3 | ENSG00000261204 | AC004449.6 |
| ILF3 | ENSG00000143374 | TARS2 |
| ILF3 | ENSG00000198646 | NCOA6 |
| ILF3 | ENSG00000100997 | ABHD12 |
| ILF3 | ENSG00000232788 | AC078883.3 |
| ILF3 | ENSG00000132305 | IMMT |
| ILF3 | ENSG00000251018 | HMMR-AS1 |
| ILF3 | ENSG00000196700 | ZNF512B |
| ILF3 | ENSG00000233060 | AC016700.2 |
| ILF3 | ENSG00000259782 | CTD-2270L9.2 |
| ILF3 | ENSG00000256944 | RP11-881M11.1 |
| ILF3 | ENSG00000107738 | C10orf54 |
| ILF3 | ENSG00000247516 | MIR4458HG |
| ILF3 | ENSG00000279809 | AC005538.3 |
| ILF3 | ENSG00000200378 | RNU5B-4P |
| ILF3 | ENSG00000130787 | HIP1R |
| ILF3 | ENSG00000128268 | MGAT3 |
| ILF3 | ENSG00000125826 | RBCK1 |
| ILF3 | ENSG00000122085 | MTERF4 |
| ILF3 | ENSG00000023608 | SNAPC1 |
| ILF3 | ENSG00000264293 | RN7SL657P |
| ILF3 | ENSG00000112053 | SLC26A8 |
| ILF3 | ENSG00000197885 | NKIRAS1 |

| ILF3 | ENSG00000167863 | ATP5H |
| --- | --- | --- |
| ILF3 | ENSG00000113761 | ZNF346 |
| ILF3 | ENSG00000142856 | ITGB3BP |
| ILF3 | ENSG00000184381 | PLA2G6 |
| ILF3 | ENSG00000140987 | ZSCAN32 |
| ILF3 | ENSG00000250719 | RP11-322N21.2 |
| ILF3 | ENSG00000148248 | SURF4 |
| ILF3 | ENSG00000143458 | GABPB2 |
| ILF3 | ENSG00000156709 | AIFM1 |
| ILF3 | ENSG00000091592 | NLRP1 |
| ILF3 | ENSG00000258315 | C17orf49 |
| ILF3 | ENSG00000216657 | GLRX3P2 |
| ILF3 | ENSG00000007264 | MATK |
| ILF3 | ENSG00000100884 | CPNE6 |
| ILF3 | ENSG00000263955 | RN7SL850P |
| ILF3 | ENSG00000236723 | RP5-1024G6.2 |
| ILF3 | ENSG00000089154 | GCN1L1 |
| ILF3 | ENSG00000137804 | NUSAP1 |
| ILF3 | ENSG00000118960 | HS1BP3 |
| ILF3 | ENSG00000272821 | CTA-384D8.36 |
| ILF3 | ENSG00000255108 | AP006621.8 |
| ILF3 | ENSG00000248101 | AC002116.8 |
| ILF3 | ENSG00000197093 | GAL3ST4 |
| ILF3 | ENSG00000153815 | CMIP |
| ILF3 | ENSG00000196890 | HIST3H2BB |
| ILF3 | ENSG00000136100 | VPS36 |
| ILF3 | ENSG00000160688 | FLAD1 |
| ILF3 | ENSG00000233762 | AC007969.5 |
| ILF3 | ENSG00000237118 | CYP2F2P |
| ILF3 | ENSG00000148358 | GPR107 |
| ILF3 | ENSG00000138744 | NAAA |
| ILF3 | ENSG00000259307 | PLCB2-AS1 |
| ILF3 | ENSG00000164111 | ANXA5 |
| ILF3 | ENSG00000236936 | RP3-329E20.2 |
| ILF3 | ENSG00000241180 | RP11-54O7.2 |
| ILF3 | ENSG00000274124 | RP11-474C8.8 |
| ILF3 | ENSG00000184990 | SIVA1 |
| ILF3 | ENSG00000257921 | RP11-571M6.15 |
| ILF3 | ENSG00000155506 | LARP1 |
| ILF3 | ENSG00000131401 | NAPSB |
| ILF3 | ENSG00000200530 | SNORD35B |
| ILF3 | ENSG00000107077 | KDM4C |
| ILF3 | ENSG00000169490 | TM2D2 |
| ILF3 | ENSG00000103227 | LMF1 |
| ILF3 | ENSG00000171492 | LRRC8D |
| ILF3 | ENSG00000274306 | MIR5088 |
| ILF3 | ENSG00000267144 | AC067968.3 |
| ILF3 | ENSG00000122705 | CLTA |
| ILF3 | ENSG00000144677 | CTDSPL |
| ILF3 | ENSG00000263489 | CTC-264K15.6 |
| ILF3 | ENSG00000231346 | LINC01160 |

| ILF3 | ENSG00000254838 | GVINP1 |
| --- | --- | --- |
| ILF3 | ENSG00000271899 | MIR4466 |
| ILF3 | ENSG00000171793 | CTPS1 |
| ILF3 | ENSG00000156469 | MTERF3 |
| ILF3 | ENSG00000161955 | TNFSF13 |
| ILF3 | ENSG00000073584 | SMARCE1 |
| ILF3 | ENSG00000185250 | PPIL6 |
| ILF3 | ENSG00000237338 | FTCD-AS1 |
| ILF3 | ENSG00000269303 | CTD-2527I21.7 |
| ILF3 | ENSG00000063169 | GLTSCR1 |
| ILF3 | ENSG00000088053 | GP6 |
| ILF3 | ENSG00000083896 | YTHDC1 |
| ILF3 | ENSG00000198520 | C1orf228 |
| ILF3 | ENSG00000246863 | RP11-325N19.3 |
| ILF3 | ENSG00000108641 | B9D1 |
| ILF3 | ENSG00000154222 | CC2D1B |
| ILF3 | ENSG00000247925 | RP3-510L9.1 |
| ILF3 | ENSG00000126790 | L3HYPDH |
| ILF3 | ENSG00000273464 | RP11-313P22.1 |
| ILF3 | ENSG00000270604 | HCG17 |
| ILF3 | ENSG00000129250 | KIF1C |
| ILF3 | ENSG00000255568 | BRWD1-AS2 |
| ILF3 | ENSG00000263465 | SRSF8 |
| ILF3 | ENSG00000046647 | GEMIN8 |
| ILF3 | ENSG00000248893 | FAM138E |
| ILF3 | ENSG00000240859 | AC093627.10 |
| ILF3 | ENSG00000165487 | MICU2 |
| ILF3 | ENSG00000230487 | PSMG3-AS1 |
| ILF3 | ENSG00000197062 | ZSCAN26 |
| ILF3 | ENSG00000196177 | ACADSB |
| ILF3 | ENSG00000153071 | DAB2 |
| ILF3 | ENSG00000143727 | ACP1 |
| ILF3 | ENSG00000157184 | CPT2 |
| ILF3 | ENSG00000215478 | CES5AP1 |
| ILF3 | ENSG00000271616 | RP11-578F21.13 |
| ILF3 | ENSG00000131471 | AOC3 |
| ILF3 | ENSG00000167487 | KLHL26 |
| ILF3 | ENSG00000242928 | RN7SL868P |
| ILF3 | ENSG00000105402 | NAPA |
| ILF3 | ENSG00000175782 | SLC35E3 |
| ILF3 | ENSG00000244509 | APOBEC3C |
| ILF3 | ENSG00000196652 | ZKSCAN5 |
| ILF3 | ENSG00000196549 | MME |
| ILF3 | ENSG00000267275 | CTD-2562J15.6 |
| ILF3 | ENSG00000167945 | PRR25 |
| ILF3 | ENSG00000235494 | RP11-498P14.4 |
| ILF3 | ENSG00000164323 | CFAP97 |
| ILF3 | ENSG00000152078 | TMEM56 |
| ILF3 | ENSG00000135931 | ARMC9 |
| ILF3 | ENSG00000072135 | PTPN18 |
| ILF3 | ENSG00000270666 | XXbac- BPGBPG34I8.1 |

| ILF3 | ENSG00000267344 | CTB-39G8.3 |
| --- | --- | --- |
| ILF3 | ENSG00000168032 | ENTPD3 |
| ILF3 | ENSG00000263179 | HNRNPCP4 |
| ILF3 | ENSG00000010318 | PHF7 |
| ILF3 | ENSG00000276712 | MIR7111 |
| ILF3 | ENSG00000175920 | DOK7 |
| ILF3 | ENSG00000135617 | PRADC1 |
| ILF3 | ENSG00000122042 | UBL3 |
| ILF3 | ENSG00000172250 | SERHL |
| ILF3 | ENSG00000226643 | RP11-358H9.1 |
| ILF3 | ENSG00000204475 | NCR3 |
| ILF3 | ENSG00000112029 | FBXO5 |
| ILF3 | ENSG00000063180 | CA11 |
| ILF3 | ENSG00000082898 | XPO1 |
| ILF3 | ENSG00000274086 | FMR1-AS1_2 |
| ILF3 | ENSG00000267769 | CTB-50L17.9 |
| ILF3 | ENSG00000136371 | MTHFS |
| ILF3 | ENSG00000117425 | PTCH2 |
| ILF3 | ENSG00000125967 | NECAB3 |
| ILF3 | ENSG00000132964 | CDK8 |
| ILF3 | ENSG00000114115 | RBP1 |
| ILF3 | ENSG00000241839 | PLEKHO2 |
| ILF3 | ENSG00000255289 | RP11-91I20.1 |
| ILF3 | ENSG00000272841 | RP3-428L16.2 |
| ILF3 | ENSG00000074317 | SNCB |
| ILF3 | ENSG00000276166 | CTD-2600O9.2 |
| ILF3 | ENSG00000258137 | RP11-753H16.3 |
| ILF3 | ENSG00000114770 | ABCC5 |
| ILF3 | ENSG00000100528 | CNIH1 |
| ILF3 | ENSG00000127995 | CASD1 |
| ILF3 | ENSG00000153214 | TMEM87B |
| ILF3 | ENSG00000241666 | RP3-455J7.4 |
| ILF3 | ENSG00000227105 | PARP1P1 |
| ILF3 | ENSG00000221238 | MIR1285-2 |
| ILF3 | ENSG00000274849 | RP11-49I11.4 |
| ILF3 | ENSG00000198380 | GFPT1 |
| ILF3 | ENSG00000254469 | RP11-849H4.2 |
| ILF3 | ENSG00000128191 | DGCR8 |
| ILF3 | ENSG00000242439 | CTD-2349P21.1 |
| ILF3 | ENSG00000263002 | ZNF234 |
| ILF3 | ENSG00000076944 | STXBP2 |
| ILF3 | ENSG00000225733 | FGD5-AS1 |
| ILF3 | ENSG00000233427 | RP1-212P9.3 |
| ILF3 | ENSG00000252236 | SNORA26 |
| ILF3 | ENSG00000065029 | ZNF76 |
| ILF3 | ENSG00000202336 | RNU6-359P |
| ILF3 | ENSG00000204438 | GPANK1 |
| ILF3 | ENSG00000207327 | RNU6-883P |
| ILF3 | ENSG00000153046 | CDYL |
| ILF3 | ENSG00000250751 | RP11-613C6.2 |
| ILF3 | ENSG00000267334 | CTD-2534I21.8 |

| ILF3 | ENSG00000101460 | MAP1LC3A |
| --- | --- | --- |
| ILF3 | ENSG00000185917 | SETD4 |
| ILF3 | ENSG00000167088 | SNRPD1 |
| ILF3 | ENSG00000178971 | CTC1 |
| ILF3 | ENSG00000163956 | LRPAP1 |
| ILF3 | ENSG00000105662 | CRTC1 |
| ILF3 | ENSG00000114480 | GBE1 |
| ILF3 | ENSG00000162433 | AK4 |
| ILF3 | ENSG00000170633 | RNF34 |
| ILF3 | ENSG00000001167 | NFYA |
| ILF3 | ENSG00000143344 | RGL1 |
| ILF3 | ENSG00000261717 | RP11-77K12.1 |
| ILF3 | ENSG00000118482 | PHF3 |
| ILF3 | ENSG00000258722 | CKAP2P1 |
| ILF3 | ENSG00000198853 | RUSC2 |
| ILF3 | ENSG00000254821 | RP3-400B16.4 |
| ILF3 | ENSG00000276058 | STMN1P1 |
| ILF3 | ENSG00000023572 | GLRX2 |
| ILF3 | ENSG00000269815 | CTD-2278I10.4 |
| ILF3 | ENSG00000255468 | RP11-867G23.8 |
| ILF3 | ENSG00000168890 | TMEM150A |
| ILF3 | ENSG00000103356 | EARS2 |
| ILF3 | ENSG00000163608 | C3orf17 |
| ILF3 | ENSG00000126759 | CFP |
| ILF3 | ENSG00000198830 | HMGN2 |
| ILF3 | ENSG00000138768 | USO1 |
| ILF3 | ENSG00000269514 | RP11-370I10.12 |
| ILF3 | ENSG00000000457 | SCYL3 |
| ILF3 | ENSG00000267405 | CTC-296K1.4 |
| ILF3 | ENSG00000165898 | ISCA2 |
| ILF3 | ENSG00000118162 | KPTN |
| ILF3 | ENSG00000198624 | CCDC69 |
| ILF3 | ENSG00000244314 | RN7SL36P |
| ILF3 | ENSG00000105664 | COMP |
| ILF3 | ENSG00000114098 | ARMC8 |
| ILF3 | ENSG00000096654 | ZNF184 |
| ILF3 | ENSG00000116679 | IVNS1ABP |
| ILF3 | ENSG00000271662 | RP11-141C7.3 |
| ILF3 | ENSG00000184281 | TSSC4 |
| ILF3 | ENSG00000196110 | ZNF699 |
| ILF3 | ENSG00000261680 | RP11-146F11.3 |
| ILF3 | ENSG00000181163 | NPM1 |
| ILF3 | ENSG00000228144 | RP11-745O10.4 |
| ILF3 | ENSG00000180921 | FAM83H |
| ILF3 | ENSG00000278156 | TSC22D1-AS1 |
| ILF3 | ENSG00000111203 | ITFG2 |
| ILF3 | ENSG00000170374 | SP7 |
| ILF3 | ENSG00000143970 | ASXL2 |
| ILF3 | ENSG00000222895 | RNU6-1133P |
| ILF3 | ENSG00000226572 | SNORD57 |
| ILF3 | ENSG00000232022 | FAAHP1 |

| ILF3 | ENSG00000273841 | AK6 |
| --- | --- | --- |
| ILF3 | ENSG00000214046 | SMIM7 |
| ILF3 | ENSG00000232756 | RP5-1185I7.1 |
| ILF3 | ENSG00000067601 | PMS2P4 |
| ILF3 | ENSG00000272989 | RP13-616I3.1 |
| ILF3 | ENSG00000138430 | OLA1 |
| ILF3 | ENSG00000259677 | RP11-493E3.1 |
| ILF3 | ENSG00000178301 | AQP11 |
| ILF3 | ENSG00000233493 | TMEM238 |
| ILF3 | ENSG00000188078 | RP5-1119A7.11 |
| ILF3 | ENSG00000260651 | AF213884.2 |
| ILF3 | ENSG00000258559 | AC005519.4 |
| ILF3 | ENSG00000273585 | RP11-14C10.5 |
| ILF3 | ENSG00000146267 | FAXC |
| ILF3 | ENSG00000248516 | RP11-265O12.1 |
| ILF3 | ENSG00000260776 | RP11-114H24.2 |
| ILF3 | ENSG00000241307 | RP11-674E16.1 |
| ILF3 | ENSG00000259611 | CTD-2544M6.2 |
| ILF3 | ENSG00000129473 | BCL2L2 |
| ILF3 | ENSG00000138592 | USP8 |
| ILF3 | ENSG00000251298 | RP11-425A23.1 |
| ILF3 | ENSG00000224316 | RP11-479O9.2 |
| ILF3 | ENSG00000107862 | GBF1 |
| ILF3 | ENSG00000230513 | THAP7-AS1 |
| ILF3 | ENSG00000226756 | AC007365.3 |
| ILF3 | ENSG00000154217 | PITPNC1 |
| ILF3 | ENSG00000231414 | AC016700.6 |
| ILF3 | ENSG00000170088 | TMEM192 |
| ILF3 | ENSG00000112406 | HECA |
| ILF3 | ENSG00000257551 | HLX-AS1 |
| ILF3 | ENSG00000101849 | TBL1X |
| ILF3 | ENSG00000262202 | SNORD3D |
| ILF3 | ENSG00000232803 | SLCO4A1-AS1 |
| ILF3 | ENSG00000146842 | TMEM209 |
| ILF3 | ENSG00000134851 | TMEM165 |
| ILF3 | ENSG00000196981 | WDR5B |
| ILF3 | ENSG00000125450 | NUP85 |
| ILF3 | ENSG00000169813 | HNRNPF |
| ILF3 | ENSG00000103490 | PYCARD |
| ILF3 | ENSG00000107874 | CUEDC2 |
| ILF3 | ENSG00000267815 | CTB-191K22.5 |
| ILF3 | ENSG00000122729 | ACO1 |
| ILF3 | ENSG00000187239 | FNBP1 |
| ILF3 | ENSG00000131183 | SLC34A1 |
| ILF3 | ENSG00000138138 | ATAD1 |
| ILF3 | ENSG00000119812 | FAM98A |
| ILF3 | ENSG00000174871 | CNIH2 |
| ILF3 | ENSG00000006327 | TNFRSF12A |
| ILF3 | ENSG00000178980 | SEPW1 |
| ILF3 | ENSG00000227803 | RP11-239H6.2 |
| ILF3 | ENSG00000235629 | AC090952.5 |

| ILF3 | ENSG00000177045 | SIX5 |
| --- | --- | --- |
| ILF3 | ENSG00000219932 | RPL12P8 |
| ILF3 | ENSG00000270673 | YTHDF3-AS1 |
| ILF3 | ENSG00000275803 | Metazoa_SRP |
| ILF3 | ENSG00000261886 | RP11-63A1.1 |
| ILF3 | ENSG00000223774 | RP11-307B6.3 |
| ILF3 | ENSG00000124659 | TBCC |
| ILF3 | ENSG00000172977 | KAT5 |
| ILF3 | ENSG00000115107 | STEAP3 |
| ILF3 | ENSG00000134278 | SPIRE1 |
| ILF3 | ENSG00000112599 | GUCA1B |
| ILF3 | ENSG00000168899 | VAMP5 |
| ILF3 | ENSG00000184068 | RP5-821D11.7 |
| ILF3 | ENSG00000144445 | KANSL1L |
| ILF3 | ENSG00000146247 | PHIP |
| ILF3 | ENSG00000099901 | RANBP1 |
| ILF3 | ENSG00000090621 | PABPC4 |
| ILF3 | ENSG00000152102 | FAM168B |
| ILF3 | ENSG00000198513 | ATL1 |
| ILF3 | ENSG00000264019 | RP11-6N17.6 |
| ILF3 | ENSG00000213413 | PVRIG |
| ILF3 | ENSG00000167588 | GPD1 |
| ILF3 | ENSG00000264548 | RP13-516M14.2 |
| ILF3 | ENSG00000188833 | ENTPD8 |
| ILF3 | ENSG00000204316 | MRPL38 |
| ILF3 | ENSG00000261351 | CTD-3185P2.1 |
| ILF3 | ENSG00000138029 | HADHB |
| ILF3 | ENSG00000136856 | SLC2A8 |
| ILF3 | ENSG00000261312 | AC002550.5 |
| ILF3 | ENSG00000275640 | MIR6793 |
| ILF3 | ENSG00000227702 | LINC00111 |
| ILF3 | ENSG00000175334 | BANF1 |
| ILF3 | ENSG00000272341 | RP1-151F17.2 |
| ILF3 | ENSG00000142655 | PEX14 |
| ILF3 | ENSG00000262246 | CORO7 |
| ILF3 | ENSG00000151693 | ASAP2 |
| ILF3 | ENSG00000207996 | MIR301A |
| ILF3 | ENSG00000157873 | TNFRSF14 |
| ILF3 | ENSG00000163874 | ZC3H12A |
| ILF3 | ENSG00000130545 | CRB3 |
| ILF3 | ENSG00000141564 | RPTOR |
| ILF3 | ENSG00000267345 | CTD-2293H3.2 |
| ILF3 | ENSG00000197363 | ZNF517 |
| ILF3 | ENSG00000113161 | HMGCR |
| ILF3 | ENSG00000271918 | CTD- |
| ILF3 | ENSG00000096080 | 2287O16.5  MRPS18A |
| ILF3 | ENSG00000266405 | CBX3P2 |
| ILF3 | ENSG00000145198 | VWA5B2 |
| ILF3 | ENSG00000148229 | POLE3 |
| ILF3 | ENSG00000173020 | ADRBK1 |
| ILF3 | ENSG00000166224 | SGPL1 |

| ILF3 | ENSG00000169220 | RGS14 |
| --- | --- | --- |
| ILF3 | ENSG00000155330 | C16orf87 |
| ILF3 | ENSG00000187522 | HSPA14 |
| ILF3 | ENSG00000105856 | HBP1 |
| ILF3 | ENSG00000255435 | RP11-770J1.3 |
| ILF3 | ENSG00000267302 | RP11-178C3.2 |
| ILF3 | ENSG00000266801 | RP11-521L9.2 |
| ILF3 | ENSG00000124383 | MPHOSPH10 |
| ILF3 | ENSG00000167925 | GHDC |
| ILF3 | ENSG00000136877 | FPGS |
| ILF3 | ENSG00000198270 | TMEM116 |
| ILF3 | ENSG00000215241 | RP11-266K4.9 |
| ILF3 | ENSG00000203963 | C1orf141 |
| ILF3 | ENSG00000079102 | RUNX1T1 |
| ILF3 | ENSG00000086827 | ZW10 |
| ILF3 | ENSG00000197006 | METTL9 |
| ILF3 | ENSG00000167110 | GOLGA2 |
| ILF3 | ENSG00000182095 | TNRC18 |
| ILF3 | ENSG00000221963 | APOL6 |
| ILF3 | ENSG00000244055 | AC007566.10 |
| ILF3 | ENSG00000158423 | RIBC1 |
| ILF3 | ENSG00000125740 | FOSB |
| ILF3 | ENSG00000216490 | IFI30 |
| ILF3 | ENSG00000185262 | UBALD2 |
| ILF3 | ENSG00000160211 | G6PD |
| ILF3 | ENSG00000250151 | ARPC4-TTLL3 |
| ILF3 | ENSG00000258232 | RP11-161H23.5 |
| ILF3 | ENSG00000107815 | C10orf2 |
| ILF3 | ENSG00000130427 | EPO |
| ILF3 | ENSG00000135775 | COG2 |
| ILF3 | ENSG00000172830 | SSH3 |
| ILF3 | ENSG00000136631 | VPS45 |
| ILF3 | ENSG00000239908 | RN7SL75P |
| ILF3 | ENSG00000071994 | PDCD2 |
| ILF3 | ENSG00000162526 | TSSK3 |
| ILF3 | ENSG00000164975 | SNAPC3 |
| ILF3 | ENSG00000202093 | SNORD58C |
| ILF3 | ENSG00000165644 | COMTD1 |
| ILF3 | ENSG00000233621 | LINC01137 |
| ILF3 | ENSG00000177606 | JUN |
| ILF3 | ENSG00000200913 | SNORD46 |
| ILF3 | ENSG00000004660 | CAMKK1 |
| ILF3 | ENSG00000197362 | ZNF786 |
| ILF3 | ENSG00000126247 | CAPNS1 |
| ILF3 | ENSG00000197483 | ZNF628 |
| ILF3 | ENSG00000108406 | DHX40 |
| ILF3 | ENSG00000153789 | FAM92B |
| ILF3 | ENSG00000248409 | KB-1269D1.8 |
| ILF3 | ENSG00000256713 | PGA5 |
| ILF3 | ENSG00000274270 | RP11-34F20.7 |
| ILF3 | ENSG00000277818 | Y_RNA |

| ILF3 | ENSG00000198816 | ZNF358 |
| --- | --- | --- |
| ILF3 | ENSG00000111859 | NEDD9 |
| ILF3 | ENSG00000167395 | ZNF646 |
| ILF3 | ENSG00000105607 | GCDH |
| ILF3 | ENSG00000172893 | DHCR7 |
| ILF3 | ENSG00000276790 | RP11-290H9.5 |
| ILF3 | ENSG00000240733 | RN7SL502P |
| ILF3 | ENSG00000100575 | TIMM9 |
| ILF3 | ENSG00000213186 | TRIM59 |
| ILF3 | ENSG00000115365 | LANCL1 |
| ILF3 | ENSG00000249488 | NACAP5 |
| ILF3 | ENSG00000199477 | SNORA31 |
| ILF3 | ENSG00000275948 | mascRNA- |
| ILF3 | ENSG00000261429 | menRNA DPPA2P4 |
| ILF3 | ENSG00000125375 | ATP5S |
| ILF3 | ENSG00000105357 | MYH14 |
| ILF3 | ENSG00000262519 | TXNP4 |
| ILF3 | ENSG00000233937 | CTC-338M12.4 |
| ILF3 | ENSG00000128805 | ARHGAP22 |
| ILF3 | ENSG00000182919 | C11orf54 |
| ILF3 | ENSG00000279423 | RP11-671J11.5 |
| ILF3 | ENSG00000149806 | FAU |
| ILF3 | ENSG00000236773 | RP11-365O16.1 |
| ILF3 | ENSG00000228634 | RP4-534N18.2 |
| ILF3 | ENSG00000167874 | TMEM88 |
| ILF3 | ENSG00000272081 | CTD-2376I4.2 |
| ILF3 | ENSG00000161970 | RPL26 |
| ILF3 | ENSG00000040608 | RTN4R |
| ILF3 | ENSG00000252186 | RNU6-781P |
| ILF3 | ENSG00000153786 | ZDHHC7 |
| ILF3 | ENSG00000155868 | MED7 |
| ILF3 | ENSG00000102265 | TIMP1 |
| ILF3 | ENSG00000141698 | NT5C3B |
| ILF3 | ENSG00000109475 | RPL34 |
| ILF3 | ENSG00000267335 | CTB-60B18.6 |
| ILF3 | ENSG00000277235 | RP4-550H1.7 |
| ILF3 | ENSG00000272240 | RP5-855D21.1 |
| ILF3 | ENSG00000205155 | PSENEN |
| ILF3 | ENSG00000275464 | CH507-9B2.5 |
| ILF3 | ENSG00000139269 | INHBE |
| ILF3 | ENSG00000231090 | RP11-101C11.1 |
| ILF3 | ENSG00000107404 | DVL1 |
| ILF3 | ENSG00000090432 | MUL1 |
| ILF3 | ENSG00000141759 | TXNL4A |
| ILF3 | ENSG00000167526 | RPL13 |
| ILF3 | ENSG00000236848 | RP11-401L13.5 |
| ILF3 | ENSG00000008324 | SS18L2 |
| ILF3 | ENSG00000231721 | LINC-PINT |
| ILF3 | ENSG00000228527 | RP11-179B15.5 |
| ILF3 | ENSG00000145216 | FIP1L1 |
| ILF3 | ENSG00000260235 | CTD-2026K11.3 |

| ILF3 | ENSG00000152382 | TADA1 |
| --- | --- | --- |
| ILF3 | ENSG00000241770 | RP11-555M1.3 |
| ILF3 | ENSG00000244086 | RPS20P35 |
| ILF3 | ENSG00000228478 | RP1-290I10.3 |
| ILF3 | ENSG00000131263 | RLIM |
| ILF3 | ENSG00000168477 | TNXB |
| ILF3 | ENSG00000119574 | ZBTB45 |
| ILF3 | ENSG00000161981 | SNRNP25 |
| ILF3 | ENSG00000252225 | Y_RNA |
| ILF3 | ENSG00000174996 | KLC2 |
| ILF3 | ENSG00000267147 | CTC-548K16.1 |
| ILF3 | ENSG00000159658 | EFCAB14 |
| ILF3 | ENSG00000092853 | CLSPN |
| ILF3 | ENSG00000230102 | RP11-407B7.1 |
| ILF3 | ENSG00000247675 | LRP4-AS1 |
| ILF3 | ENSG00000207457 | RNU6-476P |
| ILF3 | ENSG00000179715 | PCED1B |
| ILF3 | ENSG00000184277 | TM2D3 |
| ILF3 | ENSG00000256269 | HMBS |
| ILF3 | ENSG00000272540 | XXbac- |
| ILF3 | ENSG00000148908 | BPG252P9.9  RGS10 |
| ILF3 | ENSG00000272578 | AP000347.2 |
| ILF3 | ENSG00000170421 | KRT8 |
| ILF3 | ENSG00000101974 | ATP11C |
| ILF3 | ENSG00000089682 | RBM41 |
| ILF3 | ENSG00000232926 | AC000078.5 |
| ILF3 | ENSG00000240875 | LINC00886 |
| ILF3 | ENSG00000173848 | NET1 |
| ILF3 | ENSG00000171084 | FAM86JP |
| ILF3 | ENSG00000132394 | EEFSEC |
| ILF3 | ENSG00000202399 | Y_RNA |
| ILF3 | ENSG00000124570 | SERPINB6 |
| ILF3 | ENSG00000131381 | ZFYVE20 |
| ILF3 | ENSG00000260038 | RP11-407G23.4 |
| ILF3 | ENSG00000006607 | FARP2 |
| ILF3 | ENSG00000144026 | ZNF514 |
| ILF3 | ENSG00000253190 | AC084082.3 |
| ILF3 | ENSG00000257964 | RP11-133N21.10 |
| ILF3 | ENSG00000119596 | YLPM1 |
| ILF3 | ENSG00000182362 | YBEY |
| ILF3 | ENSG00000214796 | RP11-480I12.5 |
| ILF3 | ENSG00000160799 | CCDC12 |
| ILF3 | ENSG00000083097 | DOPEY1 |
| ILF3 | ENSG00000003056 | M6PR |
| ILF3 | ENSG00000073921 | PICALM |
| ILF3 | ENSG00000266328 | hsa-mir-4536-1 |
| ILF3 | ENSG00000157259 | GATAD1 |
| ILF3 | ENSG00000213672 | NCKIPSD |
| ILF3 | ENSG00000182923 | CEP63 |
| ILF3 | ENSG00000261314 | RP11-359E8.5 |
| ILF3 | ENSG00000226051 | ZNF503-AS1 |

| ILF3 | ENSG00000068878 | PSME4 |
| --- | --- | --- |
| ILF3 | ENSG00000201229 | SNORA63 |
| ILF3 | ENSG00000085788 | DDHD2 |
| ILF3 | ENSG00000126368 | NR1D1 |
| ILF3 | ENSG00000058600 | POLR3E |
| ILF3 | ENSG00000265095 | FTLP12 |
| ILF3 | ENSG00000218891 | ZNF579 |
| ILF3 | ENSG00000148834 | GSTO1 |
| ILF3 | ENSG00000159648 | TEPP |
| ILF3 | ENSG00000199603 | RNU6-951P |
| ILF3 | ENSG00000074071 | MRPS34 |
| ILF3 | ENSG00000242315 | RN7SL685P |
| ILF3 | ENSG00000048028 | USP28 |
| ILF3 | ENSG00000229474 | PATL2 |
| ILF3 | ENSG00000079332 | SAR1A |
| ILF3 | ENSG00000188566 | NDOR1 |
| ILF3 | ENSG00000117481 | NSUN4 |
| ILF3 | ENSG00000124243 | BCAS4 |
| ILF3 | ENSG00000206754 | SNORD101 |
| ILF3 | ENSG00000241563 | CORT |
| ILF3 | ENSG00000264732 | MIR4638 |
| ILF3 | ENSG00000267898 | CTD-2639E6.9 |
| ILF3 | ENSG00000159479 | MED8 |
| ILF3 | ENSG00000108825 | PTGES3L- |
| ILF3 | ENSG00000165506 | AARSD1 DNAAF2 |
| ILF3 | ENSG00000248309 | MEF2C-AS1 |
| ILF3 | ENSG00000100644 | HIF1A |
| ILF3 | ENSG00000259346 | RP11-349G13.2 |
| ILF3 | ENSG00000080189 | SLC35C2 |
| ILF3 | ENSG00000102890 | ELMO3 |
| ILF3 | ENSG00000086159 | AQP6 |
| ILF3 | ENSG00000227932 | RP13-16H11.2 |
| ILF3 | ENSG00000237310 | GS1-124K5.4 |
| ILF3 | ENSG00000258553 | RP11-16B13.1 |
| ILF3 | ENSG00000251754 | RNU6-999P |
| ILF3 | ENSG00000169379 | ARL13B |
| ILF3 | ENSG00000170291 | ELP5 |
| ILF3 | ENSG00000118418 | HMGN3 |
| ILF3 | ENSG00000172380 | GNG12 |
| ILF3 | ENSG00000054965 | FAM168A |
| ILF3 | ENSG00000111912 | NCOA7 |
| ILF3 | ENSG00000234496 | MRPS21P1 |
| ILF3 | ENSG00000276984 | LA16c-359F1.1 |
| ILF3 | ENSG00000201403 | SNORD14B |
| ILF3 | ENSG00000225190 | PLEKHM1 |
| ILF3 | ENSG00000158296 | SLC13A3 |
| ILF3 | ENSG00000148950 | IMMP1L |
| ILF3 | ENSG00000140740 | UQCRC2 |
| ILF3 | ENSG00000117174 | ZNHIT6 |
| ILF3 | ENSG00000148362 | C9orf142 |
| ILF3 | ENSG00000173457 | PPP1R14B |

| ILF3 | ENSG00000131747 | TOP2A |
| --- | --- | --- |
| ILF3 | ENSG00000155629 | PIK3AP1 |
| ILF3 | ENSG00000226621 | AC083855.5 |
| ILF3 | ENSG00000179021 | C3orf38 |
| ILF3 | ENSG00000132256 | TRIM5 |
| ILF3 | ENSG00000106415 | GLCCI1 |
| ILF3 | ENSG00000089916 | GPATCH2L |
| ILF3 | ENSG00000259071 | RP11-247L20.4 |
| ILF3 | ENSG00000148842 | CNNM2 |
| ILF3 | ENSG00000259053 | RP11-33N16.3 |
| ILF3 | ENSG00000107938 | EDRF1 |
| ILF3 | ENSG00000222821 | RNU4-27P |
| ILF3 | ENSG00000134644 | PUM1 |
| ILF3 | ENSG00000203325 | RP11-277A4.4 |
| ILF3 | ENSG00000234230 | ZFX-AS1 |
| ILF3 | ENSG00000116667 | C1orf21 |
| ILF3 | ENSG00000207771 | MIR550A1 |
| ILF3 | ENSG00000158850 | B4GALT3 |
| ILF3 | ENSG00000106399 | RPA3 |
| ILF3 | ENSG00000253392 | AC006277.2 |
| ILF3 | ENSG00000183691 | NOG |
| ILF3 | ENSG00000124507 | PACSIN1 |
| ILF3 | ENSG00000199509 | RNA5SP477 |
| ILF3 | ENSG00000149428 | HYOU1 |
| ILF3 | ENSG00000106299 | WASL |
| ILF3 | ENSG00000087253 | LPCAT2 |
| ILF3 | ENSG00000221355 | MIR1288 |
| ILF3 | ENSG00000167632 | TRAPPC9 |
| ILF3 | ENSG00000163666 | HESX1 |
| ILF3 | ENSG00000224721 | AC007182.6 |
| ILF3 | ENSG00000161860 | SYCE2 |
| ILF3 | ENSG00000104870 | FCGRT |
| ILF3 | ENSG00000144674 | GOLGA4 |
| ILF3 | ENSG00000256950 | RP11-87C12.2 |
| ILF3 | ENSG00000122126 | OCRL |
| ILF3 | ENSG00000279696 | RP11-178H8.7 |
| ILF3 | ENSG00000145817 | YIPF5 |
| ILF3 | ENSG00000259627 | RP11-244F12.2 |
| ILF3 | ENSG00000206013 | IFITM5 |
| ILF3 | ENSG00000040487 | PQLC2 |
| ILF3 | ENSG00000175841 | FAM172BP |
| ILF3 | ENSG00000188396 | TCTEX1D4 |
| ILF3 | ENSG00000091157 | WDR7 |
| ILF3 | ENSG00000227848 | SUCLA2-AS1 |
| ILF3 | ENSG00000245857 | GS1-24F4.2 |
| ILF3 | ENSG00000186432 | KPNA4 |
| ILF3 | ENSG00000122877 | EGR2 |
| ILF3 | ENSG00000269961 | CTD-2033C11.1 |
| ILF3 | ENSG00000197150 | ABCB8 |
| ILF3 | ENSG00000267321 | RP11-1094M14.11 |
| ILF3 | ENSG00000151835 | SACS |

| ILF3 | ENSG00000240803 | RN7SL231P |
| --- | --- | --- |
| ILF3 | ENSG00000273820 | USP27X |
| ILF3 | ENSG00000267174 | CTC-510F12.4 |
| ILF3 | ENSG00000162460 | TMEM82 |
| ILF3 | ENSG00000271797 | CTC-428G20.6 |
| ILF3 | ENSG00000148204 | CRB2 |
| ILF3 | ENSG00000236104 | ZBTB22 |
| ILF3 | ENSG00000141646 | SMAD4 |
| ILF3 | ENSG00000197136 | PCNXL3 |
| ILF3 | ENSG00000153989 | NUS1 |
| ILF3 | ENSG00000267552 | CTD-2528L19.4 |
| ILF3 | ENSG00000074266 | EED |
| ILF3 | ENSG00000275538 | RNVU1-19 |
| ILF3 | ENSG00000179922 | ZNF784 |
| ILF3 | ENSG00000163138 | PACRGL |
| ILF3 | ENSG00000218018 | RP4-800J21.3 |
| ILF3 | ENSG00000064666 | CNN2 |
| ILF3 | ENSG00000277161 | PIGW |
| ILF3 | ENSG00000156787 | TBC1D31 |
| ILF3 | ENSG00000237489 | LINC00959 |
| ILF3 | ENSG00000101447 | FAM83D |
| ILF3 | ENSG00000167674 | HDGFRP2; |
| ILF3 | ENSG00000137073 | UBAP2 |
| ILF3 | ENSG00000188086 | PRSS45 |
| ILF3 | ENSG00000206799 | SNORA32 |
| ILF3 | ENSG00000166272 | WBP1L |
| ILF3 | ENSG00000100813 | ACIN1 |
| ILF3 | ENSG00000273174 | RP11-434H6.6 |
| ILF3 | ENSG00000010310 | GIPR |
| ILF3 | ENSG00000129991 | TNNI3 |
| ILF3 | ENSG00000188015 | S100A3 |
| ILF3 | ENSG00000252443 | SNORA62 |
| ILF3 | ENSG00000112701 | SENP6 |
| ILF3 | ENSG00000223217 | RNU6-938P |
| ILF3 | ENSG00000179761 | PIPOX |
| ILF3 | ENSG00000173473 | SMARCC1 |
| ILF3 | ENSG00000141503 | MINK1 |
| ILF3 | ENSG00000170638 | TRABD |
| ILF3 | ENSG00000175206 | NPPA |
| ILF3 | ENSG00000259943 | RP1-39G22.7 |
| ILF3 | ENSG00000221500 | SNORD100 |
| ILF3 | ENSG00000102870 | ZNF629 |
| ILF3 | ENSG00000268543 | CTD-2619J13.16 |
| ILF3 | ENSG00000169727 | GPS1 |
| ILF3 | ENSG00000155729 | KCTD18 |
| ILF3 | ENSG00000156413 | FUT6 |
| ILF3 | ENSG00000166170 | BAG5 |
| ILF3 | ENSG00000121905 | HPCA |
| ILF3 | ENSG00000113525 | IL5 |
| ILF3 | ENSG00000085741 | WNT11 |
| ILF3 | ENSG00000267422 | CTD-2554C21.1 |

| ILF3 | ENSG00000257346 | RP11-386G11.8 |
| --- | --- | --- |
| ILF3 | ENSG00000260734 | RP11-510M2.4 |
| ILF3 | ENSG00000132275 | RRP8 |
| ILF3 | ENSG00000197050 | ZNF420 |
| ILF3 | ENSG00000144659 | SLC25A38 |
| ILF3 | ENSG00000197106 | SLC6A17 |
| ILF3 | ENSG00000165804 | ZNF219 |
| ILF3 | ENSG00000269153 | LYPLA2P2 |
| ILF3 | ENSG00000257962 | RP11-219B4.6 |
| ILF3 | ENSG00000167733 | HSD11B1L |
| ILF3 | ENSG00000278311 | GGNBP2 |
| ILF3 | ENSG00000213096 | ZNF254 |
| ILF3 | ENSG00000263167 | RP11-149I9.2 |
| ILF3 | ENSG00000197566 | ZNF624 |
| ILF3 | ENSG00000143457 | GOLPH3L |
| ILF3 | ENSG00000013275 | PSMC4 |
| ILF3 | ENSG00000270024 | C8orf44-SGK3 |
| ILF3 | ENSG00000108352 | RAPGEFL1 |
| ILF3 | ENSG00000198952 | SMG5 |
| ILF3 | ENSG00000204347 | BTBD17 |
| ILF3 | ENSG00000201754 | SNORD52 |
| ILF3 | ENSG00000268043 | NBPF12 |
| ILF3 | ENSG00000263280 | LA16c-325D7.2 |
| ILF3 | ENSG00000278829 | RP11-358B23.7 |
| ILF3 | ENSG00000244459 | RP11-1398P2.1 |
| ILF3 | ENSG00000197191 | CYSRT1 |
| ILF3 | ENSG00000204469 | PRRC2A |
| ILF3 | ENSG00000268947 | AD000684.2 |
| ILF3 | ENSG00000229604 | MTATP8P2 |
| ILF3 | ENSG00000279873 | LINC01126 |
| ILF3 | ENSG00000163545 | NUAK2 |
| ILF3 | ENSG00000170581 | STAT2 |
| ILF3 | ENSG00000205476 | CCDC85C |
| ILF3 | ENSG00000196187 | TMEM63A |
| ILF3 | ENSG00000278842 | RP3-405J10.5 |
| ILF3 | ENSG00000143493 | INTS7 |
| ILF3 | ENSG00000176043 | RP1-146I3.1 |
| ILF3 | ENSG00000170989 | S1PR1 |
| ILF3 | ENSG00000007038 | PRSS21 |
| ILF3 | ENSG00000174840 | PDE12 |
| ILF3 | ENSG00000135637 | CCDC142 |
| ILF3 | ENSG00000175471 | MCTP1 |
| ILF3 | ENSG00000169554 | ZEB2 |
| ILF3 | ENSG00000068097 | HEATR6 |
| ILF3 | ENSG00000178397 | FAM220A |
| ILF3 | ENSG00000187790 | FANCM |
| ILF3 | ENSG00000116478 | HDAC1 |
| ILF3 | ENSG00000196152 | ZNF79 |
| ILF3 | ENSG00000280303 | RP11-642A1.3 |
| ILF3 | ENSG00000279617 | AC005796.2 |
| ILF3 | ENSG00000280194 | AD000864.6 |

| ILF3 | ENSG00000165521 | EML5 |
| --- | --- | --- |
| ILF3 | ENSG00000214736 | TOMM6 |
| ILF3 | ENSG00000180228 | PRKRA |
| ILF3 | ENSG00000112578 | BYSL |
| ILF3 | ENSG00000180481 | GLIPR1L2 |
| ILF3 | ENSG00000207200 | RNU6-45P |
| ILF3 | ENSG00000131242 | RAB11FIP4 |
| ILF3 | ENSG00000213676 | ATF6B |
| ILF3 | ENSG00000272844 | RP11-484K9.4 |
| ILF3 | ENSG00000216819 | TUBB2BP1 |
| ILF3 | ENSG00000109685 | WHSC1 |
| ILF3 | ENSG00000225407 | CTD-2384B11.2 |
| ILF3 | ENSG00000140750 | ARHGAP17 |
| ILF3 | ENSG00000264741 | MIR4505 |
| ILF3 | ENSG00000200102 | RNU6-252P |
| ILF3 | ENSG00000239948 | RN7SL368P |
| ILF3 | ENSG00000115661 | STK16 |
| ILF3 | ENSG00000266913 | CTC-548K16.2 |
| ILF3 | ENSG00000260814 | RP11-107F6.3 |
| ILF3 | ENSG00000008382 | MPND |
| ILF3 | ENSG00000259030 | FPGT-TNNI3K |
| ILF3 | ENSG00000130699 | TAF4 |
| ILF3 | ENSG00000131459 | GFPT2 |
| ILF3 | ENSG00000167595 | PROSER3 |
| ILF3 | ENSG00000269516 | CYP4F23P |
| ILF3 | ENSG00000264920 | RP11-6N17.4 |
| ILF3 | ENSG00000205060 | SLC35B4 |
| ILF3 | ENSG00000092621 | PHGDH |
| ILF3 | ENSG00000250900 | CTC-338M12.6 |
| ILF3 | ENSG00000255050 | RP11-661A12.9 |
| ILF3 | ENSG00000076201 | PTPN23 |
| ILF3 | ENSG00000107902 | LHPP |
| ILF3 | ENSG00000130734 | ATG4D |
| ILF3 | ENSG00000155393 | HEATR3 |
| ILF3 | ENSG00000100219 | XBP1 |
| ILF3 | ENSG00000253719 | ATXN7L3B |
| ILF3 | ENSG00000163939 | PBRM1 |
| ILF3 | ENSG00000170802 | FOXN2 |
| ILF3 | ENSG00000088280 | ASAP3 |
| ILF3 | ENSG00000229786 | SNRPFP2 |
| ILF3 | ENSG00000251349 | MSANTD3- |
| ILF3 | ENSG00000264608 | TMEFF1  RP11-192H23.8 |
| ILF3 | ENSG00000267211 | RP5-1067M6.3 |
| ILF3 | ENSG00000137077 | CCL21 |
| ILF3 | ENSG00000160796 | NBEAL2 |
| ILF3 | ENSG00000188585 | LINC00083 |
| ILF3 | ENSG00000277170 | RP11-295D4.4 |
| ILF3 | ENSG00000134824 | FADS2 |
| ILF3 | ENSG00000137709 | POU2F3 |
| ILF3 | ENSG00000163879 | DNALI1 |
| ILF3 | ENSG00000253613 | CTC-551A13.2 |

| ILF3 | ENSG00000213742 | ZNF337-AS1 |
| --- | --- | --- |
| ILF3 | ENSG00000100526 | CDKN3 |
| ILF3 | ENSG00000005483 | KMT2E |
| ILF3 | ENSG00000169710 | FASN |
| ILF3 | ENSG00000276663 | RP11-407G23.7 |
| ILF3 | ENSG00000144785 | RP11-977G19.10 |
| ILF3 | ENSG00000163312 | HELQ |
| ILF3 | ENSG00000107643 | MAPK8 |
| ILF3 | ENSG00000173914 | RBM4B |
| ILF3 | ENSG00000146411 | SLC2A12 |
| ILF3 | ENSG00000040531 | CTNS |
| ILF3 | ENSG00000264044 | RP11-192H23.7 |
| ILF3 | ENSG00000218472 | RP1-140K8.2 |
| ILF3 | ENSG00000254949 | GNG5P3 |
| ILF3 | ENSG00000143507 | DUSP10 |
| ILF3 | ENSG00000165502 | RPL36AL |
| ILF3 | ENSG00000204560 | DHX16 |
| ILF3 | ENSG00000120738 | EGR1 |
| ILF3 | ENSG00000243022 | MARK3P3 |
| ILF3 | ENSG00000102921 | N4BP1 |
| ILF3 | ENSG00000262213 | AC144836.1 |
| ILF3 | ENSG00000224717 | RP11-576D8.4 |
| ILF3 | ENSG00000114993 | RTKN |
| ILF3 | ENSG00000199482 | RNU6-633P |
| ILF3 | ENSG00000144736 | SHQ1 |
| ILF3 | ENSG00000137331 | IER3 |
| ILF3 | ENSG00000275334 | MIR5787 |
| ILF3 | ENSG00000132388 | UBE2G1 |
| ILF3 | ENSG00000224914 | LINC00863 |
| ILF3 | ENSG00000232131 | NCOA7-AS1 |
| ILF3 | ENSG00000005700 | IBTK |
| ILF3 | ENSG00000049768 | FOXP3 |
| ILF3 | ENSG00000163006 | CCDC138 |
| ILF3 | ENSG00000199787 | SNORA42 |
| ILF3 | ENSG00000231046 | RP11-428F8.2 |
| ILF3 | ENSG00000274998 | SNORA17 |
| ILF3 | ENSG00000143514 | TP53BP2 |
| ILF3 | ENSG00000091009 | RBM27 |
| ILF3 | ENSG00000122779 | TRIM24 |
| ILF3 | ENSG00000164949 | GEM |
| ILF3 | ENSG00000260005 | AC027601.1 |
| ILF3 | ENSG00000136717 | BIN1 |
| ILF3 | ENSG00000263798 | RP11-6N17.9 |
| ILF3 | ENSG00000162702 | ZNF281 |
| ILF3 | ENSG00000251141 | RP11-53O19.1 |
| ILF3 | ENSG00000205213 | LGR4 |
| ILF3 | ENSG00000123353 | ORMDL2 |
| ILF3 | ENSG00000148429 | USP6NL |
| ILF3 | ENSG00000135766 | EGLN1 |
| ILF3 | ENSG00000135698 | MPHOSPH6 |
| ILF3 | ENSG00000151338 | MIPOL1 |

| ILF3 | ENSG00000115268 | RPS15 |
| --- | --- | --- |
| ILF3 | ENSG00000252947 | SCARNA1 |
| ILF3 | ENSG00000252042 | Y_RNA |
| ILF3 | ENSG00000186001 | LRCH3 |
| ILF3 | ENSG00000270587 | RP11-51F16.9 |
| ILF3 | ENSG00000104093 | DMXL2 |
| ILF3 | ENSG00000135679 | MDM2 |
| ILF3 | ENSG00000251656 | CTD-2201E18.4 |
| ILF3 | ENSG00000197183 | NOL4L |
| ILF3 | ENSG00000064703 | DDX20 |
| ILF3 | ENSG00000060237 | WNK1 |
| ILF3 | ENSG00000181751 | C5orf30 |
| ILF3 | ENSG00000227728 | RP11-690C23.4 |
| ILF3 | ENSG00000111671 | SPSB2 |
| ILF3 | ENSG00000214097 | SMCO1 |
| ILF3 | ENSG00000238105 | GOLGA2P5 |
| ILF3 | ENSG00000149926 | FAM57B |
| ILF3 | ENSG00000269387 | RP11-298J23.8 |
| ILF3 | ENSG00000254858 | MPV17L2 |
| ILF3 | ENSG00000269706 | CTB-60B18.15 |
| ILF3 | ENSG00000116171 | SCP2 |
| ILF3 | ENSG00000243150 | RP11-538P18.2 |
| ILF3 | ENSG00000119559 | C19orf25 |
| ILF3 | ENSG00000082516 | GEMIN5 |
| ILF3 | ENSG00000272123 | CTD-2366F13.2 |
| ILF3 | ENSG00000261587 | TMEM249 |
| ILF3 | ENSG00000197989 | SNHG12 |
| ILF3 | ENSG00000226243 | RPL37AP1 |
| ILF3 | ENSG00000167685 | ZNF444 |
| ILF3 | ENSG00000153814 | JAZF1 |
| ILF3 | ENSG00000270980 | RP11-626A5.2 |
| ILF3 | ENSG00000249209 | AP000304.12 |
| ILF3 | ENSG00000063515 | GSC2 |
| ILF3 | ENSG00000203804 | ADAMTSL4- |
| ILF3 | ENSG00000147457 | AS1 CHMP7 |
| ILF3 | ENSG00000164938 | TP53INP1 |
| ILF3 | ENSG00000146872 | TLK2 |
| ILF3 | ENSG00000163596 | ICA1L |
| ILF3 | ENSG00000200976 | Y_RNA |
| ILF3 | ENSG00000205865 | FAM99B |
| ILF3 | ENSG00000020219 | CCT8L1P |
| ILF3 | ENSG00000198843 | SELT |
| ILF3 | ENSG00000164615 | CAMLG |
| ILF3 | ENSG00000252178 | RNU7-69P |
| ILF3 | ENSG00000260570 | RP11-24N18.1 |
| ILF3 | ENSG00000158828 | PINK1 |
| ILF3 | ENSG00000031003 | FAM13B |
| ILF3 | ENSG00000267838 | AC008746.12 |
| ILF3 | ENSG00000076924 | XAB2 |
| ILF3 | ENSG00000175336 | APOF |
| ILF3 | ENSG00000109445 | ZNF330 |

| ILF3 | ENSG00000225855 | RUSC1-AS1 |
| --- | --- | --- |
| ILF3 | ENSG00000100568 | VTI1B |
| ILF3 | ENSG00000189223 | PAX8-AS1 |
| ILF3 | ENSG00000118655 | DCLRE1B |
| ILF3 | ENSG00000261610 | AP000265.1 |
| ILF3 | ENSG00000242562 | DCAF13P1 |
| ILF3 | ENSG00000268366 | CTC-492K19.4 |
| ILF3 | ENSG00000168175 | MAPK1IP1L |
| ILF3 | ENSG00000169750 | RAC3 |
| ILF3 | ENSG00000163617 | KIAA1407 |
| ILF3 | ENSG00000270326 | RP5-874C20.6 |
| ILF3 | ENSG00000259672 | RP11-69G7.1 |
| ILF3 | ENSG00000170537 | TMC7 |
| ILF3 | ENSG00000157191 | NECAP2 |
| ILF3 | ENSG00000274215 | CTD-2313J17.6 |
| ILF3 | ENSG00000250159 | RP11-381K20.2 |
| ILF3 | ENSG00000115539 | PDCL3 |
| ILF3 | ENSG00000239327 | RP11-14J7.1 |
| ILF3 | ENSG00000112137 | PHACTR1 |
| ILF3 | ENSG00000168394 | TAP1 |
| ILF3 | ENSG00000257605 | RP11-680A11.5 |
| ILF3 | ENSG00000131370 | SH3BP5 |
| ILF3 | ENSG00000120915 | EPHX2 |
| ILF3 | ENSG00000267291 | CTB-186G2.1 |
| ILF3 | ENSG00000174939 | ASPHD1 |
| ILF3 | ENSG00000235748 | RP11-32D17.4 |
| ILF3 | ENSG00000160959 | LRRC14 |
| ILF3 | ENSG00000133808 | MICALCL |
| ILF3 | ENSG00000165996 | PTPLA |
| ILF3 | ENSG00000144566 | RAB5A |
| ILF3 | ENSG00000201659 | RNU12-2P |
| ILF3 | ENSG00000229414 | KCNQ1-AS1 |
| ILF3 | ENSG00000025708 | TYMP |
| ILF3 | ENSG00000263843 | RP11-649A18.12 |
| ILF3 | ENSG00000103591 | AAGAB |
| ILF3 | ENSG00000182544 | MFSD5 |
| ILF3 | ENSG00000237438 | CECR7 |
| ILF3 | ENSG00000267244 | CTB-31O20.4 |
| ILF3 | ENSG00000148700 | ADD3 |
| ILF3 | ENSG00000168016 | TRANK1 |
| ILF3 | ENSG00000147689 | FAM83A |
| ILF3 | ENSG00000179913 | B3GNT3 |
| ILF3 | ENSG00000113758 | DBN1 |
| ILF3 | ENSG00000226979 | LTA |
| ILF3 | ENSG00000132326 | PER2 |
| ILF3 | ENSG00000133884 | DPF2 |
| ILF3 | ENSG00000213853 | EMP2 |
| ILF3 | ENSG00000233654 | AC093388.3 |
| ILF3 | ENSG00000203999 | LINC01270 |
| ILF3 | ENSG00000158773 | USF1 |
| ILF3 | ENSG00000196466 | ZNF799 |

| ILF3 | ENSG00000179577 | KB-1552D7.2 |
| --- | --- | --- |
| ILF3 | ENSG00000259262 | NDUFA3P4 |
| ILF3 | ENSG00000149313 | AASDHPPT |
| ILF3 | ENSG00000260464 | RP4-561L24.3 |
| ILF3 | ENSG00000250261 | AC007050.18 |
| ILF3 | ENSG00000135083 | CCNJL |
| ILF3 | ENSG00000172771 | EFCAB12 |
| ILF3 | ENSG00000219702 | RP5-991C6.2 |
| ILF3 | ENSG00000274286 | ADRA2B |
| ILF3 | ENSG00000205208 | C4orf46 |
| ILF3 | ENSG00000160993 | ALKBH4 |
| ILF3 | ENSG00000198755 | RPL10A |
| ILF3 | ENSG00000136108 | CKAP2 |
| ILF3 | ENSG00000123643 | SLC36A1 |
| ILF3 | ENSG00000229368 | AC090587.4 |
| ILF3 | ENSG00000120458 | MSANTD2 |
| ILF3 | ENSG00000214114 | MYCBP |
| ILF3 | ENSG00000132773 | TOE1 |
| ILF3 | ENSG00000240857 | RDH14 |
| ILF3 | ENSG00000213470 | RP11-972K6.1 |
| ILF3 | ENSG00000246731 | MGC16275 |
| ILF3 | ENSG00000151065 | DCP1B |
| ILF3 | ENSG00000099940 | SNAP29 |
| ILF3 | ENSG00000200121 | Y_RNA |
| ILF3 | ENSG00000082641 | NFE2L1 |
| ILF3 | ENSG00000169258 | GPRIN1 |
| ILF3 | ENSG00000255856 | RP11-87C12.5 |
| ILF3 | ENSG00000164167 | LSM6 |
| ILF3 | ENSG00000234075 | RPL35AP |
| ILF3 | ENSG00000063015 | SEZ6 |
| ILF3 | ENSG00000185761 | ADAMTSL5 |
| ILF3 | ENSG00000197334 | Metazoa_SRP |
| ILF3 | ENSG00000167578 | RAB4B |
| ILF3 | ENSG00000252034 | RNY4P37 |
| ILF3 | ENSG00000214140 | PRCD |
| ILF3 | ENSG00000270083 | RP1-257I20.14 |
| ILF3 | ENSG00000258337 | RP11-115H15.2 |
| ILF3 | ENSG00000013375 | PGM3 |
| ILF3 | ENSG00000237491 | RP11-206L10.9 |
| ILF3 | ENSG00000242485 | MRPL20 |
| ILF3 | ENSG00000234545 | FAM133B |
| ILF3 | ENSG00000012048 | BRCA1 |
| ILF3 | ENSG00000244952 | RP11-1000B6.5 |
| ILF3 | ENSG00000253806 | CTD-2292P10.2 |
| ILF3 | ENSG00000225091 | SNORA71A |
| ILF3 | ENSG00000077254 | USP33 |
| ILF3 | ENSG00000141582 | CBX4 |
| ILF3 | ENSG00000111907 | TPD52L1 |
| ILF3 | ENSG00000171135 | JAGN1 |
| ILF3 | ENSG00000130592 | LSP1 |
| ILF3 | ENSG00000262903 | RP11-235E17.6 |

| ILF3 | ENSG00000244266 | RP11-112N23.1 |
| --- | --- | --- |
| ILF3 | ENSG00000235831 | BHLHE40-AS1 |
| ILF3 | ENSG00000247317 | RP11-273G15.2 |
| ILF3 | ENSG00000272189 | RP3-325F22.5 |
| ILF3 | ENSG00000267472 | LOC440461 |
| ILF3 | ENSG00000158113 | LRRC43 |
| ILF3 | ENSG00000266651 | RP11-138I1.3 |
| ILF3 | ENSG00000127311 | HELB |
| ILF3 | ENSG00000256053 | APOPT1 |
| ILF3 | ENSG00000233806 | LINC01237 |
| ILF3 | ENSG00000134668 | SPOCD1 |
| ILF3 | ENSG00000070182 | SPTB |
| ILF3 | ENSG00000267783 | CTB-55O6.10 |
| ILF3 | ENSG00000179902 | C1orf194 |
| ILF3 | ENSG00000135473 | PAN2 |
| ILF3 | ENSG00000275964 | RP11-61K9.3 |
| ILF3 | ENSG00000226273 | AC079305.5 |
| ILF3 | ENSG00000215421 | ZNF407 |
| ILF3 | ENSG00000264474 | MIR4656 |
| ILF3 | ENSG00000188312 | CENPP |
| ILF3 | ENSG00000131375 | CAPN7 |
| ILF3 | ENSG00000224152 | AC009506.1 |
| ILF3 | ENSG00000103024 | NME3 |
| ILF3 | ENSG00000137522 | RNF121 |
| ILF3 | ENSG00000136305 | CIDEB |
| ILF3 | ENSG00000171320 | ESCO2 |
| ILF3 | ENSG00000131943 | C19orf12 |
| ILF3 | ENSG00000052344 | PRSS8 |
| ILF3 | ENSG00000232937 | RP11-98D18.2 |
| ILF3 | ENSG00000174938 | SEZ6L2 |
| ILF3 | ENSG00000135365 | PHF21A |
| ILF3 | ENSG00000275910 | RP11-680G24.6 |
| ILF3 | ENSG00000188811 | NHLRC3 |
| ILF3 | ENSG00000235703 | LINC00894 |
| ILF3 | ENSG00000277767 | RP11-365P13.5 |
| ILF3 | ENSG00000166734 | CASC4 |
| ILF3 | ENSG00000231881 | RP5-1120P11.3 |
| ILF3 | ENSG00000243176 | RP11-550I24.2 |
| ILF3 | ENSG00000230699 | RP11-54O7.1 |
| ILF3 | ENSG00000178719 | GRINA |
| ILF3 | ENSG00000070413 | DGCR2 |
| ILF3 | ENSG00000188610 | FAM72B |
| ILF3 | ENSG00000129480 | DTD2 |
| ILF3 | ENSG00000070269 | TMEM260 |
| ILF3 | ENSG00000109501 | WFS1 |
| ILF3 | ENSG00000268392 | AC003682.16 |
| ILF3 | ENSG00000111647 | UHRF1BP1L |
| ILF3 | ENSG00000242042 | RP11-690C23.2 |
| ILF3 | ENSG00000130203 | APOE |
| ILF3 | ENSG00000119471 | HSDL2 |
| ILF3 | ENSG00000175727 | MLXIP |

| ILF3 | ENSG00000275714 | HIST1H3A |
| --- | --- | --- |
| ILF3 | ENSG00000166340 | TPP1 |
| ILF3 | ENSG00000232393 | RPL5P6 |
| ILF3 | ENSG00000132463 | GRSF1 |
| ILF3 | ENSG00000101000 | PROCR |
| ILF3 | ENSG00000040633 | PHF23 |
| ILF3 | ENSG00000276251 | CTB-32O4.3 |
| ILF3 | ENSG00000222601 | Y_RNA |
| ILF3 | ENSG00000199047 | MIR378A |
| ILF3 | ENSG00000272843 | RP11-313P13.5 |
| ILF3 | ENSG00000243976 | RN7SL523P |
| ILF3 | ENSG00000252169 | RNA5SP200 |
| ILF3 | ENSG00000041880 | PARP3 |
| ILF3 | ENSG00000153179 | RASSF3 |
| ILF3 | ENSG00000130177 | CDC16 |
| ILF3 | ENSG00000252659 | RNU6-1088P |
| ILF3 | ENSG00000124171 | PARD6B |
| ILF3 | ENSG00000201555 | Y_RNA |
| ILF3 | ENSG00000168300 | PCMTD1 |
| ILF3 | ENSG00000198298 | ZNF485 |
| ILF3 | ENSG00000115286 | NDUFS7 |
| ILF3 | ENSG00000212145 | U8 |
| ILF3 | ENSG00000270445 | RP11-972P1.8 |
| ILF3 | ENSG00000255236 | CTD-2655K5.1 |
| ILF3 | ENSG00000166562 | SEC11C |
| ILF3 | ENSG00000267517 | CTD-2231E14.4 |
| ILF3 | ENSG00000170631 | ZNF16 |
| ILF3 | ENSG00000264613 | RN7SL290P |
| ILF3 | ENSG00000040275 | SPDL1 |
| ILF3 | ENSG00000178802 | MPI |
| ILF3 | ENSG00000177994 | C2orf73 |
| ILF3 | ENSG00000158856 | DMTN |
| ILF3 | ENSG00000255239 | AP002954.6 |
| ILF3 | ENSG00000143384 | MCL1 |
| ILF3 | ENSG00000104765 | BNIP3L |
| ILF3 | ENSG00000228510 | RP11-339B21.8 |
| ILF3 | ENSG00000236618 | PITPNA-AS1 |
| ILF3 | ENSG00000092201 | SUPT16H |
| ILF3 | ENSG00000212916 | MAP10 |
| ILF3 | ENSG00000139746 | RBM26 |
| ILF3 | ENSG00000259704 | CTD- |
| ILF3 | ENSG00000105281 | 3094K11.1  SLC1A5 |
| ILF3 | ENSG00000175602 | CCDC85B |
| ILF3 | ENSG00000197265 | GTF2E2 |
| ILF3 | ENSG00000172936 | MYD88 |
| ILF3 | ENSG00000137145 | DENND4C |
| ILF3 | ENSG00000274206 | Metazoa_SRP |
| ILF3 | ENSG00000116819 | TFAP2E |
| ILF3 | ENSG00000164715 | LMTK2 |
| ILF3 | ENSG00000231508 | RP11-452K12.6 |
| ILF3 | ENSG00000253179 | CALCP |

| ILF3 | ENSG00000228265 | RALY-AS1 |
| --- | --- | --- |
| ILF3 | ENSG00000225032 | RP11-228B15.4 |
| ILF3 | ENSG00000057657 | PRDM1 |
| ILF3 | ENSG00000016864 | GLT8D1 |
| ILF3 | ENSG00000134461 | ANKRD16 |
| ILF3 | ENSG00000227355 | RP11-162D16.2 |
| ILF3 | ENSG00000168334 | XIRP1 |
| ILF3 | ENSG00000186104 | CYP2R1 |
| ILF3 | ENSG00000124786 | SLC35B3 |
| ILF3 | ENSG00000139726 | DENR |
| ILF3 | ENSG00000277692 | RP11-358N2.2 |
| ILF3 | ENSG00000238098 | ABCA17P |
| ILF3 | ENSG00000174448 | STARD6 |
| ILF3 | ENSG00000272006 | RP11-583F2.6 |
| ILF3 | ENSG00000059378 | PARP12 |
| ILF3 | ENSG00000204713 | TRIM27 |
| ILF3 | ENSG00000277020 | RP11-476H16.1 |
| ILF3 | ENSG00000136935 | GOLGA1 |
| ILF3 | ENSG00000265458 | RP13-20L14.6 |
| ILF3 | ENSG00000099625 | C19orf26 |
| ILF3 | ENSG00000115163 | CENPA |
| ILF3 | ENSG00000113851 | CRBN |
| ILF3 | ENSG00000165171 | WBSCR27 |
| ILF3 | ENSG00000167619 | TMEM145 |
| ILF3 | ENSG00000261822 | RP11-265N6.2 |
| ILF3 | ENSG00000179262 | RAD23A |
| ILF3 | ENSG00000266907 | AC006116.22 |
| ILF3 | ENSG00000240338 | RP11-331F4.4 |
| ILF3 | ENSG00000255874 | LINC00346 |
| ILF3 | ENSG00000160683 | CXCR5 |
| ILF3 | ENSG00000100325 | ASCC2 |
| ILF3 | ENSG00000273272 | CTA-384D8.34 |
| ILF3 | ENSG00000261094 | RP11-355O1.11 |
| ILF3 | ENSG00000145331 | TRMT10A |
| ILF3 | ENSG00000227959 | RP11-276H7.2 |
| ILF3 | ENSG00000198198 | SZT2 |
| ILF3 | ENSG00000111536 | IL26 |
| ILF3 | ENSG00000120798 | NR2C1 |
| ILF3 | ENSG00000261976 | RP11-506D12.5 |
| ILF3 | ENSG00000273555 | MIR6812 |
| ILF3 | ENSG00000198948 | MFAP3L |
| ILF3 | ENSG00000224287 | MSL3P1 |
| ILF3 | ENSG00000232324 | AC008440.10 |
| ILF3 | ENSG00000231681 | AC020743.3 |
| ILF3 | ENSG00000233785 | RP13-314C10.5 |
| ILF3 | ENSG00000262554 | LA16c-360H6.2 |
| ILF3 | ENSG00000224763 | FDPSP8 |
| ILF3 | ENSG00000201274 | RNA5SP192 |
| ILF3 | ENSG00000274825 | RP4-616B8.5 |
| ILF3 | ENSG00000159111 | MRPL10 |
| ILF3 | ENSG00000167800 | TBX10 |

| ILF3 | ENSG00000185800 | DMWD |
| --- | --- | --- |
| ILF3 | ENSG00000115944 | COX7A2L |
| ILF3 | ENSG00000164252 | AGGF1 |
| ILF3 | ENSG00000117448 | AKR1A1 |
| ILF3 | ENSG00000143793 | C1orf35 |
| ILF3 | ENSG00000214194 | LINC00998 |
| ILF3 | ENSG00000249884 | RNF103- |
| ILF3 | ENSG00000130300 | CHMP3 PLVAP |
| ILF3 | ENSG00000269335 | IKBKG |
| ILF3 | ENSG00000179772 | FOXS1 |
| ILF3 | ENSG00000102030 | NAA10 |
| ILF3 | ENSG00000167772 | ANGPTL4 |
| ILF3 | ENSG00000273456 | RP11-686O6.2 |
| ILF3 | ENSG00000136059 | VILL |
| ILF3 | ENSG00000235605 | RP5-827C21.1 |
| ILF3 | ENSG00000149761 | NUDT22 |
| ILF3 | ENSG00000279792 | RP11-893F2.18 |
| ILF3 | ENSG00000105520 | LPPR2 |
| ILF3 | ENSG00000050820 | BCAR1 |
| ILF3 | ENSG00000136144 | RCBTB1 |
| ILF3 | ENSG00000277344 | U1 |
| ILF3 | ENSG00000164151 | ICE1 |
| ILF3 | ENSG00000230911 | PPIHP1 |
| ILF3 | ENSG00000014138 | POLA2 |
| ILF3 | ENSG00000136709 | WDR33 |
| ILF3 | ENSG00000100239 | PPP6R2 |
| ILF3 | ENSG00000257023 | RP11-268P4.4 |
| ILF3 | ENSG00000199673 | SNORD16 |
| ILF3 | ENSG00000224515 | RP11-5K23.5 |
| ILF3 | ENSG00000232119 | MCTS1 |
| ILF3 | ENSG00000270170 | NCBP2-AS2 |
| ILF3 | ENSG00000189339 | SLC35E2B |
| ILF3 | ENSG00000257411 | RP11-603J24.9 |
| ILF3 | ENSG00000089060 | SLC8B1 |
| ILF3 | ENSG00000177595 | PIDD1 |
| ILF3 | ENSG00000169918 | OTUD7A |
| ILF3 | ENSG00000119801 | YPEL5 |
| ILF3 | ENSG00000141750 | STAC2 |
| ILF3 | ENSG00000118369 | USP35 |
| ILF3 | ENSG00000229043 | AC091729.9 |
| ILF3 | ENSG00000109171 | SLAIN2 |
| ILF3 | ENSG00000222455 | RNA5SP296 |
| ILF3 | ENSG00000198018 | ENTPD7 |
| ILF3 | ENSG00000175395 | ZNF25 |
| ILF3 | ENSG00000182759 | MAFA |
| ILF3 | ENSG00000110931 | CAMKK2 |
| ILF3 | ENSG00000140400 | MAN2C1 |
| ILF3 | ENSG00000101574 | METTL4 |
| ILF3 | ENSG00000143222 | UFC1 |
| ILF3 | ENSG00000254275 | LINC00824 |
| ILF3 | ENSG00000205220 | PSMB10 |

| ILF3 | ENSG00000214447 | FAM187A |
| --- | --- | --- |
| ILF3 | ENSG00000136811 | ODF2 |
| ILF3 | ENSG00000166199 | ALKBH3 |
| ILF3 | ENSG00000131477 | RAMP2 |
| ILF3 | ENSG00000086300 | SNX10 |
| ILF3 | ENSG00000087053 | MTMR2 |
| ILF3 | ENSG00000219481 | NBPF1 |
| ILF3 | ENSG00000273254 | RP1-100J12.1 |
| ILF3 | ENSG00000261723 | CTD-2196E14.3 |
| ILF3 | ENSG00000222328 | RNU2-2P |
| ILF3 | ENSG00000065457 | ADAT1 |
| ILF3 | ENSG00000189042 | ZNF567 |
| ILF3 | ENSG00000278505 | C17orf78 |
| ILF3 | ENSG00000258096 | RP11-474P2.2 |
| ILF3 | ENSG00000263749 | OOSP1P2 |
| ILF3 | ENSG00000261766 | RP11-22P6.2 |
| ILF3 | ENSG00000225675 | RP4-784A16.5 |
| ILF3 | ENSG00000059804 | SLC2A3 |
| ILF3 | ENSG00000113163 | COL4A3BP |
| ILF3 | ENSG00000253772 | RP11-34P1.1 |
| ILF3 | ENSG00000166922 | SCG5 |
| ILF3 | ENSG00000259874 | RP11-170L3.2 |
| ILF3 | ENSG00000229528 | RP5-1066H13.4 |
| ILF3 | ENSG00000158764 | ITLN2 |
| ILF3 | ENSG00000266041 | MIR4690 |
| ILF3 | ENSG00000134324 | LPIN1 |
| ILF3 | ENSG00000174485 | DENND4A |
| ILF3 | ENSG00000275449 | MIR6859-1 |
| ILF3 | ENSG00000262919 | FAM58A |
| ILF3 | ENSG00000210181 | RNU6ATAC4P |
| ILF3 | ENSG00000085982 | USP40 |
| ILF3 | ENSG00000054118 | THRAP3 |
| ILF3 | ENSG00000277184 | SNORA9 |
| ILF3 | ENSG00000259685 | CTD-2315E11.1 |
| ILF3 | ENSG00000262624 | RP11-104H15.9 |
| ILF3 | ENSG00000179636 | TPPP2 |
| ILF3 | ENSG00000270322 | RP11-697E2.10 |
| ILF3 | ENSG00000236108 | RP11-383G10.1 |
| ILF3 | ENSG00000243477 | NAT6 |
| ILF3 | ENSG00000270846 | RP11-674I16.1 |
| ILF3 | ENSG00000229326 | AC069154.4 |
| ILF3 | ENSG00000234988 | AC068538.4 |
| ILF3 | ENSG00000272368 | RP4-605O3.4 |
| ILF3 | ENSG00000274494 | MIR6832 |
| ILF3 | ENSG00000139718 | SETD1B |
| ILF3 | ENSG00000203799 | CCDC162P |
| ILF3 | ENSG00000166444 | ST5 |
| ILF3 | ENSG00000171766 | GATM |
| ILF3 | ENSG00000204356 | NELFE |
| ILF3 | ENSG00000181652 | ATG9B |
| ILF3 | ENSG00000133657 | ATP13A3 |

| ILF3 | ENSG00000127831 | VIL1 |
| --- | --- | --- |
| ILF3 | ENSG00000180479 | ZNF571 |
| ILF3 | ENSG00000115484 | CCT4 |
| ILF3 | ENSG00000265908 | RP11-20B24.6 |
| ILF3 | ENSG00000196544 | C17orf59 |
| ILF3 | ENSG00000225889 | AC074289.1 |
| ILF3 | ENSG00000160752 | FDPS |
| ILF3 | ENSG00000183775 | KCTD16 |
| ILF3 | ENSG00000132300 | PTCD3 |
| ILF3 | ENSG00000251666 | ZNF346-IT1 |
| ILF3 | ENSG00000156374 | PCGF6 |
| ILF3 | ENSG00000274487 | CH17-431G21.1 |
| ILF3 | ENSG00000109270 | LAMTOR3 |
| ILF3 | ENSG00000088812 | ATRN |
| ILF3 | ENSG00000226054 | MEMO1P1 |
| ILF3 | ENSG00000267024 | CTD-2588C8.8 |
| ILF3 | ENSG00000108100 | CCNY |
| ILF3 | ENSG00000167323 | STIM1 |
| ILF3 | ENSG00000204843 | DCTN1 |
| ILF3 | ENSG00000266149 | RP11-789C17.3 |
| ILF3 | ENSG00000170445 | HARS |
| ILF3 | ENSG00000174151 | CYB561D1 |
| ILF3 | ENSG00000213123 | TCTEX1D2 |
| ILF3 | ENSG00000185338 | SOCS1 |
| ILF3 | ENSG00000168273 | SMIM4 |
| ILF3 | ENSG00000201654 | RNU6-7 |
| ILF3 | ENSG00000185624 | P4HB |
| ILF3 | ENSG00000084693 | AGBL5 |
| ILF3 | ENSG00000081692 | JMJD4 |
| ILF3 | ENSG00000260514 | RP11-368N21.1 |
| ILF3 | ENSG00000214063 | TSPAN4 |
| ILF3 | ENSG00000160867 | FGFR4 |
| ILF3 | ENSG00000264571 | MIR4529 |
| ILF3 | ENSG00000119965 | C10orf88 |
| ILF3 | ENSG00000162980 | ARL5A |
| ILF3 | ENSG00000227237 | AL672294.1 |
| ILF3 | ENSG00000273261 | RP11-531F16.4 |
| ILF3 | ENSG00000271044 | RP11-255M6.1 |
| ILF3 | ENSG00000207547 | MIR25 |
| ILF3 | ENSG00000277901 | RP5-851M4.1 |
| ILF3 | ENSG00000264304 | RP11-20B24.7 |
| ILF3 | ENSG00000204370 | SDHD |
| ILF3 | ENSG00000154001 | PPP2R5E |
| ILF3 | ENSG00000212195 | U3 |
| ILF3 | ENSG00000163075 | CFAP221 |
| ILF3 | ENSG00000169564 | PCBP1 |
| ILF3 | ENSG00000183134 | PTGDR2 |
| ILF3 | ENSG00000085978 | ATG16L1 |
| ILF3 | ENSG00000275994 | SNORA24 |
| ILF3 | ENSG00000254981 | RP11-350N15.3 |
| ILF3 | ENSG00000132434 | LANCL2 |

| ILF3 | ENSG00000175463 | TBC1D10C |
| --- | --- | --- |
| ILF3 | ENSG00000165731 | RET |
| ILF3 | ENSG00000139734 | DIAPH3 |
| ILF3 | ENSG00000213310 | CYCSP19 |
| ILF3 | ENSG00000203666 | EFCAB2 |
| ILF3 | ENSG00000214659 | KRT8P26 |
| ILF3 | ENSG00000141524 | TMC6 |
| ILF3 | ENSG00000131143 | COX4I1 |
| ILF3 | ENSG00000179002 | TAS1R2 |
| ILF3 | ENSG00000058404 | CAMK2B |
| ILF3 | ENSG00000227141 | RP11-545A16.3 |
| ILF3 | ENSG00000135469 | COQ10A |
| ILF3 | ENSG00000242294 | STAG3L5P |
| ILF3 | ENSG00000096433 | ITPR3 |
| ILF3 | ENSG00000167419 | LPO |
| ILF3 | ENSG00000197329 | PELI1 |
| ILF3 | ENSG00000080345 | RIF1 |
| ILF3 | ENSG00000244184 | RP11-314A20.2 |
| ILF3 | ENSG00000254665 | RP11-152H18.3 |
| ILF3 | ENSG00000226824 | RP4-756H11.3 |
| ILF3 | ENSG00000124508 | BTN2A2 |
| ILF3 | ENSG00000236088 | COX10-AS1 |
| ILF3 | ENSG00000123179 | EBPL |
| ILF3 | ENSG00000261386 | CTD-2012K14.6 |
| ILF3 | ENSG00000276097 | AC006538.8 |
| ILF3 | ENSG00000236283 | AC013463.2 |
| ILF3 | ENSG00000182318 | ZSCAN22 |
| ILF3 | ENSG00000259099 | RP11-348M3.2 |
| ILF3 | ENSG00000236609 | ZNF853 |
| ILF3 | ENSG00000056972 | TRAF3IP2 |
| ILF3 | ENSG00000204511 | MCCD1 |
| ILF3 | ENSG00000131669 | NINJ1 |
| ILF3 | ENSG00000232888 | RPS11P5 |
| ILF3 | ENSG00000248103 | CTC-338M12.9 |
| ILF3 | ENSG00000125510 | OPRL1 |
| ILF3 | ENSG00000185361 | TNFAIP8L1 |
| ILF3 | ENSG00000222259 | RN7SKP114 |
| ILF3 | ENSG00000215788 | TNFRSF25 |
| ILF3 | ENSG00000169021 | UQCRFS1 |
| ILF3 | ENSG00000163131 | CTSS |
| ILF3 | ENSG00000105197 | TIMM50 |
| ILF3 | ENSG00000254996 | ANKHD1- |
| ILF3 | ENSG00000198799 | EIF4EBP3 LRIG2 |
| ILF3 | ENSG00000266897 | AC005546.2 |
| ILF3 | ENSG00000278863 | RP11-567L7.3 |
| ILF3 | ENSG00000266559 | MIR4530 |
| ILF3 | ENSG00000243749 | ZMYM6NB |
| ILF3 | ENSG00000135900 | MRPL44 |
| ILF3 | ENSG00000168124 | OR1F1 |
| ILF3 | ENSG00000163348 | PYGO2 |
| ILF3 | ENSG00000248671 | ALG1L9P |

| ILF3 | ENSG00000275445 | CTD-2649C14.3 |
| --- | --- | --- |
| ILF3 | ENSG00000133134 | BEX2 |
| ILF3 | ENSG00000226864 | ATE1-AS1 |
| ILF3 | ENSG00000213699 | SLC35F6 |
| ILF3 | ENSG00000252171 | Y_RNA |
| ILF3 | ENSG00000271754 | RP3-337H4.9 |
| ILF3 | ENSG00000155008 | APOOL |
| ILF3 | ENSG00000153933 | DGKE |
| ILF3 | ENSG00000009830 | POMT2 |
| ILF3 | ENSG00000138286 | FAM149B1 |
| ILF3 | ENSG00000156515 | HK1 |
| ILF3 | ENSG00000197149 | RP11-452G18.2 |
| ILF3 | ENSG00000268107 | AC003005.4 |
| ILF3 | ENSG00000131116 | ZNF428 |
| ILF3 | ENSG00000103126 | AXIN1 |
| ILF3 | ENSG00000100307 | CBX7 |
| ILF3 | ENSG00000197903 | HIST1H2BK |
| ILF3 | ENSG00000106367 | AP1S1 |
| ILF3 | ENSG00000197442 | MAP3K5 |
| ILF3 | ENSG00000131055 | COX4I2 |
| ILF3 | ENSG00000249572 | CTD-2203K17.1 |
| ILF3 | ENSG00000174600 | CMKLR1 |
| ILF3 | ENSG00000112511 | PHF1 |
| ILF3 | ENSG00000262154 | EIF1P4 |
| ILF3 | ENSG00000168116 | KIAA1586 |
| ILF3 | ENSG00000183291 | 15-Sep |
| ILF3 | ENSG00000230835 | AP001187.11 |
| ILF3 | ENSG00000171223 | JUNB |
| ILF3 | ENSG00000064309 | CDON |
| ILF3 | ENSG00000227354 | RBM26-AS1 |
| ILF3 | ENSG00000187109 | NAP1L1 |
| ILF3 | ENSG00000128833 | MYO5C |
| ILF3 | ENSG00000238709 | RNU7-56P |
| ILF3 | ENSG00000114120 | SLC25A36 |
| ILF3 | ENSG00000227406 | RNF6P1 |
| ILF3 | ENSG00000233893 | EZR-AS1 |
| ILF3 | ENSG00000119541 | VPS4B |
| ILF3 | ENSG00000102384 | CENPI |
| ILF3 | ENSG00000225778 | PROSER2-AS1 |
| ILF3 | ENSG00000204899 | MZT1 |
| ILF3 | ENSG00000114902 | SPCS1 |
| ILF3 | ENSG00000139436 | GIT2 |
| ILF3 | ENSG00000164880 | INTS1 |
| ILF3 | ENSG00000092068 | SLC7A8 |
| ILF3 | ENSG00000255158 | RP11-754B17.1 |
| ILF3 | ENSG00000278200 | RP13-766D20.4 |
| ILF3 | ENSG00000099821 | POLRMT |
| ILF3 | ENSG00000085644 | ZNF213 |
| ILF3 | ENSG00000207343 | RNU6-225P |
| ILF3 | ENSG00000159063 | ALG8 |
| ILF3 | ENSG00000228157 | AC007952.5 |

| ILF3 | ENSG00000121064 | SCPEP1 |
| --- | --- | --- |
| ILF3 | ENSG00000270933 | CTD-2227E11.1 |
| ILF3 | ENSG00000204387 | C6orf48 |
| ILF3 | ENSG00000265073 | AC010761.6 |
| ILF3 | ENSG00000124019 | FAM124B |
| ILF3 | ENSG00000262692 | CTD-3195I5.3 |
| ILF3 | ENSG00000271335 | RP11-324I22.4 |
| ILF3 | ENSG00000111639 | MRPL51 |
| ILF3 | ENSG00000233977 | RP11-310H4.2 |
| ILF3 | ENSG00000270136 | MINOS1-NBL1 |
| ILF3 | ENSG00000100376 | FAM118A |
| ILF3 | ENSG00000168916 | ZNF608 |
| ILF3 | ENSG00000059573 | ALDH18A1 |
| ILF3 | ENSG00000244998 | CTD-3064M3.4 |
| ILF3 | ENSG00000174886 | NDUFA11 |
| ILF3 | ENSG00000260145 | RP11-322D14.2 |
| ILF3 | ENSG00000224447 | HIST1H1PS2 |
| ILF3 | ENSG00000146826 | C7orf43 |
| ILF3 | ENSG00000103657 | HERC1 |
| ILF3 | ENSG00000155115 | GTF3C6 |
| ILF3 | ENSG00000257122 | RRN3P3 |
| ILF3 | ENSG00000258021 | RP11-1100L3.4 |
| ILF3 | ENSG00000206814 | Y_RNA |
| ILF3 | ENSG00000144591 | GMPPA |
| ILF3 | ENSG00000104047 | DTWD1 |
| ILF3 | ENSG00000179855 | GIPC3 |
| ILF3 | ENSG00000244619 | RP11-315I20.3 |
| ILF3 | ENSG00000090889 | KIF4A |
| ILF3 | ENSG00000260879 | RP11-483I13.5 |
| ILF3 | ENSG00000229809 | ZNF688 |
| ILF3 | ENSG00000259856 | RAB43P1 |
| ILF3 | ENSG00000166851 | PLK1 |
| ILF3 | ENSG00000271947 | RP11-439M11.1 |
| ILF3 | ENSG00000264235 | RP13-270P17.1 |
| ILF3 | ENSG00000173744 | AGFG1 |
| ILF3 | ENSG00000226377 | AC084809.2 |
| ILF3 | ENSG00000166589 | CDH16 |
| ILF3 | ENSG00000224431 | AC063976.7 |
| ILF3 | ENSG00000183323 | CCDC125 |
| ILF3 | ENSG00000235072 | AC012074.2 |
| ILF3 | ENSG00000102543 | CDADC1 |
| ILF3 | ENSG00000207024 | Y_RNA |
| ILF3 | ENSG00000184524 | CEND1 |
| ILF3 | ENSG00000275371 | RP11-455F5.6 |
| ILF3 | ENSG00000269177 | L34079.3 |
| ILF3 | ENSG00000272070 | AC005618.6 |
| ILF3 | ENSG00000279227 | AC009303.2 |
| ILF3 | ENSG00000138050 | THUMPD2 |
| ILF3 | ENSG00000051180 | RAD51 |
| ILF3 | ENSG00000060566 | CREB3L3 |
| ILF3 | ENSG00000260179 | RP5-902P8.12 |

| ILF3 | ENSG00000204577 | LILRB3 |
| --- | --- | --- |
| ILF3 | ENSG00000125531 | C20orf195 |
| ILF3 | ENSG00000140955 | ADAD2 |
| ILF3 | ENSG00000253172 | CTC-209H22.3 |
| ILF3 | ENSG00000100029 | PES1 |
| ILF3 | ENSG00000084731 | KIF3C |
| ILF3 | ENSG00000125457 | MIF4GD |
| ILF3 | ENSG00000165698 | C9orf9 |
| ILF3 | ENSG00000261026 | CTD-3247F14.2 |
| ILF3 | ENSG00000170458 | CD14 |
| ILF3 | ENSG00000270393 | AC000095.9 |
| ILF3 | ENSG00000275975 | Metazoa_SRP |
| ILF3 | ENSG00000155256 | ZFYVE27 |
| ILF3 | ENSG00000278725 | RP11-666O2.5 |
| ILF3 | ENSG00000204439 | C6orf47 |
| ILF3 | ENSG00000111850 | SMIM8 |
| ILF3 | ENSG00000251432 | RP11-420A23.1 |
| ILF3 | ENSG00000064489 | MEF2BNB- |
| ILF3 | ENSG00000163938 | MEF2B GNL3 |
| ILF3 | ENSG00000204248 | COL11A2 |
| ILF3 | ENSG00000254862 | RP11-159H22.2 |
| ILF3 | ENSG00000277188 | NCRUPAR_1 |
| ILF3 | ENSG00000222849 | RNA5SP21 |
| ILF3 | ENSG00000204929 | AC074391.1 |
| ILF3 | ENSG00000167701 | GPT |
| ILF3 | ENSG00000012963 | UBR7 |
| ILF3 | ENSG00000267663 | RP11-64C12.9 |
| ILF3 | ENSG00000130749 | ZC3H4 |
| ILF3 | ENSG00000142156 | COL6A1 |
| ILF3 | ENSG00000105722 | ERF |
| ILF3 | ENSG00000130813 | C19orf66 |
| ILF3 | ENSG00000230286 | AC013472.4 |
| ILF3 | ENSG00000137413 | TAF8 |
| ILF3 | ENSG00000172775 | FAM192A |
| ILF3 | ENSG00000007376 | RPUSD1 |
| ILF3 | ENSG00000167130 | DOLPP1 |
| ILF3 | ENSG00000239356 | RN7SL309P |
| ILF3 | ENSG00000160310 | PRMT2 |
| ILF3 | ENSG00000128989 | ARPP19 |
| ILF3 | ENSG00000255173 | AP003068.12 |
| ILF3 | ENSG00000234134 | RP11-383C5.5 |
| ILF3 | ENSG00000261441 | RP11-217B1.2 |
| ILF3 | ENSG00000222345 | SNORD19 |
| ILF3 | ENSG00000177721 | ANXA2R |
| ILF3 | ENSG00000131697 | NPHP4 |
| ILF3 | ENSG00000278073 | MIR6726 |
| ILF3 | ENSG00000124116 | WFDC3 |
| ILF3 | ENSG00000266962 | RP11-400F19.6 |
| ILF3 | ENSG00000229531 | RP1-102G20.5 |
| ILF3 | ENSG00000227704 | RP1-266L20.4 |
| ILF3 | ENSG00000261403 | RP11-114H24.3 |

| ILF3 | ENSG00000131069 | ACSS2 |
| --- | --- | --- |
| ILF3 | ENSG00000199038 | MIR210 |
| ILF3 | ENSG00000275549 | C9orf173-AS1 |
| ILF3 | ENSG00000101811 | CSTF2 |
| ILF3 | ENSG00000096401 | CDC5L |
| ILF3 | ENSG00000140743 | CDR2 |
| ILF3 | ENSG00000253559 | OSGEPL1-AS1 |
| ILF3 | ENSG00000168393 | DTYMK |
| ILF3 | ENSG00000267319 | CTD-2528L19.3 |
| ILF3 | ENSG00000197933 | ZNF823 |
| ILF3 | ENSG00000139372 | TDG |
| ILF3 | ENSG00000158022 | TRIM63 |
| ILF3 | ENSG00000271882 | KB-1410C5.5 |
| ILF3 | ENSG00000238045 | AC009133.12 |
| ILF3 | ENSG00000167615 | LENG8 |
| ILF3 | ENSG00000268895 | A1BG-AS1 |
| ILF3 | ENSG00000215067 | ALOX12-AS1 |
| ILF3 | ENSG00000107372 | ZFAND5 |
| ILF3 | ENSG00000145194 | ECE2 |
| ILF3 | ENSG00000128891 | C15orf57 |
| ILF3 | ENSG00000267604 | RP5-905N1.2 |
| ILF3 | ENSG00000139899 | CBLN3 |
| ILF3 | ENSG00000166582 | CENPV |
| ILF3 | ENSG00000252341 | Y_RNA |
| ILF3 | ENSG00000222924 | RNU6-1148P |
| ILF3 | ENSG00000173674 | EIF1AX |
| ILF3 | ENSG00000079785 | DDX1 |
| ILF3 | ENSG00000062598 | ELMO2 |
| ILF3 | ENSG00000050344 | NFE2L3 |
| ILF3 | ENSG00000201823 | SNORD48 |
| ILF3 | ENSG00000011426 | ANLN |
| ILF3 | ENSG00000276597 | TRBV11-3 |
| ILF3 | ENSG00000067900 | ROCK1 |
| ILF3 | ENSG00000160703 | NLRX1 |
| ILF3 | ENSG00000068903 | SIRT2 |
| ILF3 | ENSG00000237004 | ZNRF2P1 |
| ILF3 | ENSG00000226510 | UPK1A-AS1 |
| ILF3 | ENSG00000135437 | RDH5 |
| ILF3 | ENSG00000125691 | RPL23 |
| ILF3 | ENSG00000226281 | RP1-80N2.2 |
| ILF3 | ENSG00000264968 | RP11-387H17.4 |
| ILF3 | ENSG00000186074 | CD300LF |
| ILF3 | ENSG00000166183 | ASPG |
| ILF3 | ENSG00000151176 | PLBD2 |
| ILF3 | ENSG00000236206 | RP11-306I1.2 |
| ILF3 | ENSG00000112983 | BRD8 |
| ILF3 | ENSG00000267234 | CTB-184G21.3 |
| ILF3 | ENSG00000137210 | TMEM14B |
| ILF3 | ENSG00000172167 | MTBP |
| ILF3 | ENSG00000272235 | RP11-22L13.1 |
| ILF3 | ENSG00000180953 | ST20 |

| ILF3 | ENSG00000250584 | LINC01511 |
| --- | --- | --- |
| ILF3 | ENSG00000159173 | TNNI1 |
| ILF3 | ENSG00000225063 | RP11-383G10.5 |
| ILF3 | ENSG00000254876 | RP11-23J9.5 |
| ILF3 | ENSG00000159259 | CHAF1B |
| ILF3 | ENSG00000236432 | AC097662.2 |
| ILF3 | ENSG00000267666 | AC004156.3 |
| ILF3 | ENSG00000119640 | ACYP1 |
| ILF3 | ENSG00000218281 | HIST1H2APS3 |
| ILF3 | ENSG00000239219 | RP11-379K17.4 |
| ILF3 | ENSG00000207708 | MIR141 |
| ILF3 | ENSG00000279001 | RP11-216P16.5 |
| ILF3 | ENSG00000151131 | C12orf45 |
| ILF3 | ENSG00000205356 | TECPR1 |
| ILF3 | ENSG00000111666 | CHPT1 |
| ILF3 | ENSG00000274104 | CTD-2553L13.10 |
| ILF3 | ENSG00000182310 | SPACA6P |
| ILF3 | ENSG00000143412 | ANXA9 |
| ILF3 | ENSG00000009307 | CSDE1 |
| ILF3 | ENSG00000113384 | GOLPH3 |
| ILF3 | ENSG00000152193 | RNF219 |
| ILF3 | ENSG00000264810 | MIR4441 |
| ILF3 | ENSG00000116096 | SPR |
| ILF3 | ENSG00000260851 | RP11-403P17.5 |
| ILF3 | ENSG00000113719 | ERGIC1 |
| ILF3 | ENSG00000065675 | PRKCQ |
| ILF3 | ENSG00000124224 | PPP4R1L |
| ILF3 | ENSG00000249962 | RP11-80H5.5 |
| ILF3 | ENSG00000269925 | RP3-467L1.6 |
| ILF3 | ENSG00000100416 | TRMU |
| ILF3 | ENSG00000230881 | RP11-486B10.3 |
| ILF3 | ENSG00000101986 | ABCD1 |
| ILF3 | ENSG00000186143 | PRR30 |
| ILF3 | ENSG00000048392 | RRM2B |
| ILF3 | ENSG00000276101 | RP11-455O6.8 |
| ILF3 | ENSG00000266758 | MIR3680-1 |
| ILF3 | ENSG00000187079 | TEAD1 |
| ILF3 | ENSG00000074855 | ANO8 |
| ILF3 | ENSG00000124767 | GLO1 |
| ILF3 | ENSG00000072958 | AP1M1 |
| ILF3 | ENSG00000090060 | PAPOLA |
| ILF3 | ENSG00000225578 | NCBP2-AS1 |
| ILF3 | ENSG00000089063 | TMEM230 |
| ILF3 | ENSG00000219375 | RP11-69L16.3 |
| ILF3 | ENSG00000229896 | RP11-456H18.2 |
| ILF3 | ENSG00000173418 | NAA20 |
| ILF3 | ENSG00000100351 | GRAP2 |
| ILF3 | ENSG00000143486 | EIF2D |
| ILF3 | ENSG00000264405 | MIR3913-1 |
| ILF3 | ENSG00000234521 | AC005041.11 |
| ILF3 | ENSG00000170801 | HTRA3 |

| ILF3 | ENSG00000096070 | BRPF3 |
| --- | --- | --- |
| ILF3 | ENSG00000279926 | RP3-406P24.5 |
| ILF3 | ENSG00000228084 | RP5-884G6.2 |
| ILF3 | ENSG00000136810 | TXN |
| ILF3 | ENSG00000237172 | B3GNT9 |
| ILF3 | ENSG00000090487 | SPG21 |
| ILF3 | ENSG00000180096 | SEPT1 |
| ILF3 | ENSG00000236430 | KRT8P29 |
| ILF3 | ENSG00000135077 | HAVCR2 |
| ILF3 | ENSG00000140105 | WARS |
| ILF3 | ENSG00000185033 | SEMA4B |
| ILF3 | ENSG00000279758 | RP11-752G15.10 |
| ILF3 | ENSG00000131238 | PPT1 |
| ILF3 | ENSG00000151304 | SRFBP1 |
| ILF3 | ENSG00000228019 | RP11-166O4.4 |
| ILF3 | ENSG00000103194 | USP10 |
| ILF3 | ENSG00000106638 | TBL2 |
| ILF3 | ENSG00000103064 | SLC7A6 |
| ILF3 | ENSG00000104133 | SPG11 |
| ILF3 | ENSG00000235354 | CTA-276O3.4 |
| ILF3 | ENSG00000172493 | AFF1 |
| ILF3 | ENSG00000265148 | BZRAP1-AS1 |
| ILF3 | ENSG00000103248 | MTHFSD |
| ILF3 | ENSG00000271780 | RP11-1017G21.5 |
| ILF3 | ENSG00000106100 | NOD1 |
| ILF3 | ENSG00000258297 | RP11-658F2.8 |
| ILF3 | ENSG00000102738 | MRPS31 |
| ILF3 | ENSG00000188643 | S100A16 |
| ILF3 | ENSG00000135913 | USP37 |
| ILF3 | ENSG00000166689 | PLEKHA7 |
| ILF3 | ENSG00000101216 | GMEB2 |
| ILF3 | ENSG00000198466 | ZNF587 |
| ILF3 | ENSG00000244705 | RP11-133K1.1 |
| ILF3 | ENSG00000166188 | ZNF319 |
| ILF3 | ENSG00000135517 | MIP |
| ILF3 | ENSG00000103266 | STUB1 |
| ILF3 | ENSG00000159884 | CCDC107 |
| ILF3 | ENSG00000065060 | UHRF1BP1 |
| ILF3 | ENSG00000042429 | MED17 |
| ILF3 | ENSG00000100218 | RSPH14 |
| ILF3 | ENSG00000166046 | TCP11L2 |
| ILF3 | ENSG00000173540 | GMPPB |
| ILF3 | ENSG00000174165 | ZDHHC24 |
| ILF3 | ENSG00000267106 | ZNF561-AS1 |
| ILF3 | ENSG00000172345 | STARD5 |
| ILF3 | ENSG00000221046 | RNU6ATAC38P |
| ILF3 | ENSG00000228903 | RASA4CP |
| ILF3 | ENSG00000128607 | KLHDC10 |
| ILF3 | ENSG00000254248 | RP11-320N21.2 |
| ILF3 | ENSG00000138190 | EXOC6 |
| ILF3 | ENSG00000267197 | CTC-429L19.3 |

| ILF3 | ENSG00000260007 | RP11-315D16.2 |
| --- | --- | --- |
| ILF3 | ENSG00000278737 | RP11-325L12.7 |
| ILF3 | ENSG00000252578 | RNU6-135P |
| ILF3 | ENSG00000262879 | RP11-156P1.3 |
| ILF3 | ENSG00000141741 | MIEN1 |
| ILF3 | ENSG00000235652 | RP11-545I5.3 |
| ILF3 | ENSG00000188825 | LINC00910 |
| ILF3 | ENSG00000143919 | CAMKMT |
| ILF3 | ENSG00000259784 | ATP6C |
| ILF3 | ENSG00000260630 | SNAI3-AS1 |
| ILF3 | ENSG00000213639 | PPP1CB |
| ILF3 | ENSG00000230519 | RP11-1060J15.5 |
| ILF3 | ENSG00000164062 | APEH |
| ILF3 | ENSG00000253276 | CCDC71L |
| ILF3 | ENSG00000140057 | AK7 |
| ILF3 | ENSG00000267607 | CTD-2369P2.8 |
| ILF3 | ENSG00000270382 | RP1-152L7.9 |
| ILF3 | ENSG00000183762 | KREMEN1 |
| ILF3 | ENSG00000221909 | FAM200A |
| ILF3 | ENSG00000276404 | MIR6835 |
| ILF3 | ENSG00000116663 | FBXO6 |
| ILF3 | ENSG00000105677 | TMEM147 |
| ILF3 | ENSG00000141736 | ERBB2 |
| ILF3 | ENSG00000223891 | OSER1-AS1 |
| ILF3 | ENSG00000124788 | ATXN1 |
| ILF3 | ENSG00000102886 | GDPD3 |
| ILF3 | ENSG00000274012 | Metazoa_SRP |
| ILF3 | ENSG00000134313 | KIDINS220 |
| ILF3 | ENSG00000002330 | BAD |
| ILF3 | ENSG00000134444 | KIAA1468 |
| ILF3 | ENSG00000156509 | FBXO43 |
| ILF3 | ENSG00000064545 | TMEM161A |
| ILF3 | ENSG00000118200 | CAMSAP2 |
| ILF3 | ENSG00000115761 | NOL10 |
| ILF3 | ENSG00000160055 | TMEM234 |
| ILF3 | ENSG00000168496 | FEN1 |
| ILF3 | ENSG00000183475 | ASB7 |
| ILF3 | ENSG00000237015 | CTA-984G1.5 |
| ILF3 | ENSG00000071553 | ATP6AP1 |
| ILF3 | ENSG00000148660 | CAMK2G |
| ILF3 | ENSG00000272144 | CTD-2035E11.5 |
| ILF3 | ENSG00000114779 | ABHD14B |
| ILF3 | ENSG00000164845 | FAM86FP |
| ILF3 | ENSG00000266240 | MIR5091 |
| ILF3 | ENSG00000145390 | USP53 |
| ILF3 | ENSG00000233757 | AC092835.2 |
| ILF3 | ENSG00000276727 | RP11-123K3.9 |
| ILF3 | ENSG00000132016 | C19orf57 |
| ILF3 | ENSG00000015285 | WAS |
| ILF3 | ENSG00000260441 | RP11-96D1.7 |
| ILF3 | ENSG00000234144 | COX6CP13 |

| ILF3 | ENSG00000239082 | RNU7-170P |
| --- | --- | --- |
| ILF3 | ENSG00000263063 | RP11-388C12.1 |
| ILF3 | ENSG00000011275 | RNF216 |
| ILF3 | ENSG00000151025 | GPR158 |
| ILF3 | ENSG00000198171 | DDRGK1 |
| ILF3 | ENSG00000212024 | MIR550A3 |
| ILF3 | ENSG00000255867 | DENND5B-AS1 |
| ILF3 | ENSG00000081870 | HSPB11 |
| ILF3 | ENSG00000125970 | RALY |
| ILF3 | ENSG00000201684 | RN7SKP239 |
| ILF3 | ENSG00000120254 | MTHFD1L |
| ILF3 | ENSG00000254458 | RP11-867G23.13 |
| ILF3 | ENSG00000185010 | F8 |
| ILF3 | ENSG00000138231 | DBR1 |
| ILF3 | ENSG00000082996 | RNF13 |
| ILF3 | ENSG00000087448 | KLHL42 |
| ILF3 | ENSG00000108551 | RASD1 |
| ILF3 | ENSG00000267634 | RPL7L1P5 |
| ILF3 | ENSG00000230188 | RP11-405L18.4 |
| ILF3 | ENSG00000132405 | TBC1D14 |
| ILF3 | ENSG00000089094 | KDM2B |
| ILF3 | ENSG00000261302 | RP11-343H19.1 |
| ILF3 | ENSG00000252866 | RNA5SP243 |
| ILF3 | ENSG00000258924 | CTB-96E2.3 |
| ILF3 | ENSG00000116747 | TROVE2 |
| ILF3 | ENSG00000167941 | SOST |
| ILF3 | ENSG00000140386 | SCAPER |
| ILF3 | ENSG00000258457 | RP11-298I3.4 |
| ILF3 | ENSG00000225854 | RP11-569G9.7 |
| ILF3 | ENSG00000110719 | TCIRG1 |
| ILF3 | ENSG00000162039 | MEIOB |
| ILF3 | ENSG00000178988 | MRFAP1L1 |
| ILF3 | ENSG00000105516 | DBP |
| ILF3 | ENSG00000245680 | ZNF585B |
| ILF3 | ENSG00000010072 | SPRTN |
| ILF3 | ENSG00000230583 | GTF2IRD1P1 |
| ILF3 | ENSG00000261346 | RP11-297C4.2 |
| ILF3 | ENSG00000120526 | NUDCD1 |
| ILF3 | ENSG00000223546 | LINC00630 |
| ILF3 | ENSG00000148943 | LIN7C |
| ILF3 | ENSG00000242338 | BMS1P4 |
| ILF3 | ENSG00000232388 | LINC00493 |
| ILF3 | ENSG00000009413 | REV3L |
| ILF3 | ENSG00000108953 | YWHAE |
| ILF3 | ENSG00000172534 | HCFC1 |
| ILF3 | ENSG00000261758 | RP11-102M11.2 |
| ILF3 | ENSG00000279945 | RP11-97O12.5 |
| ILF3 | ENSG00000204410 | MSH5 |
| ILF3 | ENSG00000172081 | MOB3A |
| ILF3 | ENSG00000043514 | TRIT1 |
| ILF3 | ENSG00000255164 | RP5-1047A19.4 |

| ILF3 | ENSG00000080823 | MOK |
| --- | --- | --- |
| ILF3 | ENSG00000143554 | SLC27A3 |
| ILF3 | ENSG00000169762 | TAPT1 |
| ILF3 | ENSG00000140463 | BBS4 |
| ILF3 | ENSG00000232746 | RP11-767C1.1 |
| ILF3 | ENSG00000137193 | PIM1 |
| ILF3 | ENSG00000109846 | CRYAB |
| ILF3 | ENSG00000269997 | RP11-214K3.21 |
| ILF3 | ENSG00000260081 | RP11-66N11.8 |
| ILF3 | ENSG00000264947 | hsa-mir-3181 |
| ILF3 | ENSG00000221886 | ZBED8 |
| ILF3 | ENSG00000162545 | CAMK2N1 |
| ILF3 | ENSG00000171860 | C3AR1 |
| ILF3 | ENSG00000131165 | CHMP1A |
| ILF3 | ENSG00000229827 | AC093899.3 |
| ILF3 | ENSG00000179041 | RRS1 |
| ILF3 | ENSG00000124380 | SNRNP27 |
| ILF3 | ENSG00000230758 | SNAP23P |
| ILF3 | ENSG00000198399 | ITSN2 |
| ILF3 | ENSG00000227252 | AC105760.2 |
| ILF3 | ENSG00000224082 | UBTFL8 |
| ILF3 | ENSG00000231149 | RP11-10J18.3 |
| ILF3 | ENSG00000167468 | GPX4 |
| ILF3 | ENSG00000204314 | PRRT1 |
| ILF3 | ENSG00000251211 | RP11-889L3.4 |
| ILF3 | ENSG00000134077 | THUMPD3 |
| ILF3 | ENSG00000162999 | DUSP19 |
| ILF3 | ENSG00000260465 | RP11-63M22.2 |
| ILF3 | ENSG00000277149 | TYW1B |
| ILF3 | ENSG00000110321 | EIF4G2 |
| ILF3 | ENSG00000221676 | RNU6ATAC |
| ILF3 | ENSG00000006118 | TMEM132A |
| ILF3 | ENSG00000162398 | C1orf177 |
| ILF3 | ENSG00000142453 | CARM1 |
| ILF3 | ENSG00000131653 | TRAF7 |
| ILF3 | ENSG00000270800 | RPS10-NUDT3 |
| ILF3 | ENSG00000237017 | AC012314.8 |
| ILF3 | ENSG00000105499 | PLA2G4C |
| ILF3 | ENSG00000277462 | ZNF670 |
| ILF3 | ENSG00000179361 | ARID3B |
| ILF3 | ENSG00000135503 | ACVR1B |
| ILF3 | ENSG00000078070 | MCCC1 |
| ILF3 | ENSG00000209645 | SNORD105 |
| ILF3 | ENSG00000171169 | NAIF1 |
| ILF3 | ENSG00000161980 | POLR3K |
| ILF3 | ENSG00000167641 | PPP1R14A |
| ILF3 | ENSG00000264438 | RN7SL560P |
| ILF3 | ENSG00000268983 | AC005253.4 |
| ILF3 | ENSG00000173905 | GOLIM4 |
| ILF3 | ENSG00000171295 | ZNF440 |
| ILF3 | ENSG00000196646 | ZNF136 |

| ILF3 | ENSG00000154710 | RABGEF1 |
| --- | --- | --- |
| ILF3 | ENSG00000254402 | LRRC24 |
| ILF3 | ENSG00000127838 | PNKD |
| ILF3 | ENSG00000228649 | AC005682.5 |
| ILF3 | ENSG00000200903 | RNU1-42P |
| ILF3 | ENSG00000204385 | SLC44A4 |
| ILF3 | ENSG00000152332 | UHMK1 |
| ILF3 | ENSG00000177337 | DLGAP1-AS1 |
| ILF3 | ENSG00000188372 | ZP3 |
| ILF3 | ENSG00000100129 | EIF3L |
| ILF3 | ENSG00000044115 | CTNNA1 |
| ILF3 | ENSG00000226266 | AC009961.3 |
| ILF3 | ENSG00000279645 | RP11-759A24.1 |
| ILF3 | ENSG00000144048 | DUSP11 |
| ILF3 | ENSG00000140396 | NCOA2 |
| ILF3 | ENSG00000197841 | ZNF181 |
| ILF3 | ENSG00000104946 | TBC1D17 |
| ILF3 | ENSG00000278272 | HIST1H3C |
| ILF3 | ENSG00000266203 | MIR5585 |
| ILF3 | ENSG00000260231 | JHDM1D-AS1 |
| ILF3 | ENSG00000260219 | RP11-347C12.10 |
| ILF3 | ENSG00000185324 | CDK10 |
| ILF3 | ENSG00000145757 | SPATA9 |
| ILF3 | ENSG00000112039 | FANCE |
| ILF3 | ENSG00000136280 | CCM2 |
| ILF3 | ENSG00000073849 | ST6GAL1 |
| ILF3 | ENSG00000111011 | RSRC2 |
| ILF3 | ENSG00000243378 | RP11-646E18.3 |
| ILF3 | ENSG00000229140 | CCDC26 |
| ILF3 | ENSG00000143768 | LEFTY2 |
| ILF3 | ENSG00000177963 | RIC8A |
| ILF3 | ENSG00000123975 | CKS2 |
| ILF3 | ENSG00000270798 | RP11-416H1.1 |
| ILF3 | ENSG00000149735 | GPHA2 |
| ILF3 | ENSG00000100350 | FOXRED2 |
| ILF3 | ENSG00000154839 | SKA1 |
| ILF3 | ENSG00000143436 | MRPL9 |
| ILF3 | ENSG00000203879 | GDI1 |
| ILF3 | ENSG00000136504 | KAT7 |
| ILF3 | ENSG00000181135 | ZNF707 |
| ILF3 | ENSG00000257453 | RP11-290L1.3 |
| ILF3 | ENSG00000271978 | RP11-428J1.4 |
| ILF3 | ENSG00000255185 | PDXDC2P |
| ILF3 | ENSG00000263585 | RP11-498C9.13 |
| ILF3 | ENSG00000075539 | FRYL |
| ILF3 | ENSG00000160256 | FAM207A |
| ILF3 | ENSG00000166492 | FAM86GP |
| ILF3 | ENSG00000253426 | RP11-10A14.4 |
| ILF3 | ENSG00000106803 | SEC61B |
| ILF3 | ENSG00000104973 | MED25 |
| ILF3 | ENSG00000273899 | NOL12 |

| ILF3 | ENSG00000267699 | RP11-729L2.2 |
| --- | --- | --- |
| ILF3 | ENSG00000256569 | RP11-173P15.5 |
| ILF3 | ENSG00000168487 | BMP1 |
| ILF3 | ENSG00000120438 | TCP1 |
| ILF3 | ENSG00000258377 | RP11-649E7.5 |
| ILF3 | ENSG00000237452 | BHMG1 |
| ILF3 | ENSG00000114767 | RRP9 |
| ILF3 | ENSG00000168350 | DEGS2 |
| ILF3 | ENSG00000259441 | MRPL15P1 |
| ILF3 | ENSG00000264563 | MIR4633 |
| ILF3 | ENSG00000216718 | RP3-522P13.1 |
| ILF3 | ENSG00000266086 | RP11-159D12.5 |
| ILF3 | ENSG00000135842 | FAM129A |
| ILF3 | ENSG00000252339 | RNU6-1061P |
| ILF3 | ENSG00000167770 | OTUB1 |
| ILF3 | ENSG00000004534 | RBM6 |
| ILF3 | ENSG00000111229 | ARPC3 |
| ILF3 | ENSG00000259869 | AL022344.7 |
| ILF3 | ENSG00000136231 | IGF2BP3 |
| ILF3 | ENSG00000254341 | SNORD87 |
| ILF3 | ENSG00000177675 | CD163L1 |
| ILF3 | ENSG00000188280 | FAM230A |
| ILF3 | ENSG00000229102 | RP11-360P21.2 |
| ILF3 | ENSG00000273186 | RP11-339B21.10 |
| ILF3 | ENSG00000171940 | ZNF217 |
| ILF3 | ENSG00000263809 | KRBA2 |
| ILF3 | ENSG00000211580 | MIR769 |
| ILF3 | ENSG00000245573 | BDNF-AS |
| ILF3 | ENSG00000171566 | PLRG1 |
| ILF3 | ENSG00000159082 | SYNJ1 |
| ILF3 | ENSG00000260001 | TGFBR3L |
| ILF3 | ENSG00000107949 | BCCIP |
| ILF3 | ENSG00000126088 | UROD |
| ILF3 | ENSG00000154783 | FGD5 |
| ILF3 | ENSG00000111678 | C12orf57 |
| ILF3 | ENSG00000266983 | CTC-232P5.1 |
| ILF3 | ENSG00000228878 | SEPT7-AS1 |
| ILF3 | ENSG00000228172 | RP1-317E23.3 |
| ILF3 | ENSG00000107831 | FGF8 |
| ILF3 | ENSG00000267543 | RP11-666A8.7 |
| ILF3 | ENSG00000233056 | ERVH48-1 |
| ILF3 | ENSG00000254826 | CTD-2530H12.2 |
| ILF3 | ENSG00000117362 | APH1A |
| ILF3 | ENSG00000224680 | PLA2G12AP1 |
| ILF3 | ENSG00000119616 | FCF1 |
| ILF3 | ENSG00000277027 | RNase_MRP |
| ILF3 | ENSG00000257588 | RP11-469H8.6 |
| ILF3 | ENSG00000143195 | ILDR2 |
| ILF3 | ENSG00000271913 | RP1-111C20.4 |
| ILF3 | ENSG00000066279 | ASPM |
| ILF3 | ENSG00000232202 | AC098824.6 |

| ILF3 | ENSG00000135317 | SNX14 |
| --- | --- | --- |
| ILF3 | ENSG00000172725 | CORO1B |
| ILF3 | ENSG00000135945 | REV1 |
| ILF3 | ENSG00000264573 | RN7SL15P |
| ILF3 | ENSG00000239468 | RN7SL569P |
| ILF3 | ENSG00000270659 | RP11-105N14.1 |
| ILF3 | ENSG00000116473 | RAP1A |
| ILF3 | ENSG00000077327 | SPAG6 |
| ILF3 | ENSG00000148396 | SEC16A |
| ILF3 | ENSG00000167106 | FAM102A |
| ILF3 | ENSG00000264229 | RNU4ATAC |
| ILF3 | ENSG00000225336 | HMGB3P1 |
| ILF3 | ENSG00000130816 | DNMT1 |
| ILF3 | ENSG00000232098 | CTD-2619J13.14 |
| ILF3 | ENSG00000273432 | RP5-1165K10.2 |
| ILF3 | ENSG00000103852 | TTC23 |
| ILF3 | ENSG00000208008 | MIR125A |
| ILF3 | ENSG00000161939 | RNASEK- |
| ILF3 | ENSG00000254631 | C17orf49  RP11-702H23.4 |
| ILF3 | ENSG00000174945 | AMZ1 |
| ILF3 | ENSG00000002549 | LAP3 |
| ILF3 | ENSG00000269445 | AC067969.1 |
| ILF3 | ENSG00000184209 | SNRNP35 |
| ILF3 | ENSG00000126522 | ASL |
| ILF3 | ENSG00000142634 | EFHD2 |
| ILF3 | ENSG00000233797 | UFL1-AS1 |
| ILF3 | ENSG00000142686 | C1orf216 |
| ILF3 | ENSG00000196950 | SLC39A10 |
| ILF3 | ENSG00000271779 | RP11-87N3.6 |
| ILF3 | ENSG00000230555 | RP11-517P14.2 |
| ILF3 | ENSG00000172602 | RND1 |
| ILF3 | ENSG00000220949 | CTC-499B15.1 |
| ILF3 | ENSG00000204282 | TNRC6C-AS1 |
| ILF3 | ENSG00000243224 | RP5-1157M23.2 |
| ILF3 | ENSG00000253102 | RP1-117B12.4 |
| ILF3 | ENSG00000160972 | PPP1R16A |
| ILF3 | ENSG00000260249 | RP11-401P9.5 |
| ILF3 | ENSG00000201725 | RNU6-304P |
| ILF3 | ENSG00000168763 | CNNM3 |
| ILF3 | ENSG00000204564 | C6orf136 |
| ILF3 | ENSG00000181222 | POLR2A |
| ILF3 | ENSG00000263409 | MIR4747 |
| ILF3 | ENSG00000231160 | KLF3-AS1 |
| ILF3 | ENSG00000211581 | MIR765 |
| ILF3 | ENSG00000108306 | FBXL20 |
| ILF3 | ENSG00000141401 | IMPA2 |
| ILF3 | ENSG00000278376 | RP11-158I9.8 |
| ILF3 | ENSG00000100994 | PYGB |
| ILF3 | ENSG00000171208 | NETO2 |
| ILF3 | ENSG00000127399 | LRRC61 |
| ILF3 | ENSG00000272301 | RP11-111M22.4 |

| ILF3 | ENSG00000236677 | AP000688.11 |
| --- | --- | --- |
| ILF3 | ENSG00000279063 | CTD-2537I9.15 |
| ILF3 | ENSG00000275293 | Metazoa_SRP |
| ILF3 | ENSG00000007168 | PAFAH1B1 |
| ILF3 | ENSG00000186815 | TPCN1 |
| ILF3 | ENSG00000234983 | AC010132.11 |
| ILF3 | ENSG00000187601 | MAGEH1 |
| ILF3 | ENSG00000156030 | ELMSAN1 |
| ILF3 | ENSG00000226782 | RP11-706D8.3 |
| ILF3 | ENSG00000089009 | RPL6 |
| ILF3 | ENSG00000250240 | CTD-2154I11.2 |
| ILF3 | ENSG00000163257 | DCAF16 |
| ILF3 | ENSG00000213246 | SUPT4H1 |
| ILF3 | ENSG00000224451 | ATP5F1P1 |
| ILF3 | ENSG00000189190 | ZNF600 |
| ILF3 | ENSG00000207752 | MIR199A1 |
| ILF3 | ENSG00000214401 | KANSL1-AS1 |
| ILF3 | ENSG00000120860 | CCDC53 |
| ILF3 | ENSG00000164576 | SAP30L |
| ILF3 | ENSG00000129562 | DAD1 |
| ILF3 | ENSG00000167325 | RRM1 |
| ILF3 | ENSG00000078142 | PIK3C3 |
| ILF3 | ENSG00000272647 | GS1-259H13.10 |
| ILF3 | ENSG00000184414 | RP11-44M6.3 |
| ILF3 | ENSG00000176809 | LRRC37A3 |
| ILF3 | ENSG00000134970 | TMED7 |
| ILF3 | ENSG00000154727 | GABPA |
| ILF3 | ENSG00000116983 | HPCAL4 |
| ILF3 | ENSG00000165637 | VDAC2 |
| ILF3 | ENSG00000213977 | TAX1BP3 |
| ILF3 | ENSG00000164104 | HMGB2 |
| ILF3 | ENSG00000111711 | GOLT1B |
| ILF3 | ENSG00000168137 | SETD5 |
| ILF3 | ENSG00000171302 | CANT1 |
| ILF3 | ENSG00000274693 | HTT-AS1_2 |
| ILF3 | ENSG00000232630 | PRPS1P2 |
| ILF3 | ENSG00000255182 | CTD- |
| ILF3 | ENSG00000113312 | 2517M22.14  TTC1 |
| ILF3 | ENSG00000260919 | CTD-3154N5.1 |
| ILF3 | ENSG00000253256 | CTB-11I22.1 |
| ILF3 | ENSG00000176407 | KCMF1 |
| ILF3 | ENSG00000207416 | Y_RNA |
| ILF3 | ENSG00000260572 | RP11-16N11.2 |
| ILF3 | ENSG00000203279 | RP11-498P14.5 |
| ILF3 | ENSG00000149636 | DSN1 |
| ILF3 | ENSG00000144034 | TPRKB |
| ILF3 | ENSG00000132357 | CARD6 |
| ILF3 | ENSG00000110958 | PTGES3 |
| ILF3 | ENSG00000238199 | UBE2V2P3 |
| ILF3 | ENSG00000262921 | RP11-141J13.3 |
| ILF3 | ENSG00000173207 | CKS1B |

| ILF3 | ENSG00000199476 | Y_RNA |
| --- | --- | --- |
| ILF3 | ENSG00000039123 | SKIV2L2 |
| ILF3 | ENSG00000275663 | HIST1H4G |
| ILF3 | ENSG00000277502 | uc_338 |
| ILF3 | ENSG00000266114 | RP11-963H4.5 |
| ILF3 | ENSG00000188766 | SPRED3 |
| ILF3 | ENSG00000263126 | CTC-479C5.10 |
| ILF3 | ENSG00000149260 | CAPN5 |
| ILF3 | ENSG00000205076 | LGALS7 |
| ILF3 | ENSG00000225711 | RP11-345I18.4 |
| ILF3 | ENSG00000181873 | IBA57 |
| ILF3 | ENSG00000125991 | ERGIC3 |
| ILF3 | ENSG00000145388 | METTL14 |
| ILF3 | ENSG00000230432 | AC114803.3 |
| ILF3 | ENSG00000276353 | Metazoa_SRP |
| ILF3 | ENSG00000145191 | EIF2B5 |
| ILF3 | ENSG00000110851 | PRDM4 |
| ILF3 | ENSG00000271973 | RP11-572O6.1 |
| ILF3 | ENSG00000247363 | RP11-637A17.2 |
| ILF3 | ENSG00000270757 | HSPE1-MOB4 |
| ILF3 | ENSG00000198707 | CEP290 |
| ILF3 | ENSG00000232303 | DFFBP1 |
| ILF3 | ENSG00000250656 | ST3GAL1P1 |
| ILF3 | ENSG00000264317 | RN7SL154P |
| ILF3 | ENSG00000241973 | PI4KA |
| ILF3 | ENSG00000068885 | IFT80 |
| ILF3 | ENSG00000200998 | Y_RNA |
| ILF3 | ENSG00000249971 | RP11-256P1.1 |
| ILF3 | ENSG00000160226 | C21orf2 |
| ILF3 | ENSG00000116649 | SRM |
| ILF3 | ENSG00000234690 | AC073283.4 |
| ILF3 | ENSG00000277588 | MIR6806 |
| ILF3 | ENSG00000232518 | AC074366.3 |
| ILF3 | ENSG00000138801 | PAPSS1 |
| ILF3 | ENSG00000271848 | RP11-464F9.21 |
| ILF3 | ENSG00000100241 | SBF1 |
| ILF3 | ENSG00000279619 | CTD-2527I21.5 |
| ILF3 | ENSG00000164535 | DAGLB |
| ILF3 | ENSG00000115255 | REEP6 |
| ILF3 | ENSG00000267232 | CTB-31O20.9 |
| ILF3 | ENSG00000221340 | RNU6ATAC18 |
| ILF3 | ENSG00000222421 | P  Y_RNA |
| ILF3 | ENSG00000263993 | RN7SL786P |
| ILF3 | ENSG00000158079 | PTPDC1 |
| ILF3 | ENSG00000105255 | FSD1 |
| ILF3 | ENSG00000158864 | NDUFS2 |
| ILF3 | ENSG00000227857 | RP4-533D7.5 |
| ILF3 | ENSG00000131187 | F12 |
| ILF3 | ENSG00000266575 | RP11-672L10.4 |
| ILF3 | ENSG00000128602 | SMO |
| ILF3 | ENSG00000221411 | MIR1227 |

| ILF3 | ENSG00000088387 | DOCK9 |
| --- | --- | --- |
| ILF3 | ENSG00000185716 | C16orf52 |
| ILF3 | ENSG00000118961 | C2orf43 |
| ILF3 | ENSG00000105186 | ANKRD27 |
| ILF3 | ENSG00000165916 | PSMC3 |
| ILF3 | ENSG00000088038 | CNOT3 |
| ILF3 | ENSG00000101997 | CCDC22 |
| ILF3 | ENSG00000186376 | ZNF75D |
| ILF3 | ENSG00000006652 | IFRD1 |
| ILF3 | ENSG00000119707 | RBM25 |
| ILF3 | ENSG00000150527 | CTAGE5 |
| ILF3 | ENSG00000241542 | RN7SL369P |
| ILF3 | ENSG00000240388 | CTD-2061E19.1 |
| ILF3 | ENSG00000230613 | HM13-AS1 |
| ILF3 | ENSG00000112242 | E2F3 |
| ILF3 | ENSG00000258938 | RP11-317N8.5 |
| ILF3 | ENSG00000250455 | RP11-648M2.1 |
| ILF3 | ENSG00000137770 | CTDSPL2 |
| ILF3 | ENSG00000260889 | CKBP1 |
| ILF3 | ENSG00000272172 | RP13-582O9.7 |
| ILF3 | ENSG00000272097 | RP11-421M1.8 |
| ILF3 | ENSG00000221491 | SNORA34 |
| ILF3 | ENSG00000203711 | C6orf99 |
| ILF3 | ENSG00000155760 | FZD7 |
| ILF3 | ENSG00000231394 | RP11-310H4.3 |
| ILF3 | ENSG00000231747 | AC079922.2 |
| ILF3 | ENSG00000151006 | PRSS53 |
| ILF3 | ENSG00000272945 | CTD-3216D2.5 |
| ILF3 | ENSG00000136715 | SAP130 |
| ILF3 | ENSG00000111615 | KRR1 |
| ILF3 | ENSG00000272279 | RP11-157J24.2 |
| ILF3 | ENSG00000168306 | ACOX2 |
| ILF3 | ENSG00000157216 | SSBP3 |
| ILF3 | ENSG00000130731 | C16orf13 |
| ILF3 | ENSG00000158292 | GPR153 |
| ILF3 | ENSG00000124251 | TP53TG5 |
| ILF3 | ENSG00000217527 | RPS16P5 |
| ILF3 | ENSG00000197056 | ZMYM1 |
| ILF3 | ENSG00000150967 | ABCB9 |
| ILF3 | ENSG00000128791 | TWSG1 |
| ILF3 | ENSG00000021762 | OSBPL5 |
| ILF3 | ENSG00000105767 | CADM4 |
| ILF3 | ENSG00000105397 | TYK2 |
| ILF3 | ENSG00000136942 | RPL35 |
| ILF3 | ENSG00000167548 | KMT2D |
| ILF3 | ENSG00000167178 | ISLR2 |
| ILF3 | ENSG00000179954 | SSC5D |
| ILF3 | ENSG00000167186 | COQ7 |
| ILF3 | ENSG00000231840 | AC073342.12 |
| ILF3 | ENSG00000072694 | FCGR2B |
| ILF3 | ENSG00000090020 | SLC9A1 |

| ILF3 | ENSG00000254814 | RP11-535A19.1 |
| --- | --- | --- |
| ILF3 | ENSG00000052841 | TTC17 |
| ILF3 | ENSG00000135932 | CAB39 |
| ILF3 | ENSG00000280419 | RP11-178L8.6 |
| ILF3 | ENSG00000269032 | AC016629.7 |
| ILF3 | ENSG00000135537 | LACE1 |
| ILF3 | ENSG00000050130 | JKAMP |
| ILF3 | ENSG00000100902 | PSMA6 |
| ILF3 | ENSG00000188886 | ASTL |
| ILF3 | ENSG00000167397 | VKORC1 |
| ILF3 | ENSG00000119950 | MXI1 |
| ILF3 | ENSG00000259645 | RP11-253M7.6 |
| ILF3 | ENSG00000160460 | SPTBN4 |
| ILF3 | ENSG00000186364 | NUDT17 |
| ILF3 | ENSG00000266967 | AARSD1 |
| ILF3 | ENSG00000065613 | SLK |
| ILF3 | ENSG00000117461 | PIK3R3 |
| ILF3 | ENSG00000263969 | RN7SL678P |
| ILF3 | ENSG00000196866 | HIST1H2AD |
| ILF3 | ENSG00000258561 | RP11-72M17.1 |
| ILF3 | ENSG00000224535 | RP11-61J19.2 |
| ILF3 | ENSG00000261821 | CTD- |
| ILF3 | ENSG00000225246 | 2311M21.3  RPS2P1 |
| ILF3 | ENSG00000058668 | ATP2B4 |
| ILF3 | ENSG00000173728 | C1orf100 |
| ILF3 | ENSG00000170946 | DNAJC24 |
| ILF3 | ENSG00000072818 | ACAP1 |
| ILF3 | ENSG00000163785 | RYK |
| ILF3 | ENSG00000223959 | AFG3L1P |
| ILF3 | ENSG00000131037 | EPS8L1 |
| ILF3 | ENSG00000173166 | RAPH1 |
| ILF3 | ENSG00000065057 | NTHL1 |
| ILF3 | ENSG00000260404 | RP11-384K6.6 |
| ILF3 | ENSG00000253603 | CTA-397H3.3 |
| ILF3 | ENSG00000228596 | AC013436.6 |
| ILF3 | ENSG00000131373 | HACL1 |
| ILF3 | ENSG00000159363 | ATP13A2 |
| ILF3 | ENSG00000104371 | DKK4 |
| ILF3 | ENSG00000142002 | DPP9 |
| ILF3 | ENSG00000166441 | RPL27A |
| ILF3 | ENSG00000269814 | CTC-273B12.10 |
| ILF3 | ENSG00000124193 | SRSF6 |
| ILF3 | ENSG00000221947 | XKR9 |
| ILF3 | ENSG00000203288 | RP11-98D18.9 |
| ILF3 | ENSG00000077454 | LRCH4 |
| ILF3 | ENSG00000102871 | TRADD |
| ILF3 | ENSG00000167680 | SEMA6B |
| ILF3 | ENSG00000213213 | CCDC183 |
| ILF3 | ENSG00000275291 | U1 |
| ILF3 | ENSG00000105982 | RNF32 |
| ILF3 | ENSG00000143119 | CD53 |

| ILF3 | ENSG00000162520 | SYNC |
| --- | --- | --- |
| ILF3 | ENSG00000228737 | AC008781.7 |
| ILF3 | ENSG00000242553 | AP001432.14 |
| ILF3 | ENSG00000189050 | RNFT1 |
| ILF3 | ENSG00000242797 | GLYCTK-AS1 |
| ILF3 | ENSG00000201102 | Y_RNA |
| ILF3 | ENSG00000272686 | RP11-390E23.6 |
| ILF3 | ENSG00000066855 | MTFR1 |
| ILF3 | ENSG00000114030 | KPNA1 |
| ILF3 | ENSG00000152767 | FARP1 |
| ILF3 | ENSG00000207575 | MIR649 |
| ILF3 | ENSG00000110756 | HPS5 |
| ILF3 | ENSG00000186187 | ZNRF1 |
| ILF3 | ENSG00000197620 | CXorf40A |
| ILF3 | ENSG00000100578 | KIAA0586 |
| ILF3 | ENSG00000123200 | ZC3H13 |
| ILF3 | ENSG00000205885 | C1RL-AS1 |
| ILF3 | ENSG00000159723 | AGRP |
| ILF3 | ENSG00000100292 | HMOX1 |
| ILF3 | ENSG00000273443 | RP11-54O7.18 |
| ILF3 | ENSG00000244486 | SCARF2 |
| ILF3 | ENSG00000151917 | BEND6 |
| ILF3 | ENSG00000177725 | CTD-2530N21.5 |
| ILF3 | ENSG00000239122 | RNU2-11P |
| ILF3 | ENSG00000186919 | ZACN |
| ILF3 | ENSG00000255198 | SNHG9 |
| ILF3 | ENSG00000100479 | POLE2 |
| ILF3 | ENSG00000158882 | TOMM40L |
| ILF3 | ENSG00000250999 | RP11-1379J22.5 |
| ILF3 | ENSG00000224985 | RP11-297K8.2 |
| ILF3 | ENSG00000170584 | NUDCD2 |
| ILF3 | ENSG00000273898 | MIR6798 |
| ILF3 | ENSG00000248710 | RP11-432B6.3 |
| ILF3 | ENSG00000141933 | TPGS1 |
| ILF3 | ENSG00000217128 | FNIP1 |
| ILF3 | ENSG00000007001 | UPP2 |
| ILF3 | ENSG00000185697 | MYBL1 |
| ILF3 | ENSG00000119392 | GLE1 |
| ILF3 | ENSG00000228251 | AC012442.6 |
| ILF3 | ENSG00000104205 | SGK3 |
| ILF3 | ENSG00000102100 | SLC35A2 |
| ILF3 | ENSG00000245025 | RP11-875O11.1 |
| ILF3 | ENSG00000163960 | UBXN7 |
| ILF3 | ENSG00000120820 | GLT8D2 |
| ILF3 | ENSG00000202047 | RNA5SP324 |
| ILF3 | ENSG00000166888 | STAT6 |
| ILF3 | ENSG00000235189 | RP4-537K23.4 |
| ILF3 | ENSG00000187800 | PEAR1 |
| ILF3 | ENSG00000254905 | RP11-712L6.7 |
| ILF3 | ENSG00000122432 | SPATA1 |
| ILF3 | ENSG00000013561 | RNF14 |

| ILF3 | ENSG00000279255 | LA16c-380A1.2 |
| --- | --- | --- |
| ILF3 | ENSG00000115289 | PCGF1 |
| ILF3 | ENSG00000253194 | RP11-351A11.1 |
| ILF3 | ENSG00000272217 | XXbac- |
| ILF3 | ENSG00000141968 | BPG157A10.21  VAV1 |
| ILF3 | ENSG00000168000 | BSCL2 |
| ILF3 | ENSG00000122512 | PMS2 |
| ILF3 | ENSG00000270828 | RP3-466P17.2 |
| ILF3 | ENSG00000205309 | NT5M |
| ILF3 | ENSG00000116044 | NFE2L2 |
| ILF3 | ENSG00000205268 | PDE7A |
| ILF3 | ENSG00000129535 | NRL |
| ILF3 | ENSG00000157927 | RADIL |
| ILF3 | ENSG00000141446 | ESCO1 |
| ILF3 | ENSG00000173714 | WFIKKN2 |
| ILF3 | ENSG00000168283 | BMI1 |
| ILF3 | ENSG00000129515 | SNX6 |
| ILF3 | ENSG00000207293 | Y_RNA |
| ILF3 | ENSG00000113108 | APBB3 |
| ILF3 | ENSG00000269794 | AC010642.2 |
| ILF3 | ENSG00000165650 | PDZD8 |
| ILF3 | ENSG00000126003 | PLAGL2 |
| ILF3 | ENSG00000277604 | Y_RNA |
| ILF3 | ENSG00000176124 | DLEU1 |
| ILF3 | ENSG00000159593 | NAE1 |
| ILF3 | ENSG00000109184 | DCUN1D4 |
| ILF3 | ENSG00000188419 | CHM |
| ILF3 | ENSG00000115041 | KCNIP3 |
| ILF3 | ENSG00000158711 | ELK4 |
| ILF3 | ENSG00000128218 | VPREB3 |
| ILF3 | ENSG00000173546 | CSPG4 |
| ILF3 | ENSG00000278965 | RP11-39H3.2 |
| ILF3 | ENSG00000164896 | FASTK |
| ILF3 | ENSG00000254578 | CTD-2517M22.16 |
| ILF3 | ENSG00000112304 | ACOT13 |
| ILF3 | ENSG00000135315 | CEP162 |
| ILF3 | ENSG00000199350 | RNA5SP432 |
| ILF3 | ENSG00000258959 | RP11-1017G21.4 |
| ILF3 | ENSG00000267353 | CTD-2162K18.3 |
| ILF3 | ENSG00000155307 | SAMSN1 |
| ILF3 | ENSG00000207624 | MIR194-1 |
| ILF3 | ENSG00000150779 | TIMM8B |
| ILF3 | ENSG00000206775 | SNORD37 |
| ILF3 | ENSG00000204003 | LCN6 |
| ILF3 | ENSG00000280278 | FLJ30679 |
| ILF3 | ENSG00000180488 | FAM73A |
| ILF3 | ENSG00000144741 | SLC25A26 |
| ILF3 | ENSG00000255652 | RP11-313F23.4 |
| ILF3 | ENSG00000236324 | RP3-403L10.3 |
| ILF3 | ENSG00000134690 | CDCA8 |
| ILF3 | ENSG00000166477 | LEO1 |

| ILF3 | ENSG00000169032 | MAP2K1 |
| --- | --- | --- |
| ILF3 | ENSG00000271914 | RP4-789D17.5 |
| ILF3 | ENSG00000116212 | LRRC42 |
| ILF3 | ENSG00000265850 | MIR4797 |
| ILF3 | ENSG00000229484 | RP5-888M10.2 |
| ILF3 | ENSG00000228982 | RP11-107G24.3 |
| ILF3 | ENSG00000253917 | AC226119.5 |
| ILF3 | ENSG00000049759 | NEDD4L |
| ILF3 | ENSG00000120662 | MTRF1 |
| ILF3 | ENSG00000143341 | HMCN1 |
| ILF3 | ENSG00000169885 | CALML6 |
| ILF3 | ENSG00000267719 | HP09025 |
| ILF3 | ENSG00000112851 | ERBB2IP |
| ILF3 | ENSG00000277602 | AC005363.11 |
| ILF3 | ENSG00000100554 | ATP6V1D |
| ILF3 | ENSG00000103507 | BCKDK |
| ILF3 | ENSG00000082074 | FYB |
| ILF3 | ENSG00000279605 | RP11-661A12.8 |
| ILF3 | ENSG00000197296 | FITM2 |
| ILF3 | ENSG00000258285 | TESC-AS1 |
| ILF3 | ENSG00000151611 | MMAA |
| ILF3 | ENSG00000198478 | SH3BGRL2 |
| ILF3 | ENSG00000147592 | LACTB2 |
| ILF3 | ENSG00000067560 | RHOA |
| ILF3 | ENSG00000182393 | IFNL1 |
| ILF3 | ENSG00000243445 | RP11-715J22.1 |
| ILF3 | ENSG00000012223 | LTF |
| ILF3 | ENSG00000125247 | TMTC4 |
| ILF3 | ENSG00000247596 | TWF2 |
| ILF3 | ENSG00000163959 | SLC51A |
| ILF3 | ENSG00000165359 | DDX26B |
| ILF3 | ENSG00000160318 | CLDND2 |
| ILF3 | ENSG00000125741 | OPA3 |
| ILF3 | ENSG00000108639 | SYNGR2 |
| ILF3 | ENSG00000263916 | RP11-110H1.4 |
| ILF3 | ENSG00000251348 | HSPD1P11 |
| ILF3 | ENSG00000141458 | NPC1 |
| ILF3 | ENSG00000189157 | FAM47E |
| ILF3 | ENSG00000110060 | PUS3 |
| ILF3 | ENSG00000233509 | ZNF197-AS1 |
| ILF3 | ENSG00000139405 | RITA1 |
| ILF3 | ENSG00000269427 | CTC-429P9.1 |
| ILF3 | ENSG00000220875 | HIST1H3PS1 |
| ILF3 | ENSG00000262678 | RP5-1050D4.4 |
| ILF3 | ENSG00000127666 | TICAM1 |
| ILF3 | ENSG00000092020 | PPP2R3C |
| ILF3 | ENSG00000115128 | SF3B6 |
| ILF3 | ENSG00000269565 | GPR32P1 |
| ILF3 | ENSG00000131149 | GSE1 |
| ILF3 | ENSG00000267054 | CTD- |
| ILF3 | ENSG00000275273 | 2540B15.10 MIR6780A |

| ILF3 | ENSG00000165458 | INPPL1 |
| --- | --- | --- |
| ILF3 | ENSG00000110172 | CHORDC1 |
| ILF3 | ENSG00000173926 | 3-Mar |
| ILF3 | ENSG00000250950 | RP11-33B1.3 |
| ILF3 | ENSG00000261703 | RP11-327F22.5 |
| ILF3 | ENSG00000090273 | NUDC |
| ILF3 | ENSG00000106336 | FBXO24 |
| ILF3 | ENSG00000227907 | RP11-102C16.3 |
| ILF3 | ENSG00000198026 | ZNF335 |
| ILF3 | ENSG00000204178 | TMEM57 |
| ILF3 | ENSG00000077080 | ACTL6B |
| ILF3 | ENSG00000258689 | LINC01269 |
| ILF3 | ENSG00000279212 | RP13-16H11.8 |
| ILF3 | ENSG00000132702 | HAPLN2 |
| ILF3 | ENSG00000011638 | TMEM159 |
| ILF3 | ENSG00000244642 | RN7SL396P |
| ILF3 | ENSG00000275863 | MIR7975 |
| ILF3 | ENSG00000092094 | OSGEP |
| ILF3 | ENSG00000167103 | PIP5KL1 |
| ILF3 | ENSG00000186825 | C2orf27B |
| ILF3 | ENSG00000165533 | TTC8 |
| ILF3 | ENSG00000117411 | B4GALT2 |
| ILF3 | ENSG00000125651 | GTF2F1 |
| ILF3 | ENSG00000112763 | BTN2A1 |
| ILF3 | ENSG00000134001 | EIF2S1 |
| ILF3 | ENSG00000262664 | OVCA2 |
| ILF3 | ENSG00000110880 | CORO1C |
| ILF3 | ENSG00000125775 | SDCBP2 |
| ILF3 | ENSG00000250699 | AP000892.4 |
| ILF3 | ENSG00000130021 | HDHD1 |
| ILF3 | ENSG00000140265 | ZSCAN29 |
| ILF3 | ENSG00000049245 | VAMP3 |
| ILF3 | ENSG00000264493 | MIR4298 |
| ILF3 | ENSG00000160282 | FTCD |
| ILF3 | ENSG00000146707 | POMZP3 |
| ILF3 | ENSG00000259673 | IQCH-AS1 |
| ILF3 | ENSG00000257086 | RP11-783K16.13 |
| ILF3 | ENSG00000124126 | PREX1 |
| ILF3 | ENSG00000100625 | SIX4 |
| ILF3 | ENSG00000225830 | ERCC6 |
| ILF3 | ENSG00000267253 | RP11-209M4.1 |
| ILF3 | ENSG00000247373 | RP11-486O12.2 |
| ILF3 | ENSG00000134419 | RPS15A |
| ILF3 | ENSG00000214439 | FAM185BP |
| ILF3 | ENSG00000187994 | RINL |
| ILF3 | ENSG00000267372 | AC005330.2 |
| ILF3 | ENSG00000263301 | RP11-104H15.8 |
| ILF3 | ENSG00000120705 | ETF1 |
| ILF3 | ENSG00000270433 | RP11-124D2.7 |
| ILF3 | ENSG00000151366 | NDUFC2 |
| ILF3 | ENSG00000132522 | GPS2 |

| ILF3 | ENSG00000258716 | RP11-661G16.2 |
| --- | --- | --- |
| ILF3 | ENSG00000136048 | DRAM1 |
| ILF3 | ENSG00000231864 | RP11-229P13.23 |
| ILF3 | ENSG00000278332 | XXbac- |
| ILF3 | ENSG00000260285 | BPGBPG34I8.2  RP11-600F24.7 |
| ILF3 | ENSG00000075151 | EIF4G3 |
| ILF3 | ENSG00000119523 | ALG2 |
| ILF3 | ENSG00000234203 | RP5-1050D4.2 |
| ILF3 | ENSG00000087191 | PSMC5 |
| ILF3 | ENSG00000266665 | MIR4739 |
| ILF3 | ENSG00000272523 | LINC01023 |
| ILF3 | ENSG00000175309 | PHYKPL |
| ILF3 | ENSG00000128272 | ATF4 |
| ILF3 | ENSG00000114346 | ECT2 |
| ILF3 | ENSG00000233159 | AC007390.4 |
| ILF3 | ENSG00000276925 | RP11-708J19.3 |
| ILF3 | ENSG00000130985 | UBA1 |
| ILF3 | ENSG00000279005 | RP5-1186P10.1 |
| ILF3 | ENSG00000008130 | NADK |
| ILF3 | ENSG00000224046 | AC005076.5 |
| ILF3 | ENSG00000230376 | MEMO1P4 |
| ILF3 | ENSG00000179397 | C1orf101 |
| ILF3 | ENSG00000202444 | RNU5E-6P |
| ILF3 | ENSG00000214185 | XPOTP1 |
| ILF3 | ENSG00000204653 | ASPDH |
| ILF3 | ENSG00000197275 | RAD54B |
| ILF3 | ENSG00000198162 | MAN1A2 |
| ILF3 | ENSG00000268006 | PTOV1-AS1 |
| ILF3 | ENSG00000134569 | LRP4 |
| ILF3 | ENSG00000142733 | MAP3K6 |
| ILF3 | ENSG00000163975 | MFI2 |
| ILF3 | ENSG00000236643 | RP11-175D17.3 |
| ILF3 | ENSG00000273536 | RP11-74M13.6 |
| ILF3 | ENSG00000138615 | CILP |
| ILF3 | ENSG00000165120 | SSMEM1 |
| ILF3 | ENSG00000224467 | AC009313.1 |
| ILF3 | ENSG00000157827 | FMNL2 |
| ILF3 | ENSG00000099795 | NDUFB7 |
| ILF3 | ENSG00000243667 | WDR92 |
| ILF3 | ENSG00000213069 | KRT8P40 |
| ILF3 | ENSG00000123091 | RNF11 |
| ILF3 | ENSG00000119723 | COQ6 |
| ILF3 | ENSG00000148334 | PTGES2 |
| ILF3 | ENSG00000198369 | SPRED2 |
| ILF3 | ENSG00000223496 | EXOSC6 |
| ILF3 | ENSG00000268995 | VN1R82P |
| ILF3 | ENSG00000100888 | CHD8 |
| ILF3 | ENSG00000201633 | Y_RNA |
| ILF3 | ENSG00000213588 | ZBTB9 |
| ILF3 | ENSG00000236290 | EEF1GP7 |
| ILF3 | ENSG00000239857 | GET4 |

| ILF3 | ENSG00000121390 | PSPC1 |
| --- | --- | --- |
| ILF3 | ENSG00000178307 | TMEM11 |
| ILF3 | ENSG00000226036 | RP11-338C15.2 |
| ILF3 | ENSG00000186174 | BCL9L |
| ILF3 | ENSG00000181264 | TMEM136 |
| ILF3 | ENSG00000176058 | TPRN |
| ILF3 | ENSG00000224611 | AC007919.18 |
| ILF3 | ENSG00000269191 | AC005387.2 |
| ILF3 | ENSG00000241015 | TPM3P9 |
| ILF3 | ENSG00000105404 | RABAC1 |
| ILF3 | ENSG00000269720 | CTD- |
| ILF3 | ENSG00000221808 | 2521M24.5  MIR1256 |
| ILF3 | ENSG00000273657 | MIR6792 |
| ILF3 | ENSG00000140406 | MESDC1 |
| ILF3 | ENSG00000213654 | GPSM3 |
| ILF3 | ENSG00000101457 | DNTTIP1 |
| ILF3 | ENSG00000160007 | ARHGAP35 |
| ILF3 | ENSG00000278420 | MIR6819 |
| ILF3 | ENSG00000163793 | DNAJC5G |
| ILF3 | ENSG00000254369 | HOXA-AS3 |
| ILF3 | ENSG00000166123 | GPT2 |
| ILF3 | ENSG00000137216 | TMEM63B |
| ILF3 | ENSG00000204839 | MROH6 |
| ILF3 | ENSG00000267523 | CTD-2537I9.12 |
| ILF3 | ENSG00000149016 | TUT1 |
| ILF3 | ENSG00000266904 | LINC00663 |
| ILF3 | ENSG00000136630 | HLX |
| ILF3 | ENSG00000241127 | YAE1D1 |
| ILF3 | ENSG00000274210 | U1 |
| ILF3 | ENSG00000117242 | PINK1-AS |
| ILF3 | ENSG00000242259 | C22orf39 |
| ILF3 | ENSG00000142661 | MYOM3 |
| ILF3 | ENSG00000143198 | MGST3 |
| ILF3 | ENSG00000164053 | ATRIP |
| ILF3 | ENSG00000089289 | IGBP1 |
| ILF3 | ENSG00000159733 | ZFYVE28 |
| ILF3 | ENSG00000277324 | RP11-850A17.1 |
| ILF3 | ENSG00000251191 | LINC00589 |
| ILF3 | ENSG00000177106 | EPS8L2 |
| ILF3 | ENSG00000107829 | FBXW4 |
| ILF3 | ENSG00000099889 | ARVCF |
| ILF3 | ENSG00000255129 | RP11-839D17.3 |
| ILF3 | ENSG00000207181 | SNORA14B |
| ILF3 | ENSG00000141086 | CTRL |
| ILF3 | ENSG00000235549 | EIF1P2 |
| ILF3 | ENSG00000104064 | GABPB1 |
| ILF3 | ENSG00000270269 | IMMP1LP1 |
| ILF3 | ENSG00000079156 | OSBPL6 |
| ILF3 | ENSG00000078177 | N4BP2 |
| ILF3 | ENSG00000049239 | H6PD |
| ILF3 | ENSG00000160539 | PPAPDC3 |

| ILF3 | ENSG00000081386 | ZNF510 |
| --- | --- | --- |
| ILF3 | ENSG00000167191 | GPRC5B |
| ILF3 | ENSG00000188223 | AC002398.9 |
| ILF3 | ENSG00000115008 | IL1A |
| ILF3 | ENSG00000196275 | GTF2IRD2 |
| ILF3 | ENSG00000270313 | COX6CP16 |
| ILF3 | ENSG00000157500 | APPL1 |
| ILF3 | ENSG00000270995 | RP11-29B11.5 |
| ILF3 | ENSG00000221823 | PPP3R1 |
| ILF3 | ENSG00000277985 | SNORA67 |
| ILF3 | ENSG00000228395 | RP11-216B9.6 |
| ILF3 | ENSG00000278740 | RP11-147L13.14 |
| ILF3 | ENSG00000138385 | SSB |
| ILF3 | ENSG00000263326 | RP11-266L9.6 |
| ILF3 | ENSG00000144815 | NXPE3 |
| ILF3 | ENSG00000134864 | GGACT |
| ILF3 | ENSG00000269894 | RP11-1020A11.1 |
| ILF3 | ENSG00000267298 | AC006116.19 |
| ILF3 | ENSG00000116754 | SRSF11 |
| ILF3 | ENSG00000275719 | CTB-147N14.6 |
| ILF3 | ENSG00000260170 | RP11-96O20.4 |
| ILF3 | ENSG00000213262 | AL365331.2 |
| ILF3 | ENSG00000199702 | RNU6-170P |
| ILF3 | ENSG00000207145 | SNORA18 |
| ILF3 | ENSG00000226179 | LINC00685 |
| ILF3 | ENSG00000117569 | PTBP2 |
| ILF3 | ENSG00000185658 | BRWD1 |
| ILF3 | ENSG00000205899 | BHLHA9 |
| ILF3 | ENSG00000262583 | RP11-77K12.5 |
| ILF3 | ENSG00000033800 | PIAS1 |
| ILF3 | ENSG00000279080 | CTA-228A9.4 |
| ILF3 | ENSG00000167377 | ZNF23 |
| ILF3 | ENSG00000232118 | BACH1-AS1 |
| ILF3 | ENSG00000252755 | RNU6-703P |
| ILF3 | ENSG00000177576 | C18orf32 |
| ILF3 | ENSG00000119977 | TCTN3 |
| ILF3 | ENSG00000163349 | HIPK1 |
| ILF3 | ENSG00000032219 | ARID4A |
| ILF3 | ENSG00000234788 | HSPA8P3 |
| ILF3 | ENSG00000236675 | MTX1P1 |
| ILF3 | ENSG00000250988 | SNHG21 |
| ILF3 | ENSG00000163933 | RFT1 |
| ILF3 | ENSG00000268093 | AC022154.7 |
| ILF3 | ENSG00000149809 | TM7SF2 |
| ILF3 | ENSG00000276603 | RP11-425M5.7 |
| ILF3 | ENSG00000139624 | CERS5 |
| ILF3 | ENSG00000164305 | CASP3 |
| ILF3 | ENSG00000267346 | EIF5AP3 |
| ILF3 | ENSG00000279382 | RP11-449J21.3 |
| ILF3 | ENSG00000109917 | ZPR1 |
| ILF3 | ENSG00000162231 | NXF1 |

| ILF3 | ENSG00000234693 | RP4-530I15.6 |
| --- | --- | --- |
| ILF3 | ENSG00000170456 | DENND5B |
| ILF3 | ENSG00000254093 | PINX1 |
| ILF3 | ENSG00000114395 | CYB561D2 |
| ILF3 | ENSG00000055917 | PUM2 |
| ILF3 | ENSG00000156427 | FGF18 |
| ILF3 | ENSG00000239335 | LLPH-AS1 |
| ILF3 | ENSG00000170412 | GPRC5C |
| ILF3 | ENSG00000268565 | AC005339.2 |
| ILF3 | ENSG00000237742 | RP11-624M8.1 |
| ILF3 | ENSG00000006025 | OSBPL7 |
| ILF3 | ENSG00000279765 | CHD2 |
| ILF3 | ENSG00000243403 | RP11-330L19.1 |
| ILF3 | ENSG00000164919 | COX6C |
| ILF3 | ENSG00000132005 | RFX1 |
| ILF3 | ENSG00000207647 | MIR153-1 |
| ILF3 | ENSG00000172992 | DCAKD |
| ILF3 | ENSG00000169994 | MYO7B |
| ILF3 | ENSG00000105486 | LIG1 |
| ILF3 | ENSG00000188064 | WNT7B |
| ILF3 | ENSG00000279583 | RP11-368N21.5 |
| ILF3 | ENSG00000077157 | PPP1R12B |
| ILF3 | ENSG00000005884 | ITGA3 |
| ILF3 | ENSG00000197223 | C1D |
| ILF3 | ENSG00000242110 | AMACR |
| ILF3 | ENSG00000125046 | SSUH2 |
| ILF3 | ENSG00000236358 | RP5-827C21.2 |
| ILF3 | ENSG00000161618 | ALDH16A1 |
| ILF3 | ENSG00000256273 | RP11-71J4.2 |
| ILF3 | ENSG00000197943 | PLCG2 |
| ILF3 | ENSG00000234166 | ARHGEF19- |
| ILF3 | ENSG00000124374 | AS1  PAIP2B |
| ILF3 | ENSG00000244187 | TMEM141 |
| ILF3 | ENSG00000206828 | U1 |
| ILF3 | ENSG00000278267 | MIR6859-1 |
| ILF3 | ENSG00000102879 | CORO1A |
| ILF3 | ENSG00000139613 | SMARCC2 |
| ILF3 | ENSG00000189241 | TSPYL1 |
| ILF3 | ENSG00000033178 | UBA6 |
| ILF3 | ENSG00000231355 | AP000302.58 |
| ILF3 | ENSG00000147050 | KDM6A |
| ILF3 | ENSG00000175203 | DCTN2 |
| ILF3 | ENSG00000131044 | TTLL9 |
| ILF3 | ENSG00000232936 | RP11-80H5.2 |
| ILF3 | ENSG00000144354 | CDCA7 |
| ILF3 | ENSG00000179526 | SHARPIN |
| ILF3 | ENSG00000185608 | MRPL40 |
| ILF3 | ENSG00000164051 | CCDC51 |
| ILF3 | ENSG00000245748 | RP11-367J11.2 |
| ILF3 | ENSG00000141551 | CSNK1D |
| ILF3 | ENSG00000213015 | ZNF580 |

| ILF3 | ENSG00000113068 | PFDN1 |
| --- | --- | --- |
| ILF3 | ENSG00000115194 | SLC30A3 |
| ILF3 | ENSG00000162236 | STX5 |
| ILF3 | ENSG00000070526 | ST6GALNAC1 |
| ILF3 | ENSG00000010292 | NCAPD2 |
| ILF3 | ENSG00000254596 | CTD-3074O7.7 |
| ILF3 | ENSG00000165914 | TTC7B |
| ILF3 | ENSG00000279673 | RP11-185E8.2 |
| ILF3 | ENSG00000225463 | ZNF70P1 |
| ILF3 | ENSG00000228150 | RP11-84A14.4 |
| ILF3 | ENSG00000253785 | CTC-308K20.3 |
| ILF3 | ENSG00000176302 | FOXR1 |
| ILF3 | ENSG00000208797 | SNORD73A |
| ILF3 | ENSG00000254016 | ALG1L10P |
| ILF3 | ENSG00000166377 | ATP9B |
| ILF3 | ENSG00000244573 | RPL30P11 |
| ILF3 | ENSG00000172037 | LAMB2 |
| ILF3 | ENSG00000106459 | NRF1 |
| ILF3 | ENSG00000238719 | RNU7-96P |
| ILF3 | ENSG00000145354 | CISD2 |
| ILF3 | ENSG00000204305 | AGER |
| ILF3 | ENSG00000167363 | FN3K |
| ILF3 | ENSG00000241962 | C2orf15 |
| ILF3 | ENSG00000104320 | NBN |
| ILF3 | ENSG00000263766 | RP11-580I16.2 |
| ILF3 | ENSG00000143162 | CREG1 |
| ILF3 | ENSG00000135372 | NAT10 |
| ILF3 | ENSG00000231927 | AC102953.6 |
| ILF3 | ENSG00000271741 | RP11-244H3.4 |
| ILF3 | ENSG00000258210 | RP11-478C19.2 |
| ILF3 | ENSG00000252882 | RNU6-1137P |
| ILF3 | ENSG00000150459 | SAP18 |
| ILF3 | ENSG00000187862 | TTC24 |
| ILF3 | ENSG00000172197 | MBOAT1 |
| ILF3 | ENSG00000138400 | MDH1B |
| ILF3 | ENSG00000271810 | RP11-426L16.10 |
| ILF3 | ENSG00000165480 | SKA3 |
| ILF3 | ENSG00000162402 | USP24 |
| ILF3 | ENSG00000254762 | RP11-867G23.2 |
| ILF3 | ENSG00000081014 | AP4E1 |
| ILF3 | ENSG00000165006 | UBAP1 |
| ILF3 | ENSG00000133983 | COX16 |
| ILF3 | ENSG00000163746 | PLSCR2 |
| ILF3 | ENSG00000248371 | CTC-347C20.2 |
| ILF3 | ENSG00000109680 | TBC1D19 |
| ILF3 | ENSG00000164985 | PSIP1 |
| ILF3 | ENSG00000183340 | JRKL |
| ILF3 | ENSG00000254760 | CTD-2616J11.3 |
| ILF3 | ENSG00000114439 | BBX |
| ILF3 | ENSG00000088451 | TGDS |
| ILF3 | ENSG00000180539 | C9orf139 |

| ILF3 | ENSG00000261357 | RP11-626G11.1 |
| --- | --- | --- |
| ILF3 | ENSG00000135723 | FHOD1 |
| ILF3 | ENSG00000201109 | RNA5SP245 |
| ILF3 | ENSG00000104907 | TRMT1 |
| ILF3 | ENSG00000231707 | PABPC1P1 |
| ILF3 | ENSG00000169955 | ZNF747 |
| ILF3 | ENSG00000115137 | DNAJC27 |
| ILF3 | ENSG00000211731 | TRBV5-7 |
| ILF3 | ENSG00000227671 | MIR3916 |
| ILF3 | ENSG00000273311 | DGCR11 |
| ILF3 | ENSG00000272516 | RP11-573G6.9 |
| ILF3 | ENSG00000225793 | RP1-234P15.4 |
| ILF3 | ENSG00000189167 | ZAR1L |
| ILF3 | ENSG00000110243 | APOA5 |
| ILF3 | ENSG00000132825 | PPP1R3D |
| ILF3 | ENSG00000156050 | FAM161B |
| ILF3 | ENSG00000078304 | PPP2R5C |
| ILF3 | ENSG00000134020 | PEBP4 |
| ILF3 | ENSG00000231006 | RPL7P32 |
| ILF3 | ENSG00000263293 | RP11-290H9.4 |
| ILF3 | ENSG00000096968 | JAK2 |
| ILF3 | ENSG00000143079 | CTTNBP2NL |
| ILF3 | ENSG00000259426 | RP11-253M7.1 |
| ILF3 | ENSG00000203395 | AC015969.3 |
| ILF3 | ENSG00000268144 | NIFKP6 |
| ILF3 | ENSG00000132842 | AP3B1 |
| ILF3 | ENSG00000147548 | WHSC1L1 |
| ILF3 | ENSG00000103174 | NAGPA |
| ILF3 | ENSG00000112149 | CD83 |
| ILF3 | ENSG00000105835 | NAMPT |
| ILF3 | ENSG00000023909 | GCLM |
| ILF3 | ENSG00000218510 | LINC00339 |
| ILF3 | ENSG00000134255 | CEPT1 |
| ILF3 | ENSG00000249602 | RP11-98D18.3 |
| ILF3 | ENSG00000122224 | LY9 |
| ILF3 | ENSG00000277022 | RP3-453C12.15 |
| ILF3 | ENSG00000228288 | PCAT6 |
| ILF3 | ENSG00000227933 | RP11-331F9.4 |
| ILF3 | ENSG00000074582 | BCS1L |
| ILF3 | ENSG00000112308 | C6orf62 |
| ILF3 | ENSG00000086967 | MYBPC2 |
| ILF3 | ENSG00000105559 | PLEKHA4 |
| ILF3 | ENSG00000172922 | RNASEH2C |
| ILF3 | ENSG00000264266 | MIR4322 |
| ILF3 | ENSG00000172746 | RP11-344H11.4 |
| ILF3 | ENSG00000204978 | ERICH4 |
| ILF3 | ENSG00000235681 | AC008746.5 |
| ILF3 | ENSG00000229320 | KRT8P12 |
| ILF3 | ENSG00000225986 | UBXN10-AS1 |
| ILF3 | ENSG00000100897 | DCAF11 |
| ILF3 | ENSG00000135968 | GCC2 |

| ILF3 | ENSG00000102580 | DNAJC3 |
| --- | --- | --- |
| ILF3 | ENSG00000014164 | ZC3H3 |
| ILF3 | ENSG00000122034 | GTF3A |
| ILF3 | ENSG00000172232 | AZU1 |
| ILF3 | ENSG00000204634 | TBC1D8 |
| ILF3 | ENSG00000145990 | GFOD1 |
| ILF3 | ENSG00000143207 | RFWD2 |
| ILF3 | ENSG00000228521 | AC099552.3 |
| ILF3 | ENSG00000100138 | NHP2L1 |
| ILF3 | ENSG00000273209 | RP11-107N15.1 |
| ILF3 | ENSG00000251583 | AC008427.2 |
| ILF3 | ENSG00000182376 | RP5-1142A6.8 |
| ILF3 | ENSG00000227060 | LINC00629 |
| ILF3 | ENSG00000198700 | IPO9 |
| ILF3 | ENSG00000254756 | RP11-867G23.12 |
| ILF3 | ENSG00000140829 | DHX38 |
| ILF3 | ENSG00000267670 | CTB-55O6.4 |
| ILF3 | ENSG00000108298 | RPL19 |
| ILF3 | ENSG00000225361 | PPP1R26-AS1 |
| ILF3 | ENSG00000230447 | CTF2P |
| ILF3 | ENSG00000118564 | FBXL5 |
| ILF3 | ENSG00000186765 | FSCN2 |
| ILF3 | ENSG00000182287 | AP1S2 |
| ILF3 | ENSG00000278341 | RP5-1142A6.10 |
| ILF3 | ENSG00000160124 | CCDC58 |
| ILF3 | ENSG00000124608 | AARS2 |
| ILF3 | ENSG00000234735 | AL022237.3 |
| ILF3 | ENSG00000105671 | DDX49 |
| ILF3 | ENSG00000083642 | PDS5B |
| ILF3 | ENSG00000171786 | NHLH1 |
| ILF3 | ENSG00000245293 | RP11-286E11.1 |
| ILF3 | ENSG00000246263 | KB-431C1.4 |
| ILF3 | ENSG00000269486 | CTC-360G5.9 |
| ILF3 | ENSG00000048162 | NOP16 |
| ILF3 | ENSG00000159337 | PLA2G4D |
| ILF3 | ENSG00000233927 | RPS28 |
| ILF3 | ENSG00000257000 | RP13-820C6.2 |
| ILF3 | ENSG00000268412 | TRMT112P6 |
| ILF3 | ENSG00000158806 | NPM2 |
| ILF3 | ENSG00000258599 | RP11-84C10.1 |
| ILF3 | ENSG00000198753 | PLXNB3 |
| ILF3 | ENSG00000108518 | PFN1 |
| ILF3 | ENSG00000133812 | SBF2 |
| ILF3 | ENSG00000217718 | AC064847.4 |
| ILF3 | ENSG00000145700 | ANKRD31 |
| ILF3 | ENSG00000228843 | RP11-112J3.15 |
| ILF3 | ENSG00000116990 | MYCL |
| ILF3 | ENSG00000204311 | DFNB59 |
| ILF3 | ENSG00000248975 | CTD-2251F13.1 |
| ILF3 | ENSG00000162437 | RAVER2 |
| ILF3 | ENSG00000204283 | FLJ45079 |

| ILF3 | ENSG00000105176 | URI1 |
| --- | --- | --- |
| ILF3 | ENSG00000273148 | RP5-1068E13.7 |
| ILF3 | ENSG00000100908 | EMC9 |
| ILF3 | ENSG00000204394 | VARS |
| ILF3 | ENSG00000040199 | PHLPP2 |
| ILF3 | ENSG00000204620 | RP11-1148L6.9 |
| ILF3 | ENSG00000107651 | SEC23IP |
| ILF3 | ENSG00000135476 | ESPL1 |
| ILF3 | ENSG00000099992 | TBC1D10A |
| ILF3 | ENSG00000214433 | GOLGA2P8 |
| ILF3 | ENSG00000112699 | GMDS |
| ILF3 | ENSG00000011451 | WIZ |
| ILF3 | ENSG00000270012 | LL0XNC01- |
| ILF3 | ENSG00000222378 | 7P3.1 RNA5SP44 |
| ILF3 | ENSG00000150281 | CTF1 |
| ILF3 | ENSG00000165476 | REEP3 |
| ILF3 | ENSG00000267551 | AC005264.2 |
| ILF3 | ENSG00000185689 | C6orf201 |
| ILF3 | ENSG00000272142 | RP11-428J1.5 |
| ILF3 | ENSG00000152464 | RPP38 |
| ILF3 | ENSG00000130733 | YIPF2 |
| ILF3 | ENSG00000074370 | ATP2A3 |
| ILF3 | ENSG00000269971 | RP3-426I6.5 |
| ILF3 | ENSG00000141753 | IGFBP4 |
| ILF3 | ENSG00000151575 | TEX9 |
| ILF3 | ENSG00000243053 | RPL31P58 |
| ILF3 | ENSG00000214881 | TMEM14D |
| ILF3 | ENSG00000258675 | RP11-299L17.3 |
| ILF3 | ENSG00000137124 | ALDH1B1 |
| ILF3 | ENSG00000138463 | DIRC2 |
| ILF3 | ENSG00000159685 | CHCHD6 |
| ILF3 | ENSG00000124587 | PEX6 |
| ILF3 | ENSG00000258153 | HSPE1P4 |
| ILF3 | ENSG00000171224 | C10orf35 |
| ILF3 | ENSG00000185670 | ZBTB3 |
| ILF3 | ENSG00000129055 | ANAPC13 |
| ILF3 | ENSG00000085872 | CHERP |
| ILF3 | ENSG00000199711 | Y_RNA |
| ILF3 | ENSG00000278259 | MYO19 |
| ILF3 | ENSG00000079337 | RAPGEF3 |
| ILF3 | ENSG00000124466 | LYPD3 |
| ILF3 | ENSG00000109911 | ELP4 |
| ILF3 | ENSG00000105220 | GPI |
| ILF3 | ENSG00000270021 | CTC-203F4.2 |
| ILF3 | ENSG00000048649 | RSF1 |
| ILF3 | ENSG00000115977 | AAK1 |
| ILF3 | ENSG00000255153 | TOLLIP-AS1 |
| ILF3 | ENSG00000127423 | AUNIP |
| ILF3 | ENSG00000178917 | ZNF852 |
| ILF3 | ENSG00000134453 | RBM17 |
| ILF3 | ENSG00000265460 | RP11-690G19.4 |

| ILF3 | ENSG00000149548 | CCDC15 |
| --- | --- | --- |
| ILF3 | ENSG00000109113 | RAB34 |
| ILF3 | ENSG00000252139 | SCARNA18 |
| ILF3 | ENSG00000215520 | RPS4XP4 |
| ILF3 | ENSG00000257639 | CTB-31N19.2 |
| ILF3 | ENSG00000160193 | WDR4 |
| ILF3 | ENSG00000105085 | MED26 |
| ILF3 | ENSG00000008226 | DLEC1 |
| ILF3 | ENSG00000166260 | COX11 |
| ILF3 | ENSG00000064300 | NGFR |
| ILF3 | ENSG00000117597 | DIEXF |
| ILF3 | ENSG00000177103 | DSCAML1 |
| ILF3 | ENSG00000114391 | RPL24 |
| ILF3 | ENSG00000142609 | CFAP74 |
| ILF3 | ENSG00000257524 | RP11-203J24.9 |
| ILF3 | ENSG00000143575 | HAX1 |
| ILF3 | ENSG00000123545 | NDUFAF4 |
| ILF3 | ENSG00000115274 | INO80B |
| ILF3 | ENSG00000237976 | RP11-126K1.6 |
| ILF3 | ENSG00000149084 | HSD17B12 |
| ILF3 | ENSG00000100034 | PPM1F |
| ILF3 | ENSG00000264249 | MIR3912 |
| ILF3 | ENSG00000092820 | EZR |
| ILF3 | ENSG00000115866 | DARS |
| ILF3 | ENSG00000123485 | HJURP |
| ILF3 | ENSG00000173213 | RP11-683L23.1 |
| ILF3 | ENSG00000149182 | ARFGAP2 |
| ILF3 | ENSG00000225401 | TGIF2P1 |
| ILF3 | ENSG00000163888 | CAMK2N2 |
| ILF3 | ENSG00000133805 | AMPD3 |
| ILF3 | ENSG00000199631 | SNORD33 |
| ILF3 | ENSG00000105808 | RASA4 |
| ILF3 | ENSG00000271715 | CTD-2256P15.5 |
| ILF3 | ENSG00000233585 | AC115617.2 |
| ILF3 | ENSG00000254501 | AP003068.9 |
| ILF3 | ENSG00000081760 | AACS |
| ILF3 | ENSG00000120071 | KANSL1 |
| ILF3 | ENSG00000154767 | XPC |
| ILF3 | ENSG00000133477 | FAM83F |
| ILF3 | ENSG00000253125 | RP11-459E5.1 |
| ILF3 | ENSG00000196923 | PDLIM7 |
| ILF3 | ENSG00000234455 | AC067960.1 |
| ILF3 | ENSG00000176236 | C10orf111 |
| ILF3 | ENSG00000117399 | CDC20 |
| ILF3 | ENSG00000131771 | PPP1R1B |
| ILF3 | ENSG00000134245 | WNT2B |
| ILF3 | ENSG00000272721 | RP11-778D9.12 |
| ILF3 | ENSG00000161904 | LEMD2 |
| ILF3 | ENSG00000103978 | TMEM87A |
| ILF3 | ENSG00000110442 | COMMD9 |
| ILF3 | ENSG00000180185 | FAHD1 |

| ILF3 | ENSG00000182010 | RTKN2 |
| --- | --- | --- |
| ILF3 | ENSG00000181915 | ADO |
| ILF3 | ENSG00000199580 | Y_RNA |
| ILF3 | ENSG00000107625 | DDX50 |
| ILF3 | ENSG00000146063 | TRIM41 |
| ILF3 | ENSG00000229407 | RP11-12M5.3 |
| ILF3 | ENSG00000054282 | SDCCAG8 |
| ILF3 | ENSG00000215190 | LINC00680 |
| ILF3 | ENSG00000165125 | TRPV6 |
| ILF3 | ENSG00000154978 | VOPP1 |
| ILF3 | ENSG00000266006 | MIR4488 |
| ILF3 | ENSG00000275655 | Metazoa_SRP |
| ILF3 | ENSG00000272886 | DCP1A |
| ILF3 | ENSG00000243725 | TTC4 |
| ILF3 | ENSG00000099783 | HNRNPM |
| ILF3 | ENSG00000104763 | ASAH1 |
| ILF3 | ENSG00000105610 | KLF1 |
| ILF3 | ENSG00000011405 | PIK3C2A |
| ILF3 | ENSG00000084774 | CAD |
| ILF3 | ENSG00000185633 | NDUFA4L2 |
| ILF3 | ENSG00000197969 | VPS13A |
| ILF3 | ENSG00000255839 | RP11-338K17.8 |
| ILF3 | ENSG00000218027 | RP11-157J24.1 |
| ILF3 | ENSG00000260494 | AC002310.10 |
| ILF3 | ENSG00000235750 | KIAA0040 |
| ILF3 | ENSG00000129315 | CCNT1 |
| ILF3 | ENSG00000166949 | SMAD3 |
| ILF3 | ENSG00000264349 | MIR4258 |
| ILF3 | ENSG00000113328 | CCNG1 |
| ILF3 | ENSG00000135974 | C2orf49 |
| ILF3 | ENSG00000115762 | PLEKHB2 |
| ILF3 | ENSG00000228729 | RP11-211A18.2 |
| ILF3 | ENSG00000121578 | B4GALT4 |
| ILF3 | ENSG00000260236 | RP11-708J19.1 |
| ILF3 | ENSG00000105879 | CBLL1 |
| ILF3 | ENSG00000101751 | POLI |
| ILF3 | ENSG00000132698 | RAB25 |
| ILF3 | ENSG00000163374 | YY1AP1 |
| ILF3 | ENSG00000101557 | USP14 |
| ILF3 | ENSG00000172315 | TP53RK |
| ILF3 | ENSG00000136986 | DERL1 |
| ILF3 | ENSG00000197043 | ANXA6 |
| ILF3 | ENSG00000260793 | RP5-882C2.2 |
| ILF3 | ENSG00000116906 | GNPAT |
| ILF3 | ENSG00000270011 | ZNF559-ZNF177 |
| ILF3 | ENSG00000117595 | IRF6 |
| ILF3 | ENSG00000279441 | LA16c-313D11.13 |
| ILF3 | ENSG00000198355 | PIM3 |
| ILF3 | ENSG00000140455 | USP3 |
| ILF3 | ENSG00000149257 | SERPINH1 |
| ILF3 | ENSG00000116750 | UCHL5 |

| ILF3 | ENSG00000260710 | RP11-616M22.7 |
| --- | --- | --- |
| ILF3 | ENSG00000243959 | RN7SL684P |
| ILF3 | ENSG00000131435 | PDLIM4 |
| ILF3 | ENSG00000268516 | CTD-3138B18.5 |
| ILF3 | ENSG00000140990 | NDUFB10 |
| ILF3 | ENSG00000110025 | SNX15 |
| ILF3 | ENSG00000121957 | GPSM2 |
| ILF3 | ENSG00000136840 | ST6GALNAC4 |
| ILF3 | ENSG00000148935 | GAS2 |
| ILF3 | ENSG00000186716 | BCR |
| ILF3 | ENSG00000145782 | ATG12 |
| ILF3 | ENSG00000144306 | SCRN3 |
| ILF3 | ENSG00000172086 | KRCC1 |
| ILF3 | ENSG00000161547 | SRSF2 |
| ILF3 | ENSG00000238498 | snoU13 |
| ILF3 | ENSG00000058866 | DGKG |
| ILF3 | ENSG00000173950 | XXYLT1 |
| ILF3 | ENSG00000175115 | PACS1 |
| ILF3 | ENSG00000216708 | CIR1P3 |
| ILF3 | ENSG00000252202 | Y_RNA |
| ILF3 | ENSG00000272768 | RP4-673M15.1 |
| ILF3 | ENSG00000229525 | AC053503.4 |
| ILF3 | ENSG00000105227 | PRX |
| ILF3 | ENSG00000262521 | AJ003147.8 |
| ILF3 | ENSG00000236226 | RP11-645N11.3 |
| ILF3 | ENSG00000166295 | ANAPC16 |
| ILF3 | ENSG00000204934 | ATP6V0E2-AS1 |
| ILF3 | ENSG00000267007 | CTB-31O20.3 |
| ILF3 | ENSG00000152683 | SLC30A6 |
| ILF3 | ENSG00000175701 | LINC00116 |
| ILF3 | ENSG00000199440 | Y_RNA |
| ILF3 | ENSG00000149575 | SCN2B |
| ILF3 | ENSG00000179528 | LBX2 |
| ILF3 | ENSG00000228979 | CTD-2501B8.5 |
| ILF3 | ENSG00000222044 | RP5-1039K5.16 |
| ILF3 | ENSG00000129292 | PHF20L1 |
| ILF3 | ENSG00000175575 | PAAF1 |
| ILF3 | ENSG00000169860 | P2RY1 |
| ILF3 | ENSG00000242066 | RN7SL214P |
| ILF3 | ENSG00000100461 | RBM23 |
| ILF3 | ENSG00000076984 | MAP2K7 |
| ILF3 | ENSG00000114784 | EIF1B |
| ILF3 | ENSG00000244274 | DBNDD2 |
| ILF3 | ENSG00000226471 | CTA-292E10.6 |
| ILF3 | ENSG00000114735 | HEMK1 |
| ILF3 | ENSG00000180279 | MGC45922 |
| ILF3 | ENSG00000110075 | PPP6R3 |
| ILF3 | ENSG00000279031 | LA16c-360H6.1 |
| ILF3 | ENSG00000204518 | AADACL4 |
| ILF3 | ENSG00000260796 | RP11-1348G14.5 |
| ILF3 | ENSG00000179455 | MKRN3 |

| ILF3 | ENSG00000252200 | snoZ6 |
| --- | --- | --- |
| ILF3 | ENSG00000125885 | MCM8 |
| ILF3 | ENSG00000139625 | MAP3K12 |
| ILF3 | ENSG00000242028 | HYPK |
| ILF3 | ENSG00000262533 | RP11-667K14.4 |
| ILF3 | ENSG00000262180 | OCLM |
| ILF3 | ENSG00000114062 | UBE3A |
| ILF3 | ENSG00000243562 | RN7SL838P |
| ILF3 | ENSG00000145439 | CBR4 |
| ILF3 | ENSG00000169105 | CHST14 |
| ILF3 | ENSG00000271551 | RP11-230C9.2 |
| ILF3 | ENSG00000142459 | EVI5L |
| ILF3 | ENSG00000143382 | ADAMTSL4 |
| ILF3 | ENSG00000252106 | RNY3P15 |
| ILF3 | ENSG00000175166 | PSMD2 |
| ILF3 | ENSG00000218107 | RP1-261G23.4 |
| ILF3 | ENSG00000255046 | RP11-297N6.4 |
| ILF3 | ENSG00000103707 | MTFMT |
| ILF3 | ENSG00000132424 | PNISR |
| ILF3 | ENSG00000165879 | FRAT1 |
| ILF3 | ENSG00000146453 | PNLDC1 |
| ILF3 | ENSG00000208009 | MIR130A |
| ILF3 | ENSG00000234420 | ZNF37BP |
| ILF3 | ENSG00000134109 | EDEM1 |
| ILF3 | ENSG00000119285 | HEATR1 |
| ILF3 | ENSG00000277959 | RP11-45A17.2 |
| ILF3 | ENSG00000266929 | RP11-400F19.8 |
| ILF3 | ENSG00000230872 | AC092338.5 |
| ILF3 | ENSG00000105223 | PLD3 |
| ILF3 | ENSG00000240225 | ZNF542P |
| ILF3 | ENSG00000130520 | LSM4 |
| ILF3 | ENSG00000270332 | SMC2-AS1 |
| ILF3 | ENSG00000218073 | RP1-13D10.2 |
| ILF3 | ENSG00000188659 | FAM154B |
| ILF3 | ENSG00000214093 | RP11-247I13.3 |
| ILF3 | ENSG00000173276 | ZBTB21 |
| ILF3 | ENSG00000138073 | PREB |
| ILF3 | ENSG00000168014 | C2CD3 |
| ILF3 | ENSG00000230524 | COL6A4P1 |
| ILF3 | ENSG00000198743 | SLC5A3 |
| ILF3 | ENSG00000164465 | DCBLD1 |
| ILF3 | ENSG00000183145 | RIPPLY3 |
| ILF3 | ENSG00000008441 | NFIX |
| ILF3 | ENSG00000118816 | CCNI |
| ILF3 | ENSG00000082512 | TRAF5 |
| ILF3 | ENSG00000125971 | DYNLRB1 |
| ILF3 | ENSG00000266853 | ITM2BP1 |
| ILF3 | ENSG00000138018 | EPT1 |
| ILF3 | ENSG00000011376 | LARS2 |
| ILF3 | ENSG00000164180 | TMEM161B |
| ILF3 | ENSG00000119711 | ALDH6A1 |

| ILF3 | ENSG00000127125 | PPCS |
| --- | --- | --- |
| ILF3 | ENSG00000171357 | LURAP1 |
| ILF3 | ENSG00000138375 | SMARCAL1 |
| ILF3 | ENSG00000105707 | HPN |
| ILF3 | ENSG00000173726 | TOMM20 |
| ILF3 | ENSG00000278463 | HIST1H2AB |
| ILF3 | ENSG00000228146 | CASP16 |
| ILF3 | ENSG00000074695 | LMAN1 |
| ILF3 | ENSG00000126773 | PCNXL4 |
| ILF3 | ENSG00000272758 | RP11-299J3.8 |
| ILF3 | ENSG00000264823 | MIR3154 |
| ILF3 | ENSG00000184009 | ACTG1 |
| ILF3 | ENSG00000228401 | RP11-251M1.1 |
| ILF3 | ENSG00000224505 | AC002117.1 |
| ILF3 | ENSG00000215158 | RP11-1023L17.1 |
| ILF3 | ENSG00000105953 | OGDH |
| ILF3 | ENSG00000235162 | C12orf75 |
| ILF3 | ENSG00000261474 | RP11-452L6.1 |
| ILF3 | ENSG00000168811 | IL12A |
| ILF3 | ENSG00000130303 | BST2 |
| ILF3 | ENSG00000167771 | RCOR2 |
| ILF3 | ENSG00000206767 | RNU6-949P |
| ILF3 | ENSG00000229950 | TFAP2A-AS1 |
| ILF3 | ENSG00000087338 | GMCL1 |
| ILF3 | ENSG00000120709 | FAM53C |
| ILF3 | ENSG00000153107 | ANAPC1 |
| ILF3 | ENSG00000230844 | ZNF674-AS1 |
| ILF3 | ENSG00000240577 | RN7SL445P |
| ILF3 | ENSG00000095587 | TLL2 |
| ILF3 | ENSG00000184205 | TSPYL2 |
| ILF3 | ENSG00000213563 | C8orf82 |
| ILF3 | ENSG00000125821 | DTD1 |
| ILF3 | ENSG00000183401 | CCDC159 |
| ILF3 | ENSG00000236499 | LINC00896 |
| ILF3 | ENSG00000231977 | RP5-963E22.4 |
| ILF3 | ENSG00000208001 | MIR431 |
| ILF3 | ENSG00000262772 | RP11-353N14.2 |
| ILF3 | ENSG00000109881 | CCDC34 |
| ILF3 | ENSG00000280113 | AL357140.1 |
| ILF3 | ENSG00000185269 | NOTUM |
| ILF3 | ENSG00000125735 | TNFSF14 |
| ILF3 | ENSG00000202252 | SNORD14C |
| ILF3 | ENSG00000258056 | RP11-644F5.11 |
| ILF3 | ENSG00000107223 | EDF1 |
| ILF3 | ENSG00000184599 | FAM19A3 |
| ILF3 | ENSG00000213916 | RPL13P |
| ILF3 | ENSG00000166682 | TMPRSS5 |
| ILF3 | ENSG00000126249 | PDCD2L |
| ILF3 | ENSG00000143515 | ATP8B2 |
| ILF3 | ENSG00000273983 | HIST1H3G |
| ILF3 | ENSG00000100316 | RPL3 |

| ILF3 | ENSG00000163704 | PRRT3 |
| --- | --- | --- |
| ILF3 | ENSG00000081181 | ARG2 |
| ILF3 | ENSG00000176020 | AMIGO3 |
| ILF3 | ENSG00000106665 | CLIP2 |
| ILF3 | ENSG00000174405 | LIG4 |
| ILF3 | ENSG00000230266 | XXYLT1-AS2 |
| ILF3 | ENSG00000106799 | TGFBR1 |
| ILF3 | ENSG00000198106 | SNX29P2 |
| ILF3 | ENSG00000108557 | RAI1 |
| ILF3 | ENSG00000275342 | SGK223; |
| ILF3 | ENSG00000167460 | TPM4 |
| ILF3 | ENSG00000262304 | TRPV1 |
| ILF3 | ENSG00000141232 | TOB1 |
| ILF3 | ENSG00000145337 | PYURF |
| ILF3 | ENSG00000242616 | GNG10 |
| ILF3 | ENSG00000182578 | CSF1R |
| ILF3 | ENSG00000269915 | AP006621.9 |
| ILF3 | ENSG00000007341 | ST7L |
| ILF3 | ENSG00000228426 | RP11-402L1.11 |
| ILF3 | ENSG00000157613 | CREB3L1 |
| ILF3 | ENSG00000265542 | RP11-60A24.3 |
| ILF3 | ENSG00000262099 | CTC-524C5.5 |
| ILF3 | ENSG00000200972 | RNU5A-8P |
| ILF3 | ENSG00000064393 | HIPK2 |
| ILF3 | ENSG00000254542 | NAV2-AS3 |
| ILF3 | ENSG00000100345 | MYH9 |
| ILF3 | ENSG00000134352 | IL6ST |
| ILF3 | ENSG00000182378 | PLCXD1 |
| ILF3 | ENSG00000143257 | NR1I3 |
| ILF3 | ENSG00000256843 | RP11-467L13.4 |
| ILF3 | ENSG00000129460 | NGDN |
| ILF3 | ENSG00000271938 | RP11-589C21.6 |
| ILF3 | ENSG00000260114 | CTD- |
| ILF3 | ENSG00000181458 | 2574D22.4  TMEM45A |
| ILF3 | ENSG00000274750 | HIST1H3E |
| ILF3 | ENSG00000155876 | RRAGA |
| ILF3 | ENSG00000178904 | DPY19L3 |
| ILF3 | ENSG00000106617 | PRKAG2 |
| ILF3 | ENSG00000203814 | HIST2H2BF |
| ILF3 | ENSG00000115561 | CHMP3 |
| ILF3 | ENSG00000227398 | KIF9-AS1 |
| ILF3 | ENSG00000236391 | AC092573.2 |
| ILF3 | ENSG00000175793 | SFN |
| ILF3 | ENSG00000134853 | PDGFRA |
| ILF3 | ENSG00000267679 | EIF5AP2 |
| ILF3 | ENSG00000072274 | TFRC |
| ILF3 | ENSG00000273542 | HIST1H4K |
| ILF3 | ENSG00000207839 | MIR33B |
| ILF3 | ENSG00000229738 | SNRNP27 |
| ILF3 | ENSG00000104903 | LYL1 |
| ILF3 | ENSG00000120616 | EPC1 |

| ILF3 | ENSG00000176170 | SPHK1 |
| --- | --- | --- |
| ILF3 | ENSG00000178773 | CPNE7 |
| ILF3 | ENSG00000214413 | BBIP1 |
| ILF3 | ENSG00000179168 | GGN |
| ILF3 | ENSG00000231794 | AC009542.2 |
| ILF3 | ENSG00000123609 | NMI |
| ILF3 | ENSG00000273063 | RP11-334G22.1 |
| ILF3 | ENSG00000206813 | Y_RNA |
| ILF3 | ENSG00000120217 | CD274 |
| ILF3 | ENSG00000277182 | CTB-58E17.5 |
| ILF3 | ENSG00000111254 | AKAP3 |
| ILF3 | ENSG00000174574 | AKIRIN1 |
| ILF3 | ENSG00000139641 | ESYT1 |
| ILF3 | ENSG00000008128 | CDK11A |
| ILF3 | ENSG00000169896 | ITGAM |
| ILF3 | ENSG00000238901 | snoU13 |
| ILF3 | ENSG00000188425 | NANOS2 |
| ILF3 | ENSG00000146556 | WASH2P |
| ILF3 | ENSG00000197497 | ZNF665 |
| ILF3 | ENSG00000274481 | RP11-345P4.11 |
| ILF3 | ENSG00000128708 | HAT1 |
| ILF3 | ENSG00000271969 | U47924.29 |
| ILF3 | ENSG00000150961 | SEC24D |
| ILF3 | ENSG00000123728 | RAP2C |
| ILF3 | ENSG00000232533 | AC093673.5 |
| ILF3 | ENSG00000108854 | SMURF2 |
| ILF3 | ENSG00000201818 | RNY4P17 |
| ILF3 | ENSG00000254221 | PCDHGB1 |
| ILF3 | ENSG00000204531 | POU5F1 |
| ILF3 | ENSG00000255152 | MSH5- SAPCD1 |
| ILF3 | ENSG00000178927 | C17orf62 |
| ILF3 | ENSG00000100056 | DGCR14 |
| ILF3 | ENSG00000187583 | PLEKHN1 |
| ILF3 | ENSG00000172724 | CCL19 |
| ILF3 | ENSG00000151746 | BICD1 |
| ILF3 | ENSG00000141385 | AFG3L2 |
| ILF3 | ENSG00000147162 | OGT |
| ILF3 | ENSG00000074181 | NOTCH3 |
| ILF3 | ENSG00000107443 | CCNJ |
| ILF3 | ENSG00000228369 | TXNDC12-AS1 |
| ILF3 | ENSG00000110274 | CEP164 |
| ILF3 | ENSG00000267532 | MIR497HG |
| ILF3 | ENSG00000168291 | PDHB |
| ILF3 | ENSG00000253744 | AC025442.3 |
| ILF3 | ENSG00000215908 | CROCCP2 |
| ILF3 | ENSG00000159840 | ZYX |
| ILF3 | ENSG00000151414 | NEK7 |
| ILF3 | ENSG00000255135 | RP11-111M22.3 |
| ILF3 | ENSG00000254894 | NAV2-AS1 |
| ILF3 | ENSG00000254545 | RP11-84A19.3 |
| ILF3 | ENSG00000221953 | C1orf229 |

| ILF3 | ENSG00000222438 | RNU6-1077P |
| --- | --- | --- |
| ILF3 | ENSG00000272801 | RP1-170O19.23 |
| ILF3 | ENSG00000147133 | TAF1 |
| ILF3 | ENSG00000254512 | RP11-472I20.2 |
| ILF3 | ENSG00000205864 | KRTAP5-6 |
| ILF3 | ENSG00000143776 | CDC42BPA |
| ILF3 | ENSG00000107140 | TESK1 |
| ILF3 | ENSG00000135541 | AHI1 |
| ILF3 | ENSG00000205531 | NAP1L4 |
| ILF3 | ENSG00000239099 | RNU7-23P |
| ILF3 | ENSG00000185972 | CCIN |
| ILF3 | ENSG00000256940 | RP11-783K16.5 |
| ILF3 | ENSG00000115211 | EIF2B4 |
| ILF3 | ENSG00000143994 | ABHD1 |
| ILF3 | ENSG00000230231 | FMO7P |
| ILF3 | ENSG00000252744 | Y_RNA |
| ILF3 | ENSG00000121270 | ABCC11 |
| ILF3 | ENSG00000143774 | GUK1 |
| ILF3 | ENSG00000108433 | GOSR2 |
| ILF3 | ENSG00000213903 | LTB4R |
| ILF3 | ENSG00000264964 | RP11-888D10.3 |
| ILF3 | ENSG00000142599 | RERE |
| ILF3 | ENSG00000263669 | RN7SL470P |
| ILF3 | ENSG00000101266 | CSNK2A1 |
| ILF3 | ENSG00000107317 | PTGDS |
| ILF3 | ENSG00000115183 | TANC1 |
| ILF3 | ENSG00000136143 | SUCLA2 |
| ILF3 | ENSG00000185105 | MYADML2 |
| ILF3 | ENSG00000248714 | RP11-1079K10.3 |
| ILF3 | ENSG00000134153 | EMC7 |
| ILF3 | ENSG00000125965 | GDF5 |
| ILF3 | ENSG00000179292 | TMEM151A |
| ILF3 | ENSG00000227484 | XX-C283C717.1 |
| ILF3 | ENSG00000207606 | MIR554 |
| ILF3 | ENSG00000064995 | TAF11 |
| ILF3 | ENSG00000090924 | PLEKHG2 |
| ILF3 | ENSG00000170439 | METTL7B |
| ILF3 | ENSG00000272991 | AF129408.17 |
| ILF3 | ENSG00000145860 | RNF145 |
| ILF3 | ENSG00000143315 | PIGM |
| ILF3 | ENSG00000105819 | PMPCB |
| ILF3 | ENSG00000181467 | RAP2B |
| ILF3 | ENSG00000271737 | CTB-113I20.2 |
| ILF3 | ENSG00000162585 | C1orf86 |
| ILF3 | ENSG00000181029 | TRAPPC5 |
| ILF3 | ENSG00000141562 | NARF |
| ILF3 | ENSG00000266677 | RP11-258F1.1 |
| ILF3 | ENSG00000178826 | TMEM139 |
| ILF3 | ENSG00000168427 | KLHL30 |
| ILF3 | ENSG00000275146 | snoU2_19 |
| ILF3 | ENSG00000214765 | SEPT7P2 |

| ILF3 | ENSG00000182117 | NOP10 |
| --- | --- | --- |
| ILF3 | ENSG00000011295 | TTC19 |
| ILF3 | ENSG00000149564 | ESAM |
| ILF3 | ENSG00000108733 | PEX12 |
| ILF3 | ENSG00000109906 | ZBTB16 |
| ILF3 | ENSG00000175895 | PLEKHF2 |
| ILF3 | ENSG00000085721 | RRN3 |
| ILF3 | ENSG00000203650 | LINC01285 |
| ILF3 | ENSG00000125522 | NPBWR2 |
| ILF3 | ENSG00000244731 | C4A |
| ILF3 | ENSG00000225313 | RP11-415J8.3 |
| ILF3 | ENSG00000205116 | TMEM88B |
| ILF3 | ENSG00000242390 | RPL6P9 |
| ILF3 | ENSG00000204625 | HCG9 |
| ILF3 | ENSG00000181904 | C5orf24 |
| ILF3 | ENSG00000236545 | AP001619.3 |
| ILF3 | ENSG00000164347 | GFM2 |
| ILF3 | ENSG00000140993 | TIGD7 |
| ILF3 | ENSG00000259488 | RP11-154J22.1 |
| ILF3 | ENSG00000112293 | GPLD1 |
| ILF3 | ENSG00000198964 | SGMS1 |
| ILF3 | ENSG00000204420 | C6orf25 |
| ILF3 | ENSG00000263412 | RP5-890E16.2 |
| ILF3 | ENSG00000221552 | MIR1303 |
| ILF3 | ENSG00000240661 | RP11-174O3.3 |
| ILF3 | ENSG00000113300 | CNOT6 |
| ILF3 | ENSG00000278540 | ACACA |
| ILF3 | ENSG00000251364 | CTD-2516F10.2 |
| ILF3 | ENSG00000169989 | TIGD4 |
| ILF3 | ENSG00000166845 | C18orf54 |
| ILF3 | ENSG00000138180 | CEP55 |
| ILF3 | ENSG00000234741 | GAS5 |
| ILF3 | ENSG00000105866 | SP4 |
| ILF3 | ENSG00000185215 | TNFAIP2 |
| ILF3 | ENSG00000140451 | PIF1 |
| ILF3 | ENSG00000198931 | APRT |
| ILF3 | ENSG00000106333 | PCOLCE |
| ILF3 | ENSG00000277880 | TRBV17 |
| ILF3 | ENSG00000124313 | IQSEC2 |
| ILF3 | ENSG00000230753 | ZNF341-AS1 |
| ILF3 | ENSG00000236305 | RP11-126L15.4 |
| ILF3 | ENSG00000163930 | BAP1 |
| ILF3 | ENSG00000267493 | CIRBP-AS1 |
| ILF3 | ENSG00000164463 | CREBRF |
| ILF3 | ENSG00000275902 | RP13-1032I1.11 |
| ILF3 | ENSG00000258450 | RP11-649E7.7 |
| ILF3 | ENSG00000159247 | TUBBP5 |
| ILF3 | ENSG00000007516 | BAIAP3 |
| ILF3 | ENSG00000145014 | TMEM44 |
| ILF3 | ENSG00000174004 | NRROS |
| ILF3 | ENSG00000248593 | DSTNP2 |

| ILF3 | ENSG00000124795 | DEK |
| --- | --- | --- |
| ILF3 | ENSG00000168778 | TCTN2 |
| ILF3 | ENSG00000274713 | MIR7974 |
| ILF3 | ENSG00000170222 | ADPRM |
| ILF3 | ENSG00000187605 | TET3 |
| ILF3 | ENSG00000206622 | SNORA69 |
| ILF3 | ENSG00000221420 | SNORA81 |
| ILF3 | ENSG00000139631 | CSAD |
| ILF3 | ENSG00000232734 | ATP5G1P7 |
| ILF3 | ENSG00000139180 | NDUFA9 |
| ILF3 | ENSG00000205084 | TMEM231 |
| ILF3 | ENSG00000240698 | RPS2P39 |
| ILF3 | ENSG00000133519 | ZDHHC8P1 |
| ILF3 | ENSG00000236165 | PRADC1P1 |
| ILF3 | ENSG00000188878 | FBF1 |
| ILF3 | ENSG00000131791 | PRKAB2 |
| ILF3 | ENSG00000172613 | RAD9A |
| ILF3 | ENSG00000196586 | MYO6 |
| ILF3 | ENSG00000277437 | MIR3687-1 |
| ILF3 | ENSG00000245970 | SNORA72 |
| ILF3 | ENSG00000223663 | RP5-890O3.3 |
| ILF3 | ENSG00000239593 | RP11-477J21.6 |
| ILF3 | ENSG00000232208 | RP3-477M7.5 |
| ILF3 | ENSG00000115602 | IL1RL1 |
| ILF3 | ENSG00000275713 | HIST1H2BH |
| ILF3 | ENSG00000204323 | SMIM5 |
| ILF3 | ENSG00000124422 | USP22 |
| ILF3 | ENSG00000132819 | RBM38 |
| ILF3 | ENSG00000188827 | SLX4 |
| ILF3 | ENSG00000156795 | WDYHV1 |
| ILF3 | ENSG00000261449 | RP11-589C21.5 |
| ILF3 | ENSG00000253921 | CTB-113P19.3 |
| ILF3 | ENSG00000241030 | RPL29P24 |
| ILF3 | ENSG00000139174 | PRICKLE1 |
| ILF3 | ENSG00000267289 | CTD- |
| ILF3 | ENSG00000204428 | 2623N2.11  LY6G5C |
| ILF3 | ENSG00000131115 | ZNF227 |
| ILF3 | ENSG00000130475 | FCHO1 |
| ILF3 | ENSG00000204822 | MRPL53 |
| ILF3 | ENSG00000073146 | MOV10L1 |
| ILF3 | ENSG00000212567 | SNORA57 |
| ILF3 | ENSG00000156453 | PCDH1 |
| ILF3 | ENSG00000268615 | RP11-65J3.15 |
| ILF3 | ENSG00000197021 | CXorf40B |
| ILF3 | ENSG00000173171 | MTX1 |
| ILF3 | ENSG00000117682 | DHDDS |
| ILF3 | ENSG00000166575 | TMEM135 |
| ILF3 | ENSG00000235119 | RP11-9M16.2 |
| ILF3 | ENSG00000249743 | RP11-60A8.1 |
| ILF3 | ENSG00000116685 | KIAA2013 |
| ILF3 | ENSG00000139842 | CUL4A |

| ILF3 | ENSG00000198040 | ZNF84 |
| --- | --- | --- |
| ILF3 | ENSG00000138777 | PPA2 |
| ILF3 | ENSG00000269019 | AC005932.1 |
| ILF3 | ENSG00000276027 | U12 |
| ILF3 | ENSG00000214456 | PLIN5 |
| ILF3 | ENSG00000139154 | AEBP2 |
| ILF3 | ENSG00000102471 | NDFIP2 |
| ILF3 | ENSG00000252962 | RNU6-993P |
| ILF3 | ENSG00000264653 | MIR5194 |
| ILF3 | ENSG00000203546 | RP11-176H8.1 |
| ILF3 | ENSG00000260750 | RP11-482M8.1 |
| ILF3 | ENSG00000153446 | C16orf89 |
| ILF3 | ENSG00000207297 | SNORD7 |
| ILF3 | ENSG00000103319 | EEF2K |
| ILF3 | ENSG00000276185 | TP53TG1_2 |
| ILF3 | ENSG00000271488 | RBM11P1 |
| ILF3 | ENSG00000162775 | RBM15 |
| ILF3 | ENSG00000259884 | RP11-1100L3.8 |
| ILF3 | ENSG00000168556 | ING2 |
| ILF3 | ENSG00000269653 | CTB-102L5.7 |
| ILF3 | ENSG00000100865 | CINP |
| ILF3 | ENSG00000241404 | EGFL8 |
| ILF3 | ENSG00000131584 | ACAP3 |
| ILF3 | ENSG00000205707 | LYRM5 |
| ILF3 | ENSG00000166483 | WEE1 |
| ILF3 | ENSG00000200059 | Y_RNA |
| ILF3 | ENSG00000106305 | AIMP2 |
| ILF3 | ENSG00000119314 | PTBP3 |
| ILF3 | ENSG00000269737 | RP11-345P4.7 |
| ILF3 | ENSG00000207713 | MIR200C |
| ILF3 | ENSG00000176261 | ZBTB8OS |
| ILF3 | ENSG00000141556 | TBCD |
| ILF3 | ENSG00000139132 | FGD4 |
| ILF3 | ENSG00000169957 | ZNF768 |
| ILF3 | ENSG00000231887 | PRH1 |
| ILF3 | ENSG00000267047 | RP11-589P10.7 |
| ILF3 | ENSG00000185495 | SEPT7P9 |
| ILF3 | ENSG00000256040 | PAPPA-AS1 |
| ILF3 | ENSG00000126254 | RBM42 |
| ILF3 | ENSG00000275651 | MIR6851 |
| ILF3 | ENSG00000239264 | TXNDC5 |
| ILF3 | ENSG00000100038 | TOP3B |
| ILF3 | ENSG00000274051 | uc_338 |
| ILF3 | ENSG00000101181 | MTG2 |
| ILF3 | ENSG00000267387 | CTD-2240E14.4 |
| ILF3 | ENSG00000255036 | RP11-23J9.4 |
| ILF3 | ENSG00000134575 | ACP2 |
| ILF3 | ENSG00000230686 | AC067945.2 |
| ILF3 | ENSG00000200176 | RNU1-19P |
| ILF3 | ENSG00000077721 | UBE2A |
| ILF3 | ENSG00000235145 | RP11-533E19.3 |

| ILF3 | ENSG00000268120 | CTD- |
| --- | --- | --- |
| ILF3 | ENSG00000111605 | 3193O13.11  CPSF6 |
| ILF3 | ENSG00000233791 | LINC01136 |
| ILF3 | ENSG00000168056 | LTBP3 |
| ILF3 | ENSG00000154874 | CCDC144B |
| ILF3 | ENSG00000267729 | AC010761.14 |
| ILF3 | ENSG00000224856 | RP4-796I17.5 |
| ILF3 | ENSG00000209702 | SNORD41 |
| ILF3 | ENSG00000261067 | RP11-264B17.3 |
| ILF3 | ENSG00000220785 | MTMR9LP |
| ILF3 | ENSG00000060971 | ACAA1 |
| ILF3 | ENSG00000230228 | RP11-4M23.4 |
| ILF3 | ENSG00000130165 | ELOF1 |
| ILF3 | ENSG00000256407 | RP11-446E24.4 |
| ILF3 | ENSG00000268996 | MAN1B1-AS1 |
| ILF3 | ENSG00000145979 | TBC1D7 |
| ILF3 | ENSG00000143627 | PKLR |
| ILF3 | ENSG00000272512 | RP11-54O7.17 |
| ILF3 | ENSG00000187954 | CYHR1 |
| ILF3 | ENSG00000100209 | HSCB |
| ILF3 | ENSG00000217094 | PPIAP31 |
| ILF3 | ENSG00000172460 | PRSS30P |
| ILF3 | ENSG00000105619 | TFPT |
| ILF3 | ENSG00000230532 | AC091133.1 |
| ILF3 | ENSG00000223725 | AC007879.5 |
| ILF3 | ENSG00000104738 | MCM4 |
| ILF3 | ENSG00000222046 | DCDC2B |
| ILF3 | ENSG00000113739 | STC2 |
| ILF3 | ENSG00000157106 | SMG1 |
| ILF3 | ENSG00000159202 | UBE2Z |
| ILF3 | ENSG00000085185 | BCORL1 |
| ILF3 | ENSG00000270330 | RP5-892F13.2 |
| ILF3 | ENSG00000226427 | HMGN2P7 |
| ILF3 | ENSG00000181274 | FRAT2 |
| ILF3 | ENSG00000166710 | B2M |
| ILF3 | ENSG00000167642 | SPINT2 |
| ILF3 | ENSG00000162086 | ZNF75A |
| ILF3 | ENSG00000213782 | DDX47 |
| ILF3 | ENSG00000119772 | DNMT3A |
| ILF3 | ENSG00000154813 | DPH3 |
| ILF3 | ENSG00000159216 | RUNX1 |
| ILF3 | ENSG00000277872 | MIR6817 |
| ILF3 | ENSG00000239137 | snoU13 |
| ILF3 | ENSG00000267576 | CTC-510F12.6 |
| ILF3 | ENSG00000214026 | MRPL23 |
| ILF3 | ENSG00000104219 | ZDHHC2 |
| ILF3 | ENSG00000083444 | PLOD1 |
| ILF3 | ENSG00000231113 | RP3-475N16.1 |
| ILF3 | ENSG00000182272 | B4GALNT4 |
| ILF3 | ENSG00000071894 | CPSF1 |
| ILF3 | ENSG00000133104 | SPG20 |

| ILF3 | ENSG00000252916 | RNU6-762P |
| --- | --- | --- |
| ILF3 | ENSG00000277532 | CTD-2319I12.9 |
| ILF3 | ENSG00000266401 | RP11-874J12.4 |
| ILF3 | ENSG00000270361 | RP11-307C12.13 |
| ILF3 | ENSG00000258604 | AL161668.5 |
| ILF3 | ENSG00000133067 | LGR6 |
| ILF3 | ENSG00000141873 | SLC39A3 |
| ILF3 | ENSG00000279667 | RP11-656D10.7 |
| ILF3 | ENSG00000154359 | LONRF1 |
| ILF3 | ENSG00000196218 | RYR1 |
| ILF3 | ENSG00000144061 | NPHP1 |
| ILF3 | ENSG00000246089 | RP11-115C21.2 |
| ILF3 | ENSG00000229951 | FLJ31356 |
| ILF3 | ENSG00000087302 | C14orf166 |
| ILF3 | ENSG00000262039 | RP11-81K2.1 |
| ILF3 | ENSG00000238236 | RP4-534P7.2 |
| ILF3 | ENSG00000265008 | RP11-53I6.3 |
| ILF3 | ENSG00000263690 | AC011591.1 |
| ILF3 | ENSG00000232352 | SEMA3B-AS1 |
| ILF3 | ENSG00000198464 | ZNF480 |
| ILF3 | ENSG00000254003 | CTB-167B5.1 |
| ILF3 | ENSG00000115998 | C2orf42 |
| ILF3 | ENSG00000104365 | IKBKB |
| ILF3 | ENSG00000245975 | RP11-30K9.6 |
| ILF3 | ENSG00000080822 | CLDND1 |
| ILF3 | ENSG00000186416 | NKRF |
| ILF3 | ENSG00000126698 | DNAJC8 |
| ILF3 | ENSG00000206637 | SNORA70 |
| ILF3 | ENSG00000230147 | RP11-305L7.5 |
| ILF3 | ENSG00000006715 | VPS41 |
| ILF3 | ENSG00000262484 | CCER2 |
| ILF3 | ENSG00000232457 | SLC16A6P1 |
| ILF3 | ENSG00000239705 | RP11-65N13.8 |
| ILF3 | ENSG00000108448 | TRIM16L |
| ILF3 | ENSG00000250939 | AC034198.7 |
| ILF3 | ENSG00000202528 | RNU6-650P |
| ILF3 | ENSG00000131013 | PPIL4 |
| ILF3 | ENSG00000260545 | CTB-134F13.1 |
| ILF3 | ENSG00000115084 | SLC35F5 |
| ILF3 | ENSG00000262171 | CTA-972D3.2 |
| ILF3 | ENSG00000114686 | MRPL3 |
| ILF3 | ENSG00000079462 | PAFAH1B3 |
| ILF3 | ENSG00000266469 | CTB-131K11.1 |
| ILF3 | ENSG00000235423 | RP11-282O18.3 |
| ILF3 | ENSG00000142632 | ARHGEF19 |
| ILF3 | ENSG00000225518 | RP11-396C23.2 |
| ILF3 | ENSG00000021826 | CPS1 |
| ILF3 | ENSG00000152944 | MED21 |
| ILF3 | ENSG00000130755 | GMFG |
| ILF3 | ENSG00000197444 | OGDHL |
| ILF3 | ENSG00000160685 | ZBTB7B |

| ILF3 | ENSG00000227048 | RP11-134G8.2 |
| --- | --- | --- |
| ILF3 | ENSG00000172889 | EGFL7 |
| ILF3 | ENSG00000073060 | SCARB1 |
| ILF3 | ENSG00000167986 | DDB1 |
| ILF3 | ENSG00000091317 | CMTM6 |
| ILF3 | ENSG00000237886 | MIR4674HG |
| ILF3 | ENSG00000172053 | QARS |
| ILF3 | ENSG00000025772 | TOMM34 |
| ILF3 | ENSG00000141258 | SGSM2 |
| ILF3 | ENSG00000159640 | ACE |
| ILF3 | ENSG00000199971 | RNU6-797P |
| ILF3 | ENSG00000112208 | BAG2 |
| ILF3 | ENSG00000231384 | AC007919.19 |
| ILF3 | ENSG00000130726 | TRIM28 |
| ILF3 | ENSG00000143653 | SCCPDH |
| ILF3 | ENSG00000207365 | Y_RNA |
| ILF3 | ENSG00000105290 | APLP1 |
| ILF3 | ENSG00000089692 | LAG3 |
| ILF3 | ENSG00000228343 | RP11-1148L6.5 |
| ILF3 | ENSG00000142959 | BEST4 |
| ILF3 | ENSG00000256576 | RP13-977J11.2 |
| ILF3 | ENSG00000258388 | PPT2-EGFL8 |
| ILF3 | ENSG00000175787 | ZNF169 |
| ILF3 | ENSG00000173464 | RNASE11 |
| ILF3 | ENSG00000197608 | ZNF841 |
| ILF3 | ENSG00000277458 | RP11-166B2.9 |
| ILF3 | ENSG00000275208 | MIR6745 |
| ILF3 | ENSG00000215424 | MCM3AP-AS1 |
| ILF3 | ENSG00000167112 | TRUB2 |
| ILF3 | ENSG00000020577 | SAMD4A |
| ILF3 | ENSG00000133250 | ZNF414 |
| ILF3 | ENSG00000269892 | RP4-761J14.10 |
| ILF3 | ENSG00000249978 | TRGV7 |
| ILF3 | ENSG00000268475 | CTC-435M10.6 |
| ILF3 | ENSG00000109832 | DDX25 |
| ILF3 | ENSG00000143401 | ANP32E |
| ILF3 | ENSG00000013523 | ANGEL1 |
| ILF3 | ENSG00000272226 | RP11-63G10.3 |
| ILF3 | ENSG00000101444 | AHCY |
| ILF3 | ENSG00000163389 | POGLUT1 |
| ILF3 | ENSG00000273375 | RP11-803P9.1 |
| ILF3 | ENSG00000144791 | LIMD1 |
| ILF3 | ENSG00000126062 | TMEM115 |
| ILF3 | ENSG00000213683 | AC002056.3 |
| ILF3 | ENSG00000156510 | HKDC1 |
| ILF3 | ENSG00000005469 | CROT |
| ILF3 | ENSG00000169951 | ZNF764 |
| ILF3 | ENSG00000117643 | MAN1C1 |
| ILF3 | ENSG00000147144 | CCDC120 |
| ILF3 | ENSG00000173517 | PEAK1 |
| ILF3 | ENSG00000172339 | ALG14 |

| ILF3 | ENSG00000099282 | TSPAN15 |
| --- | --- | --- |
| ILF3 | ENSG00000154144 | TBRG1 |
| ILF3 | ENSG00000154269 | ENPP3 |
| ILF3 | ENSG00000273890 | RP11-603J24.21 |
| ILF3 | ENSG00000207496 | SNORA7A |
| ILF3 | ENSG00000168710 | AHCYL1 |
| ILF3 | ENSG00000260541 | LA16c-429E7.1 |
| ILF3 | ENSG00000156873 | PHKG2 |
| ILF3 | ENSG00000196504 | PRPF40A |
| ILF3 | ENSG00000113600 | C9 |
| ILF3 | ENSG00000275111 | ZNF2 |
| ILF3 | ENSG00000217644 | RP4-803A2.1 |
| ILF3 | ENSG00000178814 | OPLAH |
| ILF3 | ENSG00000121741 | ZMYM2 |
| ILF3 | ENSG00000229180 | GS1-124K5.11 |
| ILF3 | ENSG00000233485 | RP3-467K16.2 |
| ILF3 | ENSG00000186575 | NF2 |
| ILF3 | ENSG00000108960 | MMD |
| ILF3 | ENSG00000132386 | SERPINF1 |
| ILF3 | ENSG00000278828 | HIST1H3H |
| ILF3 | ENSG00000274292 | RP11-347I19.7 |
| ILF3 | ENSG00000274621 | MIR6867 |
| ILF3 | ENSG00000227845 | RP11-184B22.2 |
| ILF3 | ENSG00000164081 | TEX264 |
| ILF3 | ENSG00000070985 | TRPM5 |
| ILF3 | ENSG00000277925 | Telomerase-vert |
| ILF3 | ENSG00000269102 | CTD-2525I3.5 |
| ILF3 | ENSG00000101109 | STK4 |
| ILF3 | ENSG00000275221 | HIST1H2AK |
| ILF3 | ENSG00000196387 | ZNF140 |
| ILF3 | ENSG00000168385 | SEPT2 |
| ILF3 | ENSG00000230721 | RP4-612B15.2 |
| ILF3 | ENSG00000275107 | MIR6782 |
| ILF3 | ENSG00000112357 | PEX7 |
| ILF3 | ENSG00000205664 | RP11-706O15.1 |
| ILF3 | ENSG00000099246 | RAB18 |
| ILF3 | ENSG00000158716 | DUSP23 |
| ILF3 | ENSG00000143319 | ISG20L2 |
| ILF3 | ENSG00000106246 | PTCD1 |
| ILF3 | ENSG00000279491 | RP11-810P12.7 |
| ILF3 | ENSG00000257595 | RP3-473L9.4 |
| ILF3 | ENSG00000188725 | SMIM15 |
| ILF3 | ENSG00000250214 | RP13-488H8.1 |
| ILF3 | ENSG00000187193 | MT1X |
| ILF3 | ENSG00000196313 | POM121 |
| ILF3 | ENSG00000133816 | MICAL2 |
| ILF3 | ENSG00000257913 | RP11-386G11.5 |
| ILF3 | ENSG00000169418 | NPR1 |
| ILF3 | ENSG00000198589 | LRBA |
| ILF3 | ENSG00000156273 | BACH1 |
| ILF3 | ENSG00000248235 | AC037459.4 |

| ILF3 | ENSG00000099377 | HSD3B7 |
| --- | --- | --- |
| ILF3 | ENSG00000168268 | NT5DC2 |
| ILF3 | ENSG00000230068 | CDC42-IT1 |
| ILF3 | ENSG00000121931 | LRIF1 |
| ILF3 | ENSG00000134755 | DSC2 |
| ILF3 | ENSG00000255089 | RP11-326C3.10 |
| ILF3 | ENSG00000252759 | Y_RNA |
| ILF3 | ENSG00000101290 | CDS2 |
| ILF3 | ENSG00000272247 | RP11-379F4.9 |
| ILF3 | ENSG00000064042 | LIMCH1 |
| ILF3 | ENSG00000152253 | SPC25 |
| ILF3 | ENSG00000227963 | RP5-1074L1.1 |
| ILF3 | ENSG00000162852 | CNST |
| ILF3 | ENSG00000253210 | RP11-809O17.1 |
| ILF3 | ENSG00000159147 | DONSON |
| ILF3 | ENSG00000172667 | ZMAT3 |
| ILF3 | ENSG00000255062 | RP11-712L6.5 |
| ILF3 | ENSG00000145476 | CYP4V2 |
| ILF3 | ENSG00000122643 | NT5C3A |
| ILF3 | ENSG00000115239 | GPR75-ASB3 |
| ILF3 | ENSG00000162972 | C2orf47 |
| ILF3 | ENSG00000146909 | NOM1 |
| ILF3 | ENSG00000279669 | RP11-717F1.1 |
| ILF3 | ENSG00000255507 | RP11-535A19.2 |
| ILF3 | ENSG00000255390 | RP11-732A19.5 |
| ILF3 | ENSG00000135838 | NPL |
| ILF3 | ENSG00000272783 | RP13-1016M1.2 |
| ILF3 | ENSG00000088002 | SULT2B1 |
| ILF3 | ENSG00000279520 | RP11-20I23.2 |
| ILF3 | ENSG00000161992 | PRR35 |
| ILF3 | ENSG00000144395 | CCDC150 |
| ILF3 | ENSG00000201778 | Y_RNA |
| ILF3 | ENSG00000204054 | LINC00963 |
| ILF3 | ENSG00000278009 | RP11-649E7.8 |
| ILF3 | ENSG00000147604 | RPL7 |
| ILF3 | ENSG00000108590 | MED31 |
| ILF3 | ENSG00000004961 | HCCS |
| ILF3 | ENSG00000035928 | RFC1 |
| ILF3 | ENSG00000142208 | AKT1 |
| ILF3 | ENSG00000177406 | RP11-218M22.1 |
| ILF3 | ENSG00000146232 | NFKBIE |
| ILF3 | ENSG00000253005 | RNU6-176P |
| ILF3 | ENSG00000162813 | BPNT1 |
| ILF3 | ENSG00000272402 | RP1-30M3.6 |
| ILF3 | ENSG00000263974 | RN7SL121P |
| ILF3 | ENSG00000264757 | MIR3198-1 |
| ILF3 | ENSG00000273950 | Metazoa_SRP |
| ILF3 | ENSG00000185201 | IFITM2 |
| ILF3 | ENSG00000228887 | EEF1DP1 |
| ILF3 | ENSG00000142166 | IFNAR1 |
| ILF3 | ENSG00000100124 | ANKRD54 |

| ILF3 | ENSG00000255328 | RP11-326C3.12 |
| --- | --- | --- |
| ILF3 | ENSG00000109046 | WSB1 |
| ILF3 | ENSG00000104671 | DCTN6 |
| ILF3 | ENSG00000279691 | RP11-485M7.2 |
| ILF3 | ENSG00000258867 | LINC01146 |
| ILF3 | ENSG00000127419 | TMEM175 |
| ILF3 | ENSG00000181444 | ZNF467 |
| ILF3 | ENSG00000005189 | AC004381.6 |
| ILF3 | ENSG00000243243 | AC073130.3 |
| ILF3 | ENSG00000145217 | SLC26A1 |
| ILF3 | ENSG00000038427 | VCAN |
| ILF3 | ENSG00000064547 | LPAR2 |
| ILF3 | ENSG00000175110 | MRPS22 |
| ILF3 | ENSG00000267791 | CTD-2659N19.2 |
| ILF3 | ENSG00000130175 | PRKCSH |
| ILF3 | ENSG00000164574 | GALNT10 |
| ILF3 | ENSG00000188177 | ZC3H6 |
| ILF3 | ENSG00000133706 | LARS |
| ILF3 | ENSG00000144504 | ANKMY1 |
| ILF3 | ENSG00000133835 | HSD17B4 |
| ILF3 | ENSG00000128567 | PODXL |
| ILF3 | ENSG00000219665 | CTD-2006C1.2 |
| ILF3 | ENSG00000236817 | RP11-978I15.10 |
| ILF3 | ENSG00000088682 | COQ9 |
| ILF3 | ENSG00000245614 | DDX11-AS1 |
| ILF3 | ENSG00000258984 | UBE2F-SCLY |
| ILF3 | ENSG00000214960 | ISPD |
| ILF3 | ENSG00000055118 | KCNH2 |
| ILF3 | ENSG00000042493 | CAPG |
| ILF3 | ENSG00000074603 | DPP8 |
| ILF3 | ENSG00000248092 | NNT-AS1 |
| ILF3 | ENSG00000265496 | MIR1539 |
| ILF3 | ENSG00000136925 | TSTD2 |
| ILF3 | ENSG00000129465 | RIPK3 |
| ILF3 | ENSG00000277073 | MIR6131 |
| ILF3 | ENSG00000166153 | DEPDC4 |
| ILF3 | ENSG00000252929 | RNU6-218P |
| ILF3 | ENSG00000156261 | CCT8 |
| ILF3 | ENSG00000110074 | FOXRED1 |
| ILF3 | ENSG00000197746 | PSAP |
| ILF3 | ENSG00000135636 | DYSF |
| ILF3 | ENSG00000137133 | HINT2 |
| ILF3 | ENSG00000183597 | TANGO2 |
| ILF3 | ENSG00000204422 | XXbac- |
| ILF3 | ENSG00000156802 | BPG32J3.20 ATAD2 |
| ILF3 | ENSG00000279044 | AC007787.3 |
| ILF3 | ENSG00000205559 | CHKB-AS1 |
| ILF3 | ENSG00000116039 | ATP6V1B1 |
| ILF3 | ENSG00000135736 | CCDC102A |
| ILF3 | ENSG00000164045 | CDC25A |
| ILF3 | ENSG00000250903 | GMDS-AS1 |

| ILF3 | ENSG00000131019 | ULBP3 |
| --- | --- | --- |
| ILF3 | ENSG00000161016 | RPL8 |
| ILF3 | ENSG00000269540 | CTD-2126E3.5 |
| ILF3 | ENSG00000278658 | MIR6826 |
| ILF3 | ENSG00000226419 | SLC16A1-AS1 |
| ILF3 | ENSG00000278773 | ZNRD1-AS1_3 |
| ILF3 | ENSG00000205659 | LIN52 |
| ILF3 | ENSG00000246225 | RP11-17A1.3 |
| ILF3 | ENSG00000077150 | NFKB2 |
| ILF3 | ENSG00000244268 | RP11-529G21.2 |
| ILF3 | ENSG00000253695 | RP11-177H2.2 |
| ILF3 | ENSG00000153885 | KCTD15 |
| ILF3 | ENSG00000095794 | CREM |
| ILF3 | ENSG00000105711 | SCN1B |
| ILF3 | ENSG00000145919 | BOD1 |
| ILF3 | ENSG00000263403 | MIR4673 |
| ILF3 | ENSG00000205307 | SAP25 |
| ILF3 | ENSG00000184378 | ACTRT3 |
| ILF3 | ENSG00000185332 | TMEM105 |
| ILF3 | ENSG00000137960 | GIPC2 |
| ILF3 | ENSG00000206964 | Y_RNA |
| ILF3 | ENSG00000011485 | PPP5C |
| ILF3 | ENSG00000261396 | CTD-2012K14.2 |
| ILF3 | ENSG00000082701 | GSK3B |
| ILF3 | ENSG00000135778 | NTPCR |
| ILF3 | ENSG00000117616 | RSRP1 |
| ILF3 | ENSG00000170921 | TANC2 |
| ILF3 | ENSG00000237945 | LINC00649 |
| ILF3 | ENSG00000279901 | CTD-2270P14.2 |
| ILF3 | ENSG00000267412 | CTC-265F19.2 |
| ILF3 | ENSG00000130529 | TRPM4 |
| ILF3 | ENSG00000075089 | ACTR6 |
| ILF3 | ENSG00000177427 | MIEF2 |
| ILF3 | ENSG00000173992 | CCS |
| ILF3 | ENSG00000119705 | SLIRP |
| ILF3 | ENSG00000278970 | HEIH |
| ILF3 | ENSG00000235606 | AK4P6 |
| ILF3 | ENSG00000201658 | RNU6-283P |
| ILF3 | ENSG00000124782 | RREB1 |
| ILF3 | ENSG00000119685 | TTLL5 |
| ILF3 | ENSG00000112306 | RPS12 |
| ILF3 | ENSG00000139266 | 9-Mar |
| ILF3 | ENSG00000174672 | BRSK2 |
| ILF3 | ENSG00000257830 | RP11-845M18.7 |
| ILF3 | ENSG00000111186 | WNT5B |
| ILF3 | ENSG00000184785 | SMIM10 |
| ILF3 | ENSG00000234789 | RP11-483H20.4 |
| ILF3 | ENSG00000198546 | ZNF511 |
| ILF3 | ENSG00000199349 | Y_RNA |
| ILF3 | ENSG00000137656 | BUD13 |
| ILF3 | ENSG00000167264 | DUS2 |

| ILF3 | ENSG00000121454 | LHX4 |
| --- | --- | --- |
| ILF3 | ENSG00000139547 | RDH16 |
| ILF3 | ENSG00000271989 | RP4-736L20.3 |
| ILF3 | ENSG00000112659 | CUL9 |
| ILF3 | ENSG00000273291 | RP11-136C24.3 |
| ILF3 | ENSG00000258728 | GALT |
| ILF3 | ENSG00000205277 | MUC12 |
| ILF3 | ENSG00000196381 | ZNF781 |
| ILF3 | ENSG00000272173 | RNU7-1 |
| ILF3 | ENSG00000173327 | MAP3K11 |
| ILF3 | ENSG00000169131 | ZNF354A |
| ILF3 | ENSG00000169635 | HIC2 |
| ILF3 | ENSG00000204666 | FLJ26850 |
| ILF3 | ENSG00000260209 | RP11-680F20.10 |
| ILF3 | ENSG00000251405 | CTB-109A12.1 |
| ILF3 | ENSG00000075188 | NUP37 |
| ILF3 | ENSG00000161920 | MED11 |
| ILF3 | ENSG00000134186 | PRPF38B |
| ILF3 | ENSG00000206941 | SNORD15A |
| ILF3 | ENSG00000189051 | RNF222 |
| ILF3 | ENSG00000214144 | RP11-488L18.1 |
| ILF3 | ENSG00000185022 | MAFF |
| ILF3 | ENSG00000249492 | RP11-159F24.3 |
| ILF3 | ENSG00000139133 | ALG10 |
| ILF3 | ENSG00000132677 | RHBG |
| ILF3 | ENSG00000260868 | RP11-394I13.1 |
| ILF3 | ENSG00000253549 | RP11-317J10.2 |
| ILF3 | ENSG00000124217 | MOCS3 |
| ILF3 | ENSG00000177946 | CENPBD1 |
| ILF3 | ENSG00000107863 | ARHGAP21 |
| ILF3 | ENSG00000203705 | TATDN3 |
| ILF3 | ENSG00000136933 | RABEPK |
| ILF3 | ENSG00000237296 | SMG1P1 |
| ILF3 | ENSG00000171448 | ZBTB26 |
| ILF3 | ENSG00000197535 | MYO5A |
| ILF3 | ENSG00000263923 | RP11-571L19.7 |
| ILF3 | ENSG00000196371 | FUT4 |
| ILF3 | ENSG00000260367 | RP11-264B17.4 |
| ILF3 | ENSG00000178425 | NT5DC1 |
| ILF3 | ENSG00000064607 | SUGP2 |
| ILF3 | ENSG00000264971 | PRR13P4 |
| ILF3 | ENSG00000254740 | RP11-334E6.3 |
| ILF3 | ENSG00000145907 | G3BP1 |
| ILF3 | ENSG00000249740 | OSMR-AS1 |
| ILF3 | ENSG00000109180 | OCIAD1 |
| ILF3 | ENSG00000233396 | RP11-458D21.1 |
| ILF3 | ENSG00000136122 | BORA |
| ILF3 | ENSG00000278050 | NEAT1_2 |
| ILF3 | ENSG00000260000 | RP3-467N11.1 |
| ILF3 | ENSG00000254400 | RP11-732A19.8 |
| ILF3 | ENSG00000125753 | VASP |

| ILF3 | ENSG00000146576 | C7orf26 |
| --- | --- | --- |
| ILF3 | ENSG00000279875 | RP11-474C8.7 |
| ILF3 | ENSG00000103313 | MEFV |
| ILF3 | ENSG00000032444 | PNPLA6 |
| ILF3 | ENSG00000175073 | VCPIP1 |
| ILF3 | ENSG00000255949 | AP003419.16 |
| ILF3 | ENSG00000105193 | RPS16 |
| ILF3 | ENSG00000183718 | TRIM52 |
| ILF3 | ENSG00000259080 | RP11-158I13.2 |
| ILF3 | ENSG00000273355 | RP11-672L10.6 |
| ILF3 | ENSG00000170852 | KBTBD2 |
| ILF3 | ENSG00000110536 | PTPMT1 |
| ILF3 | ENSG00000166145 | SPINT1 |
| ILF3 | ENSG00000140688 | C16orf58 |
| ILF3 | ENSG00000135677 | GNS |
| ILF3 | ENSG00000154358 | OBSCN |
| ILF3 | ENSG00000011454 | RABGAP1 |
| ILF3 | ENSG00000139187 | KLRG1 |
| ILF3 | ENSG00000101166 | SLMO2 |
| ILF3 | ENSG00000100150 | DEPDC5 |
| ILF3 | ENSG00000170899 | GSTA4 |
| ILF3 | ENSG00000271367 | RP3-483K16.4 |
| ILF3 | ENSG00000207448 | RNU6-520P |
| ILF3 | ENSG00000087842 | PIR |
| ILF3 | ENSG00000117450 | PRDX1 |
| ILF3 | ENSG00000162066 | AMDHD2 |
| ILF3 | ENSG00000149531 | FRG1B |
| ILF3 | ENSG00000130822 | PNCK |
| ILF3 | ENSG00000275120 | RP11-182J1.17 |
| ILF3 | ENSG00000137812 | CASC5 |
| ILF3 | ENSG00000089163 | SIRT4 |
| ILF3 | ENSG00000185130 | HIST1H2BL |
| ILF3 | ENSG00000119778 | ATAD2B |
| ILF3 | ENSG00000207340 | RNVU1-10 |
| ILF3 | ENSG00000110367 | DDX6 |
| ILF3 | ENSG00000134899 | ERCC5 |
| ILF3 | ENSG00000204681 | GABBR1 |
| ILF3 | ENSG00000200305 | Y_RNA |
| ILF3 | ENSG00000269119 | HNRNPA1P52 |
| ILF3 | ENSG00000147669 | POLR2K |
| ILF3 | ENSG00000146021 | KLHL3 |
| ILF3 | ENSG00000253848 | RP11-10N23.5 |
| ILF3 | ENSG00000267618 | RAD51L3- |
| ILF3 | ENSG00000228028 | RFFL  AC069257.8 |
| ILF3 | ENSG00000085760 | MTIF2 |
| ILF3 | ENSG00000157240 | FZD1 |
| ILF3 | ENSG00000134717 | BTF3L4 |
| ILF3 | ENSG00000267601 | RP11-323N12.5 |
| ILF3 | ENSG00000212446 | RNU6-131P |
| ILF3 | ENSG00000180198 | RCC1 |
| ILF3 | ENSG00000197372 | ZNF675 |

| ILF3 | ENSG00000232901 | CYCSP10 |
| --- | --- | --- |
| ILF3 | ENSG00000264070 | DND1P1 |
| ILF3 | ENSG00000253092 | SNORA81 |
| ILF3 | ENSG00000003249 | DBNDD1 |
| ILF3 | ENSG00000233366 | ZNF90P2 |
| ILF3 | ENSG00000204389 | HSPA1A |
| ILF3 | ENSG00000207483 | RNU6-1067P |
| ILF3 | ENSG00000212517 | SNORA26 |
| ILF3 | ENSG00000177879 | AP3S1 |
| ILF3 | ENSG00000234537 | RP11-100G15.7 |
| ILF3 | ENSG00000270549 | RP11-293K19.1 |
| ILF3 | ENSG00000171532 | NEUROD2 |
| ILF3 | ENSG00000237463 | RP11-280O1.2 |
| ILF3 | ENSG00000163520 | FBLN2 |
| ILF3 | ENSG00000163590 | PPM1L |
| ILF3 | ENSG00000102900 | NUP93 |
| ILF3 | ENSG00000273473 | LL09NC01- |
| ILF3 | ENSG00000228839 | 139C3.1  PIK3IP1-AS1 |
| ILF3 | ENSG00000163531 | NFASC |
| ILF3 | ENSG00000039068 | CDH1 |
| ILF3 | ENSG00000268889 | CTD-2616J11.14 |
| ILF3 | ENSG00000135845 | PIGC |
| ILF3 | ENSG00000224276 | RP11-336K24.5 |
| ILF3 | ENSG00000224177 | LINC00570 |
| ILF3 | ENSG00000258086 | RP11-753H16.5 |
| ILF3 | ENSG00000221914 | PPP2R2A |
| ILF3 | ENSG00000278412 | MIR6854 |
| ILF3 | ENSG00000152475 | ZNF837 |
| ILF3 | ENSG00000196470 | SIAH1 |
| ILF3 | ENSG00000237757 | EEF1A1P30 |
| ILF3 | ENSG00000272916 | RP11-574K11.31 |
| ILF3 | ENSG00000129255 | MPDU1 |
| ILF3 | ENSG00000163354 | DCST2 |
| ILF3 | ENSG00000099960 | SLC7A4 |
| ILF3 | ENSG00000183666 | GUSBP1 |
| ILF3 | ENSG00000231684 | EIF1P3 |
| ILF3 | ENSG00000140323 | DISP2 |
| ILF3 | ENSG00000198862 | LTN1 |
| ILF3 | ENSG00000163918 | RFC4 |
| ILF3 | ENSG00000263858 | MIR4769 |
| ILF3 | ENSG00000131876 | SNRPA1 |
| ILF3 | ENSG00000234476 | RP11-496N12.6 |
| ILF3 | ENSG00000138600 | SPPL2A |
| ILF3 | ENSG00000089775 | ZBTB25 |
| ILF3 | ENSG00000107736 | CDH23 |
| ILF3 | ENSG00000236871 | LINC00106 |
| ILF3 | ENSG00000141560 | FN3KRP |
| ILF3 | ENSG00000163788 | SNRK |
| ILF3 | ENSG00000259583 | RP11-66B24.4 |
| ILF3 | ENSG00000182271 | TMIGD1 |
| ILF3 | ENSG00000136261 | BZW2 |

| ILF3 | ENSG00000224545 | AC008264.4 |
| --- | --- | --- |
| ILF3 | ENSG00000253301 | RP11-513O17.2 |
| ILF3 | ENSG00000243738 | RN7SL181P |
| ILF3 | ENSG00000168101 | NUDT16L1 |
| ILF3 | ENSG00000228412 | RP4-625H18.2 |
| ILF3 | ENSG00000222790 | RNU4-14P |
| ILF3 | ENSG00000269919 | RP1-134E15.3 |
| ILF3 | ENSG00000067955 | CBFB |
| ILF3 | ENSG00000080608 | KIAA0020 |
| ILF3 | ENSG00000153774 | CFDP1 |
| ILF3 | ENSG00000273329 | RP11-448A19.1 |
| ILF3 | ENSG00000125657 | TNFSF9 |
| ILF3 | ENSG00000104228 | TRIM35 |
| ILF3 | ENSG00000231074 | HCG18 |
| ILF3 | ENSG00000171132 | PRKCE |
| ILF3 | ENSG00000102098 | SCML2 |
| ILF3 | ENSG00000108342 | CSF3 |
| ILF3 | ENSG00000179846 | NKPD1 |
| ILF3 | ENSG00000144712 | CAND2 |
| ILF3 | ENSG00000204257 | HLA-DMA |
| ILF3 | ENSG00000255381 | AP001258.5 |
| ILF3 | ENSG00000125968 | ID1 |
| ILF3 | ENSG00000169692 | AGPAT2 |
| ILF3 | ENSG00000138434 | SSFA2 |
| ILF3 | ENSG00000063761 | ADCK1 |
| ILF3 | ENSG00000206785 | SNORA15 |
| ILF3 | ENSG00000104408 | EIF3E |
| ILF3 | ENSG00000259343 | RP11-761I4.3 |
| ILF3 | ENSG00000184634 | MED12 |
| ILF3 | ENSG00000124357 | NAGK |
| ILF3 | ENSG00000223350 | IGLV9-49 |
| ILF3 | ENSG00000185168 | LINC00482 |
| ILF3 | ENSG00000115234 | SNX17 |
| ILF3 | ENSG00000137460 | FHDC1 |
| ILF3 | ENSG00000172183 | ISG20 |
| ILF3 | ENSG00000271871 | AC005740.6 |
| ILF3 | ENSG00000236200 | KDM4A-AS1 |
| ILF3 | ENSG00000136052 | SLC41A2 |
| ILF3 | ENSG00000258839 | MC1R |
| ILF3 | ENSG00000067082 | KLF6 |
| ILF3 | ENSG00000226421 | SLC25A5P5 |
| ILF3 | ENSG00000196715 | VKORC1L1 |
| ILF3 | ENSG00000249335 | CTC-340D7.1 |
| ILF3 | ENSG00000121210 | KIAA0922 |
| ILF3 | ENSG00000112218 | GPR63 |
| ILF3 | ENSG00000230536 | RP11-379C10.4 |
| ILF3 | ENSG00000199687 | RNU1-38P |
| ILF3 | ENSG00000255959 | RP11-804A23.2 |
| ILF3 | ENSG00000082213 | C5orf22 |
| ILF3 | ENSG00000198393 | ZNF26 |
| ILF3 | ENSG00000278771 | Metazoa_SRP |

| ILF3 | ENSG00000096395 | MLN |
| --- | --- | --- |
| ILF3 | ENSG00000179151 | EDC3 |
| ILF3 | ENSG00000189120 | SP6 |
| ILF3 | ENSG00000164663 | USP49 |
| ILF3 | ENSG00000198382 | UVRAG |
| ILF3 | ENSG00000156239 | N6AMT1 |
| ILF3 | ENSG00000138442 | WDR12 |
| ILF3 | ENSG00000178531 | CTXN1 |
| ILF3 | ENSG00000258404 | RP11-1029J19.5 |
| ILF3 | ENSG00000166002 | SMCO4 |
| ILF3 | ENSG00000159708 | LRRC36 |
| ILF3 | ENSG00000164951 | PDP1 |
| ILF3 | ENSG00000105974 | CAV1 |
| ILF3 | ENSG00000277452 | Metazoa_SRP |
| ILF3 | ENSG00000225867 | RP5-1177I5.3 |
| ILF3 | ENSG00000277501 | RP11-697E22.2 |
| ILF3 | ENSG00000233198 | RNF224 |
| ILF3 | ENSG00000185163 | DDX51 |
| ILF3 | ENSG00000003402 | CFLAR |
| ILF3 | ENSG00000160606 | TLCD1 |
| ILF3 | ENSG00000170473 | WIBG |
| ILF3 | ENSG00000187688 | TRPV2 |
| ILF3 | ENSG00000246541 | RP11-363G15.2 |
| ILF3 | ENSG00000136487 | GH2 |
| ILF3 | ENSG00000146278 | PNRC1 |
| ILF3 | ENSG00000274248 | AJ011932.1 |
| ILF3 | ENSG00000263608 | RN7SL353P |
| ILF3 | ENSG00000196597 | ZNF782 |
| ILF3 | ENSG00000133740 | E2F5 |
| ILF3 | ENSG00000001631 | KRIT1 |
| ILF3 | ENSG00000130489 | SCO2 |
| ILF3 | ENSG00000266569 | RN7SL377P |
| ILF3 | ENSG00000231982 | RP11-573D15.1 |
| ILF3 | ENSG00000124006 | OBSL1 |
| ILF3 | ENSG00000270640 | RP11-373D23.2 |
| ILF3 | ENSG00000232750 | RP11-177A2.5 |
| ILF3 | ENSG00000128585 | MKLN1 |
| ILF3 | ENSG00000104067 | TJP1 |
| ILF3 | ENSG00000185664 | PMEL |
| ILF3 | ENSG00000013563 | DNASE1L1 |
| ILF3 | ENSG00000172661 | FAM21C |
| ILF3 | ENSG00000129194 | SOX15 |
| ILF3 | ENSG00000174501 | ANKRD36C |
| ILF3 | ENSG00000221983 | UBA52 |
| ILF3 | ENSG00000243455 | RPS18P13 |
| ILF3 | ENSG00000276853 | RP11-305O6.4 |
| ILF3 | ENSG00000109189 | USP46 |
| ILF3 | ENSG00000223549 | MTND5P28 |
| ILF3 | ENSG00000196236 | XPNPEP3 |
| ILF3 | ENSG00000107897 | ACBD5 |
| ILF3 | ENSG00000278433 | MIR6070 |

| ILF3 | ENSG00000185811 | IKZF1 |
| --- | --- | --- |
| ILF3 | ENSG00000164631 | ZNF12 |
| ILF3 | ENSG00000179044 | EXOC3L1 |
| ILF3 | ENSG00000175806 | MSRA |
| ILF3 | ENSG00000274314 | MIR6749 |
| ILF3 | ENSG00000223945 | RP11-458I7.1 |
| ILF3 | ENSG00000123575 | FAM199X |
| ILF3 | ENSG00000198742 | SMURF1 |
| ILF3 | ENSG00000104312 | RIPK2 |
| ILF3 | ENSG00000181856 | SLC2A4 |
| ILF3 | ENSG00000272486 | RP11-532M24.1 |
| ILF3 | ENSG00000173673 | HES3 |
| ILF3 | ENSG00000275646 | SMAD5-AS1_2 |
| ILF3 | ENSG00000247199 | RP11-373N22.3 |
| ILF3 | ENSG00000168538 | TRAPPC11 |
| ILF3 | ENSG00000196588 | MKL1 |
| ILF3 | ENSG00000229261 | RP11-227H15.4 |
| ILF3 | ENSG00000250562 | RPL38P4 |
| ILF3 | ENSG00000178821 | TMEM52 |
| ILF3 | ENSG00000197077 | KIAA1671 |
| ILF3 | ENSG00000229922 | RP11-240M16.1 |
| ILF3 | ENSG00000269480 | CTD-3032J10.3 |
| ILF3 | ENSG00000135720 | DYNC1LI2 |
| ILF3 | ENSG00000211460 | TSN |
| ILF3 | ENSG00000013441 | CLK1 |
| ILF3 | ENSG00000196584 | XRCC2 |
| ILF3 | ENSG00000223764 | RP11-54O7.3 |
| ILF3 | ENSG00000023445 | BIRC3 |
| ILF3 | ENSG00000206974 | RNU6-1144P |
| ILF3 | ENSG00000268230 | CTD-2619J13.8 |
| ILF3 | ENSG00000135828 | RNASEL |
| ILF3 | ENSG00000068305 | MEF2A |
| ILF3 | ENSG00000105650 | PDE4C |
| ILF3 | ENSG00000163346 | PBXIP1 |
| ILF3 | ENSG00000207501 | RNVU1-14 |
| ILF3 | ENSG00000103496 | STX4 |
| ILF3 | ENSG00000277718 | CTD- |
| ILF3 | ENSG00000165819 | 2311M21.5  METTL3 |
| ILF3 | ENSG00000185379 | RAD51D |
| ILF3 | ENSG00000268262 | CTC-246B18.8 |
| ILF3 | ENSG00000137871 | ZNF280D |
| ILF3 | ENSG00000231249 | ITPR1-AS1 |
| ILF3 | ENSG00000203883 | SOX18 |
| ILF3 | ENSG00000133226 | SRRM1 |
| ILF3 | ENSG00000117620 | SLC35A3 |
| ILF3 | ENSG00000166592 | RRAD |
| ILF3 | ENSG00000205955 | HSP90AA5P |
| ILF3 | ENSG00000231353 | RP11-5P18.3 |
| ILF3 | ENSG00000198885 | ITPRIPL1 |
| ILF3 | ENSG00000148459 | PDSS1 |
| ILF3 | ENSG00000166987 | MBD6 |

| ILF3 | ENSG00000010319 | SEMA3G |
| --- | --- | --- |
| ILF3 | ENSG00000168066 | SF1 |
| ILF3 | ENSG00000204351 | SKIV2L |
| ILF3 | ENSG00000087086 | FTL |
| ILF3 | ENSG00000060069 | CTDP1 |
| ILF3 | ENSG00000134884 | ARGLU1 |
| ILF3 | ENSG00000253737 | KB-1460A1.3 |
| ILF3 | ENSG00000255491 | RP11-1082L8.4 |
| ILF3 | ENSG00000116062 | MSH6 |
| ILF3 | ENSG00000127415 | IDUA |
| ILF3 | ENSG00000075826 | SEC31B |
| ILF3 | ENSG00000086712 | TXLNG |
| ILF3 | ENSG00000225828 | FAM229A |
| ILF3 | ENSG00000162650 | ATXN7L2 |
| ILF3 | ENSG00000135631 | RAB11FIP5 |
| ILF3 | ENSG00000154743 | TSEN2 |
| ILF3 | ENSG00000188779 | SKOR1 |
| ILF3 | ENSG00000152795 | HNRNPDL |
| ILF3 | ENSG00000260852 | FBXL19-AS1 |
| ILF3 | ENSG00000150672 | DLG2 |
| ILF3 | ENSG00000117013 | KCNQ4 |
| ILF3 | ENSG00000070785 | EIF2B3 |
| ILF3 | ENSG00000173349 | SFT2D3 |
| ILF3 | ENSG00000158301 | GPRASP2 |
| ILF3 | ENSG00000178177 | LCORL |
| ILF3 | ENSG00000117533 | VAMP4 |
| ILF3 | ENSG00000213005 | PTTG3P |
| ILF3 | ENSG00000092470 | WDR76 |
| ILF3 | ENSG00000129173 | E2F8 |
| ILF3 | ENSG00000267691 | SHC1P2 |
| ILF3 | ENSG00000148187 | MRRF |
| ILF3 | ENSG00000167972 | ABCA3 |
| ILF3 | ENSG00000207405 | SNORA64 |
| ILF3 | ENSG00000022567 | SLC45A4 |
| ILF3 | ENSG00000163993 | S100P |
| ILF3 | ENSG00000127337 | YEATS4 |
| ILF3 | ENSG00000175832 | ETV4 |
| ILF3 | ENSG00000165115 | KIF27 |
| ILF3 | ENSG00000254328 | CTC-308K20.4 |
| ILF3 | ENSG00000160336 | ZNF761 |
| ILF3 | ENSG00000254483 | RP11-23J9.6 |
| ILF3 | ENSG00000162819 | BROX |
| ILF3 | ENSG00000139540 | SLC39A5 |
| ILF3 | ENSG00000148218 | ALAD |
| ILF3 | ENSG00000130669 | PAK4 |
| ILF3 | ENSG00000134283 | PPHLN1 |
| ILF3 | ENSG00000126070 | AGO3 |
| ILF3 | ENSG00000163607 | GTPBP8 |
| ILF3 | ENSG00000242199 | RP11-71H17.1 |
| ILF3 | ENSG00000196712 | NF1 |
| ILF3 | ENSG00000108175 | ZMIZ1 |

| ILF3 | ENSG00000112592 | TBP |
| --- | --- | --- |
| ILF3 | ENSG00000124784 | RIOK1 |
| ILF3 | ENSG00000235453 | TOPORS-AS1 |
| ILF3 | ENSG00000249487 | RP11-97O12.2 |
| ILF3 | ENSG00000136279 | DBNL |
| ILF3 | ENSG00000054938 | CHRDL2 |
| ILF3 | ENSG00000157895 | C12orf43 |
| ILF3 | ENSG00000160326 | SLC2A6 |
| ILF3 | ENSG00000279108 | CTC-490E21.11 |
| ILF3 | ENSG00000184432 | COPB2 |
| ILF3 | ENSG00000099840 | IZUMO4 |
| ILF3 | ENSG00000065361 | ERBB3 |
| ILF3 | ENSG00000224725 | CEP57L1P1 |
| ILF3 | ENSG00000071889 | FAM3A |
| ILF3 | ENSG00000280153 | RP11-876N24.3 |
| ILF3 | ENSG00000187260 | WDR86 |
| ILF3 | ENSG00000167748 | KLK1 |
| ILF3 | ENSG00000151365 | THRSP |
| ILF3 | ENSG00000228317 | RP11-235C23.5 |
| ILF3 | ENSG00000132823 | OSER1 |
| ILF3 | ENSG00000130758 | MAP3K10 |
| ILF3 | ENSG00000070047 | PHRF1 |
| ILF3 | ENSG00000238387 | snoU13 |
| ILF3 | ENSG00000273980 | RP13-49I15.6 |
| ILF3 | ENSG00000099875 | MKNK2 |
| ILF3 | ENSG00000206903 | SNORA24 |
| ILF3 | ENSG00000163597 | SNHG16 |
| ILF3 | ENSG00000224165 | DNAJC27-AS1 |
| ILF3 | ENSG00000009335 | UBE3C |
| ILF3 | ENSG00000231416 | RP11-422P24.9 |
| ILF3 | ENSG00000111261 | MANSC1 |
| ILF3 | ENSG00000254508 | FBXO3-AS1 |
| ILF3 | ENSG00000196074 | SYCP2 |
| ILF3 | ENSG00000206652 | RNU1-1 |
| ILF3 | ENSG00000268030 | AC005253.2 |
| ILF3 | ENSG00000120832 | MTERF2 |
| ILF3 | ENSG00000158545 | ZC3H18 |
| ILF3 | ENSG00000198001 | IRAK4 |
| ILF3 | ENSG00000238754 | snoU109 |
| ILF3 | ENSG00000196422 | PPP1R26 |
| ILF3 | ENSG00000254675 | RP11-7I15.4 |
| ILF3 | ENSG00000279528 | RP11-1148L6.8 |
| ILF3 | ENSG00000140521 | POLG |
| ILF3 | ENSG00000227630 | LINC01132 |
| ILF3 | ENSG00000252361 | RNU6-118P |
| ILF3 | ENSG00000268047 | AC018766.6 |
| ILF3 | ENSG00000267278 | MAP3K14-AS1 |
| ILF3 | ENSG00000155287 | SLC25A28 |
| ILF3 | ENSG00000267731 | RP11-147L13.8 |
| ILF3 | ENSG00000222445 | RN7SKP56 |
| ILF3 | ENSG00000215692 | AC114730.8 |

| ILF3 | ENSG00000184224 | C11orf72 |
| --- | --- | --- |
| ILF3 | ENSG00000127124 | HIVEP3 |
| ILF3 | ENSG00000164077 | MON1A |
| ILF3 | ENSG00000106144 | CASP2 |
| ILF3 | ENSG00000112697 | TMEM30A |
| ILF3 | ENSG00000197180 | CH17- |
| ILF3 | ENSG00000121680 | 340M24.3  PEX16 |
| ILF3 | ENSG00000202186 | RNU6-497P |
| ILF3 | ENSG00000250317 | SMIM20 |
| ILF3 | ENSG00000243660 | ZNF487 |
| ILF3 | ENSG00000114812 | VIPR1 |
| ILF3 | ENSG00000201675 | SNORD32A |
| ILF3 | ENSG00000249047 | COX6B1P5 |
| ILF3 | ENSG00000196420 | S100A5 |
| ILF3 | ENSG00000232233 | RP11-573D15.2 |
| ILF3 | ENSG00000162836 | ACP6 |
| ILF3 | ENSG00000125868 | DSTN |
| ILF3 | ENSG00000011347 | SYT7 |
| ILF3 | ENSG00000128298 | BAIAP2L2 |
| ILF3 | ENSG00000275167 | MIR6815 |
| ILF3 | ENSG00000132612 | VPS4A |
| ILF3 | ENSG00000187815 | ZFP69 |
| ILF3 | ENSG00000198168 | SVIP |
| ILF3 | ENSG00000159322 | ADPGK |
| ILF3 | ENSG00000225183 | RP4-758J24.4 |
| ILF3 | ENSG00000260340 | RP11-254F19.3 |
| ILF3 | ENSG00000249086 | AC051649.12 |
| ILF3 | ENSG00000271888 | RP11-560J1.2 |
| ILF3 | ENSG00000155761 | SPAG17 |
| ILF3 | ENSG00000251409 | AC008592.4 |
| ILF3 | ENSG00000215251 | FASTKD5 |
| ILF3 | ENSG00000141404 | GNAL |
| ILF3 | ENSG00000275479 | RP11-334C17.6 |
| ILF3 | ENSG00000100271 | TTLL1 |
| ILF3 | ENSG00000224616 | RTCA-AS1 |
| ILF3 | ENSG00000224261 | RP11-179G5.1 |
| ILF3 | ENSG00000229660 | RP5-1142J19.1 |
| ILF3 | ENSG00000135119 | RNFT2 |
| ILF3 | ENSG00000139629 | GALNT6 |
| ILF3 | ENSG00000101347 | SAMHD1 |
| ILF3 | ENSG00000260894 | CTD- |
| ILF3 | ENSG00000166716 | 2012K14.4  ZNF592 |
| ILF3 | ENSG00000067225 | PKM |
| ILF3 | ENSG00000165424 | ZCCHC24 |
| ILF3 | ENSG00000054611 | TBC1D22A |
| ILF3 | ENSG00000108468 | CBX1 |
| ILF3 | ENSG00000267518 | RP11-794C22.2 |
| ILF3 | ENSG00000273335 | RP11-61L19.2 |
| ILF3 | ENSG00000170382 | LRRN2 |
| ILF3 | ENSG00000224514 | LINC00620 |

ILF3 ENSG00000178209 PLEC

| ILF3 | ENSG00000177984 | LCN15 |
| --- | --- | --- |
| ILF3 | ENSG00000176087 | SLC35A4 |
| ILF3 | ENSG00000054967 | RELT |
| ILF3 | ENSG00000188760 | TMEM198 |
| ILF3 | ENSG00000101882 | NKAP |
| ILF3 | ENSG00000166569 | CPLX4 |
| ILF3 | ENSG00000202089 | RNU6-1306P |
| ILF3 | ENSG00000059377 | TBXAS1 |
| ILF3 | ENSG00000157107 | FCHO2 |
| ILF3 | ENSG00000120889 | TNFRSF10B |
| ILF3 | ENSG00000137822 | TUBGCP4 |
| ILF3 | ENSG00000117505 | DR1 |
| ILF3 | ENSG00000106034 | CPED1 |
| ILF3 | ENSG00000124459 | ZNF45 |
| ILF3 | ENSG00000158987 | RAPGEF6 |
| ILF3 | ENSG00000264102 | MIR4688 |
| ILF3 | ENSG00000273973 | RP11-973D8.5 |
| ILF3 | ENSG00000201512 | SNORA71C |
| ILF3 | ENSG00000274180 | NATD1 |
| ILF3 | ENSG00000162490 | DRAXIN |
| ILF3 | ENSG00000184271 | POU6F1 |
| ILF3 | ENSG00000278249 | SCARNA2 |
| ILF3 | ENSG00000006459 | KDM7A |
| ILF3 | ENSG00000202415 | RN7SKP269 |
| ILF3 | ENSG00000115687 | PASK |
| ILF3 | ENSG00000272186 | RP11-110I1.13 |
| ILF3 | ENSG00000253180 | RP11-410L14.1 |
| ILF3 | ENSG00000211644 | IGLV1-51 |
| ILF3 | ENSG00000165118 | C9orf64 |
| ILF3 | ENSG00000148688 | RPP30 |
| ILF3 | ENSG00000205302 | SNX2 |
| ILF3 | ENSG00000229980 | TOB1-AS1 |
| ILF3 | ENSG00000142552 | RCN3 |
| ILF3 | ENSG00000196526 | AFAP1 |
| ILF3 | ENSG00000258439 | RP11-173A8.2 |
| ILF3 | ENSG00000168256 | NKIRAS2 |
| ILF3 | ENSG00000110076 | NRXN2 |
| ILF3 | ENSG00000151090 | THRB |
| ILF3 | ENSG00000159231 | CBR3 |
| ILF3 | ENSG00000164118 | CEP44 |
| ILF3 | ENSG00000220323 | HIST2H2BD |
| ILF3 | ENSG00000265444 | MIR4733 |
| ILF3 | ENSG00000105429 | MEGF8 |
| ILF3 | ENSG00000264386 | MIR4513 |
| ILF3 | ENSG00000124226 | RNF114 |
| ILF3 | ENSG00000177076 | ACER2 |
| ILF3 | ENSG00000178997 | EXD1 |
| ILF3 | ENSG00000154124 | OTULIN |
| ILF3 | ENSG00000273568 | RP11-417L19.6 |
| ILF3 | ENSG00000101935 | AMMECR1 |
| ILF3 | ENSG00000212541 | RNU6-510P |

| ILF3 | ENSG00000101132 | PFDN4 |
| --- | --- | --- |
| ILF3 | ENSG00000163683 | SMIM14 |
| ILF3 | ENSG00000167625 | ZNF526 |
| ILF3 | ENSG00000100201 | DDX17 |
| ILF3 | ENSG00000100614 | PPM1A |
| ILF3 | ENSG00000244462 | RBM12 |
| ILF3 | ENSG00000234719 | RP11-166B2.1 |
| ILF3 | ENSG00000278224 | PRICKLE4 |
| ILF3 | ENSG00000157869 | RAB28 |
| ILF3 | ENSG00000230274 | PGAM1P3 |
| ILF3 | ENSG00000100348 | TXN2 |
| ILF3 | ENSG00000069869 | NEDD4 |
| ILF3 | ENSG00000163050 | ADCK3 |
| ILF3 | ENSG00000222675 | RNA5SP146 |
| ILF3 | ENSG00000112378 | PERP |
| ILF3 | ENSG00000212466 | RNU6-952P |
| ILF3 | ENSG00000201641 | RNU6-1187P |
| ILF3 | ENSG00000046604 | DSG2 |
| ILF3 | ENSG00000095321 | CRAT |
| ILF3 | ENSG00000198612 | COPS8 |
| ILF3 | ENSG00000134138 | MEIS2 |
| ILF3 | ENSG00000270781 | RP11-501C14.9 |
| ILF3 | ENSG00000225046 | RP1-20I3.3 |
| ILF3 | ENSG00000153879 | CEBPG |
| ILF3 | ENSG00000264647 | RP11-68I3.7 |
| ILF3 | ENSG00000125877 | ITPA |
| ILF3 | ENSG00000276131 | RP11-481J2.3 |
| ILF3 | ENSG00000126787 | DLGAP5 |
| ILF3 | ENSG00000081026 | MAGI3 |
| ILF3 | ENSG00000274934 | SMAD5-AS1_4 |
| ILF3 | ENSG00000197857 | ZNF44 |
| ILF3 | ENSG00000121749 | TBC1D15 |
| ILF3 | ENSG00000111676 | ATN1 |
| ILF3 | ENSG00000066923 | STAG3 |
| ILF3 | ENSG00000270133 | CTC-303L1.2 |
| ILF3 | ENSG00000197299 | BLM |
| ILF3 | ENSG00000164124 | TMEM144 |
| ILF3 | ENSG00000214022 | REPIN1 |
| ILF3 | ENSG00000266521 | RP11-650P15.1 |
| ILF3 | ENSG00000278716 | LA16c-352F7.1 |
| ILF3 | ENSG00000132953 | XPO4 |
| ILF3 | ENSG00000279066 | RP13-20L14.9 |
| ILF3 | ENSG00000235236 | RP13-131K19.1 |
| ILF3 | ENSG00000125458 | NT5C |
| ILF3 | ENSG00000272682 | AC004471.10 |
| ILF3 | ENSG00000127870 | RNF6 |
| ILF3 | ENSG00000096093 | EFHC1 |
| ILF3 | ENSG00000201492 | RNA5SP78 |
| ILF3 | ENSG00000144802 | NFKBIZ |
| ILF3 | ENSG00000225950 | NTF4 |
| ILF3 | ENSG00000227165 | WDR11-AS1 |

| ILF3 | ENSG00000054148 | PHPT1 |
| --- | --- | --- |
| ILF3 | ENSG00000187801 | ZFP69B |
| ILF3 | ENSG00000224397 | LINC01272 |
| ILF3 | ENSG00000198000 | NOL8 |
| ILF3 | ENSG00000274963 | Metazoa_SRP |
| ILF3 | ENSG00000007384 | RHBDF1 |
| ILF3 | ENSG00000213024 | NUP62 |
| ILF3 | ENSG00000164308 | ERAP2 |
| ILF3 | ENSG00000268991 | FAM231C |
| ILF3 | ENSG00000137449 | CPEB2 |
| ILF3 | ENSG00000177663 | IL17RA |
| ILF3 | ENSG00000164061 | BSN |
| ILF3 | ENSG00000258820 | RP11-293M10.2 |
| ILF3 | ENSG00000279087 | RP11-83B20.10 |
| ILF3 | ENSG00000254533 | AF186192.1 |
| ILF3 | ENSG00000274460 | CTD-2649C14.2 |
| ILF3 | ENSG00000251396 | LINC01301 |
| ILF3 | ENSG00000267079 | RP11-820I16.1 |
| ILF3 | ENSG00000207037 | RNU6-339P |
| ILF3 | ENSG00000273674 | CTD-2378E12.1 |
| ILF3 | ENSG00000101868 | POLA1 |
| ILF3 | ENSG00000129197 | RPAIN |
| ILF3 | ENSG00000075336 | TIMM21 |
| ILF3 | ENSG00000174498 | IGDCC3 |
| ILF3 | ENSG00000273179 | RP11-20I20.4 |
| ILF3 | ENSG00000145063 | FLJ33534 |
| ILF3 | ENSG00000225499 | RPL15P4 |
| ILF3 | ENSG00000232519 | RP11-29H23.4 |
| ILF3 | ENSG00000232850 | PTGES2-AS1 |
| ILF3 | ENSG00000080839 | RBL1 |
| ILF3 | ENSG00000119777 | TMEM214 |
| ILF3 | ENSG00000240583 | AQP1 |
| ILF3 | ENSG00000278914 | KB-1517D11.3 |
| ILF3 | ENSG00000158792 | SPATA2L |
| ILF3 | ENSG00000063978 | RNF4 |
| ILF3 | ENSG00000186272 | ZNF17 |
| ILF3 | ENSG00000129355 | CDKN2D |
| ILF3 | ENSG00000168228 | ZCCHC4 |
| ILF3 | ENSG00000158161 | EYA3 |
| ILF3 | ENSG00000231028 | LINC00271 |
| ILF3 | ENSG00000279827 | RP11-886P16.10 |
| ILF3 | ENSG00000143353 | LYPLAL1 |
| ILF3 | ENSG00000164164 | OTUD4 |
| ILF3 | ENSG00000213386 | RP11-779O18.2 |
| ILF3 | ENSG00000274356 | RP11-216L13.21 |
| ILF3 | ENSG00000138175 | ARL3 |
| ILF3 | ENSG00000196693 | ZNF33B |
| ILF3 | ENSG00000129675 | ARHGEF6 |
| ILF3 | ENSG00000177558 | FAM187B |
| ILF3 | ENSG00000258881 | AC007040.11 |
| ILF3 | ENSG00000245552 | RP11-712B9.2 |

| ILF3 | ENSG00000264107 | RP11-848P1.5 |
| --- | --- | --- |
| ILF3 | ENSG00000153207 | AHCTF1 |
| ILF3 | ENSG00000106484 | MEST |
| ILF3 | ENSG00000157404 | KIT |
| ILF3 | ENSG00000134594 | RAB33A |
| ILF3 | ENSG00000168298 | HIST1H1E |
| ILF3 | ENSG00000275092 | LA16c-329F2.2 |
| ILF3 | ENSG00000136628 | EPRS |
| ILF3 | ENSG00000249679 | RP11-279O9.4 |
| ILF3 | ENSG00000086758 | HUWE1 |
| ILF3 | ENSG00000150636 | CCDC102B |
| ILF3 | ENSG00000265291 | MIR4710 |
| ILF3 | ENSG00000162148 | PPP1R32 |
| ILF3 | ENSG00000010671 | BTK |
| ILF3 | ENSG00000124613 | ZNF391 |
| ILF3 | ENSG00000181908 | AP003774.4 |
| ILF3 | ENSG00000112996 | MRPS30 |
| ILF3 | ENSG00000122068 | FYTTD1 |
| ILF3 | ENSG00000266826 | CTD-2200P10.1 |
| ILF3 | ENSG00000256681 | CCDC58P5 |
| ILF3 | ENSG00000232909 | RP3-510O8.4 |
| ILF3 | ENSG00000130766 | SESN2 |
| ILF3 | ENSG00000134291 | TMEM106C |
| ILF3 | ENSG00000228863 | RP11-404F10.2 |
| ILF3 | ENSG00000088899 | LZTS3; |
| ILF3 | ENSG00000271895 | RP4-635E18.8 |
| ILF3 | ENSG00000267064 | UXT-AS1 |
| ILF3 | ENSG00000058799 | YIPF1 |
| ILF3 | ENSG00000214702 | RP11-247C2.1 |
| ILF3 | ENSG00000262410 | RP11-388C12.8 |
| ILF3 | ENSG00000187492 | CDHR4 |
| ILF3 | ENSG00000267069 | RP11-64C12.8 |
| ILF3 | ENSG00000165934 | CPSF2 |
| ILF3 | ENSG00000274828 | RP11-567M16.6 |
| ILF3 | ENSG00000248919 | ATP5J2-PTCD1 |
| ILF3 | ENSG00000233077 | LINC01271 |
| ILF3 | ENSG00000092850 | TEKT2 |
| ILF3 | ENSG00000258446 | RP11-895M11.2 |
| ILF3 | ENSG00000182768 | NGRN |
| ILF3 | ENSG00000263672 | RN7SL750P |
| ILF3 | ENSG00000111879 | FAM184A |
| ILF3 | ENSG00000162129 | CLPB |
| ILF3 | ENSG00000162377 | COA7 |
| ILF3 | ENSG00000163378 | EOGT |
| ILF3 | ENSG00000149932 | TMEM219 |
| ILF3 | ENSG00000121988 | ZRANB3 |
| ILF3 | ENSG00000272690 | RP11-803B1.8 |
| ILF3 | ENSG00000161011 | SQSTM1 |
| ILF3 | ENSG00000267624 | RP11-219G17.6 |
| ILF3 | ENSG00000269749 | AC005614.5 |
| ILF3 | ENSG00000114650 | SCAP |

| ILF3 | ENSG00000141026 | MED9 |
| --- | --- | --- |
| ILF3 | ENSG00000206680 | SNORD21 |
| ILF3 | ENSG00000262967 | RP11-294J22.6 |
| ILF3 | ENSG00000136758 | YME1L1 |
| ILF3 | ENSG00000225663 | FAM195B |
| ILF3 | ENSG00000150593 | PDCD4 |
| ILF3 | ENSG00000143641 | GALNT2 |
| ILF3 | ENSG00000181240 | SLC25A41 |
| ILF3 | ENSG00000183979 | NPB |
| ILF3 | ENSG00000278775 | pRNA |
| ILF3 | ENSG00000259984 | RP11-335G20.7 |
| ILF3 | ENSG00000170075 | GPR37L1 |
| ILF3 | ENSG00000233222 | RP11-216N14.9 |
| ILF3 | ENSG00000227615 | RP11-864N7.2 |
| ILF3 | ENSG00000110435 | PDHX |
| ILF3 | ENSG00000116741 | RGS2 |
| ILF3 | ENSG00000006071 | ABCC8 |
| ILF3 | ENSG00000267625 | RP11-1094M14.14 |
| ILF3 | ENSG00000174669 | SLC29A2 |
| ILF3 | ENSG00000136238 | RAC1 |
| ILF3 | ENSG00000172273 | HINFP |
| ILF3 | ENSG00000237292 | RP11-540K16.1 |
| ILF3 | ENSG00000213085 | CFAP45 |
| ILF3 | ENSG00000161381 | PLXDC1 |
| ILF3 | ENSG00000038210 | PI4K2B |
| ILF3 | ENSG00000243762 | AC006547.8 |
| ILF3 | ENSG00000197457 | STMN3 |
| ILF3 | ENSG00000197226 | TBC1D9B |
| ILF3 | ENSG00000265678 | RP11-1376P16.2 |
| ILF3 | ENSG00000256913 | RP1-102E24.6 |
| ILF3 | ENSG00000218347 | HNRNPA1P1 |
| ILF3 | ENSG00000167978 | SRRM2 |
| ILF3 | ENSG00000076382 | SPAG5 |
| ILF3 | ENSG00000080572 | PIH1D3 |
| ILF3 | ENSG00000182612 | TSPAN10 |
| ILF3 | ENSG00000198795 | ZNF521 |
| ILF3 | ENSG00000197558 | SSPO |
| ILF3 | ENSG00000095203 | EPB41L4B |
| ILF3 | ENSG00000131142 | CCL25 |
| ILF3 | ENSG00000100027 | YPEL1 |
| ILF3 | ENSG00000261459 | ZNF747 |
| ILF3 | ENSG00000239636 | RP4-728D4.2 |
| ILF3 | ENSG00000137218 | FRS3 |
| ILF3 | ENSG00000183605 | SFXN4 |
| ILF3 | ENSG00000070718 | AP3M2 |
| ILF3 | ENSG00000260992 | DOCK9-AS2 |
| ILF3 | ENSG00000132196 | HSD17B7 |
| ILF3 | ENSG00000269947 | RP11-849F2.9 |
| ILF3 | ENSG00000256013 | RP11-27M24.1 |
| ILF3 | ENSG00000267448 | AC010649.1 |
| ILF3 | ENSG00000278017 | RP11-120K19.4 |

| ILF3 | ENSG00000187010 | RHD |
| --- | --- | --- |
| ILF3 | ENSG00000255306 | RP5-901A4.1 |
| ILF3 | ENSG00000021355 | SERPINB1 |
| ILF3 | ENSG00000103222 | ABCC1 |
| ILF3 | ENSG00000269889 | RP11-816J6.3 |
| ILF3 | ENSG00000169871 | TRIM56 |
| ILF3 | ENSG00000238723 | Y_RNA |
| ILF3 | ENSG00000197375 | SLC22A5 |
| ILF3 | ENSG00000267096 | CTD-2537I9.13 |
| ILF3 | ENSG00000231704 | AC004895.4 |
| ILF3 | ENSG00000239462 | CTD-2021J15.1 |
| ILF3 | ENSG00000200623 | SNORD18A |
| ILF3 | ENSG00000278324 | PVT1_3 |
| ILF3 | ENSG00000178761 | FAM219B |
| ILF3 | ENSG00000171873 | ADRA1D |
| ILF3 | ENSG00000213600 | XXcos- LUCA16.1 |
| ILF3 | ENSG00000104883 | PEX11G |
| ILF3 | ENSG00000258982 | RP11-638I2.4 |
| ILF3 | ENSG00000152465 | NMT2 |
| ILF3 | ENSG00000241769 | LINC00893 |
| ILF3 | ENSG00000186792 | HYAL3 |
| ILF3 | ENSG00000135387 | CAPRIN1 |
| ILF3 | ENSG00000189144 | ZNF573 |
| ILF3 | ENSG00000206935 | RNU6-514P |
| ILF3 | ENSG00000252840 | SNORA44 |
| ILF3 | ENSG00000199646 | RNU6-1272P |
| ILF3 | ENSG00000259523 | RP11-680F8.3 |
| ILF3 | ENSG00000270354 | RP11-547M24.1 |
| ILF3 | ENSG00000167393 | PPP2R3B |
| ILF3 | ENSG00000264772 | SNORA67 |
| ILF3 | ENSG00000028277 | POU2F2 |
| ILF3 | ENSG00000176531 | PHLDB3 |
| ILF3 | ENSG00000232628 | RP11-365O16.3 |
| ILF3 | ENSG00000130176 | CNN1 |
| ILF3 | ENSG00000217258 | AC007249.3 |
| ILF3 | ENSG00000140941 | MAP1LC3B |
| ILF3 | ENSG00000211513 | MIR320E |
| ILF3 | ENSG00000248399 | RP11-503N18.4 |
| ILF3 | ENSG00000164855 | TMEM184A |
| ILF3 | ENSG00000115677 | HDLBP |
| ILF3 | ENSG00000228192 | RP11-342M1.3 |
| ILF3 | ENSG00000120992 | LYPLA1 |
| ILF3 | ENSG00000104321 | TRPA1 |
| ILF3 | ENSG00000138764 | CCNG2 |
| ILF3 | ENSG00000123130 | ACOT9 |
| ILF3 | ENSG00000196498 | NCOR2 |
| ILF3 | ENSG00000166225 | FRS2 |
| ILF3 | ENSG00000161180 | CCDC116 |
| ILF3 | ENSG00000137806 | NDUFAF1 |
| ILF3 | ENSG00000149201 | CCDC81 |
| ILF3 | ENSG00000276384 | RP11-186B7.7 |

| ILF3 | ENSG00000243649 | CFB |
| --- | --- | --- |
| ILF3 | ENSG00000254975 | RP11-672A2.3 |
| ILF3 | ENSG00000126903 | SLC10A3 |
| ILF3 | ENSG00000163157 | TMOD4 |
| ILF3 | ENSG00000111863 | ADTRP |
| ILF3 | ENSG00000173273 | TNKS |
| ILF3 | ENSG00000230449 | RPL7P4 |
| ILF3 | ENSG00000173264 | GPR137 |
| ILF3 | ENSG00000130255 | RPL36 |
| ILF3 | ENSG00000182890 | GLUD2 |
| ILF3 | ENSG00000260806 | RP11-872J21.3 |
| ILF3 | ENSG00000180008 | SOCS4 |
| ILF3 | ENSG00000014123 | UFL1 |
| ILF3 | ENSG00000101306 | MYLK2 |
| ILF3 | ENSG00000221464 | MIR1271 |
| ILF3 | ENSG00000215900 | SEPW1P |
| ILF3 | ENSG00000173889 | PHC3 |
| ILF3 | ENSG00000275126 | HIST1H4L |
| ILF3 | ENSG00000142892 | PIGK |
| ILF3 | ENSG00000065000 | AP3D1 |
| ILF3 | ENSG00000244945 | RP11-1379J22.2 |
| ILF3 | ENSG00000027869 | SH2D2A |
| ILF3 | ENSG00000175931 | UBE2O |
| ILF3 | ENSG00000135018 | UBQLN1 |
| ILF3 | ENSG00000072609 | CHFR |
| ILF3 | ENSG00000225037 | EIF1AX-AS1 |
| ILF3 | ENSG00000226823 | SUGT1P |
| ILF3 | ENSG00000142623 | PADI1 |
| ILF3 | ENSG00000160087 | UBE2J2 |
| ILF3 | ENSG00000263154 | RP11-1055B8.2 |
| ILF3 | ENSG00000273293 | RP11-445N20.3 |
| ILF3 | ENSG00000278588 | HIST1H2BI |
| ILF3 | ENSG00000174080 | CTSF |
| ILF3 | ENSG00000198937 | CCDC167 |
| ILF3 | ENSG00000268575 | RP1-283E3.8 |
| ILF3 | ENSG00000273331 | TM4SF19- |
| ILF3 | ENSG00000177757 | TCTEX1D2  FAM87B |
| ILF3 | ENSG00000162910 | MRPL55 |
| ILF3 | ENSG00000006007 | GDE1 |
| ILF3 | ENSG00000256712 | RP11-785H5.2 |
| ILF3 | ENSG00000136425 | CIB2 |
| ILF3 | ENSG00000074660 | SCARF1 |
| ILF3 | ENSG00000077235 | GTF3C1 |
| ILF3 | ENSG00000239697 | TNFSF12 |
| ILF3 | ENSG00000103544 | C16orf62 |
| ILF3 | ENSG00000233836 | RP11-255H23.2 |
| ILF3 | ENSG00000104129 | DNAJC17 |
| ILF3 | ENSG00000164307 | ERAP1 |
| ILF3 | ENSG00000268292 | AC006547.15 |
| ILF3 | ENSG00000173221 | GLRX |
| ILF3 | ENSG00000123146 | CD97 |

| ILF3 | ENSG00000206113 | RP11-503N18.1 |
| --- | --- | --- |
| ILF3 | ENSG00000100519 | PSMC6 |
| ILF3 | ENSG00000005893 | LAMP2 |
| ILF3 | ENSG00000108773 | KAT2A |
| ILF3 | ENSG00000114473 | IQCG |
| ILF3 | ENSG00000144426 | NBEAL1 |
| ILF3 | ENSG00000272657 | AP000320.7 |
| ILF3 | ENSG00000121753 | BAI2 |
| ILF3 | ENSG00000264937 | AC100830.5 |
| ILF3 | ENSG00000167085 | PHB |
| ILF3 | ENSG00000197563 | PIGN |
| ILF3 | ENSG00000251393 | RP1-240K6.3 |
| ILF3 | ENSG00000243027 | RN7SL354P |
| ILF3 | ENSG00000114796 | KLHL24 |
| ILF3 | ENSG00000248746 | ACTN3 |
| ILF3 | ENSG00000134574 | DDB2 |
| ILF3 | ENSG00000261447 | RP11-109D9.4 |
| ILF3 | ENSG00000081853 | PCDHGA2 |
| ILF3 | ENSG00000230023 | RP11-10N16.2 |
| ILF3 | ENSG00000037897 | METTL1 |
| ILF3 | ENSG00000115109 | EPB41L5 |
| ILF3 | ENSG00000257193 | RP11-818F20.4 |
| ILF3 | ENSG00000245060 | LINC00847 |
| ILF3 | ENSG00000134905 | CARS2 |
| ILF3 | ENSG00000274618 | HIST1H4F |
| ILF3 | ENSG00000093010 | COMT |
| ILF3 | ENSG00000180881 | CAPS2 |
| ILF3 | ENSG00000269951 | RP11-797A18.6 |
| ILF3 | ENSG00000252826 | RNU1-92P |
| ILF3 | ENSG00000197355 | UAP1L1 |
| ILF3 | ENSG00000111077 | TENC1 |
| ILF3 | ENSG00000280132 | RP11-452L6.6 |
| ILF3 | ENSG00000175279 | APITD1 |
| ILF3 | ENSG00000207132 | Y_RNA |
| ILF3 | ENSG00000161653 | NAGS |
| ILF3 | ENSG00000221763 | MIR1289-1 |
| ILF3 | ENSG00000119636 | CCDC176 |
| ILF3 | ENSG00000156097 | GPR61 |
| ILF3 | ENSG00000256458 | RP11-363J17.1 |
| ILF3 | ENSG00000137857 | DUOX1 |
| ILF3 | ENSG00000124701 | APOBEC2 |
| ILF3 | ENSG00000237922 | RP11-478H16.1 |
| ILF3 | ENSG00000162873 | KLHDC8A |
| ILF3 | ENSG00000172732 | MUS81 |
| ILF3 | ENSG00000199875 | Y_RNA |
| ILF3 | ENSG00000214708 | AC090616.2 |
| ILF3 | ENSG00000072195 | SPEG |
| ILF3 | ENSG00000275834 | RP11-440E5.1 |
| ILF3 | ENSG00000213638 | ADAT3 |
| ILF3 | ENSG00000277967 | ZNFX1-AS1_3 |
| ILF3 | ENSG00000176732 | PFN4 |

| ILF3 | ENSG00000269091 | CTD-2126E3.3 |
| --- | --- | --- |
| ILF3 | ENSG00000228930 | MTCO1P3 |
| ILF3 | ENSG00000157540 | DYRK1A |
| ILF3 | ENSG00000181007 | ZFP82 |
| ILF3 | ENSG00000207467 | Y_RNA |
| ILF3 | ENSG00000213722 | DDAH2 |
| ILF3 | ENSG00000199756 | Y_RNA |
| ILF3 | ENSG00000239199 | RPL21P6 |
| ILF3 | ENSG00000100483 | VCPKMT |
| ILF3 | ENSG00000167747 | C19orf48 |
| ILF3 | ENSG00000230424 | RP1-43E13.2 |
| ILF3 | ENSG00000162882 | HAAO |
| ILF3 | ENSG00000253722 | RP11-10N23.4 |
| ILF3 | ENSG00000268564 | AC003956.1 |
| ILF3 | ENSG00000072415 | MPP5 |
| ILF3 | ENSG00000132471 | WBP2 |
| ILF3 | ENSG00000214530 | STARD10 |
| ILF3 | ENSG00000129467 | ADCY4 |
| ILF3 | ENSG00000120451 | SNX19 |
| ILF3 | ENSG00000101199 | ARFGAP1 |
| ILF3 | ENSG00000065978 | YBX1 |
| ILF3 | ENSG00000127588 | GNG13 |
| ILF3 | ENSG00000155792 | DEPTOR |
| ILF3 | ENSG00000140285 | FGF7 |
| ILF3 | ENSG00000161040 | FBXL13 |
| ILF3 | ENSG00000163535 | SGOL2 |
| ILF3 | ENSG00000256747 | RP11-860B13.1 |
| ILF3 | ENSG00000231827 | RP11-216N14.5 |
| ILF3 | ENSG00000147862 | NFIB |
| ILF3 | ENSG00000157570 | TSPAN18 |
| ILF3 | ENSG00000161956 | SENP3 |
| ILF3 | ENSG00000171489 | SPACA5 |
| ILF3 | ENSG00000106392 | C1GALT1 |
| ILF3 | ENSG00000011028 | MRC2 |
| ILF3 | ENSG00000126391 | FRMD8 |
| ILF3 | ENSG00000134825 | TMEM258 |
| ILF3 | ENSG00000256164 | CCND2-AS1 |
| ILF3 | ENSG00000257256 | RP3-405J10.4 |
| ILF3 | ENSG00000229032 | RP11-91A18.1 |
| ILF3 | ENSG00000179342 | GS1-124K5.9 |
| ILF3 | ENSG00000073331 | ALPK1 |
| ILF3 | ENSG00000119906 | FAM178A |
| ILF3 | ENSG00000134248 | LAMTOR5 |
| ILF3 | ENSG00000176378 | PFN1P10 |
| ILF3 | ENSG00000131966 | ACTR10 |
| ILF3 | ENSG00000251922 | SNORA14 |
| ILF3 | ENSG00000110011 | DNAJC4 |
| ILF3 | ENSG00000165233 | C9orf89 |
| ILF3 | ENSG00000278966 | FKSG48 |
| ILF3 | ENSG00000149781 | FERMT3 |
| ILF3 | ENSG00000109332 | UBE2D3 |

| ILF3 | ENSG00000224356 | RP11-151A6.4 |
| --- | --- | --- |
| ILF3 | ENSG00000115073 | ACTR1B |
| ILF3 | ENSG00000273004 | GS1-279B7.2 |
| ILF3 | ENSG00000166428 | PLD4 |
| ILF3 | ENSG00000240024 | LINC00888 |
| ILF3 | ENSG00000266392 | MIR4740 |
| ILF3 | ENSG00000068654 | POLR1A |
| ILF3 | ENSG00000076043 | REXO2 |
| ILF3 | ENSG00000279959 | CTD-2621I17.8 |
| ILF3 | ENSG00000179051 | RCC2 |
| ILF3 | ENSG00000234927 | HMGN1P18 |
| ILF3 | ENSG00000212420 | RNU6-1111P |
| ILF3 | ENSG00000251611 | RP11-610P16.1 |
| ILF3 | ENSG00000064601 | CTSA |
| ILF3 | ENSG00000120539 | MASTL |
| ILF3 | ENSG00000013573 | DDX11 |
| ILF3 | ENSG00000244080 | RN7SL833P |
| ILF3 | ENSG00000264675 | MIR4285 |
| ILF3 | ENSG00000231806 | PCAT7 |
| ILF3 | ENSG00000277669 | AC009133.22 |
| ILF3 | ENSG00000143878 | RHOB |
| ILF3 | ENSG00000112531 | QKI |
| ILF3 | ENSG00000214353 | VAC14-AS1 |
| ILF3 | ENSG00000151287 | TEX30 |
| ILF3 | ENSG00000121152 | NCAPH |
| ILF3 | ENSG00000185347 | C14orf80 |
| ILF3 | ENSG00000166963 | MAP1A |
| ILF3 | ENSG00000100417 | PMM1 |
| ILF3 | ENSG00000278344 | RP11-18C24.8 |
| ILF3 | ENSG00000277672 | RP11-386G11.11 |
| ILF3 | ENSG00000182993 | C12orf60 |
| ILF3 | ENSG00000227205 | PFN1P9 |
| ILF3 | ENSG00000255835 | RP4-559A3.7 |
| ILF3 | ENSG00000267735 | CTD-2265O21.7 |
| ILF3 | ENSG00000140459 | CYP11A1 |
| ILF3 | ENSG00000178700 | DHFRL1 |
| ILF3 | ENSG00000184716 | SERINC4 |
| ILF3 | ENSG00000130856 | ZNF236 |
| ILF3 | ENSG00000243711 | RPL21P116 |
| ILF3 | ENSG00000248385 | TARM1 |
| ILF3 | ENSG00000119042 | SATB2 |
| ILF3 | ENSG00000004142 | POLDIP2 |
| ILF3 | ENSG00000139722 | VPS37B |
| ILF3 | ENSG00000279347 | RP11-85I17.2 |
| ILF3 | ENSG00000136710 | CCDC115 |
| ILF3 | ENSG00000197429 | IPP |
| ILF3 | ENSG00000230701 | FBXW4P1 |
| ILF3 | ENSG00000271840 | RP1-224A6.9 |
| ILF3 | ENSG00000197956 | S100A6 |
| ILF3 | ENSG00000229970 | AC007128.1 |
| ILF3 | ENSG00000265393 | CTD- 2517M22.17 |

| ILF3 | ENSG00000124802 | EEF1E1 |
| --- | --- | --- |
| ILF3 | ENSG00000149929 | HIRIP3 |
| ILF3 | ENSG00000276918 | Metazoa_SRP |
| ILF3 | ENSG00000187091 | PLCD1 |
| ILF3 | ENSG00000204923 | FBXO48 |
| ILF3 | ENSG00000107281 | NPDC1 |
| ILF3 | ENSG00000162599 | NFIA |
| ILF3 | ENSG00000169583 | CLIC3 |
| ILF3 | ENSG00000161249 | DMKN |
| ILF3 | ENSG00000257800 | FNBP1P1 |
| ILF3 | ENSG00000132906 | CASP9 |
| ILF3 | ENSG00000180846 | CSNK1G2-AS1 |
| ILF3 | ENSG00000266589 | MIR4512 |
| ILF3 | ENSG00000175390 | EIF3F |
| ILF3 | ENSG00000106006 | HOXA6 |
| ILF3 | ENSG00000196268 | ZNF493 |
| ILF3 | ENSG00000051620 | HEBP2 |
| ILF3 | ENSG00000264549 | SNORD95 |
| ILF3 | ENSG00000267905 | CTD-2616J11.16 |
| ILF3 | ENSG00000201761 | RNU6-336P |
| ILF3 | ENSG00000249906 | RP5-1029K10.2 |
| ILF3 | ENSG00000235424 | SUMO2P10 |
| ILF3 | ENSG00000158480 | SPATA2 |
| ILF3 | ENSG00000164163 | ABCE1 |
| ILF3 | ENSG00000134253 | TRIM45 |
| ILF3 | ENSG00000266338 | NBPF15 |
| ILF3 | ENSG00000234380 | LINC01426 |
| ILF3 | ENSG00000253174 | RP11-360L9.7 |
| ILF3 | ENSG00000253395 | KB-1460A1.1 |
| ILF3 | ENSG00000226819 | MEIS1-AS3 |
| ILF3 | ENSG00000225724 | LL22NC03- |
| ILF3 | ENSG00000014641 | 80A10.11  MDH1 |
| ILF3 | ENSG00000268798 | CTB-25B13.5 |
| ILF3 | ENSG00000207523 | SNORA66 |
| ILF3 | ENSG00000099804 | CDC34 |
| ILF3 | ENSG00000172346 | CSDC2 |
| ILF3 | ENSG00000277984 | RP11-735A19.3 |
| ILF3 | ENSG00000259277 | RP13-126C7.1 |
| ILF3 | ENSG00000267646 | CTC-499B15.7 |
| ILF3 | ENSG00000248079 | DPH6-AS1 |
| ILF3 | ENSG00000188130 | MAPK12 |
| ILF3 | ENSG00000168724 | DNAJC21 |
| ILF3 | ENSG00000127220 | ABHD8 |
| ILF3 | ENSG00000238057 | ZEB2-AS1 |
| ILF3 | ENSG00000224470 | ATXN1L |
| ILF3 | ENSG00000239615 | CTD-2410N18.1 |
| ILF3 | ENSG00000104833 | TUBB4A |
| ILF3 | ENSG00000238082 | AC009948.7 |
| ILF3 | ENSG00000254942 | RP5-1160K1.8 |
| ILF3 | ENSG00000253344 | RP11-346L1.2 |
| ILF3 | ENSG00000135148 | TRAFD1 |

| ILF3 | ENSG00000243687 | RP11-432B6.1 |
| --- | --- | --- |
| ILF3 | ENSG00000260500 | CTD-3193O13.1 |
| ILF3 | ENSG00000261664 | TTC39A-AS1 |
| ILF3 | ENSG00000213889 | PPM1N |
| ILF3 | ENSG00000225953 | SATB2-AS1 |
| ILF3 | ENSG00000165714 | LOH12CR1 |
| ILF3 | ENSG00000114742 | WDR48 |
| ILF3 | ENSG00000250496 | ABT1P1 |
| ILF3 | ENSG00000114904 | NEK4 |
| ILF3 | ENSG00000269609 | RPARP-AS1 |
| ILF3 | ENSG00000113360 | DROSHA |
| ILF3 | ENSG00000101445 | PPP1R16B |
| ILF3 | ENSG00000103742 | IGDCC4 |
| ILF3 | ENSG00000068745 | IP6K2 |
| ILF3 | ENSG00000275265 | RP11-15J22.8 |
| ILF3 | ENSG00000114933 | INO80D |
| ILF3 | ENSG00000106554 | CHCHD3 |
| ILF3 | ENSG00000171262 | FAM98B |
| ILF3 | ENSG00000272148 | RP11-195B17.1 |
| ILF3 | ENSG00000143543 | JTB |
| ILF3 | ENSG00000123810 | B9D2 |
| ILF3 | ENSG00000130511 | SSBP4 |
| ILF3 | ENSG00000227764 | RP11-354K1.1 |
| ILF3 | ENSG00000178882 | FAM101A |
| ILF3 | ENSG00000260892 | CTD-2026K11.1 |
| ILF3 | ENSG00000224892 | RPS4XP16 |
| ILF3 | ENSG00000172932 | ANKRD13D |
| ILF3 | ENSG00000166454 | ATMIN |
| ILF3 | ENSG00000062725 | APPBP2 |
| ILF3 | ENSG00000182896 | TMEM95 |
| ILF3 | ENSG00000100567 | PSMA3 |
| ILF3 | ENSG00000116337 | AMPD2 |
| ILF3 | ENSG00000161048 | NAPEPLD |
| ILF3 | ENSG00000137478 | FCHSD2 |
| ILF3 | ENSG00000153147 | SMARCA5 |
| ILF3 | ENSG00000126264 | HCST |
| ILF3 | ENSG00000115216 | NRBP1 |
| ILF3 | ENSG00000198944 | SOWAHA |
| ILF3 | ENSG00000144043 | TEX261 |
| ILF3 | ENSG00000215256 | DHRS4-AS1 |
| ILF3 | ENSG00000164105 | SAP30 |
| ILF3 | ENSG00000068781 | STON1- |
| ILF3 | ENSG00000154134 | GTF2A1L ROBO3 |
| ILF3 | ENSG00000273066 | RP11-216L13.19 |
| ILF3 | ENSG00000242193 | RP11-568K15.1 |
| ILF3 | ENSG00000131188 | PRR7 |
| ILF3 | ENSG00000235333 | PVRIG2P |
| ILF3 | ENSG00000148835 | TAF5 |
| ILF3 | ENSG00000138629 | UBL7 |
| ILF3 | ENSG00000241582 | RP11-434O22.2 |
| ILF3 | ENSG00000261215 | RP11-195F19.30 |

| ILF3 | ENSG00000229694 | RP11-305L7.6 |
| --- | --- | --- |
| ILF3 | ENSG00000200179 | Y_RNA |
| ILF3 | ENSG00000134780 | DAGLA |
| ILF3 | ENSG00000150540 | HNMT |
| ILF3 | ENSG00000099797 | TECR |
| ILF3 | ENSG00000273297 | RP11-38M8.1 |
| ILF3 | ENSG00000124201 | ZNFX1 |
| ILF3 | ENSG00000166912 | MTMR10 |
| ILF3 | ENSG00000222020 | AC062017.1 |
| ILF3 | ENSG00000175581 | MRPL48 |
| ILF3 | ENSG00000008517 | IL32 |
| ILF3 | ENSG00000265293 | ARGFXP2 |
| ILF3 | ENSG00000198625 | MDM4 |
| ILF3 | ENSG00000131351 | HAUS8 |
| ILF3 | ENSG00000164758 | MED30 |
| ILF3 | ENSG00000184661 | CDCA2 |
| ILF3 | ENSG00000186448 | ZNF197 |
| ILF3 | ENSG00000270697 | RP11-381K20.4 |
| ILF3 | ENSG00000116815 | CD58 |
| ILF3 | ENSG00000072071 | LPHN1 |
| ILF3 | ENSG00000133773 | CCDC59 |
| ILF3 | ENSG00000164087 | POC1A |
| ILF3 | ENSG00000136243 | NUPL2 |
| ILF3 | ENSG00000279817 | RP11-386I8.4 |
| ILF3 | ENSG00000226124 | FTCDNL1 |
| ILF3 | ENSG00000130517 | PGPEP1 |
| ILF3 | ENSG00000224687 | RASAL2-AS1 |
| ILF3 | ENSG00000187951 | ARHGAP11B |
| ILF3 | ENSG00000168591 | TMUB2 |
| ILF3 | ENSG00000260093 | RP11-1E4.1 |
| ILF3 | ENSG00000270050 | RP4-769N13.7 |
| ILF3 | ENSG00000111540 | RAB5B |
| ILF3 | ENSG00000146282 | RARS2 |
| ILF3 | ENSG00000274164 | 5S_rRNA |
| ILF3 | ENSG00000159618 | GPR114 |
| ILF3 | ENSG00000113119 | TMCO6 |
| ILF3 | ENSG00000174885 | NLRP6 |
| ILF3 | ENSG00000111669 | TPI1 |
| ILF3 | ENSG00000175104 | TRAF6 |
| ILF3 | ENSG00000200867 | RN7SKP36 |
| ILF3 | ENSG00000200397 | Y_RNA |
| ILF3 | ENSG00000272795 | RP11-602N24.3 |
| ILF3 | ENSG00000232415 | CTB-51J22.1 |
| ILF3 | ENSG00000143473 | KCNH1 |
| ILF3 | ENSG00000264050 | RP11-22N12.2 |
| ILF3 | ENSG00000112335 | SNX3 |
| ILF3 | ENSG00000072954 | TMEM38A |
| ILF3 | ENSG00000242590 | RP11-54O7.14 |
| ILF3 | ENSG00000236472 | AC002401.1 |
| ILF3 | ENSG00000112234 | FBXL4 |
| ILF3 | ENSG00000120437 | ACAT2 |

| ILF3 | ENSG00000253328 | SUMO2P19 |
| --- | --- | --- |
| ILF3 | ENSG00000180398 | MCFD2 |
| ILF3 | ENSG00000152291 | TGOLN2 |
| ILF3 | ENSG00000214135 | AC024560.3 |
| ILF3 | ENSG00000083937 | CHMP2B |
| ILF3 | ENSG00000179918 | SEPHS2 |
| ILF3 | ENSG00000170175 | CHRNB1 |
| ILF3 | ENSG00000176022 | B3GALT6 |
| ILF3 | ENSG00000101654 | RNMT |
| ILF3 | ENSG00000162188 | GNG3 |
| ILF3 | ENSG00000249115 | HAUS5 |
| ILF3 | ENSG00000271236 | SUMO2P15 |
| ILF3 | ENSG00000083845 | RPS5 |
| ILF3 | ENSG00000186298 | PPP1CC |
| ILF3 | ENSG00000179912 | R3HDM2 |
| ILF3 | ENSG00000262686 | AC005356.1 |
| ILF3 | ENSG00000129654 | FOXJ1 |
| ILF3 | ENSG00000138640 | FAM13A |
| ILF3 | ENSG00000207023 | RNU6-975P |
| ILF3 | ENSG00000270972 | RP11-326C3.15 |
| ILF3 | ENSG00000266174 | MIR4666A |
| ILF3 | ENSG00000197982 | C1orf122 |
| ILF3 | ENSG00000115159 | GPD2 |
| ILF3 | ENSG00000179119 | SPTY2D1 |
| ILF3 | ENSG00000133943 | C14orf159 |
| ILF3 | ENSG00000086062 | B4GALT1 |
| ILF3 | ENSG00000166595 | FAM96B |
| ILF3 | ENSG00000223749 | MIR503HG |
| ILF3 | ENSG00000145703 | IQGAP2 |
| ILF3 | ENSG00000103150 | MLYCD |
| ILF3 | ENSG00000127191 | TRAF2 |
| ILF3 | ENSG00000271014 | RP11-717A5.1 |
| ILF3 | ENSG00000270849 | CHCHD2P3 |
| ILF3 | ENSG00000162729 | IGSF8 |
| ILF3 | ENSG00000066322 | ELOVL1 |
| ILF3 | ENSG00000163536 | SERPINI1 |
| ILF3 | ENSG00000243539 | RN7SL649P |
| ILF3 | ENSG00000120215 | MLANA |
| ILF3 | ENSG00000091140 | DLD |
| ILF3 | ENSG00000264408 | MIR4470 |
| ILF3 | ENSG00000228638 | FCF1P2 |
| ILF3 | ENSG00000136881 | BAAT |
| ILF3 | ENSG00000173113 | TRMT112 |
| ILF3 | ENSG00000274605 | RP11-12G12.7 |
| ILF3 | ENSG00000223711 | AC091633.3 |
| ILF3 | ENSG00000264735 | RP11-498C9.17 |
| ILF3 | ENSG00000198276 | UCKL1 |
| ILF3 | ENSG00000180340 | FZD2 |
| ILF3 | ENSG00000169446 | MMGT1 |
| ILF3 | ENSG00000116525 | TRIM62 |
| ILF3 | ENSG00000265213 | MIR3684 |

| ILF3 | ENSG00000213347 | MXD3 |
| --- | --- | --- |
| ILF3 | ENSG00000244436 | RP11-428G5.1 |
| ILF3 | ENSG00000166856 | GPR182 |
| ILF3 | ENSG00000143373 | ZNF687 |
| ILF3 | ENSG00000116691 | MIIP |
| ILF3 | ENSG00000182872 | RBM10 |
| ILF3 | ENSG00000223504 | RP11-542F9.1 |
| ILF3 | ENSG00000164692 | COL1A2 |
| ILF3 | ENSG00000139668 | WDFY2 |
| ILF3 | ENSG00000256646 | PSMA2 |
| ILF3 | ENSG00000270084 | GAS5-AS1 |
| ILF3 | ENSG00000102001 | CACNA1F |
| ILF3 | ENSG00000140943 | MBTPS1 |
| ILF3 | ENSG00000114316 | USP4 |
| ILF3 | ENSG00000163807 | KIAA1143 |
| ILF3 | ENSG00000267698 | AC002116.7 |
| ILF3 | ENSG00000235989 | MORC2-AS1 |
| ILF3 | ENSG00000276255 | RP5-881P19.7 |
| ILF3 | ENSG00000113621 | TXNDC15 |
| ILF3 | ENSG00000199289 | RNU6-502P |
| ILF3 | ENSG00000136156 | ITM2B |
| ILF3 | ENSG00000149927 | DOC2A |
| ILF3 | ENSG00000272256 | RP11-489E7.4 |
| ILF3 | ENSG00000160117 | ANKLE1 |
| ILF3 | ENSG00000272971 | RP11-284F21.11 |
| ILF3 | ENSG00000269246 | CTC-246B18.10 |
| ILF3 | ENSG00000240294 | RN7SKP241 |
| ILF3 | ENSG00000163166 | IWS1 |
| ILF3 | ENSG00000207613 | MIR181C |
| ILF3 | ENSG00000143379 | SETDB1 |
| ILF3 | ENSG00000171115 | GIMAP8 |
| ILF3 | ENSG00000131626 | PPFIA1 |
| ILF3 | ENSG00000125447 | GGA3 |
| ILF3 | ENSG00000103335 | PIEZO1 |
| ILF3 | ENSG00000234457 | AC006960.5 |
| ILF3 | ENSG00000074054 | CLASP1 |
| ILF3 | ENSG00000267095 | CTD-2319I12.5 |
| ILF3 | ENSG00000160285 | LSS |
| ILF3 | ENSG00000138688 | KIAA1109 |
| ILF3 | ENSG00000243829 | CTB-33G10.1 |
| ILF3 | ENSG00000171130 | ATP6V0E2 |
| ILF3 | ENSG00000275942 | MIAT_exon5_2 |
| ILF3 | ENSG00000136045 | PWP1 |
| ILF3 | ENSG00000163866 | SMIM12 |
| ILF3 | ENSG00000170525 | PFKFB3 |
| ILF3 | ENSG00000182685 | BRICD5 |
| ILF3 | ENSG00000228623 | ZNF883 |
| ILF3 | ENSG00000167969 | ECI1 |
| ILF3 | ENSG00000171865 | RNASEH1 |
| ILF3 | ENSG00000226332 | RP11-157P1.4 |
| ILF3 | ENSG00000148290 | SURF1 |

| ILF3 | ENSG00000254373 | RP11-34P1.2 |
| --- | --- | --- |
| ILF3 | ENSG00000160294 | MCM3AP |
| ILF3 | ENSG00000197912 | SPG7 |
| ILF3 | ENSG00000113649 | TCERG1 |
| ILF3 | ENSG00000166886 | NAB2 |
| ILF3 | ENSG00000217707 | SERPINB8P1 |
| ILF3 | ENSG00000267395 | AC074212.6 |
| ILF3 | ENSG00000259773 | RP11-507J18.2 |
| ILF3 | ENSG00000072736 | NFATC3 |
| ILF3 | ENSG00000255478 | RP11-867O8.5 |
| ILF3 | ENSG00000267070 | RP11-10K17.6 |
| ILF3 | ENSG00000276470 | NPPA-AS1_1 |
| ILF3 | ENSG00000186687 | LYRM7 |
| ILF3 | ENSG00000267048 | RP11-566K11.7 |
| ILF3 | ENSG00000260282 | EIF4EBP2P2 |
| ILF3 | ENSG00000267436 | AC005786.7 |
| ILF3 | ENSG00000243964 | RP11-16F15.1 |
| ILF3 | ENSG00000200164 | Y_RNA |
| ILF3 | ENSG00000139505 | MTMR6 |
| ILF3 | ENSG00000228703 | RP5-1160K1.6 |
| ILF3 | ENSG00000008838 | MED24 |
| ILF3 | ENSG00000101407 | TTI1 |
| ILF3 | ENSG00000103148 | NPRL3 |
| ILF3 | ENSG00000197580 | BCO2 |
| ILF3 | ENSG00000261773 | WI2-89031B12.1 |
| ILF3 | ENSG00000167965 | MLST8 |
| ILF3 | ENSG00000260635 | RP11-21M24.3 |
| ILF3 | ENSG00000003400 | CASP10 |
| ILF3 | ENSG00000087095 | NLK |
| ILF3 | ENSG00000234685 | NUS1P2 |
| ILF3 | ENSG00000160838 | LRRC71 |
| ILF3 | ENSG00000131462 | TUBG1 |
| ILF3 | ENSG00000184319 | RPL23AP82 |
| ILF3 | ENSG00000273142 | RP11-458F8.4 |
| ILF3 | ENSG00000184144 | CNTN2 |
| ILF3 | ENSG00000205808 | PPAPDC2 |
| ILF3 | ENSG00000175513 | TSGA10IP |
| ILF3 | ENSG00000225770 | AC092933.3 |
| ILF3 | ENSG00000115758 | ODC1 |
| ILF3 | ENSG00000164091 | WDR82 |
| ILF3 | ENSG00000055070 | SZRD1 |
| ILF3 | ENSG00000228940 | RP11-348A7.1 |
| ILF3 | ENSG00000112877 | CEP72 |
| ILF3 | ENSG00000272444 | RP11-1017G21.6 |
| ILF3 | ENSG00000230702 | RP11-681L4.1 |
| ILF3 | ENSG00000113282 | CLINT1 |
| ILF3 | ENSG00000112972 | HMGCS1 |
| ILF3 | ENSG00000145220 | LYAR |
| ILF3 | ENSG00000260979 | RP11-77H9.8 |
| ILF3 | ENSG00000223478 | RP11-545E17.3 |
| ILF3 | ENSG00000155542 | SETD9 |

| ILF3 | ENSG00000186162 | CIDECP |
| --- | --- | --- |
| ILF3 | ENSG00000173486 | FKBP2 |
| ILF3 | ENSG00000167131 | CCDC103 |
| ILF3 | ENSG00000178082 | TWF1P1 |
| ILF3 | ENSG00000254087 | LYN |
| ILF3 | ENSG00000163482 | STK36 |
| ILF3 | ENSG00000074800 | ENO1 |
| ILF3 | ENSG00000158055 | GRHL3 |
| ILF3 | ENSG00000168765 | GSTM4 |
| ILF3 | ENSG00000204267 | TAP2 |
| ILF3 | ENSG00000128683 | GAD1 |
| ILF3 | ENSG00000234684 | SDCBP2-AS1 |
| ILF3 | ENSG00000215271 | HOMEZ |
| ILF3 | ENSG00000197561 | ELANE |
| ILF3 | ENSG00000204219 | TCEA3 |
| ILF3 | ENSG00000267577 | CTD- 2587H24.5 |
| ILF3 | ENSG00000180353 | HCLS1 |
| ILF3 | ENSG00000151150 | ANK3 |
| ILF3 | ENSG00000237950 | RP11-7O11.3 |
| ILF3 | ENSG00000267262 | CTC-232P5.3 |
| ILF3 | ENSG00000262117 | BCAR4 |
| ILF3 | ENSG00000141013 | GAS8 |
| ILF3 | ENSG00000160783 | PMF1 |
| ILF3 | ENSG00000119689 | DLST |
| ILF3 | ENSG00000168060 | NAALADL1 |
| ILF3 | ENSG00000265408 | RP11-361L15.4 |
| ILF3 | ENSG00000130702 | LAMA5 |
| ILF3 | ENSG00000015532 | XYLT2 |
| ILF3 | ENSG00000279107 | RP11-823P9.4 |
| ILF3 | ENSG00000212719 | C17orf51 |
| ILF3 | ENSG00000167123 | CERCAM |
| ILF3 | ENSG00000245719 | RP11-34F13.2 |
| ILF3 | ENSG00000267261 | CTD- |
| ILF3 | ENSG00000153574 | 2132N18.3  RPIA |
| ILF3 | ENSG00000188167 | TMPPE |
| ILF3 | ENSG00000203724 | C1orf53 |
| ILF3 | ENSG00000228487 | RP13-225O21.2 |
| ILF3 | ENSG00000249483 | CTD- |
| ILF3 | ENSG00000276931 | 2249K22.1  RP11-161M6.6 |
| ILF3 | ENSG00000273986 | Metazoa_SRP |
| ILF3 | ENSG00000226312 | CFLAR-AS1 |
| ILF3 | ENSG00000219355 | RPL31P52 |
| ILF3 | ENSG00000213726 | RPS2P52 |
| ILF3 | ENSG00000184451 | CCR10 |
| ILF3 | ENSG00000243029 | RN7SL635P |
| ILF3 | ENSG00000262468 | RP11-95P2.1 |
| ILF3 | ENSG00000140497 | SCAMP2 |
| ILF3 | ENSG00000203362 | RP3-337H4.8 |
| ILF3 | ENSG00000065970 | FOXJ2 |

| ILF3 | ENSG00000127804 | METTL16 |
| --- | --- | --- |
| ILF3 | ENSG00000271586 | RP11-52M17.1 |

| ILF3 | ENSG00000069966 | GNB5 |
| --- | --- | --- |
| ILF3 | ENSG00000115392 | FANCL |
| ILF3 | ENSG00000102144 | PGK1 |
| ILF3 | ENSG00000173786 | CNP |
| ILF3 | ENSG00000245904 | RP11-796E2.4 |
| ILF3 | ENSG00000116260 | QSOX1 |
| ILF3 | ENSG00000260196 | RP1-239B22.5 |
| ILF3 | ENSG00000240616 | AD000092.3 |
| ILF3 | ENSG00000115718 | PROC |
| ILF3 | ENSG00000275700 | AATF |
| ILF3 | ENSG00000236582 | PRPF38AP2 |
| ILF3 | ENSG00000140263 | SORD |
| ILF3 | ENSG00000079277 | MKNK1 |
| ILF3 | ENSG00000237476 | XXbac- |
| ILF3 | ENSG00000039987 | B135H6.15  BEST2 |
| ILF3 | ENSG00000224424 | PRKAR2A-AS1 |
| ILF3 | ENSG00000231519 | AC007285.7 |
| ILF3 | ENSG00000254424 | RP11-810P12.6 |
| ILF3 | ENSG00000168434 | COG7 |
| ILF3 | ENSG00000177359 | RP11-551L14.1 |
| ILF3 | ENSG00000170485 | NPAS2 |
| ILF3 | ENSG00000275052 | SMEK2 |
| ILF3 | ENSG00000207579 | MIR662 |
| ILF3 | ENSG00000226291 | AC091729.8 |
| ILF3 | ENSG00000276180 | HIST1H4I |
| ILF3 | ENSG00000259720 | RP11-348B17.1 |
| ILF3 | ENSG00000122386 | ZNF205 |
| ILF3 | ENSG00000272509 | RP11-347C18.5 |
| ILF3 | ENSG00000103245 | NARFL |
| ILF3 | ENSG00000278126 | RP11-454E5.4 |
| ILF3 | ENSG00000130222 | GADD45G |
| ILF3 | ENSG00000147905 | ZCCHC7 |
| ILF3 | ENSG00000255498 | RP11-618K13.2 |
| ILF3 | ENSG00000208028 | MIR616 |
| ILF3 | ENSG00000075624 | ACTB |
| ILF3 | ENSG00000108592 | FTSJ3 |
| ILF3 | ENSG00000213593 | TMX2 |
| ILF3 | ENSG00000146263 | MMS22L |
| ILF3 | ENSG00000268601 | AC115522.3 |
| ILF3 | ENSG00000155463 | OXA1L |
| ILF3 | ENSG00000129521 | EGLN3 |
| ILF3 | ENSG00000182179 | UBA7 |
| ILF3 | ENSG00000238251 | RP11-172F4.2 |
| ILF3 | ENSG00000277509 | RP11-944L7.6 |
| ILF3 | ENSG00000270598 | RP11-396C23.3 |
| ILF3 | ENSG00000109320 | NFKB1 |
| ILF3 | ENSG00000182134 | TDRKH |
| ILF3 | ENSG00000274066 | MIR6514 |
| ILF3 | ENSG00000169371 | SNUPN |
| ILF3 | ENSG00000100147 | CCDC134 |
| ILF3 | ENSG00000272017 | RP1-199J3.7 |

| ILF3 | ENSG00000156253 | RWDD2B |
| --- | --- | --- |
| ILF3 | ENSG00000166833 | NAV2 |
| ILF3 | ENSG00000205189 | ZBTB10 |
| ILF3 | ENSG00000136213 | CHST12 |
| ILF3 | ENSG00000118217 | ATF6 |
| ILF3 | ENSG00000238862 | SNORD19B |
| ILF3 | ENSG00000180385 | EMC3-AS1 |
| ILF3 | ENSG00000102984 | ZNF821 |
| ILF3 | ENSG00000143067 | ZNF697 |
| ILF3 | ENSG00000204568 | MRPS18B |
| ILF3 | ENSG00000160471 | COX6B2 |
| ILF3 | ENSG00000197302 | ZNF720 |
| ILF3 | ENSG00000222365 | SNORD12B |
| ILF3 | ENSG00000236107 | AC010127.3 |
| ILF3 | ENSG00000175352 | NRIP3 |
| ILF3 | ENSG00000005022 | SLC25A5 |
| ILF3 | ENSG00000106351 | AGFG2 |
| ILF3 | ENSG00000244151 | RP11-148K1.12 |
| ILF3 | ENSG00000267432 | DNAH17-AS1 |
| ILF3 | ENSG00000120694 | HSPH1 |
| ILF3 | ENSG00000104827 | CGB |
| ILF3 | ENSG00000171202 | TMEM126A |
| ILF3 | ENSG00000230064 | RP11-244N20.7 |
| ILF3 | ENSG00000136643 | RPS6KC1 |
| ILF3 | ENSG00000064313 | TAF2 |
| ILF3 | ENSG00000168438 | CDC40 |
| ILF3 | ENSG00000099864 | PALM |
| ILF3 | ENSG00000119004 | CYP20A1 |
| ILF3 | ENSG00000162006 | MSLNL |
| ILF3 | ENSG00000132330 | SCLY |
| ILF3 | ENSG00000236514 | RP11-162G10.5 |
| ILF3 | ENSG00000169403 | PTAFR |
| ILF3 | ENSG00000115993 | TRAK2 |
| ILF3 | ENSG00000100263 | RHBDD3 |
| ILF3 | ENSG00000185432 | METTL7A |
| ILF3 | ENSG00000267778 | AC004221.2 |
| ILF3 | ENSG00000280161 | CTC-205M6.1 |
| ILF3 | ENSG00000186103 | ARGFX |
| ILF3 | ENSG00000279198 | CTD-2231E14.2 |
| ILF3 | ENSG00000272333 | KMT2B |
| ILF3 | ENSG00000243024 | RPS11P6 |
| ILF3 | ENSG00000168610 | STAT3 |
| ILF3 | ENSG00000136381 | IREB2 |
| ILF3 | ENSG00000113441 | LNPEP |
| ILF3 | ENSG00000269934 | RP5-1139B12.3 |
| ILF3 | ENSG00000268401 | CTC-344H19.4 |
| ILF3 | ENSG00000125814 | NAPB |
| ILF3 | ENSG00000261188 | CTA-445C9.14 |
| ILF3 | ENSG00000104524 | PYCRL |
| ILF3 | ENSG00000267469 | AC005944.2 |
| ILF3 | ENSG00000257267 | ZNF271P |

| ILF3 | ENSG00000025156 | HSF2 |
| --- | --- | --- |
| ILF3 | ENSG00000188243 | COMMD6 |
| ILF3 | ENSG00000255968 | RP11-513G19.1 |
| ILF3 | ENSG00000149100 | EIF3M |
| ILF3 | ENSG00000203356 | LINC01562 |
| ILF3 | ENSG00000166192 | SENP8 |
| ILF3 | ENSG00000204278 | TMEM235 |
| ILF3 | ENSG00000105122 | RASAL3 |
| ILF3 | ENSG00000173715 | C11orf80 |
| ILF3 | ENSG00000228809 | RP4-705D16.3 |
| ILF3 | ENSG00000263327 | TAPT1-AS1 |
| ILF3 | ENSG00000185739 | SRL |
| ILF3 | ENSG00000096746 | HNRNPH3 |
| ILF3 | ENSG00000116016 | EPAS1 |
| ILF3 | ENSG00000229931 | RP1-151F17.1 |
| ILF3 | ENSG00000228063 | LYPLAL1-AS1 |
| ILF3 | ENSG00000179029 | TMEM107 |
| ILF3 | ENSG00000105889 | STEAP1B |
| ILF3 | ENSG00000250635 | CTD- |
| ILF3 | ENSG00000213972 | 3224K15.2  RP3-522P13.2 |
| ILF3 | ENSG00000054796 | SPO11 |
| ILF3 | ENSG00000271784 | RP1-28H20.3 |
| ILF3 | ENSG00000180901 | KCTD2 |
| ILF3 | ENSG00000201184 | RNU4-68P |
| ILF3 | ENSG00000258643 | BCL2L2- |
| ILF3 | ENSG00000072310 | PABPN1  SREBF1 |
| ILF3 | ENSG00000176953 | NFATC2IP |
| ILF3 | ENSG00000217702 | RP11-287D1.4 |
| ILF3 | ENSG00000280416 | RP11-361L15.3 |
| ILF3 | ENSG00000107341 | UBE2R2 |
| ILF3 | ENSG00000257315 | ZBED6 |
| ILF3 | ENSG00000167612 | ANKRD33 |
| ILF3 | ENSG00000110047 | EHD1 |
| ILF3 | ENSG00000094631 | HDAC6 |
| ILF3 | ENSG00000159720 | ATP6V0D1 |
| ILF3 | ENSG00000132002 | DNAJB1 |
| ILF3 | ENSG00000107968 | MAP3K8 |
| ILF3 | ENSG00000163161 | ERCC3 |
| ILF3 | ENSG00000124391 | IL17C |
| ILF3 | ENSG00000277232 | GTSE1-AS1 |
| ILF3 | ENSG00000131495 | NDUFA2 |
| ILF3 | ENSG00000038382 | TRIO |
| ILF3 | ENSG00000106692 | FKTN |
| ILF3 | ENSG00000171863 | RPS7 |
| ILF3 | ENSG00000125954 | CHURC1- |
| ILF3 | ENSG00000130803 | FNTB  ZNF317 |
| ILF3 | ENSG00000149634 | SPATA25 |
| ILF3 | ENSG00000258186 | SLC7A5P2 |
| ILF3 | ENSG00000231908 | IDH1-AS1 |

| ILF3 | ENSG00000162298 | SYVN1 |
| --- | --- | --- |
| ILF3 | ENSG00000150093 | ITGB1 |

| ILF3 | ENSG00000137337 | MDC1 |
| --- | --- | --- |
| ILF3 | ENSG00000256537 | RP11-785H5.1 |
| ILF3 | ENSG00000171490 | RSL1D1 |
| ILF3 | ENSG00000237190 | CDKN2AIPNL |
| ILF3 | ENSG00000142546 | NOSIP |
| ILF3 | ENSG00000169372 | CRADD |
| ILF3 | ENSG00000240950 | RP11-372E1.1 |
| ILF3 | ENSG00000235927 | NEXN-AS1 |
| ILF3 | ENSG00000259013 | RP11-1017G21.3 |
| ILF3 | ENSG00000188542 | DUSP28 |
| ILF3 | ENSG00000165943 | MOAP1 |
| ILF3 | ENSG00000260495 | RP11-55K13.1 |
| ILF3 | ENSG00000172071 | EIF2AK3 |
| ILF3 | ENSG00000148488 | ST8SIA6 |
| ILF3 | ENSG00000164300 | SERINC5 |
| ILF3 | ENSG00000143545 | RAB13 |
| ILF3 | ENSG00000101639 | CEP192 |
| ILF3 | ENSG00000105438 | KDELR1 |
| ILF3 | ENSG00000125246 | CLYBL |
| ILF3 | ENSG00000196411 | EPHB4 |
| ILF3 | ENSG00000183665 | TRMT12 |
| ILF3 | ENSG00000053918 | KCNQ1 |
| ILF3 | ENSG00000140416 | TPM1 |
| ILF3 | ENSG00000158290 | CUL4B |
| ILF3 | ENSG00000270557 | RP11-546J1.1 |
| ILF3 | ENSG00000102096 | PIM2 |
| ILF3 | ENSG00000280186 | RP11-483I13.6 |
| ILF3 | ENSG00000167461 | RAB8A |
| ILF3 | ENSG00000111252 | SH2B3 |
| ILF3 | ENSG00000179930 | ZNF648 |
| ILF3 | ENSG00000059122 | FLYWCH1 |
| ILF3 | ENSG00000259970 | AC099668.5 |
| ILF3 | ENSG00000151116 | UEVLD |
| ILF3 | ENSG00000108961 | RANGRF |
| ILF3 | ENSG00000111860 | CEP85L |
| ILF3 | ENSG00000110422 | HIPK3 |
| ILF3 | ENSG00000255339 | RP11-411B6.6 |
| ILF3 | ENSG00000158869 | FCER1G |
| ILF3 | ENSG00000117632 | STMN1 |
| ILF3 | ENSG00000205763 | RP9P |
| ILF3 | ENSG00000167635 | ZNF146 |
| ILF3 | ENSG00000138413 | IDH1 |
| ILF3 | ENSG00000148925 | BTBD10 |
| ILF3 | ENSG00000202092 | RNA5SP190 |
| ILF3 | ENSG00000068079 | IFI35 |
| ILF3 | ENSG00000112874 | NUDT12 |
| ILF3 | ENSG00000245812 | RP11-175K6.1 |
| ILF3 | ENSG00000186017 | ZNF566 |
| ILF3 | ENSG00000128694 | OSGEPL1 |
| ILF3 | ENSG00000198454 | PRR31 |
| ILF3 | ENSG00000273249 | LL09NC01- 251B2.3 |

| ILF3 ILF3 | ENSG00000138279 ENSG00000248682 | ANXA7  ARHGAP22- IT1 |
| --- | --- | --- |
| ILF3 | ENSG00000269194 | AC006942.4 |
| ILF3 | ENSG00000280187 | CTC-351M12.1 |
| ILF3 | ENSG00000197182 | MIRLET7BHG |
| ILF3 | ENSG00000254859 | RP11-661A12.5 |
| ILF3 | ENSG00000202222 | Y_RNA |
| ILF3 | ENSG00000222529 | Y_RNA |
| ILF3 | ENSG00000235263 | RP13-392I16.1 |
| ILF3 | ENSG00000181392 | SYNE4 |
| ILF3 | ENSG00000236591 | RP11-162J8.3 |
| ILF3 | ENSG00000114978 | MOB1A |
| ILF3 | ENSG00000156697 | UTP14A |
| ILF3 | ENSG00000163823 | CCR1 |
| ILF3 | ENSG00000262120 | RP11-510M2.7 |
| ILF3 | ENSG00000006451 | RALA |
| ILF3 | ENSG00000096063 | SRPK1 |
| ILF3 | ENSG00000111300 | NAA25 |
| ILF3 | ENSG00000165733 | BMS1 |
| ILF3 | ENSG00000269843 | CTC-490E21.10 |
| ILF3 | ENSG00000136738 | STAM |
| ILF3 | ENSG00000131504 | DIAPH1 |
| ILF3 | ENSG00000047315 | POLR2B |
| ILF3 | ENSG00000108262 | GIT1 |
| ILF3 | ENSG00000274363 | RP11-104O19.4 |
| ILF3 | ENSG00000187600 | TMEM247 |
| ILF3 | ENSG00000172831 | CES2 |
| ILF3 | ENSG00000175868 | CALCB |
| ILF3 | ENSG00000132881 | RSG1 |
| ILF3 | ENSG00000229462 | AC127383.1 |
| ILF3 | ENSG00000174173 | TRMT10C |
| ILF3 | ENSG00000257359 | RP11-780K2.1 |
| ILF3 | ENSG00000137692 | DCUN1D5 |
| ILF3 | ENSG00000241102 | RP11-286H14.2 |
| ILF3 | ENSG00000163516 | ANKZF1 |
| ILF3 | ENSG00000259349 | RP11-15E18.1 |
| ILF3 | ENSG00000272183 | RP11-523H20.3 |
| ILF3 | ENSG00000235480 | RP11-363D14.1 |
| ILF3 | ENSG00000174576 | NPAS4 |
| ILF3 | ENSG00000167094 | TTC16 |
| ILF3 | ENSG00000103353 | UBFD1 |
| ILF3 | ENSG00000017797 | RALBP1 |
| ILF3 | ENSG00000197461 | PDGFA |
| ILF3 | ENSG00000112144 | ICK |
| ILF3 | ENSG00000077549 | CAPZB |
| ILF3 | ENSG00000105723 | GSK3A |
| ILF3 | ENSG00000146416 | AIG1 |
| ILF3 | ENSG00000225185 | RP11-481K9.4 |
| ILF3 | ENSG00000197980 | LEKR1 |
| ILF3 | ENSG00000183963 | SMTN |
| ILF3 | ENSG00000221038 | RNU6ATAC7P |

| ILF3 | ENSG00000250067 | YJEFN3 |
| --- | --- | --- |
| ILF3 | ENSG00000111816 | FRK |
| ILF3 | ENSG00000260467 | RP11-405F3.4 |
| ILF3 | ENSG00000179456 | ZBTB18 |
| ILF3 | ENSG00000113558 | SKP1 |
| ILF3 | ENSG00000232807 | RP11-536K7.3 |
| ILF3 | ENSG00000205771 | CATSPER2P1 |
| ILF3 | ENSG00000275691 | RP11-249C24.12 |
| ILF3 | ENSG00000143149 | ALDH9A1 |
| ILF3 | ENSG00000097007 | ABL1 |
| ILF3 | ENSG00000207165 | SNORA70 |
| ILF3 | ENSG00000276368 | HIST1H2AJ |
| ILF3 | ENSG00000065135 | GNAI3 |
| ILF3 | ENSG00000178401 | DNAJC22 |
| ILF3 | ENSG00000198056 | PRIM1 |
| ILF3 | ENSG00000101203 | COL20A1 |
| ILF3 | ENSG00000147400 | CETN2 |
| ILF3 | ENSG00000228153 | RP11-23I7.1 |
| ILF3 | ENSG00000136026 | CKAP4 |
| ILF3 | ENSG00000133624 | ZNF767P |
| ILF3 | ENSG00000179698 | KIAA1875 |
| ILF3 | ENSG00000130764 | LRRC47 |
| ILF3 | ENSG00000160404 | TOR2A |
| ILF3 | ENSG00000264278 | RP11-162A12.2 |
| ILF3 | ENSG00000184402 | SS18L1 |
| ILF3 | ENSG00000187244 | BCAM |
| ILF3 | ENSG00000197622 | CDC42SE1 |
| ILF3 | ENSG00000224621 | RP11-276H7.3 |
| ILF3 | ENSG00000253630 | CTC-370J7.1 |
| ILF3 | ENSG00000102901 | CENPT |
| ILF3 | ENSG00000254333 | CTC-367J11.1 |
| ILF3 | ENSG00000270982 | RP11-490D19.11 |
| ILF3 | ENSG00000228486 | LINC01125 |
| ILF3 | ENSG00000276110 | RP4-790G17.7 |
| ILF3 | ENSG00000130254 | SAFB2 |
| ILF3 | ENSG00000261114 | RP11-325K4.2 |
| ILF3 | ENSG00000255858 | RP11-612B6.1 |
| ILF3 | ENSG00000025423 | HSD17B6 |
| ILF3 | ENSG00000123600 | METTL8 |
| ILF3 | ENSG00000201239 | Y_RNA |
| ILF3 | ENSG00000132952 | USPL1 |
| ILF3 | ENSG00000204685 | STARD7-AS1 |
| ILF3 | ENSG00000154079 | C6orf57 |
| ILF3 | ENSG00000174353 | STAG3L3 |
| ILF3 | ENSG00000200646 | Y_RNA |
| ILF3 | ENSG00000100906 | NFKBIA |
| ILF3 | ENSG00000200259 | SNORD35A |
| ILF3 | ENSG00000182004 | SNRPE |
| ILF3 | ENSG00000107672 | NSMCE4A |
| ILF3 | ENSG00000175970 | UNC119B |
| ILF3 | ENSG00000256826 | AC138744.2 |

| ILF3 | ENSG00000147853 | AK3 |
| --- | --- | --- |
| ILF3 | ENSG00000105755 | ETHE1 |
| ILF3 | ENSG00000157881 | PANK4 |
| ILF3 | ENSG00000280323 | AC053503.12 |
| ILF3 | ENSG00000260410 | RP11-505K9.3 |
| ILF3 | ENSG00000142188 | TMEM50B |
| ILF3 | ENSG00000142684 | ZNF593 |
| ILF3 | ENSG00000267165 | RP11-78A19.3 |
| ILF3 | ENSG00000253973 | RP11-467K18.2 |
| ILF3 | ENSG00000124228 | DDX27 |
| ILF3 | ENSG00000173641 | HSPB7 |
| ILF3 | ENSG00000130921 | C12orf65 |
| ILF3 | ENSG00000229097 | CALM2P2 |
| ILF3 | ENSG00000116670 | MAD2L2 |
| ILF3 | ENSG00000240972 | MIF |
| ILF3 | ENSG00000261366 | MANEA-AS1 |
| ILF3 | ENSG00000213057 | C1orf220 |
| ILF3 | ENSG00000101220 | C20orf27 |
| ILF3 | ENSG00000187848 | P2RX2 |
| ILF3 | ENSG00000230295 | RP11-458F8.2 |
| ILF3 | ENSG00000122376 | FAM35A |
| ILF3 | ENSG00000243488 | RN7SL337P |
| ILF3 | ENSG00000248323 | LUCAT1 |
| ILF3 | ENSG00000004939 | SLC4A1 |
| ILF3 | ENSG00000163328 | GPR155 |
| ILF3 | ENSG00000198898 | CAPZA2 |
| ILF3 | ENSG00000266953 | RP11-618P17.4 |
| ILF3 | ENSG00000177674 | AGTRAP |
| ILF3 | ENSG00000279964 | RP11-69J7.1 |
| ILF3 | ENSG00000237668 | RPS15AP38 |
| ILF3 | ENSG00000198814 | GK |
| ILF3 | ENSG00000188511 | C22orf34 |
| ILF3 | ENSG00000238649 | SNORD42A |
| ILF3 | ENSG00000105647 | PIK3R2 |
| ILF3 | ENSG00000205464 | ATP6AP1L |
| ILF3 | ENSG00000137731 | FXYD2 |
| ILF3 | ENSG00000123636 | BAZ2B |
| ILF3 | ENSG00000149743 | TRPT1 |
| ILF3 | ENSG00000264007 | RP11-68I3.10 |
| ILF3 | ENSG00000254928 | RP11-702H23.6 |
| ILF3 | ENSG00000223060 | Y_RNA |
| ILF3 | ENSG00000254911 | SCARNA9 |
| ILF3 | ENSG00000146755 | TRIM50 |
| ILF3 | ENSG00000235169 | SMIM1 |
| ILF3 | ENSG00000168944 | CEP120 |
| ILF3 | ENSG00000260742 | RP11-366L5.1 |
| ILF3 | ENSG00000267459 | AC006116.27 |
| ILF3 | ENSG00000228782 | MRPL45P2 |
| ILF3 | ENSG00000163344 | PMVK |
| ILF3 | ENSG00000160469 | BRSK1 |
| ILF3 | ENSG00000274177 | LLNLR- 284B4.1 |

| ILF3 | ENSG00000132465 | IGJ |
| --- | --- | --- |
| ILF3 | ENSG00000264712 | MIR4504 |
| ILF3 | ENSG00000199953 | RNA5SP443 |
| ILF3 | ENSG00000268866 | AC020915.2 |
| ILF3 | ENSG00000215068 | AC025171.1 |
| ILF3 | ENSG00000255377 | DUXAP5 |
| ILF3 | ENSG00000171606 | ZNF274 |
| ILF3 | ENSG00000275411 | MIR6882 |
| ILF3 | ENSG00000172794 | RAB37 |
| ILF3 | ENSG00000073578 | SDHA |
| ILF3 | ENSG00000002016 | RAD52 |
| ILF3 | ENSG00000120555 | SEPT7P9 |
| ILF3 | ENSG00000105523 | FAM83E |
| ILF3 | ENSG00000158406 | HIST1H4H |
| ILF3 | ENSG00000228451 | SDAD1P1 |
| ILF3 | ENSG00000153922 | CHD1 |
| ILF3 | ENSG00000279331 | RBM12B-AS1 |
| ILF3 | ENSG00000206848 | RNU6-890P |
| ILF3 | ENSG00000140254 | DUOXA1 |
| ILF3 | ENSG00000117407 | ARTN |
| ILF3 | ENSG00000188010 | MORN2 |
| ILF3 | ENSG00000172663 | TMEM134 |
| ILF3 | ENSG00000070669 | ASNS |
| ILF3 | ENSG00000129667 | RHBDF2 |
| ILF3 | ENSG00000105229 | PIAS4 |
| ILF3 | ENSG00000169193 | CCDC126 |
| ILF3 | ENSG00000108591 | DRG2 |
| ILF3 | ENSG00000262136 | CTD-2033A16.3 |
| ILF3 | ENSG00000271414 | RP11-751K21.1 |
| ILF3 | ENSG00000086544 | ITPKC |
| ILF3 | ENSG00000263528 | IKBKE |
| ILF3 | ENSG00000100926 | TM9SF1 |
| ILF3 | ENSG00000259066 | RP11-371E8.4 |
| ILF3 | ENSG00000223797 | ENTPD3-AS1 |
| ILF3 | ENSG00000197380 | DACT3 |
| ILF3 | ENSG00000267633 | CTB-5E10.3 |
| ILF3 | ENSG00000265089 | MIR4655 |
| ILF3 | ENSG00000254602 | AP000662.4 |
| ILF3 | ENSG00000274528 | CTD-2650P22.2 |
| ILF3 | ENSG00000225808 | DNAJC19P5 |
| ILF3 | ENSG00000130159 | ECSIT |
| ILF3 | ENSG00000004866 | ST7 |
| ILF3 | ENSG00000206921 | RNU6-481P |
| ILF3 | ENSG00000207005 | RNU1-2 |
| ILF3 | ENSG00000132017 | DCAF15 |
| ILF3 | ENSG00000197272 | IL27 |
| ILF3 | ENSG00000266995 | RP11-703I16.3 |
| ILF3 | ENSG00000239306 | RBM14 |
| ILF3 | ENSG00000141279 | NPEPPS |
| ILF3 | ENSG00000130656 | HBZ |
| ILF3 | ENSG00000272752 | STAG3L5P-PVRIG2P- PILRB |

| ILF3 | ENSG00000136448 | NMT1 |
| --- | --- | --- |
| ILF3 | ENSG00000267968 | AC011523.2 |
| ILF3 | ENSG00000078808 | SDF4 |
| ILF3 | ENSG00000138757 | G3BP2 |
| ILF3 | ENSG00000070061 | IKBKAP |
| ILF3 | ENSG00000163093 | BBS5 |
| ILF3 | ENSG00000186814 | ZSCAN30 |
| ILF3 | ENSG00000271092 | TMEM56- |
| ILF3 | ENSG00000116918 | RWDD3  TSNAX |
| ILF3 | ENSG00000175287 | PHYHD1 |
| ILF3 | ENSG00000251136 | RP11-37B2.1 |
| ILF3 | ENSG00000067533 | RRP15 |
| ILF3 | ENSG00000133065 | SLC41A1 |
| ILF3 | ENSG00000260238 | PMF1-BGLAP |
| ILF3 | ENSG00000214259 | RP11-568J23.1 |
| ILF3 | ENSG00000202360 | RN7SKP249 |
| ILF3 | ENSG00000204138 | PHACTR4 |
| ILF3 | ENSG00000210825 | SNORA40 |
| ILF3 | ENSG00000125037 | EMC3 |
| ILF3 | ENSG00000130962 | PRRG1 |
| ILF3 | ENSG00000166783 | KIAA0430 |
| ILF3 | ENSG00000201643 | SNORA14A |
| ILF3 | ENSG00000159348 | CYB5R1 |
| ILF3 | ENSG00000125347 | IRF1 |
| ILF3 | ENSG00000257496 | RP11-474P2.4 |
| ILF3 | ENSG00000073614 | KDM5A |
| ILF3 | ENSG00000241933 | RP11-755B10.3 |
| ILF3 | ENSG00000254443 | RP11-304C12.3 |
| ILF3 | ENSG00000222890 | RNU6-1068P |
| ILF3 | ENSG00000152492 | CCDC50 |
| ILF3 | ENSG00000165609 | NUDT5 |
| ILF3 | ENSG00000076662 | ICAM3 |
| ILF3 | ENSG00000113048 | MRPS27 |
| ILF3 | ENSG00000020922 | MRE11A |
| ILF3 | ENSG00000130943 | PKDREJ |
| ILF3 | ENSG00000276823 | Metazoa_SRP |
| ILF3 | ENSG00000271732 | RP5-1182A14.5 |
| ILF3 | ENSG00000110375 | UPK2 |
| ILF3 | ENSG00000136870 | ZNF189 |
| ILF3 | ENSG00000185947 | ZNF267 |
| ILF3 | ENSG00000184924 | PTRHD1 |
| ILF3 | ENSG00000204147 | ASAH2B |
| ILF3 | ENSG00000156467 | UQCRB |
| ILF3 | ENSG00000090661 | CERS4 |
| ILF3 | ENSG00000266107 | MIR4525 |
| ILF3 | ENSG00000135336 | ORC3 |
| ILF3 | ENSG00000172247 | C1QTNF4 |
| ILF3 | ENSG00000167183 | PRR15L |
| ILF3 | ENSG00000106153 | CHCHD2 |
| ILF3 | ENSG00000125149 | C16orf70 |
| ILF3 | ENSG00000143815 | LBR |

| ILF3 | ENSG00000100079 | LGALS2 |
| --- | --- | --- |
| ILF3 | ENSG00000181218 | HIST3H2A |
| ILF3 | ENSG00000109618 | SEPSECS |
| ILF3 | ENSG00000231521 | RP11-244N9.4 |
| ILF3 | ENSG00000236024 | PRRX2-AS1 |
| ILF3 | ENSG00000261045 | RP11-673P17.4 |
| ILF3 | ENSG00000275207 | MIR6740 |
| ILF3 | ENSG00000243789 | JMJD7 |
| ILF3 | ENSG00000223810 | KRT8P28 |
| ILF3 | ENSG00000087365 | SF3B2 |
| ILF3 | ENSG00000138758 | SEPT11 |
| ILF3 | ENSG00000173064 | HECTD4 |
| ILF3 | ENSG00000261575 | RP11-259G18.1 |
| ILF3 | ENSG00000182054 | IDH2 |
| ILF3 | ENSG00000224818 | RP11-134G8.10 |
| ILF3 | ENSG00000128285 | MCHR1 |
| ILF3 | ENSG00000271483 | RP11-384A12.1 |
| ILF3 | ENSG00000137078 | SIT1 |
| ILF3 | ENSG00000102858 | MGRN1 |
| ILF3 | ENSG00000089820 | ARHGAP4 |
| ILF3 | ENSG00000163611 | SPICE1 |
| ILF3 | ENSG00000185112 | FAM43A |
| ILF3 | ENSG00000176884 | GRIN1 |
| ILF3 | ENSG00000201340 | Y_RNA |
| ILF3 | ENSG00000213689 | TREX1 |
| ILF3 | ENSG00000223300 | Y_RNA |
| ILF3 | ENSG00000005020 | SKAP2 |
| ILF3 | ENSG00000169660 | HEXDC |
| ILF3 | ENSG00000143891 | GALM |
| ILF3 | ENSG00000234465 | PINLYP |
| ILF3 | ENSG00000036549 | ZZZ3 |
| ILF3 | ENSG00000165512 | ZNF22 |
| ILF3 | ENSG00000126233 | SLURP1 |
| ILF3 | ENSG00000236846 | RP11-239E10.2 |
| ILF3 | ENSG00000238793 | SNORD124 |
| ILF3 | ENSG00000095383 | TBC1D2 |
| ILF3 | ENSG00000229390 | MICD |
| ILF3 | ENSG00000272273 | XXbac- |
| ILF3 | ENSG00000218806 | BPG252P9.10 RP3-369A17.2 |
| ILF3 | ENSG00000152082 | MZT2B |
| ILF3 | ENSG00000177613 | CSTF2T |
| ILF3 | ENSG00000167703 | SLC43A2 |
| ILF3 | ENSG00000221116 | SNORD110 |
| ILF3 | ENSG00000144671 | SLC22A14 |
| ILF3 | ENSG00000204472 | AIF1 |
| ILF3 | ENSG00000165025 | SYK |
| ILF3 | ENSG00000183935 | HTR7P1 |
| ILF3 | ENSG00000091947 | TMEM101 |
| ILF3 | ENSG00000265064 | MIR4692 |
| ILF3 | ENSG00000183617 | MRPL54 |
| ILF3 | ENSG00000234705 | HMGA1P4 |

| ILF3 | ENSG00000253958 | CLDN23 |
| --- | --- | --- |
| ILF3 | ENSG00000113721 | PDGFRB |
| ILF3 | ENSG00000213462 | ERV3-1 |
| ILF3 | ENSG00000112081 | SRSF3 |
| ILF3 | ENSG00000177943 | MAMDC4 |
| ILF3 | ENSG00000200737 | Y_RNA |
| ILF3 | ENSG00000273765 | RP11-370I10.11 |
| ILF3 | ENSG00000182986 | ZNF320 |
| ILF3 | ENSG00000230366 | DSCR9 |
| ILF3 | ENSG00000005007 | UPF1 |
| ILF3 | ENSG00000260021 | RP11-480I12.10 |
| ILF3 | ENSG00000267359 | RP11-1094M14.12 |
| ILF3 | ENSG00000245598 | DACT3-AS1 |
| ILF3 | ENSG00000169967 | MAP3K2 |
| ILF3 | ENSG00000239825 | RN7SL549P |
| ILF3 | ENSG00000132004 | FBXW9 |
| ILF3 | ENSG00000141367 | CLTC |
| ILF3 | ENSG00000254973 | RP11-429J17.7 |
| ILF3 | ENSG00000061794 | MRPS35 |
| ILF3 | ENSG00000264765 | RP11-92B11.4 |
| ILF3 | ENSG00000102910 | LONP2 |
| ILF3 | ENSG00000234222 | RP11-315I20.1 |
| ILF3 | ENSG00000170959 | DCDC1 |
| ILF3 | ENSG00000264500 | MIR3124 |
| ILF3 | ENSG00000160785 | SLC25A44 |
| ILF3 | ENSG00000153006 | SREK1IP1 |
| ILF3 | ENSG00000137955 | RABGGTB |
| ILF3 | ENSG00000141576 | RNF157 |
| ILF3 | ENSG00000072571 | HMMR |
| ILF3 | ENSG00000196510 | ANAPC7 |
| ILF3 | ENSG00000172172 | MRPL13 |
| ILF3 | ENSG00000181409 | AATK |
| ILF3 | ENSG00000230269 | RP1-40E16.9 |
| ILF3 | ENSG00000257108 | NHLRC4 |
| ILF3 | ENSG00000147526 | TACC1 |
| ILF3 | ENSG00000168286 | THAP11 |
| ILF3 | ENSG00000261404 | AC009120.4 |
| ILF3 | ENSG00000156345 | CDK20 |
| ILF3 | ENSG00000172508 | CARNS1 |
| ILF3 | ENSG00000204528 | PSORS1C3 |
| ILF3 | ENSG00000213128 | RPL32P31 |
| ILF3 | ENSG00000115306 | SPTBN1 |
| ILF3 | ENSG00000278030 | TRBV7-9 |
| ILF3 | ENSG00000234761 | RP11-38O14.5 |
| ILF3 | ENSG00000252174 | RNU7-18P |
| ILF3 | ENSG00000103942 | HOMER2 |
| ILF3 | ENSG00000179965 | ZNF771 |
| ILF3 | ENSG00000105467 | SYNGR4 |
| ILF3 | ENSG00000105976 | MET |
| ILF3 | ENSG00000103168 | TAF1C |
| ILF3 | ENSG00000015479 | MATR3 |

| ILF3 | ENSG00000189030 | VHLL |
| --- | --- | --- |
| ILF3 | ENSG00000136319 | TTC5 |
| ILF3 | ENSG00000231203 | KRT8P10 |
| ILF3 | ENSG00000201801 | RNU5E-4P |
| ILF3 | ENSG00000177885 | GRB2 |
| ILF3 | ENSG00000141858 | SAMD1 |
| ILF3 | ENSG00000106993 | CDC37L1 |
| ILF3 | ENSG00000182481 | KPNA2 |
| ILF3 | ENSG00000129559 | NEDD8 |
| ILF3 | ENSG00000271360 | RP11-138I18.1 |
| ILF3 | ENSG00000237424 | FOXD2-AS1 |
| ILF3 | ENSG00000234585 | CCT6P3 |
| ILF3 | ENSG00000251387 | CTB-35F21.3 |
| ILF3 | ENSG00000136842 | TMOD1 |
| ILF3 | ENSG00000166452 | AKIP1 |
| ILF3 | ENSG00000175611 | LINC00476 |
| ILF3 | ENSG00000253805 | RP11-1049H7.2 |
| ILF3 | ENSG00000174516 | PELI3 |
| ILF3 | ENSG00000206344 | HCG27 |
| ILF3 | ENSG00000119688 | ABCD4 |
| ILF3 | ENSG00000181027 | FKRP |
| ILF3 | ENSG00000212493 | SNORD19 |
| ILF3 | ENSG00000148337 | CIZ1 |
| ILF3 | ENSG00000086717 | PPEF1 |
| ILF3 | ENSG00000137944 | CCBL2 |
| ILF3 | ENSG00000042753 | AP2S1 |
| ILF3 | ENSG00000091640 | SPAG7 |
| ILF3 | ENSG00000074621 | SLC24A1 |
| ILF3 | ENSG00000125944 | HNRNPR |
| ILF3 | ENSG00000214812 | RP5-1053E7.3 |
| ILF3 | ENSG00000266821 | RP11-6N17.2 |
| ILF3 | ENSG00000279196 | RP11-1072A3.3 |
| ILF3 | ENSG00000264511 | MIR3678 |
| ILF3 | ENSG00000101343 | CRNKL1 |
| ILF3 | ENSG00000265675 | RN7SL708P |
| ILF3 | ENSG00000265991 | MIR4519 |
| ILF3 | ENSG00000119333 | WDR34 |
| ILF3 | ENSG00000228069 | RP11-100G15.3 |
| ILF3 | ENSG00000224794 | RP3-333H23.8 |
| ILF3 | ENSG00000261796 | ISY1-RAB43 |
| ILF3 | ENSG00000170037 | CNTROB |
| ILF3 | ENSG00000111885 | MAN1A1 |
| ILF3 | ENSG00000075945 | KIFAP3 |
| ILF3 | ENSG00000158941 | CCAR2 |
| ILF3 | ENSG00000250709 | CCDC169- |
| ILF3 | ENSG00000106992 | SOHLH2  AK1 |
| ILF3 | ENSG00000196118 | C16orf93 |
| ILF3 | ENSG00000197734 | C14orf178 |
| ILF3 | ENSG00000249693 | THEGL |
| ILF3 | ENSG00000267757 | C19orf83 |
| ILF3 | ENSG00000258466 | RP11-1012A1.4 |

| ILF3 | ENSG00000250948 | RP11-1079K10.2 |
| --- | --- | --- |
| ILF3 | ENSG00000234492 | RPL34-AS1 |
| ILF3 | ENSG00000078804 | TP53INP2 |
| ILF3 | ENSG00000230063 | RP11-384C4.6 |
| ILF3 | ENSG00000112118 | MCM3 |
| ILF3 | ENSG00000108561 | C1QBP |
| ILF3 | ENSG00000140983 | RHOT2 |
| ILF3 | ENSG00000138802 | SEC24B |
| ILF3 | ENSG00000260565 | ERVK13-1 |
| ILF3 | ENSG00000260643 | RP11-303E16.8 |
| ILF3 | ENSG00000240898 | RP11-48B14.1 |
| ILF3 | ENSG00000254317 | RP11-473O4.5 |
| ILF3 | ENSG00000226174 | TEX22 |
| ILF3 | ENSG00000236307 | AC104651.1 |
| ILF3 | ENSG00000128016 | ZFP36 |
| ILF3 | ENSG00000278677 | HIST1H2AM |
| ILF3 | ENSG00000244270 | RPL32P29 |
| ILF3 | ENSG00000071909 | MYO3B |
| ILF3 | ENSG00000200156 | RNU5B-1 |
| ILF3 | ENSG00000114054 | PCCB |
| ILF3 | ENSG00000164366 | CCDC127 |
| ILF3 | ENSG00000117133 | RPF1 |
| ILF3 | ENSG00000233822 | HIST1H2BN |
| ILF3 | ENSG00000156313 | RPGR |
| ILF3 | ENSG00000162849 | KIF26B |
| ILF3 | ENSG00000011600 | TYROBP |
| ILF3 | ENSG00000126775 | ATG14 |
| ILF3 | ENSG00000255154 | RPP14 |
| ILF3 | ENSG00000139131 | YARS2 |
| ILF3 | ENSG00000257621 | FLJ31306 |
| ILF3 | ENSG00000141316 | SPACA3 |
| ILF3 | ENSG00000174442 | ZWILCH |
| ILF3 | ENSG00000164934 | DCAF13 |
| ILF3 | ENSG00000175984 | DENND2C |
| ILF3 | ENSG00000113734 | BNIP1 |
| ILF3 | ENSG00000224574 | COL18A1-AS2 |
| ILF3 | ENSG00000224797 | RP11-57C19.6 |
| ILF3 | ENSG00000231663 | RP5-827C21.4 |
| ILF3 | ENSG00000129757 | CDKN1C |
| ILF3 | ENSG00000171443 | ZNF524 |
| ILF3 | ENSG00000255883 | RP11-79P5.10 |
| ILF3 | ENSG00000180209 | MYLPF |
| ILF3 | ENSG00000164031 | DNAJB14 |
| ILF3 | ENSG00000145901 | TNIP1 |
| ILF3 | ENSG00000167525 | PROCA1 |
| ILF3 | ENSG00000177051 | FBXO46 |
| ILF3 | ENSG00000143178 | TBX19 |
| ILF3 | ENSG00000271547 | RP11-579D7.8 |
| ILF3 | ENSG00000132718 | SYT11 |
| ILF3 | ENSG00000166359 | WDR88 |
| ILF3 | ENSG00000148219 | ASTN2 |

| ILF3 | ENSG00000160072 | ATAD3B |
| --- | --- | --- |
| ILF3 | ENSG00000258366 | RTEL1 |
| ILF3 | ENSG00000202337 | RNU6-8 |
| ILF3 | ENSG00000138468 | SENP7 |
| ILF3 | ENSG00000263050 | RP11-667K14.3 |
| ILF3 | ENSG00000214546 | AC087491.2 |
| ILF3 | ENSG00000232445 | RP11-132A1.4 |
| ILF3 | ENSG00000279136 | LA16c-335H7.2 |
| ILF3 | ENSG00000104808 | DHDH |
| ILF3 | ENSG00000229152 | ANKRD10-IT1 |
| ILF3 | ENSG00000255467 | RP11-144G7.2 |
| ILF3 | ENSG00000129493 | HEATR5A |
| ILF3 | ENSG00000150455 | TIRAP |
| ILF3 | ENSG00000236893 | ASS1P7 |
| ILF3 | ENSG00000133818 | RRAS2 |
| ILF3 | ENSG00000105063 | PPP6R1 |
| ILF3 | ENSG00000202569 | MIR146B |
| ILF3 | ENSG00000273802 | HIST1H2BG |
| ILF3 | ENSG00000112651 | MRPL2 |
| ILF3 | ENSG00000226287 | TMEM191A |
| ILF3 | ENSG00000270775 | AP000436.4 |
| ILF3 | ENSG00000275523 | MIR6508 |
| ILF3 | ENSG00000058729 | RIOK2 |
| ILF3 | ENSG00000178229 | ZNF543 |
| ILF3 | ENSG00000235098 | ANKRD65 |
| ILF3 | ENSG00000157764 | BRAF |
| ILF3 | ENSG00000252526 | SNORA70 |
| ILF3 | ENSG00000212521 | RNU6-918P |
| ILF3 | ENSG00000260830 | RP11-524O1.4 |
| ILF3 | ENSG00000099381 | SETD1A |
| ILF3 | ENSG00000134375 | TIMM17A |
| ILF3 | ENSG00000272092 | RP11-350N15.5 |
| ILF3 | ENSG00000211575 | MIR760 |
| ILF3 | ENSG00000187051 | RPS19BP1 |
| ILF3 | ENSG00000268790 | CTC-429P9.4 |
| ILF3 | ENSG00000102699 | PARP4 |
| ILF3 | ENSG00000270006 | RP11-178L8.7 |
| ILF3 | ENSG00000139182 | CLSTN3 |
| ILF3 | ENSG00000183020 | AP2A2 |
| ILF3 | ENSG00000114491 | UMPS |
| ILF3 | ENSG00000085831 | TTC39A |
| ILF3 | ENSG00000143443 | C1orf56 |
| ILF3 | ENSG00000231906 | RP11-541M12.3 |
| ILF3 | ENSG00000265724 | MIR4284 |
| ILF3 | ENSG00000179240 | RP11-111M22.2 |
| ILF3 | ENSG00000255112 | CHMP1B |
| ILF3 | ENSG00000068024 | HDAC4 |
| ILF3 | ENSG00000259424 | RP11-35O15.1 |
| ILF3 | ENSG00000221838 | AP4M1 |
| ILF3 | ENSG00000259584 | RP11-521C20.2 |
| ILF3 | ENSG00000146966 | DENND2A |

| ILF3 | ENSG00000051382 | PIK3CB |
| --- | --- | --- |
| ILF3 | ENSG00000186073 | C15orf41 |
| ILF3 | ENSG00000235560 | AC002310.12 |
| ILF3 | ENSG00000177125 | ZBTB34 |
| ILF3 | ENSG00000204947 | ZNF425 |
| ILF3 | ENSG00000104960 | PTOV1 |
| ILF3 | ENSG00000276753 | MIR6821 |
| ILF3 | ENSG00000171595 | DNAI2 |
| ILF3 | ENSG00000230751 | AC007036.4 |
| ILF3 | ENSG00000149231 | CCDC82 |
| ILF3 | ENSG00000167004 | PDIA3 |
| ILF3 | ENSG00000272677 | RP11-127B20.3 |
| ILF3 | ENSG00000092964 | DPYSL2 |
| ILF3 | ENSG00000167196 | FBXO22 |
| ILF3 | ENSG00000151014 | CCRN4L |
| ILF3 | ENSG00000252608 | RNU6-1191P |
| ILF3 | ENSG00000236234 | AC091132.1 |
| ILF3 | ENSG00000100418 | DESI1 |
| ILF3 | ENSG00000141540 | TTYH2 |
| ILF3 | ENSG00000226272 | ARHGAP26- |
| ILF3 | ENSG00000198863 | AS1  RUNDC1 |
| ILF3 | ENSG00000254860 | TMEM9B-AS1 |
| ILF3 | ENSG00000087008 | ACOX3 |
| ILF3 | ENSG00000207421 | SNORD38B |
| ILF3 | ENSG00000135482 | ZC3H10 |
| ILF3 | ENSG00000254992 | RP11-215H18.5 |
| ILF3 | ENSG00000171174 | RBKS |
| ILF3 | ENSG00000111319 | SCNN1A |
| ILF3 | ENSG00000138439 | FAM117B |
| ILF3 | ENSG00000232971 | RP11-293A10.3 |
| ILF3 | ENSG00000166598 | HSP90B1 |
| ILF3 | ENSG00000085063 | CD59 |
| ILF3 | ENSG00000116981 | NT5C1A |
| ILF3 | ENSG00000178567 | EPM2AIP1 |
| ILF3 | ENSG00000200334 | Y_RNA |
| ILF3 | ENSG00000166435 | XRRA1 |
| ILF3 | ENSG00000132478 | UNK |
| ILF3 | ENSG00000196227 | FAM217B |
| ILF3 | ENSG00000167657 | DAPK3 |
| ILF3 | ENSG00000105371 | ICAM4 |
| ILF3 | ENSG00000131507 | NDFIP1 |
| ILF3 | ENSG00000187097 | ENTPD5 |
| ILF3 | ENSG00000256222 | MTRNR2L3 |
| ILF3 | ENSG00000240634 | RP11-1348G14.1 |
| ILF3 | ENSG00000172262 | ZNF131 |
| ILF3 | ENSG00000072682 | P4HA2 |
| ILF3 | ENSG00000224596 | ZMIZ1-AS1 |
| ILF3 | ENSG00000174807 | CD248 |
| ILF3 | ENSG00000280299 | RP11-583F2.7 |
| ILF3 | ENSG00000268108 | CTB-60B18.12 |
| ILF3 | ENSG00000155100 | OTUD6B |

| ILF3 | ENSG00000259623 | RP11-156E6.1 |
| --- | --- | --- |
| ILF3 | ENSG00000122483 | CCDC18 |
| ILF3 | ENSG00000078269 | SYNJ2 |
| ILF3 | ENSG00000160075 | SSU72 |
| ILF3 | ENSG00000279840 | RP1-228P16.9 |
| ILF3 | ENSG00000206150 | RNASE13 |
| ILF3 | ENSG00000245910 | SNHG6 |
| ILF3 | ENSG00000133114 | GPALPP1 |
| ILF3 | ENSG00000227373 | RP11-160H22.5 |
| ILF3 | ENSG00000136943 | CTSV |
| ILF3 | ENSG00000127616 | SMARCA4 |
| ILF3 | ENSG00000159871 | LYPD5 |
| ILF3 | ENSG00000237531 | RP11-309M23.1 |
| ILF3 | ENSG00000066933 | MYO9A |
| ILF3 | ENSG00000206028 | CTA-373H7.7 |
| ILF3 | ENSG00000204922 | UQCC3 |
| ILF3 | ENSG00000187902 | SHISA7 |
| ILF3 | ENSG00000255587 | RAB44 |
| ILF3 | ENSG00000239988 | RPL31P60 |
| ILF3 | ENSG00000146587 | RBAK |
| ILF3 | ENSG00000123562 | MORF4L2 |
| ILF3 | ENSG00000167880 | EVPL |
| ILF3 | ENSG00000227198 | C6orf47-AS1 |
| ILF3 | ENSG00000252657 | SNORA70 |
| ILF3 | ENSG00000130024 | PHF10 |
| ILF3 | ENSG00000116350 | SRSF4 |
| ILF3 | ENSG00000268400 | CTD- |
| ILF3 | ENSG00000180061 | 3214H19.4 TMEM150B |
| ILF3 | ENSG00000272810 | U91328.22 |
| ILF3 | ENSG00000112041 | TULP1 |
| ILF3 | ENSG00000202400 | SNORD82 |
| ILF3 | ENSG00000198723 | C19orf45 |
| ILF3 | ENSG00000252143 | SCARNA17 |
| ILF3 | ENSG00000113575 | PPP2CA |
| ILF3 | ENSG00000090612 | ZNF268 |
| ILF3 | ENSG00000143452 | HORMAD1 |
| ILF3 | ENSG00000161888 | SPC24 |
| ILF3 | ENSG00000272707 | RP11-534C12.1 |
| ILF3 | ENSG00000234678 | RP11-465N4.4 |
| ILF3 | ENSG00000248489 | CTD- |
| ILF3 | ENSG00000176386 | 2007H13.3  CDC26 |
| ILF3 | ENSG00000260032 | LINC00657 |
| ILF3 | ENSG00000224600 | RP4-612B18.1 |
| ILF3 | ENSG00000265137 | MIR3192 |
| ILF3 | ENSG00000202515 | VTRNA1-3 |
| ILF3 | ENSG00000173145 | NOC3L |
| ILF3 | ENSG00000137936 | BCAR3 |
| ILF3 | ENSG00000174276 | ZNHIT2 |
| ILF3 | ENSG00000235415 | AC005808.3 |
| ILF3 | ENSG00000223475 | RP11-310H4.1 |

ILF3 ENSG00000225642 SNRPEP5

| ILF3 | ENSG00000255432 | RP11-831H9.11 |
| --- | --- | --- |
| ILF3 | ENSG00000159217 | IGF2BP1 |
| ILF3 | ENSG00000255581 | RP11-69M1.4 |
| ILF3 | ENSG00000226247 | SUPT4H1P |
| ILF3 | ENSG00000152234 | ATP5A1 |
| ILF3 | ENSG00000214347 | CTB-180A7.8 |
| ILF3 | ENSG00000157077 | ZFYVE9 |
| ILF3 | ENSG00000127993 | RBM48 |
| ILF3 | ENSG00000214612 | RPS19P1 |
| ILF3 | ENSG00000279861 | RP11-43N16.4 |
| ILF3 | ENSG00000278449 | MIR6892 |
| ILF3 | ENSG00000132744 | ACY3 |
| ILF3 | ENSG00000130332 | LSM7 |
| ILF3 | ENSG00000165030 | NFIL3 |
| ILF3 | ENSG00000243646 | IL10RB |
| ILF3 | ENSG00000202317 | RNU1-84P |
| ILF3 | ENSG00000229607 | RP5-1125N11.1 |
| ILF3 | ENSG00000187742 | SECISBP2 |
| ILF3 | ENSG00000185504 | C17orf70 |
| ILF3 | ENSG00000237686 | RP5-1120P11.1 |
| ILF3 | ENSG00000207381 | RNU6-950P |
| ILF3 | ENSG00000262869 | CTD-2545H1.1 |
| ILF3 | ENSG00000213553 | RPLP0P6 |
| ILF3 | ENSG00000224100 | AP001630.5 |
| ILF3 | ENSG00000250312 | ZNF718 |
| ILF3 | ENSG00000259172 | RP11-299G20.2 |
| ILF3 | ENSG00000256312 | RP13-977J11.8 |
| ILF3 | ENSG00000136295 | TTYH3 |
| ILF3 | ENSG00000166794 | PPIB |
| ILF3 | ENSG00000130703 | OSBPL2 |
| ILF3 | ENSG00000272748 | RP11-222G7.2 |
| ILF3 | ENSG00000132535 | DLG4 |
| ILF3 | ENSG00000106348 | IMPDH1 |
| ILF3 | ENSG00000258458 | CTD-2555K7.2 |
| ILF3 | ENSG00000225742 | RP11-513G11.4 |
| ILF3 | ENSG00000275213 | Metazoa_SRP |
| ILF3 | ENSG00000212146 | RNU6-910P |
| ILF3 | ENSG00000167740 | CYB5D2 |
| ILF3 | ENSG00000184939 | ZFP90 |
| ILF3 | ENSG00000258529 | ALG9 |
| ILF3 | ENSG00000124762 | CDKN1A |
| ILF3 | ENSG00000121481 | RNF2 |
| ILF3 | ENSG00000238650 | SNORD54 |
| ILF3 | ENSG00000267498 | CTB-32O4.2 |
| ILF3 | ENSG00000240518 | RP11-515C16.1 |
| ILF3 | ENSG00000224183 | SDHDP6 |
| ILF3 | ENSG00000117009 | KMO |
| ILF3 | ENSG00000279243 | CMB9-22P13.2 |
| ILF3 | ENSG00000015171 | ZMYND11 |
| ILF3 | ENSG00000198400 | NTRK1 |
| ILF3 | ENSG00000011523 | CEP68 |

| ILF3 | ENSG00000205593 | DENND6B |
| --- | --- | --- |
| ILF3 | ENSG00000104859 | CLASRP |
| ILF3 | ENSG00000276083 | MIR7976 |
| ILF3 | ENSG00000162062 | C16orf59 |
| ILF3 | ENSG00000275489 | C17orf98 |
| ILF3 | ENSG00000224848 | RP11-535M15.1 |
| ILF3 | ENSG00000276405 | TRBV13 |
| ILF3 | ENSG00000102119 | EMD |
| ILF3 | ENSG00000110619 | CARS |
| ILF3 | ENSG00000108932 | SLC16A6 |
| ILF3 | ENSG00000207382 | Y_RNA |
| ILF3 | ENSG00000232296 | RP11-510N19.2 |
| ILF3 | ENSG00000214176 | PLEKHM1P |
| ILF3 | ENSG00000207773 | MIR642A |
| ILF3 | ENSG00000247077 | PGAM5 |
| ILF3 | ENSG00000112855 | HARS2 |
| ILF3 | ENSG00000231507 | LINC01353 |
| ILF3 | ENSG00000213994 | RP11-414H17.5 |
| ILF3 | ENSG00000198211 | TUBB3 |
| ILF3 | ENSG00000244355 | LY6G6D |
| ILF3 | ENSG00000200653 | RNU4-92P |
| ILF3 | ENSG00000133935 | C14orf1 |
| ILF3 | ENSG00000261659 | LA16c-313D11.12 |
| ILF3 | ENSG00000232163 | RPLP1P13 |
| ILF3 | ENSG00000148300 | REXO4 |
| ILF3 | ENSG00000125812 | GZF1 |
| ILF3 | ENSG00000260442 | RP11-22P6.3 |
| ILF3 | ENSG00000217078 | RP1-13D10.3 |
| ILF3 | ENSG00000142102 | ATHL1 |
| ILF3 | ENSG00000101057 | MYBL2 |
| ILF3 | ENSG00000275025 | RP5-875H18.10 |
| ILF3 | ENSG00000186234 | FAM86MP |
| ILF3 | ENSG00000171862 | PTEN |
| ILF3 | ENSG00000042445 | RETSAT |
| ILF3 | ENSG00000204519 | ZNF551 |
| ILF3 | ENSG00000124787 | RPP40 |
| ILF3 | ENSG00000160917 | CPSF4 |
| ILF3 | ENSG00000201301 | RNA5SP130 |
| ILF3 | ENSG00000140675 | SLC5A2 |
| ILF3 | ENSG00000178977 | LINC00324 |
| ILF3 | ENSG00000252680 | RNA5SP449 |
| ILF3 | ENSG00000131503 | ANKHD1 |
| ILF3 | ENSG00000084092 | NOA1 |
| ILF3 | ENSG00000233554 | B4GALT1-AS1 |
| ILF3 | ENSG00000225196 | RP5-1118D24.2 |
| ILF3 | ENSG00000140332 | TLE3 |
| ILF3 | ENSG00000083454 | P2RX5 |
| ILF3 | ENSG00000154814 | OXNAD1 |
| ILF3 | ENSG00000229399 | RP11-378J18.6 |
| ILF3 | ENSG00000044446 | PHKA2 |
| ILF3 | ENSG00000204529 | GUCY2EP |

| ILF3 | ENSG00000168028 | RPSA |
| --- | --- | --- |
| ILF3 | ENSG00000129696 | TTI2 |
| ILF3 | ENSG00000203943 | SAMD13 |
| ILF3 | ENSG00000120784 | ZFP30 |
| ILF3 | ENSG00000159352 | PSMD4 |
| ILF3 | ENSG00000101017 | CD40 |
| ILF3 | ENSG00000207614 | MIR193A |
| ILF3 | ENSG00000252542 | SNORD36C |
| ILF3 | ENSG00000183624 | HMCES |
| ILF3 | ENSG00000167700 | MFSD3 |
| ILF3 | ENSG00000159377 | PSMB4 |
| ILF3 | ENSG00000202300 | RNU6-487P |
| ILF3 | ENSG00000170917 | NUDT6 |
| ILF3 | ENSG00000083544 | TDRD3 |
| ILF3 | ENSG00000237972 | TUBG1P |
| ILF3 | ENSG00000265396 | MIR3128 |
| ILF3 | ENSG00000139826 | ABHD13 |
| ILF3 | ENSG00000256771 | ZNF253 |
| ILF3 | ENSG00000266341 | RP5-890E16.4 |
| ILF3 | ENSG00000166507 | NDST2 |
| ILF3 | ENSG00000278276 | RP3-324O17.8 |
| ILF3 | ENSG00000196922 | ZNF252P |
| ILF3 | ENSG00000178498 | DTX3 |
| ILF3 | ENSG00000237341 | SYP-AS1 |
| ILF3 | ENSG00000260252 | RP11-384M15.3 |
| ILF3 | ENSG00000238704 | RNU7-97P |
| ILF3 | ENSG00000258798 | RP11-895M11.3 |
| ILF3 | ENSG00000268173 | PIK3R2 |
| ILF3 | ENSG00000252033 | RNU6-311P |
| ILF3 | ENSG00000172531 | PPP1CA |
| ILF3 | ENSG00000233614 | DDX11L10 |
| ILF3 | ENSG00000269318 | AC007292.3 |
| ILF3 | ENSG00000034053 | APBA2 |
| ILF3 | ENSG00000232456 | RP11-5P18.10 |
| ILF3 | ENSG00000274535 | SNORD39 |
| ILF3 | ENSG00000215914 | MMP23A |
| ILF3 | ENSG00000278002 | RP11-596C23.2 |
| ILF3 | ENSG00000116138 | DNAJC16 |
| ILF3 | ENSG00000230483 | AC124057.5 |
| ILF3 | ENSG00000279747 | CTA-363E6.8 |
| ILF3 | ENSG00000275703 | U47924.32 |
| ILF3 | ENSG00000008869 | HEATR5B |
| ILF3 | ENSG00000266202 | RP1-66C13.4 |
| ILF3 | ENSG00000204574 | ABCF1 |
| ILF3 | ENSG00000174917 | C19orf70 |
| ILF3 | ENSG00000188215 | DCUN1D3 |
| ILF3 | ENSG00000149679 | CABLES2 |
| ILF3 | ENSG00000207384 | Y_RNA |
| ILF3 | ENSG00000132563 | REEP2 |
| ILF3 | ENSG00000074219 | TEAD2 |
| ILF3 | ENSG00000165271 | NOL6 |

| ILF3 | ENSG00000240184 | PCDHGC3 |
| --- | --- | --- |
| ILF3 | ENSG00000232065 | LINC01063 |
| ILF3 | ENSG00000213160 | KLHL23 |
| ILF3 | ENSG00000168917 | SLC35G2 |
| ILF3 | ENSG00000152527 | PLEKHH2 |
| ILF3 | ENSG00000205129 | C4orf47 |
| ILF3 | ENSG00000138074 | SLC5A6 |
| ILF3 | ENSG00000124733 | MEA1 |
| ILF3 | ENSG00000254536 | RP11-108K14.8 |
| ILF3 | ENSG00000143622 | RIT1 |
| ILF3 | ENSG00000100083 | GGA1 |
| ILF3 | ENSG00000090857 | PDPR |
| ILF3 | ENSG00000255114 | RP11-110I1.6 |
| ILF3 | ENSG00000164181 | ELOVL7 |
| ILF3 | ENSG00000272391 | POM121C |
| ILF3 | ENSG00000259319 | RP11-293M10.6 |
| ILF3 | ENSG00000263641 | MIR4777 |
| ILF3 | ENSG00000117697 | NSL1 |
| ILF3 | ENSG00000228292 | RP11-220I1.4 |
| ILF3 | ENSG00000275871 | RP11-394O4.6 |
| ILF3 | ENSG00000104412 | EMC2 |
| ILF3 | ENSG00000222432 | Y_RNA |
| ILF3 | ENSG00000169992 | NLGN2 |
| ILF3 | ENSG00000259917 | HNRNPLP2 |
| ILF3 | ENSG00000105640 | RPL18A |
| ILF3 | ENSG00000175348 | TMEM9B |
| ILF3 | ENSG00000131398 | KCNC3 |
| ILF3 | ENSG00000066557 | LRRC40 |
| ILF3 | ENSG00000235143 | RP1-65J11.5 |
| ILF3 | ENSG00000213145 | CRIP1 |
| ILF3 | ENSG00000109929 | SC5D |
| ILF3 | ENSG00000238288 | RP11-100G15.4 |
| ILF3 | ENSG00000163576 | EFHB |
| ILF3 | ENSG00000166073 | GPR176 |
| ILF3 | ENSG00000213793 | ZNF888 |
| ILF3 | ENSG00000108604 | SMARCD2 |
| ILF3 | ENSG00000160961 | ZNF333 |
| ILF3 | ENSG00000260290 | CTD- |
| ILF3 | ENSG00000171681 | 2033A16.2 ATF7IP |
| ILF3 | ENSG00000260088 | RP11-92G12.3 |
| ILF3 | ENSG00000061938 | TNK2 |
| ILF3 | ENSG00000117602 | RCAN3 |
| ILF3 | ENSG00000188958 | UTS2B |
| ILF3 | ENSG00000259520 | CTD- |
| ILF3 | ENSG00000172954 | 2651B20.3 LCLAT1 |
| ILF3 | ENSG00000212864 | RNF208 |
| ILF3 | ENSG00000185946 | RNPC3 |
| ILF3 | ENSG00000106526 | ACTR3C |
| ILF3 | ENSG00000278356 | RP11-372B4.3 |
| ILF3 | ENSG00000261281 | RP11-361M10.4 |

ILF3 ENSG00000185513 L3MBTL1

| ILF3 | ENSG00000169016 | E2F6 |
| --- | --- | --- |
| ILF3 | ENSG00000137824 | RMDN3 |
| ILF3 | ENSG00000084710 | EFR3B |
| ILF3 | ENSG00000187741 | FANCA |
| ILF3 | ENSG00000214553 | LRRC37A11P |
| ILF3 | ENSG00000133597 | ADCK2 |
| ILF3 | ENSG00000140326 | CDAN1 |
| ILF3 | ENSG00000277136 | Metazoa_SRP |
| ILF3 | ENSG00000128789 | PSMG2 |
| ILF3 | ENSG00000206659 | Y_RNA |
| ILF3 | ENSG00000222213 | RNU6-588P |
| ILF3 | ENSG00000166130 | IKBIP |
| ILF3 | ENSG00000279070 | RP11-287D1.2 |
| ILF3 | ENSG00000269181 | CTD- |
| ILF3 | ENSG00000251085 | 3073N11.5  RP11-893F2.6 |
| ILF3 | ENSG00000174898 | CATSPERD |
| ILF3 | ENSG00000214367 | HAUS3 |
| ILF3 | ENSG00000135404 | CD63 |
| ILF3 | ENSG00000171960 | PPIH |
| ILF3 | ENSG00000265091 | RP11-835E18.5 |
| ILF3 | ENSG00000229589 | ACVR2B-AS1 |
| ILF3 | ENSG00000143248 | RGS5 |
| ILF3 | ENSG00000233461 | RP11-295G20.2 |
| ILF3 | ENSG00000010256 | UQCRC1 |
| ILF3 | ENSG00000142528 | ZNF473 |
| ILF3 | ENSG00000080200 | CRYBG3 |
| ILF3 | ENSG00000167105 | TMEM92 |
| ILF3 | ENSG00000262633 | RP11-156P1.2 |
| ILF3 | ENSG00000266938 | AC119403.1 |
| ILF3 | ENSG00000278922 | AC002310.14 |
| ILF3 | ENSG00000165805 | C12orf50 |
| ILF3 | ENSG00000178636 | RP11-455G16.1 |
| ILF3 | ENSG00000233078 | RP11-5P18.5 |
| ILF3 | ENSG00000272572 | RP11-179B2.2 |
| ILF3 | ENSG00000173581 | CCDC106 |
| ILF3 | ENSG00000149506 | ZP1 |
| ILF3 | ENSG00000151151 | IPMK |
| ILF3 | ENSG00000274515 | CTD- |
| ILF3 | ENSG00000178053 | 2026K11.5  MLF1 |
| ILF3 | ENSG00000271366 | AC002128.5 |
| ILF3 | ENSG00000105373 | GLTSCR2 |
| ILF3 | ENSG00000163431 | LMOD1 |
| ILF3 | ENSG00000279261 | PP2672 |
| ILF3 | ENSG00000278338 | VWA8-AS1 |
| ILF3 | ENSG00000260452 | TPRKBP2 |
| ILF3 | ENSG00000239419 | RN7SL535P |
| ILF3 | ENSG00000088205 | DDX18 |
| ILF3 | ENSG00000261839 | RP1-265C24.8 |
| ILF3 | ENSG00000126562 | WNK4 |
| ILF3 | ENSG00000144827 | ABHD10 |

ILF3 ENSG00000181544 FANCB

| ILF3 | ENSG00000169249 | ZRSR2 |
| --- | --- | --- |
| ILF3 | ENSG00000186510 | CLCNKA |
| ILF3 | ENSG00000074842 | C19orf10 |
| ILF3 | ENSG00000239203 | AC093484.4 |
| ILF3 | ENSG00000167074 | TEF |
| ILF3 | ENSG00000132604 | TERF2 |
| ILF3 | ENSG00000234005 | GAPDHP22 |
| ILF3 | ENSG00000243230 | RP11-286H14.8 |
| ILF3 | ENSG00000125779 | PANK2 |
| ILF3 | ENSG00000204160 | ZDHHC18 |
| ILF3 | ENSG00000177464 | GPR4 |
| ILF3 | ENSG00000236778 | INTS6-AS1 |
| ILF3 | ENSG00000100815 | TRIP11 |
| ILF3 | ENSG00000005844 | ITGAL |
| ILF3 | ENSG00000164219 | PGGT1B |
| ILF3 | ENSG00000232671 | RP11-126K1.2 |
| ILF3 | ENSG00000143761 | ARF1 |
| ILF3 | ENSG00000280233 | RP11-159D12.3 |
| ILF3 | ENSG00000277494 | GPIHBP1 |
| ILF3 | ENSG00000179935 | LINC00652 |
| ILF3 | ENSG00000264857 | MIR5696 |
| ILF3 | ENSG00000116857 | TMEM9 |
| ILF3 | ENSG00000068120 | COASY |
| ILF3 | ENSG00000100359 | SGSM3 |
| ILF3 | ENSG00000144040 | SFXN5 |
| ILF3 | ENSG00000267080 | ASB16-AS1 |
| ILF3 | ENSG00000200975 | RNU1-7P |
| ILF3 | ENSG00000106624 | AEBP1 |
| ILF3 | ENSG00000215467 | RPL27AP |
| ILF3 | ENSG00000154518 | ATP5G3 |
| ILF3 | ENSG00000146414 | SHPRH |
| ILF3 | ENSG00000185344 | ATP6V0A2 |
| ILF3 | ENSG00000255538 | OR10V2P |
| ILF3 | ENSG00000159423 | ALDH4A1 |
| ILF3 | ENSG00000252915 | Y_RNA |
| ILF3 | ENSG00000272031 | ANKRD34A |
| ILF3 | ENSG00000104941 | RSPH6A |
| ILF3 | ENSG00000114956 | DGUOK |
| ILF3 | ENSG00000135097 | MSI1 |
| ILF3 | ENSG00000169169 | CPT1C |
| ILF3 | ENSG00000258512 | LINC00239 |
| ILF3 | ENSG00000164187 | LMBRD2 |
| ILF3 | ENSG00000266963 | AC005625.1 |
| ILF3 | ENSG00000136861 | CDK5RAP2 |
| ILF3 | ENSG00000135045 | C9orf40 |
| ILF3 | ENSG00000229386 | OR8B9P |
| ILF3 | ENSG00000178913 | TAF7 |
| ILF3 | ENSG00000163541 | SUCLG1 |
| ILF3 | ENSG00000259444 | RP11-736N17.8 |
| ILF3 | ENSG00000140939 | NOL3 |
| ILF3 | ENSG00000169696 | ASPSCR1 |

| ILF3 | ENSG00000161036 | LRWD1 |
| --- | --- | --- |
| ILF3 | ENSG00000160588 | MPZL3 |
| ILF3 | ENSG00000164818 | HEATR2 |
| ILF3 | ENSG00000130513 | GDF15 |
| ILF3 | ENSG00000124486 | USP9X |
| ILF3 | ENSG00000199705 | Y_RNA |
| ILF3 | ENSG00000157837 | SPPL3 |
| ILF3 | ENSG00000274712 | RP11-147L13.15 |
| ILF3 | ENSG00000104147 | OIP5 |
| ILF3 | ENSG00000204520 | MICA |
| ILF3 | ENSG00000261663 | RP11-304L19.11 |
| ILF3 | ENSG00000090989 | EXOC1 |
| ILF3 | ENSG00000265683 | RP11-173M1.5 |
| ILF3 | ENSG00000270808 | RP11-574K11.27 |
| ILF3 | ENSG00000183309 | ZNF623 |
| ILF3 | ENSG00000177854 | TMEM187 |
| ILF3 | ENSG00000168152 | THAP9 |
| ILF3 | ENSG00000223203 | RNA5SP221 |
| ILF3 | ENSG00000178338 | ZNF354B |
| ILF3 | ENSG00000089639 | GMIP |
| ILF3 | ENSG00000114021 | NIT2 |
| ILF3 | ENSG00000235488 | JARID2-AS1 |
| ILF3 | ENSG00000166439 | RNF169 |
| ILF3 | ENSG00000228414 | LINC01185 |
| ILF3 | ENSG00000105401 | CDC37 |
| ILF3 | ENSG00000122484 | RPAP2 |
| ILF3 | ENSG00000160714 | UBE2Q1 |
| ILF3 | ENSG00000273145 | CITF22-92A6.1 |
| ILF3 | ENSG00000105668 | UPK1A |
| ILF3 | ENSG00000182446 | NPLOC4 |
| ILF3 | ENSG00000005175 | RPAP3 |
| ILF3 | ENSG00000267062 | CTD- |
| ILF3 | ENSG00000135341 | 2659N19.10  MAP3K7 |
| ILF3 | ENSG00000278549 | MIR6736 |
| ILF3 | ENSG00000139437 | TCHP |
| ILF3 | ENSG00000168807 | SNTB2 |
| ILF3 | ENSG00000249216 | RP11-227F19.5 |
| ILF3 | ENSG00000269371 | CTD- |
| ILF3 | ENSG00000161921 | 2207O23.11 CXCL16 |
| ILF3 | ENSG00000159625 | DRC7 |
| ILF3 | ENSG00000161896 | IP6K3 |
| ILF3 | ENSG00000171649 | ZIK1 |
| ILF3 | ENSG00000249628 | LINC00942 |
| ILF3 | ENSG00000249087 | ZNF436-AS1 |
| ILF3 | ENSG00000182938 | OTOP3 |
| ILF3 | ENSG00000261578 | RP11-21L23.2 |
| ILF3 | ENSG00000258462 | RP11-831F12.4 |
| ILF3 | ENSG00000088756 | ARHGAP28 |
| ILF3 | ENSG00000035687 | ADSS |
| ILF3 | ENSG00000151247 | EIF4E |

ILF3 ENSG00000276281 Metazoa_SRP

| ILF3 | ENSG00000200816 | SNORA38 |
| --- | --- | --- |
| ILF3 | ENSG00000108559 | NUP88 |
| ILF3 | ENSG00000271821 | XXbac- |
| ILF3 | ENSG00000235168 | BPG299F13.14 RP1-12G14.5 |
| ILF3 | ENSG00000127511 | SIN3B |
| ILF3 | ENSG00000115042 | FAHD2A |
| ILF3 | ENSG00000166317 | SYNPO2L |
| ILF3 | ENSG00000103423 | DNAJA3 |
| ILF3 | ENSG00000183527 | PSMG1 |
| ILF3 | ENSG00000233223 | AC113189.5 |
| ILF3 | ENSG00000248322 | RP4-559A3.6 |
| ILF3 | ENSG00000128534 | LSM8 |
| ILF3 | ENSG00000125843 | AP5S1 |
| ILF3 | ENSG00000249673 | NOP14-AS1 |
| ILF3 | ENSG00000152240 | HAUS1 |
| ILF3 | ENSG00000177683 | THAP5 |
| ILF3 | ENSG00000115053 | NCL |
| ILF3 | ENSG00000172500 | FIBP |
| ILF3 | ENSG00000048828 | FAM120A |
| ILF3 | ENSG00000051596 | THOC3 |
| ILF3 | ENSG00000139714 | MORN3 |
| ILF3 | ENSG00000185803 | SLC52A2 |
| ILF3 | ENSG00000234636 | MED14OS |
| ILF3 | ENSG00000223138 | RNA5SP450 |
| ILF3 | ENSG00000111325 | OGFOD2 |
| ILF3 | ENSG00000213281 | NRAS |
| ILF3 | ENSG00000138095 | LRPPRC |
| ILF3 | ENSG00000237330 | RNF223 |
| ILF3 | ENSG00000201135 | RNU6-777P |
| ILF3 | ENSG00000172239 | PAIP1 |
| ILF3 | ENSG00000252919 | Y_RNA |
| ILF3 | ENSG00000252628 | RNU6-453P |
| ILF3 | ENSG00000271889 | RP11-493E12.1 |
| ILF3 | ENSG00000127481 | UBR4 |
| ILF3 | ENSG00000273437 | RP11-434H6.7 |
| ILF3 | ENSG00000245385 | RP11-334E6.10 |
| ILF3 | ENSG00000237566 | AC090955.5 |
| ILF3 | ENSG00000136867 | SLC31A2 |
| ILF3 | ENSG00000132541 | HRSP12 |
| ILF3 | ENSG00000174327 | SLC16A13 |
| ILF3 | ENSG00000129214 | SHBG |
| ILF3 | ENSG00000151474 | FRMD4A |
| ILF3 | ENSG00000053372 | MRTO4 |
| ILF3 | ENSG00000272874 | RP5-1103G7.10 |
| ILF3 | ENSG00000234638 | AC053503.6 |
| ILF3 | ENSG00000200278 | RNA5SP352 |
| ILF3 | ENSG00000198130 | HIBCH |
| ILF3 | ENSG00000165724 | ZMYND19 |
| ILF3 | ENSG00000078124 | ACER3 |
| ILF3 | ENSG00000160741 | CRTC2 |
| ILF3 | ENSG00000068976 | PYGM |

| ILF3 | ENSG00000260589 | STAM-AS1 |
| --- | --- | --- |
| ILF3 | ENSG00000125434 | SLC25A35 |
| ILF3 | ENSG00000183647 | ZNF530 |
| ILF3 | ENSG00000107331 | ABCA2 |
| ILF3 | ENSG00000278359 | MIR7155 |
| ILF3 | ENSG00000224001 | RP11-270F18.2 |
| ILF3 | ENSG00000196071 | OR2L13 |
| ILF3 | ENSG00000135605 | TEC |
| ILF3 | ENSG00000277784 | MIR6786 |
| ILF3 | ENSG00000169515 | CCDC8 |
| ILF3 | ENSG00000230310 | CTD-2192J16.11 |
| ILF3 | ENSG00000254978 | ALG1L9P |
| ILF3 | ENSG00000001629 | ANKIB1 |
| ILF3 | ENSG00000092531 | SNAP23 |
| ILF3 | ENSG00000012779 | ALOX5 |
| ILF3 | ENSG00000197323 | TRIM33 |
| ILF3 | ENSG00000259399 | TGIF2-C20orf24 |
| ILF3 | ENSG00000064199 | SPA17 |
| ILF3 | ENSG00000034713 | GABARAPL2 |
| ILF3 | ENSG00000227875 | RP11-426M1.2 |
| ILF3 | ENSG00000185480 | PARPBP |
| ILF3 | ENSG00000146215 | CRIP3 |
| ILF3 | ENSG00000272656 | RP11-219D15.3 |
| ILF3 | ENSG00000162929 | KIAA1841 |
| ILF3 | ENSG00000161513 | FDXR |
| ILF3 | ENSG00000126804 | ZBTB1 |
| ILF3 | ENSG00000269970 | RP11-498E2.9 |
| ILF3 | ENSG00000164190 | NIPBL |
| ILF3 | ENSG00000247137 | RP11-727A23.5 |
| ILF3 | ENSG00000114107 | CEP70 |
| ILF3 | ENSG00000144357 | UBR3 |
| ILF3 | ENSG00000090520 | DNAJB11 |
| ILF3 | ENSG00000168096 | ANKS3 |
| ILF3 | ENSG00000149646 | CNBD2 |
| ILF3 | ENSG00000201850 | Y_RNA |
| ILF3 | ENSG00000185238 | PRMT3 |
| ILF3 | ENSG00000092140 | G2E3 |
| ILF3 | ENSG00000226900 | RP11-432J24.5 |
| ILF3 | ENSG00000034510 | TMSB10 |
| ILF3 | ENSG00000157782 | CABP1 |
| ILF3 | ENSG00000270324 | RP11-180P8.4 |
| ILF3 | ENSG00000171136 | RLN3 |
| ILF3 | ENSG00000197020 | ZNF100 |
| ILF3 | ENSG00000088888 | MAVS |
| ILF3 | ENSG00000267192 | AC006116.12 |
| ILF3 | ENSG00000166436 | TRIM66 |
| ILF3 | ENSG00000101945 | SUV39H1 |
| ILF3 | ENSG00000181381 | DDX60L |
| ILF3 | ENSG00000228702 | RP11-671E7.1 |
| ILF3 | ENSG00000275417 | RP11-844G16.3 |
| ILF3 | ENSG00000125319 | C17orf53 |

| ILF3 | ENSG00000099365 | STX1B |
| --- | --- | --- |
| ILF3 | ENSG00000178726 | THBD |
| ILF3 | ENSG00000206203 | TSSK2 |
| ILF3 | ENSG00000160199 | PKNOX1 |
| ILF3 | ENSG00000120727 | PAIP2 |
| ILF3 | ENSG00000272192 | CTD-2532N20.1 |
| ILF3 | ENSG00000222011 | FAM185A |
| ILF3 | ENSG00000163882 | POLR2H |
| ILF3 | ENSG00000263176 | RP11-893F2.15 |
| ILF3 | ENSG00000167987 | VPS37C |
| ILF3 | ENSG00000200170 | Y_RNA |
| ILF3 | ENSG00000224346 | AC005037.1 |
| ILF3 | ENSG00000162746 | FCRLB |
| ILF3 | ENSG00000073111 | MCM2 |
| ILF3 | ENSG00000269176 | RP11-727F15.12 |
| ILF3 | ENSG00000202008 | Y_RNA |
| ILF3 | ENSG00000119514 | GALNT12 |
| ILF3 | ENSG00000179889 | PDXDC1 |
| ILF3 | ENSG00000239671 | CTD-3193K9.1 |
| ILF3 | ENSG00000122873 | CISD1 |
| ILF3 | ENSG00000260031 | RPL10P14 |
| ILF3 | ENSG00000129810 | SGOL1 |
| ILF3 | ENSG00000143376 | SNX27 |
| ILF3 | ENSG00000207944 | MIR574 |
| ILF3 | ENSG00000261151 | RP11-332G1.1 |
| ILF3 | ENSG00000230415 | RP5-902P8.10 |
| ILF3 | ENSG00000158006 | PAFAH2 |
| ILF3 | ENSG00000163510 | CWC22 |
| ILF3 | ENSG00000273262 | RP11-18I14.11 |
| ILF3 | ENSG00000205629 | LCMT1 |
| ILF3 | ENSG00000196437 | ZNF569 |
| ILF3 | ENSG00000204421 | LY6G6C |
| ILF3 | ENSG00000184967 | NOC4L |
| ILF3 | ENSG00000046651 | OFD1 |
| ILF3 | ENSG00000160767 | FAM189B |
| ILF3 | ENSG00000250286 | RP11-94C24.8 |
| ILF3 | ENSG00000101255 | TRIB3 |
| ILF3 | ENSG00000175048 | ZDHHC14 |
| ILF3 | ENSG00000120910 | PPP3CC |
| ILF3 | ENSG00000149269 | PAK1 |
| ILF3 | ENSG00000257878 | RP11-256L6.3 |
| ILF3 | ENSG00000177098 | SCN4B |
| ILF3 | ENSG00000272562 | RP11-396C23.4 |
| ILF3 | ENSG00000265331 | MIR4524B |
| ILF3 | ENSG00000128595 | CALU |
| ILF3 | ENSG00000111371 | SLC38A1 |
| ILF3 | ENSG00000240589 | RN7SL258P |
| ILF3 | ENSG00000173915 | USMG5 |
| ILF3 | ENSG00000207009 | Y_RNA |
| ILF3 | ENSG00000188060 | RAB42 |
| ILF3 | ENSG00000151229 | SLC2A13 |

| ILF3 | ENSG00000214252 | AZGP1P2 |
| --- | --- | --- |
| ILF3 | ENSG00000146530 | VWDE |
| ILF3 | ENSG00000106785 | TRIM14 |
| ILF3 | ENSG00000170270 | C14orf142 |
| ILF3 | ENSG00000265176 | MIR3202-1 |
| ILF3 | ENSG00000265033 | RN7SL262P |
| ILF3 | ENSG00000268744 | CTD- |
| ILF3 | ENSG00000022976 | 3105H18.14 ZNF839 |
| ILF3 | ENSG00000259316 | CTD-2116N17.1 |
| ILF3 | ENSG00000255737 | AGAP2-AS1 |
| ILF3 | ENSG00000254595 | CTD-2010I16.1 |
| ILF3 | ENSG00000100532 | CGRRF1 |
| ILF3 | ENSG00000204406 | MBD5 |
| ILF3 | ENSG00000089876 | DHX32 |
| ILF3 | ENSG00000076356 | PLXNA2 |
| ILF3 | ENSG00000200579 | Y_RNA |
| ILF3 | ENSG00000006837 | CDKL3 |
| ILF3 | ENSG00000235770 | LINC00607 |
| ILF3 | ENSG00000201151 | SNORD56 |
| ILF3 | ENSG00000141934 | PPAP2C |
| ILF3 | ENSG00000236540 | AC006547.13 |
| ILF3 | ENSG00000132613 | MTSS1L |
| ILF3 | ENSG00000234500 | GS1-124K5.10 |
| ILF3 | ENSG00000114859 | CLCN2 |
| ILF3 | ENSG00000100490 | CDKL1 |
| ILF3 | ENSG00000249526 | CTB-35F21.1 |
| ILF3 | ENSG00000196290 | NIF3L1 |
| ILF3 | ENSG00000164024 | METAP1 |
| ILF3 | ENSG00000238516 | Y_RNA |
| ILF3 | ENSG00000263620 | RP11-599B13.6 |
| ILF3 | ENSG00000163947 | ARHGEF3 |
| ILF3 | ENSG00000179818 | PCBP1-AS1 |
| ILF3 | ENSG00000270526 | SNRPGP19 |
| ILF3 | ENSG00000201793 | RN7SKP9 |
| ILF3 | ENSG00000149532 | CPSF7 |
| ILF3 | ENSG00000230698 | CTD-2330K9.2 |
| ILF3 | ENSG00000172366 | FAM195A |
| ILF3 | ENSG00000176182 | MYPOP |
| ILF3 | ENSG00000274290 | HIST1H2BE |
| ILF3 | ENSG00000108395 | TRIM37 |
| ILF3 | ENSG00000112679 | DUSP22 |
| ILF3 | ENSG00000276026 | RP4-545L17.11 |
| ILF3 | ENSG00000180530 | NRIP1 |
| ILF3 | ENSG00000148926 | ADM |
| ILF3 | ENSG00000267755 | AC004623.2 |
| ILF3 | ENSG00000258316 | KLF17P1 |
| ILF3 | ENSG00000260815 | RP11-24M17.3 |
| ILF3 | ENSG00000139610 | CELA1 |
| ILF3 | ENSG00000198517 | MAFK |
| ILF3 | ENSG00000110400 | PVRL1 |
| ILF3 | ENSG00000174915 | PTDSS2 |

| ILF3 | ENSG00000105135 | ILVBL |
| --- | --- | --- |
| ILF3 | ENSG00000212163 | SNORD91A |
| ILF3 | ENSG00000011260 | UTP18 |
| ILF3 | ENSG00000143476 | DTL |
| ILF3 | ENSG00000235058 | ZMYND10-AS1 |
| ILF3 | ENSG00000200169 | RNU5D-1 |
| ILF3 | ENSG00000225648 | SBDSP1 |
| ILF3 | ENSG00000188342 | GTF2F2 |
| ILF3 | ENSG00000270123 | VTRNA2-1 |
| ILF3 | ENSG00000216101 | MIR877 |
| ILF3 | ENSG00000143499 | SMYD2 |
| ILF3 | ENSG00000196636 | ACN9 |
| ILF3 | ENSG00000276075 | CTD-2012K14.8 |
| ILF3 | ENSG00000240522 | RPL7AP10 |
| ILF3 | ENSG00000275234 | CTD-2396E7.11 |
| ILF3 | ENSG00000120055 | C10orf95 |
| ILF3 | ENSG00000059758 | CDK17 |
| ILF3 | ENSG00000199461 | Y_RNA |
| ILF3 | ENSG00000084652 | TXLNA |
| ILF3 | ENSG00000218690 | HIST1H2APS4 |
| ILF3 | ENSG00000188368 | PRR19 |
| ILF3 | ENSG00000274603 | Y_RNA |
| ILF3 | ENSG00000275041 | Metazoa_SRP |
| ILF3 | ENSG00000272993 | RP11-196G18.24 |
| ILF3 | ENSG00000272719 | CTB-161C1.1 |
| ILF3 | ENSG00000171777 | RASGRP4 |
| ILF3 | ENSG00000166823 | MESP1 |
| ILF3 | ENSG00000203573 | Metazoa_SRP |
| ILF3 | ENSG00000110104 | CCDC86 |
| ILF3 | ENSG00000280173 | RP11-447D11.3 |
| ILF3 | ENSG00000255472 | RP11-998D10.1 |
| ILF3 | ENSG00000007402 | CACNA2D2 |
| ILF3 | ENSG00000125730 | C3 |
| ILF3 | ENSG00000173230 | GOLGB1 |
| ILF3 | ENSG00000104899 | AMH |
| ILF3 | ENSG00000227372 | TP73-AS1 |
| ILF3 | ENSG00000110107 | PRPF19 |
| ILF3 | ENSG00000234062 | RP11-308D16.2 |
| ILF3 | ENSG00000100092 | SH3BP1 |
| ILF3 | ENSG00000185515 | BRCC3 |
| ILF3 | ENSG00000056050 | C4orf27 |
| ILF3 | ENSG00000261434 | CTD-2083E4.7 |
| ILF3 | ENSG00000149503 | INCENP |
| ILF3 | ENSG00000269946 | RP11-2B6.3 |
| ILF3 | ENSG00000164850 | GPER1 |
| ILF3 | ENSG00000075303 | SLC25A40 |
| ILF3 | ENSG00000199598 | RNU6-859P |
| ILF3 | ENSG00000278931 | bP-2189O9.2 |
| ILF3 | ENSG00000236021 | RP11-195C7.1 |
| ILF3 | ENSG00000176390 | CRLF3 |
| ILF3 | ENSG00000160113 | NR2F6 |

| ILF3 | ENSG00000167470 | MIDN |
| --- | --- | --- |
| ILF3 | ENSG00000258413 | RP11-665C16.6 |
| ILF3 | ENSG00000259468 | RP11-1084A12.2 |
| ILF3 | ENSG00000108091 | CCDC6 |
| ILF3 | ENSG00000276390 | RP1-197B17.5 |
| ILF3 | ENSG00000185627 | PSMD13 |
| ILF3 | ENSG00000162068 | NTN3 |
| ILF3 | ENSG00000110841 | PPFIBP1 |
| ILF3 | ENSG00000185453 | C19orf68 |
| ILF3 | ENSG00000149089 | APIP |
| ILF3 | ENSG00000070756 | PABPC1 |
| ILF3 | ENSG00000198917 | C9orf114 |
| ILF3 | ENSG00000132510 | KDM6B |
| ILF3 | ENSG00000241343 | RPL36A |
| ILF3 | ENSG00000248643 | RBM14-RBM4 |
| ILF3 | ENSG00000182944 | EWSR1 |
| ILF3 | ENSG00000235863 | B3GALT4 |
| ILF3 | ENSG00000201415 | RNA5SP204 |
| ILF3 | ENSG00000189306 | RRP7A |
| ILF3 | ENSG00000184903 | IMMP2L |
| ILF3 | ENSG00000272655 | POLR2J4 |
| ILF3 | ENSG00000035720 | STAP1 |
| ILF3 | ENSG00000148516 | ZEB1 |
| ILF3 | ENSG00000262251 | RP11-199F11.2 |
| ILF3 | ENSG00000107872 | FBXL15 |
| ILF3 | ENSG00000267522 | CTD-2621I17.6 |
| ILF3 | ENSG00000232245 | RP4-705F19.1 |
| ILF3 | ENSG00000087301 | TXNDC16 |
| ILF3 | ENSG00000186153 | WWOX |
| ILF3 | ENSG00000205500 | AC013472.3 |
| ILF3 | ENSG00000228037 | RP3-395M20.9 |
| ILF3 | ENSG00000165568 | AKR1E2 |
| ILF3 | ENSG00000205423 | CNEP1R1 |
| ILF3 | ENSG00000273365 | RP11-466F5.10 |
| ILF3 | ENSG00000241429 | EEF1A1P25 |
| ILF3 | ENSG00000254370 | RP11-181B11.1 |
| ILF3 | ENSG00000105708 | ZNF14 |
| ILF3 | ENSG00000171988 | JMJD1C |
| ILF3 | ENSG00000108312 | UBTF |
| ILF3 | ENSG00000222363 | RNU4-34P |
| ILF3 | ENSG00000136936 | XPA |
| ILF3 | ENSG00000197061 | HIST1H4C |
| ILF3 | ENSG00000177191 | B3GNT8 |
| ILF3 | ENSG00000235432 | RP5-930J4.5 |
| ILF3 | ENSG00000172215 | CXCR6 |
| ILF3 | ENSG00000137275 | RIPK1 |
| ILF3 | ENSG00000141096 | DPEP3 |
| ILF3 | ENSG00000020256 | ZFP64 |
| ILF3 | ENSG00000156928 | MALSU1 |
| ILF3 | ENSG00000255513 | AC005363.9 |
| ILF3 | ENSG00000266903 | CTB-171A8.1 |

| ILF3 | ENSG00000227619 | RP11-492E3.2 |
| --- | --- | --- |
| ILF3 | ENSG00000147475 | ERLIN2 |
| ILF3 | ENSG00000100347 | SAMM50 |
| ILF3 | ENSG00000173567 | GPR113 |
| ILF3 | ENSG00000130377 | ACSBG2 |
| ILF3 | ENSG00000241258 | CRCP |
| ILF3 | ENSG00000162869 | PPP1R21 |
| ILF3 | ENSG00000117318 | ID3 |
| ILF3 | ENSG00000277147 | LINC00869 |
| ILF3 | ENSG00000174903 | RAB1B |
| ILF3 | ENSG00000274752 | TRBV12-3 |
| ILF3 | ENSG00000268949 | MRPS17P1 |
| ILF3 | ENSG00000205534 | SMG1P2 |
| ILF3 | ENSG00000272153 | RP1-286D6.5 |
| ILF3 | ENSG00000196782 | MAML3 |
| ILF3 | ENSG00000170545 | SMAGP |
| ILF3 | ENSG00000252892 | RNU6-548P |
| ILF3 | ENSG00000225938 | RP4-575N6.4 |
| ILF3 | ENSG00000240137 | ERICH6-AS1 |
| ILF3 | ENSG00000229338 | RP11-92C4.4 |
| ILF3 | ENSG00000131467 | PSME3 |
| ILF3 | ENSG00000102904 | TSNAXIP1 |
| ILF3 | ENSG00000259112 | NDUFC2- |
| ILF3 | ENSG00000184060 | KCTD14 ADAP2 |
| ILF3 | ENSG00000112977 | DAP |
| ILF3 | ENSG00000168026 | TTC21A |
| ILF3 | ENSG00000080815 | PSEN1 |
| ILF3 | ENSG00000198728 | LDB1 |
| ILF3 | ENSG00000198346 | ZNF813 |
| ILF3 | ENSG00000233080 | LINC01399 |
| ILF3 | ENSG00000105738 | SIPA1L3 |
| ILF3 | ENSG00000206620 | SNORD45C |
| ILF3 | ENSG00000258045 | Metazoa_SRP |
| ILF3 | ENSG00000105854 | PON2 |
| ILF3 | ENSG00000270259 | RP13-122B23.9 |
| ILF3 | ENSG00000273071 | RP11-337C18.10 |
| ILF3 | ENSG00000162783 | IER5 |
| ILF3 | ENSG00000165891 | E2F7 |
| ILF3 | ENSG00000198515 | CNGA1 |
| ILF3 | ENSG00000200309 | Y_RNA |
| ILF3 | ENSG00000225573 | RPL35P5 |
| ILF3 | ENSG00000220583 | RPL35P2 |
| ILF3 | ENSG00000269881 | AC004754.3 |
| ILF3 | ENSG00000165272 | AQP3 |
| ILF3 | ENSG00000271849 | CTC-332L22.1 |
| ILF3 | ENSG00000161133 | USP41 |
| ILF3 | ENSG00000235319 | AC012360.4 |
| ILF3 | ENSG00000260727 | SLC7A5P1 |
| ILF3 | ENSG00000204366 | ZBTB12 |
| ILF3 | ENSG00000101421 | CHMP4B |
| ILF3 | ENSG00000023697 | DERA |

| ILF3 | ENSG00000271677 | RP1-274L7.3 |
| --- | --- | --- |
| ILF3 | ENSG00000109118 | PHF12 |
| ILF3 | ENSG00000196072 | BLOC1S2 |
| ILF3 | ENSG00000109381 | ELF2 |
| ILF3 | ENSG00000152292 | SH2D6 |
| ILF3 | ENSG00000109458 | GAB1 |
| ILF3 | ENSG00000162551 | ALPL |
| ILF3 | ENSG00000219863 | RPL31P28 |
| ILF3 | ENSG00000099860 | GADD45B |
| ILF3 | ENSG00000166855 | CLPX |
| ILF3 | ENSG00000239742 | RN7SL672P |
| ILF3 | ENSG00000174989 | FBXW8 |
| ILF3 | ENSG00000148343 | FAM73B |
| ILF3 | ENSG00000188603 | CLN3 |
| ILF3 | ENSG00000166938 | DIS3L |
| ILF3 | ENSG00000136560 | TANK |
| ILF3 | ENSG00000260281 | RP11-329J18.2 |
| ILF3 | ENSG00000214875 | MED28P1 |
| ILF3 | ENSG00000138604 | GLCE |
| ILF3 | ENSG00000236153 | AC104076.3 |
| ILF3 | ENSG00000265918 | RN7SL543P |
| ILF3 | ENSG00000105556 | MIER2 |
| ILF3 | ENSG00000253710 | ALG11 |
| ILF3 | ENSG00000197122 | SRC |
| ILF3 | ENSG00000173302 | GPR148 |
| ILF3 | ENSG00000059145 | UNKL |
| ILF3 | ENSG00000111331 | OAS3 |
| ILF3 | ENSG00000114745 | GORASP1 |
| ILF3 | ENSG00000108599 | AKAP10 |
| ILF3 | ENSG00000247699 | CTB-127C13.1 |
| ILF3 | ENSG00000011422 | PLAUR |
| ILF3 | ENSG00000162772 | ATF3 |
| ILF3 | ENSG00000271598 | CTD- |
| ILF3 | ENSG00000018869 | 2542C24.9  ZNF582 |
| ILF3 | ENSG00000206634 | SNORA22 |
| ILF3 | ENSG00000160862 | AZGP1 |
| ILF3 | ENSG00000251445 | RP11-483A20.3 |
| ILF3 | ENSG00000204052 | LRRC73 |
| ILF3 | ENSG00000251072 | RP11-434D11.4 |
| ILF3 | ENSG00000101150 | TPD52L2 |
| ILF3 | ENSG00000090470 | PDCD7 |
| ILF3 | ENSG00000206836 | RNU6-1029P |
| ILF3 | ENSG00000241493 | RP11-274B21.5 |
| ILF3 | ENSG00000260052 | CTC-527H23.3 |
| ILF3 | ENSG00000200225 | RNA5SP382 |
| ILF3 | ENSG00000100099 | HPS4 |
| ILF3 | ENSG00000060688 | SNRNP40 |
| ILF3 | ENSG00000078747 | ITCH |
| ILF3 | ENSG00000117543 | DPH5 |
| ILF3 | ENSG00000204604 | ZNF468 |
| ILF3 | ENSG00000198894 | CIPC |

| ILF3 | ENSG00000026025 | VIM |
| --- | --- | --- |
| ILF3 | ENSG00000100162 | CENPM |
| ILF3 | ENSG00000166349 | RAG1 |
| ILF3 | ENSG00000258668 | COX6CP11 |
| ILF3 | ENSG00000264343 | NOTCH2NL |
| ILF3 | ENSG00000072121 | ZFYVE26 |
| ILF3 | ENSG00000153558 | FBXL2 |
| ILF3 | ENSG00000183742 | MACC1 |
| ILF3 | ENSG00000100485 | SOS2 |
| ILF3 | ENSG00000187838 | TMEM256- |
| ILF3 | ENSG00000183765 | PLSCR3  CHEK2 |
| ILF3 | ENSG00000259054 | AE000662.93 |
| ILF3 | ENSG00000175356 | SCUBE2 |
| ILF3 | ENSG00000175414 | ARL10 |
| ILF3 | ENSG00000259891 | CTA-204B4.2 |
| ILF3 | ENSG00000222019 | URAHP |
| ILF3 | ENSG00000221792 | MIR1282 |
| ILF3 | ENSG00000254372 | RP11-343P9.1 |
| ILF3 | ENSG00000228439 | TSTD3 |
| ILF3 | ENSG00000204120 | GIGYF2 |
| ILF3 | ENSG00000111799 | COL12A1 |
| ILF3 | ENSG00000276116 | FUT8-AS1 |
| ILF3 | ENSG00000145107 | TM4SF19 |
| ILF3 | ENSG00000141985 | SH3GL1 |
| ILF3 | ENSG00000248905 | FMN1 |
| ILF3 | ENSG00000224352 | AC132479.4 |
| ILF3 | ENSG00000213304 | CTC-398G3.1 |
| ILF3 | ENSG00000257285 | RP11-298I3.1 |
| ILF3 | ENSG00000184986 | TMEM121 |
| ILF3 | ENSG00000235245 | RP11-122K13.12 |
| ILF3 | ENSG00000100290 | BIK |
| ILF3 | ENSG00000100731 | PCNX |
| ILF3 | ENSG00000077463 | SIRT6 |
| ILF3 | ENSG00000234431 | AC007283.5 |
| ILF3 | ENSG00000235295 | FLJ41941 |
| ILF3 | ENSG00000276957 | RP11-29B2.6 |
| ILF3 | ENSG00000198860 | TSEN15 |
| ILF3 | ENSG00000226744 | AC079781.5 |
| ILF3 | ENSG00000239625 | RN7SL241P |
| ILF3 | ENSG00000262429 | RP5-1050D4.3 |
| ILF3 | ENSG00000116560 | SFPQ |
| ILF3 | ENSG00000068394 | GPKOW |
| ILF3 | ENSG00000171570 | RAB4B-EGLN2 |
| ILF3 | ENSG00000268066 | FMR1-AS1 |
| ILF3 | ENSG00000254154 | RP4-798P15.3 |
| ILF3 | ENSG00000269139 | CTD-3193O13.8 |
| ILF3 | ENSG00000118518 | RNF146 |
| ILF3 | ENSG00000279098 | RP11-15B17.4 |
| ILF3 | ENSG00000259595 | RP11-516C1.1 |
| ILF3 | ENSG00000167797 | CDK2AP2 |
| ILF3 | ENSG00000111450 | STX2 |

| ILF3 | ENSG00000196505 | GDAP2 |
| --- | --- | --- |
| ILF3 | ENSG00000151694 | ADAM17 |
| ILF3 | ENSG00000134086 | VHL |
| ILF3 | ENSG00000206145 | P2RX6P |
| ILF3 | ENSG00000051341 | POLQ |
| ILF3 | ENSG00000197753 | LHFPL5 |
| ILF3 | ENSG00000253408 | RP11-231D20.2 |
| ILF3 | ENSG00000240303 | ACAD11 |
| ILF3 | ENSG00000130522 | JUND |
| ILF3 | ENSG00000260133 | CA5AP1 |
| ILF3 | ENSG00000125351 | UPF3B |
| ILF3 | ENSG00000110090 | CPT1A |
| ILF3 | ENSG00000164626 | KCNK5 |
| ILF3 | ENSG00000140931 | CMTM3 |
| ILF3 | ENSG00000253445 | CTB-79E8.2 |
| ILF3 | ENSG00000265168 | RP11-192H23.5 |
| ILF3 | ENSG00000123815 | ADCK4 |
| ILF3 | ENSG00000248256 | OCIAD1-AS1 |
| ILF3 | ENSG00000144228 | SPOPL |
| ILF3 | ENSG00000116285 | ERRFI1 |
| ILF3 | ENSG00000263583 | MIR4522 |
| ILF3 | ENSG00000258001 | RP11-756H6.1 |
| ILF3 | ENSG00000205740 | RP11-363N22.3 |
| ILF3 | ENSG00000175877 | WBSCR28 |
| ILF3 | ENSG00000075914 | EXOSC7 |
| ILF3 | ENSG00000160172 | FAM86C2P |
| ILF3 | ENSG00000170425 | ADORA2B |
| ILF3 | ENSG00000269524 | CTB-83J4.1 |
| ILF3 | ENSG00000228259 | RP11-1379J22.7 |
| ILF3 | ENSG00000174109 | C16orf91 |
| ILF3 | ENSG00000023287 | RB1CC1 |
| ILF3 | ENSG00000131023 | LATS1 |
| ILF3 | ENSG00000187980 | PLA2G2C |
| ILF3 | ENSG00000248508 | SRP14-AS1 |
| ILF3 | ENSG00000267314 | AC104532.2 |
| ILF3 | ENSG00000104918 | RETN |
| ILF3 | ENSG00000171823 | FBXL14 |
| ILF3 | ENSG00000203485 | INF2 |
| ILF3 | ENSG00000188613 | NANOS1 |
| ILF3 | ENSG00000213303 | CTC-398G3.2 |
| ILF3 | ENSG00000213445 | SIPA1 |
| ILF3 | ENSG00000280400 | RP11-20G6.1 |
| ILF3 | ENSG00000265981 | MIR544B |
| ILF3 | ENSG00000106829 | TLE4 |
| ILF3 | ENSG00000168495 | POLR3D |
| ILF3 | ENSG00000214369 | AC009967.3 |
| ILF3 | ENSG00000266643 | MIR3677 |
| ILF3 | ENSG00000213433 | RP11-54C4.1 |
| ILF3 | ENSG00000169241 | SLC50A1 |
| ILF3 | ENSG00000084073 | ZMPSTE24 |
| ILF3 | ENSG00000198087 | CD2AP |

| ILF3 | ENSG00000261284 | RBM22P13 |
| --- | --- | --- |
| ILF3 | ENSG00000185298 | CCDC137 |
| ILF3 | ENSG00000176681 | LRRC37A |
| ILF3 | ENSG00000138942 | RNF185 |
| ILF3 | ENSG00000188242 | PP7080 |
| ILF3 | ENSG00000265395 | MIR3944 |
| ILF3 | ENSG00000201742 | RNU6-563P |
| ILF3 | ENSG00000128928 | IVD |
| ILF3 | ENSG00000204791 | CTD-3065J16.6 |
| ILF3 | ENSG00000207333 | RNU6-680P |
| ILF3 | ENSG00000238132 | CASC4P1 |
| ILF3 | ENSG00000124074 | ENKD1 |
| ILF3 | ENSG00000068912 | ERLEC1 |
| ILF3 | ENSG00000228998 | RP11-697E2.7 |
| ILF3 | ENSG00000235101 | SETP9 |
| ILF3 | ENSG00000155903 | RASA2 |
| ILF3 | ENSG00000158623 | COPG2 |
| ILF3 | ENSG00000240723 | RN7SL382P |
| ILF3 | ENSG00000198833 | UBE2J1 |
| ILF3 | ENSG00000262049 | RP13-1032I1.7 |
| ILF3 | ENSG00000186352 | ANKRD37 |
| ILF3 | ENSG00000121644 | DESI2 |
| ILF3 | ENSG00000238297 | U3 |
| ILF3 | ENSG00000090581 | GNPTG |
| ILF3 | ENSG00000172728 | FUT10 |
| ILF3 | ENSG00000106462 | EZH2 |
| ILF3 | ENSG00000140009 | ESR2 |
| ILF3 | ENSG00000163686 | ABHD6 |
| ILF3 | ENSG00000024422 | EHD2 |
| ILF3 | ENSG00000228398 | HMGN2P25 |
| ILF3 | ENSG00000266959 | AC005786.3 |
| ILF3 | ENSG00000270049 | RP11-297D21.4 |
| ILF3 | ENSG00000125454 | SLC25A19 |
| ILF3 | ENSG00000133961 | NUMB |
| ILF3 | ENSG00000128011 | LRFN1 |
| ILF3 | ENSG00000246526 | RP11-539L10.2 |
| ILF3 | ENSG00000160691 | SHC1 |
| ILF3 | ENSG00000265818 | EEF1E1- |
| ILF3 | ENSG00000236423 | BLOC1S5 LINC01134 |
| ILF3 | ENSG00000269275 | CTD-2105E13.15 |
| ILF3 | ENSG00000130598 | TNNI2 |
| ILF3 | ENSG00000179546 | HTR1D |
| ILF3 | ENSG00000187866 | FAM122A |
| ILF3 | ENSG00000184436 | THAP7 |
| ILF3 | ENSG00000175216 | CKAP5 |
| ILF3 | ENSG00000142396 | ERVK3-1 |
| ILF3 | ENSG00000278834 | RP11-458J1.1 |
| ILF3 | ENSG00000266472 | MRPS21 |
| ILF3 | ENSG00000261465 | RP11-626G11.5 |
| ILF3 | ENSG00000198133 | TMEM229B |
| ILF3 | ENSG00000273175 | RP11-11N7.4 |

| ILF3 | ENSG00000108021 | FAM208B |
| --- | --- | --- |
| ILF3 | ENSG00000279713 | CTD-3035K23.3 |
| ILF3 | ENSG00000198951 | NAGA |
| ILF3 | ENSG00000083093 | PALB2 |
| ILF3 | ENSG00000273521 | RP11-45A17.4 |
| ILF3 | ENSG00000174446 | SNAPC5 |
| ILF3 | ENSG00000082068 | WDR70 |
| ILF3 | ENSG00000150628 | SPATA4 |
| ILF3 | ENSG00000169155 | ZBTB43 |
| ILF3 | ENSG00000249352 | RP11-141O11.2 |
| ILF3 | ENSG00000228315 | GUSBP11 |
| ILF3 | ENSG00000079950 | STX7 |
| ILF3 | ENSG00000177479 | ARIH2 |
| ILF3 | ENSG00000249982 | RP11-1018N14.3 |
| ILF3 | ENSG00000228727 | SAPCD1 |
| ILF3 | ENSG00000272149 | RP11-627J17.1 |
| ILF3 | ENSG00000092051 | JPH4 |
| ILF3 | ENSG00000146828 | SLC12A9 |
| ILF3 | ENSG00000278404 | MIAT_exon1 |
| ILF3 | ENSG00000205744 | DENND1C |
| ILF3 | ENSG00000185437 | SH3BGR |
| ILF3 | ENSG00000085999 | RAD54L |
| ILF3 | ENSG00000102974 | CTCF |
| ILF3 | ENSG00000228830 | RP4-781K5.2 |
| ILF3 | ENSG00000214941 | ZSWIM7 |
| ILF3 | ENSG00000180747 | SMG1P3 |
| ILF3 | ENSG00000159208 | CIART |
| ILF3 | ENSG00000242531 | RP11-299J3.6 |
| ILF3 | ENSG00000153904 | DDAH1 |
| ILF3 | ENSG00000196352 | CD55 |
| ILF3 | ENSG00000240925 | RPS20P31 |
| ILF3 | ENSG00000112130 | RNF8 |
| ILF3 | ENSG00000275711 | MIR6795 |
| ILF3 | ENSG00000089195 | TRMT6 |
| ILF3 | ENSG00000279886 | RP11-219G17.8 |
| ILF3 | ENSG00000255836 | RP11-157G21.2 |
| ILF3 | ENSG00000160999 | SH2B2 |
| ILF3 | ENSG00000153898 | MCOLN2 |
| ILF3 | ENSG00000178149 | DALRD3 |
| ILF3 | ENSG00000136828 | RALGPS1 |
| ILF3 | ENSG00000133243 | BTBD2 |
| ILF3 | ENSG00000159140 | SON |
| ILF3 | ENSG00000214646 | RP11-114H24.4 |
| ILF3 | ENSG00000171017 | LRRC8E |
| ILF3 | ENSG00000179950 | PUF60 |
| ILF3 | ENSG00000155714 | PDZD9 |
| ILF3 | ENSG00000226706 | RP11-426A6.5 |
| ILF3 | ENSG00000175189 | INHBC |
| ILF3 | ENSG00000105676 | ARMC6 |
| ILF3 | ENSG00000183735 | TBK1 |
| ILF3 | ENSG00000232940 | HCG25 |

| ILF3 | ENSG00000257303 | RP11-977G19.11 |
| --- | --- | --- |
| ILF3 | ENSG00000199903 | RNU6-1273P |
| ILF3 | ENSG00000235843 | RP13-16H11.5 |
| ILF3 | ENSG00000103375 | AQP8 |
| ILF3 | ENSG00000101337 | TM9SF4 |
| ILF3 | ENSG00000274833 | RP11-1055B8.10 |
| ILF3 | ENSG00000142252 | GEMIN7 |
| ILF3 | ENSG00000197879 | MYO1C |
| ILF3 | ENSG00000199715 | Y_RNA |
| ILF3 | ENSG00000189046 | ALKBH2 |
| ILF3 | ENSG00000143363 | PRUNE |
| ILF3 | ENSG00000145868 | FBXO38 |
| ILF3 | ENSG00000131233 | GJA9 |
| ILF3 | ENSG00000256116 | RP11-783K16.14 |
| ILF3 | ENSG00000198420 | FAM115A |
| ILF3 | ENSG00000134830 | C5AR2 |
| ILF3 | ENSG00000229502 | RP11-52J3.2 |
| ILF3 | ENSG00000275481 | RP11-474P2.6 |
| ILF3 | ENSG00000166387 | PPFIBP2 |
| ILF3 | ENSG00000257365 | FNTB |
| ILF3 | ENSG00000115816 | CEBPZ |
| ILF3 | ENSG00000264769 | RP11-498C9.12 |
| ILF3 | ENSG00000101294 | HM13 |
| ILF3 | ENSG00000236930 | RP11-852E15.1 |
| ILF3 | ENSG00000252316 | RNY4 |
| ILF3 | ENSG00000267595 | RP11-242D8.2 |
| ILF3 | ENSG00000143252 | SDHC |
| ILF3 | ENSG00000228008 | CTD-2330K9.3 |
| ILF3 | ENSG00000132801 | ZSWIM3 |
| ILF3 | ENSG00000177752 | YIPF7 |
| ILF3 | ENSG00000129071 | MBD4 |
| ILF3 | ENSG00000168214 | RBPJ |
| ILF3 | ENSG00000155158 | TTC39B |
| ILF3 | ENSG00000135709 | KIAA0513 |
| ILF3 | ENSG00000127743 | IL17B |
| ILF3 | ENSG00000178028 | DMAP1 |
| ILF3 | ENSG00000151725 | CENPU |
| ILF3 | ENSG00000201207 | Y_RNA |
| ILF3 | ENSG00000262115 | RP11-455O6.2 |
| ILF3 | ENSG00000145675 | PIK3R1 |
| ILF3 | ENSG00000273176 | RP3-510H16.3 |
| ILF3 | ENSG00000146729 | GBAS |
| ILF3 | ENSG00000260017 | RP11-1035H13.2 |
| ILF3 | ENSG00000262050 | RP11-74E22.3 |
| ILF3 | ENSG00000279609 | RP11-235C23.6 |
| ILF3 | ENSG00000172638 | EFEMP2 |
| ILF3 | ENSG00000163539 | CLASP2 |
| ILF3 | ENSG00000142273 | CBLC |
| ILF3 | ENSG00000259661 | AC068831.15 |
| ILF3 | ENSG00000260895 | RP11-554A11.7 |
| ILF3 | ENSG00000185100 | ADSSL1 |

| ILF3 | ENSG00000265972 | TXNIP |
| --- | --- | --- |
| ILF3 | ENSG00000178585 | CTNNBIP1 |
| ILF3 | ENSG00000137168 | PPIL1 |
| ILF3 | ENSG00000106009 | BRAT1 |
| ILF3 | ENSG00000080947 | CROCCP3 |
| ILF3 | ENSG00000141424 | SLC39A6 |
| ILF3 | ENSG00000231365 | RP11-418J17.1 |
| ILF3 | ENSG00000160877 | NACC1 |
| ILF3 | ENSG00000207067 | SNORA72 |
| ILF3 | ENSG00000196937 | FAM3C |
| ILF3 | ENSG00000198720 | ANKRD13B |
| ILF3 | ENSG00000223387 | RP11-408H1.3 |
| ILF3 | ENSG00000276980 | CTD- |
| ILF3 | ENSG00000122696 | 3128G10.7 SLC25A51 |
| ILF3 | ENSG00000214226 | C17orf67 |
| ILF3 | ENSG00000127527 | EPS15L1 |
| ILF3 | ENSG00000204060 | FOXO6 |
| ILF3 | ENSG00000065485 | PDIA5 |
| ILF3 | ENSG00000252122 | SNORA76 |
| ILF3 | ENSG00000205106 | AC044839.1 |
| ILF3 | ENSG00000186222 | BLOC1S4 |
| ILF3 | ENSG00000067829 | IDH3G |
| ILF3 | ENSG00000207500 | SNORD102 |
| ILF3 | ENSG00000068323 | TFE3 |
| ILF3 | ENSG00000207650 | MIR570 |
| ILF3 | ENSG00000253227 | RP11-383J24.1 |
| ILF3 | ENSG00000259521 | RP11-540O11.4 |
| ILF3 | ENSG00000274309 | SNORA71E |
| ILF3 | ENSG00000229882 | RP13-30A9.2 |
| ILF3 | ENSG00000268536 | AC005523.3 |
| ILF3 | ENSG00000090932 | DLL3 |
| ILF3 | ENSG00000213927 | CCL27 |
| ILF3 | ENSG00000175087 | PDIK1L |
| ILF3 | ENSG00000106330 | MOSPD3 |
| ILF3 | ENSG00000176371 | ZSCAN2 |
| ILF3 | ENSG00000083168 | KAT6A |
| ILF3 | ENSG00000170275 | CRTAP |
| ILF3 | ENSG00000168906 | MAT2A |
| ILF3 | ENSG00000186635 | ARAP1 |
| ILF3 | ENSG00000243431 | RPL5P30 |
| ILF3 | ENSG00000168092 | PAFAH1B2 |
| ILF3 | ENSG00000076928 | ARHGEF1 |
| ILF3 | ENSG00000175567 | UCP2 |
| ILF3 | ENSG00000129226 | CD68 |
| ILF3 | ENSG00000272969 | RP11-528I4.2 |
| ILF3 | ENSG00000174437 | ATP2A2 |
| ILF3 | ENSG00000206633 | SNORA80B |
| ILF3 | ENSG00000275709 | RP11-96O20.5 |
| ILF3 | ENSG00000100225 | FBXO7 |
| ILF3 | ENSG00000102805 | CLN5 |
| ILF3 | ENSG00000251271 | ALG1L7P |

| ILF3 ILF3 ILF3 | ENSG00000230733 ENSG00000179094 ENSG00000234608 | AC092171.4 PER1  MAPKAPK5- AS1 |
| --- | --- | --- |
| ILF3 | ENSG00000259456 | ADNP-AS1 |
| ILF3 | ENSG00000163635 | ATXN7 |
| ILF3 | ENSG00000254829 | RP11-7I15.3 |
| ILF3 | ENSG00000198826 | ARHGAP11A |
| ILF3 | ENSG00000274986 | MIR6750 |
| ILF3 | ENSG00000198663 | C6orf89 |
| ILF3 | ENSG00000252969 | SNORA70 |
| ILF3 | ENSG00000237461 | RP11-554F20.1 |
| ILF3 | ENSG00000167851 | CD300A |
| ILF3 | ENSG00000250568 | RP11-333E13.2 |
| ILF3 | ENSG00000229766 | RP5-971N18.3 |
| ILF3 | ENSG00000238280 | RP11-436D10.3 |
| ILF3 | ENSG00000185261 | KIAA0825 |
| ILF3 | ENSG00000263818 | CTD-2206N4.4 |
| ILF3 | ENSG00000225264 | ZNRF2P2 |
| ILF3 | ENSG00000123992 | DNPEP |
| ILF3 | ENSG00000160991 | ORAI2 |
| ILF3 | ENSG00000235934 | AC007405.8 |
| ILF3 | ENSG00000228040 | RP3-522D1.2 |
| ILF3 | ENSG00000206693 | SNORA56 |
| ILF3 | ENSG00000169682 | SPNS1 |
| ILF3 | ENSG00000255200 | AP003068.18 |
| ILF3 | ENSG00000106733 | NMRK1 |
| ILF3 | ENSG00000249680 | TUBA3GP |
| ILF3 | ENSG00000277186 | RP13-554M15.8 |
| ILF3 | ENSG00000113269 | RNF130 |
| ILF3 | ENSG00000172057 | ORMDL3 |
| ILF3 | ENSG00000231856 | RP11-327P2.5 |
| ILF3 | ENSG00000235527 | HIPK1-AS1 |
| ILF3 | ENSG00000248334 | WHAMMP2 |
| ILF3 | ENSG00000203761 | MSTO2P |
| ILF3 | ENSG00000273428 | RP4-539M6.22 |
| ILF3 | ENSG00000261481 | RP11-77H9.6 |
| ILF3 | ENSG00000278107 | RP11-162A12.4 |
| ILF3 | ENSG00000234515 | PPP1R2P1 |
| ILF3 | ENSG00000130695 | CEP85 |
| ILF3 | ENSG00000129317 | PUS7L |
| ILF3 | ENSG00000198055 | GRK6 |
| ILF3 | ENSG00000245317 | CTC-241N9.1 |
| ILF3 | ENSG00000147394 | ZNF185 |
| ILF3 | ENSG00000215840 | RP11-122G18.7 |
| ILF3 | ENSG00000275401 | RP4-564F22.7 |
| ILF3 | ENSG00000162891 | IL20 |
| ILF3 | ENSG00000278467 | RP11-504P24.9 |
| ILF3 | ENSG00000276291 | RP11-87H9.2 |
| ILF3 | ENSG00000231978 | RP11-132G19.3 |
| ILF3 | ENSG00000181513 | ACBD4 |
| ILF3 | ENSG00000280177 | RP5-906A24.1 |

| ILF3 | ENSG00000230638 | RP11-486B10.4 |
| --- | --- | --- |
| ILF3 | ENSG00000244748 | RN7SL153P |
| ILF3 | ENSG00000277478 | MIR6165 |
| ILF3 | ENSG00000125652 | ALKBH7 |
| ILF3 | ENSG00000272146 | RP11-755B10.4 |
| ILF3 | ENSG00000265749 | RP11-849F2.5 |
| ILF3 | ENSG00000165669 | FAM204A |
| ILF3 | ENSG00000224786 | CETN4P |
| ILF3 | ENSG00000273516 | U1 |
| ILF3 | ENSG00000221498 | SNORA77 |
| ILF3 | ENSG00000267544 | AC007229.3 |
| ILF3 | ENSG00000149582 | TMEM25 |
| ILF3 | ENSG00000187713 | TMEM203 |
| ILF3 | ENSG00000081791 | KIAA0141 |
| ILF3 | ENSG00000100916 | BRMS1L |
| ILF3 | ENSG00000120686 | UFM1 |
| ILF3 | ENSG00000166925 | TSC22D4 |
| ILF3 | ENSG00000075856 | SART3 |
| ILF3 | ENSG00000204540 | PSORS1C1 |
| ILF3 | ENSG00000118689 | FOXO3 |
| ILF3 | ENSG00000271420 | RP5-1057J7.7 |
| ILF3 | ENSG00000148832 | PAOX |
| ILF3 | ENSG00000278899 | AL358852.1 |
| ILF3 | ENSG00000277142 | LINC00235 |
| ILF3 | ENSG00000001084 | GCLC |
| ILF3 | ENSG00000198189 | HSD17B11 |
| ILF3 | ENSG00000220643 | RP3-391O22.1 |
| ILF3 | ENSG00000267259 | CTD-2008P7.9 |
| ILF3 | ENSG00000214753 | HNRNPUL2 |
| ILF3 | ENSG00000270638 | RP3-466P17.1 |
| ILF3 | ENSG00000118495 | PLAGL1 |
| ILF3 | ENSG00000271781 | CTD- |
| ILF3 | ENSG00000175182 | 2589H19.6  FAM131A |
| ILF3 | ENSG00000273658 | PCBP2-OT1 |
| ILF3 | ENSG00000224292 | AF196972.9 |
| ILF3 | ENSG00000157890 | MEGF11 |
| ILF3 | ENSG00000164961 | KIAA0196 |
| ILF3 | ENSG00000142798 | HSPG2 |
| ILF3 | ENSG00000276410 | HIST1H2BB |
| ILF3 | ENSG00000187231 | SESTD1 |
| ILF3 | ENSG00000271717 | CTD- |
| ILF3 | ENSG00000175489 | 3020H12.4  LRRC25 |
| ILF3 | ENSG00000147130 | ZMYM3 |
| ILF3 | ENSG00000163728 | TTC14 |
| ILF3 | ENSG00000129518 | EAPP |
| ILF3 | ENSG00000215734 | MRPL20P1 |
| ILF3 | ENSG00000100889 | PCK2 |
| ILF3 | ENSG00000272973 | KB-1125A3.11 |
| ILF3 | ENSG00000031698 | SARS |
| ILF3 | ENSG00000143498 | TAF1A |

ILF3 ENSG00000148346 LCN2

| ILF3 | ENSG00000131931 | THAP1 |
| --- | --- | --- |
| ILF3 | ENSG00000211689 | TRGC1 |
| ILF3 | ENSG00000183684 | ALYREF |
| ILF3 | ENSG00000130283 | GDF1 |
| ILF3 | ENSG00000212443 | SNORA53 |
| ILF3 | ENSG00000186193 | SAPCD2 |
| ILF3 | ENSG00000110324 | IL10RA |
| ILF3 | ENSG00000158874 | APOA2 |
| ILF3 | ENSG00000225964 | NRIR |
| ILF3 | ENSG00000263466 | RP1-56K13.2 |
| ILF3 | ENSG00000226688 | ENTPD1-AS1 |
| ILF3 | ENSG00000267091 | CTBP2P7 |
| ILF3 | ENSG00000112096 | SOD2 |
| ILF3 | ENSG00000131711 | MAP1B |
| ILF3 | ENSG00000254367 | RP11-211C9.1 |
| ILF3 | ENSG00000228792 | RP11-354K1.2 |
| ILF3 | ENSG00000005302 | MSL3 |
| ILF3 | ENSG00000104388 | RAB2A |
| ILF3 | ENSG00000265246 | RP11-663N22.1 |
| ILF3 | ENSG00000139160 | METTL20 |
| ILF3 | ENSG00000203747 | FCGR3A |
| ILF3 | ENSG00000177800 | TMEM78 |
| ILF3 | ENSG00000178502 | KLHL11 |
| ILF3 | ENSG00000060339 | CCAR1 |
| ILF3 | ENSG00000168140 | VASN |
| ILF3 | ENSG00000202332 | Y_RNA |
| ILF3 | ENSG00000177628 | GBA |
| ILF3 | ENSG00000160633 | SAFB |
| ILF3 | ENSG00000119487 | MAPKAP1 |
| ILF3 | ENSG00000277001 | Metazoa_SRP |
| ILF3 | ENSG00000003509 | NDUFAF7 |
| ILF3 | ENSG00000249685 | RP11-360F5.3 |
| ILF3 | ENSG00000167644 | C19orf33 |
| ILF3 | ENSG00000083312 | TNPO1 |
| ILF3 | ENSG00000201558 | RNVU1-6 |
| ILF3 | ENSG00000138398 | PPIG |
| ILF3 | ENSG00000143153 | ATP1B1 |
| ILF3 | ENSG00000105339 | DENND3 |
| ILF3 | ENSG00000257848 | RP11-474C8.5 |
| ILF3 | ENSG00000148399 | DPH7 |
| ILF3 | ENSG00000105968 | H2AFV |
| ILF3 | ENSG00000163710 | PCOLCE2 |
| ILF3 | ENSG00000173531 | MST1 |
| ILF3 | ENSG00000204619 | PPP1R11 |
| ILF3 | ENSG00000079974 | RABL2B |
| ILF3 | ENSG00000251831 | RNU6-1114P |
| ILF3 | ENSG00000251988 | RNU4ATAC18 |
| ILF3 | ENSG00000041988 | P THAP3 |
| ILF3 | ENSG00000243423 | RP5-837J1.1 |
| ILF3 | ENSG00000279114 | RP3-425C14.5 |
| ILF3 | ENSG00000112200 | ZNF451 |

| ILF3 | ENSG00000105379 | ETFB |
| --- | --- | --- |
| ILF3 | ENSG00000142235 | LMTK3 |
| ILF3 | ENSG00000213551 | DNAJC9 |
| ILF3 | ENSG00000274997 | HIST1H2AH |
| ILF3 | ENSG00000231005 | RP3-481F12.1 |
| ILF3 | ENSG00000169026 | MFSD7 |
| ILF3 | ENSG00000166016 | ABTB2 |
| ILF3 | ENSG00000260928 | SPCS2P1 |
| ILF3 | ENSG00000237641 | RP11-690I21.1 |
| ILF3 | ENSG00000213020 | ZNF611 |
| ILF3 | ENSG00000248240 | RP11-159F24.5 |
| ILF3 | ENSG00000254662 | RP11-872D17.4 |
| ILF3 | ENSG00000205318 | GCNT6 |
| ILF3 | ENSG00000202198 | RN7SK |
| ILF3 | ENSG00000054690 | PLEKHH1 |
| ILF3 | ENSG00000261147 | RP11-697E2.6 |
| ILF3 | ENSG00000106211 | HSPB1 |
| ILF3 | ENSG00000207956 | MIR579 |
| ILF3 | ENSG00000272888 | AC013394.2 |
| ILF3 | ENSG00000118894 | FAM86A |
| ILF3 | ENSG00000047932 | GOPC |
| ILF3 | ENSG00000162669 | HFM1 |
| ILF3 | ENSG00000255561 | FDXACB1 |
| ILF3 | ENSG00000271380 | RP11-307C12.12 |
| ILF3 | ENSG00000264057 | RP11-583F2.1 |
| ILF3 | ENSG00000070367 | EXOC5 |
| ILF3 | ENSG00000100116 | GCAT |
| ILF3 | ENSG00000182264 | IZUMO1 |
| ILF3 | ENSG00000171604 | CXXC5 |
| ILF3 | ENSG00000237054 | PRMT5-AS1 |
| ILF3 | ENSG00000111602 | TIMELESS |
| ILF3 | ENSG00000186787 | SPIN2B |
| ILF3 | ENSG00000133063 | CHIT1 |
| ILF3 | ENSG00000113302 | IL12B |
| ILF3 | ENSG00000140650 | PMM2 |
| ILF3 | ENSG00000267546 | RP11-666A8.8 |
| ILF3 | ENSG00000231768 | LINC01354 |
| ILF3 | ENSG00000139697 | SBNO1 |
| ILF3 | ENSG00000148730 | EIF4EBP2 |
| ILF3 | ENSG00000204758 | CTC-308K20.1 |
| ILF3 | ENSG00000119638 | NEK9 |
| ILF3 | ENSG00000260273 | RP11-425D10.10 |
| ILF3 | ENSG00000156853 | ZNF689 |
| ILF3 | ENSG00000243446 | RN7SL284P |
| ILF3 | ENSG00000205765 | C5orf51 |
| ILF3 | ENSG00000143324 | XPR1 |
| ILF3 | ENSG00000243968 | RN7SL402P |
| ILF3 | ENSG00000184163 | FAM132A |
| ILF3 | ENSG00000160570 | DEDD2 |
| ILF3 | ENSG00000231890 | DARS-AS1 |
| ILF3 | ENSG00000182793 | GSTA5 |

| ILF3 | ENSG00000198870 | STKLD1 |
| --- | --- | --- |
| ILF3 | ENSG00000255730 | CTC-435M10.3 |
| ILF3 | ENSG00000231367 | AC016995.3 |
| ILF3 | ENSG00000117054 | ACADM |
| ILF3 | ENSG00000247271 | ZBED5-AS1 |
| ILF3 | ENSG00000108830 | RND2 |
| ILF3 | ENSG00000146223 | RPL7L1 |
| ILF3 | ENSG00000163121 | NEURL3 |
| ILF3 | ENSG00000141429 | GALNT1 |
| ILF3 | ENSG00000276213 | Metazoa_SRP |
| ILF3 | ENSG00000138794 | CASP6 |
| ILF3 | ENSG00000104983 | CCDC61 |
| ILF3 | ENSG00000103037 | SETD6 |
| ILF3 | ENSG00000168918 | INPP5D |
| ILF3 | ENSG00000006468 | ETV1 |
| ILF3 | ENSG00000161132 | XXbac-B444P24.10 |
| ILF3 | ENSG00000149403 | GRIK4 |
| ILF3 | ENSG00000060558 | GNA15 |
| ILF3 | ENSG00000187475 | HIST1H1T |
| ILF3 | ENSG00000232437 | RP11-487I5.4 |
| ILF3 | ENSG00000118873 | RAB3GAP2 |
| ILF3 | ENSG00000271966 | RP11-7F18.2 |
| ILF3 | ENSG00000163794 | UCN |
| ILF3 | ENSG00000148572 | NRBF2 |
| ILF3 | ENSG00000170471 | RALGAPB |
| ILF3 | ENSG00000148719 | DNAJB12 |
| ILF3 | ENSG00000271983 | RP11-28H5.2 |
| ILF3 | ENSG00000133059 | DSTYK |
| ILF3 | ENSG00000142530 | FAM71E1 |
| ILF3 | ENSG00000204569 | PPP1R10 |
| ILF3 | ENSG00000277510 | Metazoa_SRP |
| ILF3 | ENSG00000233922 | AL133493.2 |
| ILF3 | ENSG00000273382 | RP5-1065J22.8 |
| ILF3 | ENSG00000251864 | Y_RNA |
| ILF3 | ENSG00000235112 | HSPE1P27 |
| ILF3 | ENSG00000165632 | TAF3 |
| ILF3 | ENSG00000229539 | RP11-119B16.2 |
| ILF3 | ENSG00000066926 | FECH |
| ILF3 | ENSG00000239393 | CTD-2301A4.1 |
| ILF3 | ENSG00000136603 | SKIL |
| ILF3 | ENSG00000196323 | ZBTB44 |
| ILF3 | ENSG00000112561 | TFEB |
| ILF3 | ENSG00000164494 | PDSS2 |
| ILF3 | ENSG00000242282 | AC108488.4 |
| ILF3 | ENSG00000244239 | AC007009.1 |
| ILF3 | ENSG00000234030 | TMEM97P1 |
| ILF3 | ENSG00000188662 | HILS1 |
| ILF3 | ENSG00000188051 | TMEM221 |
| ILF3 | ENSG00000223446 | RP11-274J16.5 |
| ILF3 | ENSG00000101940 | WDR13 |
| ILF3 | ENSG00000125734 | GPR108 |

| ILF3 | ENSG00000153048 | CARHSP1 |
| --- | --- | --- |
| ILF3 | ENSG00000196659 | TTC30B |
| ILF3 | ENSG00000100321 | SYNGR1 |
| ILF3 | ENSG00000120063 | GNA13 |
| ILF3 | ENSG00000168010 | ATG16L2 |
| ILF3 | ENSG00000143355 | LHX9 |
| ILF3 | ENSG00000201898 | SNORA72 |
| ILF3 | ENSG00000215012 | C22orf29 |
| ILF3 | ENSG00000273106 | RP11-559M23.1 |
| ILF3 | ENSG00000140382 | HMG20A |
| ILF3 | ENSG00000167985 | SDHAF2 |
| ILF3 | ENSG00000275924 | MIR6807 |
| ILF3 | ENSG00000134042 | MRO |
| ILF3 | ENSG00000214160 | ALG3 |
| ILF3 | ENSG00000145247 | OCIAD2 |
| ILF3 | ENSG00000265606 | MIR4695 |
| ILF3 | ENSG00000137171 | KLC4 |
| ILF3 | ENSG00000246477 | AF131216.6 |
| ILF3 | ENSG00000259177 | RP11-154B12.3 |
| ILF3 | ENSG00000236528 | RP1-125I3.2 |
| ILF3 | ENSG00000144895 | EIF2A |
| ILF3 | ENSG00000196668 | LINC00173 |
| ILF3 | ENSG00000207585 | MIR181D |
| ILF3 | ENSG00000163466 | ARPC2 |
| ILF3 | ENSG00000104879 | CKM |
| ILF3 | ENSG00000236853 | OR2R1P |
| ILF3 | ENSG00000279789 | CTD- |
| ILF3 | ENSG00000174842 | 2574D22.3 GLMN |
| ILF3 | ENSG00000186566 | GPATCH8 |
| ILF3 | ENSG00000157315 | TMED6 |
| ILF3 | ENSG00000199574 | SNORD18C |
| ILF3 | ENSG00000104356 | POP1 |
| ILF3 | ENSG00000166313 | APBB1 |
| ILF3 | ENSG00000137814 | HAUS2 |
| ILF3 | ENSG00000168876 | ANKRD49 |
| ILF3 | ENSG00000277308 | Metazoa_SRP |
| ILF3 | ENSG00000004455 | AK2 |
| ILF3 | ENSG00000261431 | RP4-616B8.4 |
| ILF3 | ENSG00000104897 | SF3A2 |
| ILF3 | ENSG00000134146 | DPH6 |
| ILF3 | ENSG00000228237 | EFCAB14-AS1 |
| ILF3 | ENSG00000142871 | CYR61 |
| ILF3 | ENSG00000112212 | TSPO2 |
| ILF3 | ENSG00000229239 | RP11-223J15.2 |
| ILF3 | ENSG00000111679 | PTPN6 |
| ILF3 | ENSG00000183172 | SMDT1 |
| ILF3 | ENSG00000217275 | RP1-34B20.4 |
| ILF3 | ENSG00000176986 | SEC24C |
| ILF3 | ENSG00000163399 | ATP1A1 |
| ILF3 | ENSG00000189410 | SH2D5 |
| ILF3 | ENSG00000274417 | MIR6515 |

| ILF3 | ENSG00000252622 | RNU6-881P |
| --- | --- | --- |
| ILF3 | ENSG00000077380 | DYNC1I2 |
| ILF3 | ENSG00000229444 | RP11-184I16.4 |
| ILF3 | ENSG00000168067 | MAP4K2 |
| ILF3 | ENSG00000110203 | FOLR3 |
| ILF3 | ENSG00000213225 | AC018804.7 |
| ILF3 | ENSG00000200247 | RNU6-254P |
| ILF3 | ENSG00000112367 | FIG4 |
| ILF3 | ENSG00000100151 | PICK1 |
| ILF3 | ENSG00000202512 | RN7SKP230 |
| ILF3 | ENSG00000029534 | ANK1 |
| ILF3 | ENSG00000123360 | PDE1B |
| ILF3 | ENSG00000259207 | ITGB3 |
| ILF3 | ENSG00000196189 | SEMA4A |
| ILF3 | ENSG00000101188 | NTSR1 |
| ILF3 | ENSG00000279467 | KB-1125A3.12 |
| ILF3 | ENSG00000225791 | TRAM2-AS1 |
| ILF3 | ENSG00000153531 | ADPRHL1 |
| ILF3 | ENSG00000280063 | RP11-295D4.3 |
| ILF3 | ENSG00000176055 | MBLAC2 |
| ILF3 | ENSG00000231889 | TRAF3IP2-AS1 |
| ILF3 | ENSG00000228353 | RP11-184A2.2 |
| ILF3 | ENSG00000278535 | DHRS11 |
| ILF3 | ENSG00000267185 | PTP4A2P1 |
| ILF3 | ENSG00000276674 | IGKV1OR1-1 |
| ILF3 | ENSG00000011007 | TCEB3 |
| ILF3 | ENSG00000180354 | MTURN |
| ILF3 | ENSG00000213920 | MDP1 |
| ILF3 | ENSG00000008516 | MMP25 |
| ILF3 | ENSG00000199697 | RNU6-446P |
| ILF3 | ENSG00000197728 | RPS26 |
| ILF3 | ENSG00000071655 | MBD3 |
| ILF3 | ENSG00000163521 | GLB1L |
| ILF3 | ENSG00000207631 | MIR641 |
| ILF3 | ENSG00000228365 | RP1-90J20.2 |
| ILF3 | ENSG00000070540 | WIPI1 |
| ILF3 | ENSG00000272817 | RP11-402D21.2 |
| ILF3 | ENSG00000166012 | TAF1D |
| ILF3 | ENSG00000163870 | TPRA1 |
| ILF3 | ENSG00000135821 | GLUL |
| ILF3 | ENSG00000089177 | KIF16B |
| ILF3 | ENSG00000167524 | SGK494 |
| ILF3 | ENSG00000275066 | SYNRG |
| ILF3 | ENSG00000167528 | ZNF641 |
| ILF3 | ENSG00000246016 | LINC01513 |
| ILF3 | ENSG00000125686 | MED1 |
| ILF3 | ENSG00000103550 | KNOP1 |
| ILF3 | ENSG00000235805 | SOCS5P3 |
| ILF3 | ENSG00000136802 | LRRC8A |
| ILF3 | ENSG00000167202 | TBC1D2B |
| ILF3 | ENSG00000235579 | AC007283.4 |

| ILF3 | ENSG00000265112 | MIR3153 |
| --- | --- | --- |
| ILF3 | ENSG00000267052 | CTB-30L5.1 |
| ILF3 | ENSG00000145743 | FBXL17 |
| ILF3 | ENSG00000254990 | RP11-108O10.2 |
| ILF3 | ENSG00000213779 | RP11-452G18.1 |
| ILF3 | ENSG00000189060 | H1F0 |
| ILF3 | ENSG00000125538 | IL1B |
| ILF3 | ENSG00000143971 | ETAA1 |
| ILF3 | ENSG00000169609 | C15orf40 |
| ILF3 | ENSG00000175691 | ZNF77 |
| ILF3 | ENSG00000129195 | FAM64A |
| ILF3 | ENSG00000222361 | RNU6-1186P |
| ILF3 | ENSG00000244671 | RN7SL280P |
| ILF3 | ENSG00000120306 | CYSTM1 |
| ILF3 | ENSG00000086475 | SEPHS1 |
| ILF3 | ENSG00000158552 | ZFAND2B |
| ILF3 | ENSG00000092108 | SCFD1 |
| ILF3 | ENSG00000125266 | EFNB2 |
| ILF3 | ENSG00000106628 | POLD2 |
| ILF3 | ENSG00000272986 | RP11-46J23.1 |
| ILF3 | ENSG00000243927 | MRPS6 |
| ILF3 | ENSG00000114209 | PDCD10 |
| ILF3 | ENSG00000167900 | TK1 |
| ILF3 | ENSG00000092929 | UNC13D |
| ILF3 | ENSG00000184925 | LCN12 |
| ILF3 | ENSG00000265692 | RP13-516M14.4 |
| ILF3 | ENSG00000184465 | WDR27 |
| ILF3 | ENSG00000158805 | ZNF276 |
| ILF3 | ENSG00000163605 | PPP4R2 |
| ILF3 | ENSG00000164687 | FABP5 |
| ILF3 | ENSG00000234127 | TRIM26 |
| ILF3 | ENSG00000116977 | LGALS8 |
| ILF3 | ENSG00000263307 | RP11-166B2.8 |
| ILF3 | ENSG00000163739 | CXCL1 |
| ILF3 | ENSG00000161847 | RAVER1 |
| ILF3 | ENSG00000176890 | TYMS |
| ILF3 | ENSG00000132361 | CLUH |
| ILF3 | ENSG00000206885 | SNORA75 |
| ILF3 | ENSG00000249859 | PVT1 |
| ILF3 | ENSG00000117010 | ZNF684 |
| ILF3 | ENSG00000164411 | GJB7 |
| ILF3 | ENSG00000223382 | RP1-65J11.1 |
| ILF3 | ENSG00000271937 | RP11-424N24.2 |
| ILF3 | ENSG00000056097 | ZFR |
| ILF3 | ENSG00000051128 | HOMER3 |
| ILF3 | ENSG00000170310 | STX8 |
| ILF3 | ENSG00000234327 | AC012146.7 |
| ILF3 | ENSG00000269891 | ARHGAP19- |
| ILF3 | ENSG00000121067 | SLIT1  SPOP |
| ILF3 | ENSG00000267772 | RP11-15E18.4 |
| ILF3 | ENSG00000158691 | ZSCAN12 |

| ILF3 | ENSG00000261135 | RP4-798A10.7 |
| --- | --- | --- |
| ILF3 | ENSG00000243521 | RPL5P33 |
| ILF3 | ENSG00000141027 | NCOR1 |
| ILF3 | ENSG00000127054 | CPSF3L |
| ILF3 | ENSG00000202251 | Y_RNA |
| ILF3 | ENSG00000179364 | PACS2 |
| ILF3 | ENSG00000106089 | STX1A |
| ILF3 | ENSG00000164398 | ACSL6 |
| ILF3 | ENSG00000166455 | C16orf46 |
| ILF3 | ENSG00000200985 | RNA5SP493 |
| ILF3 | ENSG00000229068 | TMPOP1 |
| ILF3 | ENSG00000103671 | TRIP4 |
| ILF3 | ENSG00000170191 | NANP |
| ILF3 | ENSG00000120057 | SFRP5 |
| ILF3 | ENSG00000279306 | RP5-915N17.11 |
| ILF3 | ENSG00000116791 | CRYZ |
| ILF3 | ENSG00000266075 | RN7SL574P |
| ILF3 | ENSG00000251314 | CTD- |
| ILF3 | ENSG00000168090 | 2337A12.1 COPS6 |
| ILF3 | ENSG00000198972 | MIRLET7E |
| ILF3 | ENSG00000235862 | RP11-338C15.5 |
| ILF3 | ENSG00000160963 | COL26A1 |
| ILF3 | ENSG00000120029 | C10orf76 |
| ILF3 | ENSG00000146410 | MTFR2 |
| ILF3 | ENSG00000227218 | RP11-203J24.8 |
| ILF3 | ENSG00000270195 | RP11-572O17.1 |
| ILF3 | ENSG00000110237 | ARHGEF17 |
| ILF3 | ENSG00000167720 | SRR |
| ILF3 | ENSG00000163041 | H3F3A |
| ILF3 | ENSG00000100023 | PPIL2 |
| ILF3 | ENSG00000170476 | MZB1 |
| ILF3 | ENSG00000188070 | C11orf95 |
| ILF3 | ENSG00000206815 | RNU6-483P |
| ILF3 | ENSG00000239775 | AC017116.11 |
| ILF3 | ENSG00000260272 | RP11-20I23.1 |
| ILF3 | ENSG00000176542 | KIAA2018 |
| ILF3 | ENSG00000237848 | RP11-739N20.3 |
| ILF3 | ENSG00000152219 | ARL14EP |
| ILF3 | ENSG00000172115 | CYCS |
| ILF3 | ENSG00000150977 | RILPL2 |
| ILF3 | ENSG00000137434 | C6orf52 |
| ILF3 | ENSG00000072858 | SIDT1 |
| ILF3 | ENSG00000083814 | ZNF671 |
| ILF3 | ENSG00000242170 | RN7SL329P |
| ILF3 | ENSG00000141295 | SCRN2 |
| ILF3 | ENSG00000202522 | Y_RNA |
| ILF3 | ENSG00000188352 | FOCAD |
| ILF3 | ENSG00000148690 | FRA10AC1 |
| ILF3 | ENSG00000266943 | RP11-15E18.2 |
| ILF3 | ENSG00000128266 | GNAZ |
| ILF3 | ENSG00000130935 | NOL11 |

| ILF3 | ENSG00000167930 | ITFG3 |
| --- | --- | --- |
| ILF3 | ENSG00000059728 | MXD1 |
| ILF3 | ENSG00000121653 | MAPK8IP1 |
| ILF3 | ENSG00000260659 | RP11-46C24.6 |
| ILF3 | ENSG00000099385 | BCL7C |
| ILF3 | ENSG00000276675 | TTC28-AS1_4 |
| ILF3 | ENSG00000257268 | RP1-74B13.2 |
| ILF3 | ENSG00000106077 | ABHD11 |
| ILF3 | ENSG00000119630 | PGF |
| ILF3 | ENSG00000238221 | RP11-69L16.4 |
| ILF3 | ENSG00000240219 | RP11-430C7.5 |
| ILF3 | ENSG00000199411 | SNORD62 |
| ILF3 | ENSG00000078596 | ITM2A |
| ILF3 | ENSG00000176871 | WSB2 |
| ILF3 | ENSG00000232645 | LINC01431 |
| ILF3 | ENSG00000230622 | UQCRHP1 |
| ILF3 | ENSG00000151164 | RAD9B |
| ILF3 | ENSG00000213440 | H2AFZP1 |
| ILF3 | ENSG00000270182 | RP1-170O19.24 |
| ILF3 | ENSG00000270264 | NDUFB8P2 |
| ILF3 | ENSG00000112685 | EXOC2 |
| ILF3 | ENSG00000115282 | TTC31 |
| ILF3 | ENSG00000200403 | RNU6-1099P |
| ILF3 | ENSG00000218300 | RP11-63K6.4 |
| ILF3 | ENSG00000142330 | CAPN10 |
| ILF3 | ENSG00000141298 | SSH2 |
| ILF3 | ENSG00000247011 | RP11-700H6.1 |
| ILF3 | ENSG00000151491 | EPS8 |
| ILF3 | ENSG00000181896 | ZNF101 |
| ILF3 | ENSG00000228149 | RPL3P1 |
| ILF3 | ENSG00000092036 | HAUS4 |
| ILF3 | ENSG00000230445 | LRRC37A6P |
| ILF3 | ENSG00000261113 | RP11-141O15.1 |
| ILF3 | ENSG00000139579 | NABP2 |
| ILF3 | ENSG00000166685 | COG1 |
| ILF3 | ENSG00000259322 | RP11-762H8.1 |
| ILF3 | ENSG00000260708 | CTA-29F11.1 |
| ILF3 | ENSG00000252677 | SNORA81 |
| ILF3 | ENSG00000160472 | TMEM190 |
| ILF3 | ENSG00000267952 | CTD- |
| ILF3 | ENSG00000124610 | 2207O23.12  HIST1H1A |
| ILF3 | ENSG00000090615 | GOLGA3 |
| ILF3 | ENSG00000162337 | LRP5 |
| ILF3 | ENSG00000274598 | RP11-423G4.10 |
| ILF3 | ENSG00000197283 | SYNGAP1 |
| ILF3 | ENSG00000120833 | SOCS2 |
| ILF3 | ENSG00000214417 | KRT18P13 |
| ILF3 | ENSG00000021300 | PLEKHB1 |
| ILF3 | ENSG00000001497 | LAS1L |
| ILF3 | ENSG00000232434 | C9orf172 |
| ILF3 | ENSG00000141030 | COPS3 |

| ILF3 | ENSG00000126709 | IFI6 |
| --- | --- | --- |
| ILF3 | ENSG00000186051 | TAL2 |
| ILF3 | ENSG00000131469 | RPL27 |
| ILF3 | ENSG00000212303 | RNU6-1154P |
| ILF3 | ENSG00000253250 | C8orf88 |
| ILF3 | ENSG00000184489 | PTP4A3 |
| ILF3 | ENSG00000186642 | PDE2A |
| ILF3 | ENSG00000273796 | LL21NC02- |
| ILF3 | ENSG00000214517 | 21A1.1  PPME1 |
| ILF3 | ENSG00000005100 | DHX33 |
| ILF3 | ENSG00000182511 | FES |
| ILF3 | ENSG00000144401 | METTL21A |
| ILF3 | ENSG00000223776 | LGALS8-AS1 |
| ILF3 | ENSG00000136634 | IL10 |
| ILF3 | ENSG00000172795 | DCP2 |
| ILF3 | ENSG00000125731 | SH2D3A |
| ILF3 | ENSG00000091527 | CDV3 |
| ILF3 | ENSG00000162959 | MEMO1 |
| ILF3 | ENSG00000254974 | RP11-702H23.2 |
| ILF3 | ENSG00000097046 | CDC7 |
| ILF3 | ENSG00000278274 | SNORA61 |
| ILF3 | ENSG00000279689 | RP11-574K11.32 |
| ILF3 | ENSG00000267206 | LCN6 |
| ILF3 | ENSG00000106384 | MOGAT3 |
| ILF3 | ENSG00000185220 | PGBD2 |
| ILF3 | ENSG00000235042 | AC098820.2 |
| ILF3 | ENSG00000168038 | ULK4 |
| ILF3 | ENSG00000076604 | TRAF4 |
| ILF3 | ENSG00000155561 | NUP205 |
| ILF3 | ENSG00000104957 | CCDC130 |
| ILF3 | ENSG00000139572 | GPR84 |
| ILF3 | ENSG00000269893 | SNHG8 |
| ILF3 | ENSG00000058335 | RASGRF1 |
| ILF3 | ENSG00000258914 | CTD-2134A5.3 |
| ILF3 | ENSG00000275833 | RP11-479O9.3 |
| ILF3 | ENSG00000108187 | PBLD |
| ILF3 | ENSG00000158109 | TPRG1L |
| ILF3 | ENSG00000197170 | PSMD12 |
| ILF3 | ENSG00000124942 | AHNAK |
| ILF3 | ENSG00000115486 | GGCX |
| ILF3 | ENSG00000260996 | RP13-122B23.8 |
| ILF3 | ENSG00000231724 | RP11-573D15.3 |
| ILF3 | ENSG00000116985 | BMP8B |
| ILF3 | ENSG00000138443 | ABI2 |
| ILF3 | ENSG00000172640 | OR10AD1 |
| ILF3 | ENSG00000277301 | RP5-1184F4.7 |
| ILF3 | ENSG00000204277 | RP11-219G17.4 |
| ILF3 | ENSG00000115904 | SOS1 |
| ILF3 | ENSG00000197548 | ATG7 |
| ILF3 | ENSG00000260173 | RP11-2I17.4 |
| ILF3 | ENSG00000199248 | RNU6-28P |

| ILF3 | ENSG00000197102 | DYNC1H1 |
| --- | --- | --- |
| ILF3 | ENSG00000148200 | NR6A1 |
| ILF3 | ENSG00000168275 | COA6 |
| ILF3 | ENSG00000100288 | CHKB |
| ILF3 | ENSG00000096717 | SIRT1 |
| ILF3 | ENSG00000188157 | AGRN |
| ILF3 | ENSG00000257342 | RP11-571M6.7 |
| ILF3 | ENSG00000111906 | HDDC2 |
| ILF3 | ENSG00000101104 | PABPC1L |
| ILF3 | ENSG00000198722 | UNC13B |
| ILF3 | ENSG00000111196 | MAGOHB |
| ILF3 | ENSG00000164744 | SUN3 |
| ILF3 | ENSG00000225929 | AC000036.4 |
| ILF3 | ENSG00000157538 | DSCR3 |
| ILF3 | ENSG00000102781 | KATNAL1 |
| ILF3 | ENSG00000130684 | ZNF337 |
| ILF3 | ENSG00000076242 | MLH1 |
| ILF3 | ENSG00000198604 | BAZ1A |
| ILF3 | ENSG00000120800 | UTP20 |
| ILF3 | ENSG00000128059 | PPAT |
| ILF3 | ENSG00000201487 | SNORD45B |
| ILF3 | ENSG00000168827 | GFM1 |
| ILF3 | ENSG00000261687 | RP11-473C18.3 |
| ILF3 | ENSG00000232037 | RP11-26H16.1 |
| ILF3 | ENSG00000204580 | DDR1 |
| ILF3 | ENSG00000189149 | CRYM-AS1 |
| ILF3 | ENSG00000146216 | TTBK1 |
| ILF3 | ENSG00000169139 | UBE2V2 |
| ILF3 | ENSG00000143570 | SLC39A1 |
| ILF3 | ENSG00000138821 | SLC39A8 |
| ILF3 | ENSG00000123144 | C19orf43 |
| ILF3 | ENSG00000177685 | CRACR2B |
| ILF3 | ENSG00000207101 | Y_RNA |
| ILF3 | ENSG00000196365 | LONP1 |
| ILF3 | ENSG00000272056 | RP11-503P10.1 |
| ILF3 | ENSG00000228943 | RP5-1053E7.2 |
| ILF3 | ENSG00000214770 | RP11-544I20.2 |
| ILF3 | ENSG00000234171 | RNASEH1-AS1 |
| ILF3 | ENSG00000137409 | MTCH1 |
| ILF3 | ENSG00000053501 | USE1 |
| ILF3 | ENSG00000147164 | SNX12 |
| ILF3 | ENSG00000265806 | MIR4292 |
| ILF3 | ENSG00000182405 | PGBD4 |
| ILF3 | ENSG00000197724 | PHF2 |
| ILF3 | ENSG00000163923 | RPL39L |
| ILF3 | ENSG00000271161 | RP11-708J19.2 |
| ILF3 | ENSG00000097096 | SYDE2 |
| ILF3 | ENSG00000222750 | RNU4-46P |
| ILF3 | ENSG00000083635 | NUFIP1 |
| ILF3 | ENSG00000265713 | RP11-82O19.2 |
| ILF3 | ENSG00000082781 | ITGB5 |

| ILF3 | ENSG00000077232 | DNAJC10 |
| --- | --- | --- |
| ILF3 | ENSG00000183508 | FAM46C |
| ILF3 | ENSG00000108176 | DNAJC12 |
| ILF3 | ENSG00000167637 | ZNF283 |
| ILF3 | ENSG00000235586 | AC011247.3 |
| ILF3 | ENSG00000128829 | EIF2AK4 |
| ILF3 | ENSG00000278816 | RP5-1116H23.4 |
| ILF3 | ENSG00000239722 | IMP3P2 |
| ILF3 | ENSG00000163875 | MEAF6 |
| ILF3 | ENSG00000135409 | AMHR2 |
| ILF3 | ENSG00000112146 | FBXO9 |
| ILF3 | ENSG00000279742 | RP11-700A24.1 |
| ILF3 | ENSG00000103855 | CD276 |
| ILF3 | ENSG00000083223 | ZCCHC6 |
| ILF3 | ENSG00000105642 | KCNN1 |
| ILF3 | ENSG00000121101 | TEX14 |
| ILF3 | ENSG00000199490 | Y_RNA |
| ILF3 | ENSG00000260267 | RP11-452L6.5 |
| ILF3 | ENSG00000123473 | STIL |
| ILF3 | ENSG00000176428 | VPS37D |
| ILF3 | ENSG00000278713 | CTD- |
| ILF3 | ENSG00000262500 | 2574D22.7 RP11-259G18.2 |
| ILF3 | ENSG00000223548 | AC034228.3 |
| ILF3 | ENSG00000237651 | C2orf74 |
| ILF3 | ENSG00000170092 | SPDYE5 |
| ILF3 | ENSG00000276867 | CTD- |
| ILF3 | ENSG00000085719 | 2358C21.5  CPNE3 |
| ILF3 | ENSG00000140395 | WDR61 |
| ILF3 | ENSG00000218499 | RP3-422G23.3 |
| ILF3 | ENSG00000116199 | FAM20B |
| ILF3 | ENSG00000062282 | DGAT2 |
| ILF3 | ENSG00000228137 | AP001469.7 |
| ILF3 | ENSG00000117139 | KDM5B |
| ILF3 | ENSG00000171611 | PTCRA |
| ILF3 | ENSG00000240531 | RPL21P123 |
| ILF3 | ENSG00000142864 | SERBP1 |
| ILF3 | ENSG00000124641 | MED20 |
| ILF3 | ENSG00000101442 | ACTR5 |
| ILF3 | ENSG00000088766 | CRLS1 |
| ILF3 | ENSG00000163527 | STT3B |
| ILF3 | ENSG00000115657 | ABCB6 |
| ILF3 | ENSG00000163848 | ZNF148 |
| ILF3 | ENSG00000213438 | YBX2P1 |
| ILF3 | ENSG00000106682 | EIF4H |
| ILF3 | ENSG00000109667 | SLC2A9 |
| ILF3 | ENSG00000090339 | ICAM1 |
| ILF3 | ENSG00000275180 | RP11-631N16.4 |
| ILF3 | ENSG00000167136 | ENDOG |
| ILF3 | ENSG00000266721 | MIR5695 |
| ILF3 | ENSG00000270698 | RP11-568J23.7 |

ILF3 ENSG00000279569 RP11-394B2.1

| ILF3 | ENSG00000142507 | PSMB6 |
| --- | --- | --- |
| ILF3 | ENSG00000263932 | MIR4448 |
| ILF3 | ENSG00000075292 | ZNF638 |
| ILF3 | ENSG00000276124 | MIR6855 |
| ILF3 | ENSG00000189362 | TMEM194B |
| ILF3 | ENSG00000261061 | RP11-303E16.2 |
| ILF3 | ENSG00000251866 | SCARNA21 |
| ILF3 | ENSG00000162522 | KIAA1522 |
| ILF3 | ENSG00000213402 | PTPRCAP |
| ILF3 | ENSG00000264834 | MIR1273F |
| ILF3 | ENSG00000258051 | RP11-474C8.3 |
| ILF3 | ENSG00000242612 | DECR2 |
| ILF3 | ENSG00000253738 | OTUD6B-AS1 |
| ILF3 | ENSG00000274492 | RP11-976B16.1 |
| ILF3 | ENSG00000130150 | MOSPD2 |
| ILF3 | ENSG00000011243 | AKAP8L |
| ILF3 | ENSG00000131944 | C19orf40 |
| ILF3 | ENSG00000254338 | MAFA-AS1 |
| ILF3 | ENSG00000114850 | SSR3 |
| ILF3 | ENSG00000070814 | TCOF1 |
| ILF3 | ENSG00000235106 | LINC00094 |
| ILF3 | ENSG00000249825 | CTD-2201I18.1 |
| ILF3 | ENSG00000266094 | RASSF5 |
| ILF3 | ENSG00000177192 | PUS1 |
| ILF3 | ENSG00000246283 | CTD-2036P10.3 |
| ILF3 | ENSG00000254999 | BRK1 |
| ILF3 | ENSG00000154639 | CXADR |
| ILF3 | ENSG00000232093 | RP11-307C12.11 |
| ILF3 | ENSG00000269560 | CTD-2192J16.21 |
| ILF3 | ENSG00000141956 | PRDM15 |
| ILF3 | ENSG00000174521 | TTC9B |
| ILF3 | ENSG00000135912 | TTLL4 |
| ILF3 | ENSG00000184270 | HIST2H2AB |
| ILF3 | ENSG00000221630 | MIR1179 |
| ILF3 | ENSG00000177728 | KIAA0195 |
| ILF3 | ENSG00000207569 | MIR433 |
| ILF3 | ENSG00000244265 | SIAH2-AS1 |
| ILF3 | ENSG00000183726 | TMEM50A |
| ILF3 | ENSG00000115641 | FHL2 |
| ILF3 | ENSG00000116273 | PHF13 |
| ILF3 | ENSG00000251900 | VTRNA2-2P |
| ILF3 | ENSG00000109133 | TMEM33 |
| ILF3 | ENSG00000269736 | CTD- |
| ILF3 | ENSG00000154845 | 2521M24.11  PPP4R1 |
| ILF3 | ENSG00000175536 | LIPT2 |
| ILF3 | ENSG00000137992 | DBT |
| ILF3 | ENSG00000137343 | ATAT1 |
| ILF3 | ENSG00000277084 | U2 |
| ILF3 | ENSG00000172426 | RSPH9 |
| ILF3 | ENSG00000198551 | ZNF627 |
| ILF3 | ENSG00000183943 | PRKX |

| ILF3 | ENSG00000124615 | MOCS1 |
| --- | --- | --- |
| ILF3 | ENSG00000175592 | FOSL1 |
| ILF3 | ENSG00000268465 | CTC-273B12.7 |
| ILF3 | ENSG00000186280 | KDM4D |
| ILF3 | ENSG00000259396 | RP11-16O9.2 |
| ILF3 | ENSG00000235332 | RP11-366O20.5 |
| ILF3 | ENSG00000243904 | RP11-600F24.1 |
| ILF3 | ENSG00000255362 | RP11-619A14.3 |
| ILF3 | ENSG00000185883 | ATP6V0C |
| ILF3 | ENSG00000199032 | MIR425 |
| ILF3 | ENSG00000240718 | RN7SL851P |
| ILF3 | ENSG00000125995 | ROMO1 |
| ILF3 | ENSG00000241357 | RP11-758P17.2 |
| ILF3 | ENSG00000224157 | HCG14 |
| ILF3 | ENSG00000136854 | STXBP1 |
| ILF3 | ENSG00000173762 | CD7 |
| ILF3 | ENSG00000135622 | SEMA4F |
| ILF3 | ENSG00000167508 | MVD |
| ILF3 | ENSG00000217442 | SYCE3 |
| ILF3 | ENSG00000164385 | C6orf195 |
| ILF3 | ENSG00000116237 | ICMT |
| ILF3 | ENSG00000127578 | WFIKKN1 |
| ILF3 | ENSG00000189403 | HMGB1 |
| ILF3 | ENSG00000157212 | PAXIP1 |
| ILF3 | ENSG00000159023 | EPB41 |
| ILF3 | ENSG00000167434 | CA4 |
| ILF3 | ENSG00000260095 | RP11-715J22.3 |
| ILF3 | ENSG00000229413 | RP11-274B21.12 |
| ILF3 | ENSG00000162927 | PUS10 |
| ILF3 | ENSG00000179580 | RNF151 |
| ILF3 | ENSG00000274582 | SNORA16A |
| ILF3 | ENSG00000122550 | KLHL7 |
| ILF3 | ENSG00000251022 | THAP9-AS1 |
| ILF3 | ENSG00000100982 | PCIF1 |
| ILF3 | ENSG00000146858 | ZC3HAV1L |
| ILF3 | ENSG00000181938 | GINS3 |
| ILF3 | ENSG00000108443 | RPS6KB1 |
| ILF3 | ENSG00000122674 | CCZ1 |
| ILF3 | ENSG00000260592 | CTA-363E6.6 |
| ILF3 | ENSG00000254770 | OR4D7P |
| ILF3 | ENSG00000105426 | PTPRS |
| ILF3 | ENSG00000167608 | TMC4 |
| ILF3 | ENSG00000166200 | COPS2 |
| ILF3 | ENSG00000204188 | GGNBP1 |
| ILF3 | ENSG00000240023 | RP11-561B11.1 |
| ILF3 | ENSG00000130414 | NDUFA10 |
| ILF3 | ENSG00000116001 | TIA1 |
| ILF3 | ENSG00000103932 | RPAP1 |
| ILF3 | ENSG00000140718 | FTO |
| ILF3 | ENSG00000168792 | ABHD15 |
| ILF3 | ENSG00000100312 | ACR |

| ILF3 | ENSG00000204624 | PTCHD2 |
| --- | --- | --- |
| ILF3 | ENSG00000151445 | VIPAS39 |
| ILF3 | ENSG00000127863 | TNFRSF19 |
| ILF3 | ENSG00000116957 | TBCE |
| ILF3 | ENSG00000277453 | CTC-492K19.7 |
| ILF3 | ENSG00000161021 | MAML1 |
| ILF3 | ENSG00000112159 | MDN1 |
| ILF3 | ENSG00000228335 | AC073063.10 |
| ILF3 | ENSG00000173465 | SSSCA1 |
| ILF3 | ENSG00000159239 | C2orf81 |
| ILF3 | ENSG00000111664 | GNB3 |
| ILF3 | ENSG00000250608 | RP11-933H2.4 |
| ILF3 | ENSG00000228800 | RP11-253D19.1 |
| ILF3 | ENSG00000163795 | ZNF513 |
| ILF3 | ENSG00000090554 | FLT3LG |
| ILF3 | ENSG00000102786 | INTS6 |
| ILF3 | ENSG00000213032 | NDUFA3P3 |
| ILF3 | ENSG00000151893 | CACUL1 |
| ILF3 | ENSG00000235081 | AC010492.2 |
| ILF3 | ENSG00000271868 | RP11-1293J14.1 |
| ILF3 | ENSG00000272954 | KB-1440D3.13 |
| ILF3 | ENSG00000168566 | SNRNP48 |
| ILF3 | ENSG00000186866 | POFUT2 |
| ILF3 | ENSG00000258555 | SPECC1L- |
| ILF3 | ENSG00000137713 | ADORA2A PPP2R1B |
| ILF3 | ENSG00000239195 | SNORD5 |
| ILF3 | ENSG00000253931 | RP11-909N17.2 |
| ILF3 | ENSG00000144045 | DQX1 |
| ILF3 | ENSG00000249471 | ZNF324B |
| ILF3 | ENSG00000156502 | SUPV3L1 |
| ILF3 | ENSG00000136888 | ATP6V1G1 |
| ILF3 | ENSG00000252228 | SNORA48 |
| ILF3 | ENSG00000197448 | GSTK1 |
| ILF3 | ENSG00000124356 | STAMBP |
| ILF3 | ENSG00000253399 | AC078852.2 |
| ILF3 | ENSG00000180304 | OAZ2 |
| ILF3 | ENSG00000277159 | RP11-88E10.4 |
| ILF3 | ENSG00000099904 | ZDHHC8 |
| ILF3 | ENSG00000168439 | STIP1 |
| ILF3 | ENSG00000163249 | CCNYL1 |
| ILF3 | ENSG00000168653 | NDUFS5 |
| ILF3 | ENSG00000239128 | snoU13 |
| ILF3 | ENSG00000090971 | NAT14 |
| ILF3 | ENSG00000152953 | STK32B |
| ILF3 | ENSG00000280228 | RP11-67L14.1 |
| ILF3 | ENSG00000253729 | PRKDC |
| ILF3 | ENSG00000197549 | PRAMENP |
| ILF3 | ENSG00000136444 | RSAD1 |
| ILF3 | ENSG00000132646 | PCNA |
| ILF3 | ENSG00000131408 | NR1H2 |
| ILF3 | ENSG00000226698 | RP1-50O24.6 |

| ILF3 | ENSG00000131368 | MRPS25 |
| --- | --- | --- |
| ILF3 | ENSG00000135525 | MAP7 |
| ILF3 | ENSG00000089356 | FXYD3 |
| ILF3 | ENSG00000268750 | CTD-2583A14.10 |
| ILF3 | ENSG00000182687 | GALR2 |
| ILF3 | ENSG00000139428 | MMAB |
| ILF3 | ENSG00000163902 | RPN1 |
| ILF3 | ENSG00000124160 | NCOA5 |
| ILF3 | ENSG00000065308 | TRAM2 |
| ILF3 | ENSG00000201302 | SNORA65 |
| ILF3 | ENSG00000137760 | ALKBH8 |
| ILF3 | ENSG00000154719 | MRPL39 |
| ILF3 | ENSG00000136167 | LCP1 |
| ILF3 | ENSG00000213240 | NOTCH2NL |
| ILF3 | ENSG00000205838 | TTC23L |
| ILF3 | ENSG00000274670 | RP13-820C6.4 |
| ILF3 | ENSG00000271361 | HTATSF1P2 |
| ILF3 | ENSG00000257815 | RP11-611E13.2 |
| ILF3 | ENSG00000165264 | NDUFB6 |
| ILF3 | ENSG00000269095 | AC010646.3 |
| ILF3 | ENSG00000105865 | DUS4L |
| ILF3 | ENSG00000172738 | TMEM217 |
| ILF3 | ENSG00000146463 | ZMYM4 |
| ILF3 | ENSG00000270068 | RP4-761J14.9 |
| ILF3 | ENSG00000146476 | C6orf211 |
| ILF3 | ENSG00000080546 | SESN1 |
| ILF3 | ENSG00000132664 | POLR3F |
| ILF3 | ENSG00000221978 | CCNL2 |
| ILF3 | ENSG00000251665 | RP11-700H6.2 |
| ILF3 | ENSG00000272630 | RP11-344N10.5 |
| ILF3 | ENSG00000184178 | SCFD2 |
| ILF3 | ENSG00000212102 | MIR301B |
| ILF3 | ENSG00000226912 | ISCA2P1 |
| ILF3 | ENSG00000176444 | CLK2 |
| ILF3 | ENSG00000049192 | ADAMTS6 |
| ILF3 | ENSG00000164236 | ANKRD33B |
| ILF3 | ENSG00000116675 | DNAJC6 |
| ILF3 | ENSG00000005238 | FAM214B |
| ILF3 | ENSG00000155508 | CNOT8 |
| ILF3 | ENSG00000121940 | CLCC1 |
| ILF3 | ENSG00000273544 | SNORA44 |
| ILF3 | ENSG00000131848 | ZSCAN5A |
| ILF3 | ENSG00000122481 | RWDD3 |
| ILF3 | ENSG00000101350 | KIF3B |
| ILF3 | ENSG00000117859 | OSBPL9 |
| ILF3 | ENSG00000115970 | THADA |
| ILF3 | ENSG00000173838 | 10-Mar |
| ILF3 | ENSG00000187867 | PALM3 |
| ILF3 | ENSG00000267288 | RP13-890H12.2 |
| ILF3 | ENSG00000263325 | LA16c-325D7.1 |
| ILF3 | ENSG00000106701 | FSD1L |

| ILF3 | ENSG00000163558 | PRKCI |
| --- | --- | --- |
| ILF3 | ENSG00000140830 | TXNL4B |
| ILF3 | ENSG00000172007 | RAB33B |
| ILF3 | ENSG00000199744 | SNORD36A |
| ILF3 | ENSG00000266978 | CTD-2369P2.5 |
| ILF3 | ENSG00000152270 | PDE3B |
| ILF3 | ENSG00000085662 | AKR1B1 |
| ILF3 | ENSG00000173141 | MRPL57 |
| ILF3 | ENSG00000126883 | NUP214 |
| ILF3 | ENSG00000175155 | YPEL2 |
| ILF3 | ENSG00000261442 | RP11-709D24.6 |
| ILF3 | ENSG00000134982 | APC |
| ILF3 | ENSG00000100036 | SLC35E4 |
| ILF3 | ENSG00000086598 | TMED2 |
| ILF3 | ENSG00000179403 | VWA1 |
| ILF3 | ENSG00000109762 | SNX25 |
| ILF3 | ENSG00000087157 | PGS1 |
| ILF3 | ENSG00000204256 | BRD2 |
| ILF3 | ENSG00000176927 | EFCAB5 |
| ILF3 | ENSG00000232953 | HSPA8P18 |
| ILF3 | ENSG00000188770 | OPTC |
| ILF3 | ENSG00000174013 | FBXO45 |
| ILF3 | ENSG00000228393 | LINC01004 |
| ILF3 | ENSG00000115207 | GTF3C2 |
| ILF3 | ENSG00000206811 | SNORA10 |
| ILF3 | ENSG00000171462 | DLK2 |
| ILF3 | ENSG00000106648 | GALNTL5 |
| ILF3 | ENSG00000188508 | KRTDAP |
| ILF3 | ENSG00000120688 | WBP4 |
| ILF3 | ENSG00000276645 | Metazoa_SRP |
| ILF3 | ENSG00000257730 | LSM6P2 |
| ILF3 | ENSG00000121104 | FAM117A |
| ILF3 | ENSG00000113368 | LMNB1 |
| ILF3 | ENSG00000166004 | CEP295 |
| ILF3 | ENSG00000123329 | ARHGAP9 |
| ILF3 | ENSG00000274922 | RP11-88E10.5 |
| ILF3 | ENSG00000261949 | GFY |
| ILF3 | ENSG00000100601 | ALKBH1 |
| ILF3 | ENSG00000115840 | SLC25A12 |
| ILF3 | ENSG00000196730 | DAPK1 |
| ILF3 | ENSG00000250476 | ENPP7P9 |
| ILF3 | ENSG00000175229 | GAL3ST3 |
| ILF3 | ENSG00000179163 | FUCA1 |
| ILF3 | ENSG00000224281 | SLC25A5-AS1 |
| ILF3 | ENSG00000267216 | AC010642.1 |
| ILF3 | ENSG00000162543 | UBXN10 |
| ILF3 | ENSG00000126858 | RHOT1 |
| ILF3 | ENSG00000259840 | LA16c-380A1.1 |
| ILF3 | ENSG00000182359 | KBTBD3 |
| ILF3 | ENSG00000198673 | FAM19A2 |
| ILF3 | ENSG00000237655 | AC073834.3 |

| ILF3 | ENSG00000204950 | LRRC10B |
| --- | --- | --- |
| ILF3 | ENSG00000276523 | RP11-490B18.6 |
| ILF3 | ENSG00000122786 | CALD1 |
| ILF3 | ENSG00000257386 | RP11-56G10.2 |
| ILF3 | ENSG00000124232 | RBPJL |
| ILF3 | ENSG00000141696 | LEPREL4 |
| ILF3 | ENSG00000205269 | TMEM170B |
| ILF3 | ENSG00000145715 | RASA1 |
| ILF3 | ENSG00000126067 | PSMB2 |
| ILF3 | ENSG00000174572 | RP11-209A2.1 |
| ILF3 | ENSG00000229325 | ACAP2-IT1 |
| ILF3 | ENSG00000015475 | BID |
| ILF3 | ENSG00000214113 | LYRM4 |
| ILF3 | ENSG00000139410 | SDSL |
| ILF3 | ENSG00000277249 | MIR6784 |
| ILF3 | ENSG00000121690 | DEPDC7 |
| ILF3 | ENSG00000127530 | OR7C1 |
| ILF3 | ENSG00000200345 | RNU6-485P |
| ILF3 | ENSG00000186283 | TOR3A |
| ILF3 | ENSG00000274259 | XXbac- |
| ILF3 | ENSG00000174851 | BPG294E21.9  YIF1A |
| ILF3 | ENSG00000225377 | NRSN2-AS1 |
| ILF3 | ENSG00000152601 | MBNL1 |
| ILF3 | ENSG00000143612 | C1orf43 |
| ILF3 | ENSG00000270453 | CTB-10G5.1 |
| ILF3 | ENSG00000157368 | IL34 |
| ILF3 | ENSG00000176912 | TYMSOS |
| ILF3 | ENSG00000266531 | MIR4706 |
| ILF3 | ENSG00000236297 | RP11-175P19.2 |
| ILF3 | ENSG00000135940 | COX5B |
| ILF3 | ENSG00000142512 | SIGLEC10 |
| ILF3 | ENSG00000240970 | RPL23AP64 |
| ILF3 | ENSG00000175066 | GK5 |
| ILF3 | ENSG00000171428 | NAT1 |
| ILF3 | ENSG00000248405 | PRR5-ARHGAP8 |
| ILF3 | ENSG00000105221 | AKT2 |
| ILF3 | ENSG00000279744 | RP13-20L14.10 |
| ILF3 | ENSG00000261359 | PYCARD-AS1 |
| ILF3 | ENSG00000221968 | FADS3 |
| ILF3 | ENSG00000107951 | MTPAP |
| ILF3 | ENSG00000161542 | PRPSAP1 |
| ILF3 | ENSG00000089327 | FXYD5 |
| ILF3 | ENSG00000263069 | CTD-2047H16.4 |
| ILF3 | ENSG00000124222 | STX16 |
| ILF3 | ENSG00000272968 | RBAK-RBAKDN |
| ILF3 | ENSG00000103479 | RBL2 |
| ILF3 | ENSG00000228594 | C1orf233 |
| ILF3 | ENSG00000100802 | C14orf93 |
| ILF3 | ENSG00000118898 | PPL |
| ILF3 | ENSG00000235919 | ASH1L-AS1 |
| ILF3 | ENSG00000106397 | PLOD3 |

| ILF3 | ENSG00000258908 | RP11-203M5.8 |
| --- | --- | --- |
| ILF3 | ENSG00000104343 | UBE2W |
| ILF3 | ENSG00000167194 | C16orf92 |
| ILF3 | ENSG00000142065 | ZFP14 |
| ILF3 | ENSG00000048991 | R3HDM1 |
| ILF3 | ENSG00000081177 | EXD2 |
| ILF3 | ENSG00000266101 | RP5-906A24.2 |
| ILF3 | ENSG00000268157 | AC010524.4 |
| ILF3 | ENSG00000176563 | CNTD1 |
| ILF3 | ENSG00000231057 | RP11-122M14.1 |
| ILF3 | ENSG00000087237 | CETP |
| ILF3 | ENSG00000201529 | Y_RNA |
| ILF3 | ENSG00000215559 | ANKRD20A11 |
| ILF3 | ENSG00000256658 | P  RP11-180M15.4 |
| ILF3 | ENSG00000092871 | RFFL |
| ILF3 | ENSG00000159905 | ZNF221 |
| ILF3 | ENSG00000264575 | LINC00526 |
| ILF3 | ENSG00000229750 | AC096649.2 |
| ILF3 | ENSG00000272589 | ZSWIM8-AS1 |
| ILF3 | ENSG00000261959 | RP11-893F2.14 |
| ILF3 | ENSG00000101773 | RBBP8 |
| ILF3 | ENSG00000163815 | CLEC3B |
| ILF3 | ENSG00000278007 | Metazoa_SRP |
| ILF3 | ENSG00000138101 | DTNB |
| ILF3 | ENSG00000115756 | HPCAL1 |
| ILF3 | ENSG00000203812 | HIST2H2AA4 |
| ILF3 | ENSG00000177853 | ZNF518A |
| ILF3 | ENSG00000100403 | ZC3H7B |
| ILF3 | ENSG00000269680 | CTD- |
| ILF3 | ENSG00000175514 | 3128G10.6 GPR152 |
| ILF3 | ENSG00000229178 | AC069513.4 |
| ILF3 | ENSG00000230082 | PRRT3-AS1 |
| ILF3 | ENSG00000188428 | BLOC1S5 |
| ILF3 | ENSG00000272662 | RP11-190C22.8 |
| ILF3 | ENSG00000196367 | TRRAP |
| ILF3 | ENSG00000270739 | RP11-608O8.2 |
| ILF3 | ENSG00000198829 | SUCNR1 |
| ILF3 | ENSG00000222041 | LINC00152 |
| ILF3 | ENSG00000120656 | TAF12 |
| ILF3 | ENSG00000170325 | PRDM10 |
| ILF3 | ENSG00000231690 | LINC00574 |
| ILF3 | ENSG00000179603 | GRM8 |
| ILF3 | ENSG00000163104 | SMARCAD1 |
| ILF3 | ENSG00000120696 | KBTBD7 |
| ILF3 | ENSG00000076108 | BAZ2A |
| ILF3 | ENSG00000125618 | PAX8 |
| ILF3 | ENSG00000260261 | RP11-480A16.1 |
| ILF3 | ENSG00000187595 | ZNF385C |
| ILF3 | ENSG00000104884 | ERCC2 |
| ILF3 | ENSG00000095261 | PSMD5 |

ILF3 ENSG00000066697 MSANTD3

| ILF3 | ENSG00000168803 | ADAL |
| --- | --- | --- |
| ILF3 | ENSG00000159788 | RGS12 |
| ILF3 | ENSG00000278683 | RP11-132A1.6 |
| ILF3 | ENSG00000213762 | ZNF134 |
| ILF3 | ENSG00000233527 | ZNF529-AS1 |
| ILF3 | ENSG00000076258 | FMO4 |
| ILF3 | ENSG00000247796 | CTD-2366F13.1 |
| ILF3 | ENSG00000223398 | AC027124.2 |
| ILF3 | ENSG00000253258 | RP11-539E17.5 |
| ILF3 | ENSG00000147044 | CASK |
| ILF3 | ENSG00000124688 | MAD2L1BP |
| ILF3 | ENSG00000221719 | SNORA3 |
| ILF3 | ENSG00000238363 | SNORA13 |
| ILF3 | ENSG00000205250 | E2F4 |
| ILF3 | ENSG00000243018 | RP5-1070G24.2 |
| ILF3 | ENSG00000272182 | RP11-802O23.3 |
| ILF3 | ENSG00000178996 | SNX18 |
| ILF3 | ENSG00000013364 | MVP |
| ILF3 | ENSG00000263342 | RP1-4G17.2 |
| ILF3 | ENSG00000107159 | CA9 |
| ILF3 | ENSG00000196396 | PTPN1 |
| ILF3 | ENSG00000156482 | RPL30 |
| ILF3 | ENSG00000213943 | KRT18P17 |
| ILF3 | ENSG00000213928 | IRF9 |
| ILF3 | ENSG00000243156 | MICAL3 |
| ILF3 | ENSG00000207305 | Y_RNA |
| ILF3 | ENSG00000135596 | MICAL1 |
| ILF3 | ENSG00000214106 | PAXIP1-AS2 |
| ILF3 | ENSG00000232880 | RP3-453C12.8 |
| ILF3 | ENSG00000179833 | SERTAD2 |
| ILF3 | ENSG00000274072 | mascRNA- |
| ILF3 | ENSG00000124702 | menRNA  KLHDC3 |
| ILF3 | ENSG00000215041 | NEURL4 |
| ILF3 | ENSG00000136754 | ABI1 |
| ILF3 | ENSG00000168172 | HOOK3 |
| ILF3 | ENSG00000185900 | POMK |
| ILF3 | ENSG00000028137 | TNFRSF1B |
| ILF3 | ENSG00000105696 | TMEM59L |
| ILF3 | ENSG00000265333 | MIR3137 |
| ILF3 | ENSG00000275072 | SNORD50B |
| ILF3 | ENSG00000145022 | TCTA |
| ILF3 | ENSG00000126882 | FAM78A |
| ILF3 | ENSG00000077684 | JADE1 |
| ILF3 | ENSG00000087087 | SRRT |
| ILF3 | ENSG00000122557 | HERPUD2 |
| ILF3 | ENSG00000123607 | TTC21B |
| ILF3 | ENSG00000117691 | NENF |
| ILF3 | ENSG00000188277 | C15orf62 |
| ILF3 | ENSG00000128609 | NDUFA5 |
| ILF3 | ENSG00000160766 | GBAP1 |
| ILF3 | ENSG00000250634 | LINC01182 |

| ILF3 | ENSG00000259780 | RP11-304L19.12 |
| --- | --- | --- |
| ILF3 | ENSG00000216775 | RP1-152L7.5 |
| ILF3 | ENSG00000071967 | CYBRD1 |
| ILF3 | ENSG00000241627 | UBQLN4P1 |
| ILF3 | ENSG00000166189 | HPS6 |
| ILF3 | ENSG00000271109 | CTC-523E23.11 |
| ILF3 | ENSG00000131061 | ZNF341 |
| ILF3 | ENSG00000151779 | NBAS |
| ILF3 | ENSG00000087076 | HSD17B14 |
| ILF3 | ENSG00000215039 | CD27-AS1 |
| ILF3 | ENSG00000105327 | BBC3 |
| ILF3 | ENSG00000125459 | MSTO1 |
| ILF3 | ENSG00000270914 | RP5-1077B9.5 |
| ILF3 | ENSG00000175224 | ATG13 |
| ILF3 | ENSG00000260304 | RP11-388M20.6 |
| ILF3 | ENSG00000115297 | TLX2 |
| ILF3 | ENSG00000234233 | KCNH1-IT1 |
| ILF3 | ENSG00000205758 | CRYZL1 |
| ILF3 | ENSG00000263800 | MIR5684 |
| ILF3 | ENSG00000072422 | RHOBTB1 |
| ILF3 | ENSG00000258515 | RP11-203M5.7 |
| ILF3 | ENSG00000266445 | RP13-991F5.2 |
| ILF3 | ENSG00000228951 | RP11-336A10.4 |
| ILF3 | ENSG00000184274 | LINC00315 |
| ILF3 | ENSG00000259171 | RNASE4 |
| ILF3 | ENSG00000252128 | SNORD27 |
| ILF3 | ENSG00000172425 | TTC36 |
| ILF3 | ENSG00000008018 | PSMB1 |
| ILF3 | ENSG00000241129 | RPL22P19 |
| ILF3 | ENSG00000152147 | GEMIN6 |
| ILF3 | ENSG00000275559 | DLEU2_6 |
| ILF3 | ENSG00000143643 | TTC13 |
| ILF3 | ENSG00000253304 | TMEM200B |
| ILF3 | ENSG00000266498 | RP11-45M22.5 |
| ILF3 | ENSG00000132879 | FBXO44 |
| ILF3 | ENSG00000096060 | FKBP5 |
| ILF3 | ENSG00000221829 | FANCG |
| ILF3 | ENSG00000196296 | ATP2A1 |
| ILF3 | ENSG00000215006 | CHCHD2P2 |
| ILF3 | ENSG00000176401 | EID2B |
| ILF3 | ENSG00000141837 | CACNA1A |
| ILF3 | ENSG00000100445 | SDR39U1 |
| ILF3 | ENSG00000253720 | RP11-473O4.3 |
| ILF3 | ENSG00000144524 | COPS7B |
| ILF3 | ENSG00000172840 | PDP2 |
| ILF3 | ENSG00000182093 | WRB |
| ILF3 | ENSG00000152484 | USP12 |
| ILF3 | ENSG00000135297 | MTO1 |
| ILF3 | ENSG00000164073 | MFSD8 |
| ILF3 | ENSG00000069329 | VPS35 |
| ILF3 | ENSG00000275818 | MIR6818 |

| ILF3 | ENSG00000143258 | USP21 |
| --- | --- | --- |
| ILF3 | ENSG00000187122 | SLIT1 |
| ILF3 | ENSG00000250600 | ROPN1L-AS1 |
| ILF3 | ENSG00000231494 | AC104634.3 |
| ILF3 | ENSG00000260468 | LINC01290 |
| ILF3 | ENSG00000196155 | PLEKHG4 |
| ILF3 | ENSG00000249286 | CTD-2210P15.2 |
| ILF3 | ENSG00000166323 | C11orf65 |
| ILF3 | ENSG00000110315 | RNF141 |
| ILF3 | ENSG00000101972 | STAG2 |
| ILF3 | ENSG00000134698 | AGO4 |
| ILF3 | ENSG00000196459 | TRAPPC2 |
| ILF3 | ENSG00000106686 | SPATA6L |
| ILF3 | ENSG00000136940 | PDCL |
| ILF3 | ENSG00000186312 | CA5BP1 |
| ILF3 | ENSG00000144747 | TMF1 |
| ILF3 | ENSG00000168216 | LMBRD1 |
| ILF3 | ENSG00000240180 | RP11-318E3.4 |
[truncated: 326,476 more chars]
