# Supplementary material for: Simvastatin inhibits PD-L1 via ILF3 to induce ferroptosis in gastric cancer cells
Source: Cell Death Dis. 2025 Mar 26;16(1):208. doi: 10.1038/s41419-025-07562-8 (PMC11947124; doi:10.1038/s41419-025-07562-8)
Supplement: Supplementary file 2 — Original western blot [file 41419_2025_7562_MOESM2_ESM.docx]

Figure1E:

ILF3:


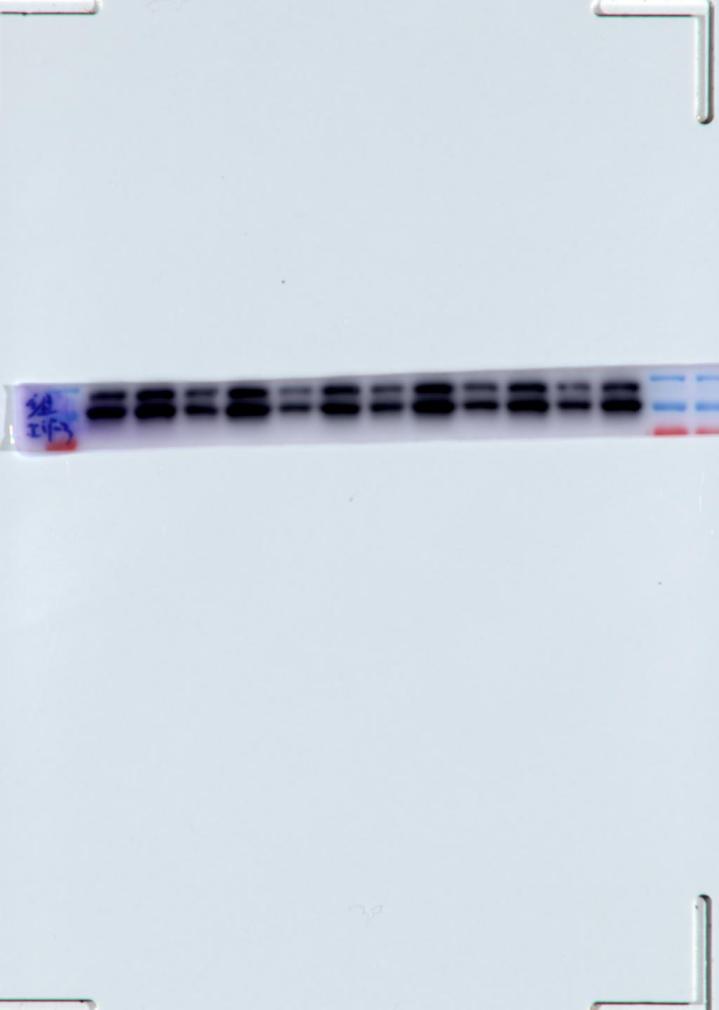


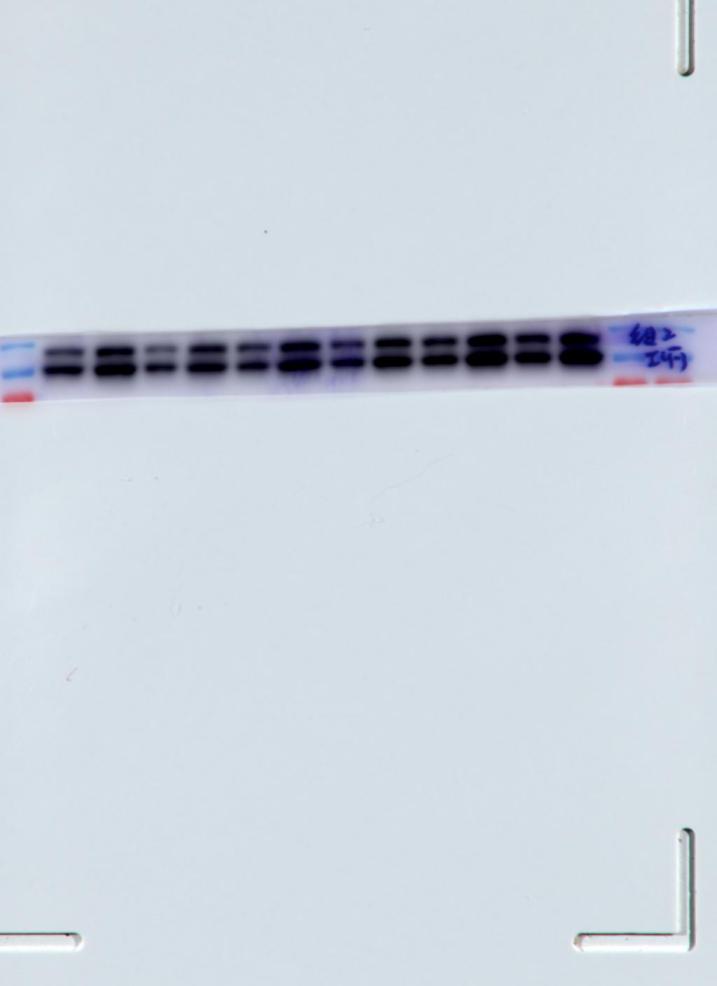


GAPDH:


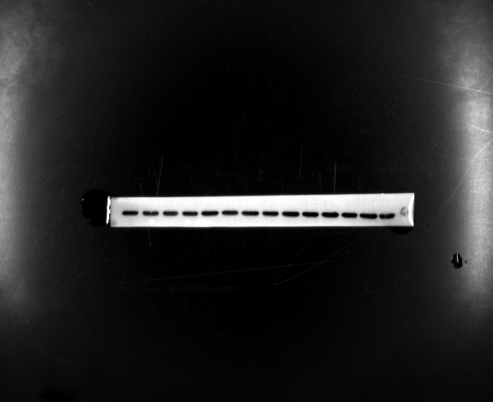


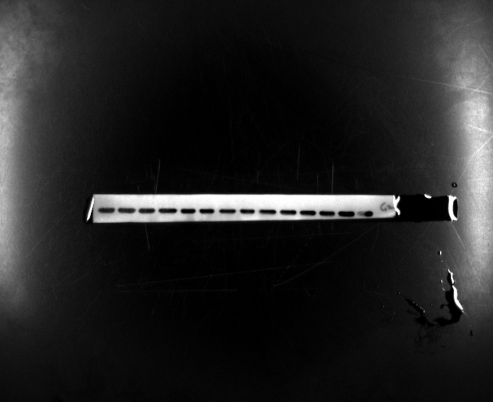


Figure2F:

ILF3:


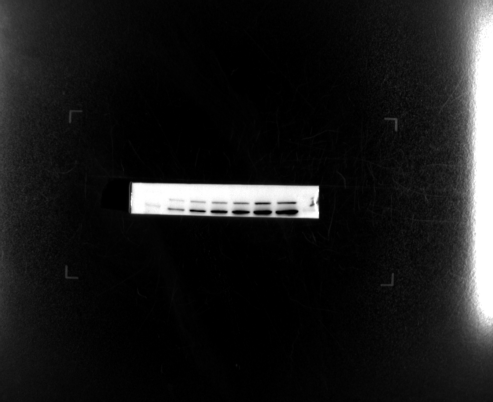


GAPDH:


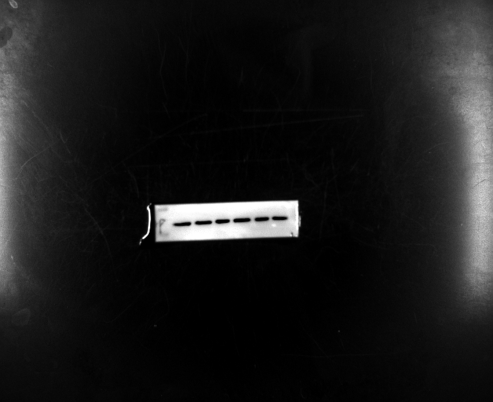


G: ILF3:


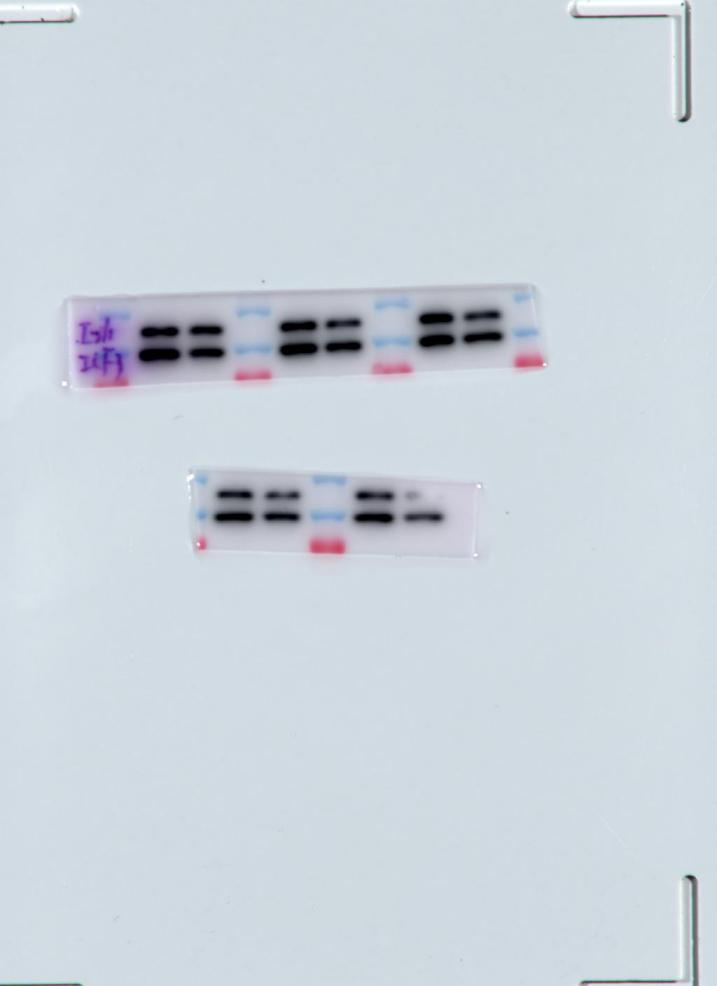


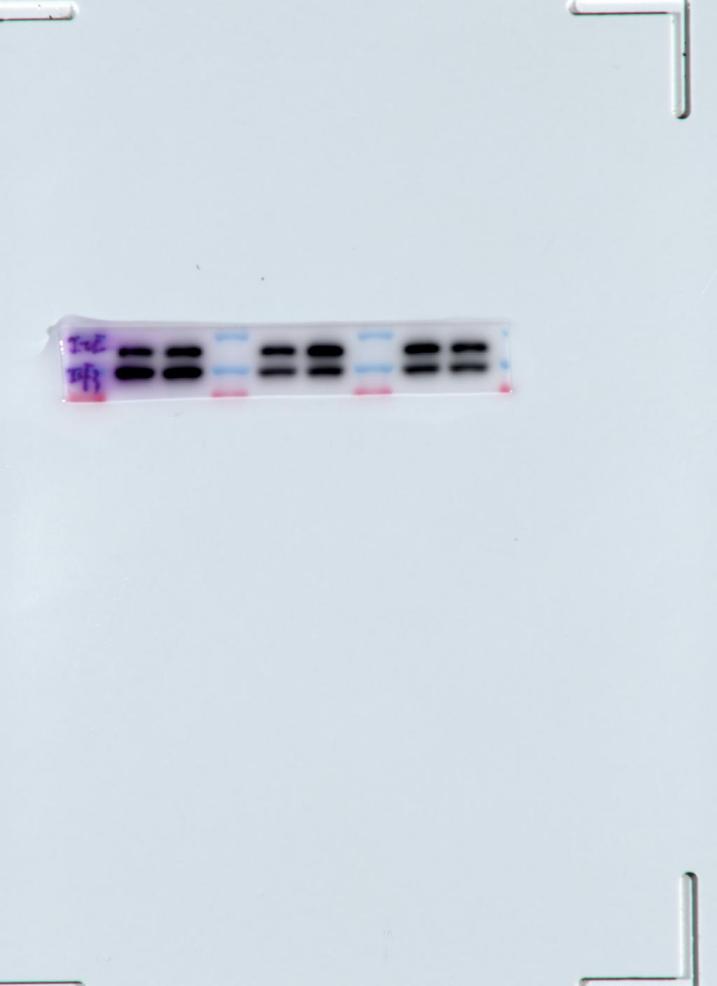

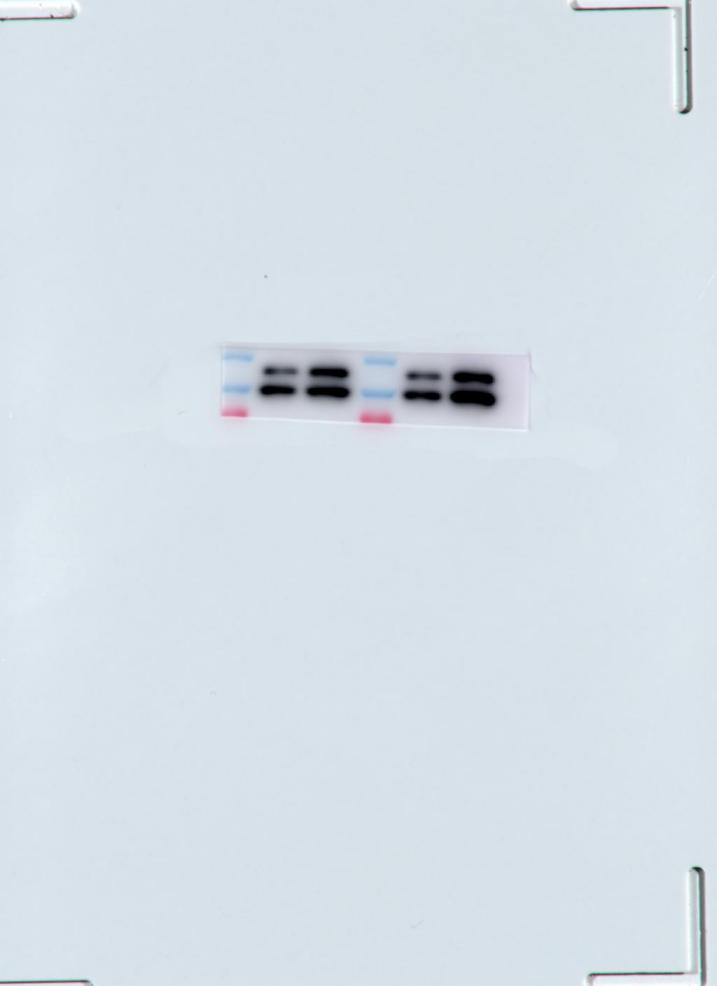


PD-L1:


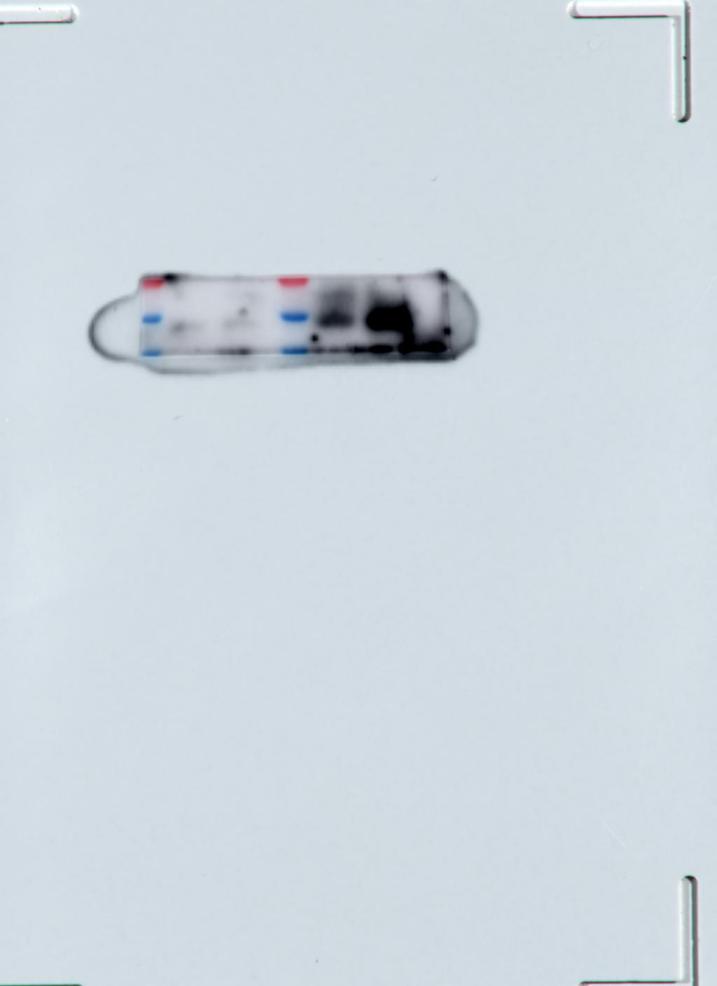


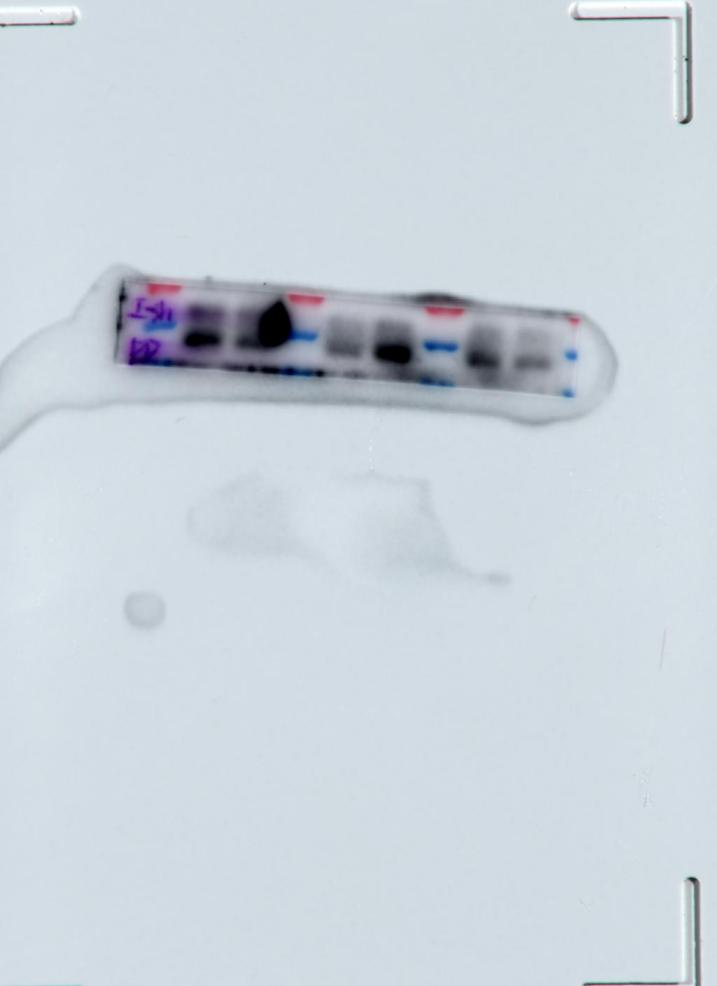


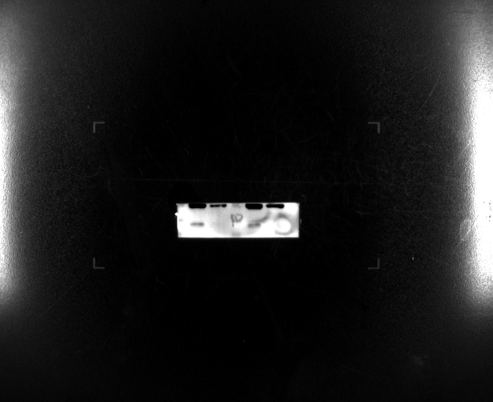


GAPDH:


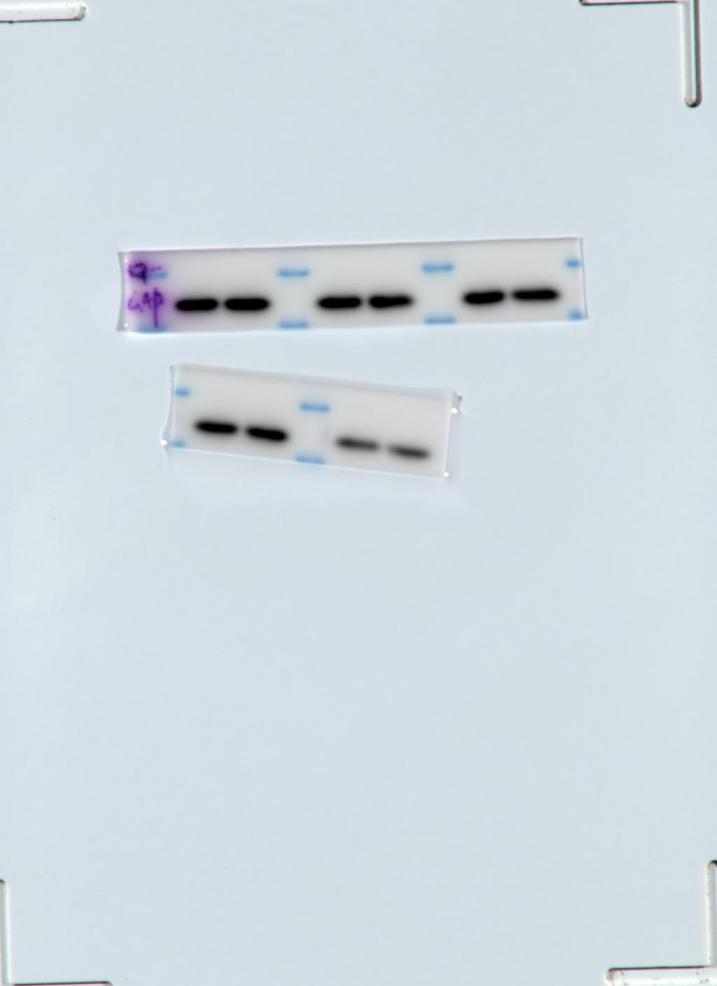


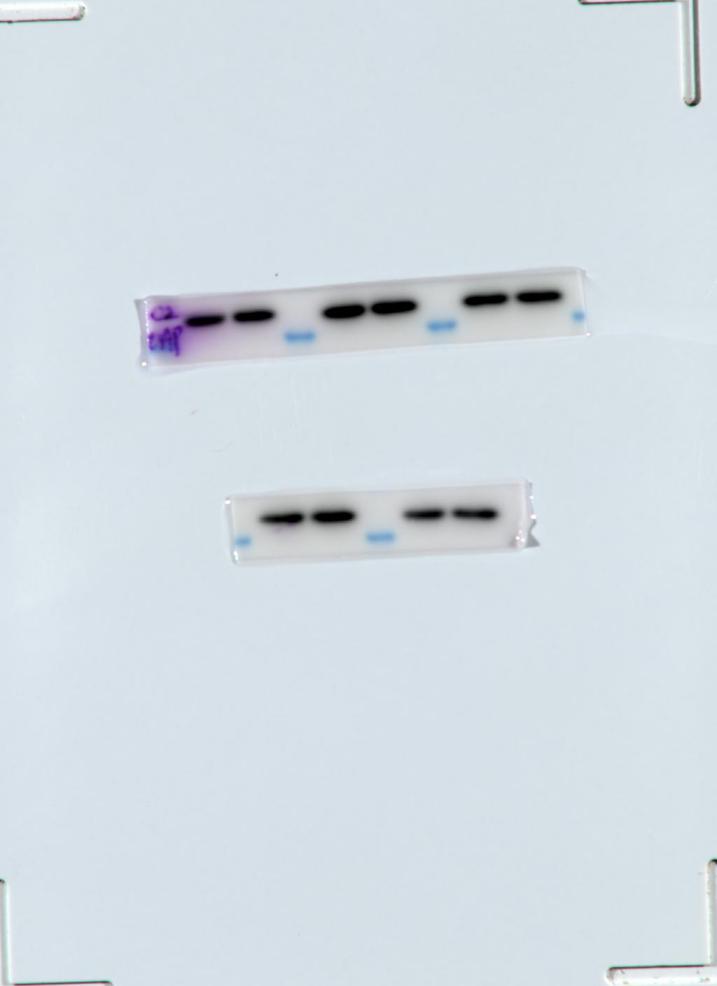


Figure3A:

ILF3:


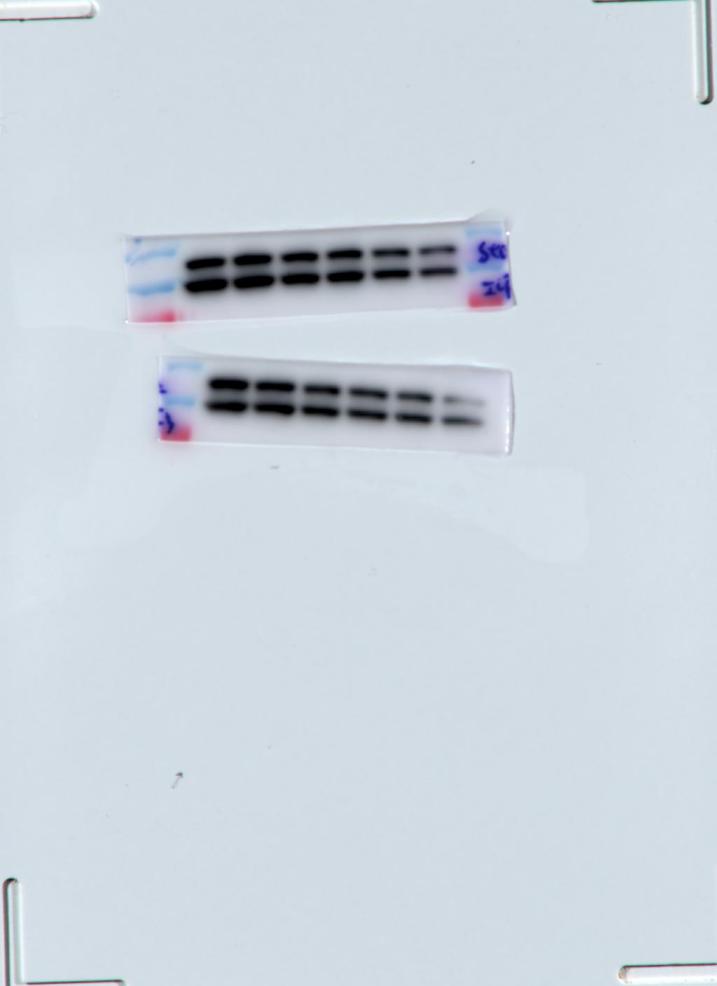


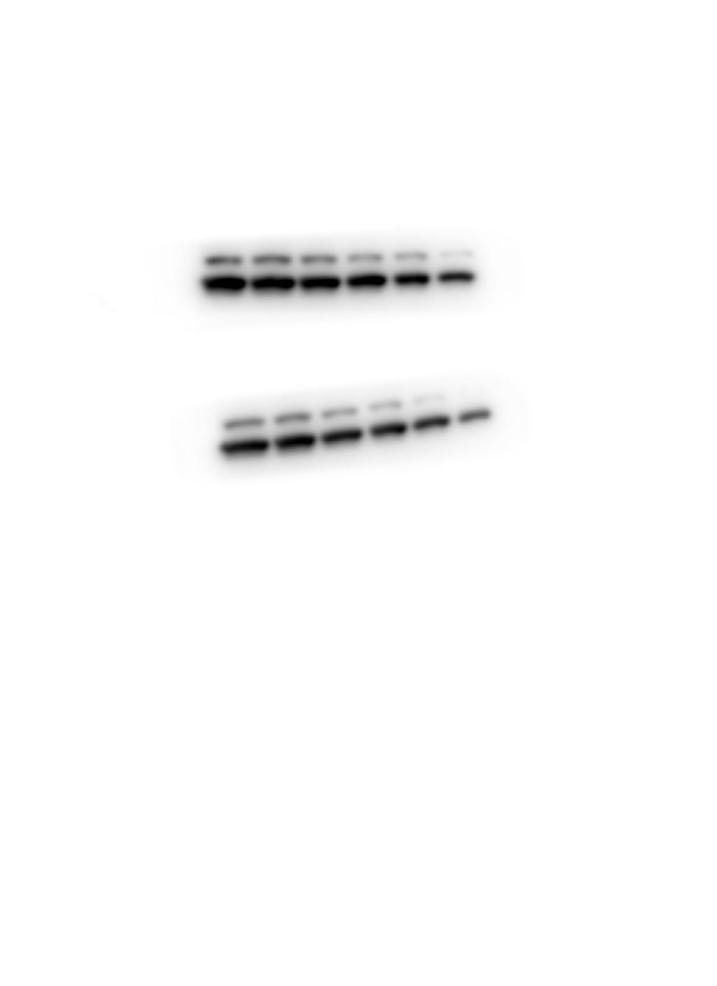


GAPDH：


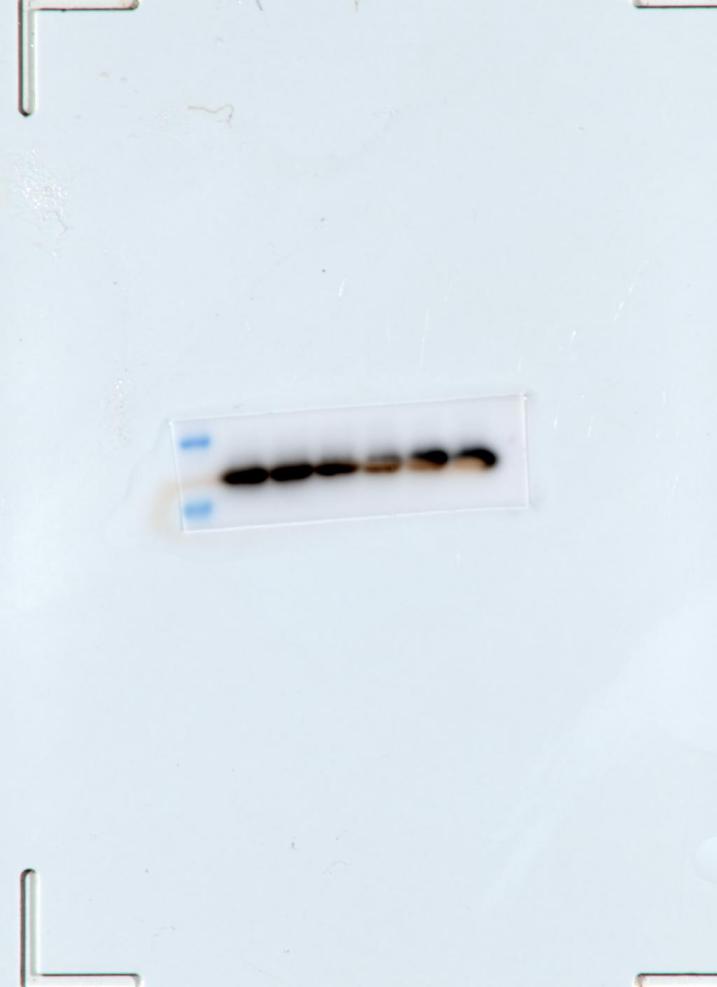


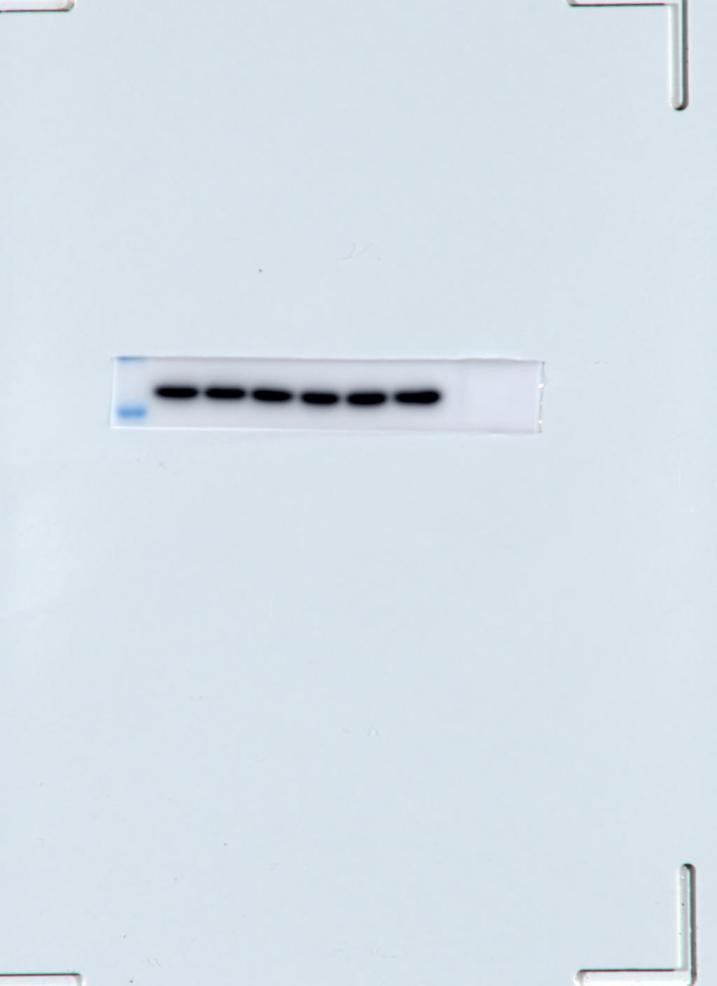


Figure3F:

ILF3：


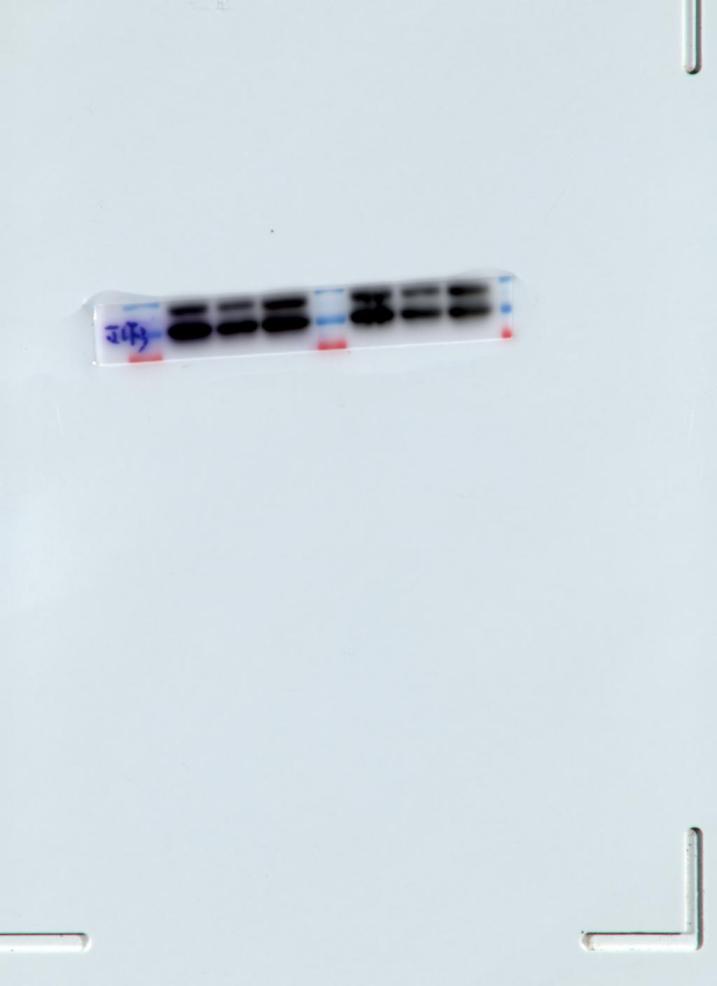

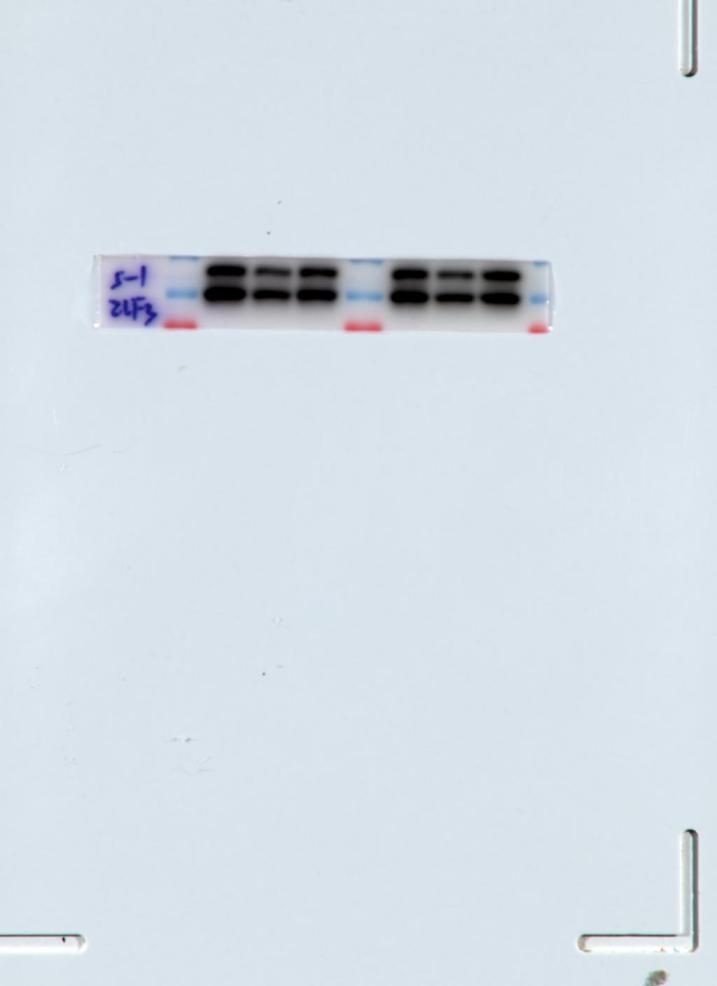


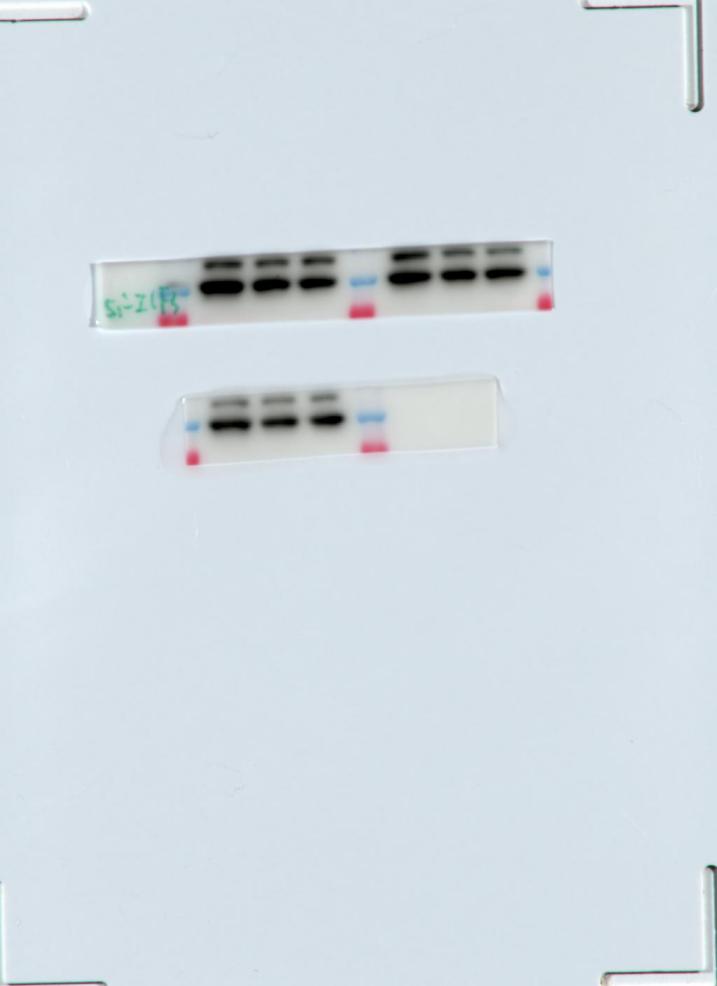


SLC7A11：


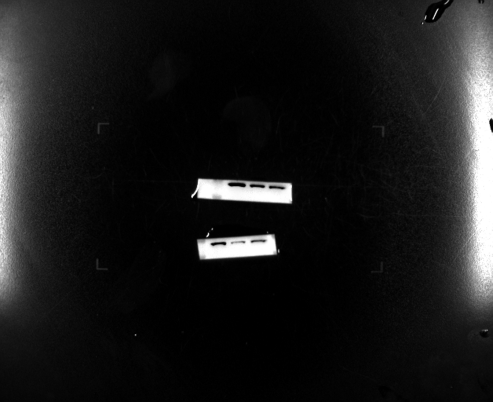


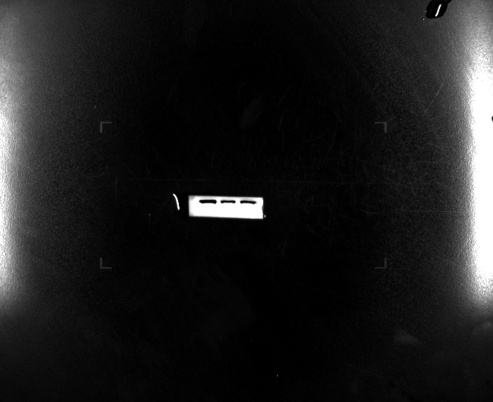


GPX4：


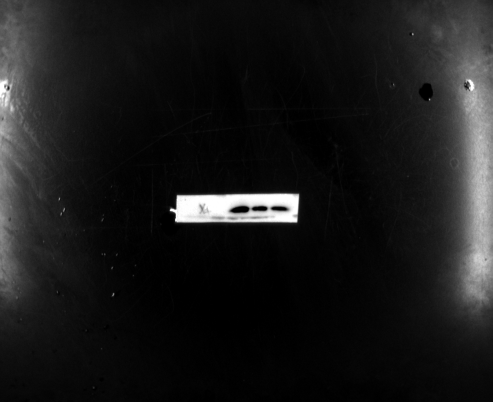


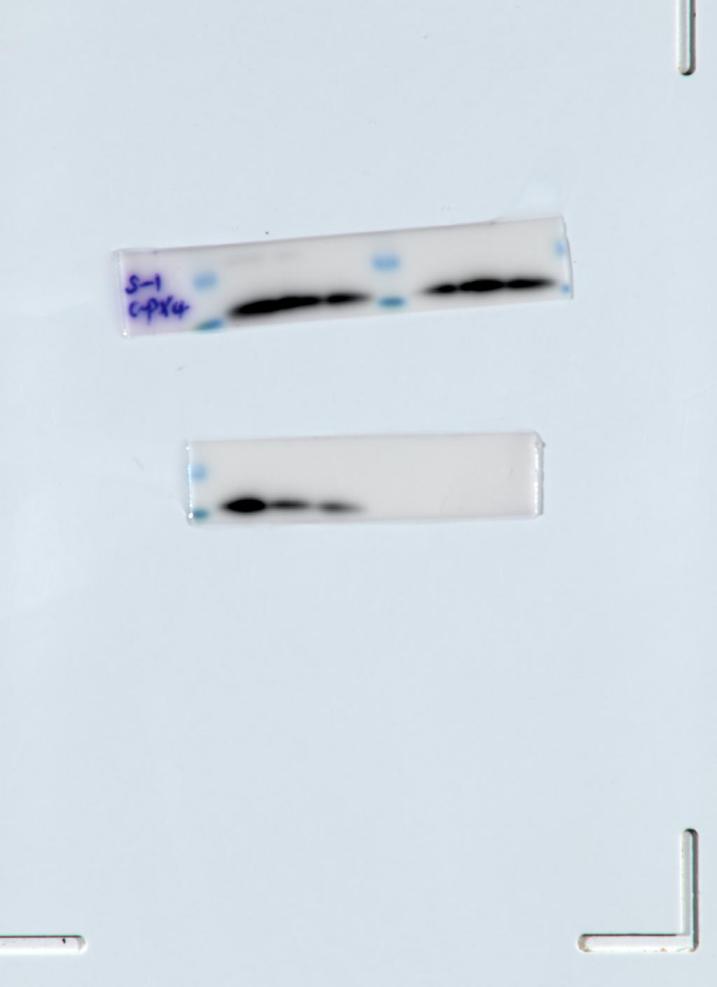


GAPDH：


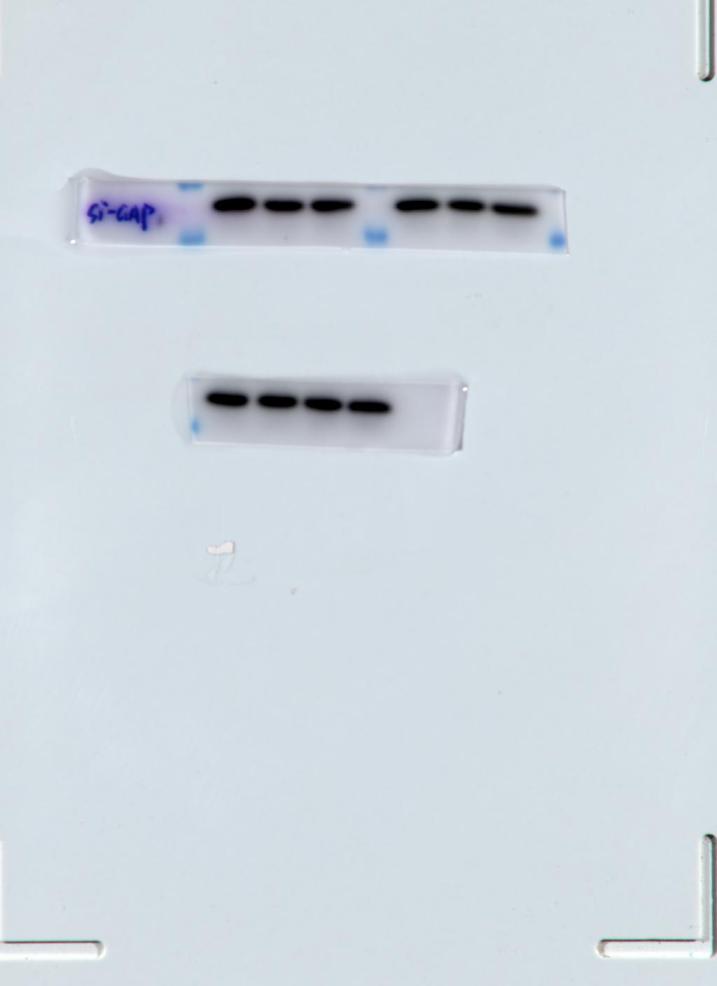


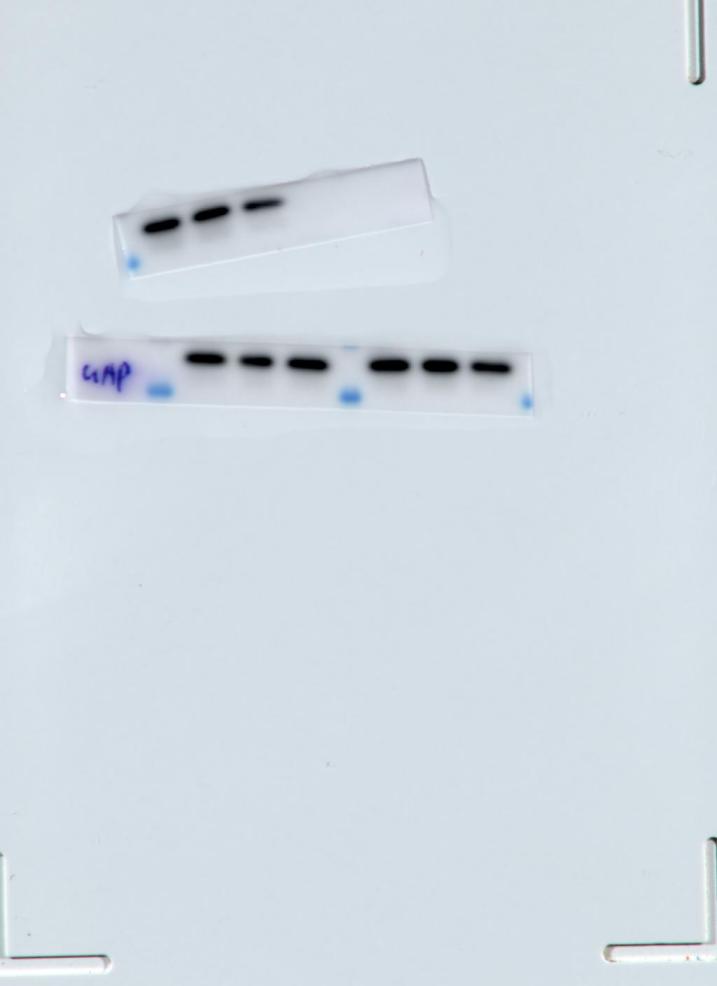


Figure 4

A:PD-L1:


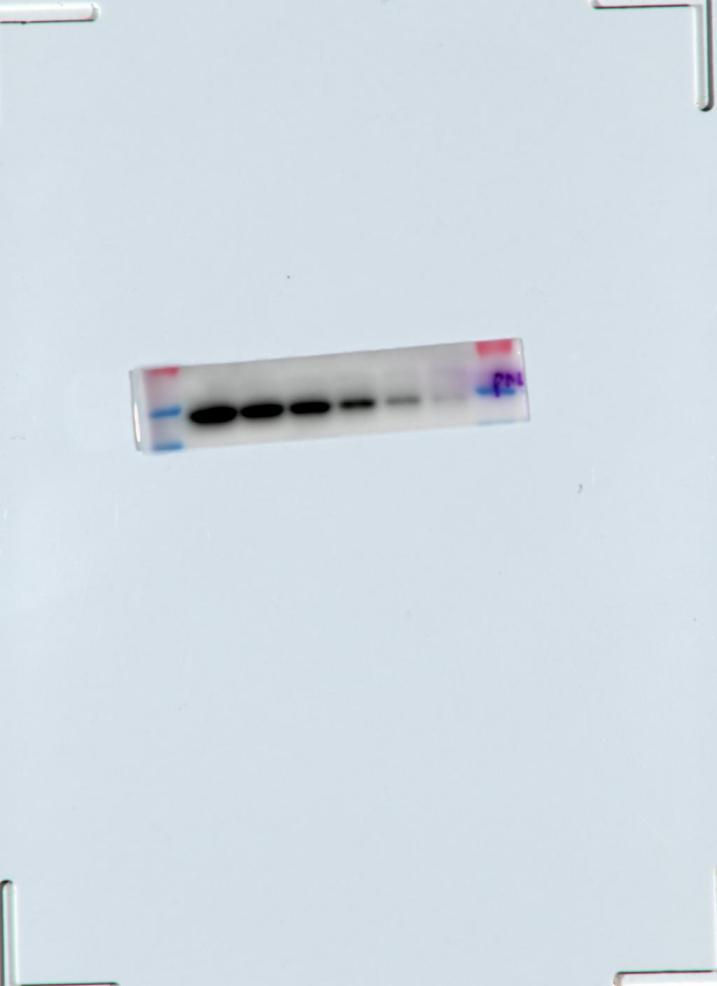

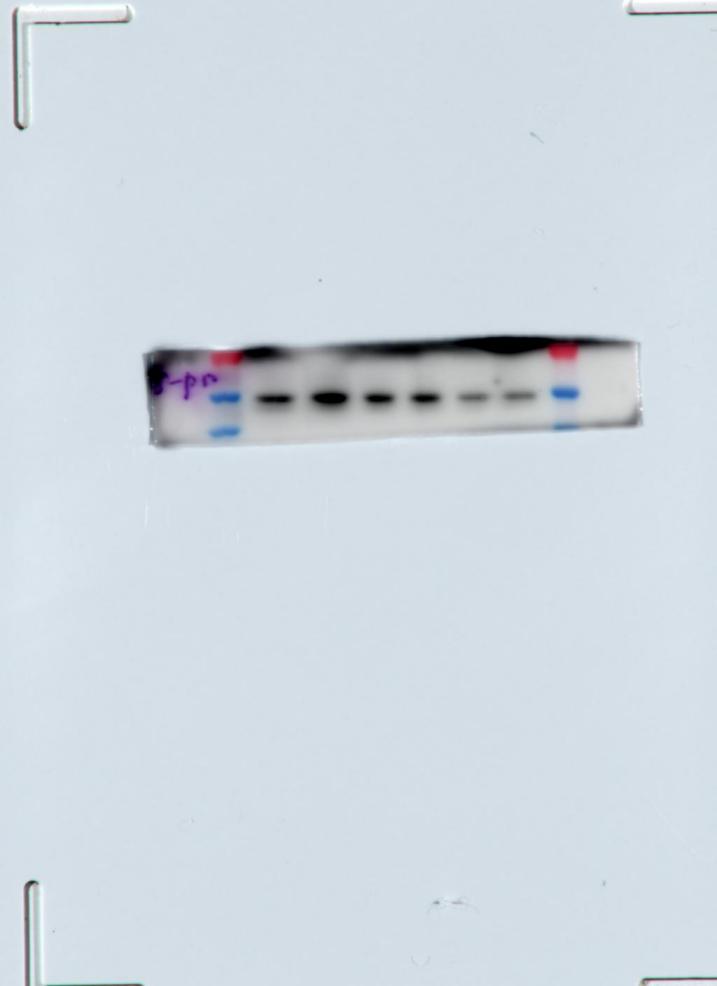


GAPDH:


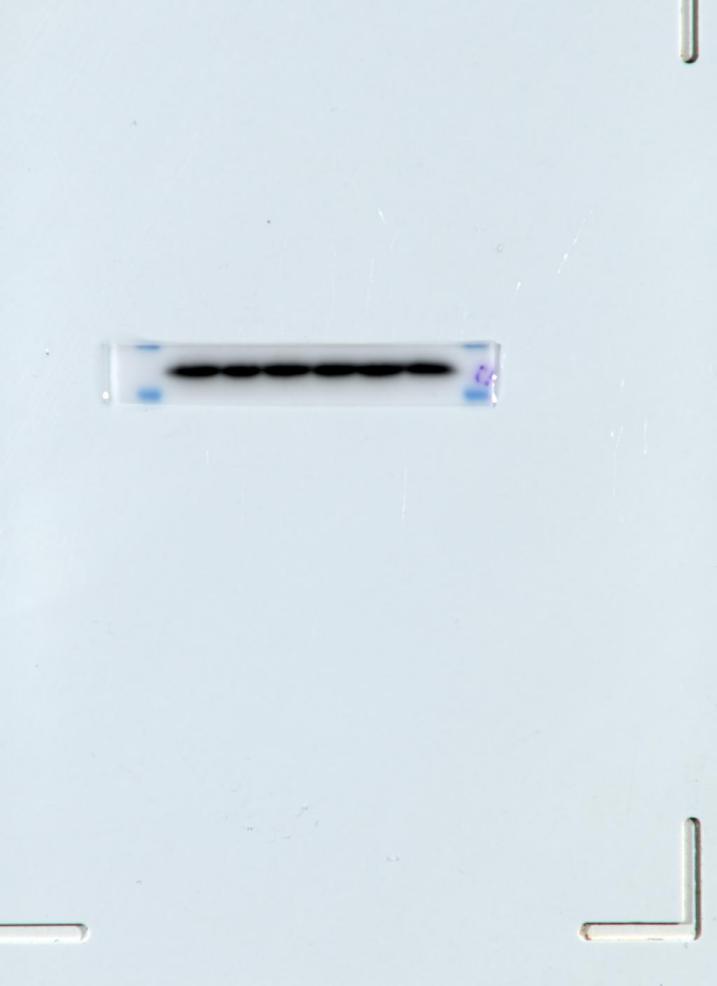


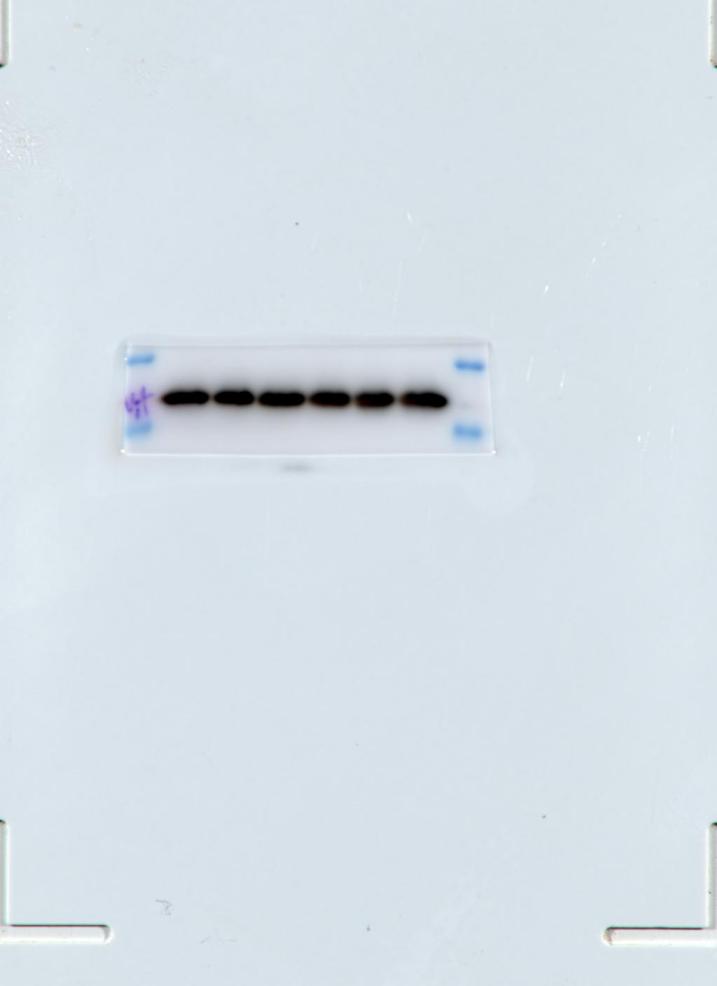


B: PD-L1


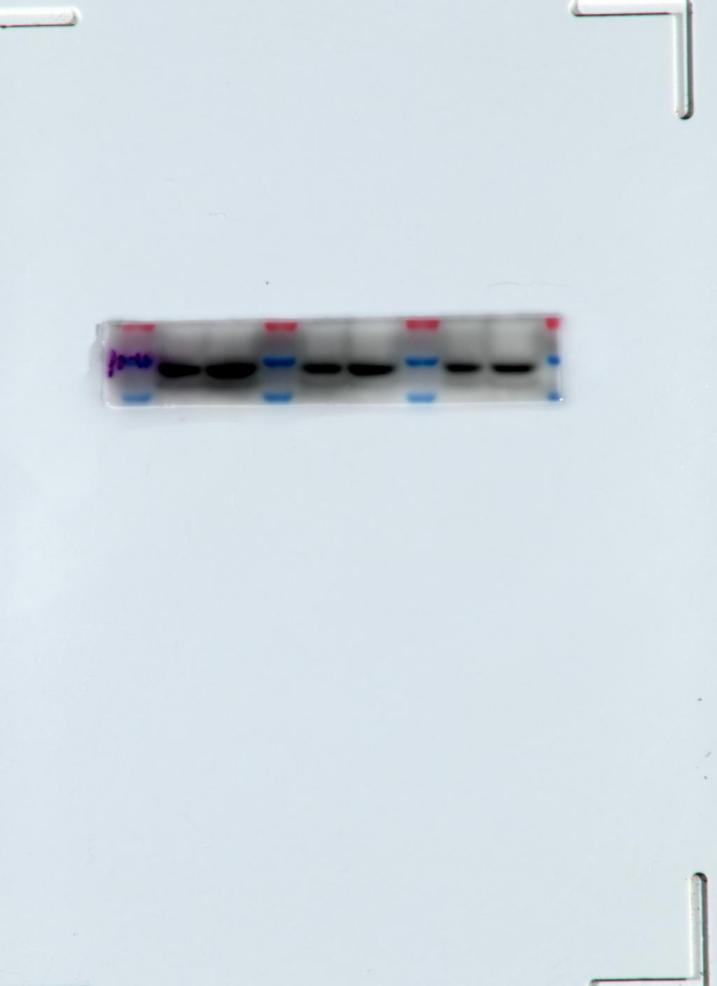


GAPDH:


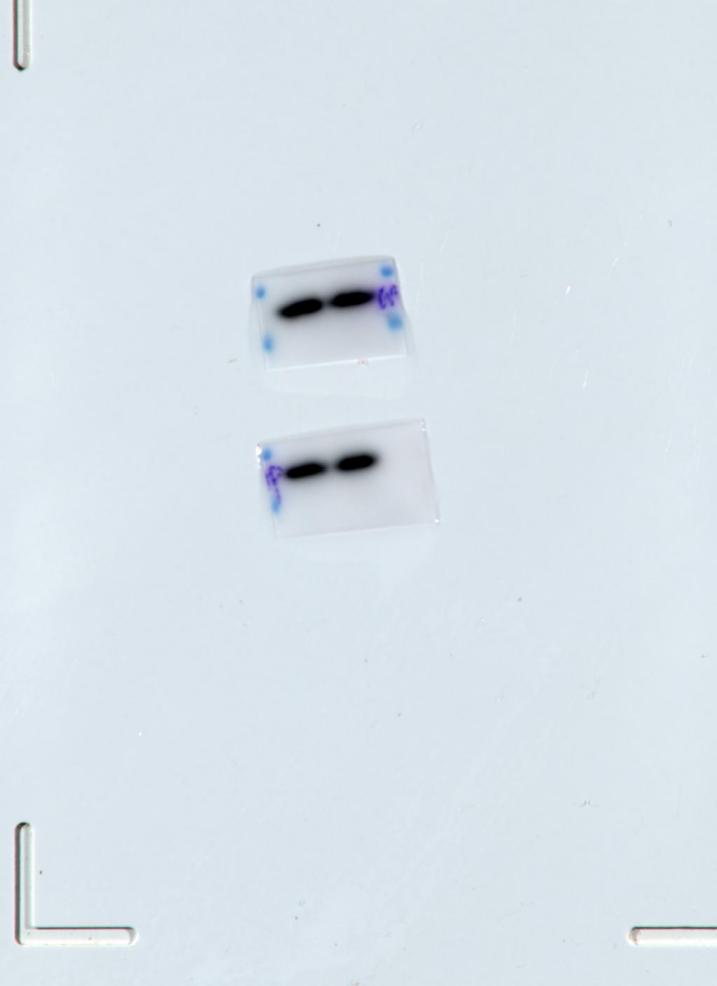


C:PD-L1：


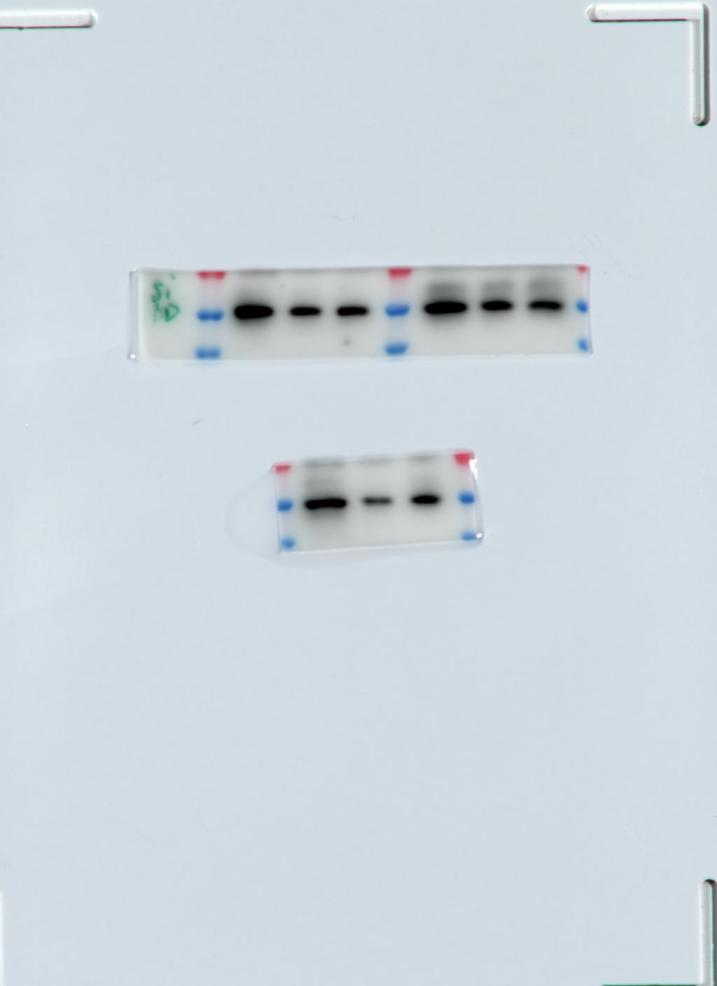


SLC7A11:


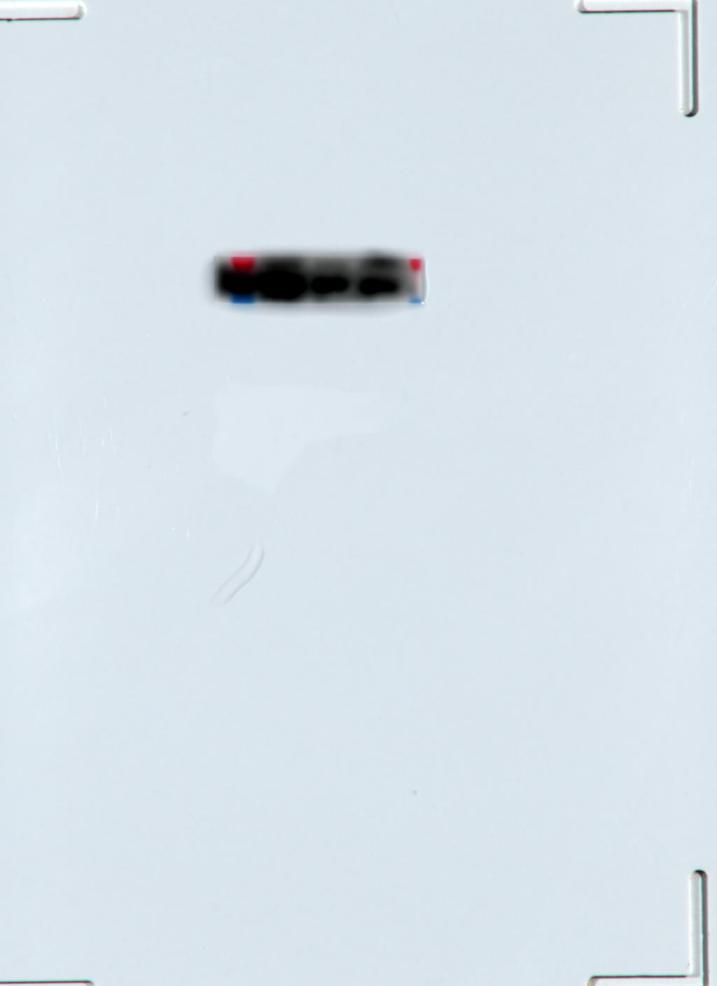

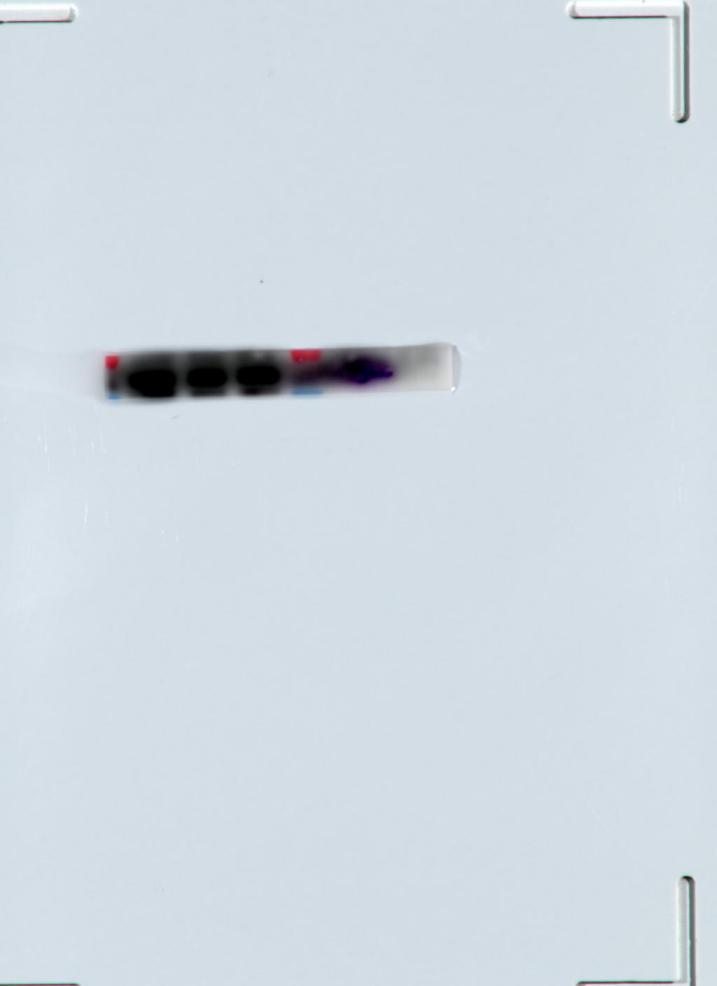


GPX4:


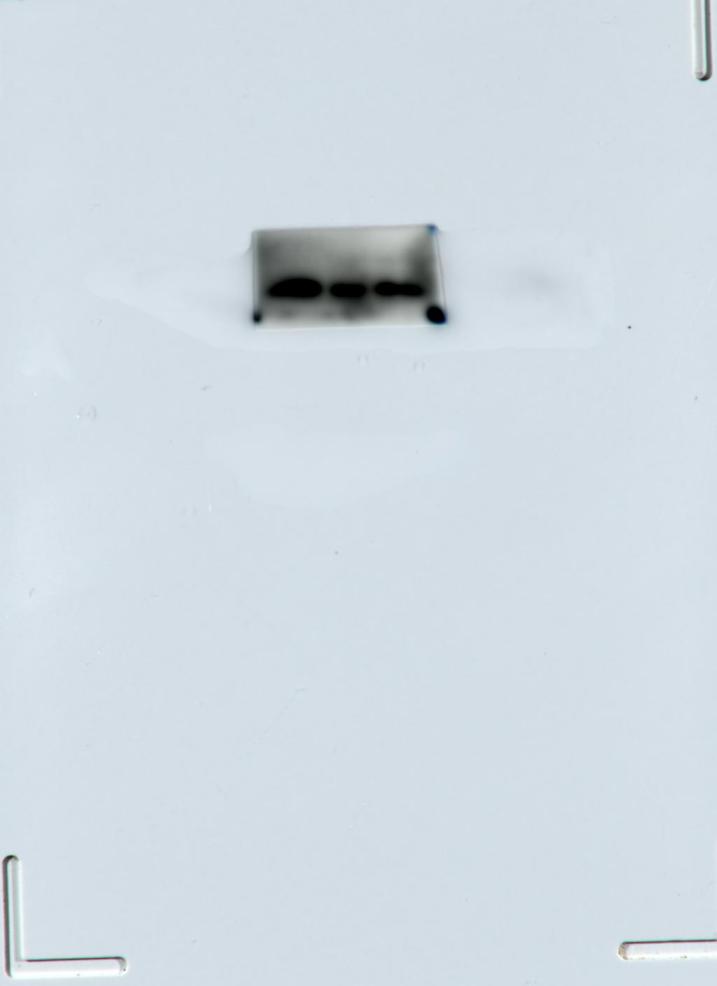


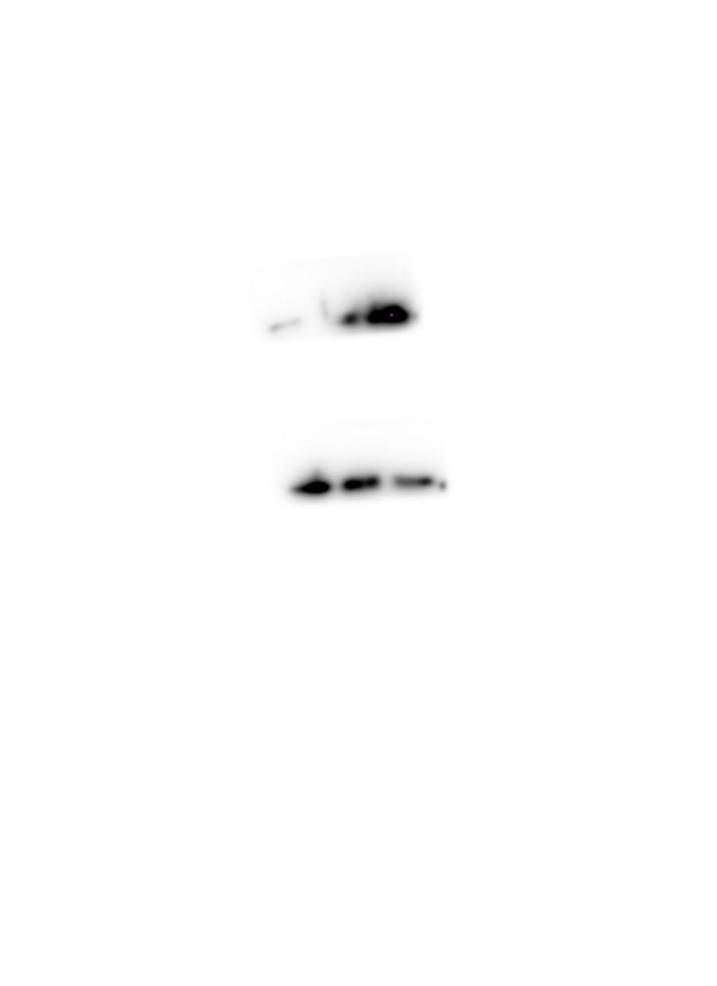


GAPDH:


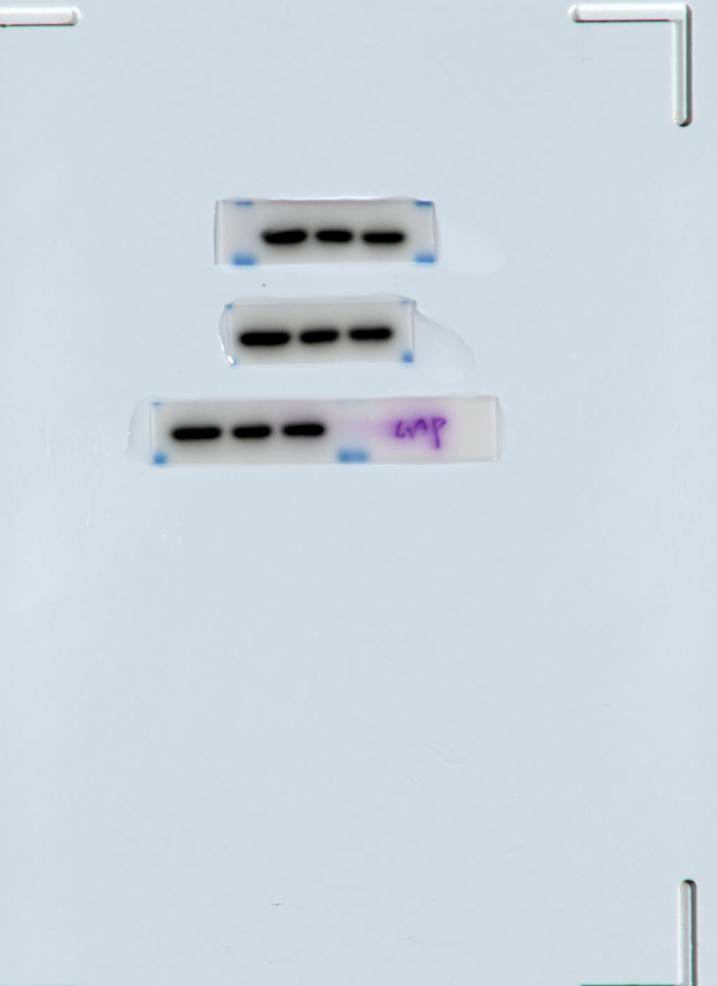


D:ILF3:


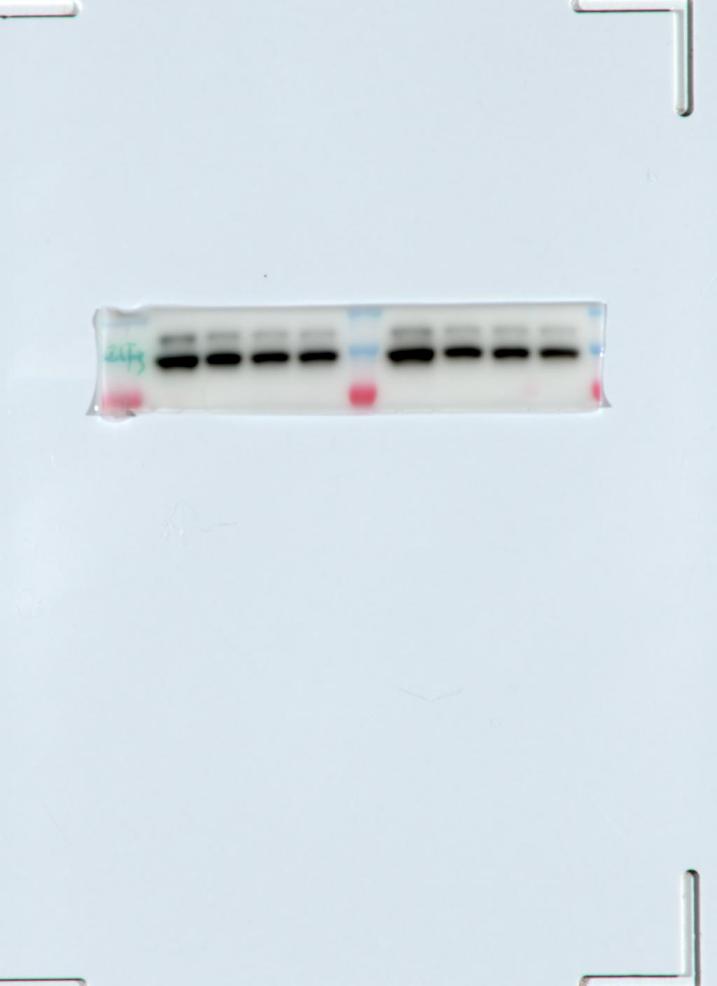


PD-L1:


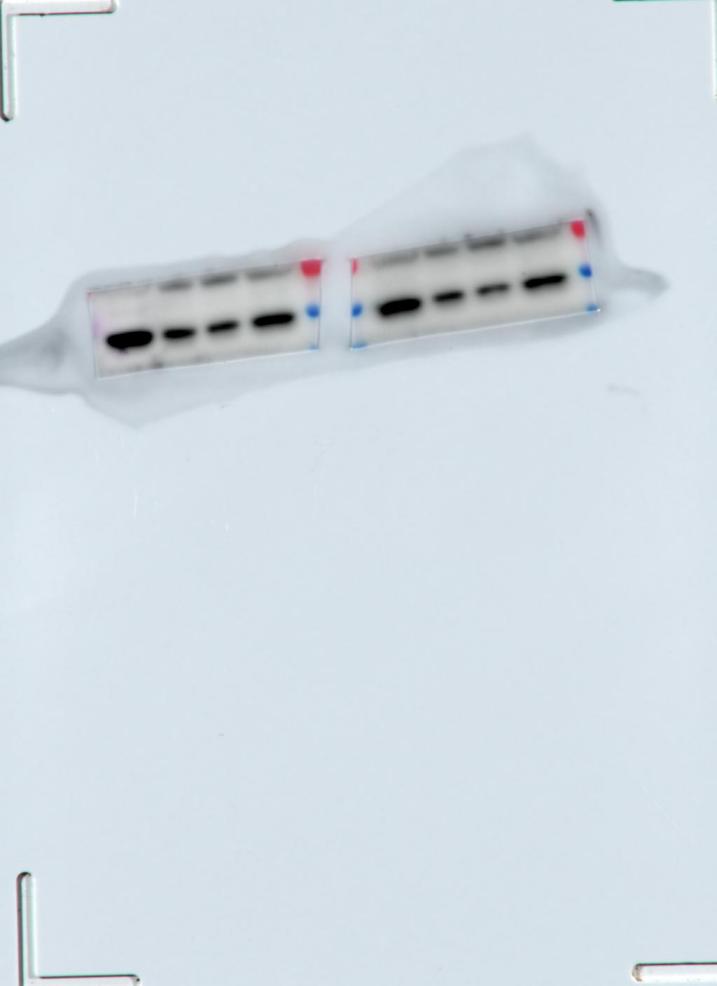


SLC7A11


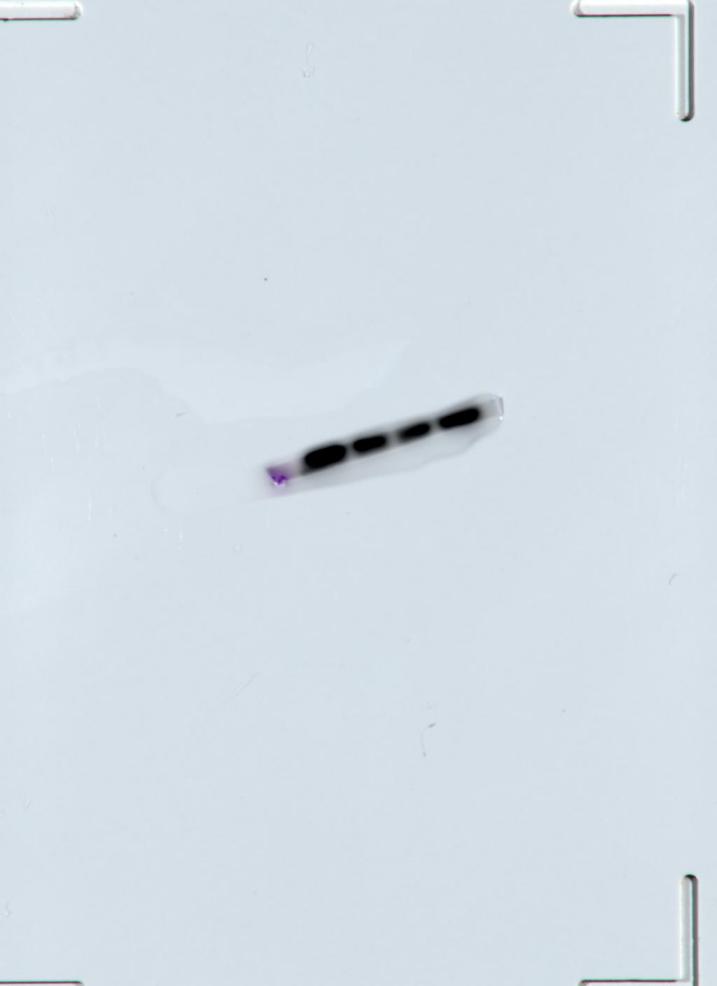


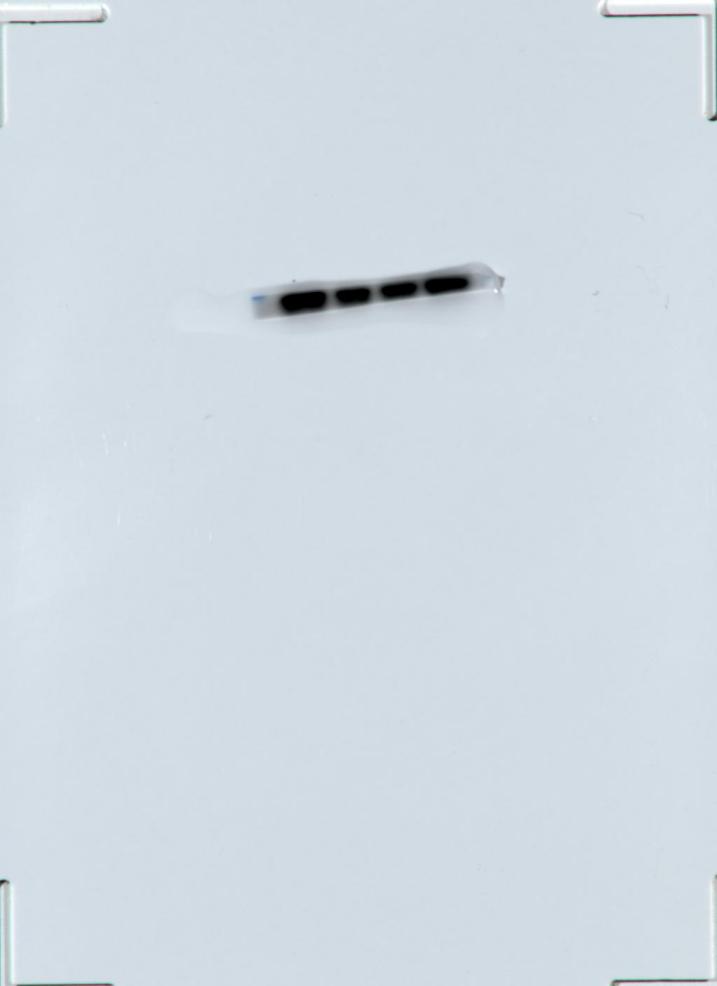


GPX4:


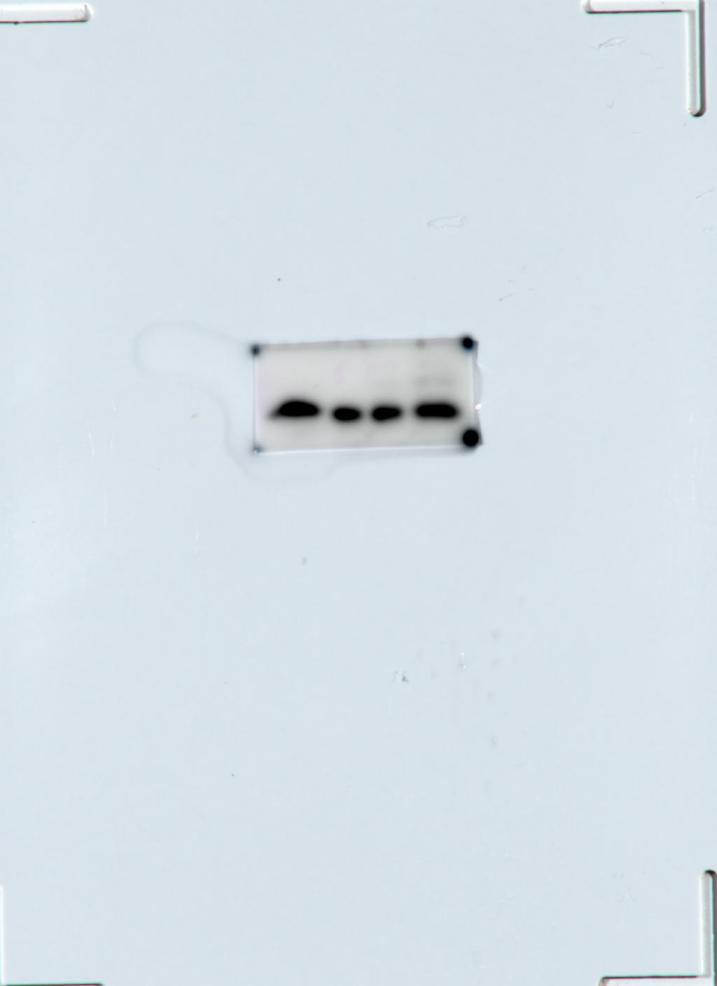


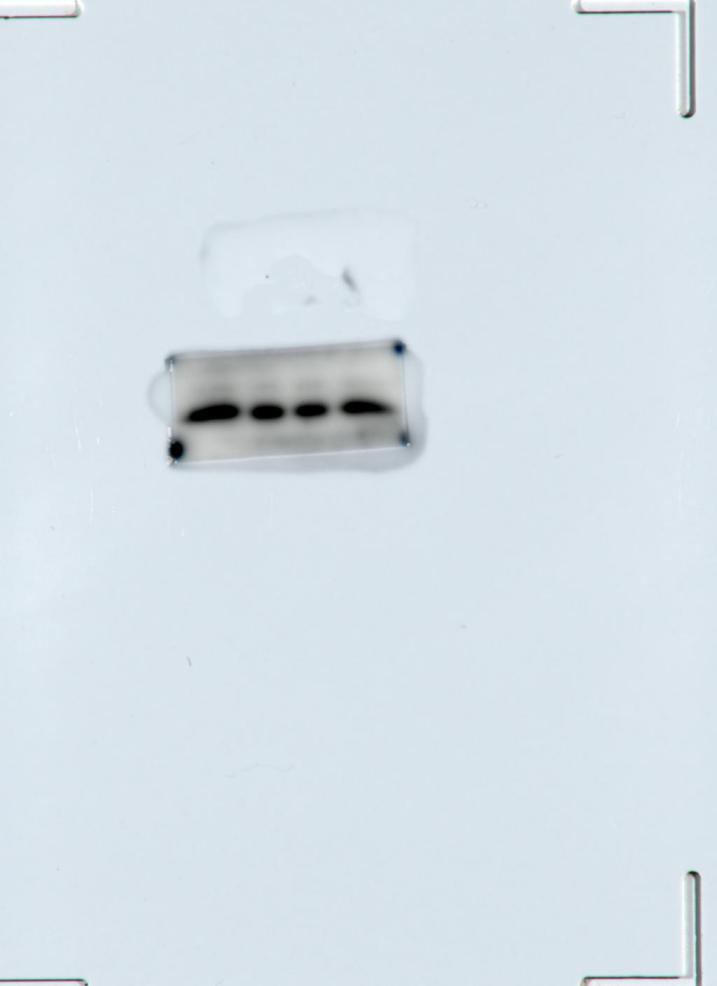


GAPDH:


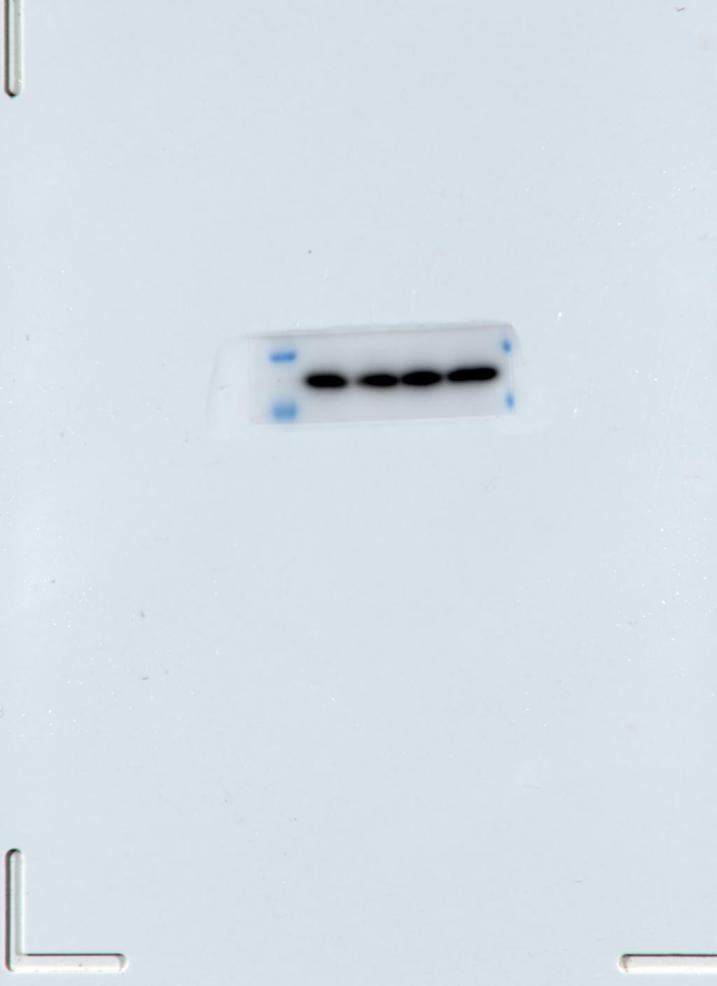


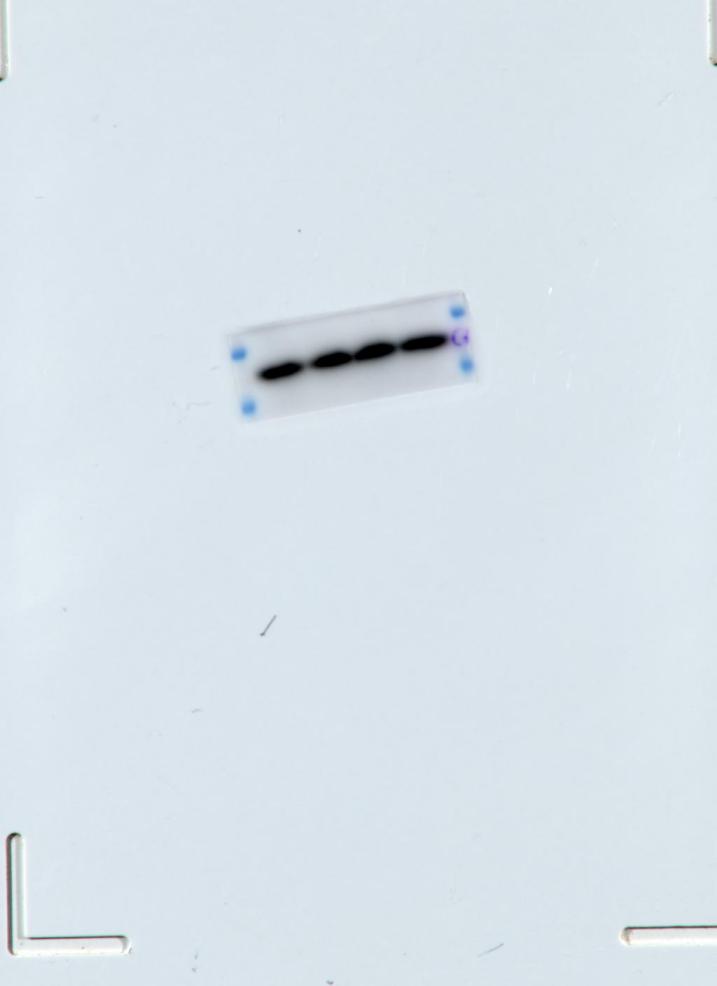


Fig 5B:

ILF3:


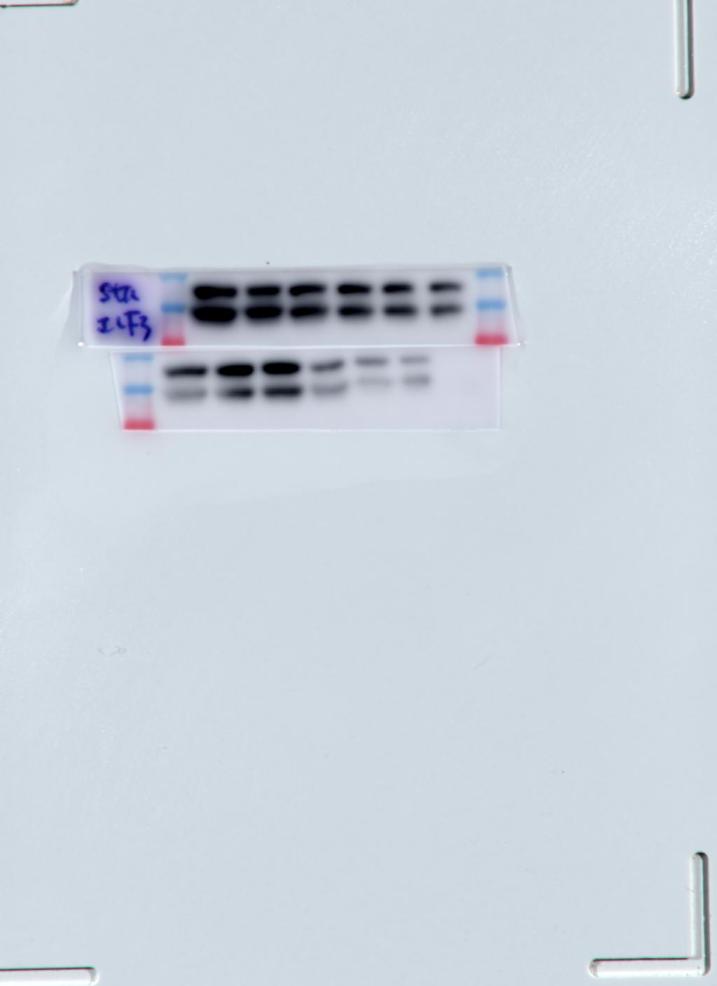


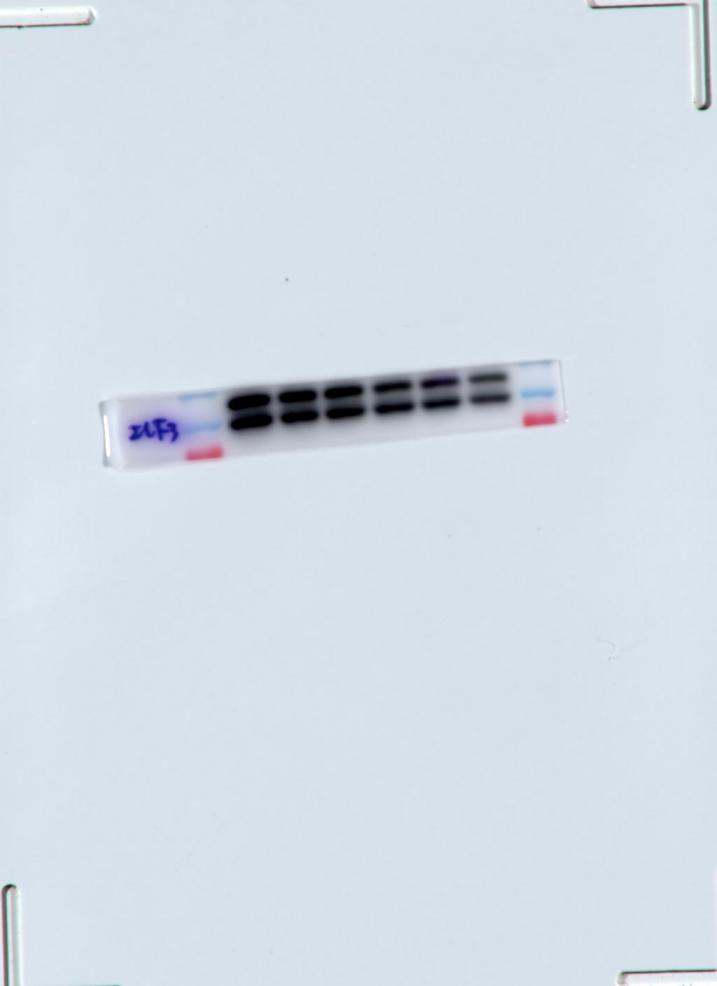


HDAC1:


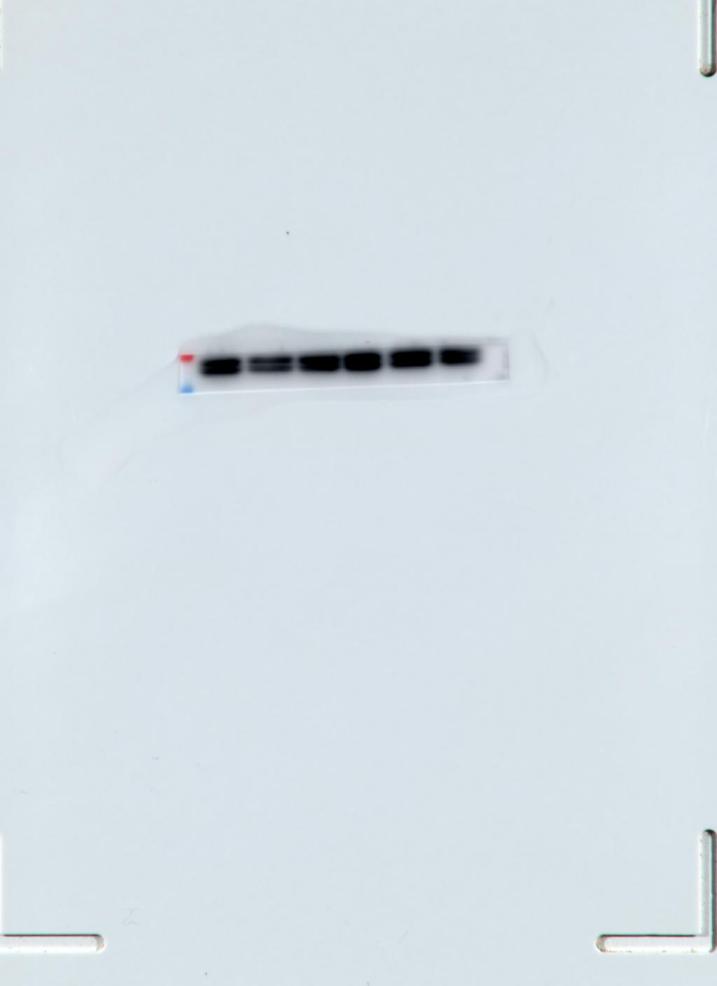


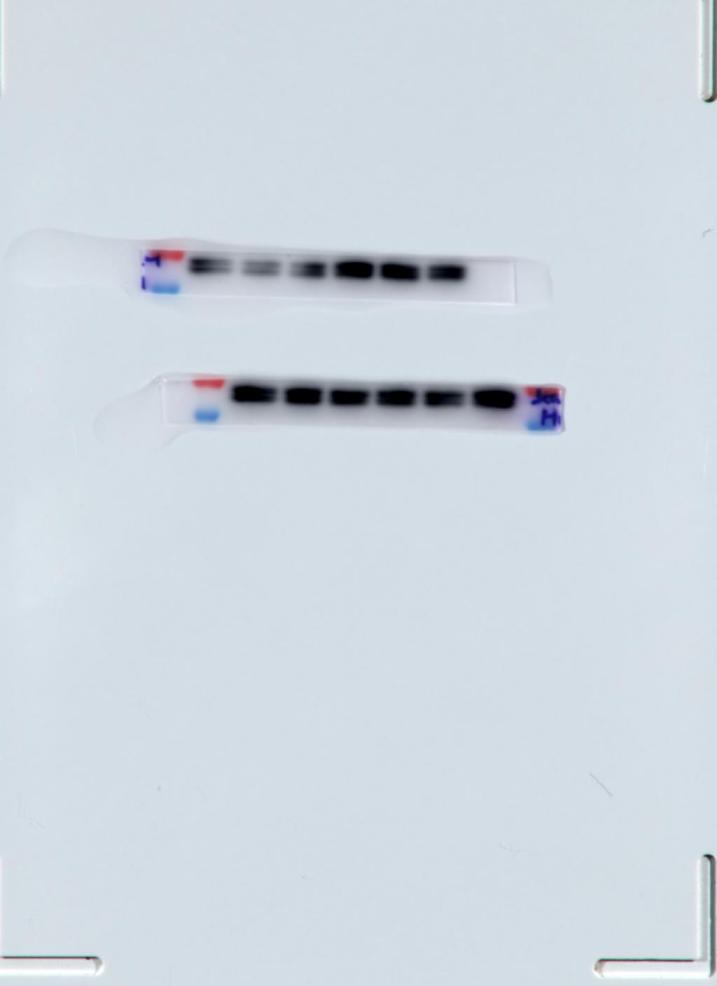


HDAC2:


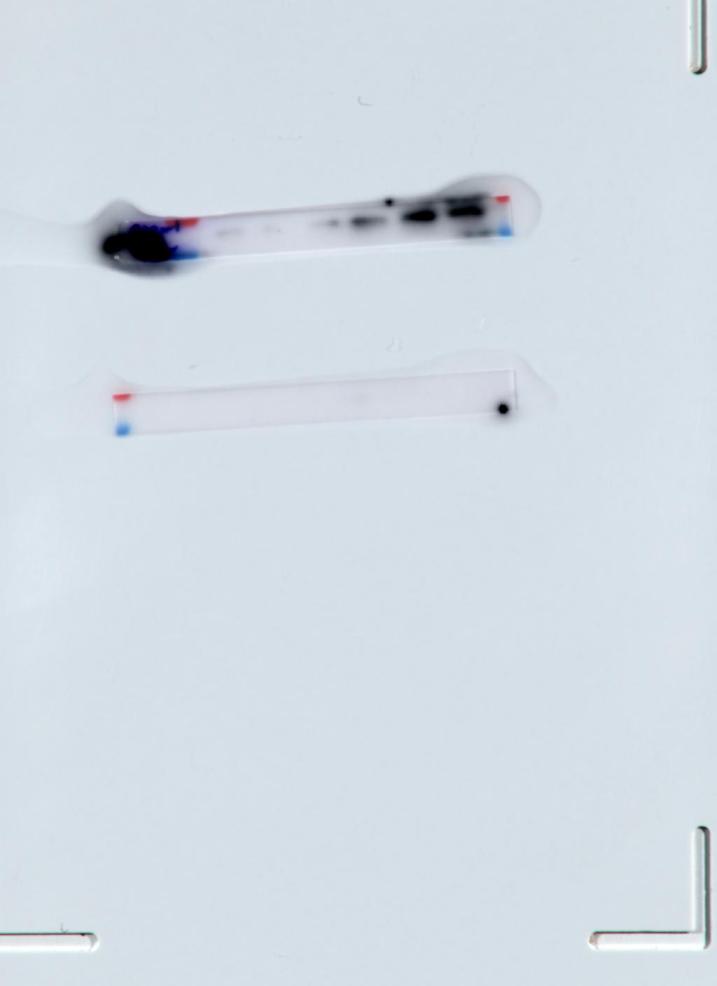


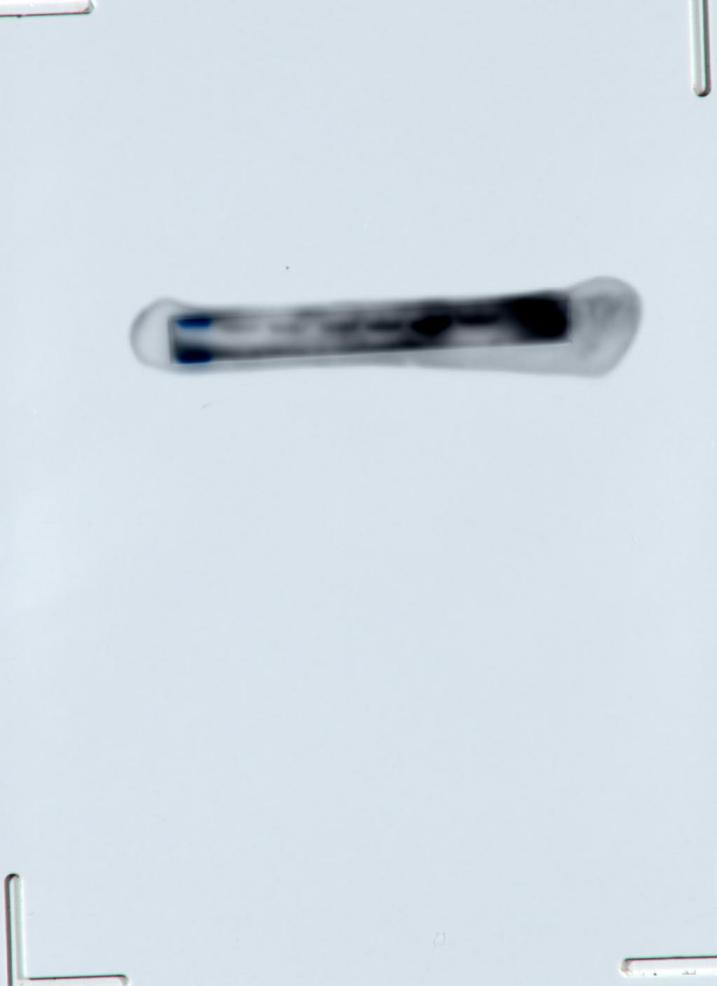


HDAC6:


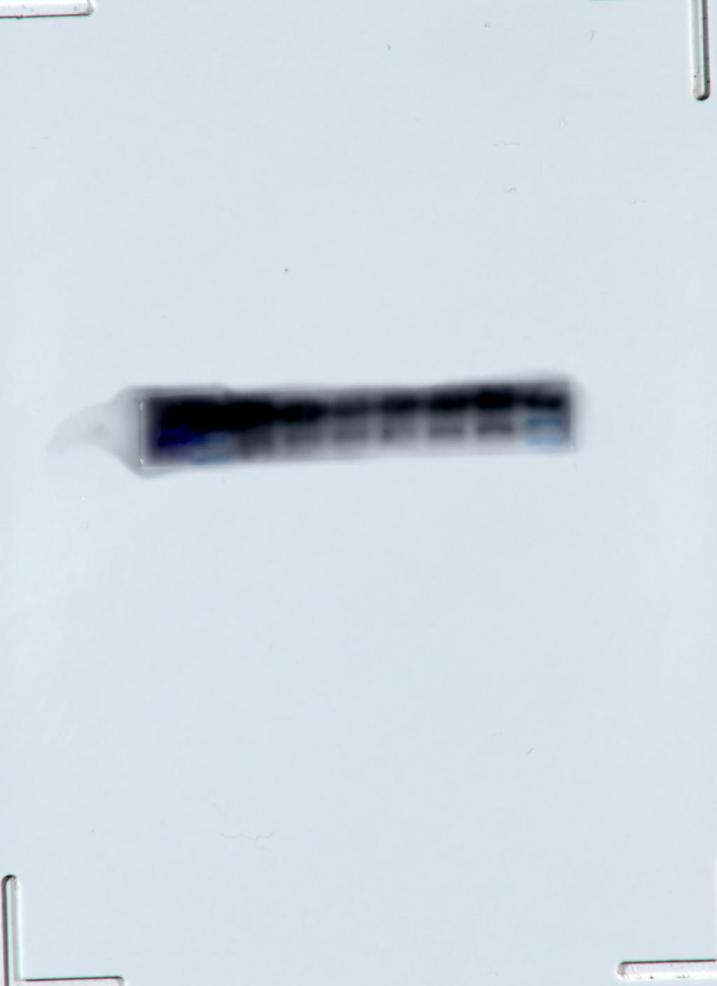


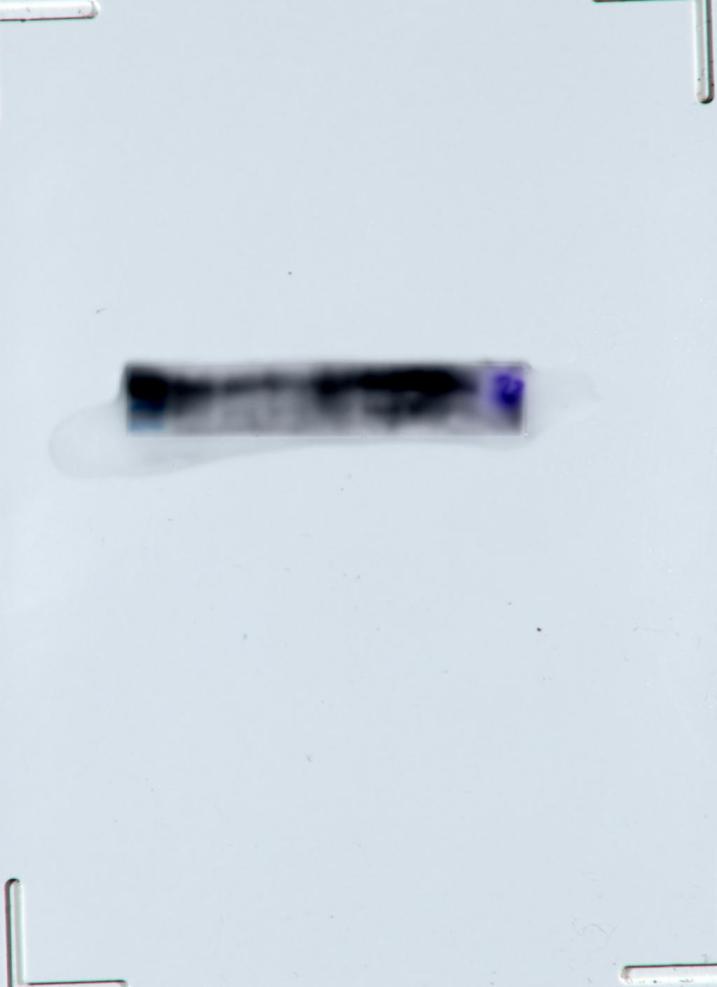


GAPDH


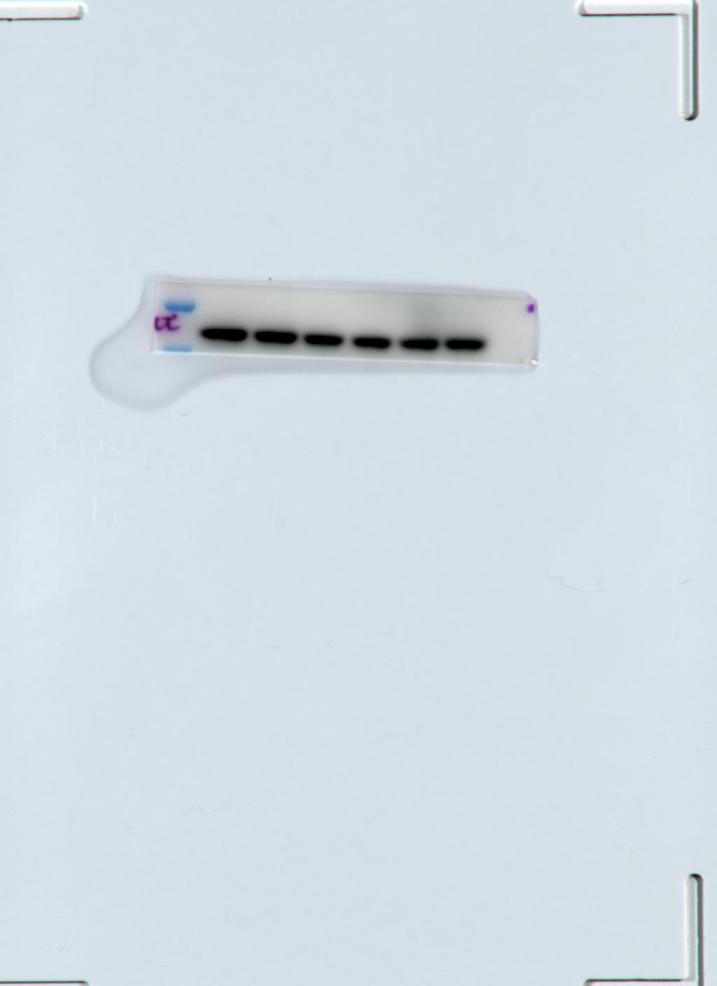


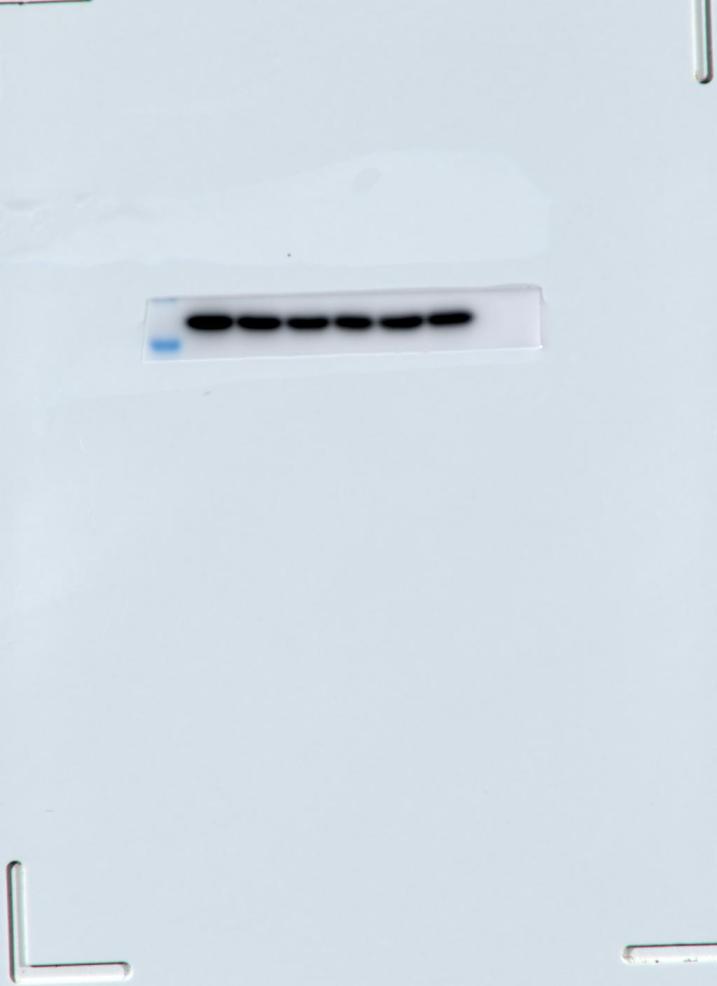


Figure5C:

ILF3:


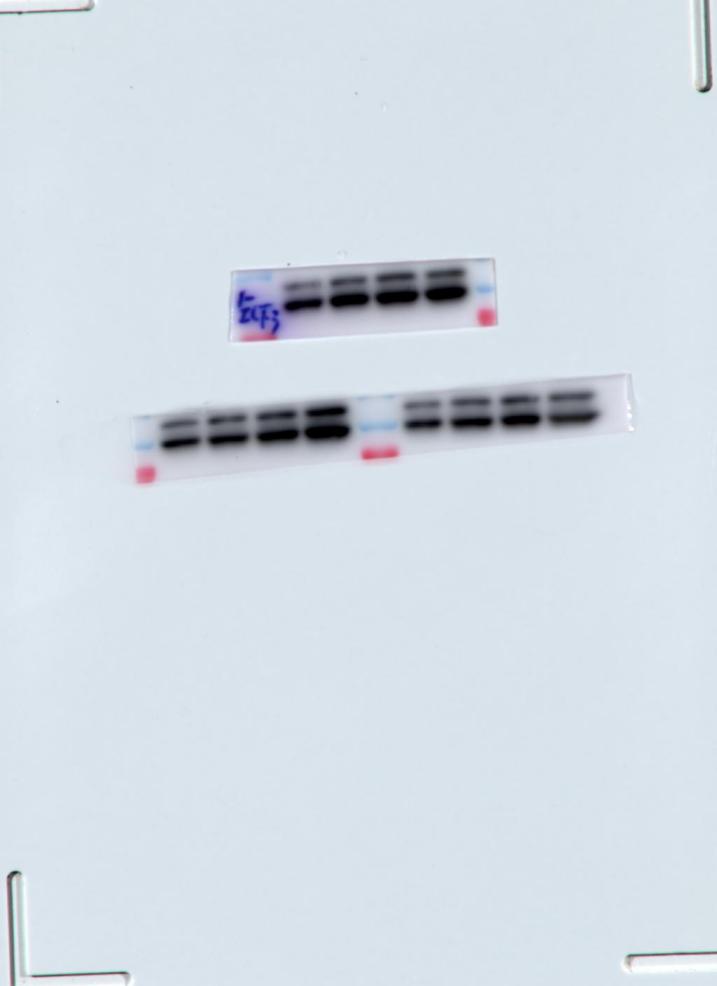


HDAC1:


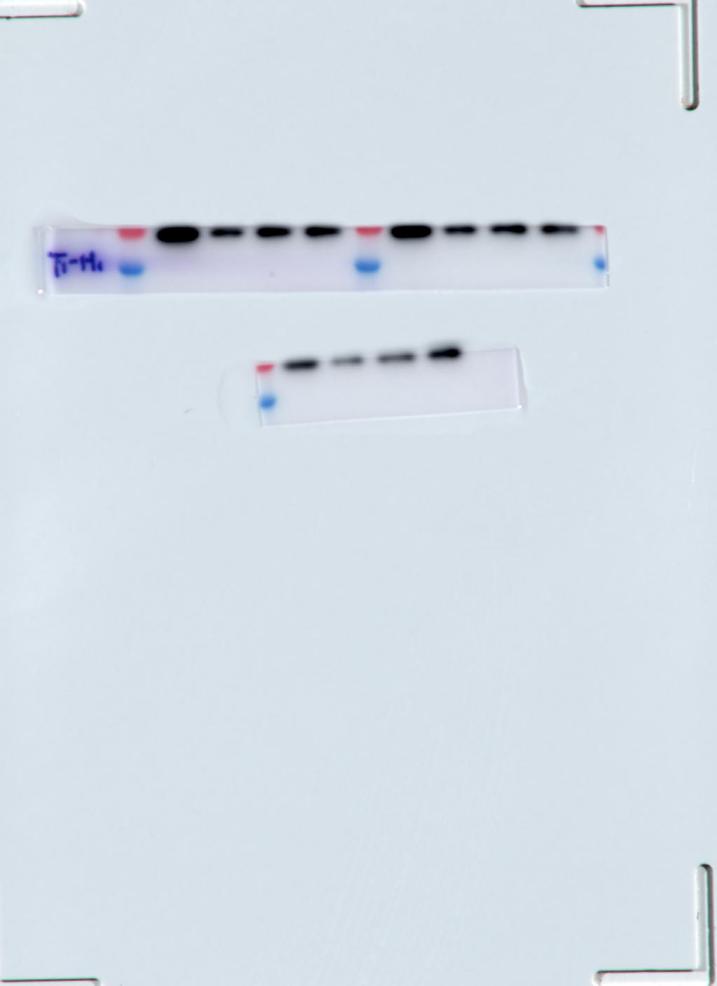


HDAC2:


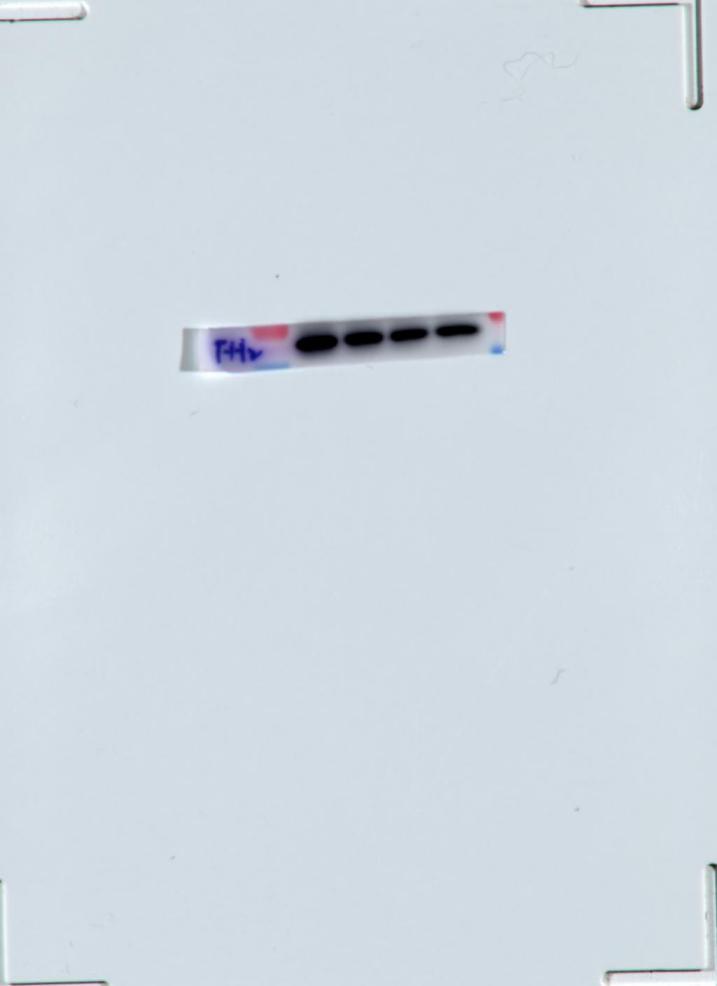


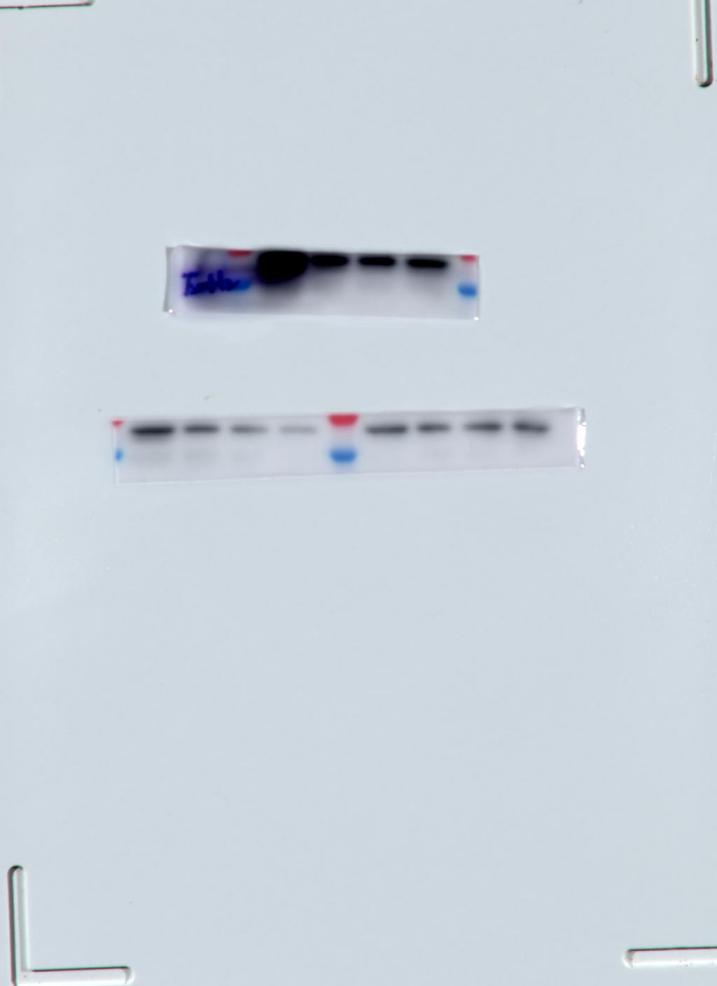


HDAC6:


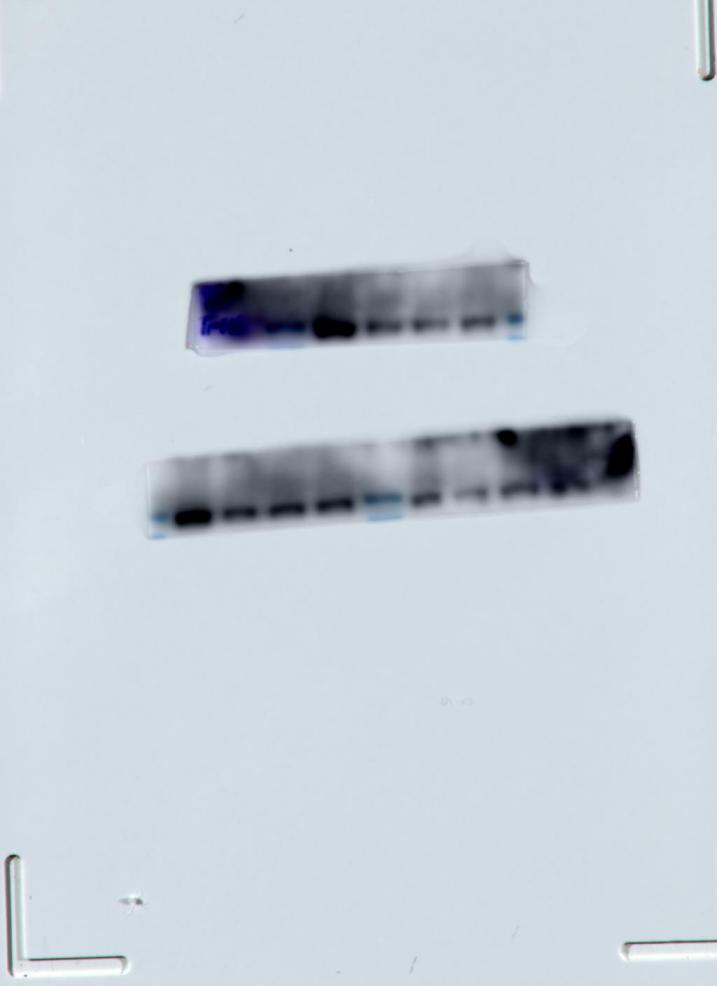


GAPDH:


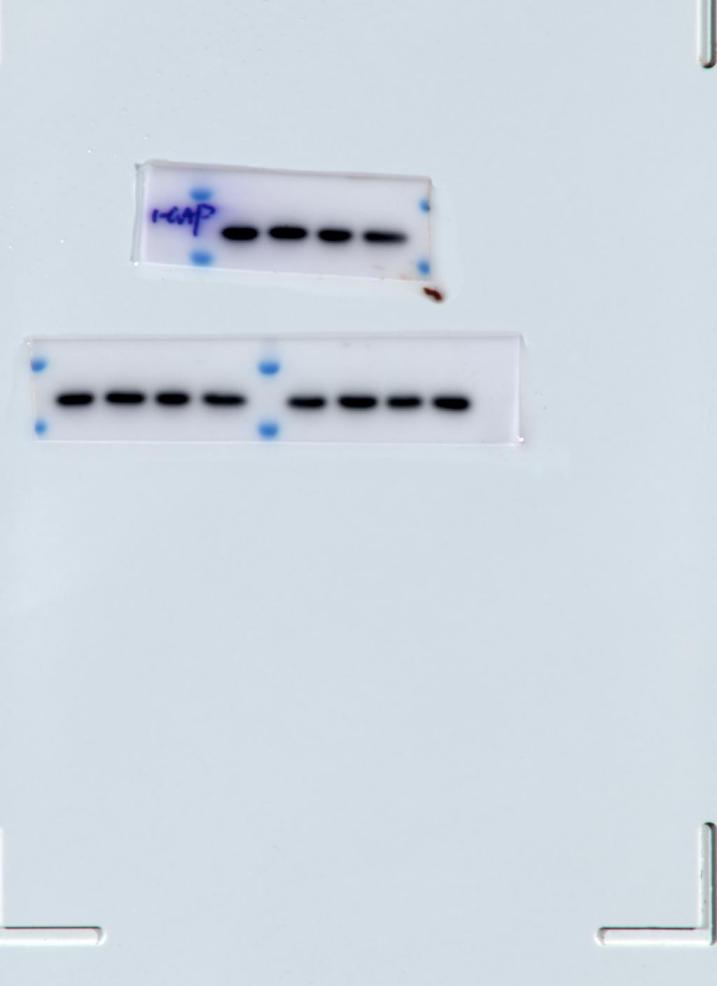


Figure5D:

Total acetylation:


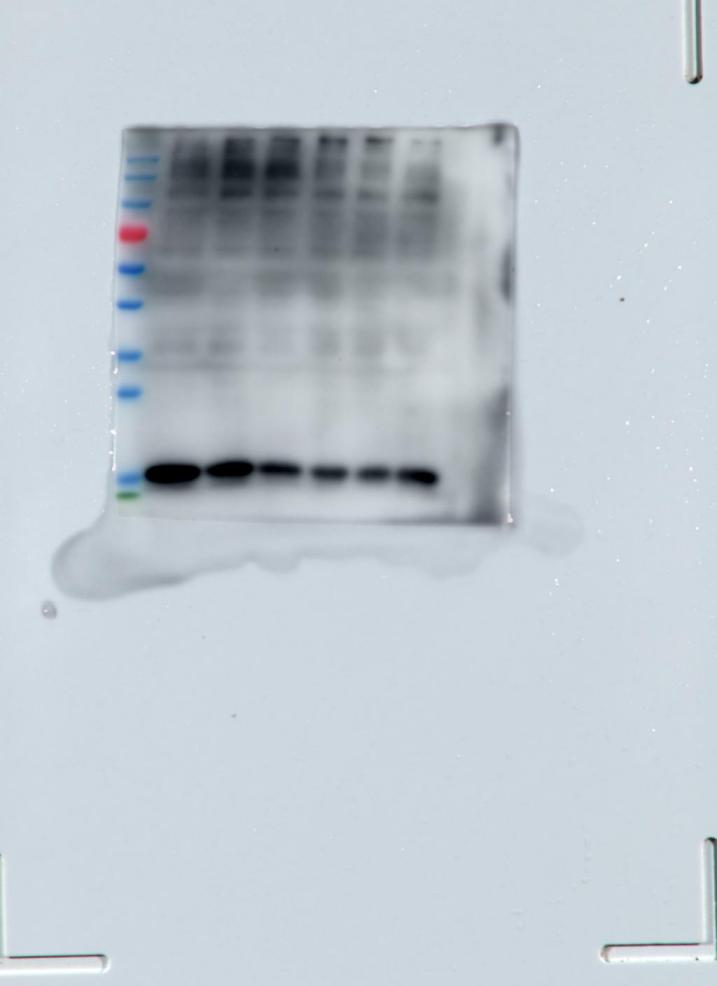


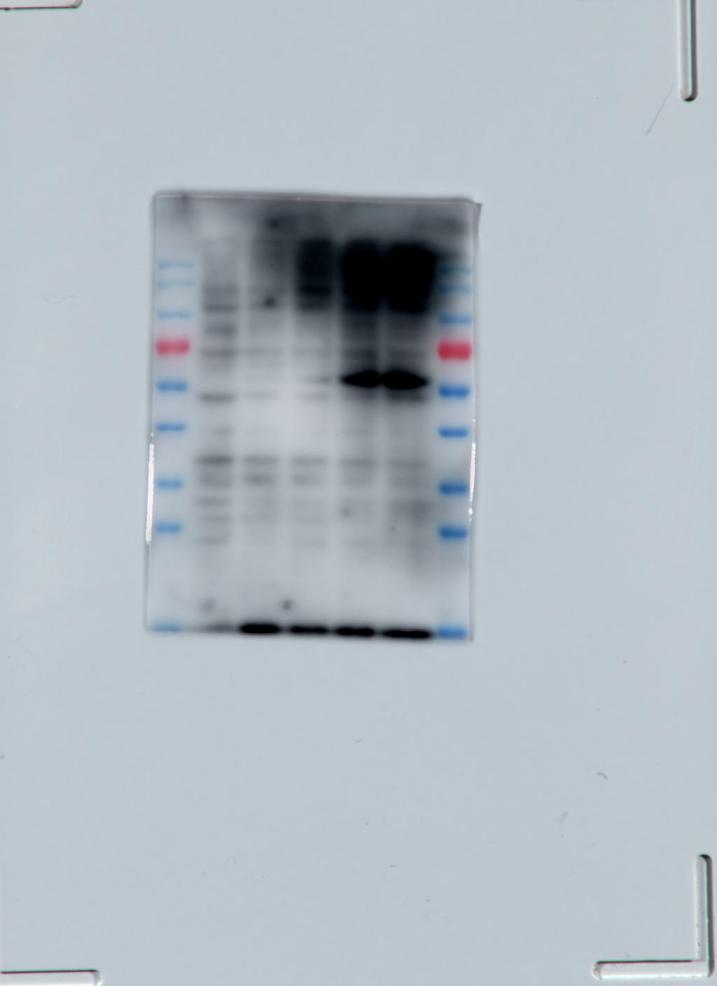


GAPDH：


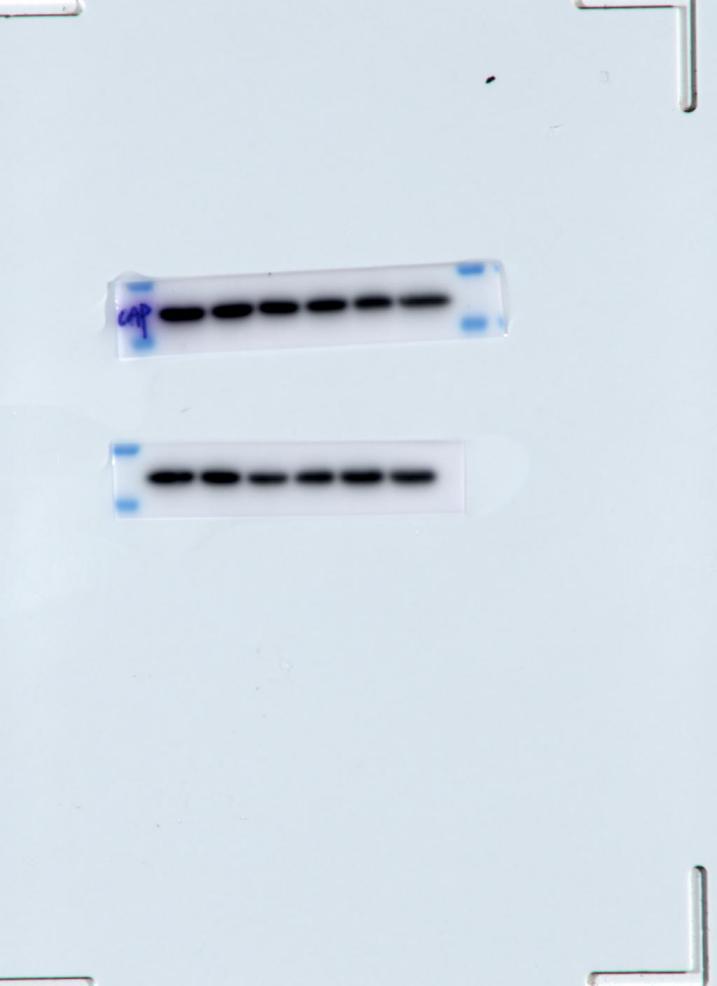


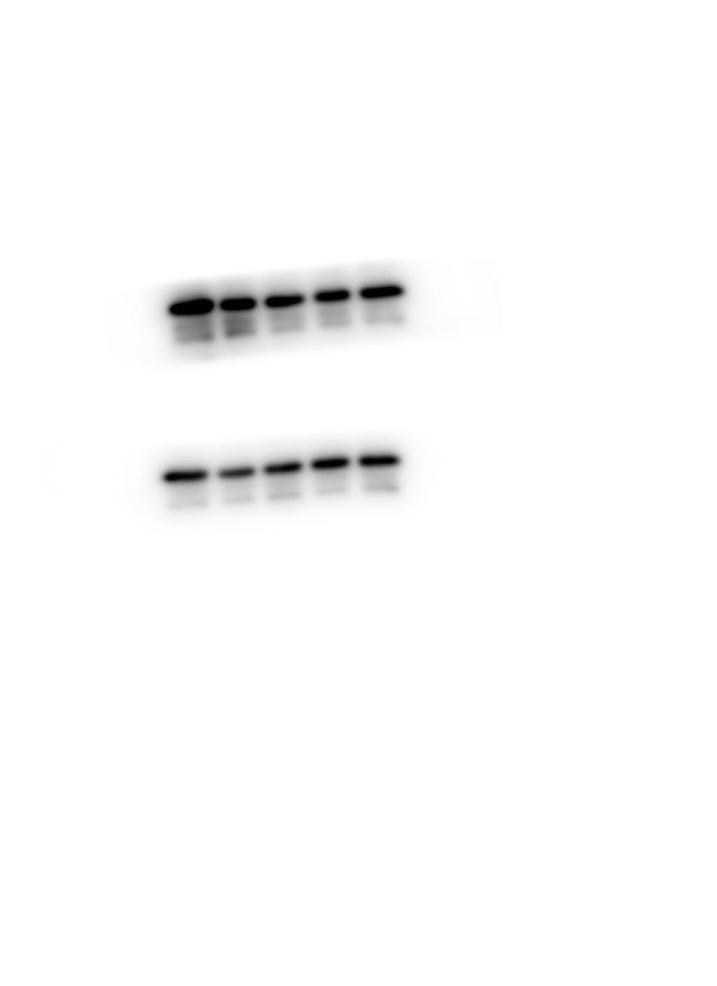


Figure5E:

ILF3:


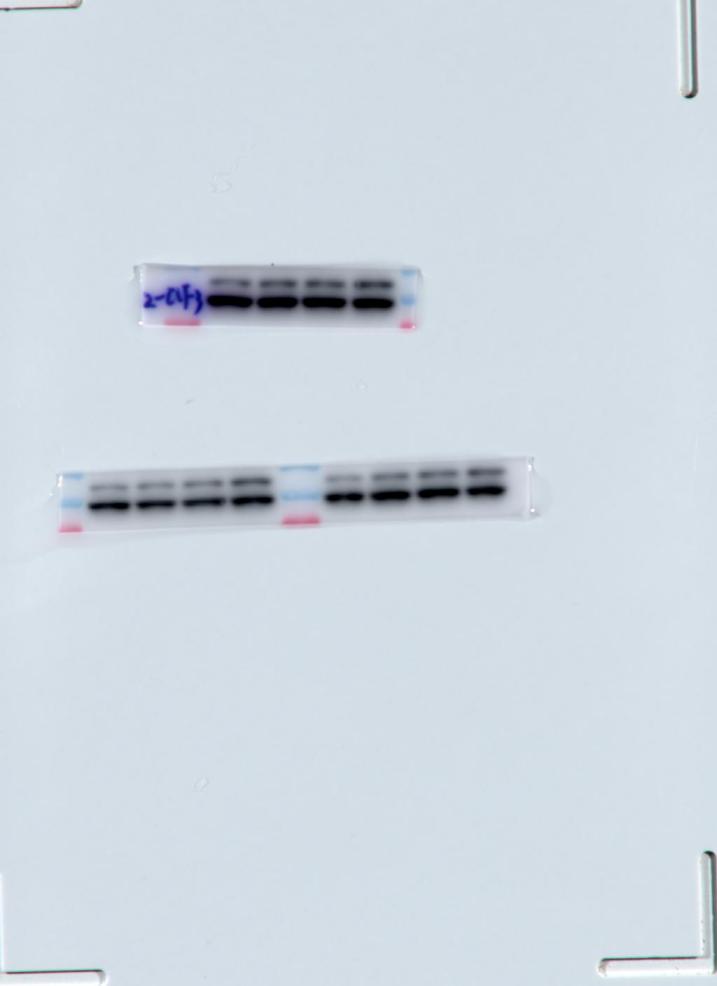

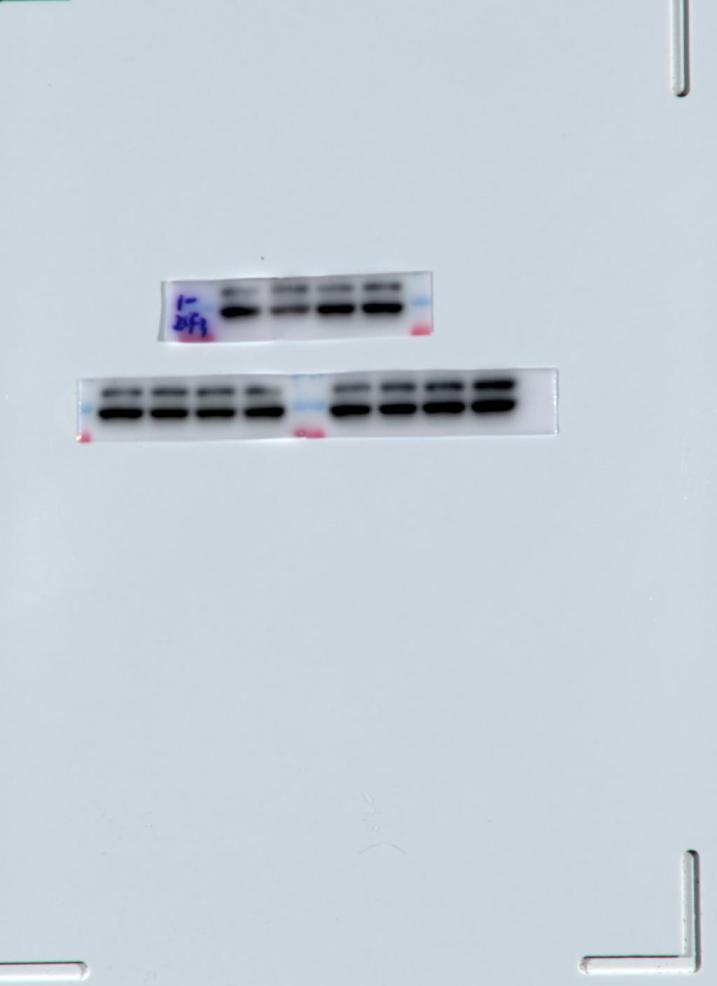


HDAC1:


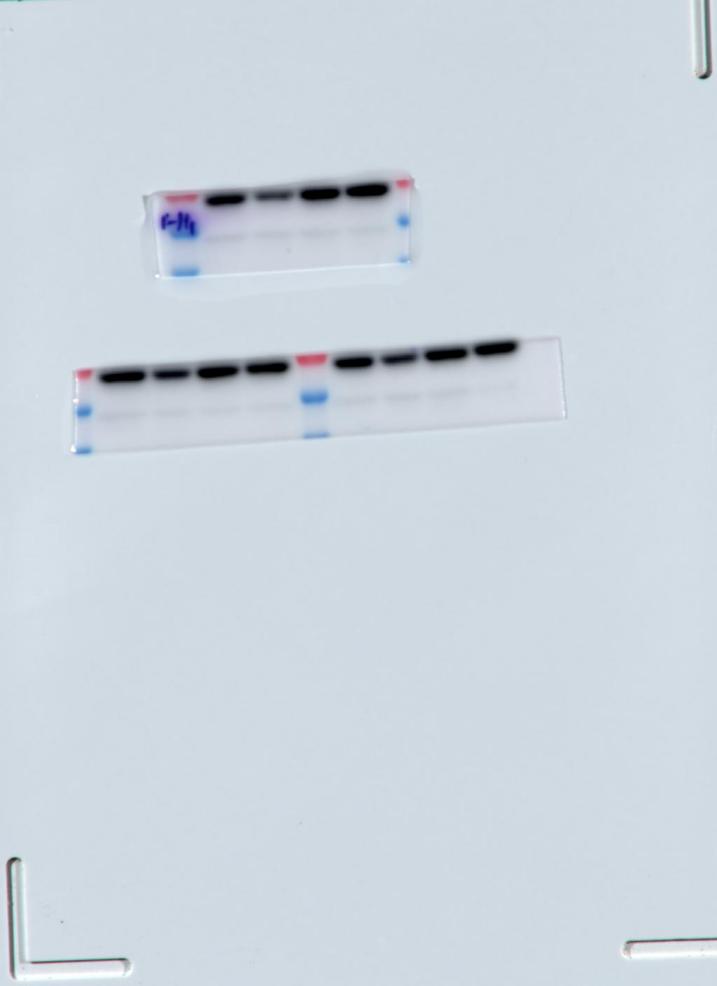


HDAC2:


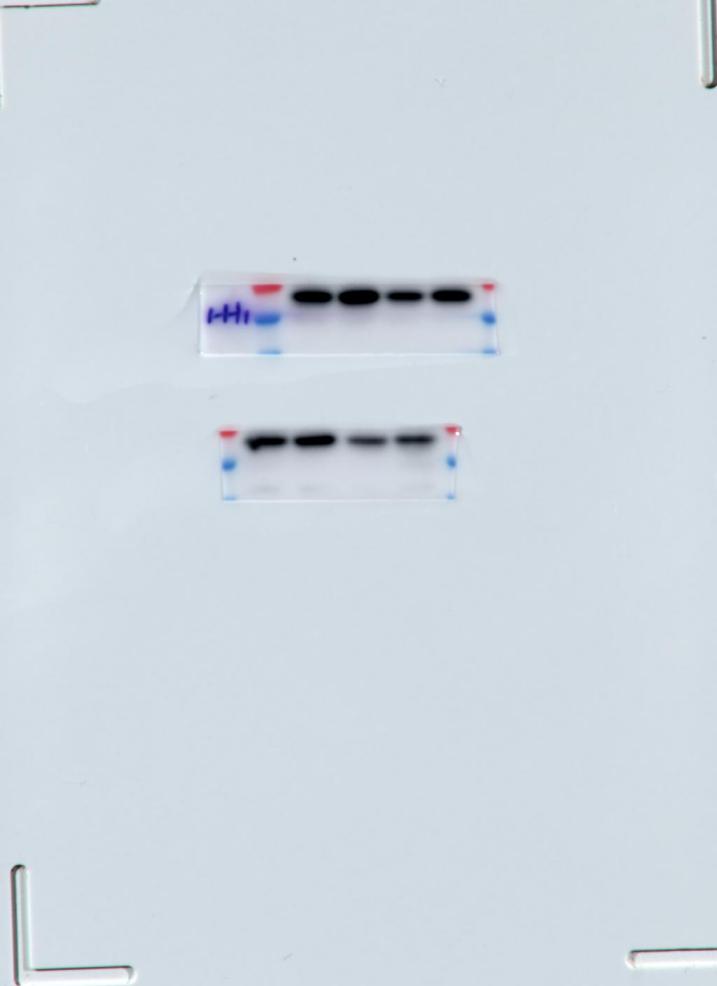


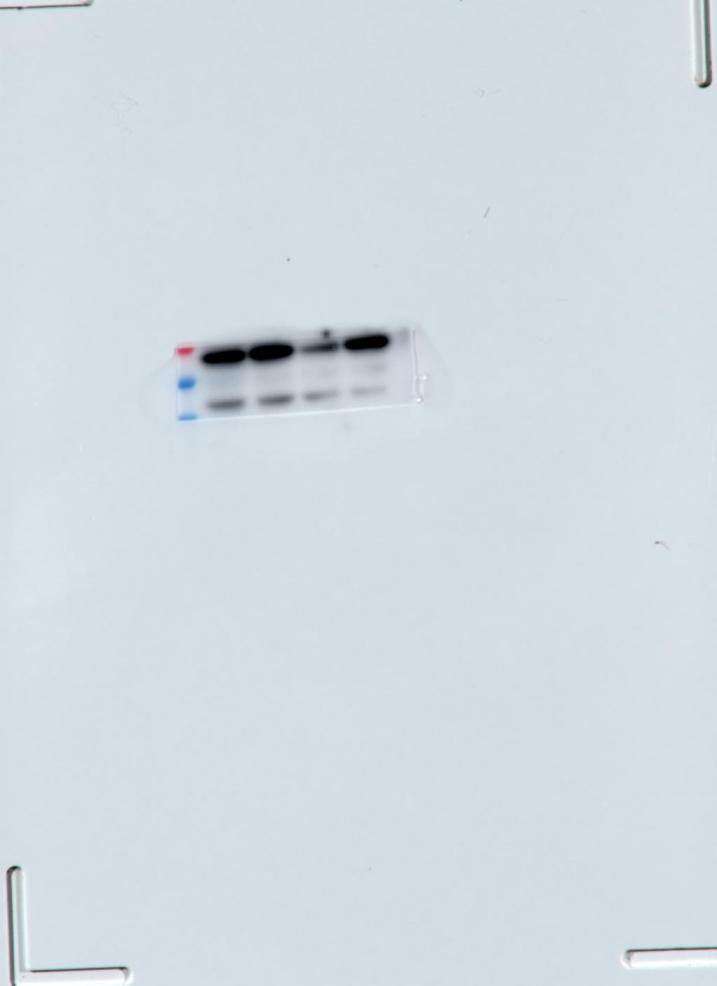


HDAC6:


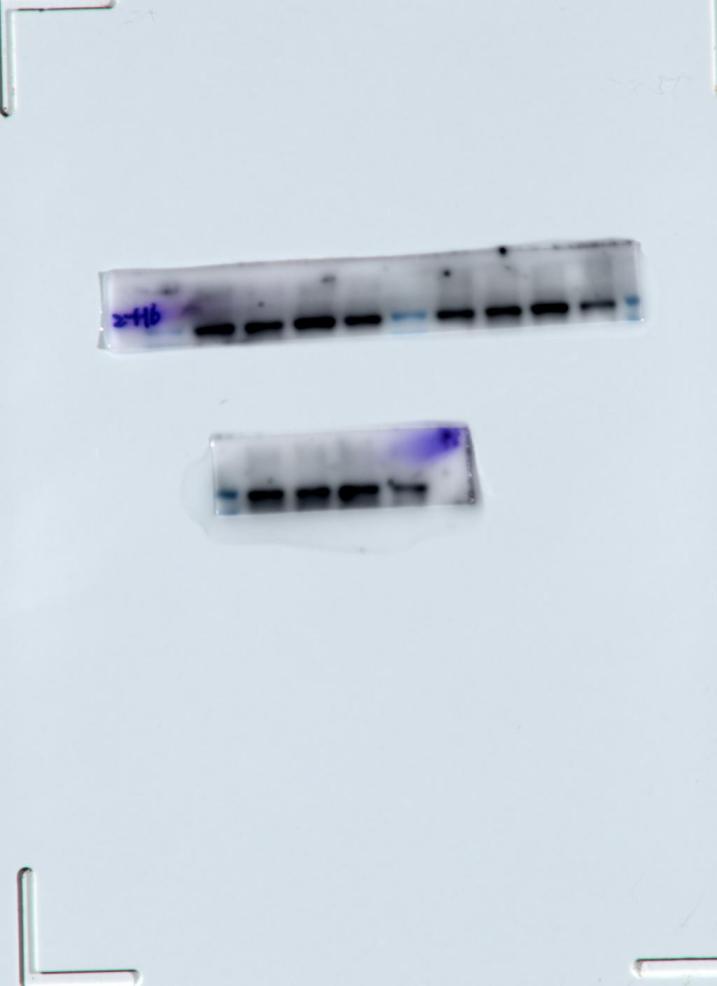


GAPDH:


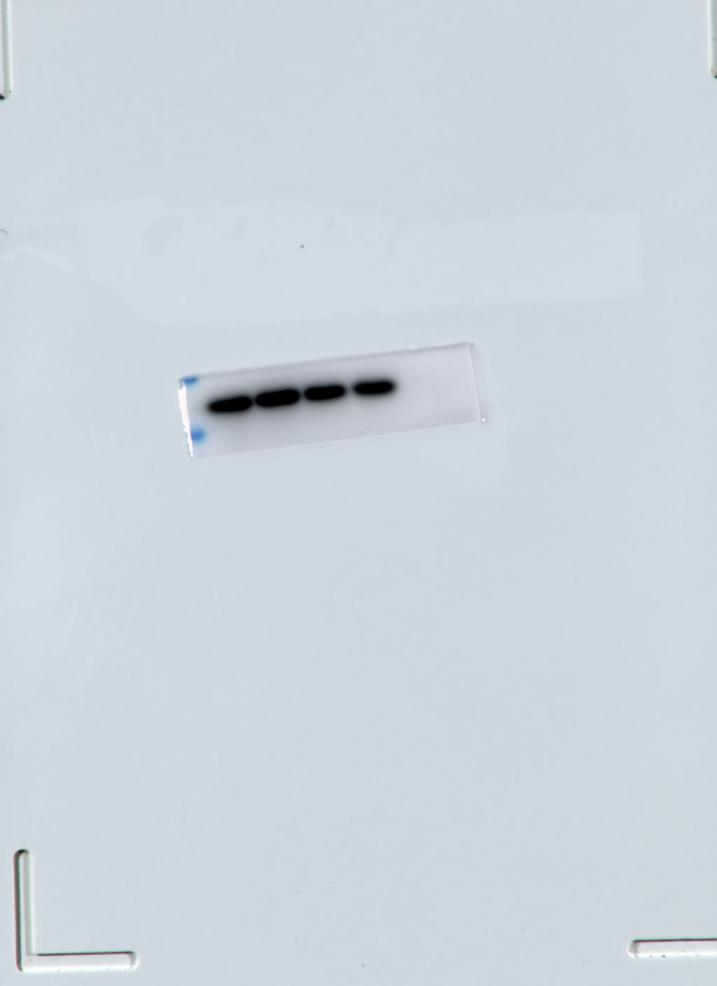


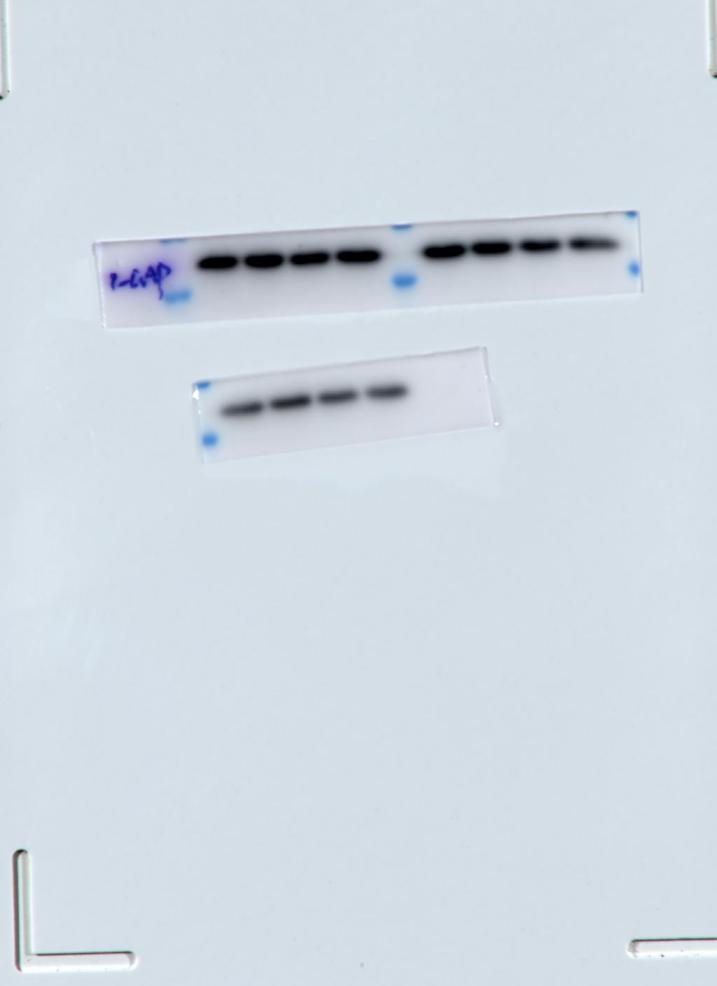


Fig 5H:

H3K13ac:


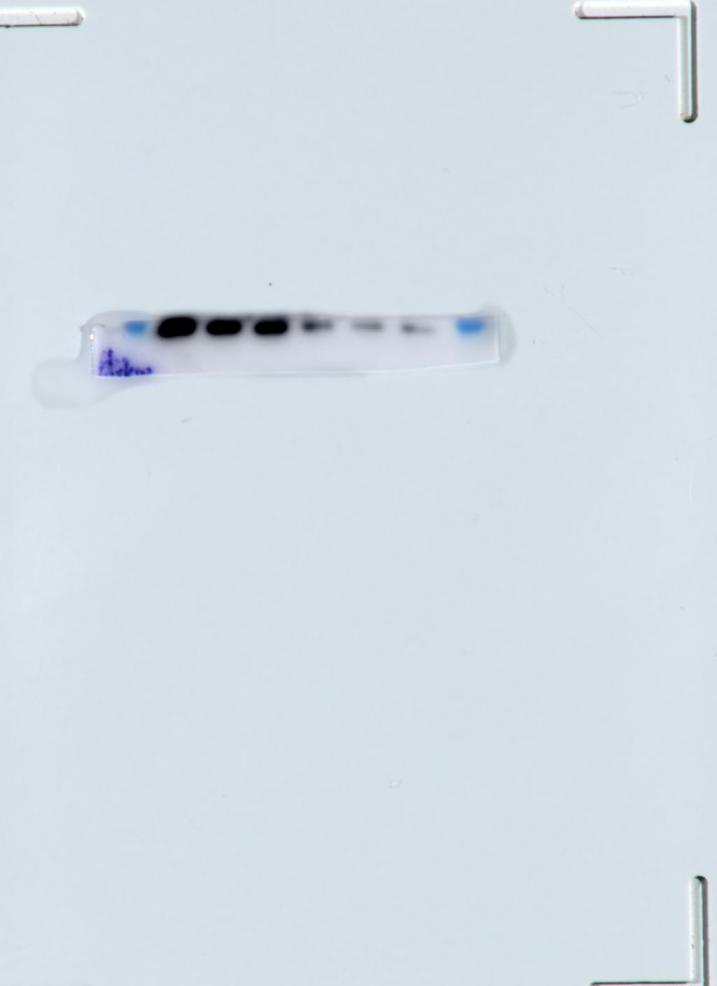


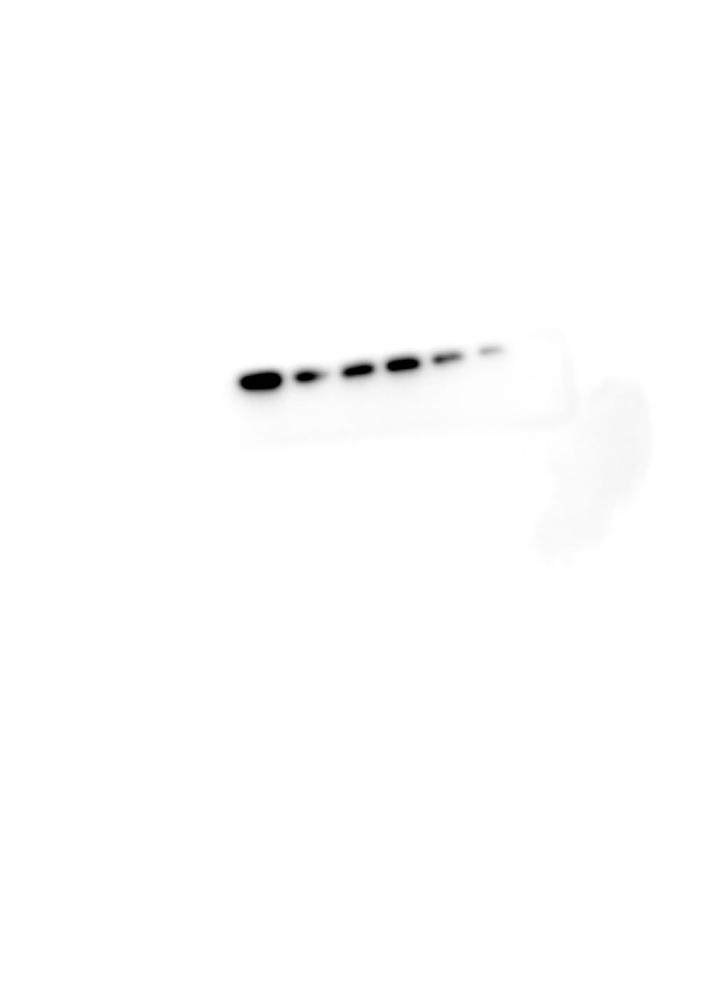


PCNA:


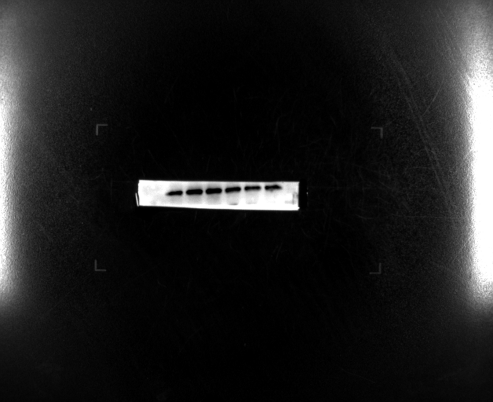


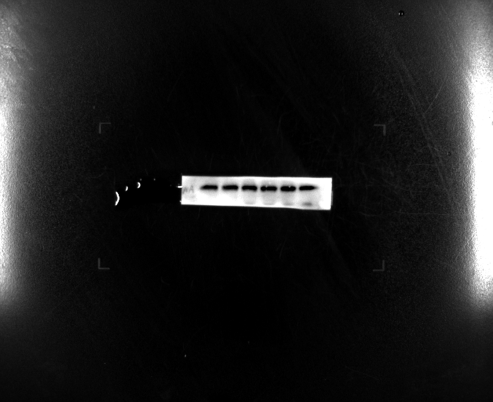


Figure 5I:

ILF3:


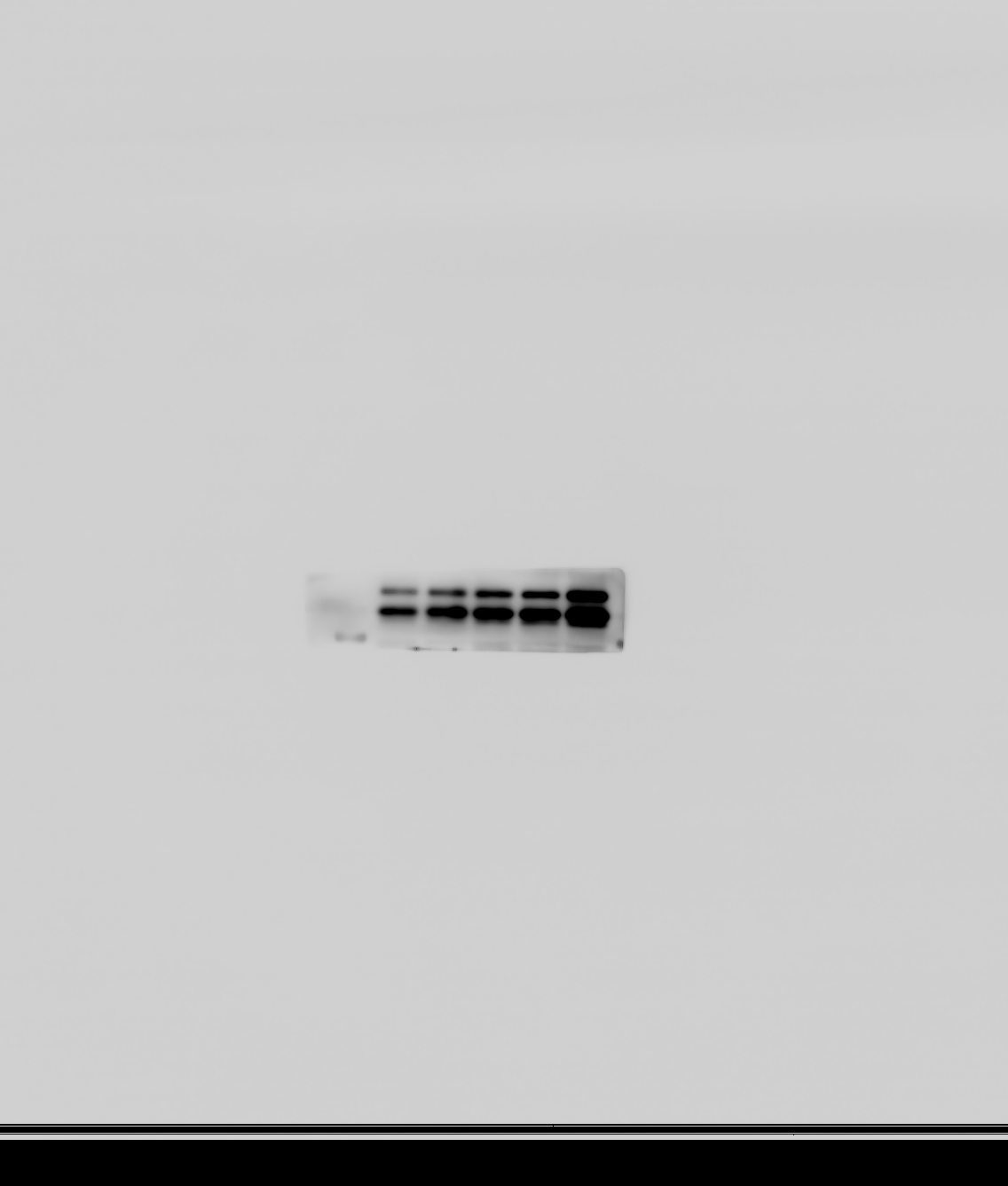


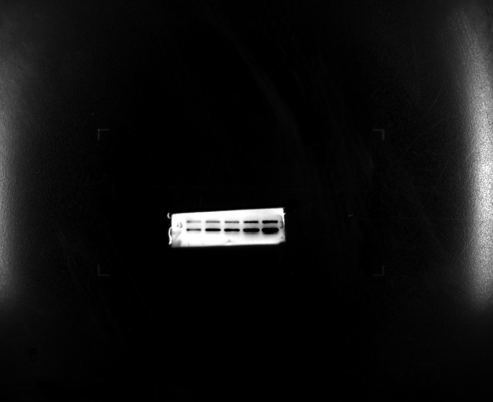


H3K13ac


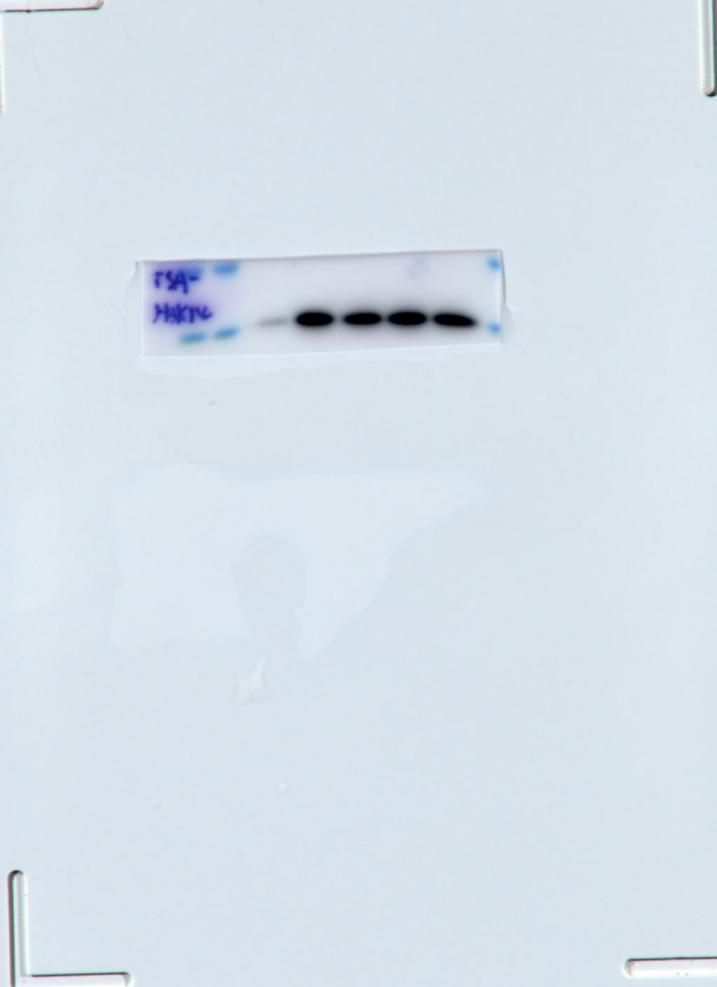


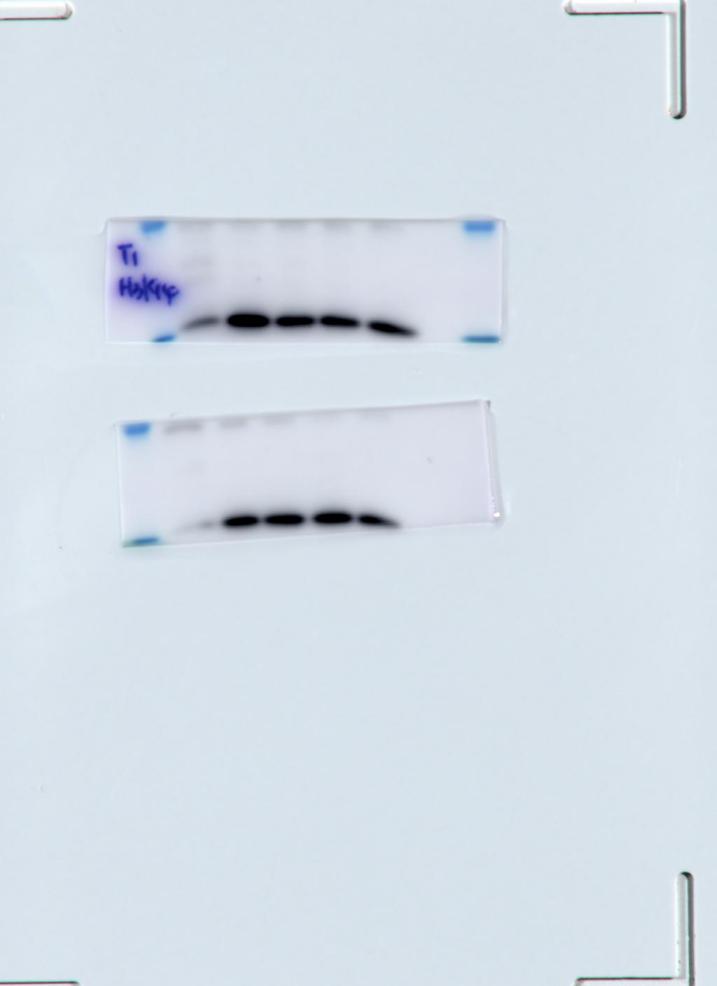


PCNA:


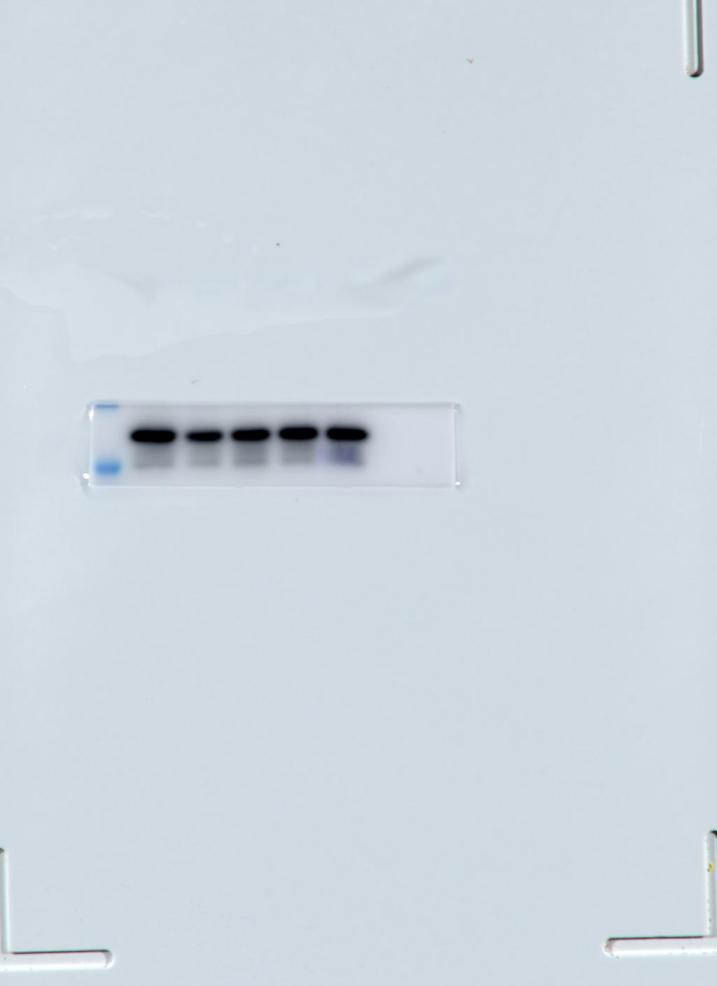


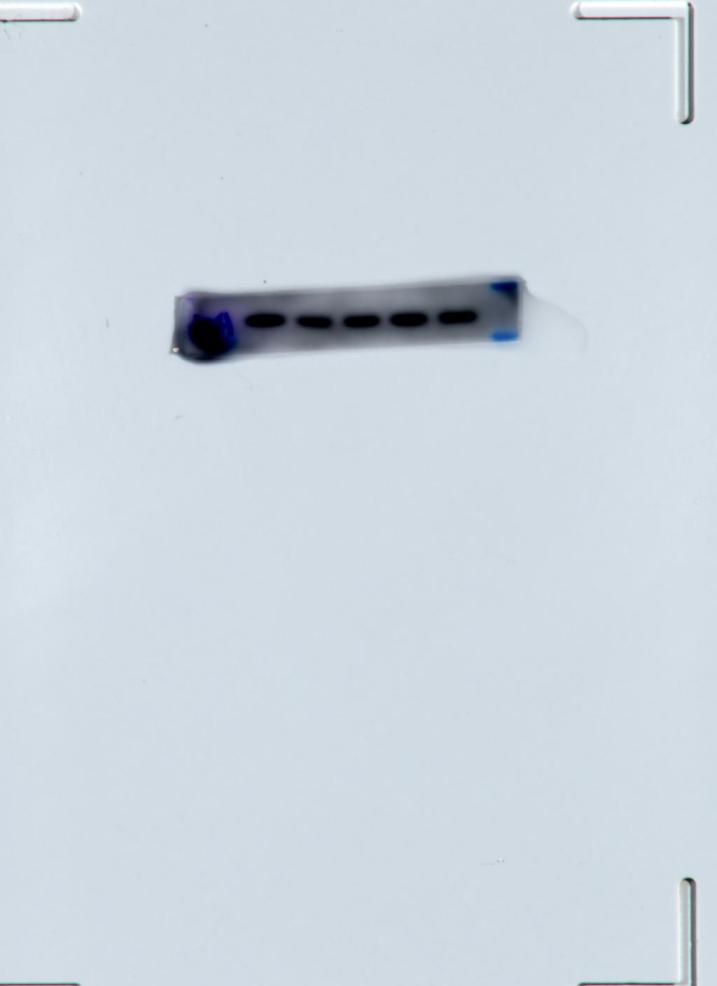


Figure 5J：

H3K14ac：


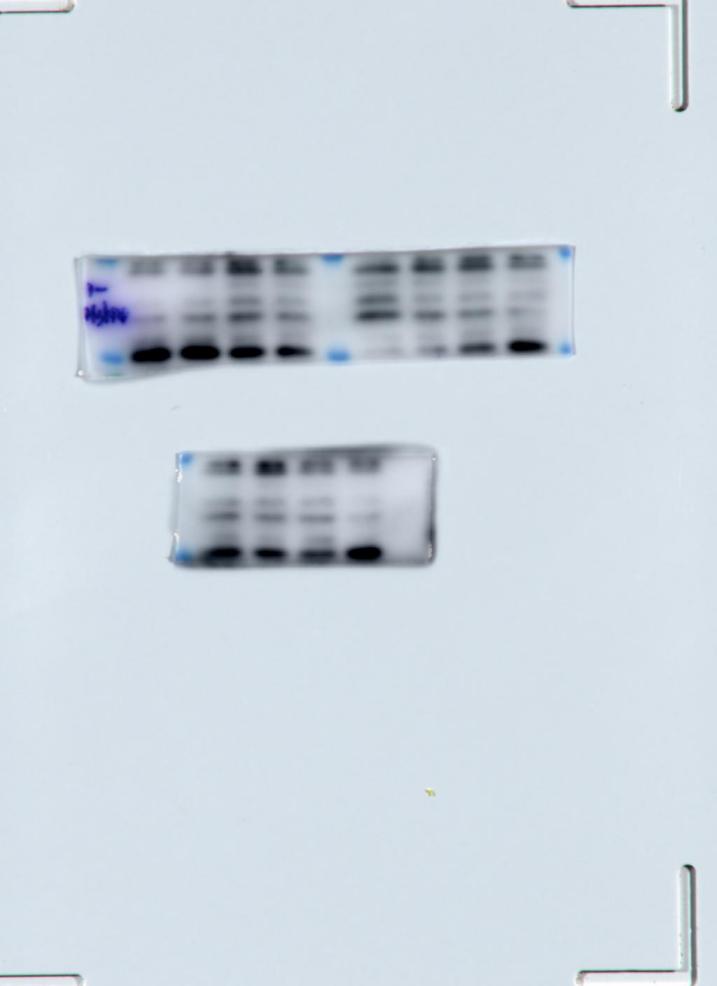

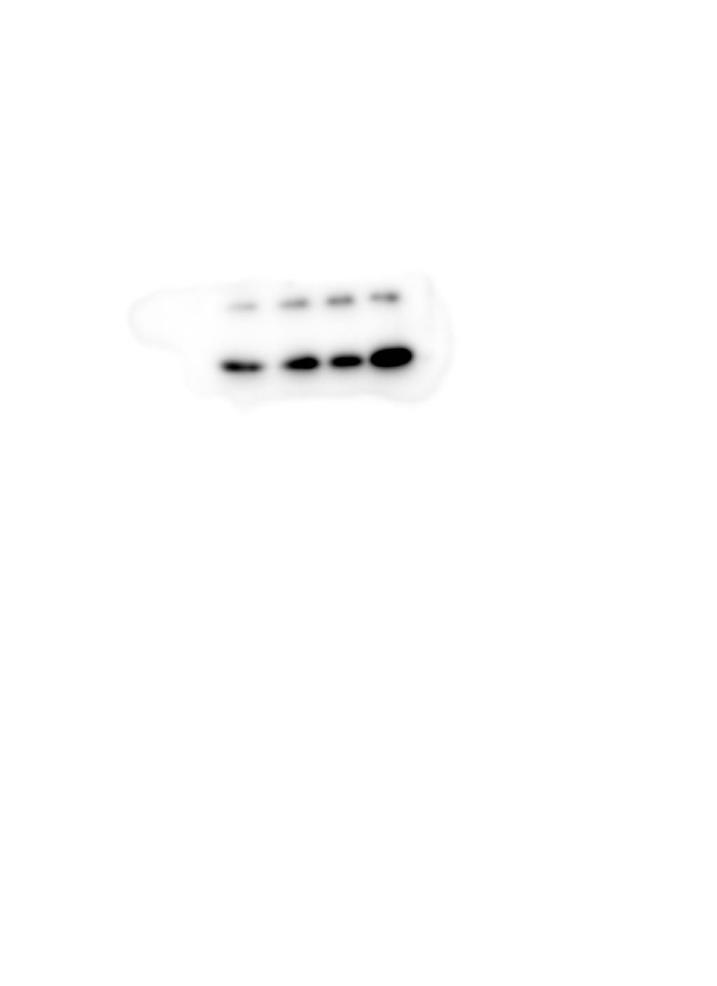


PCNA：


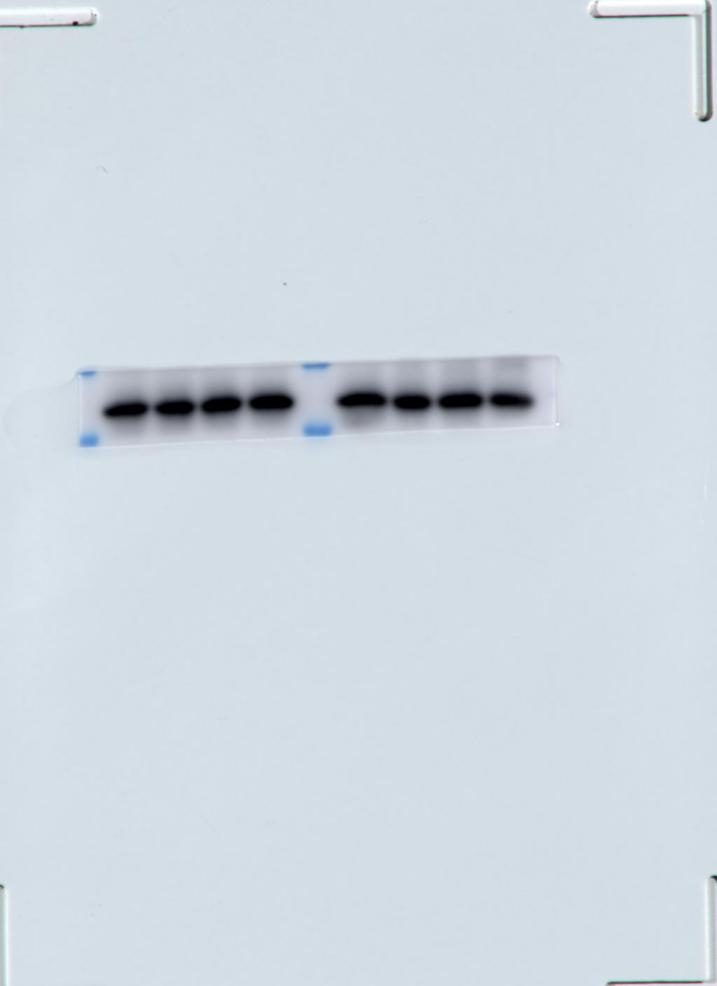


Figure 6F:

ILF3：


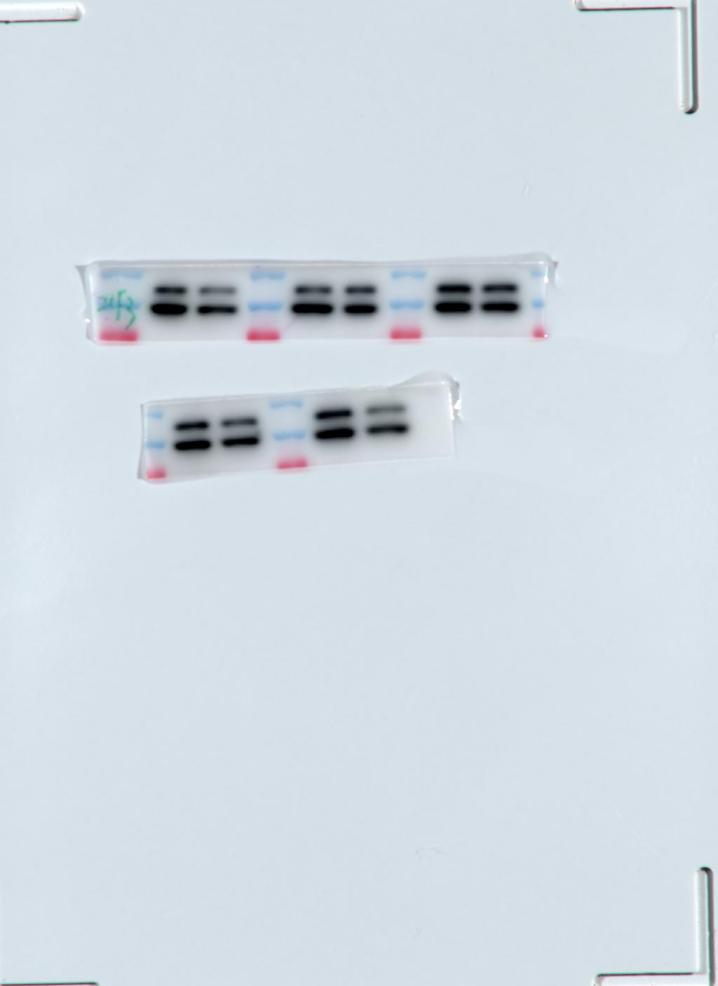


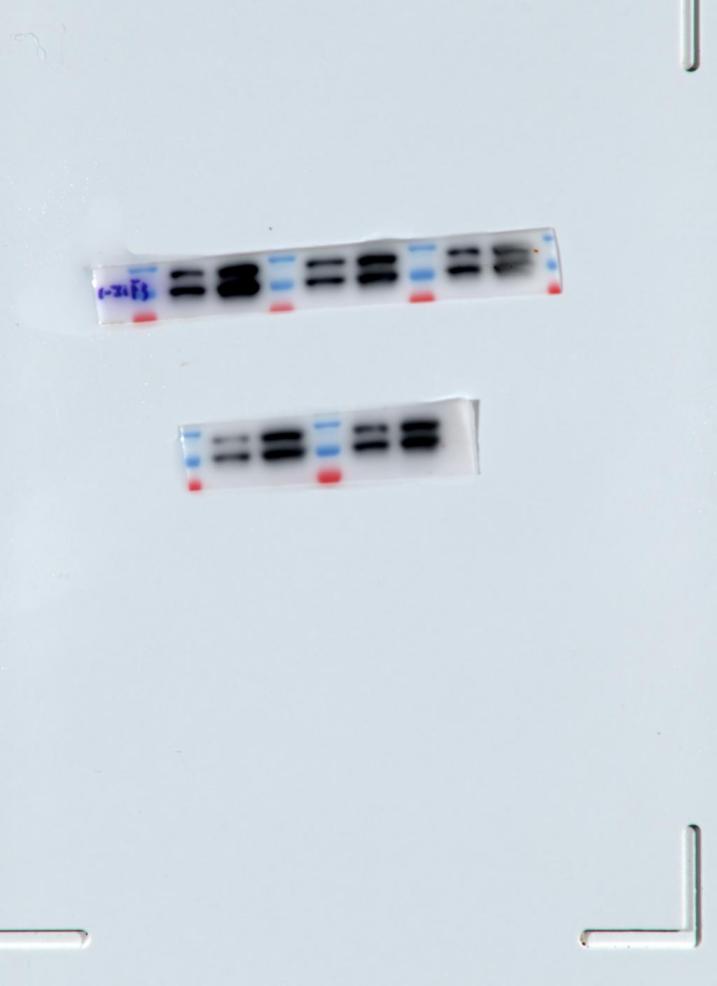


DEPTOR:


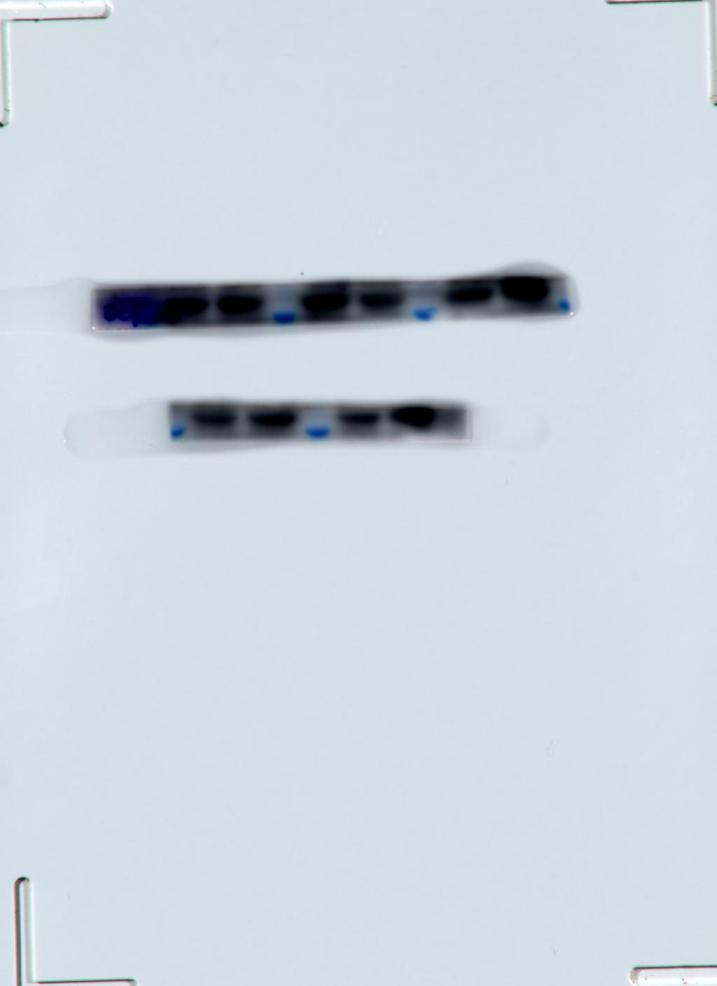

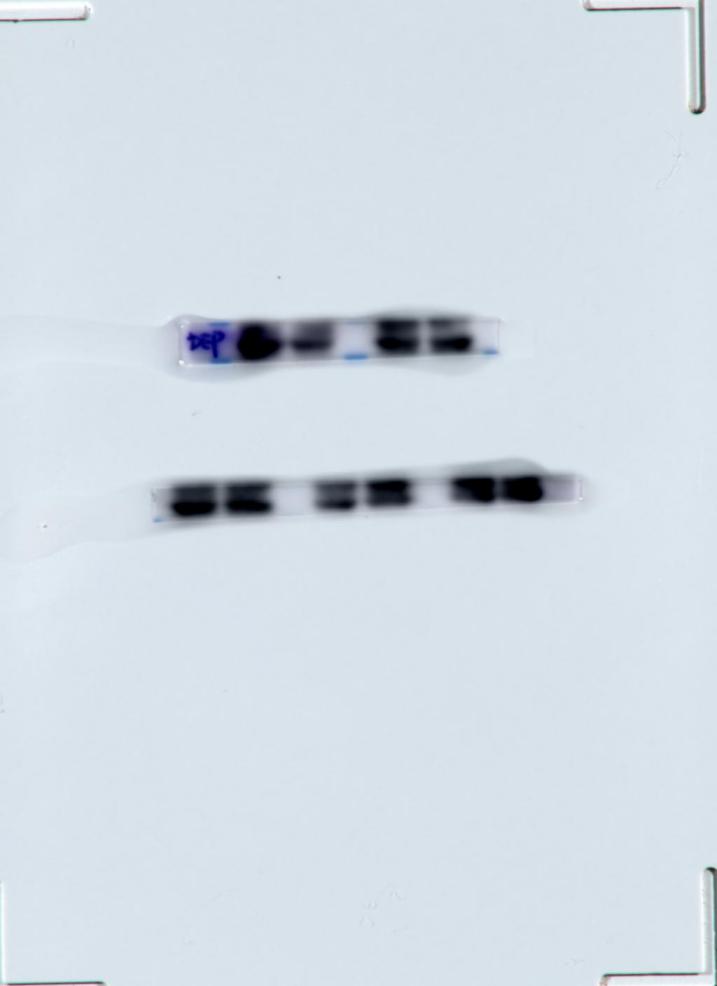


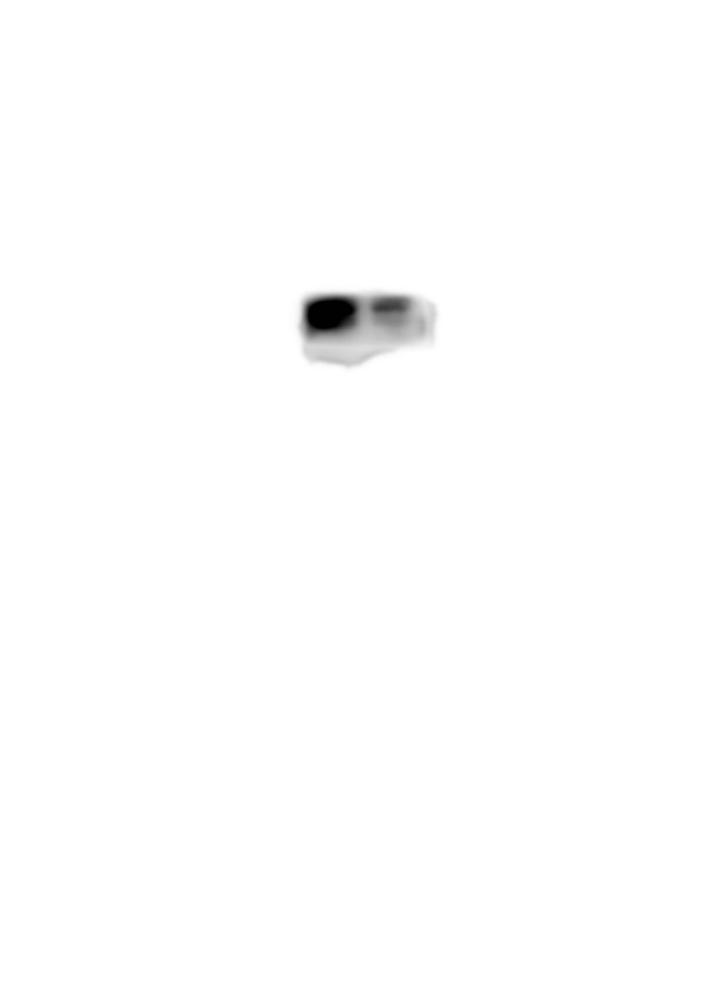


p-mTOR:


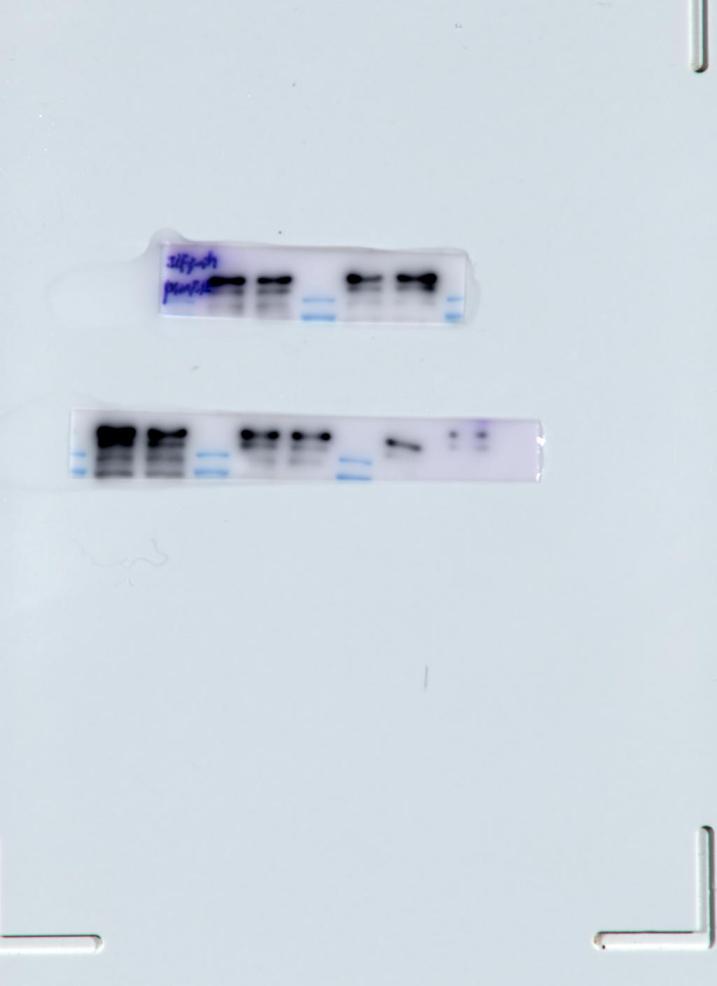


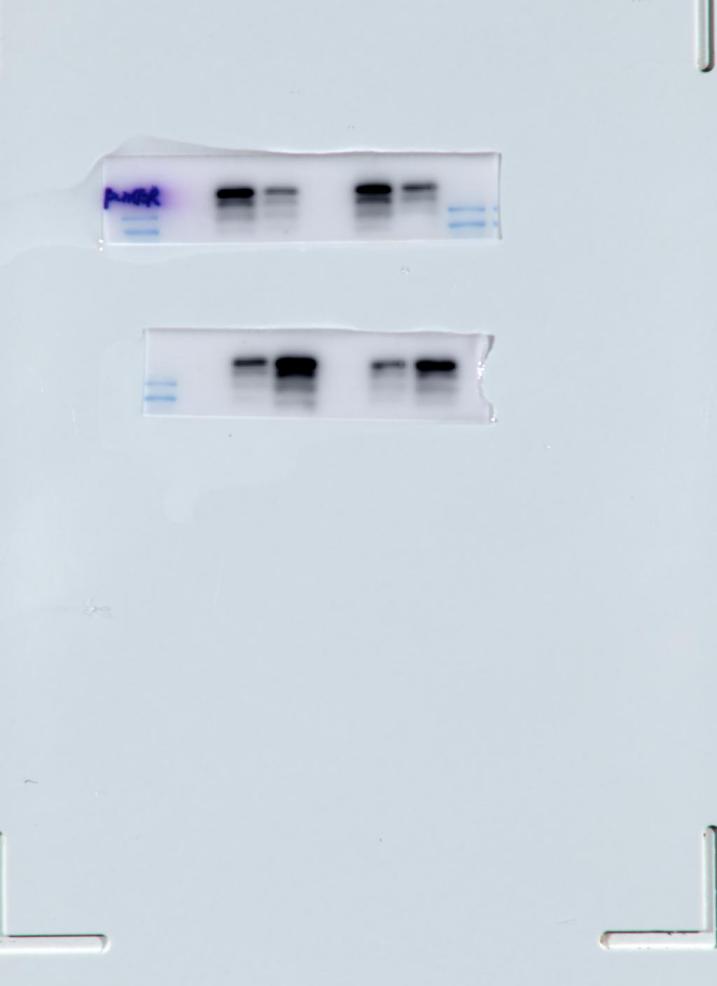


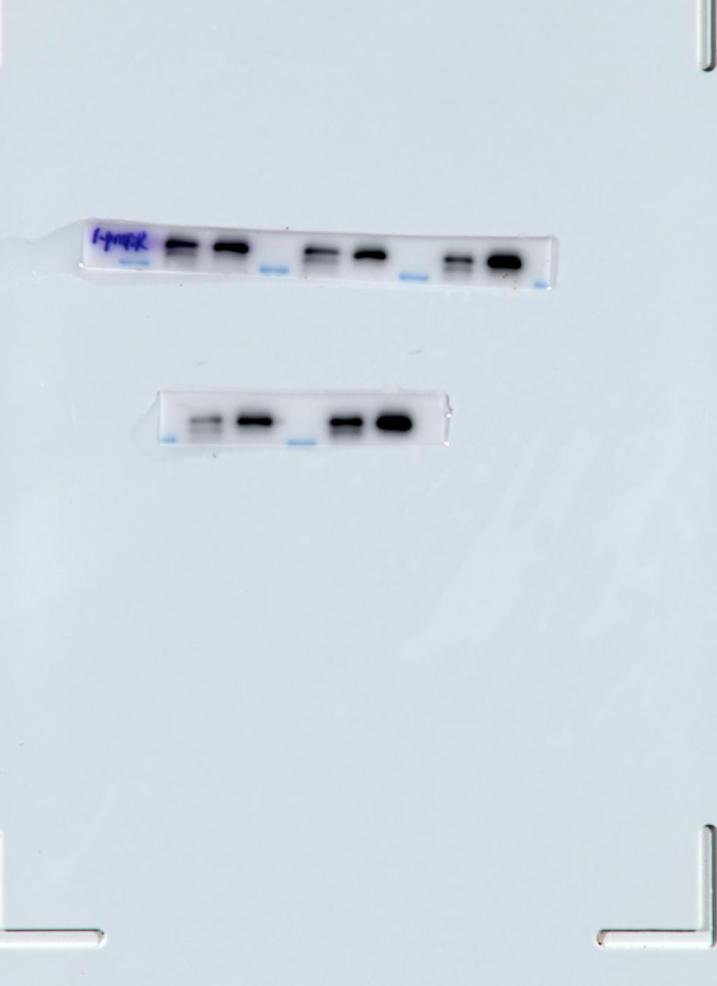


mTOR:


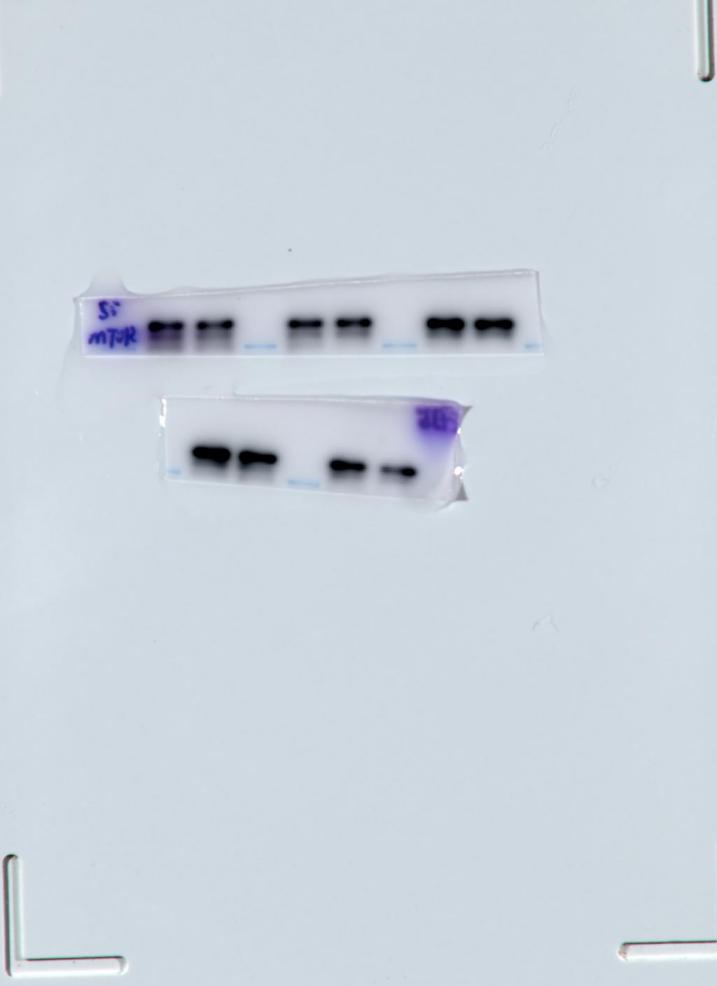


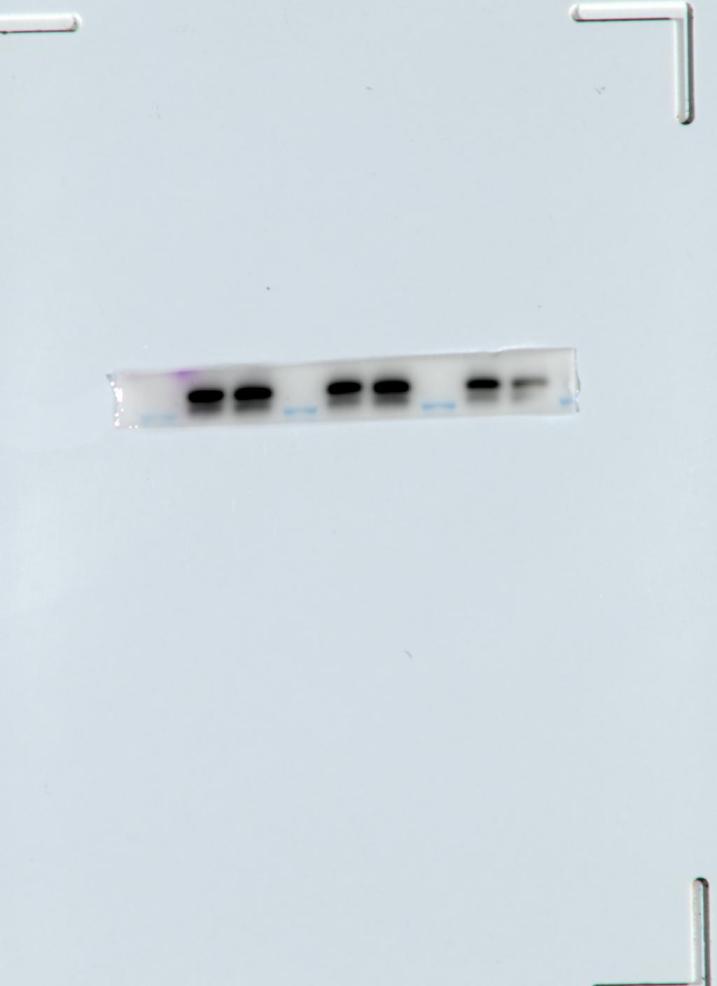


GAPDH:


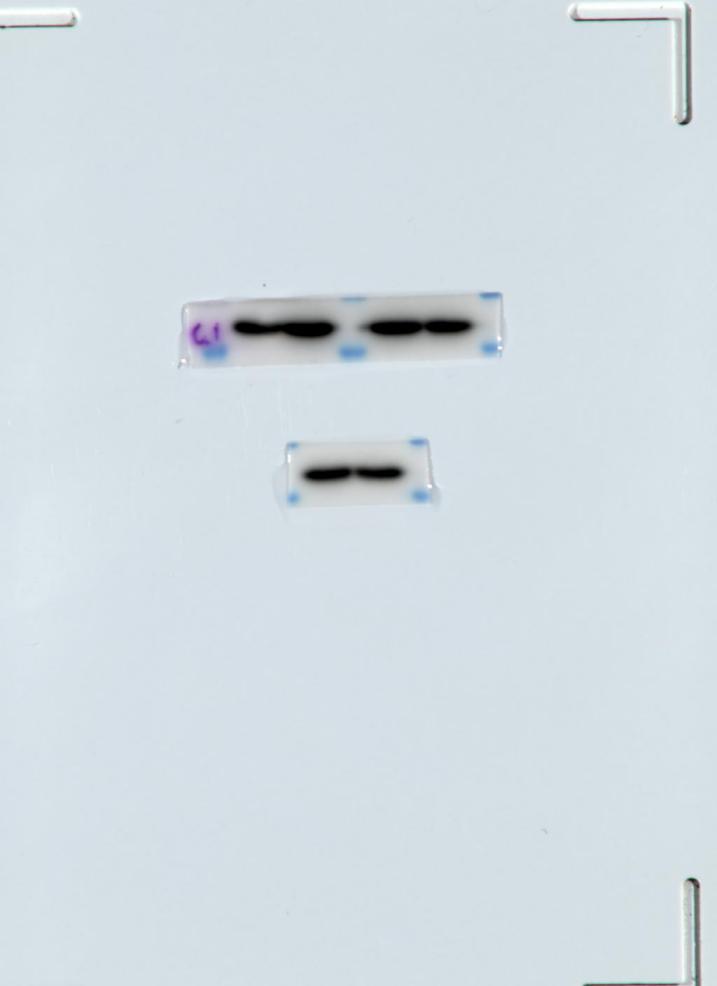


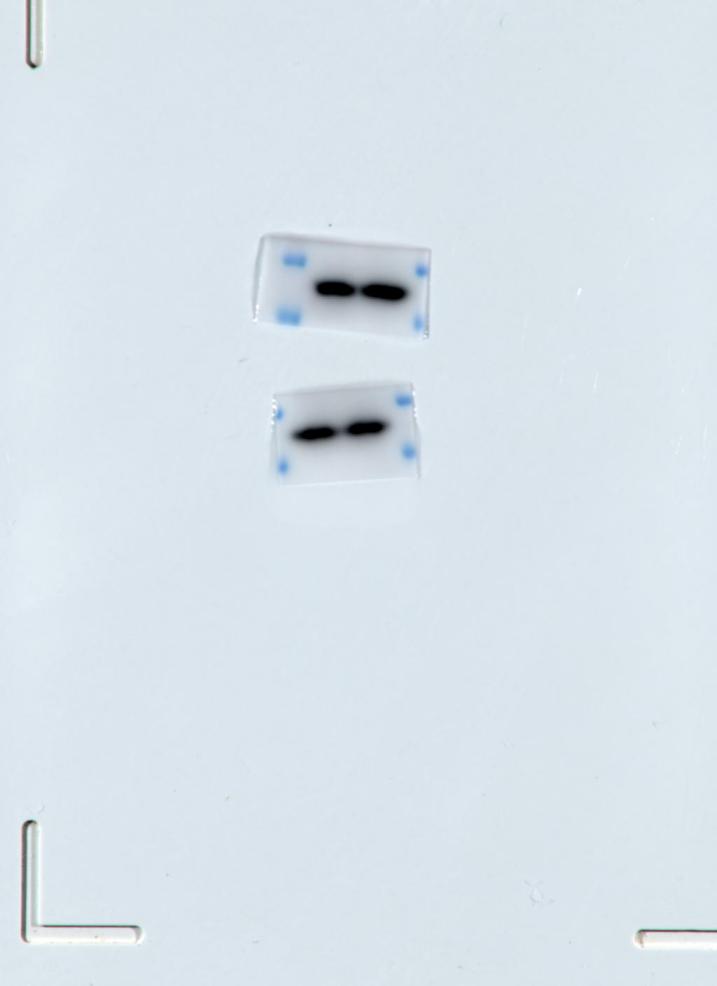


Figure 6G:

DEPTOR:

p-mTOR:

mTOR:

GAPDH

Fig 6H:

ILF3:

DEPTOR:

mTOR:

p-mTOR:

PD-L1:

SLC7A11:

GPX4:

GAPDH:

Figure6I:

ILF3:

mTOR：

p-mTOR:

PD-L1:

SLC7A11:

GPX4:

GAPDH:

Figure 7：

A:ILF3

B:GAPDH

Figure7H:

ILF3:

PD-L1:

GAPDH:

SigFigure 1
D:PD-L1

GAPDH:

SigFigure 2E:

ILF3:

SLC7A11:

GPX4:

GAPDH:
